# Structure Activity Relationship Studies around *DB18*, a Potent and Selective Inhibitor of CLK Kinases

**DOI:** 10.3390/molecules27196149

**Published:** 2022-09-20

**Authors:** Dabbugoddu Brahmaiah, Anagani Kanaka Durga Bhavani, Pasula Aparna, Nangunoori Sampath Kumar, Hélène Solhi, Rémy Le Guevel, Blandine Baratte, Thomas Robert, Sandrine Ruchaud, Stéphane Bach, Surender Singh Jadav, Chada Raji Reddy, Paul Mosset, Nicolas Gouault, Nicolas Levoin, René Grée

**Affiliations:** 1 Chemveda Life Sciences India Pvt. Ltd., #B-11/1, IDA Uppal, Hyderabad-500039, Telangana, India; 2 Jawaharlal Nehru Technological University Hyderabad, Kukatpally, Hyderabad-500 085, Telangana, India; 3 Department of Chemistry, Osmania University, Hyderabad 500007, Telangana, India; 4 Univ Rennes, PlateformImPACcell, BIOSIT, F-35000 Rennes, France; 5 Sorbonne Université, CNRS, FR 2424, Plateforme de criblage KISSf (Kinase Inhibitor Specialized Screening facility), Station Biologique de Roscoff, CS 90074, 29688 Roscoff Cedex, France; 6 Sorbonne Université, CNRS, UMR 8227, Integrative Biology of Marine Models Laboratory (LBI2M), Station Biologique de Roscoff, CS 90074, 29688 Roscoff Cedex, France; 7 Centre of Excellence for Pharmaceutical Sciences, North-West University, Private Bag X6001, Potchefstroom 2520, South Africa; 8 CSIR-Indian Institute of Chemical Technology, Uppal Road, Tarnaka, Hyderabad 500007, TS, India; 9 Univ Rennes, CNRS, ISCR (Institut des Sciences Chimiques de Rennes), UMR 6226, F-35000 Rennes, France; 10 Bioprojet-Biotech, 4 rue du Chesnay Beauregard, BP 96205, 35762 Saint Grégoire, France

**Keywords:** kinases, CLK-kinases, DYRK1A kinase, quinazoline, cytotoxicity, molecular modelling

## Abstract

Three series of our lead CLK1 inhibitor ***DB18*** have been designed, synthetized and tested against CLKs and DYRK1A kinases. Their cytotoxicity was subsequently measured on seven representative cancer cell lines. Guided by docking experiments, we focused on the less constrained part of the scaffold, and showed that drastically different substituents can be tolerated here. This work ended with the discovery of another promising derivative **12g**, with IC_50_ = 0.004 µM in the inhibition of *Hs*CLK1 and IC_50_ = 3.94 µM for the inhibition of *Hs*DYRK1A. The SAR results are discussed in the light of extensive molecular modeling analyses. Finally, a kinome scan (463 human kinases) confirmed the outstanding selectivity of our lead compound ***DB18***, suggesting that this scaffold is of prominent interest for selective CLK inhibitors. Altogether, these results pave the way for the development of inhibitors with novel selectivities in this family of kinases.

## 1. Introduction

The human kinome comprises 538 kinases playing essential functions by catalyzing protein (518 enzymes) or lipid (20) phosphorylation (encoded by almost 3% of the 19,000 human genes) [1]. Protein kinases (PKs) are involved in the regulation of numerous cellular processes, such as metabolism, cell cycle progression, cell adhesion, vascular function and angiogenesis, often in response to an external stimulus. Aberrant kinase activity (e.g., by hyperactivation, or genetic alterations such as mutations and translocations) plays an important role in the pathogenesis of many diseases including neurodegenerative, cardiovascular, autoimmune and inflammatory diseases as well as in numerous cancers. Over the past three decades, this family of enzymes has emerged as one of the most important suppliers of drug targets and it is estimated that one-quarter of the current drug discovery efforts worldwide are focused on the PKs [2]. There are already 71 American Food and Drug Administration (FDA)-approved small molecule PK inhibitors (http://www.brimr.org/PKI/PKIs.htm, accessed on 29 May 2022) and more than 80 kinases inhibitors are marketed around the world [3]. Moreover, about 175 different orally effective protein-kinase inhibitors are in clinical trials worldwide [2]. Most of these drugs and lead inhibitors of protein kinases are used for the treatment of malignancies. As reported by Sung et al. [4], an estimated 19.3 million new cancer cases and almost 10.0 million cancer deaths occurred in the world in 2020. Cancer is the second-leading cause of death worldwide. The same authors estimated that the global cancer burden is expected to be 28.4 million cases in 2040. This expected rise should motivate the discovery of new potent therapeutic compounds targeting cancer-related kinases.

CLKs (Cdc2-like kinases) and DYRKs kinases (dual specificity tyrosine-phosphorylation-regulated kinases) are known for their important biological properties [5,6]. Over the last decade, CLKs have been explored as attractive targets for their pharmacological inhibition. CLKs are members of the CMGC (for CDKs, MAP kinases, GSK and CDK-like kinases) group of kinases and part of the so-called group of splicing kinases alongside SRPKs (serine-arginine protein kinases), PRP4K/PRPF4B (pre-mRNA splicing 4 kinase), and Topoisomerase I [5,7]. Splicing kinases regulate the alternative splicing process through the phosphorylation of splicing factors such as SR proteins (serine-arginine rich protein family) [8]. They have been found implicated in the tumorigenesis process and dysregulated in numerous cancers, making them attractive pharmacological targets [9]. Hence, recently, CLKs have attracted interest with the development of a potent inhibitor, SM08502, actually in phase I clinical trial for the treatment of advanced solid tumors [10]. Many of the marketed drugs targeting PKs are multikinase inhibitors [2]. Efforts to identify CLK chemical inhibitors of pharmacological interest tend to identify molecules with high efficiency and selectivity to meet the two criteria required for clinical development of a drug: efficiency and harmlessness. Selective inhibition of a kinase remains clearly a challenging problem. Even if a few CLKs selective inhibitors have been reported recently, [11,12] more selective drugs, as second-generation molecules, are awaited. Identification of selective inhibitors targeting CLKs is a non-trivial task since CLKs are in particular structurally very closely related to DYRKs and to homeodomain-interacting protein kinases (HIPKs) leading to inhibitor cross reactivity [13]. We have recently discovered a molecule called ***DB18*** which exhibit very attractive properties: it is a potent inhibitor of the human CLK1, CLK2 and CLK4 kinases in the 10–20 nM range [14]. On the other hand, it is not active at 100 µM on the human DYRK1A kinase, thus demonstrating a remarkable selectivity (in the 10^5^ range) between these kinases which are very close from a structural point of view. Furthermore, extensive molecular modelling and molecular dynamic simulations have allowed us to propose a rationale to this selectivity of CLKs vs. DYRK1A [14].

**Figure 1:**
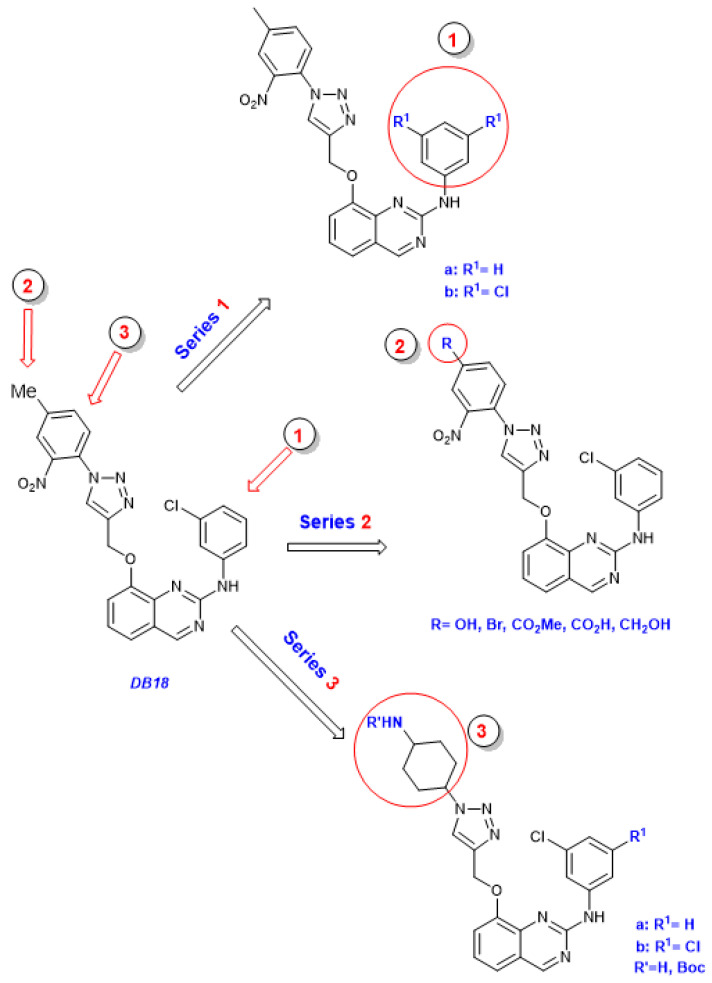
Design of the three series of target molecules.

Our previous molecular modelling data (for precise docking see [14]) clearly indicated that the 2-anilino quinazoline scaffold played a key role in anchoring this molecule in the ATP binding site of the CLK1 kinase with, in particular, strong hydrogen bonds with Leu 244. Therefore, this part was kept constant during these second-generation studies and we selected three other parts for the modulations. The design of our new molecules is indicated in Figure 1. In the first series, we explored the possible role of the *meta* chlorine in the anilino group and therefore we designed two molecules: one without chlorine and a second with two chlorine atoms in *meta*-*meta’* positions. In the second series, the replacement of the terminal methyl group of the *o*-nitro toluene substituent by bromine or polar groups such as OH, acid or alcohol was considered. In the third series, the goal was to explore the possibility of changes in the chain linked to the triazole moiety. Further, based on preliminary molecular modelling studies, we replaced the aromatic group by a cyclohexyl substituent with the introduction of a polar amino group in the upper part of the targets.

Thus, the goals of this study are: 1. to describe the synthesis of these target molecules; 2. to report their activity on the CLK kinases and, for the most promising compounds, also their action on the DYRK1A kinase; 3. to present their cytotoxicities against a representative panel of seven cancer cell lines; and 4. for the most active inhibitors, to rationalize their action on CLKs by extensive molecular modelling studies. In a final stage, the kinome selectivity profile of ***DB18*** against a panel of 463 human kinases will be presented.

## 2. Results and Discussion

### 2.1. Chemical Syntheses

**Scheme 1:**
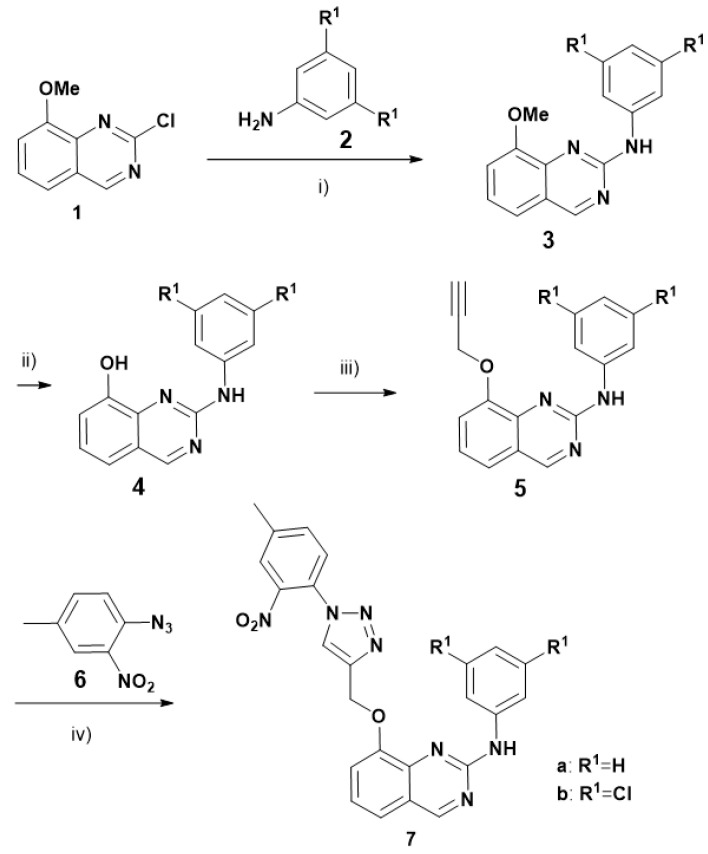
Synthesis of the first targets, the anilino-2-quinazoline **7.** Reagents andconditions; (**i**) aniline **2a** or 3-chloroaniline **2b**, Pd(OAc)_2_, BINAP, Cs_2_CO_3_, 1,4-dioxane, 100 °C, 16 h, **3a** (52%), **3b** (61%); (**ii**) BBr_3_, DCM, 16 h, RT, **4a** (49%), **4b** (54%); (**iii**) propargyl bromide, K_2_CO_3_, acetone, reflux, 6 h, **5a** (47%), **5b** (68%); (**iv**) **6** (2 equiv), CuSO_4_.5H_2_O, sodium ascorbate, RT, 16 h, **7a** (58%); **7b** (54%).

The synthesis of our first target molecules **7** uses the same route as for the compound ***DB18*** (Scheme 1). It starts from the known 2-chloro-8-methoxyquinazoline **1** [14], which by reaction with aniline **2a**, or the 3,5-dichloroaniline **2b**, in the presence of a palladium catalyst gave the 2-substituted anilinoquinazolines **3a** and **3b** in fair yields. The 8-hydroxyquinazolines **4a** and **4b**, obtained from intermediates **3a** and **3b** by the demethylation of the methoxy group in the presence of BBr_3_, reacted with propargyl bromide in the presence of three equivalents of potassium carbonate in acetone under reflux for 8 h to give the key intermediates **5a** and **5b**. The presence of propargyl groups in compounds **5** was evidenced by the presence of a doublet at δ 5.01 and a triplet at δ 3.65 ppm in their ^1^H-NMR spectrum. The ^13^C-NMR spectrum also confirmed the presence of *O*-propargyl groups in **5** by showing the carbon peaks at δ 56.46, 78.91 and 78.58 ppm. Then, the target molecules **7a** and **7b** were obtained in good yields, using a click reaction [15,16,17,18] between the alkynes **5** and the azide **6 [19]**. The triazoles were evidenced by all spectral data and in particular the disappearance of propargylic groups and appearance of the triazole protons at 8.79 ppm. If we take **7a** as a representative example we observe the NH at 9.88 ppm, the triazole proton at 8.09 ppm, the CH_2_ at 5.48 ppm, with the methyl of the tolyl group at 2.53 ppm. The characteristic proton in position 4 of the quinazoline appears at 9.29 ppm. The other signals belong to the aromatic/heteroaromatic protons. In ^13^C NMR we identify the (C-H) carbon signal of triazole at 125.8 ppm, the methylene group at 62.1 ppm and the methyl of the tolyl group at 20.3 ppm. The signal of the carbon in position 4 of the quinazoline is at 161.8 ppm. The other signals belong to the aromatic/heteroaromatic systems. All these data have been confirmed by extensive 2D experiments and similar results have been obtained for the other quinazolines.

**Scheme 2:**
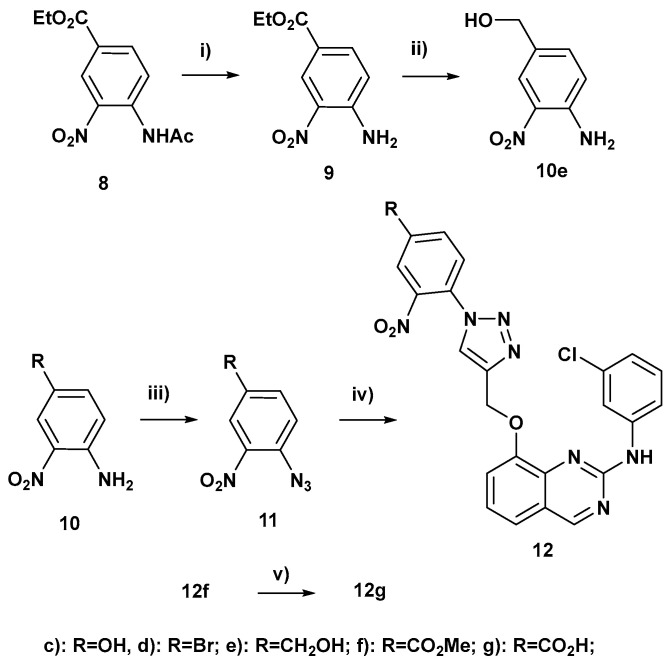
Synthesis of the second targets, the anilino-2-quinazolines **12**. (**i**) H_2_NNH_2_.H_2_O, CH_3_CO_2_H (1:1, 8.7 equiv), HO(CH_2_)_3_OH, 90 °C, 22 h (91%); (**ii**) dry LiCl (19 equiv), NaBH_4_ (19 equiv), EtOH/MeOH (10:1), 45 °C, 12 h [“in situ” formation of LiBH(OMe)_3_] (35%); (**iii**) HCl, H_2_O, EtOH, NaNO_2_, 0 °C then NaN_3_, H_2_O, **11c** (97%), **11d** (used crude for next step), **11e** (47%), **11f** (used crude for next step); (**iv**) CuSO_4_.5H_2_O (0.15 equiv), Na ascorbate (0.47 equiv), urea (0.53 equiv), THF/DMSO/H_2_O (10:1.75:1) + 13 (1.0 equiv), rt, 4.5 h, **12c** (99%), **12d** (56%), **12e** (81%), **12f** (23%); (**v**) **12g** (1 equiv), LiOH (2 equiv), MeOH, 45 °C, 16 h, (52%).

The synthesis of the second series of molecules is presented in Scheme 2.

**Scheme 3:**
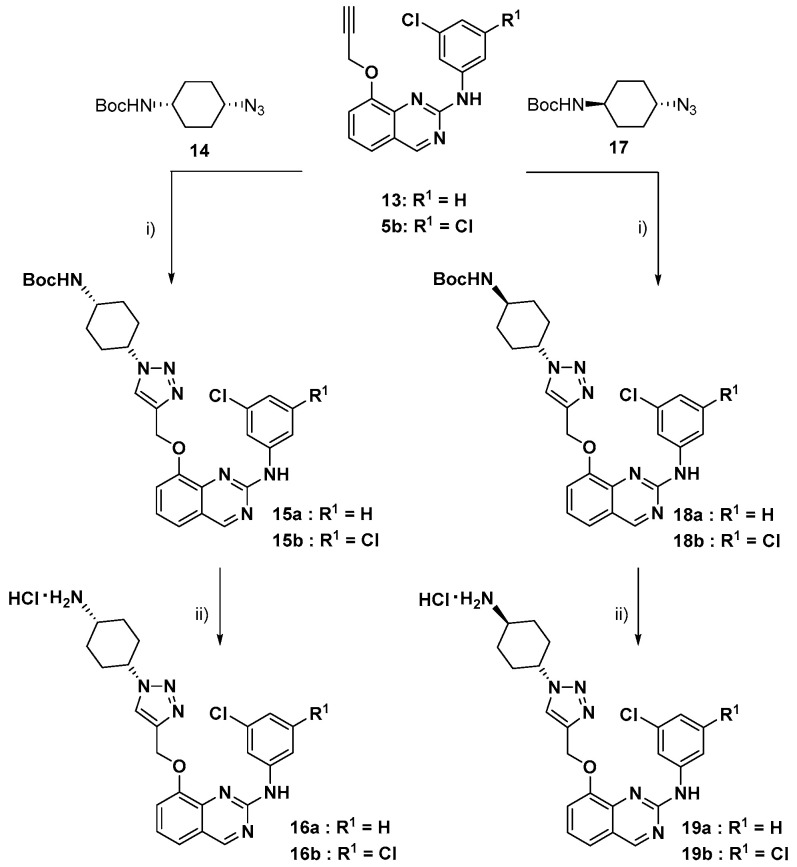
Synthesis of the third targets, the anilino-2-quinazolines **16**–**19**. Reagents andconditions: (**i**) from **14**, t-BuOH/H_2_O (1/1), CuSO_4_.5H_2_O, sodium ascorbate, RT, 16 h, **15a** (67%) and **15b** (59%); from **17**, **18a** (56%) and **18b** (59%); (**ii**) 4 M HCl in dioxane, RT, 16 h, **16a** (76%), **16b** (74%), **19a** (72%), **19b** (74%).

The starting nitroanilines **10** are known compounds, except **10e** which was prepared in two steps from commercially available **8**. The deprotection of **8** to the amine **9** was followed by reduction, under carefully controlled conditions, to the desired molecule **10e**. All these nitroanilines **10** were transformed into the corresponding azides **11**, which gave the targets **12c**–**12g** through the same click-type reaction as above with alkyne **13**. The synthesis of the last series of molecules followed a similar strategy, as indicated in Scheme 3. Reaction of previously described [14], propargylic derivative **13**, with a single chlorine atom in *meta* position of the aniline, with known [20] *N*-Boc-protected *cis* 4-amino cyclohexylazide **14** gave in good yield the triazole **15a**. A final deprotection step gave the first target molecule **16a**. Then, starting from **13**, similar reactions with the, also known [20], *trans* isomeric azide **17** gave first the triazole **18a** and after deprotection the second target **19a**. Next, the same series of reactions were performed starting from the dichloro-analogue **5b**, affording the compounds **15b** to **19b**. All these molecules have spectral and analytical data in agreement with their structures, as indicated in the experimental section and Supplementary Materials.

### 2.2. Kinase Inhibition Studies

All molecules prepared during this study have been first screened against a short panel of kinases to perform a primary evaluation of their bioactivity. Our compounds were tested at 10 µM and 1 µM on eight disease-related serine/threonine protein kinases including cyclin-dependent kinases (CDK5/p25, CDK9/cyclinT), proviral integration site for Moloney murine leukemia virus kinase (PIM1), glycogen synthase kinase-3 beta (GSK3β), CDC-like kinase 1 (CLK1), dual specificity tyrosine phosphorylation regulated kinase 1A (DYRK1A), casein kinase 1-ε (CK1ε) and the mitotic protein kinase Haspin. The results are reported in Table S1. For the compounds demonstrating significant activities, more in-depth studies have been performed on human CLKs and DYRK1A kinases. Corresponding results will be presented by a series of designed molecules and compared to the data obtained earlier with our lead molecule ***DB18*** [14].

In the first series, we explored the role of the substituents on the aniline linked to the quinazoline (Table 1). The ***DB18*** had a chlorine in *meta* position; **7a** had no substituent, and **7b** had two chlorine atoms in *meta*-*meta*’ positions. The unsubstituted compound **7a** proved to be 100 times less active than our lead compound on the human CLK1 (IC_50_: 1100 and 11 nM respectively). Therefore, it was not explored further on the other CLK kinases and on DYRK1A. On the other hand, the dichloro molecule **7b** proved to be just slightly less active than ***DB18*** on the *Mm*CLK1 and thus its activity was studied on the other human CLK kinases. It showed a profile similar to our reference compound with a small decrease in affinity for the four kinases and was completely inactive on the human DYRK1A. All together these results indicated that a *meta*-chloro substituent on the aniline group is required for activity but that there should be some flexibility in this binding pocket since the dichloro remained significantly active and selective. However, at this stage, the *meta*-chloroaniline group used for ***DB18*** appeared as the best compromise.

In the second series of molecules, we explored the possibilities of modifications in the upper part of the ***DB18*** lead compound by replacement of the methyl group through polar atoms and/or functional groups. The results of the biological studies are reported in Table 2.

The introduction of bromine led to a compound (**12d**) with low activities on the human CLK1 kinases. Similarly, the corresponding phenolic derivative **12c** demonstrated poor IC_50_ for human CLK1. Therefore, these two molecules were not explored on the other CLK kinases, as well as on DYRK1A. Next, functional groups were introduced in this *para* position of the aromatic group. The alcohol **12e** showed low affinities (IC_50_ at 150–200 nM) on the human CLK1 and CLK4 kinases. The affinity was slightly higher (50 nM) on CLK2 and very low (7.5 μM) on CLK3 but a significant activity was also observed on DYRK1A (2.3 μM). The last designed compounds were the ester **12f** and the acid derivative **12g**. The activity of the ester **12d** was low on the human CLK1, in the 0.2–0.3 μM range and therefore it was not further characterized, neither on the other CLK kinases, nor on DYRK1A. On the contrary, the corresponding acid **12g** proved to be very active (4 nM), on the human CLK1. For *Hs*CLK2 the IC_50_ was found at 28 nM and for *Hs*CLK4 at 55 nM. As often found in these series, the IC_50_ is higher for *Hs*CLK3 with a 339 nM value. Finally, this molecule exhibited also a low, but significant, affinity for *Hs*DYRK1A with an IC_50_ of 3.94 μM. Altogether, these results demonstrated that some modifications were possible in the upper part of the reference compound. In particular, the acid **12g** demonstrated IC_50_ values very similar to ***DB18*** on the human kinases CLK1 to CLK4. However, if we consider the selectivity CLK1 versus DYRK1A, **12g** is less selective than ***DB18*** which exhibited no action at 100 μM on this particular kinase.

In the third series, we explored the possibility to replace the central aromatic group by cyclohexyl substituents. Based on some preliminary molecular modelling studies it appeared useful to add a primary amine on the cyclohexyl in order to obtain some extra hydrogen bond interactions on the upper part of the kinases. The *N*-Boc protected intermediates **15** and **18** proved to be completely inactive in the preliminary screening assays on CLKs and other kinases (Table S1) and therefore were not studied further. Thus, only the molecules **16** and **19** have been considered and the results are given in Table 3. 

These molecules differ both by the *cis*- and *trans*-stereochemistry of the cyclohexyl substituents and by the number of chlorine atoms (**1** or **2**) on the aniline group. The four derivatives **16a**, **16b**, **19a** and **19b** demonstrated a low, but significant activity on the human DYRK1A kinase, in the 2–5 μM range. Thus, two molecules **16a** and **16b** were selected for evaluation on the four human CLKs. Interestingly, their selectivity profiles were similar to ***DB18*** (good affinities for CLK1, CLK2 and CLK4 with lower activity on CLK3) albeit with a slight loss in efficacy compared to our lead molecule. Altogether, these results indicated that there is some space in this central part of the CLK kinase active pockets to adjust for substituents like cyclohexyl groups, which are bulkier than an aromatic system. However, with the compounds **16** and **19**, there was a considerable loss in selectivity regarding DYRK1A. For these compounds, the IC_50_ values were in the low micromolar range althoughin the case of ***DB18***, no inhibition was observed at 100 μM. These results indicate that, in spite of significant structural changes, modifications in this central part of our lead compound were still possible while keeping a similar global profile of CLKs’ inhibition. However, this occurred with a significant loss of selectivity for CLKs vs. DYRK1A.

The most significant results from these studies will be discussed by using molecular modelling methods in the next part of this paper. 

### 2.3. Structural Investigation by Molecular Simulations

**Figure 2:**
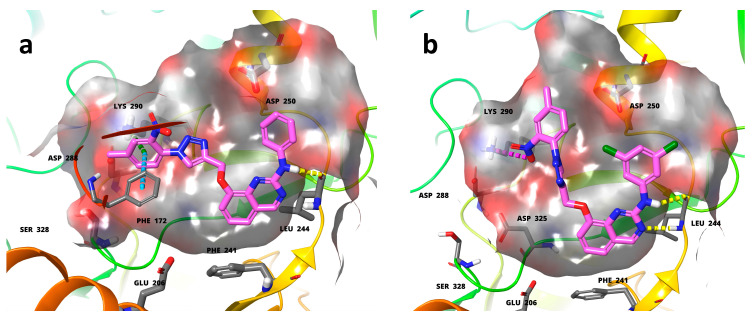
(**a**) **7a** in CLK1 cavity; (**b**) **7b** in CLK1 cavity; the ligands **7a** and **7b** are shown as thick residues in color-faded magenta. Color coding: oxygen-red, nitrogen-blue, carbon-grey (in case of protein). Backbone, side chain hydrogen bonds are shown as yellow dotted lines, π-π stackings are given in light blue dotted lines and and π-cation showed in green dotted lines whereas, charged interactions are provided in pink dotted lines. To focus the interactions of active site residues with ligands 6.0, Å surface area of protein has been shown in the figures.

The first series constituted a brief exploration of the aniline part of our lead ***DB18***. It showed that the *meta*-chloro position has a huge effect on CLK1, since ***DB18*** is 100 times more potent than the non-substituted analogue **7a** (Table 1). Interestingly, the *meta*-*meta’*-dichloro **7b** showed an intermediate activity. We performed thorough molecular modelling investigations in order to understand these results (See Supplementary Materials), and we present a summary of them in the following. The binding mode of **7a** and **7b** have significant similarities to that already described for ***DB18*** [14]. The anilinoquinazoline is deeply buried in the active site, stacked with F241, and forming two key hydrogen bonds with the backbone amide of L244 (Figure 2).

The nitrophenyl ring is stacked between the F172 and K290 through π-π and π-cation network. The orientation of nitrophenyl and triazole ring are altered in the pocket due to absence of *meta* chlorine in **7a**, because of the lack of intramolecular interactions (Figure 2a). The anilinoquinazoline of **7b** is also involved in two backbone hydrogen bonds with L244 amide (Figure 2b). The nitrophenyl core is accommodated in the pocket like ***DB18*** and its 2-nitro group formed electrostatic interactions with the primary amine of K290 residue. Thus, from the molecular docking experiments, the binding behaviour of **7a** and **7b** in the CLK1 pocket are identified as slightly different. For both inhibitors, the 2-anilino quinazoline occupy exactly the same pocket, with a RMSD of 2.5 Å, while the triazolo phenyl rotated along the phenol axis. According to molecular simulations, the higher affinity of ***DB18*** compared to **7a** results from a combination of several features: (i) an increase in van der Waals interactions with the protein due to the size of the chloro substituent, (ii) a strong intramolecular halogen/π interaction which limits the entropic cost of ligand binding, (iii) a greater stabilization of the protein active site.

Docking, confirmed by molecular dynamics, revealed that the active conformation of ***DB18*** is maintained by several intramolecular interactions. In addition to the strong halogen/π bond, a CH:O interaction restricts phenyltriazole movements (Table 4). This stabilization of the ligand allowed a stronger hydrogen bond with the key residue L244. The anilinoquinazoline forms two hydrogen bonds respectively during 54 and 60% of the simulation for **7a**, andthe same atoms form two hydrogen bonds respectively during 89 and 93% of the simulation for ***DB18***.

As far as the protein is concerned, the whole CLK1 is globally a little more stabilized by the binding of ***DB18*** as compared to **7a** (RMSD of 2.4 vs. 2.6 Å). Particularly, the Nt-domain is slightly more structured (Figure S1).

Since the anilino group is only partially buried in the active site, the dichloro analogue **7b** is penalized by the solvent exposure of the hydrophobic *meta’*-chloro substituent, which makes no interaction with the protein (Figure 2b).

Because of its low affinity for CLK1, **7a** was not examined further, but we determined the profile of ***DB18*** and **7b** for the other CLKs and DYRK1A kinases. As we already mentioned for ***DB18***, [14] the binding mode of our inhibitors is very similar for the four CLKs. So, it was not unexpected that we observed a similar activity profile of ***DB18*** and **7b** for CLK1, CLK2 and CLK4. The huge drop of affinity for CLK3 and DYRK1A was already analyzed in details for ***DB18***, and imputed to the inability of the enzymes to adapt their conformation to the inhibitors by an induced-fit mechanism, as CLK1, -2 and -4 do [14].

**Figure 3:**
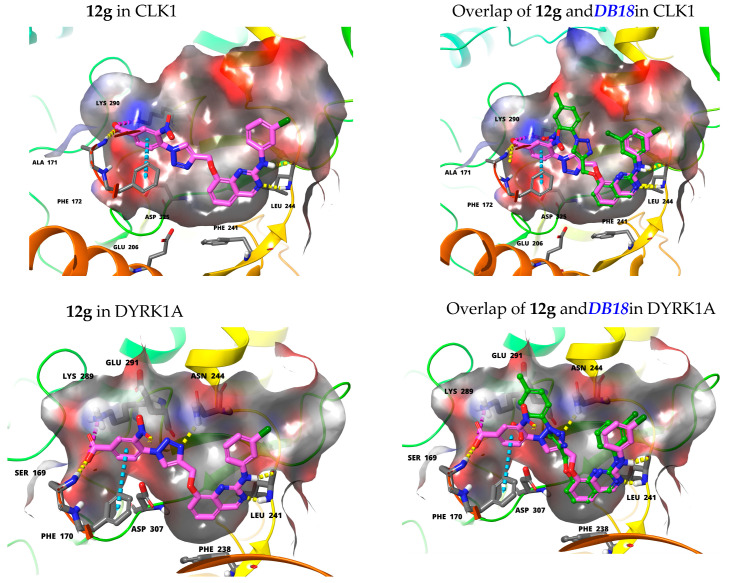
(**a**) **12g** in CLK1 cavity; (**b**) overlap of **12g** with ***DB18*** in CLK1 active site; (**c**) **12g** in DYRK1A cavity; (**d**) overlap of **12g** with ***DB18*** in DYRK1A active site. The ligands ***DB18*** and **12g** are shown as thick residues in color-faded magenta and ***DB18*** is highlighted in green ball-and-stick models. Color coding: oxygen-red, nitrogen-blue, carbon-grey in case of protein. Background covers the rest of the protein chain in ribbon model and all the images were prepared using Maestro 13.1. Backbone, side-chain hydrogen bonds are shown as yellow-dotted lines, π-π stackings are given in light blue-dotted lines whereas, charged interactions are provided in pink-dotted lines. To focus the interactions of active site residues with ligands 6.0, Å surface area of protein has been shown in the figures.

Because molecular docking suggested that the *para*-methyl of the first series is solvent-exposed (Figure 2), we tested if a polar substituent could be more favorable, giving rise to the second series. Surprisingly, the *para*-hydroxy was not so good, as shown in Table 2. However, increasing the distance between the polar head and the phenyl permitted to recover part of the affinity, thanks to a strong hydrogen bond with D250 and/or Y249. Even more surprisingly, a carboxylic acid proved to be the best substituent on this position. Docking of **12g** suggested that this extreme modification of the physico-chemical nature of the inhibitor forced a rotation of 90° along the phenol (Figure 3a). In this conformation, the nitrophenyl forms a strong π-stacking with F172, and the carboxylic acid forms a salt bridge with K290, in addition to a hydrogen bond with the backbone of A171. This strong link between the bottom of the active site and its lid formed by the β-sheet domain G154-N195/Q226-E242 further stabilized the active site and explains why this compound is the best in this second series. The overlap between **12g** and ***DB18*** in CLK1 is presented in Figure 3b. **12g** is also very potent on other CLKs, and still relatively selective towards CLK3 and DYRK1A. The docking of **12g** in Dyrk1A is represented in Figure 3c and its overlap with ***DB18*** in Figure 3d.

**Figure 4:**
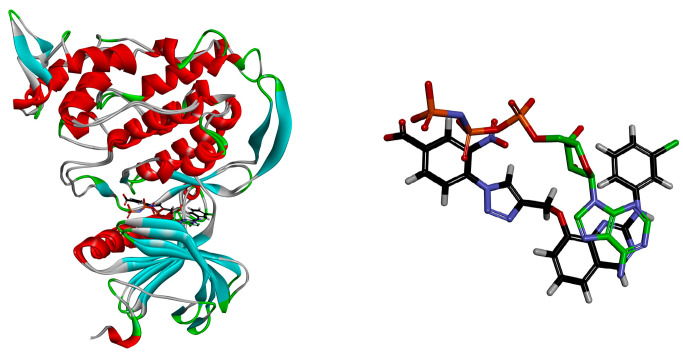
Superimposition of DYRK1A cocrystallized with ANP (carbons atoms in green on the right) and CLK1 withdocked **12g** (black carbon atoms on the right).

The very high affinity of the carboxylic acid derivative was a surprise. We were first astonished that the active site could tolerate an electronegative substituent in this region, because it is surrounded by many acidic amino-acids: E169, E206, D288, E292 and D325. Of course, several basic residues are also present, but they are few (K191, K194 and K290), and can only partially equilibrate the charges. The resulting electrostatic clash explains probably why docking experiments suggested a reorientation of the benzoic acid part of the inhibitor, as compared with ***DB18***. We checked this aspect through molecular dynamic simulation, by replacing the *para*-methyl of ***DB18*** by a carboxylic acid, in the exact binding mode of ***DB18***. After 300 ns, we observed an abrupt shift of the inhibitor position. It seems that the electronegative environment generated by acidic residues, particularly D250 and E292, forced an expulsion of the benzoic acid via a rotation of the phenol dihedral (Figure S2). We realized thereafter that this acidic region of the active site is precisely the domain encompassing the phosphate groups of the natural substrate ATP of kinases as evidenced by several crystal structures (For example, Figure 4 shows a {DYRK1A:ATP} complex (PDB:7A4O_B)} [21]. Since ATP is often bound to Mg^2+^ (see for example PDB:4IIR for a {kinase:ANP:Mg^2+^} complex [22], it is probable that CLKs also bind Mg^2+^ in this position, even if the cation has not been seen in X-ray structures.

The tolerance of CLKs’ active site towards very diverse substituents of the phenyl encourage us to test another extreme, with a basic group. To this end, we synthetized the third series by replacing the nitrophenyl of the first series by a cyclohexylamine. The resulting compounds still showed some activity (Table 3), even if it was weaker than the other series, and they are still weaker on CLK3 and DYRK1A than other kinases. Interestingly, the affinity is higher for DYRK1A than the first series. Since the DYRK1A active site is more polar than that of other kinases [14], it is not surprising that a polar inhibitor is better on this enzyme. Docking of **16b** suggested that its binding mode is closer to ***DB18***’s than **12g**’s (Figure S10). The triazole forms a stronger π-stacking with F172, however, and the primary amine forms a salt bridge with D250. Because of this interaction, the inhibitor curls itself up, allowing the *meta*’-chloro substituent of the phenyl to be more buried in the active site than for ***DB18***. This explains why it has a figure of the same order on CLK1 as the mono chloro **16a**.

To further evaluate our CLK inhibitors, it will be necessary to use in vivo models, most likely in mouse. So, we also tested the thirdseries on mouse CLK1 orthologue (*Mm*CLK1) and the results are reported in Table 5.

The results showed that the inhibitors are also active, or very active, on this species, but that their IC_50_’s showed variable differences with the human protein. In the first series, the best inhibitor is 10 times more potent on human than mouse CLK1. In the second and third series, they are in the same range but, interestingly, **12g** is the best inhibitor for both species in these new series of analogues. 

**Figure 5:**
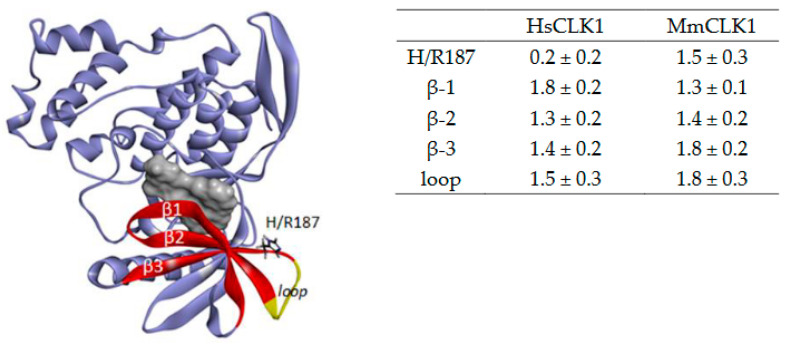
RMSD calculation (in Å) of H/R187 environment during 1 µs molecular dynamics of Apo human and mouse CLK1. On the top, cartoon representation of CLK1 shows the active site (grey surface) and analyzed secondary structures.

It was surprising to see such differences between human and mouse IC_50_’s in the first series, since the two orthologues share a high-sequence homology (90% sequence identity). In the active site region, the only difference is a H187R mutation. These residues are not in direct contact with inhibitors, and they even face the opposite direction, outside the active site. Therefore, we hypothesized that this sequence variation could have an indirect effect on the ligand affinity. To investigate this hypothesis, we ran molecular dynamics experiments for both human and mouse CLK1. A deep analysis of the Apo conformation of the enzymes showed that the 187 position is more flexible in mouse than in human enzyme, in agreement with the chemical nature of corresponding amino-acids. Consequently, the secondary structures around H/R187 are globally more flexible in the mouse orthologue (Figure 5). 

This flexibility drives a slight instability of the R186-V193 β-strand of the lid domain (β-3), which propagates to the whole active site and limits the affinity of the inhibitors. On the contrary, movements of the loop and the attached β-strand seems to stabilize the upper R160-A171 β-strand in *Mm*CLK1 (β-1), which covers the active site. Taken together, these differential movements simultaneously favoured the induced-fit mechanism in *Hs*CLK1 vs. *Mm*CLK1, while perturbing inhibitor/protein contacts in *Mm*CLK1. Interestingly, we already stressed the importance of this domain movement for explaining CLK3 and DYRK1A selectivity of ***DB18*** [14].

To summarize our SAR/molecular modelling analyses, several conclusions can be drawn. First, the *meta* substituent of the aniline part of the inhibitors is crucial for its activity, because of three features: (i) an increase in interactions with the protein, (ii) an intramolecular interaction that favours the active conformation of ligands, and (iii) an increased structural stabilization of the active site. Second, the triazole-bearing group (phenyl of cyclohexyl) is very versatile, since the protein tolerate here both hydrophobic (toluene), and hydrophilic (benzyl alcool) portions, and even ionic groups of opposite charge (benzoic acid, cyclohexylamine). Third, the intrinsic flexibility of CLKs’ active site has a pronounced effect on inhibitors selectivity, as well as orthologue affinity (human/mouse), and appeared as more important than simple amino acid conservation/mutation.

### 2.4. Cytotoxicity Studies

To complete our study, we performed a cytotoxicity screening of the 15 molecules, investigating 7 representative cancer cell lines. The compound’s effect on cell viability was evaluated on Huh-7 (hepatocellular carcinoma), CaCo_2_, HCT-116 (colorectal adenocarcinoma), MCF7, MDA-MB231, MDA-MB468 (breast carcinoma), PC3 (prostate carcinoma) and NCI-H727 (lung carcinoid). Human skin fibroblasts were used as reference for non-cancerous cells. Roscovitine, Doxorubicin and Taxol were used as positive controls. The cells were treated for 48 h with 25 µM compounds and fixed in cooled 90% ethanol/5% acetic acid for 20 min. The nuclei were stained with Hoechst 33342 and counted using HCS technology. Compounds that induced more than 30% cell viability decrease when compared to DMSO control treatment (set at 100%), for at least one cell line, were retained for the determination of the IC_50_ over a range of 6 concentrations. The results are presented in Table S2.

In the first series, the molecule **7a** showed a good cytotoxicity against most of the cancer cell lines and was non-toxic towards fibroblasts. On the contrary, the dichloro molecule **7b** exhibited no cytotoxicity, except a very low one with PC3 cells. It was also non-toxic to fibroblasts and therefore its profile was very close to our lead molecule ***DB18***.

In the second series, the results were also very structure-dependent: **12d** showed that it was highly cytotoxic for HuH7 cells and not for the others. Further, it had a moderate toxicity for fibroblasts. The two next molecules **12c** and **12e** demonstrated potent cytotoxicity for all cancer cells and a moderate toxicity towards fibroblasts. Finally, the last two compounds **12f** and **12g** were non-active against all cancer cells and also towards fibroblasts. Thus, their profile was very similar to our previous lead molecule ***DB18***.

For the third series, the results were sensitive to the structures. All *N*-Boc protected compounds were non-toxic to fibroblasts. On the other hand, **18a**, and to a lesser extent **15a**, demonstrated significant cytotoxicities against a good number of the selected cancer cell lines, and **15b** and **18a** had cytotoxicities more focused on a few (MDA-MB-231 or HuH7). On the contrary, the derivatives with primary amines (**16** and **19**) showed potent cytotoxicities against the complete series of cancer cells. However, they demonstrated also moderate to high toxicities against the fibroblasts.

### 2.5. Study of **DB18** Activity/Selectivity against a Large Panel of 468 Kinases

**Figure 6:**
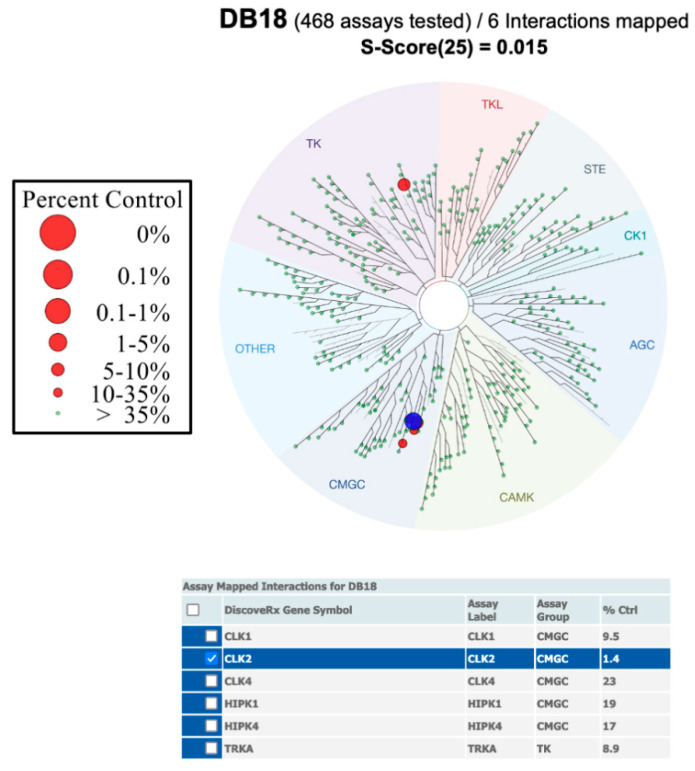
***DB18*** is a highly selective inhibitor of CLKs.

Since ***DB18*** proved to have the best compromise between a high activity on CLKs and a very high selectivity vs. DYRK1A, it was selected for assay against a large panel of 468 kinases (KINOMEscan^SM^ Assay) at 1 µM. Very interestingly, the compound was found to be highly selective for CLKs and affected to a lower extent HIPK1, 4 and TRKA. As an example, only 1.4% of CLK2 activity remained after incubation with ***DB18*** (the full list of results is reported in Table S5). The results are shown on a TREEspot™ Kinase dendrogram (DiscoverX) as an illustrative representation of the human kinome phylogenetic tree (Figure 6). The codes reported on this figure indicate the subclasses of protein kinases: CMGC for CDKs, MAP kinases, GSK and CDK-like kinases; AGC for protein kinase A, G, and C families (PKA, PKC, PKG); CAMK for Ca^2+^/calmodulin-dependent protein kinases; CK1, cell kinases 1 (originally known as Casein Kinase 1); STE, STE kinases (homologs of yeast STErile kinases); TKL, tyrosine kinases-like; TK, tyrosine kinases. Each kinase tested in the assay panel was marked with a green dot. The hit kinases reported were marked with a red circle, except CLK2 which was marked with a blue circle. 0% represents the higher affinity, whereas small green dots indicate that at 1 µM, ***DB18*** cannot inhibit significantly the kinase (as over 25% of the tested kinase are still on the affinity matrix after competition with the tested compound). S-Score (25) = (number of non-mutant kinases with %Ctrl < 25)/(number of non-mutant kinases tested = 403).

## 3. Experimental

### 3.1. Chemical Syntheses

General information:

For this, see corresponding data in ref [14]. All triazole compounds were purified by flash column chromatography on silica gel using 20% ethyl acetate in hexane, unless otherwise noted. For all final compounds (**7**, **12**, **16** and **19**) a purity >95% was established by LC-MS.

Preparation of compounds **3**, **4**, **7a**, **7b**, **13**:

Compounds **3**, **4**, **5**, **6**, **7a**, **7b**, **13** were prepared as already described for ***DB18*** [14].

*N*-Phenyl-8-((1-(4-methyl-2-nitrophenyl)-1*H*-1,2,3-triazol-4-yl)methoxy)quinazolin-2-amine (**7a**) (58%):

^1^H NMR (300 MHz, DMSO-d_6_, *δ* ppm): 9.88 (br s, 1H, NH), 9.29 (s, 1H), 8.80 (s, 1H), 8.09 (br dd, *J* = 1.1, 0.5 Hz, 1H), 8.06–7.99 (m, 2H), 7.79 (ddd with a strong roof effect, *J* = 8.2, 1.7, 0.5 Hz, 1H), 7.75 (br d with a strong roof effect, *J* = 8.1 Hz, 1H), 7.58–7.49 (m, 2H), 7.33 (t, *J* = 7.9 Hz, 1H), 7.26–7.17 (m, 2H), 6.88 (ddt, *J* = 7.6, 7.0, 1.0 Hz, 1H), 5.48 (s, 2H), 2.53 (s, 3H); ^13^C NMR (100 MHz, DMSO-d_6_, *δ* ppm): 161.8 (CH), 156.2 (C), 151.3 (C), 143.8 (C), 143.6 (C), 142.9 (C), 142.0 (C), 140.5 (C), 134.6 (CH), 128.3 (2CH), 127.2 (CH), 126.6 (C), 125.8 (CH), 125.5 (CH), 123.2 (CH), 121.2 (CH), 121.0 (C), 120.1 (CH), 118.5 (2CH), 115.8 (CH), 62.1 (CH_2_), 20.3 (CH_3_); HRMS (CH_3_OH/CH_2_Cl_2_ = 9:1) *m/z* calcd for C_24_H_20_N_7_O_3_ [M + H]^+^: 454.16221, found: 454.1626, calcd for C_24_H_19_N_7_NaO_3_ [M+Na]^+^: 476.14416, found: 476.1445, calcd for C_24_H_19_N_7_KO_3_ [M+K]^+^: 492.11810, found: 492.1182.

*N*-(3,5-dichlorophenyl)-8-((1-(4-methyl-2-nitrophenyl)-1*H*-1,2,3-triazol-4-yl)methoxy)quinazolin-2-amine (**7b**) (54%):

^1^H NMR (400 MHz, DMSO-d_6_) δ ppm: 10.34 (s, 1H), 9.38 (s, 1H), 8.86 (s, 1H), 8.17 (d, *J* = 1.6 Hz, 2H), 8.07 (s, 1H), 7.80–7.78 (m, 1H), 7.73 (d, *J* = 8.0 Hz, 1H), 7.61–7.58 (m, 2H), 7.42 (t, *J* = 8.0 Hz, 1H), 7.02 (t, *J* = 2.0 Hz, 1H), 5.45 (s, 2H), 2.08 (s, 3H); Mass (*m/z*): 522.1 (M + H).

Ethyl 4-amino-3-nitrobenzoate (**9**):

In a 100 mL round-bottomed flask, ethyl 4-acetamido-3-nitrobenzoate (**8**) (1.04 g, 4 mmol), acetic acid (2 mL, 34.9 mmol, 8.72 equiv), 1,3-propanediol (6 mL), hydrazine monohydrate (1.785 g, 35 mmol, 8.75 equiv) and an olive-shaped magnetic stirring bar were successively introduced. This reaction flask was flushed under nitrogen, stoppered tightly and steeped in a preheated oil bath at 90 °C. It was left for 15 h at 90 °C under smooth stirring (160 rpm). After cooling down to rt, water (30 mL) was added and the resulting mixture was stirred at 200 rpm. It was filtered using a Büchner and the yellow precipitated solid was washed several times with water. It was transferred into a bigger flask and dried under vacuum with a pump. It afforded ethyl 4-amino-3-nitrobenzoate (**9**) (718 mg) whose NMR showed that it was pure. Rinsing by hot methanol the Büchner and the filter paper afforded additional **9** (60 mg) so the overall yield of **9** was 91%; ^1^H NMR (300 MHz, acetone-d_6_, *δ* ppm): 8.71 (d, *J* = 2.0 Hz, 1H), 7.94 (dd, *J* = 8.9, 2.0 Hz, 1H), 7.7–7.3 (envelope which topped at 7.52 ppm, 2H, NH_2_), 7.14 (d, *J* = 8.9 Hz, 1H), 4.33 (q, *J* = 7.1 Hz, 2H), 1.36 (t, *J* = 7.1 Hz, 3H); ^13^C NMR (100 MHz, acetone-d_6_, *δ* ppm): 165.4 (C), 149.6 (C), 136.0 (CH), 129.0 (CH), 120.0 (CH), 118.9 (C), 61.4 (CH_2_), 14.6 (CH_3_).

4-Amino-3-nitrobenzyl alcohol (**10e**):

In a 250 mL round-bottomed flask, lithium chloride (initially 1.07 g, 0.813 g after drying, 19 mmol) was dried by flaming under vacuum. After cooling down rt, ethyl 4-amino-3-nitrobenzoate (201.9 mg, 0.96 mmol), 95% ethanol (14.4 mL), methanol (1.44 mL) and an olive-shaped magnetic stirring bar were successively introduced. This mixture was stirred at rt until LiCl was dissolved and sodium borohydride (735 mg, 19 mmol) was added. A red color appeared. The reaction flask was flushed under nitrogen, tightly stoppered and stirred at rt for 40 min. At this time, thickening was observed and LiBH(OMe)_3_ was formedin situas the reducing species. An excess of pressure (due to hydrogen formation) was evacuated by brief opening before tight stoppering again. This resulting mixture was stirred at 45 °C for 12 h upon which it changed from red to orange. TLC showed a complete reaction with a more polar spot of the expected alcohol (R*_f_* = 0.22 with petroleum ether/acetone 70:30 vs. 0.48 for the starting ethyl ester). Ethyl acetate (60 mL) and water (15 mL) were added and stirring at 500 pm was continued for 20–30 min. The remaining mineral mass was washed more than 10 times with stirring at rt with ethyl acetate. Ethyl acetate was then removed in vacuo followed by ethanol and water under high vacuum. The remaining residue and intermediate impure fractions were subjected to 4 successive chromatographies on silica gel (8–9 g) columns with gradient elution with petroleum ether/acetone 85:15 to 75:25 (deposit on silica gel was made with toluene + ethanol). It afforded 4-amino-3-nitrobenzyl alcohol (**10e**) as a red crystallized solid (57.0 mg, 35%); ^1^H NMR (300 MHz, acetone-d_6_, *δ* ppm): 8.02 (ddd after improving the resolution with Traficante, *J* = 2.0, 1.2, 0.8 Hz, 1H), 7.41 (dd, *J* = 8.6, 2.0 Hz (ddt after improving the resolution with Traficante, *J* = 8.6, 2.0, 0.5 Hz), 1H), 7.04 (dd after improving the resolution with Traficante, *J* = 8.6, 0.4 Hz, 1H), 7.3–6.8 (envelope which topped at 6.94 ppm, 2H, NH_2_), 4.54 (d, *J* = 4.6 Hz, 2H), 4.33–4.15 (m which topped at 4.24 ppm, 1H, OH); ^13^C NMR (75 MHz, acetone-d_6_, *δ* ppm): 146.0 (C), 135.7 (CH), 132.0 (broad and small, C), 131.4 (C), 124.0 (CH), 120.0 (CH), 63.6 (CH_2_).

4-Azido-3-nitrophenol (**11c**):

37% Aqueous hydrochloric acid (3.2 equiv) was added to a stirred solution of 4-amino-3-nitrophenol (157.6 mg, 1 mmol) in water (2.2 mL). After cooling by an ice bath, an aqueous solution of sodium nitrite (91.7 mg, 1.31 mmol, 1.31 equiv) in water (0.41 mL) was added dropwise. Transfer of NaNO_2_ was completed by rinsing with water (0.12 mL). After 1 h upon which the bath temperature had risen from 0 to 2 °C, an aqueous solution of sodium azide (104.7 mg, 1.6 mmol, 1.6 equiv) in water (0.53 mL) was added dropwise under stirring. Transfer of NaN_3_ was completed by rinsing with water (0.15 mL). An abundant precipitation of yellow crystals immediately occurred. After 45 min further stirring at 0 °C, the ice bath was removed and the reaction mixture was left aside to stir at rt overnight. The aspect of the reaction mixture had not at all changed so the reaction of N_3_^−^ was probably very fast. Extraction with ethyl acetate readily dissolved the precipitate and the organic extract was washed with water until neutral (3 times). Combined aqueous layers were reextracted with ethyl acetate and this second organic extract was washed with water until neutral (2 times). Combined organic extracts were dried (Na_2_SO_4_), concentrated and put under vacuum to afford a brown solid. TLC spot of the azide **11c** (0.42 with hexane/acetone 7:3) was yellow at the start and became brownish after less than one hour contact with air. Chromatography of the brown crude product on silica gel (2.0 g) with elution with hexane/acetone 90:10 and then 85:15 afforded **11c** as a yellow crystallized solid (174.8 mg, 97%); ^1^H NMR (300 MHz, acetone-d_6_, *δ* ppm): 12-8 (envelope which topped at 9.56 ppm, 1H, OH), 7.39 (d, *J* = 8.7 Hz, 1H), 7.39 (d, *J* = 2.9 Hz, 1H), 7.24 (dd, *J* = 8.9, 2.8 Hz, 1H); ^13^C NMR (100 MHz, acetone-d_6_, *δ* ppm): 155.8 (C), 142.7 (broad and small, C), 126.0 (C), 123.6 (CH), 122.4 (CH), 112.5 (CH).

1-Azido-4-bromo-2-nitrobenzene (**11d**):

To a stirred solution of 1-amino-4-bromo-2-nitrobenzene **(11d)** (500 mg, 2.30 mmol) in 6N HCl (10 mL) was added NaNO_2_ (317 mg, 4.60 mmol) in 2 mL water at 0 °C. Then stirred for 2 h at 0 °C, after then add NaN_3_ (299 mg, 4.60 mmol) lot wise at 0 °C, and stirred for overnight at rt. After completion of the reaction by TLC, the reaction mixture was poured into water and EtOAc. The phases were separated, and the organic layer was washed with water and concentrated to obtain the azide **11d** as a yellow solid. Compound directly used for the next step without purification; ^1^H NMR (400 MHz, CDCl_3_, *δ* ppm): 8.07 (d, *J* = 2.3 Hz, 1H), 7.73 (dd, *J* = 8.6, 2.3 Hz, 1H), 7.22 (d, *J* = 8.6 Hz, 1H); ^13^C NMR (100 MHz, CDCl_3_, *δ* ppm): 141.1 (small, C), 136.9 (CH), 134.0 (C), 128.9 (CH), 122.2 (CH), 117.1 (C).

4-Azido-3-nitrobenzyl alcohol (**11e**):

In a 25 mL round-bottomed flask, 4-amino-3-nitrobenzyl alcohol (19.7 mg, 0.117 mmol) was dissolved under stirring by adding water (1.25 mL), 37% aqueous hydrochloric acid (156 mg, 6 drops) and 95% ethanol (2.35 mL). The resulting orange solution was cooled at 0 °C. An aqueous solution of sodium nitrite (41.4 mg NaNO_2_, 0.594 mmol, 5.08 équiv + 462.4 mg water) was added dropwise under stirring. After 3 h further stirring at 0 °C, an aqueous solution of sodium azide (31.3 mg NaN_3_, 0.479 mmol, 4.1 équiv + 226.4 mg water) was added dropwise. Transfer of NaN_3_ was completed by rinsing with water (77.1 mg). Upon addition of NaN_3_, the color of the raction mixture changed from orange to yellow and a precipitation appeared. Stirring was then continued for 100 min at 0 °C and the resulting mixture was then left aside in a freezer overnight. After warming up to rt, TLC showed a less polar spot of azide (R*_f_* = 0.35 with petroleum ether/acetone 70:30) and two minor more polar spots, the most polar seemed to be that of starting amine. Water (about 5 mL) and sodium bicarbonate (90 mg) were added. The resulting mixture was extracted 4 times with ethyl acetate. After drying over sodium sulfate and concentration, the remaining orange crude wax (22.4 mg) was subjected to a chromatography on a column of silica gel (5 g) with gradient elution with petroleum ether/acetone 95:5 to 80:20 (deposit on silica gel was made with toluene + a small amount of ethanol with heating). It afforded 4-azido-3-nitrobenzyl alcohol (**11e**) as a cream yellow crystallized solid (10.7 mg, 47%); ^1^H NMR (300 MHz, CDCl_3_, *δ* ppm): 7.95 (dt after improving the resolution with Traficante, *J* = 2.0, 0.9 Hz, 1H), 7.62 (ddt, *J* = 8.3, 2.0, 0.7 Hz), 1H), 7.32 (d, *J* = 8.3 Hz, 1H), 4.76 (s, 2H), 2.2-1.9 (envelope which topped at 2.06 ppm, 2H, OH); ^13^C NMR (100 MHz, CDCl_3_, *δ* ppm): 140.8 (broad and small, C), 138.5 (C), 133.8 (C), 132.1 (CH), 124.1 (CH), 120.9 (CH), 63.4 (CH_2_).

Methyl 4-azido-3-nitrobenzoate (**11f**):

To a stirred solution of methyl 4-amino-3-nitrobenzoate (**10f**) (500 mg, 2.55 mmol) in 6N HCl (10 mL) was added NaNO_2_ (352 mg, 5.1 mmol) in 2 mL water at 0 °C. Then stirred at 0 °C for 2 h, after then added NaN_3_ (331 mg, 5.1 mmol) lot wise at 0 °C, stirred for overnight at rt. After completion of the reaction by TLC, the reaction mixture was poured into water and EtOAc. The phases were separated, and the organic layer was washed with water and concentrated to obtain the azide **11f** as a yellow solid. This compound was directly used for the next step without purification.

*N*-(3-Chlorophenyl)-8-((1-(4-hydroxy-2-nitrophenyl)-1*H*-1,2,3-triazol-4-yl)methoxy)quinazolin-2-amine (**12c**):

In a 10 mL round-bottomed flask containing 4-azido-3-nitrophenol (**11c**) (22.8 mg, 0.1266 mmol) was added alkyne **13** (37.7 mg, 0.1217 mmol), copper(II) sulfate pentahydrate (2.8 mg, 0.011 mmol, 0.09 equiv), sodium ascorbate (6.7 mg, 0.0335 mmol, 0.275 equiv), urea (2.3 mg, 0.0375 mmol, 0.31 equiv), water (89.0 mg, 3 drops), DMSO (149.0 mg, 8 drops), THF (0.655 mL) and a small olive-shaped magnetic stirring bar. That gave a mixture THF/DMSO/H_2_O about 15:3:2. The reaction flask was flushed under nitrogen, tightly stoppered and left under moderate stirring at rt (22–23 °C) for 5.5 h. In a separating funnel, water (0.42 mL), brine (saturated aq. NaCl: 1.57 mL) and 28–30% aqueous ammonium hydroxide (3 drops) were introduced to which was added the reaction mixture which was transferred by ethyl acetate and THF followed by diluted brine in order to transfer residual minerals which were remaining in the reaction flask. After partitioning, the aqueous phase was reextracted with ethyl acetate plus THF. Combined organic extracts were washed once with brine, dried (Na_2_SO_4_), concentrated and the remaining residue was put under vacuum. Chromatography on silica gel (2.1 g) column, which was loaded with dichloromethane (deposit of the crude product on the silica gel by dissolving in THF plus a little bit of methanol with smooth heating) eluting with 2% MeOH-chloroform, afforded triazole **12c** as a light orange powder (65.2 mg, quant., R*_f_* = 0.09 with 2% MeOH-chloroform). An analytically pure sample was obtained by crystallization in acetone/petroleum ether about 1:1 with final cooling in a freezer at about −35 °C to afford a yellow powder; ^1^H NMR (300 MHz, DMSO-d_6_, *δ* ppm): 10.13 (br s, 1H, NH), 9.34 (s, 1H), 8.72 (s, 1H), 8.42 (t, *J* = 2.0 Hz, 1H), 7.80 (ddd, *J* = 8.3, 2.1, 0.9 Hz, 1H), 7.63 (d, *J* = 8.7 Hz, 1H), 7.57 (d, *J* = 7.9 Hz, 2H), 7.54 (d, *J* = 2.7 Hz, 1H), 7.38 (dd, *J* = 8.2, 7.5 Hz, 1H), 7.30 (dd, *J* = 8.7, 2.7 Hz, 1H), 7.24 (t, *J* = 8.1 Hz, 1H), 6.92 (ddd, *J* = 7.9, 2.1, 0.9 Hz, 1H), 5.46 (s, 2H); ^13^C NMR (100 MHz, DMSO-d_6_, *δ* ppm): 162.0 (CH), 159.2 (C), 155.9 (C), 151.5 (C), 144.9 (C), 143.1 (C), 142.5 (C), 142.1 (C), 133.0 (C), 129.8 (CH), 128.9 (CH), 126.0 (CH), 123.8 (CH), 121.2 (C), 120.6 (CH), 120.5 (C), 120.5 (CH), 119.9 (CH), 117.5 (CH), 116.8 (CH), 115.4 (CH), 111.6 (CH), 62.0 (CH_2_); HRMS (CH_3_OH/CH_2_Cl_2_ = 9:1) *m/z* calcd for C_23_H_16_^35^ClN_7_NaO_4_ [M + Na]^+^: 512.08445, found: 512.0848, calcd for C_23_H_16_^35^ClN_5_NaO_4_[M-N_2_ + Na]^+^: 484.07830, found: 484.0782, C_23_H_16_^35^ClN_7_KO_4_ [M + K]^+^: 528.05839, found: 528.0579.

*N*-(3-Chlorophenyl)-8-((1-(4-bromo-2-nitrophenyl)-1*H*-1,2,3-triazol-4-yl)methoxy)quinazolin-2-amine (**12d**):

To a stirred solution of alkyne **13** (100 mg, 0.32 mmol) in t-BuOH: water (1:1) (2 mL) was added azide **18d** (157 mg, 0.64 mmol), sodium ascorbate (128 mg, 0.64 mmol), CuSO_4_.5H_2_O (161 mg, 0.64 mmol) in 4 mL water at RT. Then stirred for overnight at rt. After completion of the reaction by TLC, the reaction mixture was poured into water and EtOAc. The phases were separated, and the organic layer was washed with brine solution, dried over sodium sulphate and concentrated. Crude was purified by column chromatography (60–120 silica gel, plane ethyl acetate) to afford triazole **19d** (100 mg, 56%) as a brown solid (LC-MS purity: 92.8%); ^1^H NMR (300 MHz, DMSO-d_6_, *δ* ppm): 10.13 (br s, 1H, NH), 9.34 (s, 1H), 8.89 (s, 1H), 8.52 (d, *J* = 2.1 Hz, 1H), 8.43 (t, *J* = 2.0 Hz, 1H), 8.22 (dd, *J* = 8.5, 2.2 Hz, 1H), 7.85 (d, *J* = 8.5 Hz, 1H), 7.78 (ddd, *J* = 8.3, 2.1, 0.9 Hz, 1H), 7.60-7.53 (m, 2H), 7.38 (t, *J* = 7.9 Hz, 1H), 7.23 (t, *J* = 8.1 Hz, 1H), 6.92 (ddd, *J* = 7.9, 2.1, 0.9 Hz, 1H), 5.48 (s, 2H); ^13^C NMR (100 MHz, DMSO-d_6_, *δ* ppm): 162.0 (CH), 155.9 (C), 151.5 (C), 144.2 (C), 143.6 (C), 142.4 (C), 142.1 (C), 137.0 (CH), 132.9 (C), 129.8 (CH), 128.6 (CH), 128.2 (CH), 128.0 (C), 125.7 (CH), 123.7 (CH), 123.0 (C), 121.2 (C), 120.6 (CH), 120.0 (CH), 117.5 (CH), 116.7 (CH), 115.4 (CH), 61.9 (CH_2_); LC-MS: 554.1 [M + 2H] ^+^; HRMS (CH_3_OH/CH_2_Cl_2_ = 9:1) *m/z* calcd for C_23_H_15_^79^Br^35^ClN_7_NaO_3_ [M + Na]^+^: 574.00005, found: 574.0005, calcd for C_23_H_15_^79^Br^35^ClN_7_KO_3_ [M + K]^+^: 589.97398, found: 589.9740, calcd for C_23_H_16_^79^Br^35^ClN_7_O_3_ [M + H]^+^: 552.01810, found: 552.0176.

4-(4-(((2-((3-Chlorophenyl)amino)quinazolin-8-yl)oxy)methyl)-1*H*-1,2,3-triazol-1-yl)-3-nitrobenzyl alcohol (**12e**):

In a 25 mL round-bottomed flask containing 4-azido-3-nitrobenzyl alcohol (**11e**) (10.7 mg, 0,055 mmol) was added alkyne **5** (17.1 mg, 0.055 mmol), copper(II) sulfate pentahydrate (2.1 mg, 0.00824 mmol, 0.15 equiv), sodium ascorbate (5.2 mg, 0.026 mmol, 0.47 equiv), urea (1.8 mg, 0.0294 mmol, 0.53 equiv), water (53.1 mg, 2 drops), DMSO (105.0 mg, 6 drops), THF (0.553 mL) and a small olive-shaped magnetic stirring bar. The reaction flask was flushed under nitrogen, tightly stoppered and left under moderate stirring at rt for 4.5 h. TLC showed a complete reaction with the expected triazole as a very polar compound (R*_f_* ~ 0.04 with petroleum ether/acetone 70:30 vs. 0.45 for the starting alkyne and 0.35 for the starting azide. In a separating funnel, water (1 mL) and 25% aqueous ammonium hydroxide (4 drops) were introduced to which was added the reaction mixture which was transferred by ethyl acetate (8.3 mL overall). A fine brick red precipitate appeared (presumably Cu_2_O). After partitioning, the aqueous phase was reextracted with ethyl acetate (3–4 mL). Combined yellow organic extracts were dried (Na_2_SO_4_), concentrated and the brown oily residue was put under vacuum. Chromatography on silica gel (6.6 g) eluting with ethyl acetate afforded triazole **12e** (R*_f_* = 0.45 with ethyl acetate) initially as a yellow oil which entirely crystallized in about 15 min at 50 °C to yield a yellow solid (22.4 mg, 81%).

^1^H NMR (300 MHz, DMSO-d_6_, *δ* ppm): 10.13 (br s, 1H, NH), 9.34 (s, 1H), 8.86 (s, 1H), 8.44 (t, *J* = 2.0 Hz, 1H), 8.15 (dt, *J* = 1.7, 0.9 Hz, 1H), 7.90 (ddt with a strong roof effect, *J* = 8.2, 1.8, 0.7 Hz, 1H), 7.82 (d with a strong roof effect, *J* = 8.2 Hz, 1H), 7.78 (ddd, *J* = 8.3, 2.1, 0.9 Hz, 1H), 7.57 (pseudo d, *J* = 8.0 Hz, 2H), 7.38 (dd, *J* = 8.4, 7.4 Hz, 1H), 7.23 (t, *J* = 8.1 Hz, 1H), 6.91 (ddd, *J* = 7.9, 2.1, 0.9 Hz, 1H), 5.68 (t, *J* = 5.7 Hz, 1H), 5.48 (s, 2H), 4.72 (d, *J* = 5.7 Hz, 2H); ^13^C NMR (100 MHz, DMSO-d_6_, *δ* ppm): 162.0 (CH), 155.9 (C), 151.5 (C), 146.6 (C), 143.7 (C), 143.4 (C), 142.4 (C), 142.1 (C), 132.9 (C), 131.4 (CH), 129.8 (CH), 127.3 (C), 127.1 (CH), 125.8 (CH), 123.7 (CH), 122.6 (CH), 121.2 (C), 120.6 (CH), 119.9 (CH), 117.5 (CH), 116.7 (CH), 115.3 (CH), 61.9 (CH_2_), 61.3 (CH_2_)); HRMS (CH_3_OH/CH_2_Cl_2_ = 9:1) *m/z* calcd for C_24_H_18_^35^ClN_7_NaO_4_ [M + Na]^+^: 526.10010, found: 526.1008, C_24_H_18_^35^ClN_7_KO_4_ [M + K]^+^: 542.07404, found: 542.0743.

Methyl 4-(4-(((2-((3-chlorophenyl)amino)quinazolin-8-yl)oxy)methyl)-1*H*-1,2,3-triazol-1-yl)-3-nitrobenzoate (**12f**):

To a stirred solution of alkyne **13** (100 mg, 0.32 mmol) in t-BuOH: water (1:1) (2 mL) was added azide **11f** (134 mg, 0.64 mmol), sodium ascorbate (128 mg, 0.64 mmol), CuSO_4_.5H_2_O (161 mg, 0.64 mmol) in 4 mL water at rt. Then stirred at rt for overnight. After completion of the reaction by TLC, the reaction mixture was poured into water and EtOAc. The phases were separated, and the organic layer was washed with brine solution, dried over sodium sulphate and concentrated. Crude purified by column chromatography (60–120 silica gel, plane ethyl acetate) to afford triazole **12f** (40 mg, 23%) as a brown solid. ^1^H NMR (300 MHz, DMSO-d_6_, *δ* ppm): 10.13 (br s, 1H, NH), 9.34 (s, 1H), 8.98 (s, 1H), 8.64 (d, *J* = 1.8 Hz, 1H), 8.49–8.42 (m, 2H), 8.06 (d, *J* = 8.3 Hz, 1H), 7.77 (ddd, *J* = 8.3, 2.0, 0.8 Hz, 1H), 7.58 (pseudo dm, *J* = 7.8 Hz, 2H), 7.38 (t, *J* = 7.9 Hz, 1H), 7.23 (t, *J* = 8.1 Hz, 1H), 6.90 (ddd, *J* = 7.9, 2.1, 0.8 Hz, 1H), 5.50 (s, 2H), 3.97 (s, 3H); ^13^C NMR (100 MHz, DMSO-d_6_, *δ* ppm): 163.7 (C), 162.0 (CH), 155.9 (C), 151.5 (C), 143.8 (C), 143.6 (C), 142.5 (C), 142.0 (C), 134.3 (CH), 132.9 (C), 131.9 (C), 131.6 (C), 129.8 (CH), 127.5 (CH), 126.1 (CH), 125.7 (CH), 123.7 (CH), 121.2 (C), 120.6 (CH), 120.0 (CH), 117.6 (CH), 116.7 (CH), 115.4 (CH), 61.8 (CH_2_), 53.0 (CH_3_); HRMS (CH_3_OH/CH_2_Cl_2_=9:1) *m/z* calcd for C_25_H_18_^35^ClN_7_NaO_5_ [M + Na]^+^: 554.09501, found: 554.0953, calcd for C_25_H_18_^35^ClN_7_KO_5_ [M + K]^+^: 570.06895, found: 570.0689, calcd for C_25_H_19_^35^ClN_7_O_5_ [M + H]^+^: 532.11307, found: 532.1129.

4-(4-(((2-((3-Chlorophenyl)amino)quinazolin-8-yl)oxy)methyl)-1*H*-1,2,3-triazol-1-yl)-3-nitrobenzoic acid (**12g**):

To a stirred solution of methyl ester **12f** (30 mg, 0.0564 mol) in MeOH/THF/water (3:2:1) (3 mL) was added LiOH.H_2_O (7.0 mg, 0.169 mol) and stirring was continued at rt for 16 h. After completion of the reaction by TLC, the reaction mixture was concentrated, diluted with water and acidified with 2N aqueous HCl. Then the solid which was formed was filtered and dried to obtain carboxylic acid **12g** as a yellow solid (15 mg, 52%). ^1^H NMR (300 MHz, DMSO-d_6_, *δ* ppm): 10.13 (br s, 1H, NH), 9.34 (s, 1H), 8.96 (s, 1H), 8.61 (d, *J* = 1.8 Hz, 1H), 8.47–8.41 (m, 2H), 8.02 (d, *J* = 8.3 Hz, 1H), 7.78 (ddd, *J* = 8.3, 2.1, 0.9 Hz, 1H), 7.58 (pseudo dm, *J* = 7.8 Hz, 2H), 7.38 (t, *J* = 7.9 Hz, 1H), 7.23 (t, *J* = 8.1 Hz, 1H), 6.91 (ddd, *J* = 7.9, 2.1, 0.9 Hz, 1H), 5.50 (s, 2H) [Remark: H of CO_2_H was not detected]; ^13^C NMR (75 MHz, DMSO-d_6_, *δ* ppm): 164.6 (C), 162.0 (CH), 155.9 (C), 151.5 (C), 143.8 (C), 143.6 (C), 142.5 (C), 142.0 (C), 134.4 (CH), 133.0 (C), 132.9 (C), 131.6 (C), 129.8 (CH), 127.5 (CH), 126.1 (CH), 125.7 (CH), 123.7 (CH), 121.2 (C), 120.6 (CH), 120.0 (CH), 117.6 (CH), 116.8 (CH), 115.5 (CH), 61.9 (CH_2_); LC-MS purity: 95%, LC-MS: 518.1 [M + H]^+^; HRMS (CH_3_OH/CH_2_Cl_2_ = 9:1) *m/z* calcd for C_24_H_15_^35^ClN_7_O_5_ [M-H]^−^: 516.08287, found: 516.0831, calcd for C_24_H_16_^35^Cl_2_N_7_O_5_ [M + Cl]^−^: 552.05955, found: 552.0598, calcd for C_24_H_15_^35^Cl_2_N_7_NaO_5_ [M-H + Na + Cl]^−^: 574.04149, found: 574.0417.

*tert*-Butyl (*cis*-4-azidocyclohexyl)carbamate (**14**):

Step 1. Synthesis of *trans*-4-(*tert*-butoxycarbonylamino)cyclohexyl methanesulfonate:

To a stirred solution of *trans*-4-Boc-aminocyclohexanol (500 mg, 2.32 mmol) in DCM (5 mL) was added TEA (1 mL, 6.97 mmol) at rt and stirred for 15 min. Then added Ms-Cl (0.3 mL, 3.48 mmol) drop wisely, stirred at RT for 6 h. After completion of the reaction by TLC, the reaction mixture was poured into water (5 mL). The phases were separated, and the organic layer was washed with a sat aq. NaHCO_3_ solution (5 mL), dried over sodium sulfate and concentrated and dried to obtain the *trans*-4-(*tert*-butoxycarbonylamino)cyclohexyl methanesulfonate as a colorless liquid.

Step 2. Synthesis of *tert*-butyl (*cis*-4-azidocyclohexyl)carbamate (**14**):

To a stirred solution of *trans*-4-(*tert*-butoxycarbonylamino)cyclohexyl methanesulfonate (500 mg, 1.70 mmol) in DMF (3 mL) was added NaN_3_ (1.1 g, 17.06 mmol) at rt. Then heated to 80 °C for 24 h. After completion of the reaction by TLC, the reaction mixture was poured into ice water, solid was formed, filtered and dried to obtain the azide **14** as an off-white solid. This compound was directly used for the next step without purification.

*tert*-Butyl ((*cis*)-4-(4-(((2-((3-chlorophenyl)amino)quinazolin-8-yl)oxy)methyl)-1*H*-1,2,3-triazol-1-yl)cyclohexyl)carbamate (**15a**):

To a stirred solution of alkyne **13** (100 mg, 0.32 mmol) in t-BuOH: water (1:1) (2 mL) was added azide **14** (77.6 mg, 0.32 mmol), followed by sodium ascorbate (64 mg, 0.32 mmol) and CuSO_4_.5H_2_O (26 mg, 0.104 mmol) in 4 mL water at RT. Then stirred for overnight at RT. After completion of the reaction by TLC, the reaction mixture was poured into water and EtOAc. The phases were separated, and the organic layer was washed with brine solution, dried over sodium sulphate and concentrated. Crude was purified by column chromatography (60–120 silica gel, plane ethyl acetate) to afford triazole **15a** as an off-white solid (120 mg, 67%); ^1^H NMR (300 MHz, DMSO-d_6_, *δ* ppm): 10.10 (br s, 1H, NH), 9.32 (s, 1H), 8.40 (t, *J* = 2.0 Hz, 1H), 8.35 (s, 1H), 7.78 (ddd, *J* = 8.3, 2.1, 0.9 Hz, 1H), 7.57–7.49 (m, 2H: 1 dd with a roof effect at 7.55 ppm, *J* = 8.0, 1.2 Hz and 1 dd with a roof effect at 7.53 ppm, *J* = 7.8, 1.2 Hz), 7.36 (t, *J* = 7.9 Hz, 1H), 7.24 (t, *J* = 8.1 Hz, 1H), 6.96 (ddd, *J* = 7.9, 2.1, 0.9 Hz, 1H), 6.99–6.87 (broad envelope which topped at 6.92 ppm, 1H), 5.35 (s, 2H), 4.56 (tt, *J* = 9.9, 3.9 Hz, 1H), 3.72–3.59 (m which topped at 3.66 ppm, 1H), 2.26–2.08 (m, 2H), 1.94–1.81 (m, 2H), 1.77–1.59 (m, 4H), 1.39 (s, 9H); ^13^C NMR (75 MHz, DMSO-d_6_, *δ* ppm): 161.9 (CH), 155.8 (C), 155.0 (C), 151.7 (C), 142.5 (C), 142.2 (C), 142.1 (C), 133.0 (C), 129.8 (CH), 123.7 (CH), 122.8 (CH), 121.2 (C), 120.6 (CH), 119.7 (CH), 117.6 (CH), 116.7 (CH), 115.2 (CH), 77.6 (C), 62.2 (CH_2_), 57.6 (CH), 44.7 (broad and small, CH), 28.4 (2CH_2_), 28.2 (3CH_3_), 27.4 (2CH_2_); HRMS (CH_3_OH/CH_2_Cl_2_ = 9:1) *m/z* calcd for C_28_H_32_^35^ClN_7_NaO_3_ [M + Na]^+^: 572.21474, found: 572.2150, calcd for C_24_H_17_^35^Cl_3_N_6_KO [M + K]^+^: 588.18867, found: 588.1888, calcd for C_28_H_33_^35^ClN_7_O_3_ [M + H]^+^: 550.23279, found: 550.2330.

*tert*-Butyl ((*cis*)-4-(4-(((2-((3,5-dichlorophenyl)amino)quinazolin-8-yl)oxy)methyl)-1*H*-1,2,3-triazol-1-yl)cyclohexyl)carbamate (**15b**):

Reaction of alkyne **5b** (100 mg, 0.29 mmol) with azide **14** (70 mg, 0.29 mmol) gave triazole **15b** (100 mg, 59%); ^1^H NMR (300 MHz, DMSO-d_6_, *δ* ppm): 10.34 (br s, 1H, NH), 9.37 (s, 1H), 8.37 (s, 1H), 8.17 (d, *J* = 1.9 Hz, 2H), 7.57 (nearly d with a roof effect, *J* = 7.9 Hz, 2H), 7.41 (nearly dd with a roof effect (protons at 7.41 and 7.57 ppm are coupled with each other), *J* = 8.2, 7.6 Hz, 1H), 7.06 (t, *J* = 1.9 Hz, 1H), 6.98 (br d, *J* = 7.0 Hz, 1H, NH), 5.33 (s, 2H), 4.54 (tt, *J* = 10.1, 3.9 Hz, 1H), 3.72–3.59 (m which topped at 3.66 ppm, 1H), 2.25–2.08 (m, 2H), 1.93–1.79 (m, 2H), 1.77–1.59 (m, 4H), 1.39 (s, 9H); ^13^C NMR (100 MHz, DMSO-d_6_, *δ* ppm): 162.1 (CH), 155.5 (C), 155.0 (broad and small, C), 151.8 (C), 143.0 (C), 142.1 (C), 141.9 (C), 133.7 (2C), 124.2 (CH), 123.0 (CH), 121.3 (C), 119.8 (CH), 119.5 (CH), 116.1 (2CH), 114.8 (CH), 77.5 (C), 62.0 (CH_2_), 57.6 (CH), 44.6 (broad and small, CH), 28.4 (2CH_2_), 28.2 (3CH_3_), 27.4 (2CH_2_); HRMS (CH_3_OH/CH_2_Cl_2_ = 9:1) *m/z* calcd for C_28_H_31_^35^Cl_2_N_7_NaO_3_ [M + Na]^+^: 606.17576, found: 606.1761, calcd for C_28_H_31_^35^Cl_2_N_7_KO_3_ [M + K]^+^: 622.14970, found: 622.1490, calcd for C_28_H_30_^35^Cl_2_N_7_Na_2_O_3_ [M-H + 2Na]^+^: 628.15771, found: 628.1571.

8-((1-((*cis*)-4-Aminocyclohexyl)-1*H*-1,2,3-triazol-4-yl)methoxy)-*N*-(3-chlorophenyl)quinazolin-2-amine hydrochloride (**16a**):

To a stirred solution of carbamate **15a** (50 mg) in methanol (2.0 mL) was added 4 M HCl in dioxane (1 mL) dropwise and stirred at room temperature for 1 h, After completion of the reaction by TLC, the reaction mixture was concentrated to obtain the hydrochloride **16a** as an off white solid in 76% yield. ^1^H NMR (400 MHz, DMSO-d_6_) δ ppm: 10.28 (s, 1H), 9.36 (s, 1H), 8.58 (s, 1H), 8.39 (s, 1H), 8.31 (s, 3H), 7.75 (dd, *J* = 1.6, 8.4 Hz, 1H), 7.58–7.55 (m, 2H), 7.38 (t, *J* = 8.0 Hz, 1H), 7.26 (t, *J* = 8.0 Hz, 1H), 6.98 (dd, *J* = 1.6, 8.0 Hz, 1H), 5.36 (s, 2H), 4.69–4.66 (m, 1H), 3.34 (bs, 1H), 2.39–2.34 (m, 2H), 1.97–1.86 (m, 5H); ^13^C NMR (400 MHz, DMSO-d_6_): **δ** 163.0, 156.2, 151.8, 142.9, 142.4, 133.6, 130.4, 124.5, 123.9, 121.6, 121.4, 120.3, 118.4, 117.5, 115.9, 66.8, 62.7, 56.6, 29.5, 27.2, 26.5: Mass (*m/z*): 450.3 (M + H).

8-((1-((*cis*)-4-Aminocyclohexyl)-1*H*-1,2,3-triazol-4-yl)methoxy)-*N*-(3,5-dichlorophenyl)quinazolin-2-amine hydrochloride (**16b**):

Reaction of carbamate **15b** (50 mg, 0.085 mmol) with HCl in dioxane gave hydrochloride **16b** (31 mg, 74%). ^1^H NMR (400 MHz, DMSO-d_6_) δ ppm: 10.36 (s, 1H), 9.37 (s, 1H), 8.55 (s, 1H), 8.29 (s, 2H), 8.17 (d, *J* = 1.6 Hz, 2H), 7.59–7.56 (m, 2H), 7.40 (t, *J* = 7.6 Hz, 1H), 7.06 (t, *J* = 1.6Hz, 1H), 5.33 (s, 2H), 4.69–4.63 (m, 1H), 3.66 (bs, 1H), 2.38–2.33 (m, 2H), 1.96–1.85 (m, 6H); ^13^C NMR (400 MHz, DMSO-d_6_): δ 162.7, 156.1, 152.2, 143.5, 142.6, 134.3, 124.8, 124.1, 121.8, 120.4, 120.1, 116.8, 115.3, 62.4, 56.65, 46.6, 31.8, 27.2, 26.5: Mass (*m/z*): 484.0 (M + H).

*tert*-Butyl ((*trans*)-4-(4-(((2-((3-chlorophenyl)amino) quinazolin-8-yl) oxy) methyl)-1*H*-1,2,3-triazol-1-yl)cyclohexyl)carbamate (**18a**):

Reaction of alkyne **13** (100 mg, 0.32 mmol) with azide **17** (77.6 mg, 0.32 mmol) gave triazole **18a** (100 mg, 56%); ^1^H NMR (300 MHz, DMSO-d_6_, *δ* ppm): 10.11 (br s, 1H, NH), 9.32 (s, 1H), 8.44 (t, *J* = 2.0 Hz, 1H), 8.34 (s, 1H), 7.74 (ddd, *J* = 8.3, 2.1, 0.9 Hz, 1H), 7.57–7.50 (m, 2H), 7.36 (t, *J* = 7.9 Hz, 1H), 7.24 (t, *J* = 8.1 Hz, 1H), 6.97 (ddd, *J* = 7.9, 2.1, 0.9 Hz, 1H), 6.82 (br d, *J* = 7.3 Hz, 1H, exchanged by D_2_O), 5.32 (s, 2H), 4.47 (tt, *J* = 11.6, 3.8 Hz, 1H), 3.42–3.26 (m, 1H, superimposed to the signal of water which was disclosed by addition of D_2_O), 2.16–2.04 (m, 2H), 1.99–1.78 (m, 4H), 1.40 (s, 9H) and superimposed 2H m at 1.50–1.30 ppm; ^13^C NMR (100 MHz, DMSO-d_6_, *δ* ppm): 161.9 (CH), 155.8 (C), 154.8 (C), 151.7 (C), 142.5 (C), 142.1 (2C), 133.0 (C), 129.7 (CH), 123.7 (CH), 122.9 (CH), 121.1 (C), 120.5 (CH), 119.6 (CH), 117.6 (CH), 116.7 (CH), 115.0 (CH), 77.4 (C), 62.1 (CH_2_), 58.3 (CH), 48.0 (small, CH), 31.4 (2CH_2_), 30.9 (2CH_2_), 28.2 (3CH_3_); HRMS (CH_3_OH/CH_2_Cl_2_ = 9:1) *m/z* calcd for C_28_H_32_^35^ClN_7_NaO_3_ [M + Na]^+^: 572.21474, found: 572.2148, calcd for C_24_H_17_^35^Cl_3_N_6_KO [M + K]^+^: 588.18867, found: 588.1889.

*tert*-Butyl ((*trans*)-4-(4-(((2-((3,5-dichlorophenyl)amino)quinazolin-8-yl)oxy) methyl)-1*H*-1,2,3-triazol-1-yl)cyclohexyl)carbamate (**18b**):

Reaction of alkyne **5b** (100 mg, 0.29 mmol) with azide **17** (70 mg, 0.29 mmol) gave triazole **18b** (100 mg, 59%); ^1^H NMR (300 MHz, DMSO-d_6_, *δ* ppm): 10.30 (br s, 1H, NH), 9.36 (s, 1H), 8.35 (s, 1H), 8.16 (d, *J* = 1.9 Hz, 2H), 7.59–7.53 (m: approximately br d at 7.56 ppm with a roof effect, *J*_~_ 8 Hz, 2H), 7.40 (dd plus 2 small inner bands with a roof effect, *J* = 8.1, 7.7 Hz, 1H), 7.07 (t, *J* = 1.9 Hz, 1H), 6.82 (br d, *J* = 7.3 Hz, 1H, exchanged by D_2_O), 5.31 (s, 2H), 4.46 (tt, *J* = 11.7, 3.8 Hz, 1H), 3.42–3.22 (m, 1H, superimposed to the signal of water which was disclosed by addition of D_2_O), 2.16–2.04 (m, 2H), 1.99–1.78 (m, 4H), 1.40 (s, 9H) and superimposed 2H m at 1.45–1.31 ppm; ^13^C NMR (100 MHz, DMSO-d_6_, *δ* ppm): 162.1 (CH), 155.5 (C), 154.8 (C), 151.8 (C), 143.0 (C), 142.1 (C), 141.8 (C), 133.7 (2C), 124.2 (CH), 123.1 (CH), 121.3 (C), 119.8 (CH), 119.5 (CH), 116.1 (2CH), 114.6 (CH), 77.4 (C), 61.9 (CH_2_), 58.3 (CH), 48.0 (small, CH), 31.4 (2CH_2_), 30.9 (2CH_2_), 28.2 (3CH_3_); HRMS (CH_3_OH/CH_2_Cl_2_ = 9:1) *m/z* calcd for C_28_H_31_^35^Cl_2_N_7_NaO_3_ [M + Na]^+^: 606.17576, found: 606.1752, calcd for C_24_H_23_^35^Cl_2_N_7_NaO_3_ [M-C_4_H_8_ + Na]^+^: 550.11316, found: 550.1120.

8-((1-((*trans*)-4-Aminocyclohexyl)-1*H*-1,2,3-triazol-4-yl)methoxy)-*N*-(3-chlorophenyl)quinazolin-2-amine hydrochloride (**19a**):

Reaction of carbamate **18a** (50 mg, 0.091 mmol) with HCl in dioxane gave hydrochloride **19a** (30 mg, 72%). ^1^H NMR (400 MHz, DMSO-d_6_): δ 10.23 (s,1H), 9.34 (s, 1H), 8.43 (s, 1H), 8.37 (s, 1H), 8.28 (d, *J* = 4.0 Hz, 3H), 7.73 (dd, *J* = 1.2 Hz, 8.0 Hz, 1H), 7.56–7.53 (m, 2H), 7.37 (t, *J* = 8.0 Hz, 1H), 7.25 (t, *J* = 8.0 Hz, 1H), 6.98 (dd, *J* = 1.6 Hz, 8.0 Hz, 1H), 5.34 (s, 2H), 4.56–4.50 (m, 1H), 3.17–3.12 (m, 1H), 2.15 (t, *J* = 9.2 Hz, 4H), 1.98–1.88 (m, 2H), 1.67–1.60 (m, 2H). ^13^C NMR (400 MHz, DMSO-d_6_): δ 162.8, 156.26, 152.0, 142.6, 133.6, 130.3, 124.4, 123.6, 121.7, 121.3, 120.28, 118.3, 117.4, 115.7, 66.8, 62.7, 58.2, 48.5, 30.9, 29.2; Mass (*m/z*): 450.0 (M + H).

8-((1-((*trans*)-4-Aminocyclohexyl)-1*H*-1,2,3-triazol-4-yl)methoxy)-*N*-(3,5-dichlorophenyl)quinazolin-2-amine hydrochloride (**19b**):

Reaction of carbamate **18b** (50 mg, 0.085 mmol) with HCl in dioxane gave hydrochloride **19b** (31 mg, 74%). ^1^H NMR (400 MHz, DMSO-d_6_): δ 10.37 (s, 1H), 9.37 (s, 1H), 8.43 (s, 1H), 8.37 (s, 1H), 8.28 (d, *J* = 3.6 Hz, 2H), 8.17 (d, *J* = 1.6 Hz, 2H), 7.56 (d, *J* = 8.0 Hz, 2H), 7.40 (t, *J* = 8.0 Hz, 1H), 7.07 (t, *J* = 2.0 Hz, 1H), 5.31 (s, 2H), 4.55–4.50 (m, 1H), 3.17–3.12 (m, 1H), 2.51 (t, *J* = 1.6 Hz, 4H), 2.17–2.12 (m, 2H), 1.97–1.89 (m, 2H). ^13^C NMR (400 MHz, DMSO-d_6_): δ 162.7, 156.0, 152.3, 143.5, 142.4, 134.3, 124.8, 123.9, 121.8, 120.4, 120.0, 116.7, 115.2, 66.8, 62.4, 58.2, 48.5, 30.9, 29.2; Mass (*m/z*): 484.2 (M + H).

### 3.2. Kinase Inhibition Studies

ADP-Glo™ assay kit (Promega, Madison, WI, USA) was used to quantify the protein kinase enzymatic activities according to manufacturer’s recommendations [23]. Briefly, reactions were carried out in a final volume of 6 µL for 30 min at 30 °C in appropriate kinase buffer (10 mM MgCl_2_, 1 mM EGTA, 1 mM DTT, 25 mM Tris-HCl pH 7.5, 50 µg/mL heparin), with either protein or peptide as substrate in the presence of 10 µM ATP. All recombinant protein kinases used during this study were expressed by baculovirus in *Sf*9 insect cells except for *Hs*CDK5/p25, *Mm*CLK1, *Hs*Haspin, and *Rn*DYRK1A, that were expressed in bacteria. These targets were assayed with the following substrates: *Hs*CK1ε was assayed with 170 µM of the peptide RRKHAAIGSpAYSITA; *Hs*CDK5/p25 was assayed with 37.2 µM of Histone H1 as substrate; *Hs*CDK9/CyclinT was assayed with 83 µM of the peptide YSPTSPSYSPTSPSYSPTSPSKKKK as substrate; *Hs*GSK-3β was assayed with 20 µM of GS-1 peptide, a GSK-3-selective substrate (YRRAAVPPSPSLSRHSSPHQSpEDEEE); *Hs*PIM1 was assayed with 630 µM of PimTide (ARKRRRHPSGPPTA) as substrate; *Hs*HASPIN was assayed with 8 µM of Histone H3 (1-21) peptide (ARTKQTARKSTGGKAPRKQLA) as substrate; *Mm*CLK1 and *Hs*CLK1 were assayed with 57.3 µM of the peptide GRSRSRSRSRSR as substrate; *Rn*DYRK1A and *Hs*DYRK1A were assayed with 10.7 µM of the peptide KKISGRLSPIMTEQ as substrate. The control inhibitors used to validate each kinase assay were: Staurosporine from *Streptomyces sp*. (#S5921, purity 95%, Sigma-Aldrich, St. Louis, NO, USA) for *Hs*CK1ε; Indirubin-3′ -oxime (#I0404, Sigma-Aldrich, St. Louis, NO, USA) for *Hs*CDK5/p25, *Hs*CDK9/CyclinT, *Hs*GSK-3β, *Rn*DYRK1A, *Hs*DYRK1A, *Mm*CLK1 and *Hs*CLK1; CHR6494 (#SML0648, Sigma-Aldrich, St. Louis, NO, USA) for *Hs*HASPIN and SGI-1776 (#S2198, Selleckchem) for *Hs*PIM1. In order to determine the half maximal inhibitory concentration (IC_50_), the assays were performed in duplicate in the absence or presence of increasing doses of the tested compounds. Kinase activities were expressed in % of maximal activity, i.e., measured in the absence of inhibitor. The data were processed using GraphPad PRISM Software (GraphPad Software, San Diego, CA, USA) to fit a sigmoidal curve that allowed us to determine the IC_50_ values. We used R-squared as goodness-of-fit measure to characterize how the sigmoidal curve model fit our experimental data (R-squared > 85%).

Several inhibition studies for human kinases (*Hs*CLK2, *Hs*CLK3, *Hs*CLK4), as well as the KINOMEscan^SM^ Assaywere performed by the Eurofins company, following protocols reported on their website.

### 3.3. Cytotoxicity Studies

#### 3.3.1. Cell Culture 

HuH7, CaCo-2, MDA-MB-231, HCT116, PC3, MCF7 and NCI-H727 cancer cell lines were obtained from the ECACC collection (Porton Down, UK). Cells were grown at 37 °C, 5% CO_2_ in ECACC recommended media: DMEM for HuH7, MDA-MB-231 and fibroblast, EMEM for MCF7and CaCo-2, McCoy’s for HCT116 and RPMI for PC3 and NCI-H727. All culture media were supplemented with 10% of FBS, 1% of penicillin-streptomycin and 2 mM glutamine.

#### 3.3.2. Cytotoxic Assay

Chemicals were solubilized in DMSO at a concentration of 10 mM (stock solution) and diluted in culture medium to the desired final concentrations.

The cytotoxic assay of chemical was performed at 25 µM. Cells were plated in 96 wells plates (4000 cells/well). Twenty-four hours after seeding, cells were exposed to chemicals. After 48 h of treatment, cells were washed in PBS and fixed in cooled 90% ethanol/5% acetic acid for 20 min and the nuclei were stained with Hoechst 33342 (B2261 Sigma). Image acquisition and analysis were performed using a CellomicsArrayScan VTI/HCS Reader (ThermoScientific, Waltham, MA, USA). The survival percentages were calculated as the percentage of cell number after compound treatment over cell number after DMSO treatment.

### 3.4. Molecular Modelling Studies

#### 3.4.1. Molecular Docking

To investigate binding modes of ***DB18*** analogues, the experimental 3D structures of human CLK proteins were taken from the PDB: 5X8I (*Hs*CLK1), 6FYV (*Hs*CLK2), 6FYL (*Hs*CLK3), 2WU6 (*Hs*CLK4) and 4AZE (*Hs*DYRK1A). The co-crystal ligands present in ATP-binding site were employed to define the active sites and generate docking grids. Glide 9.4 module implemented in Maestro 13.1 suite (Schrodinger LLC) was used for docking. The ligands were drawn using 2D-sketcher, converted into 3D, and submitted to Macromodel conformational searching. Molecular docking was performed with induced-fit option, employing extra precision mode.

#### 3.4.2. Molecular Dynamics

The Desmond system and OPLS_2005 force field were used for molecular dynamics experiments (D.E. Shaw Research). Briefly, the protein complexes were soaked with the SPC model of water and ions if needed, and periodic boundary conditions were built. For each complex, the solvent was first optimized through energy minimization, keeping protein-ligand complex frozen. The system was thereafter optimized, keeping only protein backbone frozen, before a short molecular dynamics run of 50 ns with the same constraints. NPT conditions (NHC thermostat at 1 atm pressure, T = 300 K) and default parameters were used. Finally, constraints were released for the molecular dynamics production, carried out for 1 µs per complex system, with trajectory sampling every 2 ns. For *Hs*CLK1, 13,235 water molecules were used, and 13,028 water molecules and one chloride ion were necessary for *Mm*CLK1.

## 4. Conclusions

The compound ***DB18*** was established recently as a very potent inhibitor of the human CLKs kinases, with a remarkable selectivity vs. human DYRK1A. Starting from this lead molecule, we designed three series of analogues in order to explore the roles of the different parts of this compound: -the substituents on the aniline, -the tolyl group in the top part of ***DB18*** anchored into CLK1 and,-the central part of this lead.

Fifteen new analogues have been designed, prepared and analysed for their inhibitory potency on various CLKs plus DYRK1A kinases, as well as for cytotoxicity studies. SAR data were analyzed through molecular docking experiments which afforded useful rationales for the observed activities. Furthermore, molecular dynamic studies highlighted the importance of protein kinases flexibility to explain inhibitors selectivity.

Although it had a slightly different binding mode compared to ***DB18***, 12 gemerged as the most promising compound for this new series of molecules. It exhibited CLK inhibitory properties similar to those obtained with ***DB18***, albeit with a slight drop in selectivity vs. DYRK1A. Based on these results, a kinome scan assay was performed on ***DB18*** on 463 human kinases. It fully demonstrated the high selectivity of this molecule on CLKs, with limited extra activity only on HIPKs and TRKA. 

Altogether, these results confirmed the qualities of the 2-anilinoquinazoline scaffold in the design of CLK inhibitors and the potentialities of ***DB18*** and **12g** as potent inhibitors of human CLK-1, -2 and -4 kinases, with low to no action on DYRK1A. Therefore, these compounds should be useful tools, for example, to explore the possible roles of CLKs’ kinases in various aspects of human health.

## Figures and Tables

**Table 1:** IC_50_ data (µM) for the inhibition of human kinases CLK1-4 and DYRK1A by the quinazolines ***DB18*** and **7a** and **7b**. Each concentration of compound was tested in duplicate.

Entry	N°	*Hs*CLK1	*Hs*CLK2	*Hs*CLK3	*Hs*CLK4	*Hs*DYRK1A
1	***DB18*** ^[a]^	0.011	0.027	1.280	0.020	>100
2	**7a**	1.100	ND ^[b]^	ND	ND	ND
3	**7b**	0.085	0.097	3.313	0.380	>100

^[a]^ Data taken from reference14, ^[b]^ Not determined.

**Table 2:** IC_50_ data (µM) for the inhibition of human kinases CLK1-4 and DYRK1A by the quinazolines **12**. Each concentration of compound was tested in duplicate.

Entry	N°	*Hs*CLK1	*Hs*CLK2	*Hs*CLK3	*Hs*CLK4	*Hs*DYRK1A
1	**12d**	0.378	ND	ND	ND	ND
2	**12c**	1.64–1.37	ND	ND	ND	ND
3	**12e**	0.146	0.050	7.490	0.195	2.330
4	**12f**	0.293	ND ^[a]^	ND	ND	ND
5	**12g**	0.004	0.028	0.339	0.055	3.940

^[a]^ Not determined.

**Table 3:** IC_50_ data (μM) for the inhibition of human kinases CLK1-4 and DYRK1A by the quinazolines **16** and **19**. Each concentration of compound was tested in duplicate.

Entry	N°	*Hs*CLK1	*Hs*CLK2	*Hs*CLK3	*Hs*CLK4	*Hs*DYRK1A
1	**16a**	0.146	0.050	7.490	0.195	2.760
2	**16b**	0.053	0.020	3.255	0.083	1.900
3	**19a**	ND ^[a]^	ND	ND	ND	3.720
4	**19b**	ND	ND	ND	ND	5.440

^[a]^ Not determined.

**Table 4:** Geometric properties of inhibitors during molecular dynamics of the complexes with CLK1 (mean value). The last line represents bar plots of the dihedral (probability density of torsion during the simulation). Note the higher flexibility of **7a**, as evidenced by the larger standard deviation of its dihedral, as compared with ***DB18***. The halogen/π bond is symbolized by a dotted line on the chemical structure (right).

	7a	*DB18*	
d_1_ (Å)	6.5 ± 1.2	7.1 ± 1.0	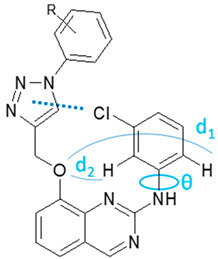
d_2_ (Å)	6.2 ± 0.5	6.3 ± 0.4	
Ɵ (°)	66 ± 64	148 ± 45	
	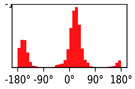	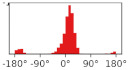	

**Table 5:** IC_50_ data (μM) for the inhibition of *Mm*CLK1 by selectednew quinazolines.

Number	*Mm*ClK1	Number	*Mm*CLK1	Number	*Mm*CLK1
Series 1		Series 2		Series 3	
***DB18***	0.120	**12d**	0.391	**16b**	0.180
**7a**	0.466	**12c**	0.177	**19a**	0.200
**7b**	0.280–0.369	**12e**	0.191	**16a**	0.220
		**12f**	0.245	**19b**	0.210
		**12g**	0.024		

## Data Availability

Not applicable.

## Supplementary Materials

The following supporting information can be downloaded at: https://www.mdpi.com/article/10.3390/molecules27196149/s1, Part 1: Biology & Molecular Modelling. Table S1: Primary evaluation of the inhibition of synthetized quinazolines against a short panel of mammalian kinases; Table S2: Cytotoxic studies (IC50, in μM) of the synthetized quinazolines; Figure S1: Secondary structure composition of the *Hs*CLK1/***DB18*** (up) and *Hs*CLK1/**7a** (down) complex during 1 μs molecular dynamics; Figure S2: RMSD of DB35 during 1 μs molecular dynamics; Figures S3 and S4: Interactive poses of **12c** & **12e** ligands with *Hs*CLK1; Figure S5: Interactive poses of DB35 ligand with *Hs*CLK1; Figures S6 and S7: Interactive poses of **15a**, **15b**, **18a** & **18b** ligands with HsCLK1; Figures S8 and S9: Interactive poses of **16b**, **19a**, **16a** & **19b** ligands with *Hs*CLK1; Figure S10: Interactive poses of **7b**, ***DB18***, **16b**, **16a** & **12g** ligands with *Hs*DYRK1A; Table S3: Prime-based MM-GBSA energies in CLK1; Table S4: Prime-based MM-GBSA energies in DYRK1A; Table S5: Full data of kinome scan study for ***DB18***: Inhibition of compounds against mammalian kinases; Cytotoxicity studies; Molecular docking/dynamics experiments; Full data of kinome scan study of ***DB18***; Part 2: Views of NMR spectra.

## Sample Availability

Samples of the compounds are available from authors.
