# Supplementary material for: Structure Activity Relationship Studies around DB18, a Potent and Selective Inhibitor of CLK Kinases
Source: Molecules. 2022 Sep 20;27(19):6149. doi: 10.3390/molecules27196149 (PMC9571063; doi:10.3390/molecules27196149)
Supplement: Supplementary file 1 [file molecules-27-06149-s001.zip › 2022 DB3 Molecules SI part 1.pdf]

Submitted to Molecules

Structure Activity Relationship Studies around DB18, a Potent and Selective Inhibitor of CLK Kinases

Dabbugoddu Brahmaiah [1,2], Anagani Kanaka Durga Bhavani [3,*], Pasula Aparna [2], Nangunoori Sampath Kumar [1], Hélène Solhi [4], Rémy Le Guevel [4], Blandine Baratte [5,6], Thomas Robert [5,6], Sandrine Ruchaud [6], Stéphane Bach [5,6,7], Surender Singh Jadav [8], Chada Raji Reddy [8], Paul Mosset [9], Nicolas Gouault [9], Nicolas Levoin [10,*] and René Grée [9,*]

[1] Chemveda Life Sciences India Pvt. Ltd., #B-11/1, IDA Uppal, Hyderabad-500039, Telangana, India
[2] Jawaharlal Nehru Technological University Hyderabad, Kukatpally, Hyderabad-500 085, Telangana, India
[3] Department of Chemistry, Osmania University, Hyderabad 500007, Telangana, India
[4] Univ Rennes, PlateformImPACcell, BIOSIT, F-35000 Rennes, France
[5] Sorbonne Université, CNRS, FR 2424, Plateforme de criblage KISSf (Kinase Inhibitor Specialized Screening facility), Station Biologique de Roscoff, CS 90074, 29688 Roscoff Cedex, France
[6] Sorbonne Université, CNRS, UMR 8227, Integrative Biology of Marine Models Laboratory (LBI2M), Station Biologique de Roscoff, CS 90074, 29688 Roscoff Cedex, France
[7] Centre of Excellence for Pharmaceutical Sciences, North-West University, Private Bag X6001, Potchefstroom 2520, South Africa
[8] CSIR-Indian Institute of Chemical Technology, Uppal Road, Tarnaka, Hyderabad 500007, TS, India
[9] Univ Rennes, CNRS, ISCR (Institut des Sciences Chimiques de Rennes), UMR 6226, F-35000 Rennes, France
[10] Bioprojet-Biotech, 4 rue du Chesnay Beauregard, BP 96205, 35762 Saint Grégoire, France
* Correspondence: akdbhavani4ou@gmail.com (A.K.D.B.); n.levoin@bioprojet.com (N.L.); rene.gree@univ-rennes1.fr (R.G.)

Supporting Information

PART 1

Biology & Molecular Modelling

-ESI Table S1 Primary evaluation of the inhibition of synthetized quinazolines against a short panel of mammalian kinases: Page S2

-ESI Table S2 Cytotoxic studies (IC_{50}, in µM) of the synthetized quinazolines: Page S3

-ESI Figure S1. Secondary structure composition of the *Hs*CLK1/***DB18*** (up) and *Hs*CLK1/**7a** (down) complex during 1 µs molecular dynamics: Page S4

-ESI Figure S2 RMSD of ***DB35*** during 1µs molecular dynamics: Page S4

-ESI Figures S3 and S4: Interactive poses of **12c** & **12e** ligands with *Hs*CLK1 Page S5

-ESI Figure S5: Interactive poses of **DB35** ligand with *Hs*CLK1 Page S6

-ESI Figures S6 and S7: Interactive poses of **15a**, **15b**, **18a** & **18b** ligands with *Hs*CLK1 Page S7

Page S8

-ESI Figures S8 and S9: Interactive poses of **16b**, **19a**, **16a** & **19b** ligands with *Hs*CLK1 Page S9

-ESI Figure S10 Interactive poses of **7b**, ***DB18***, **16b**, **16a** & **12g** ligands with *Hs*DYRK1A: Page S10

-ESI Table S3 Prime-based MM-GBSA energies in CLK1: Page S10

-ESI Table S4 Prime-based MM-GBSA energies in DYRK1A: Pages S11-S23

-ESI Table S5 Full data of kinome scan study for ***DB18***: Page S24

-TREE*spot*™ Interaction Maps for ***DB18***

ESI Table S1 Primary evaluation of the inhibition of synthetized quinazolines against a short panel of mammalian kinases:

	Compound Id	Concentration	Hs_CDK5/ p25	Hs_CDK9/ CyclinT	Hs_PIM1	Mm_CLK1	Rn_DYRK1A	Hs_DYRK1A	Hs_HASPIN	Hs_GSK3β	Hs_CK1ε
	DB18 [(a)]	10 µM	84	54	81	11	75	74	94	57	81
		1 µM	≥100	90	97	21	≥100	≥100	96	95	90
Series 1	7a	10 µM	65	45	60	30	90	92	94	70	89
		1 µM	≥100	55	81	43	56	≥100	95	87	93
	7b	10 µM	68	34	84	17	95	80	46	36	47
		1 µM	93	80	87	14	87	≥100	81	85	81
Series 2	12d	10 µM	≥100	43	70	22	76	77	95	75	90
		1 µM	≥100	85	85	27	≥100	≥100	91	≥100	≥100
	12c	10 µM	30	39	68	20	45	60	88	60	81
		1 µM	71	58	59	36	81	≥100	83	89	94
	12e	10 µM	54	21	69	35	34	31	88	35	52
		1 µM	≥100	74	96	74	80	84	92	99	≥100
	12f	10 µM	61	45	66	18	83	93	76	69	95
		1 µM	≥100	73	73	28	≥100	≥100	73	99	≥100
	12g (DB35)	10 µM	97	21	31	5	39	50	69	19	72
		1 µM	45	38	66	13	61	86	75	76	98
Series 3	15a	10 µM	96	55	74	60	97	63	74	86	97
		1 µM	≥100	64	86	98	≥100	95	92	≥100	≥100
	15b	10 µM	68	61	73	73	≥100	74	58	57	81
		1 µM	≥100	92	75	96	95	≥100	89	93	≥100
	18a	10 µM	58	59	74	70	≥100	66	70	60	78
		1 µM	69	92	77	93	92	≥100	77	88	≥100
	18b	10 µM	81	83	68	72	≥100	85	53	35	92
		1 µM	85	98	94	86	≥100	≥100	72	75	≥100
	16a	10 µM	44	18	15	8	20	38	76	8	24
		1 µM	87	61	69	19	71	85	76	47	82
	16b	10 µM	30	30	40	10	64	34	92	23	22
		1 µM	≥100	65	62	20	78	84	70	50	73
	19a	10 µM	36	12	14	8	33	36	31	49	21
		1 µM	75	68	54	17	82	84	72	32	87
	19b	10 µM	42	42	44	7	92	55	90	15	20
		1 µM	≥100	91	79	12	92	≥100	95	44	63

[(a)] Values taken from ref 13.

Legend: Kinase inhibitory activity of compounds expressed in % of residual activity at two concentrations (10 µM and 1 µM). Kinase are from human origin (*Homo sapiens*) unless specified: *Rn*, *Rattus norvegicus* or *Mm*, *Mus musculus*.
100% of residual activity is mesured in the absence of inhibitor. Values are means, n=2.

ESI Table S2 Cytotoxic studies (IC_{50}, in µM) of the synthetized quinazolines

	Ic_{50} µM	**HuH7**	**Caco2**	**MDA-MB-231**	**MDA-MB-468**	**HCT116**	**MCF7**	**Fibroblasts**
	**DB18**[a]	25	37	>25	ND	>25	4	21
Series 1								
	**7a**	3	ND	9	>25	7	7	>25
	**7b**	>25	>25	>25	>25	>25	24	>25
**Series 2**								
	**12d**	2	>25	>25	>25	>25	>25	13 (moderate amplitude)
	**12c**	5	24	19	4	1.3	9	6 (moderate amplitude)
	**12e**	3	7	6	4	3	4	6 (moderate amplitude)
	**12f**	>25	>25	>25	>25	>25	>25	>25
	**12g (DB35)**	>25	>25	>25	>25	>25	>25	>25
**Series 3**								
	**15a**	7	>25	3	18	7	23	>25
	**15b**	20	21	8	>25	>25	>25	>25
	**18a**	4	16	7	15	5	>25	>25
	**18b**	10	>25	>25	>25	>25	>25	>25
	**16b**	2	3	2	1	2.4	3	2
	**19a**	8	11	6	7	7	11	1.3 (low amplitude)
	**16a**	8	9	9	9	10	8	9 (high amplitude)
	**19b**	14	3	2	1.3	4	3	3 (high amplitude)

ESI Figure S1. Secondary structure composition of the *Hs*CLK1/***DB18*** (up) and *Hs*CLK1/**7a** (down) complex during 1 µs molecular dynamics. β-strands are a little more present in the former (black ellipses).

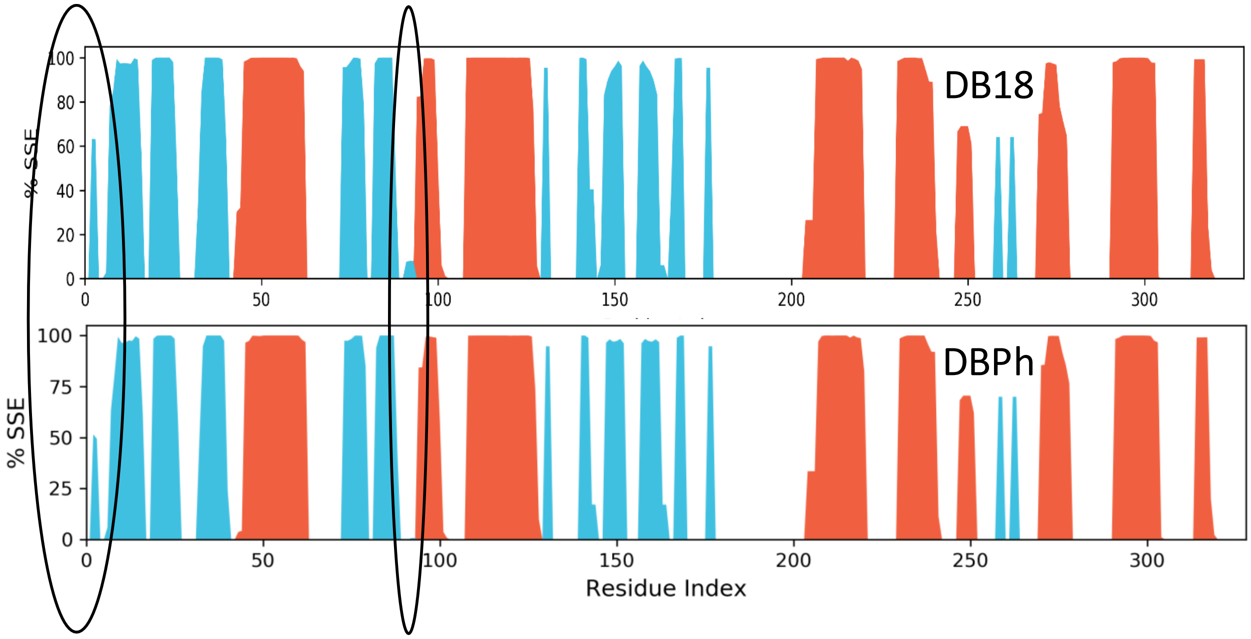

ESI Figure S2 RMSD of ***DB35*** during 1µs molecular dynamics. The initial binding mode was exactly that of ***DB18***. It evolved afterwards (note the strong shift around 300 ns), and became superimposable to the predicted docking pose.

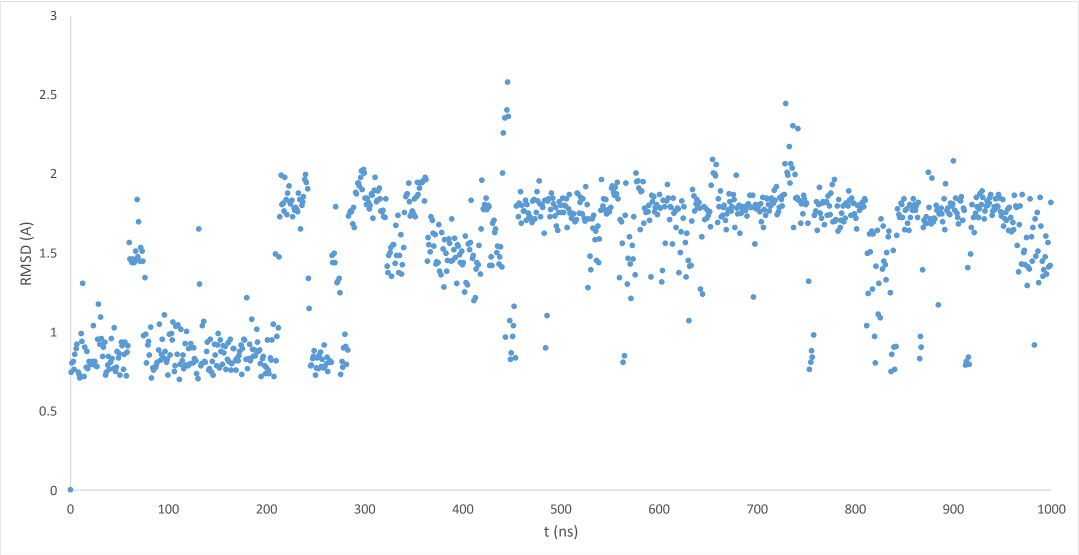

ESI Figures S3 and S4: Interactive poses of **12c** & **12e** ligands with *Hs*CLK1.

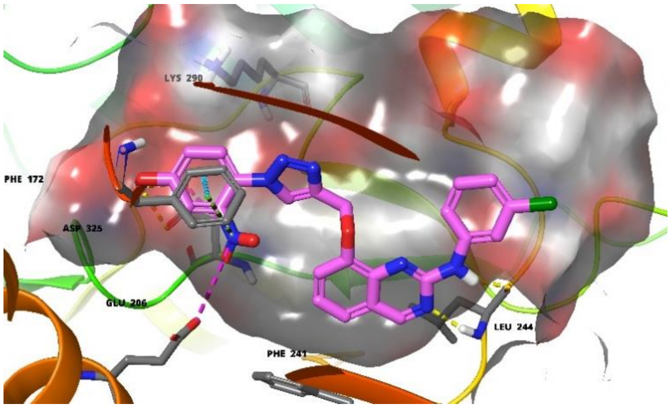

Figure S3 12c in CLK1 cavity; The ligands are shown as thick residues in colour faded magenta. Colour coding: oxygen-red, nitrogen-blue, carbon-grey in case of protein. Backbone, side chain hydrogen bonds are shown as yellow dotted lines, π-π stackings are given in light blue dotted lines whereas, charged interactions are provided in pink dotted lines. To focus the interactions of active site residues with ligands 6.0 Å surface area of protein has been shown in the figures.

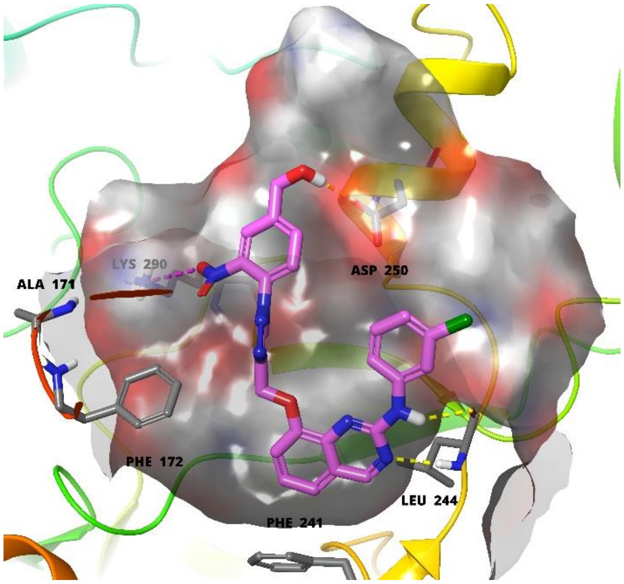

Figure S4 12e in CLK1 cavity; The ligands are shown as thick residues in colour faded magenta. Colour coding: oxygen-red, nitrogen-blue, carbon-grey in case of protein. Backbone, side chain hydrogen bonds are shown as yellow dotted lines, π-π stackings are given in light blue dotted lines whereas, charged interactions are provided in pink dotted lines. To focus the interactions of active site residues with ligands 6.0 Å surface area of protein has been shown in the figures.

ESI Figure S5: Interactive poses of **DB35** ligand with *Hs*CLK1.

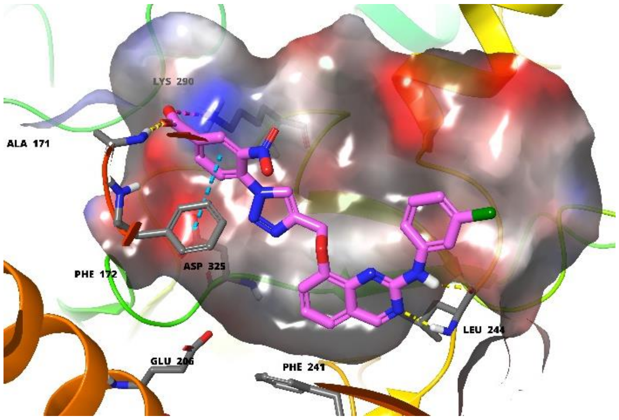

Figure S5 DB35 in CLK1 cavity; The ligands are shown as thick residues in colour faded magenta. Colour coding: oxygen-red, nitrogen-blue, carbon-grey in case of protein. Backbone, side chain hydrogen bonds are shown as yellow dotted lines, π-π stackings are given in light blue dotted lines whereas, charged interactions are provided in pink dotted lines. To focus the interactions of active site residues with ligands 6.0 Å surface area of protein has been shown in the figures.

ESI Figures S6 and S7: Interactive poses of **15a**, **15b**, **18a** & **18b** ligands with *Hs*CLK1.

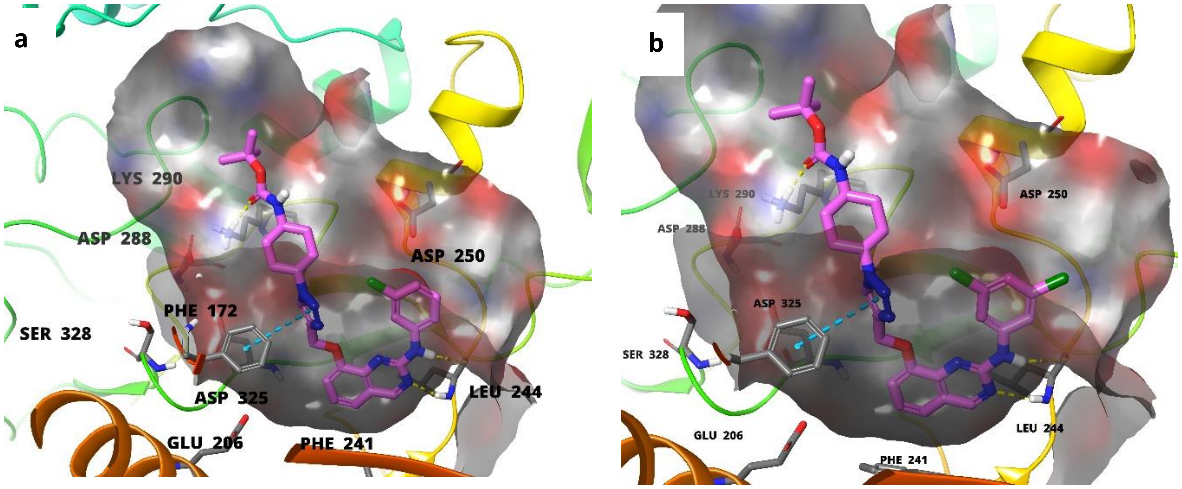

Figure S6 (a) & (b) 15a and **15b** in CLK1 cavity; The ligands are shown as thick residues in colour faded magenta. Colour coding: oxygen-red, nitrogen-blue, carbon-grey in case of protein. Backbone, side chain hydrogen bonds are shown as yellow dotted lines, π-π stackings are given in light blue dotted lines whereas, charged interactions are provided in pink dotted lines. To focus the interactions of active site residues with ligands 6.0 Å surface area of protein has been shown in the figures.

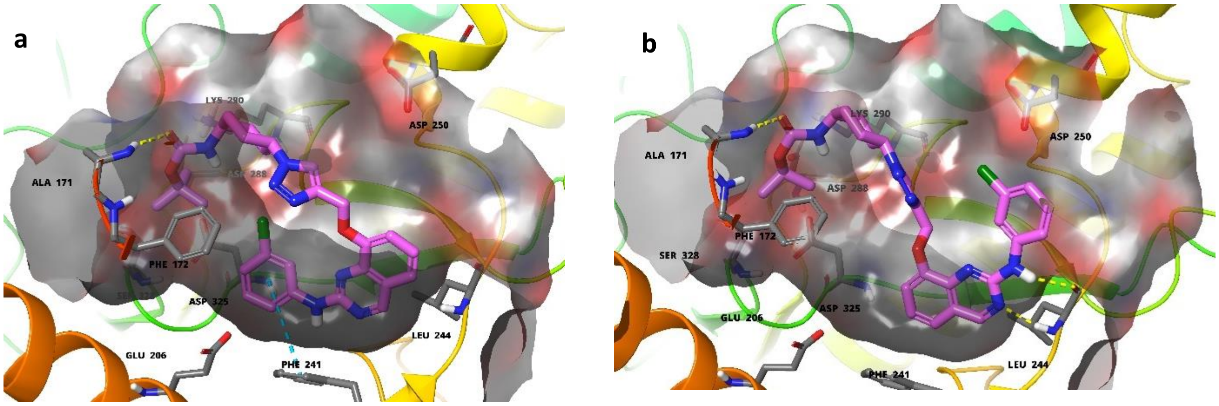

Figure S7 (a)& (b) 18a and **18b** in CLK1 cavity; The ligands are shown as thick residues in colour faded magenta. Colour coding: oxygen-red, nitrogen-blue, carbon-grey in case of protein. Backbone, side chain hydrogen bonds are shown as yellow dotted lines, π-π stackings are given in light blue dotted lines whereas, charged interactions are provided in pink dotted lines. To focus the interactions of active site residues with ligands 6.0 Å surface area of protein has been shown in the figures.

- **ESI Figures S8 and S9**: Interactive poses of **16b**, **19a**, **16a** & **19b** ligands with *Hs*CLK1.

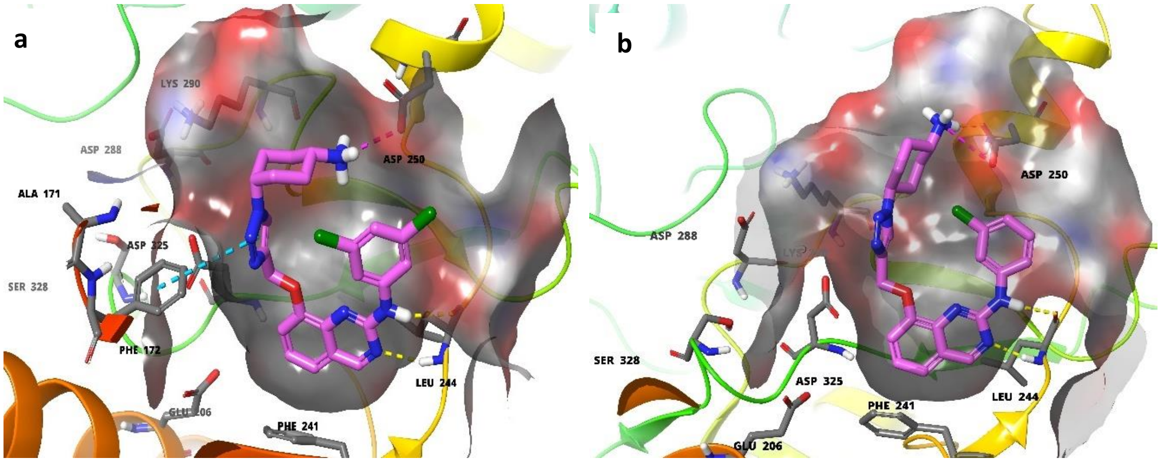

Figure S8: a & b) 16b and **19a** in CLK1 cavity; The ligands are shown as thick residues in colour faded magenta. Colour coding: oxygen-red, nitrogen-blue, carbon-grey in case of protein. Backbone, side chain hydrogen bonds are shown as yellow dotted lines, π-π stackings are given in light blue dotted lines whereas, charged interactions are provided in pink dotted lines. To focus the interactions of active site residues with ligands 6.0 Å surface area of protein has been shown in the figures.

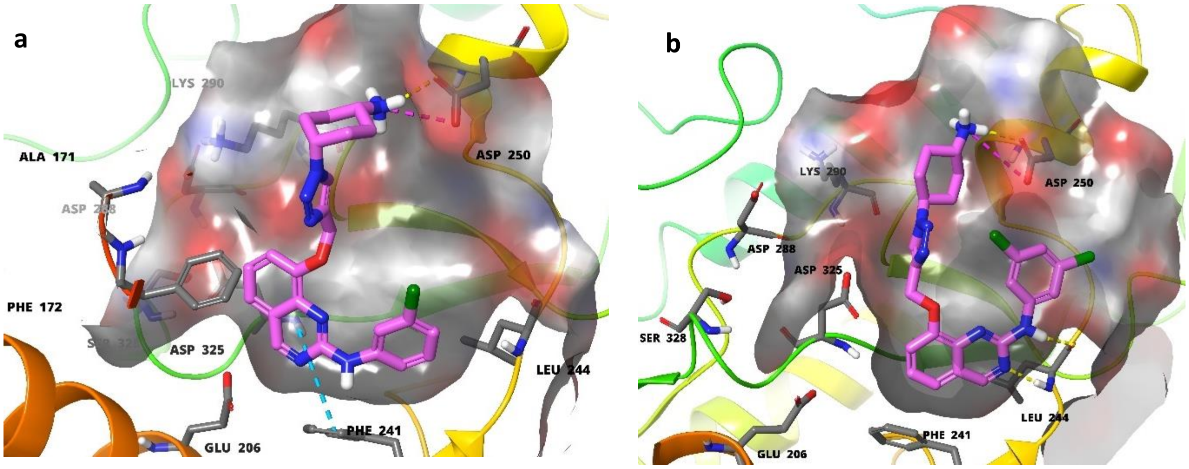

Figure S9: a & b) 16a and **19b** in CLK1 cavity; The ligands are shown as thick residues in colour faded magenta. Colour coding: oxygen-red, nitrogen-blue, carbon-grey in case of protein. Backbone, side chain hydrogen bonds are shown as yellow dotted lines, π-π stackings are given in light blue dotted lines whereas, charged interactions are provided in pink dotted lines. To focus the interactions of active site residues with ligands 6.0 Å surface area of protein has been shown in the figures.

ESI Figure S10 Interactive poses of **7b**, **DB18**, **16b**, **16a** & **12g** ligands with *Hs*DYRK1A.

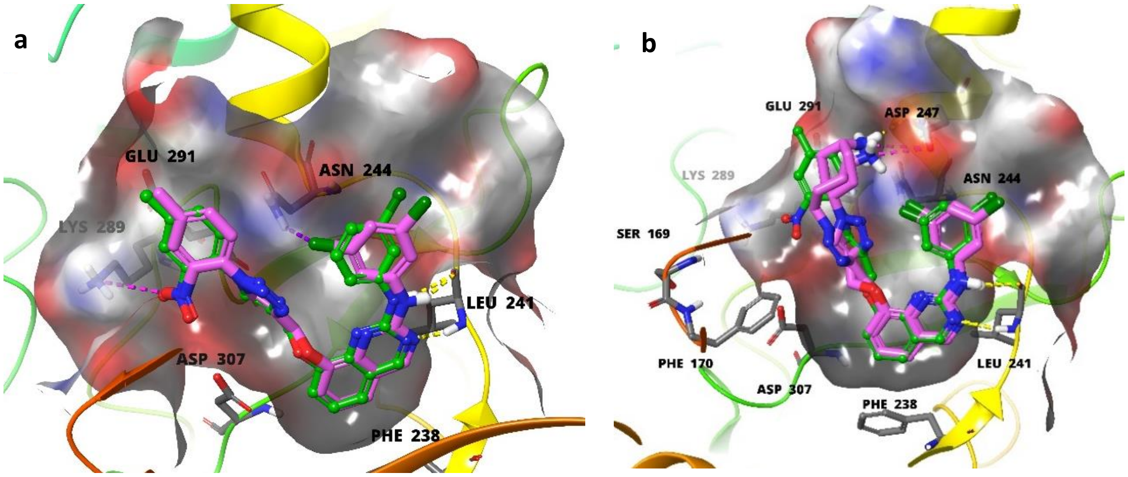

ESI Figure S10: a) Overlap of *meta meta'* 3,5 dichloro derivative **7b** with *mono* 3-chloro substituted previous standard ***DB18*** in DYRK1A cavity; b) comparison of *meta meta'* 3,5 dichloro derivative **16b**, mono chloro analogue **16a** with ***DB18*** in DYRK1A active site. The ligands **7b, 16b, 16a** and **12g** (***DB35***) are shown as thick residues in colour faded magenta and ***DB18*** is highlighted in green ball and stick models. Colour coding: oxygen-red, nitrogen-blue, carbon-grey in case of protein. Background covers the rest of the protein chain in ribbon model and all the images were prepared using Maestro 13.1. Backbone, side chain hydrogen bonds are shown as yellow dotted lines, π-π stackings are given in light blue dotted lines whereas, charged interactions are provided in pink dotted lines. To focus the interactions of active site residues with ligands 6.0 Å surface area of protein has been shown in the figures.

ESI Table S3. Prime-based MM-GBSA energies in *Hs*CLK1

	MM-GBSA/CLK1 (kcal/mole)				
	**ΔdG binding**	**ΔdGhbond**	**ΔdGLipo**	**ΔdG packing**	**ΔdGvdw**
DB18	-70.28	-1.32	-39.82	-0.17	-56.77
**7a**	-62.64	-1.02	-24.64	-2.06	-60.36
**7b**	-88.74	-1.35	-43.40	-2.00	-54.15
**12c**	-70.00	-1.65	-26.56	-4.27	-62.13
**12e**	-64.32	-1.80	-20.03	-1.46	-51.74
**12g (*DB35*)**	-66.26	-2.00	-24.93	-2.98	-62.56
**15b**	-65.16	-1.74	-21.76	-1.76	-58.92
**16a**	-61.41	-2.00	-24.22	-1.19	-59.32
**16b**	-73.29	-1.85	-28.26	-2.29	-63.24
**18a**	-66.19	-0.41	-27.20	-1.95	-68.75
**18b**	-70.58	-1.63	-23.57	-0.97	-61.41
**19a**	-69.13	-2.41	-23.79	-1.22	-60.36
**19b**	-70.76	-2.14	-21.79	-1.17	-58.72

ESI Table S4. Prime-based MM-GBSA energies in *Hs*DYRK1A

	MM-GBSA/DYRK1A (kcal/mole)				
	**ΔdG binding**	**ΔdGhbond**	**ΔdGLipo**	**ΔdG packing**	**ΔdGvdw**
DB18	-65.14	-1.27	-20.69	-3.04	-58.05
**7a**	-59.38	-1.06	-20.00	-2.21	-52.69
**7b**	-60.94	-1.05	-1.04	-2.85	-55.79
**12c**	-60.76	-1.06	-19.67	-3.01	-54.55
**12e**	-64.27	-2.94	-21.20	-2.05	-54.24
**12g (*DB35*)**	-66.79	-2.94	-24.97	-2.61	-64.63
**15a**	-55.62	-1.78	-24.54	-1.06	-61.99
**15b**	-59.75	-1.23	-19.67	-1.31	-56.93
**16a**	-61.21	-3.09	-22.97	-0.92	-59.02
**16b**	-55.35	-2.01	-21.83	-1.56	-51.99
**18a**	-55.27	-0.87	-23.48	-2.01	-60.22
**18b**	-65.08	-1.35	-26.43	-2.76	-70.40
**19a**	-56.24	-1.55	-23.29	-1.79	-57.46
**19b**	-55.35	-2.02	-21.82	-1.56	-51.97

ESI Table S5:Matrix of screen for the selectivity profiling of DB18 (related to Figure 6). The table reports the full list of the 468 kinases used in the selectivity profiling of ***DB18***. ***DB18*** was assayed at 1 µM and results are reported as "% Ctrl", where lower numbers indicate stronger hits in the panel of kinases tested. %Ctrl calculation: (test compound signal – positive control signal)/(negative control signal - positive control signal) x 100. Test compound = ***DB18***; negative control = DMSO (100%Ctrl); positive control = control compound (0%Ctrl).

KINOMEscan[SM] (468 kinases) on ***DB18***		
**DiscoveRx Gene Symbol**	**Entered Gene Symbol**	**Percent Control**
AAK1	AAK1	100
ABL1(E255K)-phosphorylated	ABL1	97
ABL1(F317I)-nonphosphorylated	ABL1	100
ABL1(F317I)-phosphorylated	ABL1	100
ABL1(F317L)-nonphosphorylated	ABL1	100
ABL1(F317L)-phosphorylated	ABL1	100
ABL1(H396P)-nonphosphorylated	ABL1	96
ABL1(H396P)-phosphorylated	ABL1	98
ABL1(M351T)-phosphorylated	ABL1	99
ABL1(Q252H)-nonphosphorylated	ABL1	88
ABL1(Q252H)-phosphorylated	ABL1	100
ABL1(T315I)-nonphosphorylated	ABL1	89
ABL1(T315I)-phosphorylated	ABL1	100
ABL1(Y253F)-phosphorylated	ABL1	97
ABL1-nonphosphorylated	ABL1	85
ABL1-phosphorylated	ABL1	98
ABL2	ABL2	100
ACVR1	ACVR1	100
ACVR1B	ACVR1B	88
ACVR2A	ACVR2A	100
ACVR2B	ACVR2B	100
ACVRL1	ACVRL1	93
ADCK3	CABC1	100
ADCK4	ADCK4	100
AKT1	AKT1	87
AKT2	AKT2	100
AKT3	AKT3	100
ALK	ALK	100
ALK(C1156Y)	ALK	99
ALK(L1196M)	ALK	98

AMPK-alpha1	PRKAA1	94
AMPK-alpha2	PRKAA2	91
ANKK1	ANKK1	100
ARK5	NUAK1	100
ASK1	MAP3K5	100
ASK2	MAP3K6	99
AURKA	AURKA	100
AURKB	AURKB	99
AURKC	AURKC	100
AXL	AXL	87
BIKE	BMP2K	99
BLK	BLK	100
BMPR1A	BMPR1A	68
BMPR1B	BMPR1B	96
BMPR2	BMPR2	100
BMX	BMX	100
BRAF	BRAF	93
BRAF(V600E)	BRAF	96
BRK	PTK6	100
BRSK1	BRSK1	100
BRSK2	BRSK2	97
BTK	BTK	100
BUB1	BUB1	97
CAMK1	CAMK1	100
CAMK1B	PNCK	86
CAMK1D	CAMK1D	100
CAMK1G	CAMK1G	100
CAMK2A	CAMK2A	96
CAMK2B	CAMK2B	100
CAMK2D	CAMK2D	100
CAMK2G	CAMK2G	100
CAMK4	CAMK4	100
CAMKK1	CAMKK1	100
CAMKK2	CAMKK2	100
CASK	CASK	99
CDC2L1	CDK11B	100
CDC2L2	CDC2L2	100
CDC2L5	CDK13	100
CDK11	CDK19	100

CDK2	CDK2	100
CDK3	CDK3	100
CDK4	CDK4	100
CDK4-cyclinD1	CDK4	93
CDK4-cyclinD3	CDK4	100
CDK5	CDK5	100
CDK7	CDK7	100
CDK8	CDK8	100
CDK9	CDK9	100
CDKL1	CDKL1	88
CDKL2	CDKL2	97
CDKL3	CDKL3	94
CDKL5	CDKL5	100
CHEK1	CHEK1	100
CHEK2	CHEK2	100
CIT	CIT	100
CLK1	CLK1	9.5
CLK2	CLK2	1.4
CLK3	CLK3	29
CLK4	CLK4	23
CSF1R	CSF1R	100
CSF1R-autoinhibited	CSF1R	71
CSK	CSK	100
CSNK1A1	CSNK1A1	94
CSNK1A1L	CSNK1A1L	100
CSNK1D	CSNK1D	100
CSNK1E	CSNK1E	100
CSNK1G1	CSNK1G1	100
CSNK1G2	CSNK1G2	90
CSNK1G3	CSNK1G3	100
CSNK2A1	CSNK2A1	75
CSNK2A2	CSNK2A2	61
CTK	MATK	96
DAPK1	DAPK1	100
DAPK2	DAPK2	100
DAPK3	DAPK3	98
DCAMKL1	DCLK1	88
DCAMKL2	DCLK2	100
DCAMKL3	DCLK3	100

DDR1	DDR1	100
DDR2	DDR2	100
DLK	MAP3K12	100
DMPK	DMPK	100
DMPK2	CDC42BPG	100
DRAK1	STK17A	100
DRAK2	STK17B	100
DYRK1A	DYRK1A	97
DYRK1B	DYRK1B	59
DYRK2	DYRK2	90
EGFR	EGFR	97
EGFR(E746-A750del)	EGFR	82
EGFR(G719C)	EGFR	100
EGFR(G719S)	EGFR	100
EGFR(L747-E749del, A750P)	EGFR	93
EGFR(L747-S752del, P753S)	EGFR	83
EGFR(L747-T751del,Sins)	EGFR	100
EGFR(L858R)	EGFR	94
EGFR(L858R,T790M)	EGFR	100
EGFR(L861Q)	EGFR	99
EGFR(S752-I759del)	EGFR	91
EGFR(T790M)	EGFR	100
EIF2AK1	EIF2AK1	100
EPHA1	EPHA1	95
EPHA2	EPHA2	100
EPHA3	EPHA3	100
EPHA4	EPHA4	88
EPHA5	EPHA5	100
EPHA6	EPHA6	100
EPHA7	EPHA7	100
EPHA8	EPHA8	100
EPHB1	EPHB1	100
EPHB2	EPHB2	100
EPHB3	EPHB3	100
EPHB4	EPHB4	100
EPHB6	EPHB6	95
ERBB2	ERBB2	57
ERBB3	ERBB3	99
ERBB4	ERBB4	100

ERK1	MAPK3	100
ERK2	MAPK1	100
ERK3	MAPK6	100
ERK4	MAPK4	100
ERK5	MAPK7	100
ERK8	MAPK15	95
ERN1	ERN1	94
FAK	PTK2	100
FER	FER	100
FES	FES	100
FGFR1	FGFR1	100
FGFR2	FGFR2	100
FGFR3	FGFR3	100
FGFR3(G697C)	FGFR3	97
FGFR4	FGFR4	100
FGR	FGR	96
FLT1	FLT1	100
FLT3	FLT3	84
FLT3(D835H)	FLT3	55
FLT3(D835V)	FLT3	67
FLT3(D835Y)	FLT3	29
FLT3(ITD)	FLT3	62
FLT3(ITD,D835V)	FLT3	53
FLT3(ITD,F691L)	FLT3	94
FLT3(K663Q)	FLT3	69
FLT3(N841I)	FLT3	77
FLT3(R834Q)	FLT3	87
FLT3-autoinhibited	FLT3	100
FLT4	FLT4	100
FRK	FRK	83
FYN	FYN	93
GAK	GAK	100
GCN2(Kin.Dom.2,S808G)	EIF2AK4	100
GRK1	GRK1	97
GRK2	ADRBK1	94
GRK3	ADRBK2	89
GRK4	GRK4	100
GRK7	GRK7	100
GSK3A	GSK3A	100

GSK3B	GSK3B	90
HASPIN	GSG2	72
HCK	HCK	100
HIPK1	HIPK1	19
HIPK2	HIPK2	33
HIPK3	HIPK3	46
HIPK4	HIPK4	17
HPK1	MAP4K1	100
HUNK	HUNK	90
ICK	ICK	81
IGF1R	IGF1R	100
IKK-alpha	CHUK	100
IKK-beta	IKBKB	100
IKK-epsilon	IKBKE	96
INSR	INSR	93
INSRR	INSRR	100
IRAK1	IRAK1	100
IRAK3	IRAK3	59
IRAK4	IRAK4	100
ITK	ITK	100
JAK1(JH1domain-catalytic)	JAK1	100
JAK1(JH2domain-pseudokinase)	JAK1	100
JAK2(JH1domain-catalytic)	JAK2	100
JAK3(JH1domain-catalytic)	JAK3	93
JNK1	MAPK8	93
JNK2	MAPK9	96
JNK3	MAPK10	95
KIT	KIT	100
KIT(A829P)	KIT	100
KIT(D816H)	KIT	100
KIT(D816V)	KIT	100
KIT(L576P)	KIT	100
KIT(V559D)	KIT	100
KIT(V559D,T670I)	KIT	96
KIT(V559D,V654A)	KIT	100
KIT-autoinhibited	KIT	97
LATS1	LATS1	99
LATS2	LATS2	100
LCK	LCK	100

LIMK1	LIMK1	100
LIMK2	LIMK2	100
LKB1	STK11	100
LOK	STK10	100
LRRK2	LRRK2	100
LRRK2(G2019S)	LRRK2	95
LTK	LTK	80
LYN	LYN	93
LZK	MAP3K13	100
MAK	MAK	100
MAP3K1	MAP3K1	98
MAP3K15	MAP3K15	93
MAP3K2	MAP3K2	97
MAP3K3	MAP3K3	99
MAP3K4	MAP3K4	100
MAP4K2	MAP4K2	100
MAP4K3	MAP4K3	95
MAP4K4	MAP4K4	100
MAP4K5	MAP4K5	100
MAPKAPK2	MAPKAPK2	100
MAPKAPK5	MAPKAPK5	99
MARK1	MARK1	99
MARK2	MARK2	94
MARK3	MARK3	100
MARK4	MARK4	100
MAST1	MAST1	79
MEK1	MAP2K1	97
MEK2	MAP2K2	89
MEK3	MAP2K3	98
MEK4	MAP2K4	100
MEK5	MAP2K5	95
MEK6	MAP2K6	99
MELK	MELK	100
MERTK	MERTK	100
MET	MET	95
MET(M1250T)	MET	88
MET(Y1235D)	MET	100
MINK	MINK1	95
MKK7	MAP2K7	100

MKNK1	MKNK1	100
MKNK2	MKNK2	100
MLCK	MYLK3	89
MLK1	MAP3K9	100
MLK2	MAP3K10	100
MLK3	MAP3K11	100
MRCKA	CDC42BPA	100
MRCKB	CDC42BPB	94
MST1	STK4	100
MST1R	MST1R	100
MST2	STK3	100
MST3	STK24	100
MST4	MST4	97
MTOR	MTOR	55
MUSK	MUSK	100
MYLK	MYLK	92
MYLK2	MYLK2	100
MYLK4	MYLK4	94
MYO3A	MYO3A	100
MYO3B	MYO3B	84
NDR1	STK38	89
NDR2	STK38L	95
NEK1	NEK1	100
NEK10	NEK10	100
NEK11	NEK11	100
NEK2	NEK2	100
NEK3	NEK3	83
NEK4	NEK4	99
NEK5	NEK5	100
NEK6	NEK6	100
NEK7	NEK7	85
NEK9	NEK9	100
NIK	MAP3K14	86
NIM1	MGC42105	100
NLK	NLK	87
OSR1	OXSR1	100
p38-alpha	MAPK14	100
p38-beta	MAPK11	100
p38-delta	MAPK13	100

p38-gamma	MAPK12	100
PAK1	PAK1	100
PAK2	PAK2	100
PAK3	PAK3	100
PAK4	PAK4	100
PAK6	PAK6	100
PAK7	PAK7	92
PCTK1	CDK16	94
PCTK2	CDK17	98
PCTK3	CDK18	100
PDGFRA	PDGFRA	100
PDGFRB	PDGFRB	100
PDPK1	PDPK1	92
PFCDPK1(P.falciparum)	CDPK1	100
PFPK5(P.falciparum)	MAL13P1.279	100
PFTAIRE2	CDK15	95
PFTK1	CDK14	100
PHKG1	PHKG1	100
PHKG2	PHKG2	95
PIK3C2B	PIK3C2B	100
PIK3C2G	PIK3C2G	97
PIK3CA	PIK3CA	95
PIK3CA(C420R)	PIK3CA	100
PIK3CA(E542K)	PIK3CA	80
PIK3CA(E545A)	PIK3CA	100
PIK3CA(E545K)	PIK3CA	66
PIK3CA(H1047L)	PIK3CA	100
PIK3CA(H1047Y)	PIK3CA	96
PIK3CA(I800L)	PIK3CA	77
PIK3CA(M1043I)	PIK3CA	100
PIK3CA(Q546K)	PIK3CA	96
PIK3CB	PIK3CB	100
PIK3CD	PIK3CD	100
PIK3CG	PIK3CG	62
PIK4CB	PI4KB	94
PIKFYVE	PIKFYVE	61
PIM1	PIM1	100
PIM2	PIM2	100
PIM3	PIM3	100

PIP5K1A	PIP5K1A	100
PIP5K1C	PIP5K1C	100
PIP5K2B	PIP4K2B	100
PIP5K2C	PIP4K2C	28
PKAC-alpha	PRKACA	100
PKAC-beta	PRKACB	100
PKMYT1	PKMYT1	62
PKN1	PKN1	100
PKN2	PKN2	100
PKNB(M.tuberculosis)	pknB	97
PLK1	PLK1	100
PLK2	PLK2	100
PLK3	PLK3	98
PLK4	PLK4	99
PRKCD	PRKCD	100
PRKCE	PRKCE	100
PRKCH	PRKCH	100
PRKCI	PRKCI	96
PRKCQ	PRKCQ	100
PRKD1	PRKD1	100
PRKD2	PRKD2	100
PRKD3	PRKD3	100
PRKG1	PRKG1	100
PRKG2	PRKG2	100
PRKR	EIF2AK2	97
PRKX	PRKX	97
PRP4	PRPF4B	100
PYK2	PTK2B	100
QSK	KIAA0999	96
RAF1	RAF1	100
RET	RET	92
RET(M918T)	RET	100
RET(V804L)	RET	100
RET(V804M)	RET	96
RIOK1	RIOK1	100
RIOK2	RIOK2	100
RIOK3	RIOK3	100
RIPK1	RIPK1	100
RIPK2	RIPK2	98

RIPK4	RIPK4	82
RIPK5	DSTYK	100
ROCK1	ROCK1	100
ROCK2	ROCK2	100
ROS1	ROS1	100
RPS6KA4(Kin.Dom.1-N-terminal)	RPS6KA4	100
RPS6KA4(Kin.Dom.2-C-terminal)	RPS6KA4	100
RPS6KA5(Kin.Dom.1-N-terminal)	RPS6KA5	100
RPS6KA5(Kin.Dom.2-C-terminal)	RPS6KA5	100
RSK1(Kin.Dom.1-N-terminal)	RPS6KA1	98
RSK1(Kin.Dom.2-C-terminal)	RPS6KA1	100
RSK2(Kin.Dom.1-N-terminal)	RPS6KA3	100
RSK2(Kin.Dom.2-C-terminal)	RPS6KA3	100
RSK3(Kin.Dom.1-N-terminal)	RPS6KA2	100
RSK3(Kin.Dom.2-C-terminal)	RPS6KA2	100
RSK4(Kin.Dom.1-N-terminal)	RPS6KA6	96
RSK4(Kin.Dom.2-C-terminal)	RPS6KA6	100
S6K1	RPS6KB1	94
SBK1	SBK1	100
SGK	SGK1	100
SgK110	SgK110	85
SGK2	SGK2	100
SGK3	SGK3	100
SIK	SIK1	95
SIK2	SIK2	100
SLK	SLK	100
SNARK	NUAK2	100
SNRK	SNRK	100
SRC	SRC	100
SRMS	SRMS	98
SRPK1	SRPK1	100
SRPK2	SRPK2	87
SRPK3	SRPK3	100
STK16	STK16	41
STK33	STK33	100
STK35	STK35	100
STK36	STK36	100
STK39	STK39	97
SYK	SYK	97

TAK1	MAP3K7	100
TAOK1	TAOK1	100
TAOK2	TAOK2	98
TAOK3	TAOK3	100
TBK1	TBK1	91
TEC	TEC	100
TESK1	TESK1	95
TGFBR1	TGFBR1	100
TGFBR2	TGFBR2	100
TIE1	TIE1	100
TIE2	TEK	100
TLK1	TLK1	100
TLK2	TLK2	100
TNIK	TNIK	100
TNK1	TNK1	100
TNK2	TNK2	100
TNNI3K	TNNI3K	92
TRKA	NTRK1	8.9
TRKB	NTRK2	86
TRKC	NTRK3	59
TRPM6	TRPM6	91
TSSK1B	TSSK1B	100
TSSK3	TSSK3	96
TTK	TTK	86
TXK	TXK	96
TYK2(JH1domain-catalytic)	TYK2	99
TYK2(JH2domain-pseudokinase)	TYK2	100
TYRO3	TYRO3	100
ULK1	ULK1	100
ULK2	ULK2	100
ULK3	ULK3	100
VEGFR2	KDR	99
VPS34	PIK3C3	100
VRK2	VRK2	100
WEE1	WEE1	100
WEE2	WEE2	100
WNK1	WNK1	100
WNK2	WNK2	67
WNK3	WNK3	100

WNK4	WNK4	100
YANK1	STK32A	100
YANK2	STK32B	100
YANK3	STK32C	100
YES	YES1	100
YSK1	STK25	98
YSK4	MAP3K19	34
ZAK	ZAK	97
ZAP70	ZAP70	100

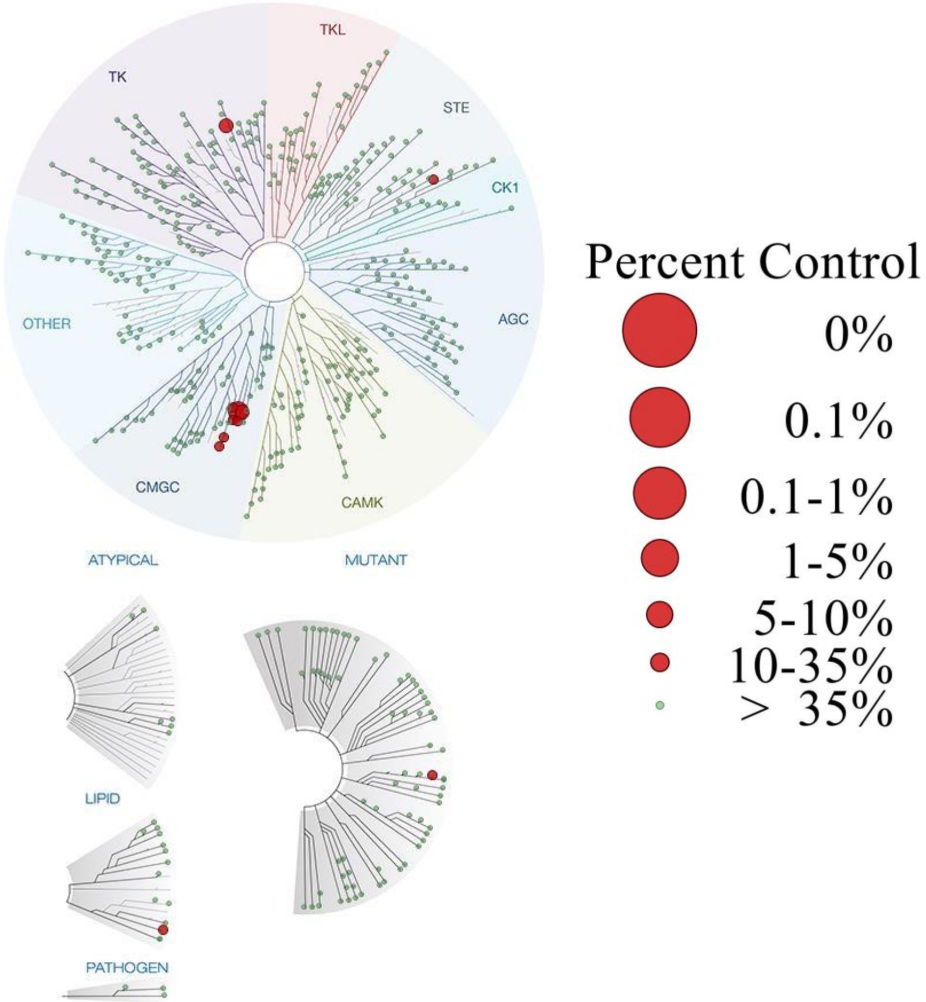

- TREE*spot*™ Interaction Maps for ***DB18***
