# Supplementary material for: Structure Activity Relationship Studies around DB18, a Potent and Selective Inhibitor of CLK Kinases
Source: Molecules. 2022 Sep 20;27(19):6149. doi: 10.3390/molecules27196149 (PMC9571063; doi:10.3390/molecules27196149)

Structure Activity Relationship Studies around DB18, a Potent and Selective Inhibitor of CLK Kinases

Dabbugoddu Brahmaiah [1,2], Anagani Kanaka Durga Bhavani [3,*], Pasula Aparna [2], Nangunoori Sampath Kumar [1], Hélène Solhi [4], Rémy Le Guevel [4], Blandine Baratte [5,6], Thomas Robert [5,6], Sandrine Ruchaud [6], Stéphane Bach [5,6,7], Surender Singh Jadav [8], Chada Raji Reddy [8], Paul Mosset [9], Nicolas Gouault [9], Nicolas Levoin [10,*] and René Grée [9,*]

Views of NMR spectra

_N_-Phenyl-8-((1-(4-methyl-2-nitrophenyl)-1_H_-1,2,3-triazol-4-yl)methoxy)quinazolin-2-amine (7a) : Pages S3-S13

_N_-(3,5-dichlorophenyl)-8-((1-(4-methyl-2-nitrophenyl)-1_H_-1,2,3-triazol-4-yl)methoxy)quinazolin-2-amine (7b) : Pages S14-S20

Ethyl 4-amino-3-nitrobenzoate (9): Pages S21-S24

4-Amino-3-nitrobenzyl alcohol (10e): Pages S25-S31

4-Azido-3-nitrophenol (11c): Pages S32-S36

1-Azido-4-bromo-2-nitrobenzene (11d): Pages S37-S41

4-Azido-3-nitrobenzyl alcohol (11e): Pages S42-S48

_N_-(3-Chlorophenyl)-8-((1-(4-hydroxy-2-nitrophenyl)-1_H_-1,2,3-triazol-4-yl)methoxy)quinazolin-2-amine (12c) : Pages S49-S63

_N_-(3-Chlorophenyl)-8-((1-(4-bromo-2-nitrophenyl)-1_H_-1,2,3-triazol-4-yl)methoxy)quinazolin-2-amine (12d) : Pages S64-S75

4-(4-(((2-((3-Chlorophenyl)amino)quinazolin-8-yl)oxy)methyl)-1_H_-1,2,3-triazol-1-yl)-3-nitrobenzyl alcohol (12e) : Pages S76-S89

Methyl 4-(4-(((2-((3-chlorophenyl)amino)quinazolin-8-yl)oxy)methyl)-1_H_-1,2,3-triazol-1-yl)-3-nitrobenzoate (12f) : Pages S90-S101

4-(4-(((2-((3-Chlorophenyl)amino)quinazolin-8-yl)oxy)methyl)-1_H_-1,2,3-triazol-1-yl)-3-nitrobenzoic acid (12g): Pages S102-S112

tert-Butyl ((_cis_)-4-(4-(((2-((3-chlorophenyl)amino)quinazolin-8-yl)oxy)methyl)-1_H_-1,2,3-triazol-1-yl)cyclohexyl)carbamate (15a): Pages S113-S123

tert-Butyl ((_cis_)-4-(4-(((2-((3,5-dichlorophenyl)amino)quinazolin-8-yl)oxy)methyl)-1_H_-1,2,3-triazol-1-yl)cyclohexyl)carbamate (15b): Pages S124-S133

tert-Butyl ((*trans*)-4-(4-(((2-((3-chlorophenyl)amino) quinazolin-8-yl) oxy) methyl)-1*H*-1,2,3-triazol-1-yl)cyclohexyl)carbamate (18a): Pages S134-S147

tert-Butyl ((*trans*)-4-(4-(((2-((3,5-dichlorophenyl)amino)quinazolin-8-yl)oxy) methyl)-1*H*-1,2,3-triazol-1-yl)cyclohexyl)carbamate (18b): Pages S148-S159

8-((1-((*cis*)-4-Aminocyclohexyl)-1*H*-1,2,3-triazol-4-yl)methoxy)-*N*-(3-chlorophenyl)quinazolin-2-amine hydrochloride (16a): Pages S160-S165

8-((1-((*cis*)-4-Aminocyclohexyl)-1*H*-1,2,3-triazol-4-yl)methoxy)-*N*-(3,5-dichlorophenyl)quinazolin-2-amine hydrochloride (16b): Pages S166-S171

8-((1-((*trans*)-4-Aminocyclohexyl)-1*H*-1,2,3-triazol-4-yl)methoxy)-*N*-(3-chlorophenyl)quinazolin-2-amine hydrochloride (19a): Pages S172-S177

8-((1-((*trans*)-4-Aminocyclohexyl)-1*H*-1,2,3-triazol-4-yl)methoxy)-*N*-(3,5-dichlorophenyl)quinazolin-2-amine hydrochloride (19b): Pages S178-S183

High resolution mass spectra

N-(3,5-dichlorophenyl)-8-((1-(4-methyl-2-nitrophenyl)-1*H*-1,2,3-triazol-4-yl)methoxy)quinazolin-2-amine (7b): (**DBPh**) Page 184

N-(3-Chlorophenyl)-8-((1-(4-hydroxy-2-nitrophenyl)-1*H*-1,2,3-triazol-4-yl)methoxy)quinazolin-2-amine (12c): (**PM 8173**) Page 185

N-(3-Chlorophenyl)-8-((1-(4-bromo-2-nitrophenyl)-1*H*-1,2,3-triazol-4-yl)methoxy)quinazolin-2-amine (12d): (**DB33**) Page 186

4-(4-(((2-((3-Chlorophenyl)amino)quinazolin-8-yl)oxy)methyl)-1*H*-1,2,3-triazol-1-yl)-3-nitrobenzyl alcohol (12e): (**PM 9019**) Page 187

Methyl 4-(4-(((2-((3-chlorophenyl)amino)quinazolin-8-yl)oxy)methyl)-1*H*-1,2,3-triazol-1-yl)-3-nitrobenzoate (12f): (**DB34**) Page 188

4-(4-(((2-((3-Chlorophenyl)amino)quinazolin-8-yl)oxy)methyl)-1*H*-1,2,3-triazol-1-yl)-3-nitrobenzoic acid (12g): (**DB35**) Page 189

tert-Butyl ((*cis*)-4-(4-(((2-((3-chlorophenyl)amino)quinazolin-8-yl)oxy)methyl)-1*H*-1,2,3-triazol-1-yl)cyclohexyl)carbamate (15a): (**DB25**) Page 190

tert-Butyl ((*cis*)-4-(4-(((2-((3,5-dichlorophenyl)amino)quinazolin-8-yl)oxy)methyl)-1*H*-1,2,3-triazol-1-yl)cyclohexyl)carbamate (15b): (**DB26**) Page 191

tert-Butyl ((*trans*)-4-(4-(((2-((3-chlorophenyl)amino) quinazolin-8-yl) oxy) methyl)-1*H*-1,2,3-triazol-1-yl)cyclohexyl)carbamate (18a): (**DB27**) Page 192

tert-Butyl ((*trans*)-4-(4-(((2-((3,5-dichlorophenyl)amino)quinazolin-8-yl)oxy) methyl)-1*H*-1,2,3-triazol-1-yl)cyclohexyl)carbamate (18b): (**DB28**) Page 193

For each compound, 3 spectra are displayed on a page: the whole spectrum at the top, the expansion of the molecular peak in the middle end the calculated (theoretical) isotopic cluster at the bottom (nearly identical to the experimental expansion).

_N_-Phenyl-8-((1-(4-methyl-2-nitrophenyl)-1_H_-1,2,3-triazol-4-yl)methoxy)quinazolin-2-amine (7a)

Pages S3-S12

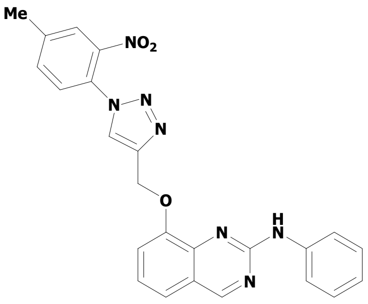

^{1}H - 300.13 MHz
DMSO-d$_6$, ref. TMS

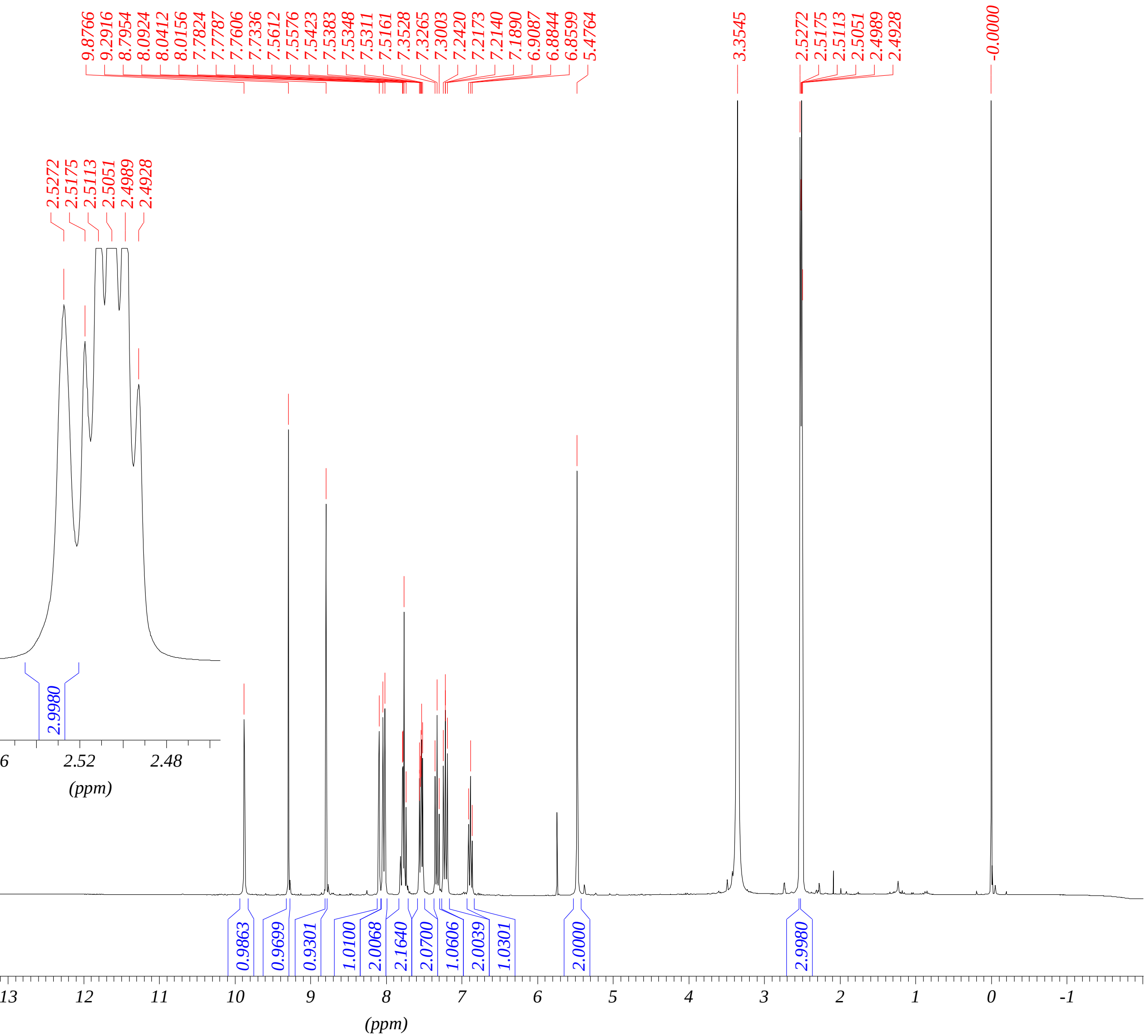

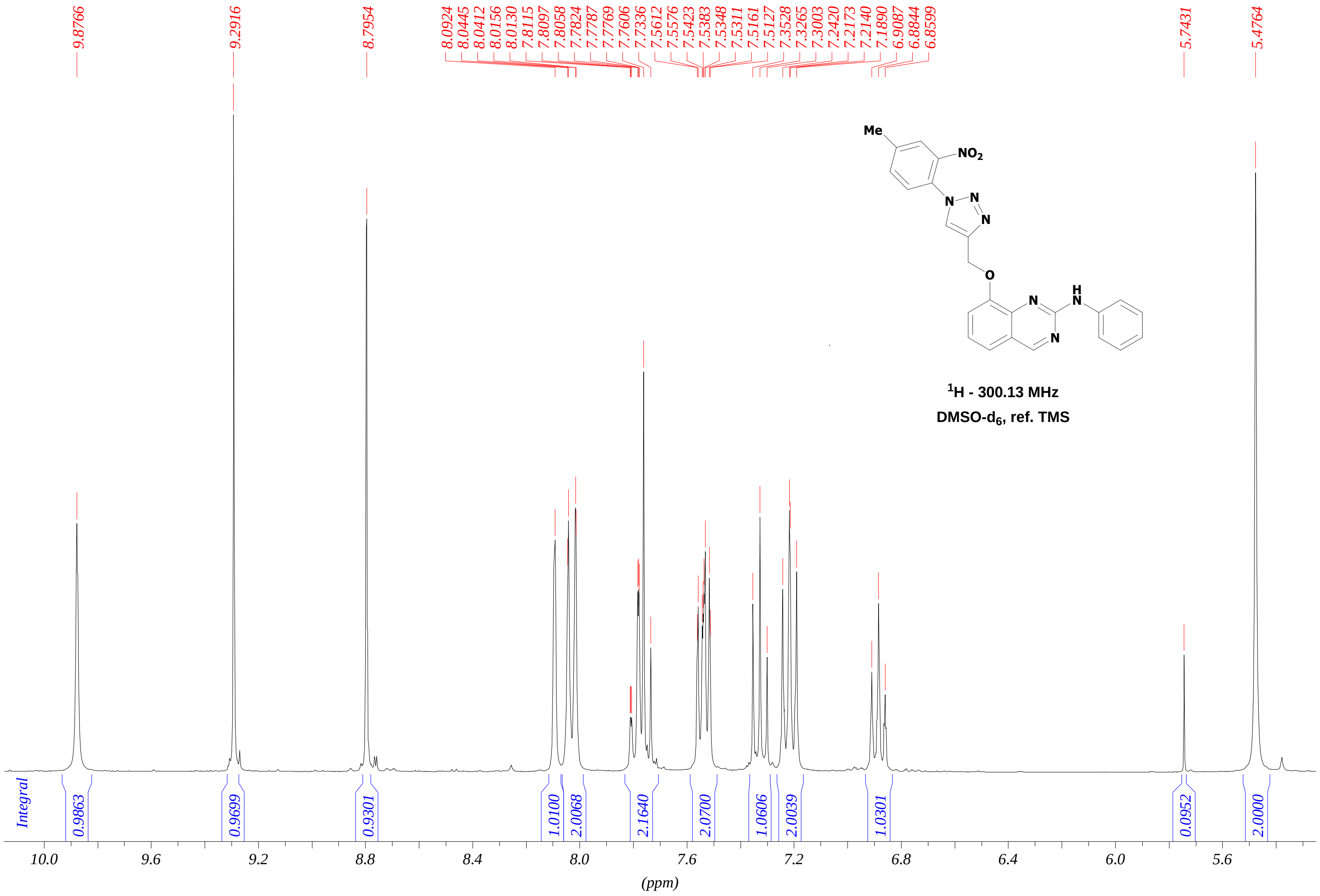
9.8766
9.2916
8.7954
8.0924 8.0445 8.0412 8.0156 8.0130 7.8115 7.8097 7.8058 7.7824 7.7787 7.7769 7.7606 7.7336 7.5612 7.5576 7.5423 7.5383 7.5348 7.5311 7.5161 7.5127 7.3528 7.3265 7.3003 7.2420 7.2173 7.2140 7.1890 6.9087 6.8844 6.8599
5.7431
5.4764
Me
NO2
N
N
N
O
N
H
N
N
1H - 300.13 MHz
DMSO-d6, ref. TMS
Integral
0.9863
0.9699
0.9301
1.0100
2.0068
2.1640
2.0700
1.0606
2.0039
1.0301
0.0952
2.0000
10.0
9.6
9.2
8.8
8.4
8.0
7.6
7.2
6.8
6.4
6.0
5.6
(ppm)

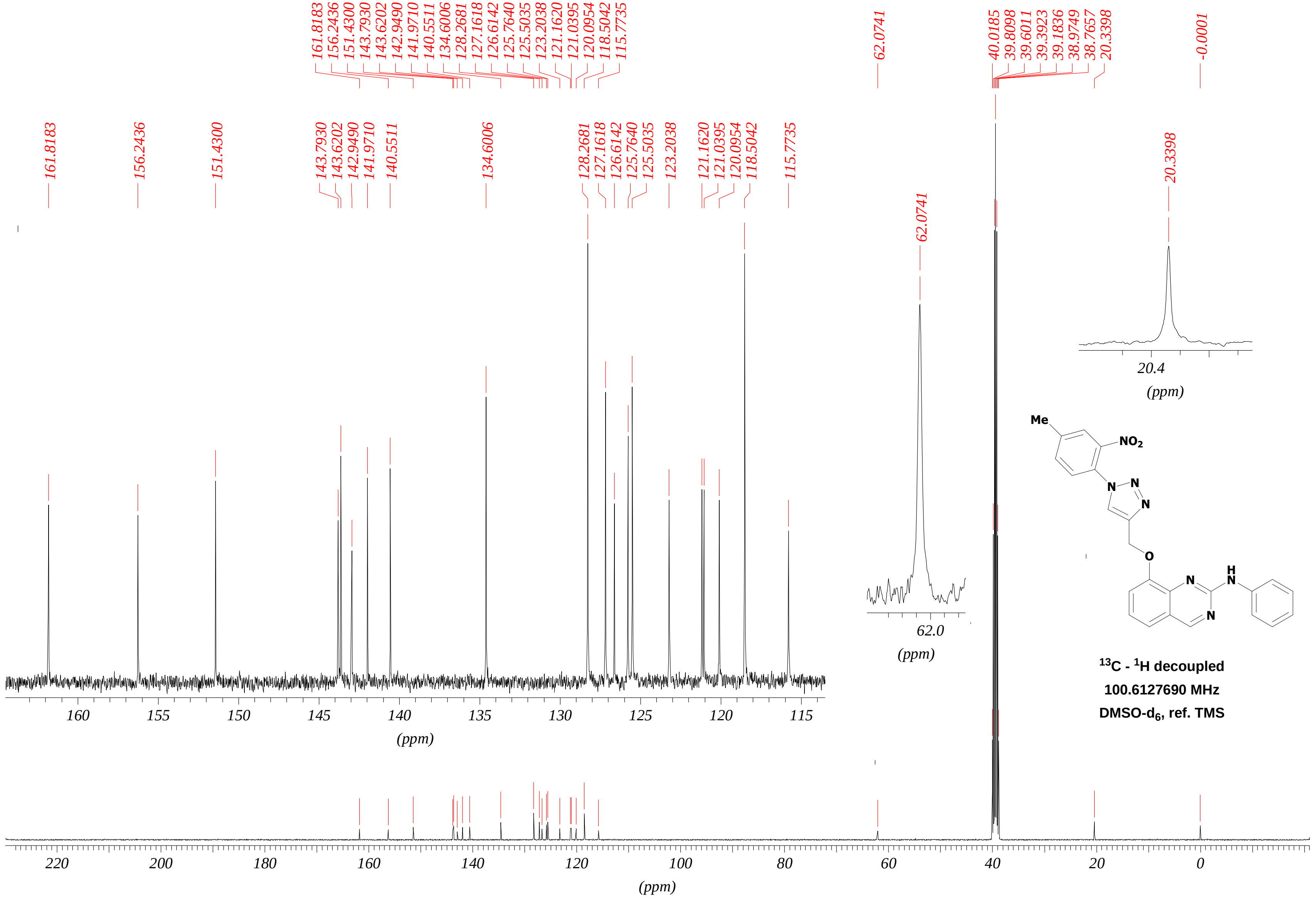
13C - 1H decoupled
100.6127690 MHz
DMSO-d6, ref. TMS
Me
NO2
N
N
N
O
N
N
H
N
(ppm)
62.0741
20.3398
-0.0001
40.0185
39.8098
39.6011
39.3923
39.1836
38.9749
38.7657
161.8183
156.2436
151.4300
143.7930
143.6202
142.9490
141.9710
140.5511
134.6006
128.2681
127.1618
126.6142
125.7640
125.5035
123.2038
121.1620
121.0395
120.0954
118.5042
115.7735

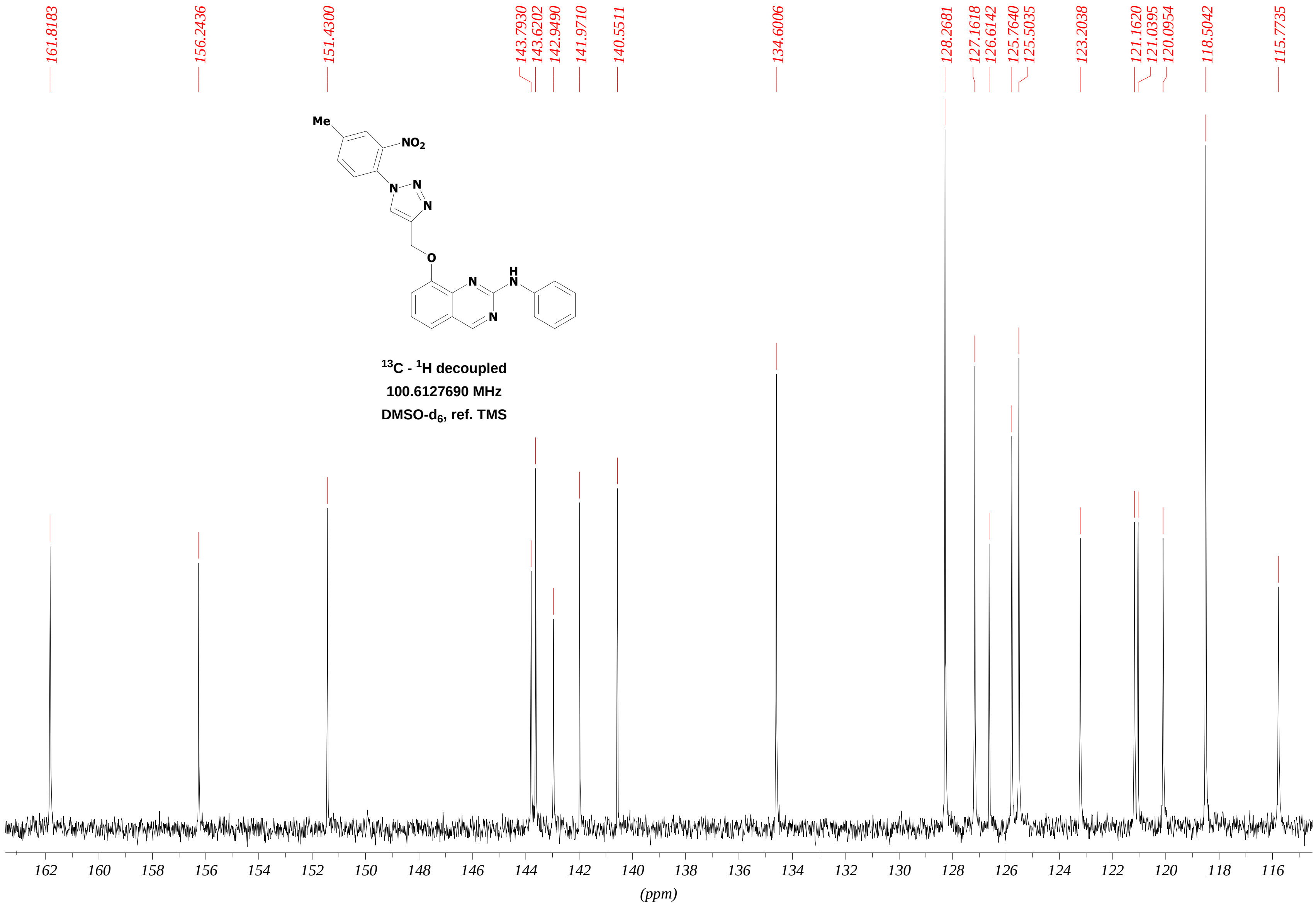
161.8183
156.2436
151.4300
143.7930
143.6202
142.9490
141.9710
140.5511
134.6006
128.2681
127.1618
126.6142
125.7640
125.5035
123.2038
121.1620
121.0395
120.0954
118.5042
115.7735
Me
NO2
N
N
N
O
N
N
H
N
13C - 1H decoupled
100.6127690 MHz
DMSO-d6, ref. TMS
162
160
158
156
154
152
150
148
146
144
142
140
138
136
134
132
130
128
126
124
122
120
118
116
(ppm)

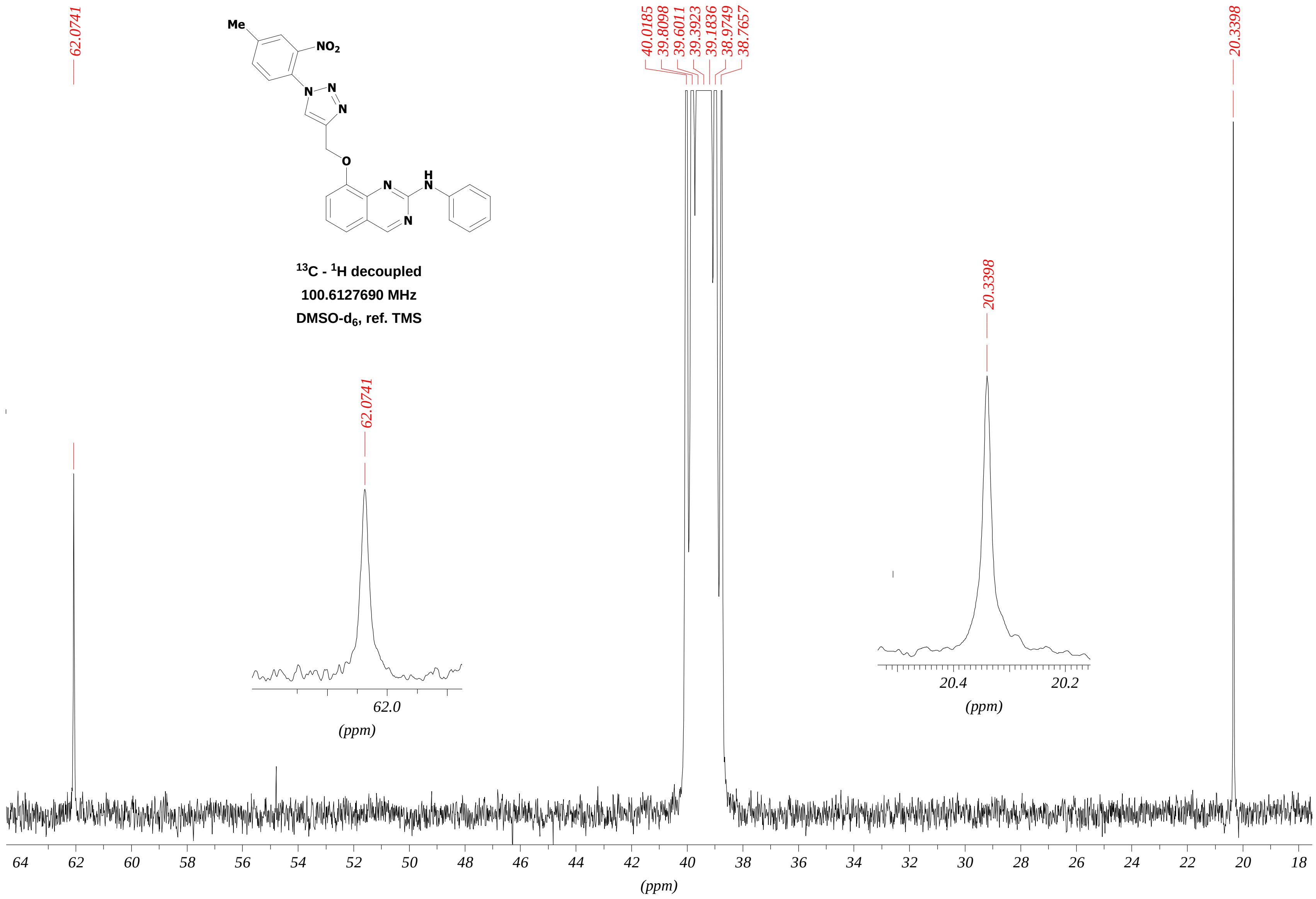
62.0741
Me
NO_2
N
N
N
O
N
H
N
N
^{13}C - ^{1}H decoupled
100.6127690 MHz
DMSO-d_6, ref. TMS
40.0185
39.8098
39.6011
39.3923
39.1836
38.9749
38.7657
20.3398
62.0
(ppm)
20.4
20.2
(ppm)
64
62
60
58
56
54
52
50
48
46
44
42
40
38
36
34
32
30
28
26
24
22
20
18
(ppm)

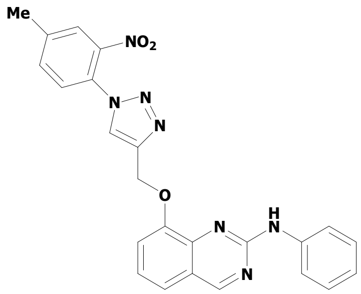

^{13}C - DEPT 135

100.6127690 MHz

DMSO-d_6, ref. TMS

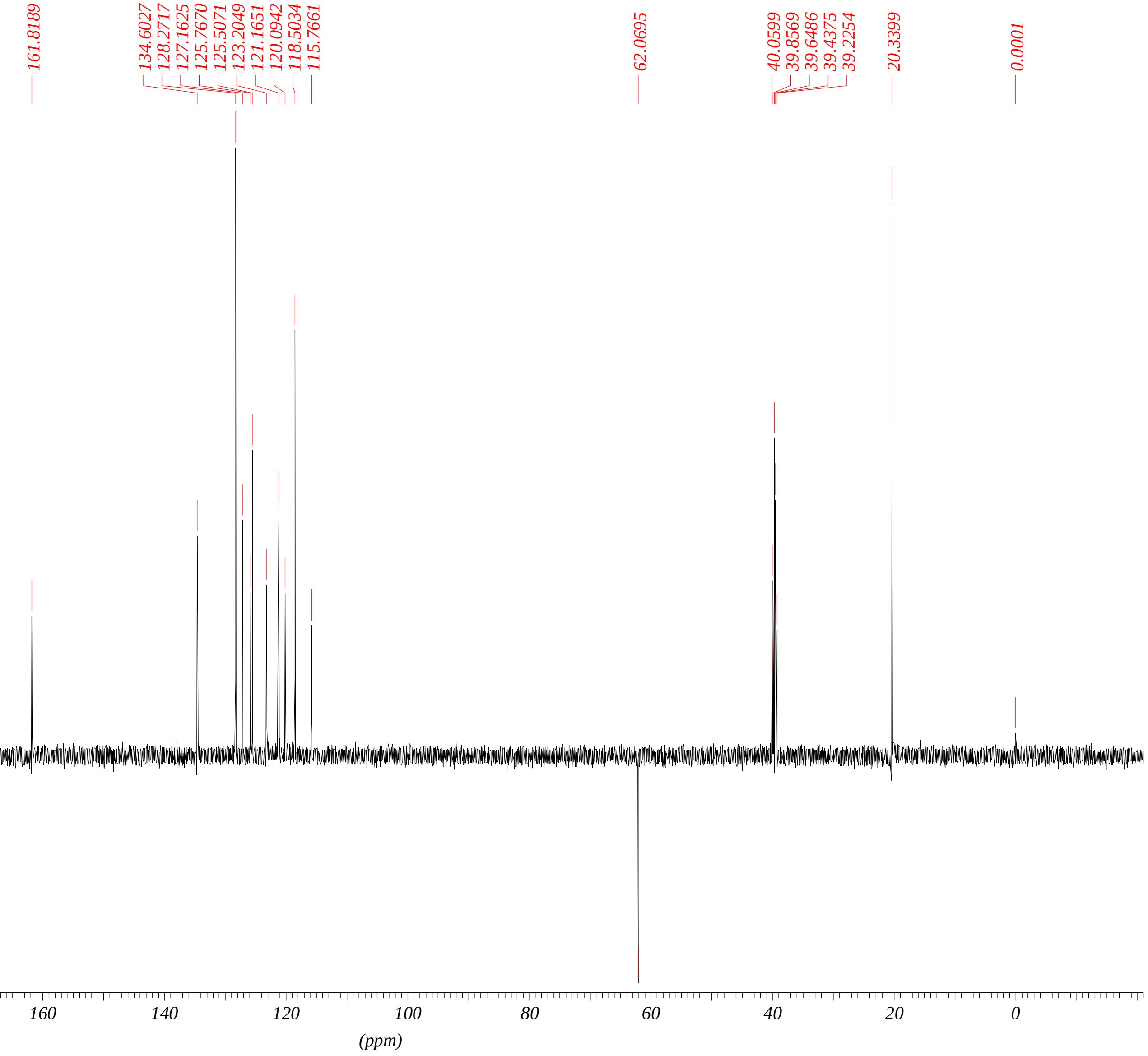

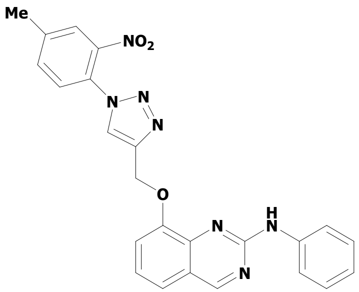

^{13}C - DEPT 135
100.6127690 MHz
DMSO-d_6, ref. TMS

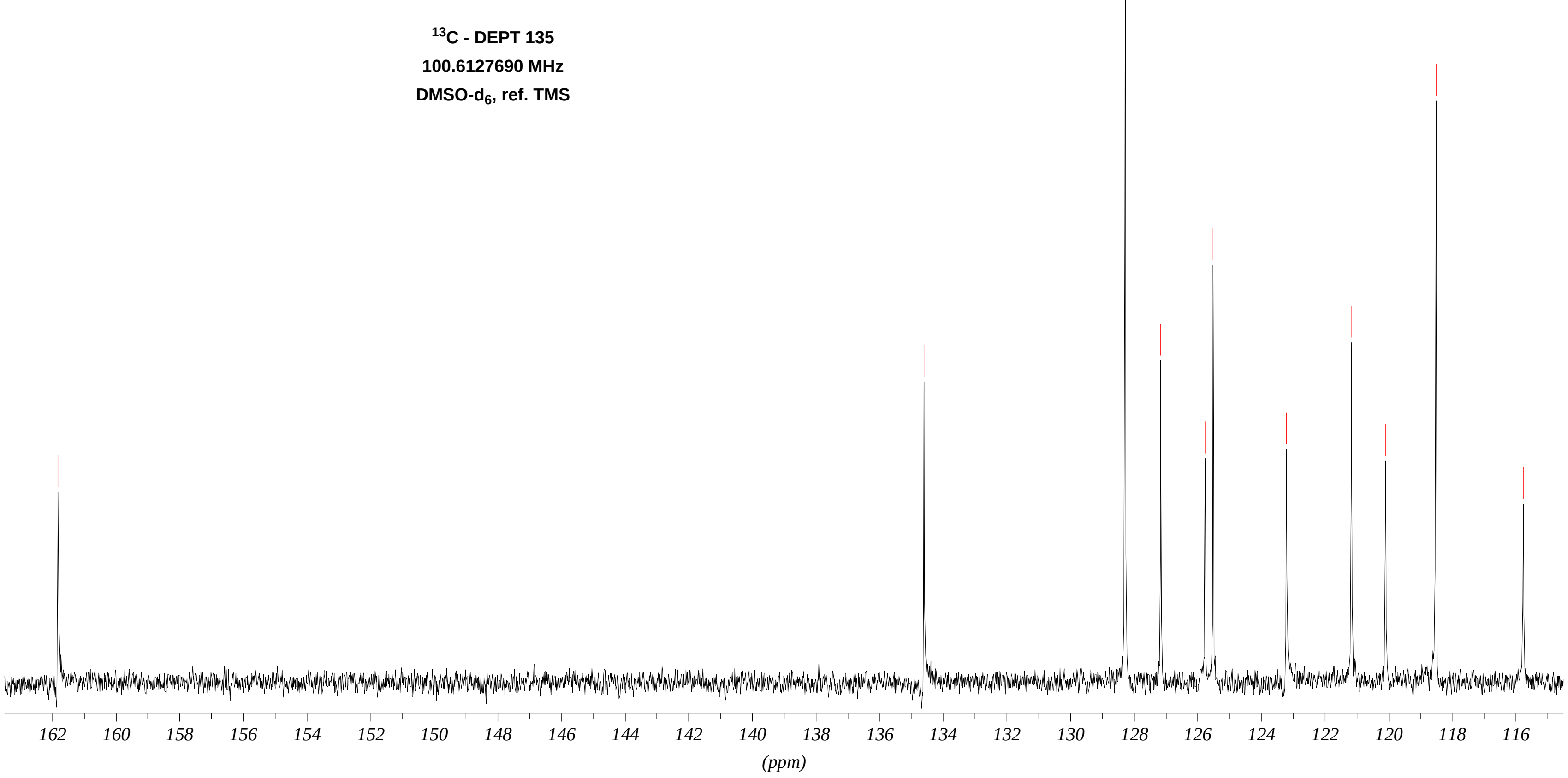

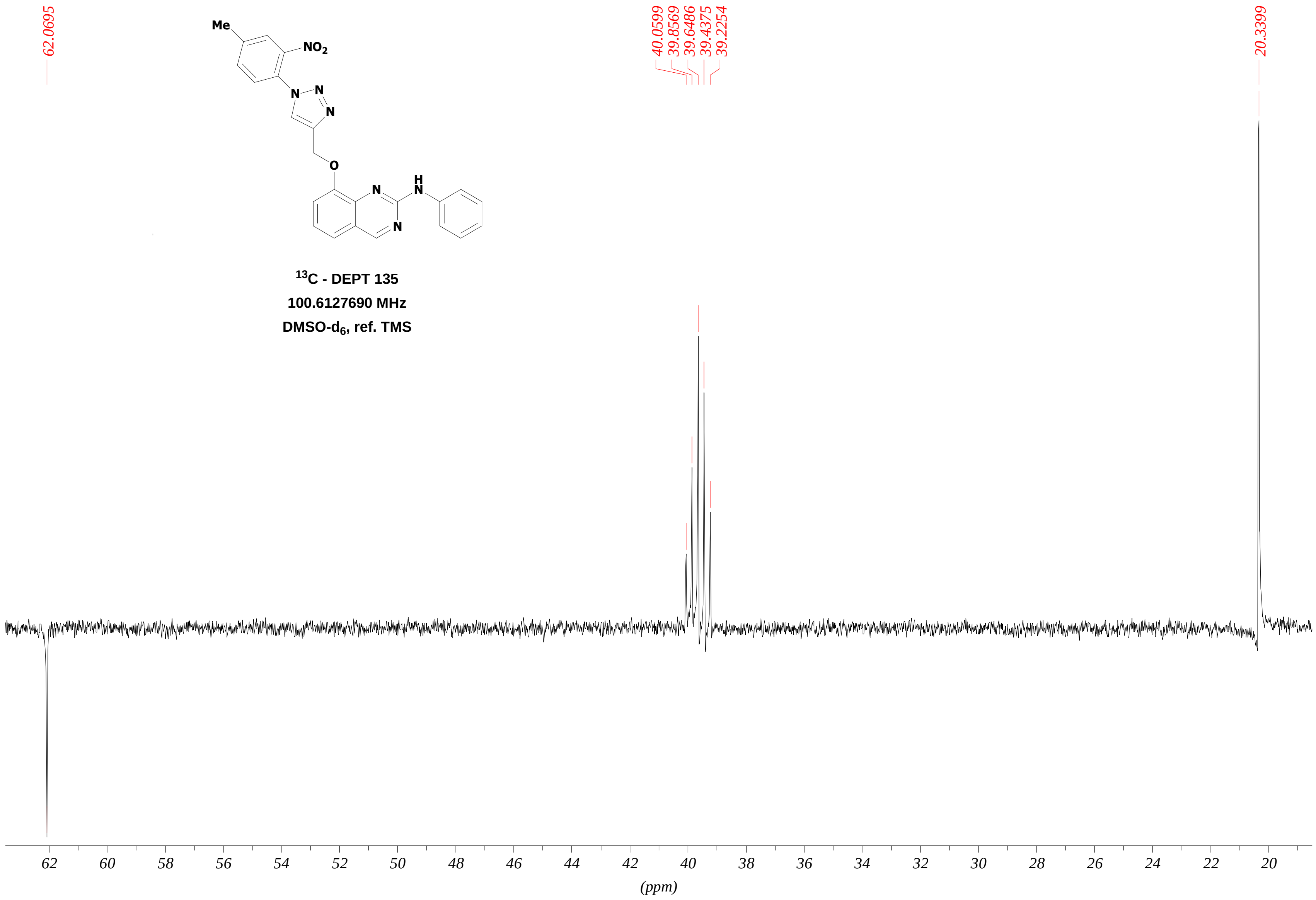

^{13}C - DEPT 135

100.6127690 MHz

DMSO-d_6, ref. TMS

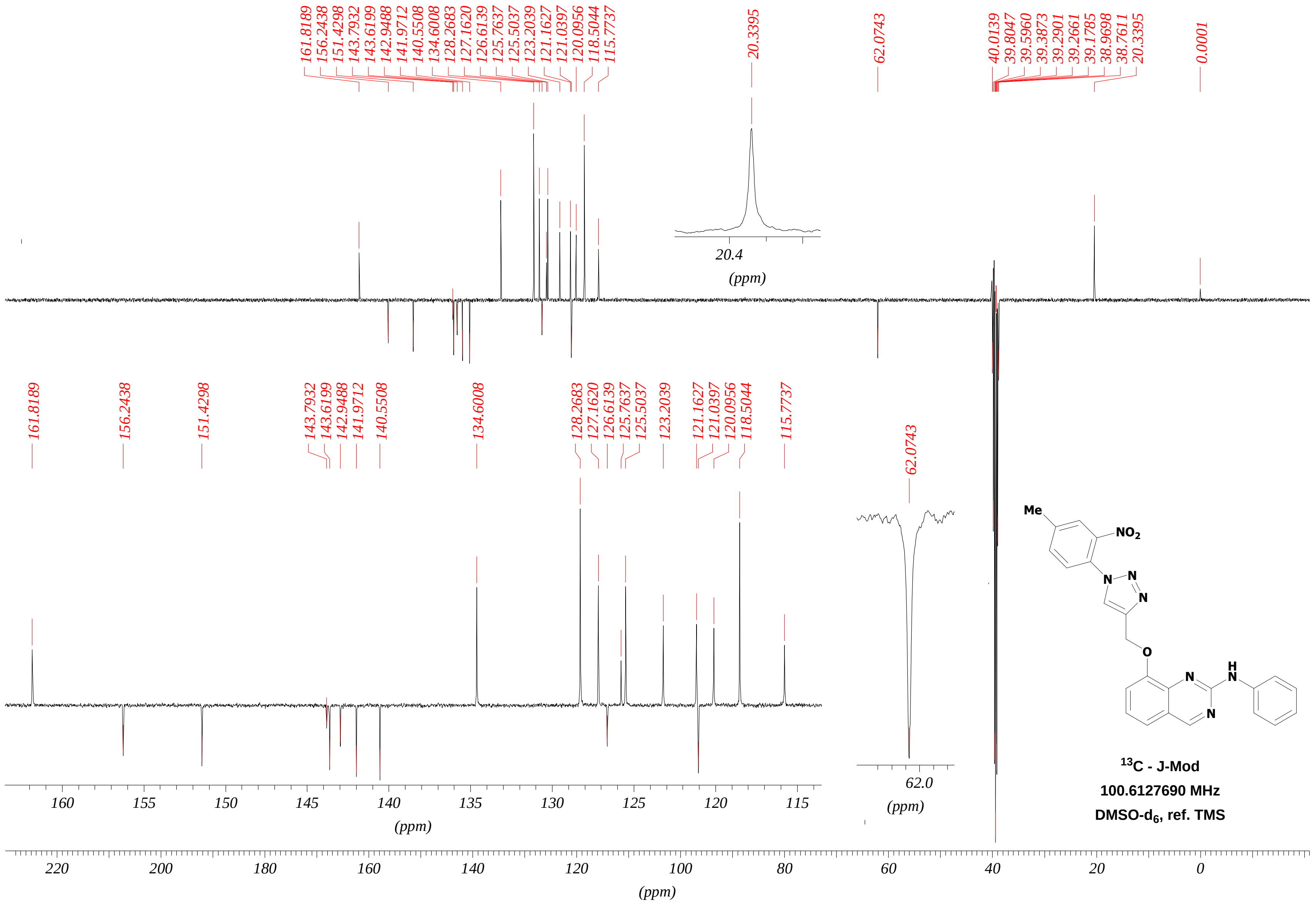

161.8189
156.2438
151.4298
143.7932
143.6199
142.9488
141.9712
140.5508
134.6008
128.2683
127.1620
126.6139
125.7637
125.5037
123.2039
121.1627
121.0397
120.0956
118.5044
115.7737
20.3395
62.0743
40.0139
39.8047
39.5960
39.3873
39.2901
39.2661
39.1785
38.9698
38.7611
20.3395
0.0001
20.4
(ppm)
62.0
(ppm)
160 155 150 145 140 135 130 125 120 115
(ppm)
220 200 180 160 140 120 100 80 60 40 20 0
(ppm)
Me
NO2
N
N
N
O
N
H
N
N
13C - J-Mod
100.6127690 MHz
DMSO-d6, ref. TMS

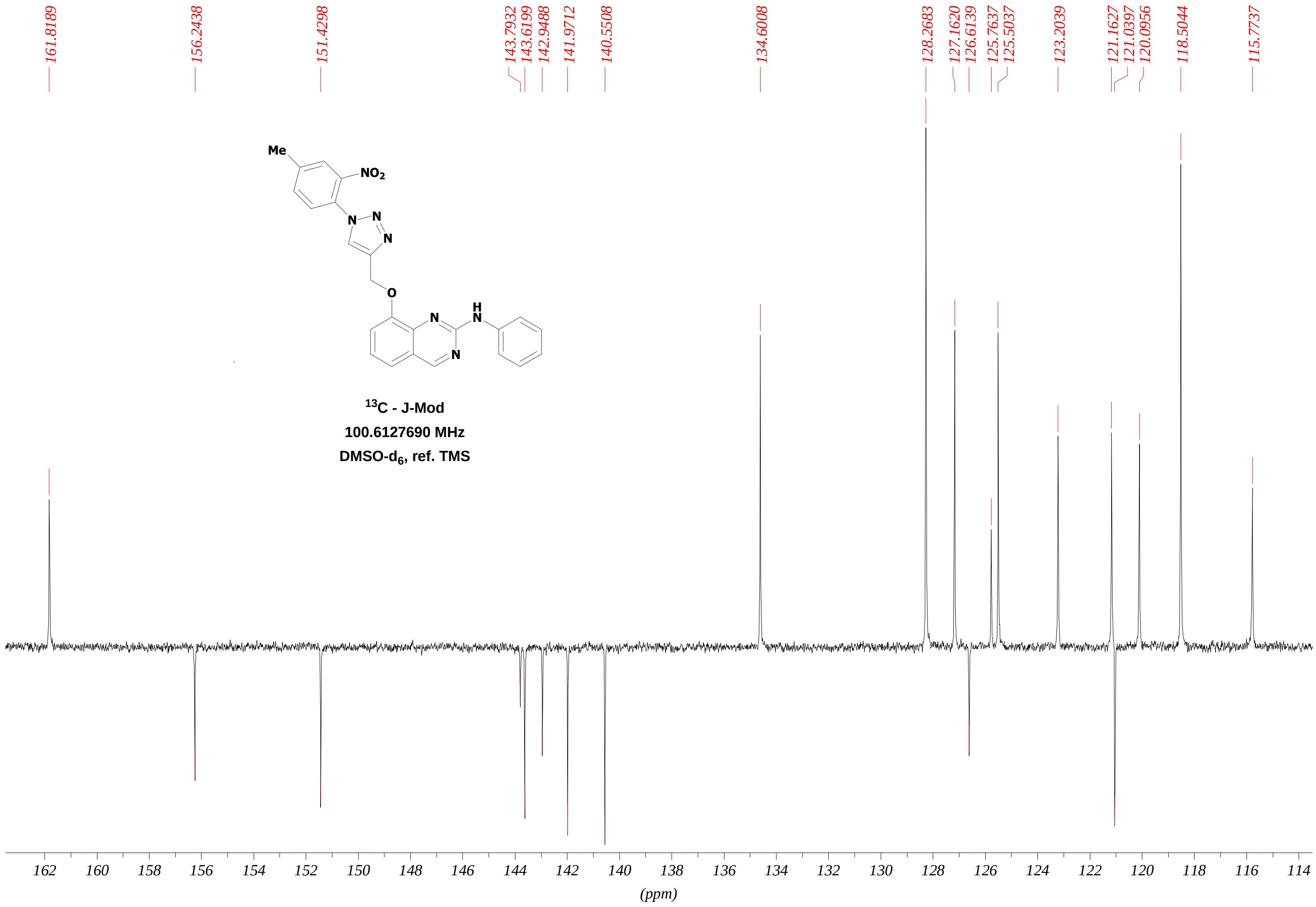
161.8189
156.2438
151.4298
143.7932
143.6199
142.9488
141.9712
140.5508
134.6008
128.2683
127.1620
126.6139
125.7637
125.5037
123.2039
121.1627
121.0397
120.0956
118.5044
115.7737
Me
NO_2
N
N
N
O
N
H
N
N
^{13}C - J-Mod
100.6127690 MHz
DMSO-d_6, ref. TMS
162 160 158 156 154 152 150 148 146 144 142 140 138 136 134 132 130 128 126 124 122 120 118 116 114
(ppm)

***N*-(3,5-dichlorophenyl)-8-((1-(4-methyl-2-nitrophenyl)-1*H*-1,2,3-triazol-4-yl)methoxy)quinazolin-2-amine (7b)**

Pages S14-S 20

A909-26D-GP1

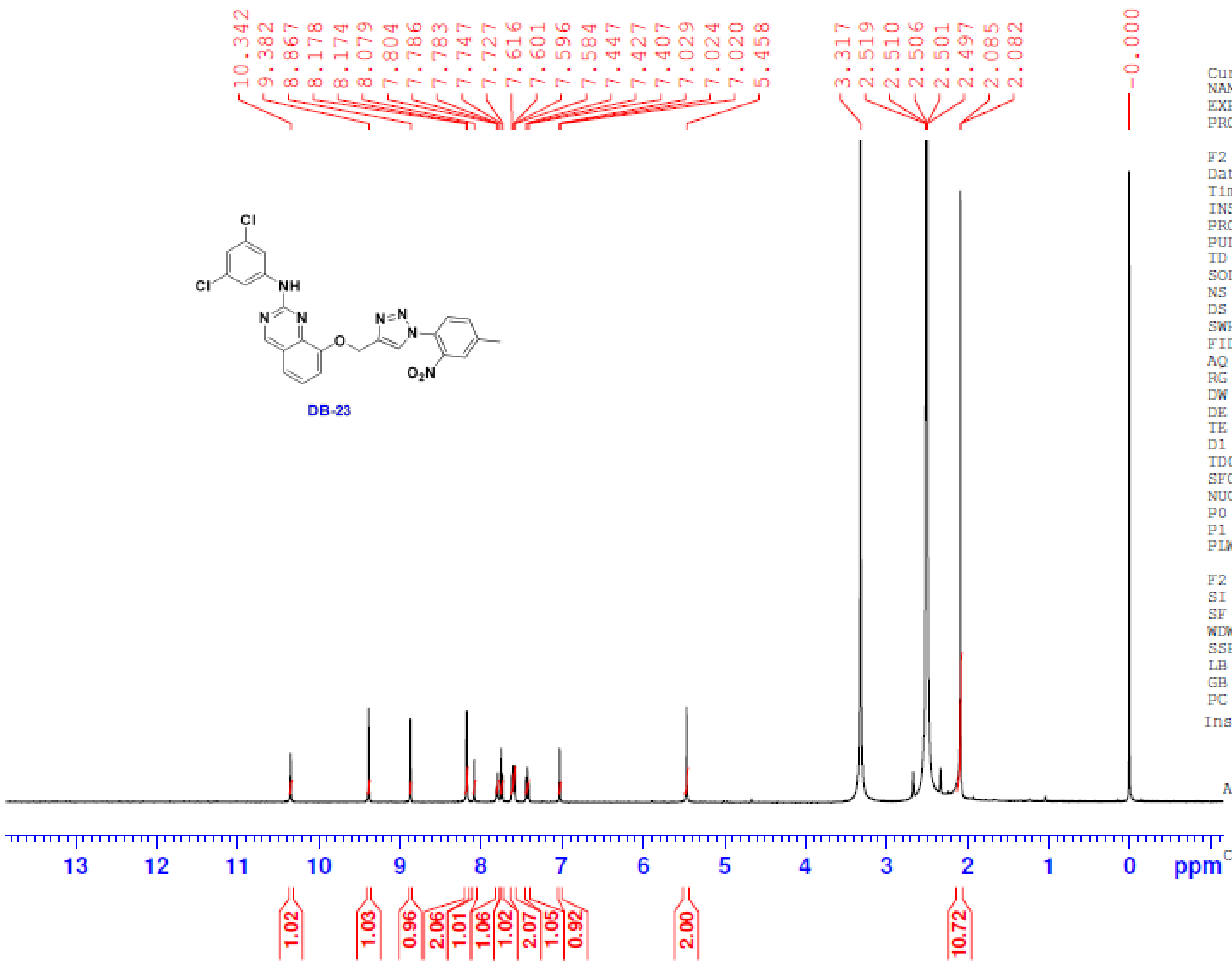

Current Data Parameters
NAME UNM200914339
EXPNO 1
PROCNO 1

F2 - Acquisition Parameters
Date_ 20200915
Time 3.56 h
INSTRUM spect
PROBHD Z150453_0004 (
PULPROG zg30
TD 65536
SOLVENT DMSO
NS 32
DS 2
SWH 8012.820 Hz
FIDRES 0.244532 Hz
AQ 4.0894465 sec
RG 204.77
DW 62.400 usec
DE 6.50 usec
TE 298.1 K
D1 1.00000000 sec
TD0 1
SFO1 400.1324708 MHz
NUC1 1H
P0 3.33 usec
P1 10.00 usec
PLW1 16.87899971 W

F2 - Processing parameters
SI 65536
SF 400.1300030 MHz
WDW EM
SSB 0
LB 0.30 Hz
GB 0
PC 1.00
Inst.ID: CVL1-AD-NMR-001

Analyzed By: Ranjith

Checked by:

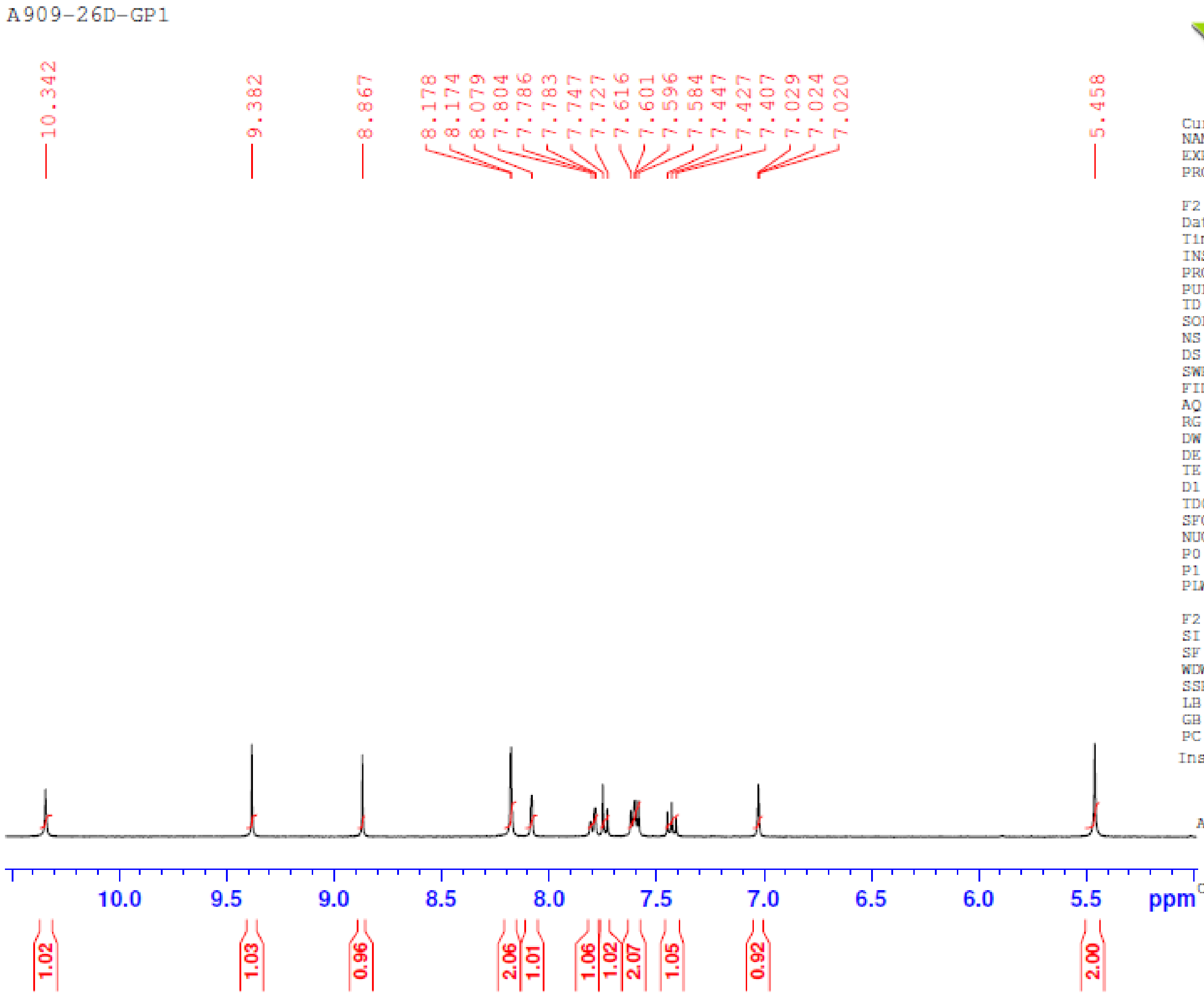

Current Data Parameters
NAME UNM200914339
EXPNO 1
PROCNO 1

F2 - Acquisition Parameters
Date_ 20200915
Time 3.56 h
INSTRUM spect
PROBHD Z150453_0004 (
PULPROG zg30
TD 65536
SOLVENT DMSO
NS 32
DS 2
SWH 8012.820 Hz
FIDRES 0.244532 Hz
AQ 4.0894465 sec
RG 204.77
DW 62.400 usec
DE 6.50 usec
TE 298.1 K
D1 1.00000000 sec
TD0 1
SFO1 400.1324708 MHz
NUC1 1H
P0 3.33 usec
P1 10.00 usec
PLW1 16.87899971 W

F2 - Processing parameters
SI 65536
SF 400.1300030 MHz
WDW EM
SSB 0
LB 0.30 Hz
GB 0
PC 1.00

Inst.ID: CVL1-AD-NMR-001

Analyzed By: Ranjith

Checked by:

A909-26D-GP1-D2OEXC

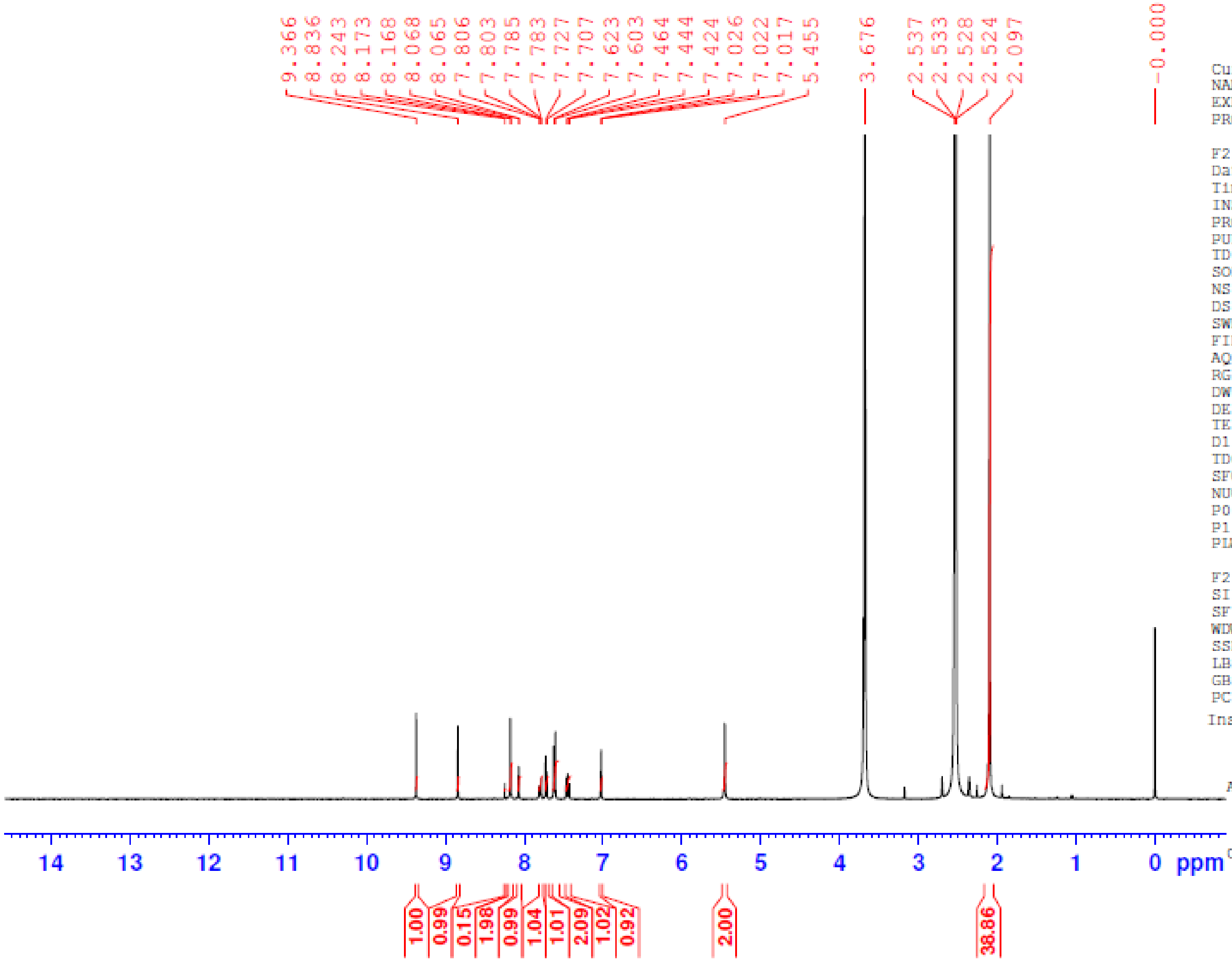

Current Data Parameters
NAME UNM200914339-D2OEXC
EXPNO 1
PROCNO 1

F2 - Acquisition Parameters
Date_ 20200915
Time 10.44 h
INSTRUM spect
PROBHD Z150453_0004 (
PULPROG zg30
TD 65536
SOLVENT DMSO
NS 32
DS 2
SWH 8012.820 Hz
FIDRES 0.244532 Hz
AQ 4.0894465 sec
RG 204.77
DW 62.400 usec
DE 6.50 usec
TE 298.1 K
D1 1.00000000 sec
TD0 1
SFO1 400.1324708 MHz
NUC1 1H
P0 3.33 usec
P1 10.00 usec
PLW1 16.87899971 W

F2 - Processing parameters
SI 65536
SF 400.1299920 MHz
WDW EM
SSB 0
LB 0.30 Hz
GB 0
PC 1.00

Inst. ID: CVL1-AD-NMR-001

Analyzed By: Ranjith

Checked by:

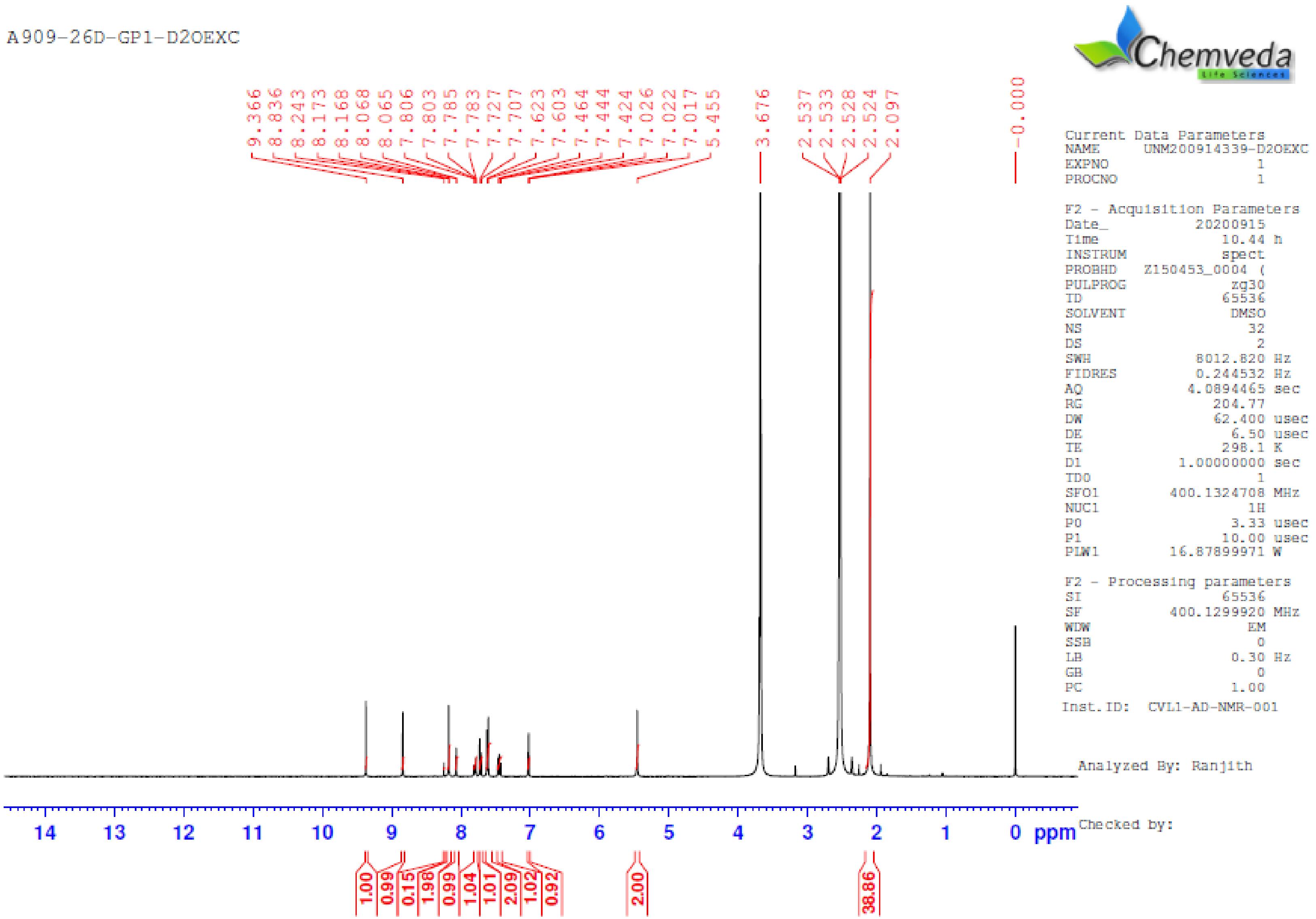
A909-26D-GP1-D2OEXC
9.366 8.836 8.243 8.173 8.168 8.068 8.065 7.806 7.803 7.785 7.783 7.727 7.707 7.623 7.603 7.464 7.444 7.424 7.026 7.022 7.017 5.455
3.676
2.537 2.533 2.528 2.524 2.097
-0.000
Current Data Parameters
NAME UNM200914339-D2OEXC
EXPNO 1
PROCNO 1
F2 - Acquisition Parameters
Date_ 20200915
Time 10.44 h
INSTRUM spect
PROBHD Z150453_0004 (
PULPROG zg30
TD 65536
SOLVENT DMSO
NS 32
DS 2
SWH 8012.820 Hz
FIDRES 0.244532 Hz
AQ 4.0894465 sec
RG 204.77
DW 62.400 usec
DE 6.50 usec
TE 298.1 K
D1 1.00000000 sec
TD0 1
SFO1 400.1324708 MHz
NUC1 1H
P0 3.33 usec
P1 10.00 usec
PLW1 16.87899971 W
F2 - Processing parameters
SI 65536
SF 400.1299920 MHz
WDW EM
SSB 0
LB 0.30 Hz
GB 0
PC 1.00
Inst. ID: CVL1-AD-NMR-001
Analyzed By: Ranjith
Checked by:
14 13 12 11 10 9 8 7 6 5 4 3 2 1 0 ppm
1.00 0.99 0.15 1.98 0.99 1.04 1.01 2.09 1.02 0.92 2.00 38.86

A909-26D-GP1-D2OEXC

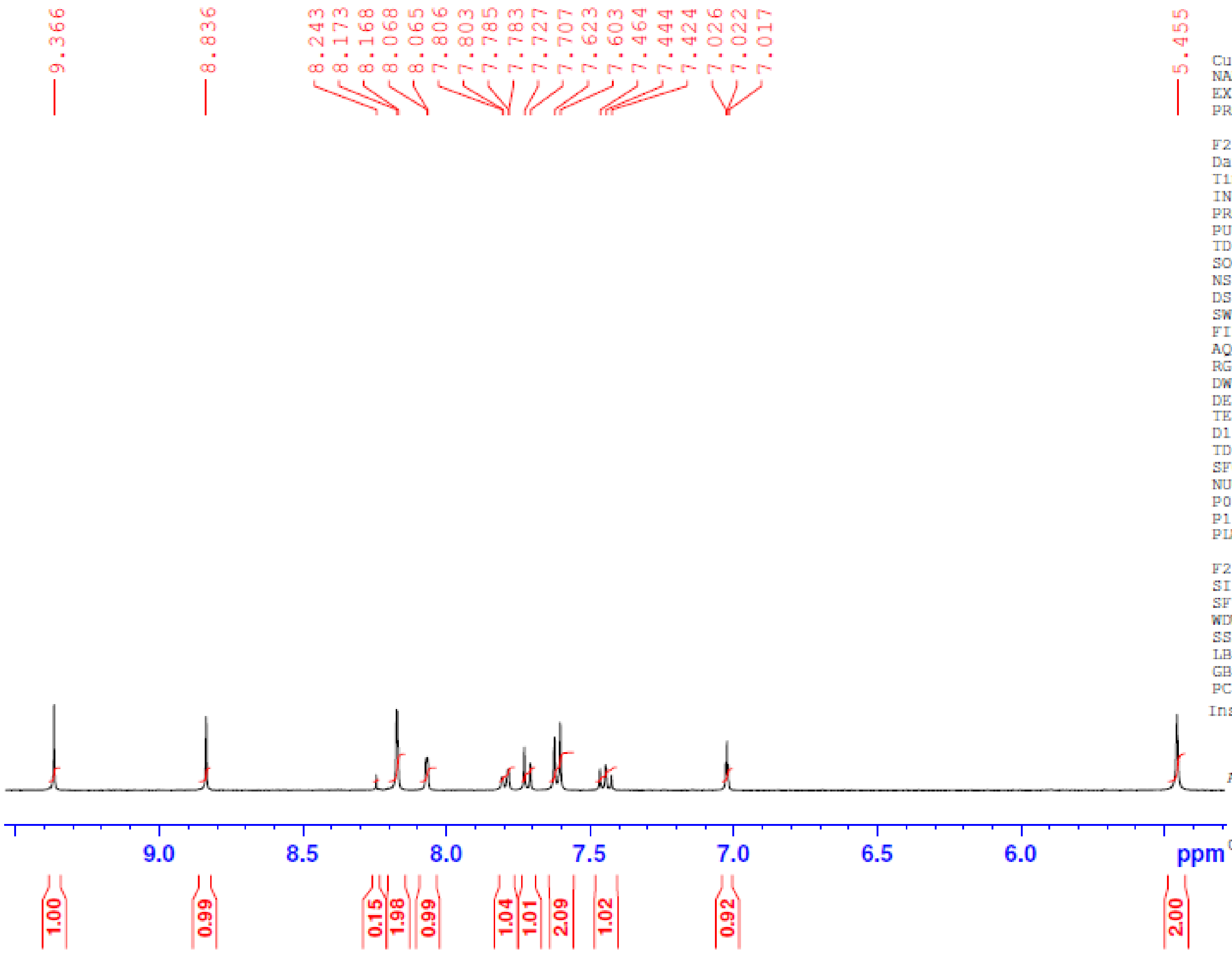

Current Data Parameters
NAME UNM200914339-D2OEXC
EXPNO 1
PROCNO 1

F2 - Acquisition Parameters
Date_ 20200915
Time 10.44 h
INSTRUM spect
PROBHD Z150453_0004 (
PULPROG zg30
TD 65536
SOLVENT DMSO
NS 32
DS 2
SWH 8012.820 Hz
FIDRES 0.244532 Hz
AQ 4.0894465 sec
RG 204.77
DW 62.400 usec
DE 6.50 usec
TE 298.1 K
D1 1.00000000 sec
TD0 1
SFO1 400.1324708 MHz
NUC1 1H
P0 3.33 usec
P1 10.00 usec
PLW1 16.87899971 W

F2 - Processing parameters
SI 65536
SF 400.1299920 MHz
WDW EM
SSB 0
LB 0.30 Hz
GB 0
PC 1.00

Inst.ID: CVL1-AD-NMR-001

Analyzed By: Ranjith

Checked by:

A909-26D-GP1-D2OEXC

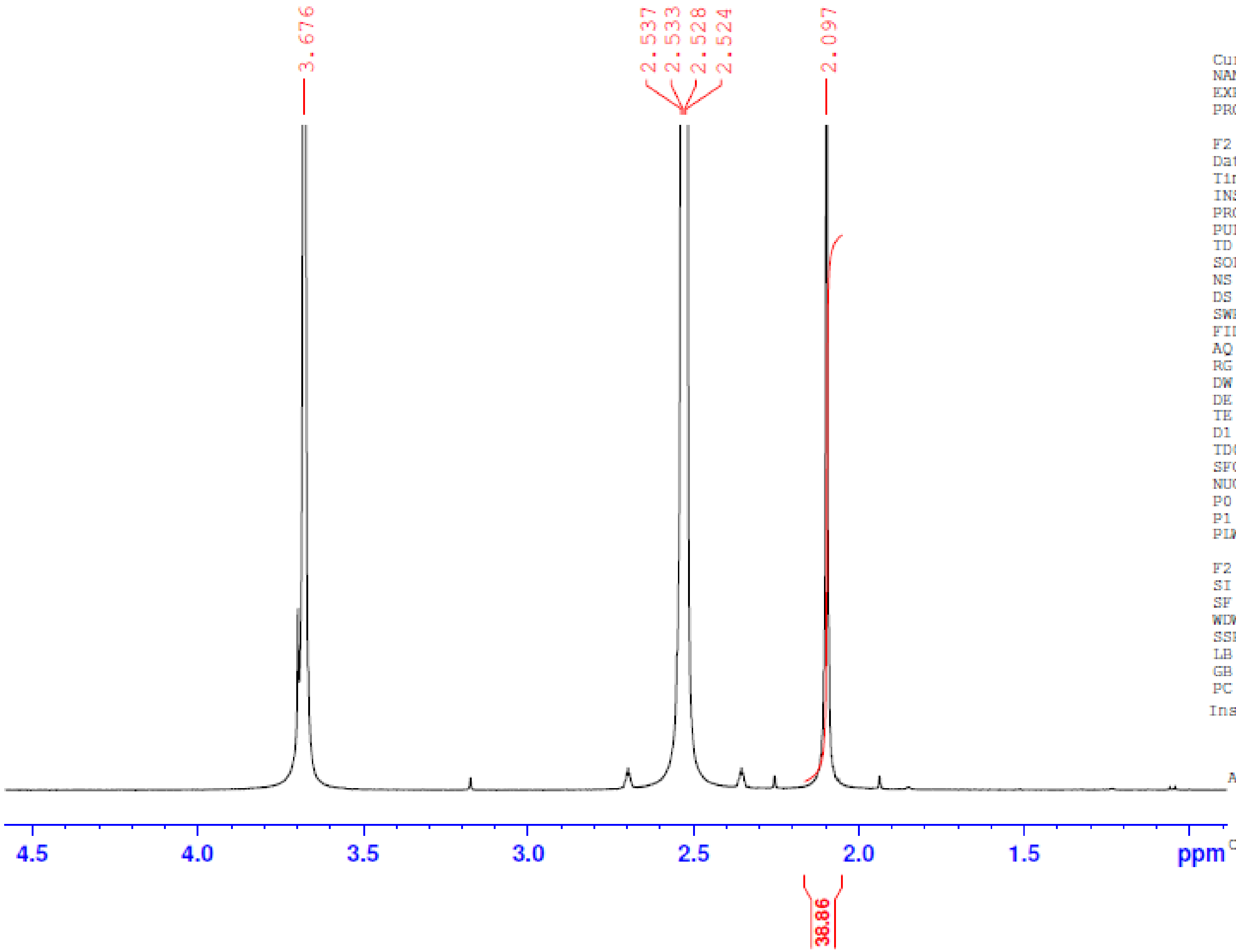

Chemveda Life Sciences

Current Data Parameters
NAME UNM200914339-D2OEXC
EXPNO 1
PROCNO 1

F2 - Acquisition Parameters
Date_ 20200915
Time 10.44 h
INSTRUM spect
PROBHD Z150453_0004 (
PULPROG zg30
TD 65536
SOLVENT DMSO
NS 32
DS 2
SWH 8012.820 Hz
FIDRES 0.244532 Hz
AQ 4.0894465 sec
RG 204.77
DW 62.400 usec
DE 6.50 usec
TE 298.1 K
D1 1.00000000 sec
TD0 1
SFO1 400.1324708 MHz
NUC1 1H
P0 3.33 usec
P1 10.00 usec
PLW1 16.87899971 W

F2 - Processing parameters
SI 65536
SF 400.1299920 MHz
WDW EM
SSB 0
LB 0.30 Hz
GB 0
PC 1.00

Inst.ID: CVL1-AD-NMR-001

Analyzed By: Ranjith

Checked by:

Ethyl 4-amino-3-nitrobenzoate (9):

Pages S21-S24

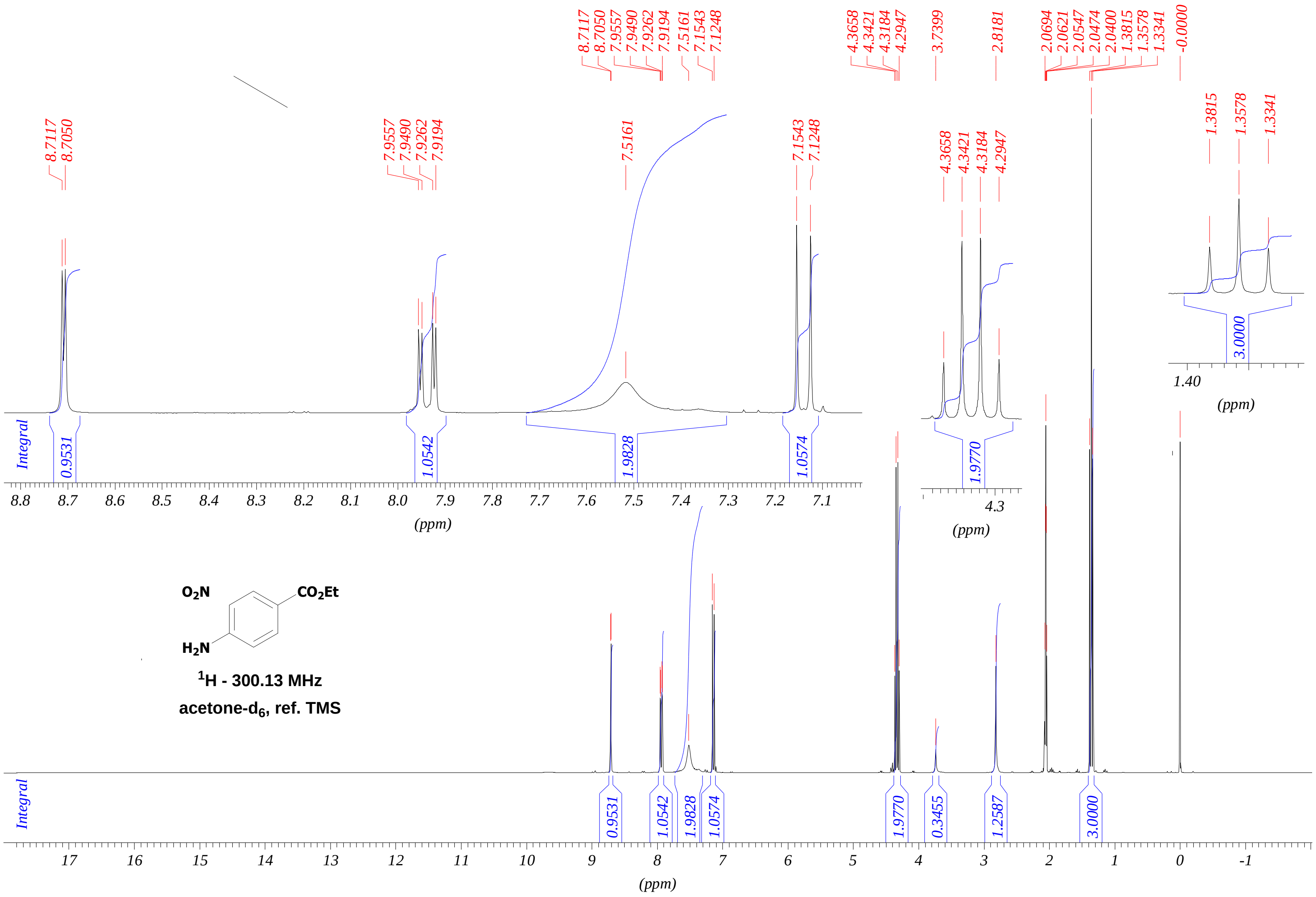

O_2N
CO_2Et
H_2N
^1H - 300.13 MHz
acetone-d_6, ref. TMS
Integral
(ppm)

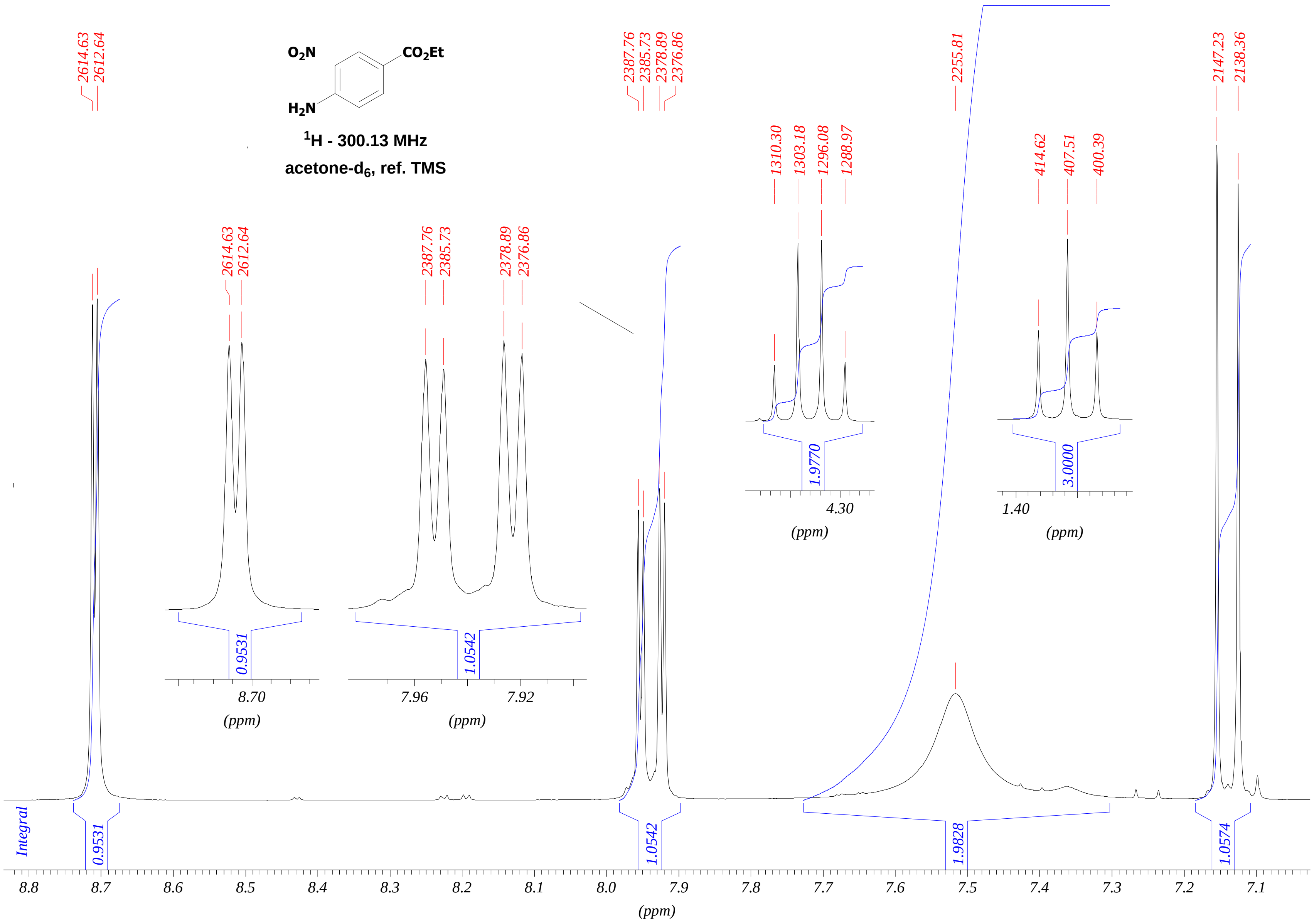
O2N
CO2Et
H2N
1H - 300.13 MHz
acetone-d6, ref. TMS
2614.63
2612.64
2387.76
2385.73
2378.89
2376.86
1310.30
1303.18
1296.08
1288.97
414.62
407.51
400.39
2255.81
2147.23
2138.36
0.9531
1.0542
1.9770
3.0000
1.9828
1.0574
Integral
(ppm)

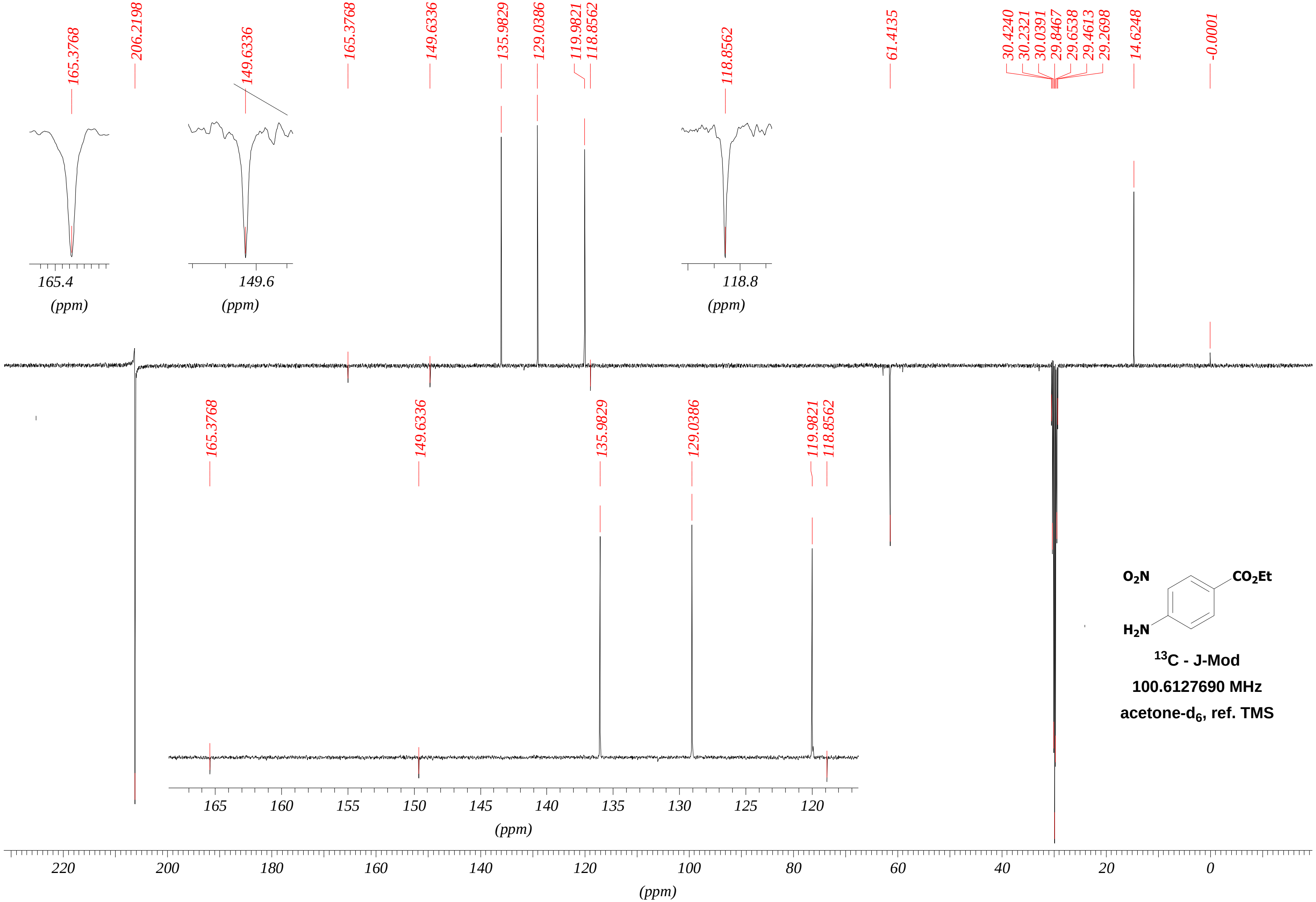

O_2N
CO_2Et
H_2N
^{13}C - J-Mod
100.6127690 MHz
acetone-d_6, ref. TMS
165.3768
206.2198
149.6336
135.9829
129.0386
119.9821
118.8562
61.4135
30.4240
30.2321
30.0391
29.8467
29.6538
29.4613
29.2698
14.6248
-0.0001
165.4
149.6
118.8
(ppm)

4-Amino-3-nitrobenzyl alcohol (10e):

Pages S25-S31

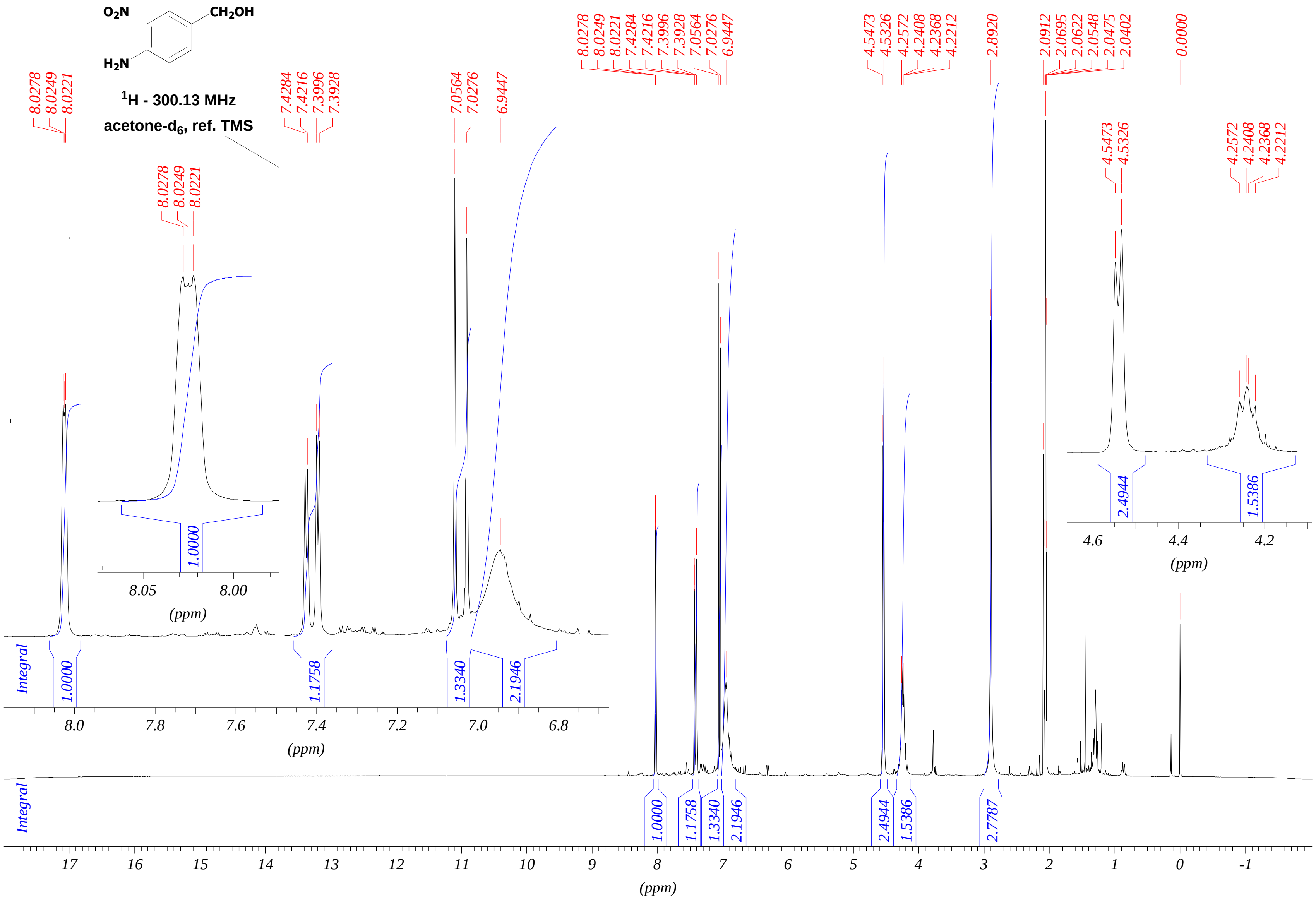

O2N
CH2OH
H2N
1H - 300.13 MHz
acetone-d6, ref. TMS
8.0278
8.0249
8.0221
7.4284
7.4216
7.3996
7.3928
7.0564
7.0276
6.9447
4.5473
4.5326
4.2572
4.2408
4.2368
4.2212
2.8920
2.0912
2.0695
2.0622
2.0548
2.0475
2.0402
0.0000
Integral
1.0000
1.1758
1.3340
2.1946
2.4944
1.5386
2.7787
(ppm)

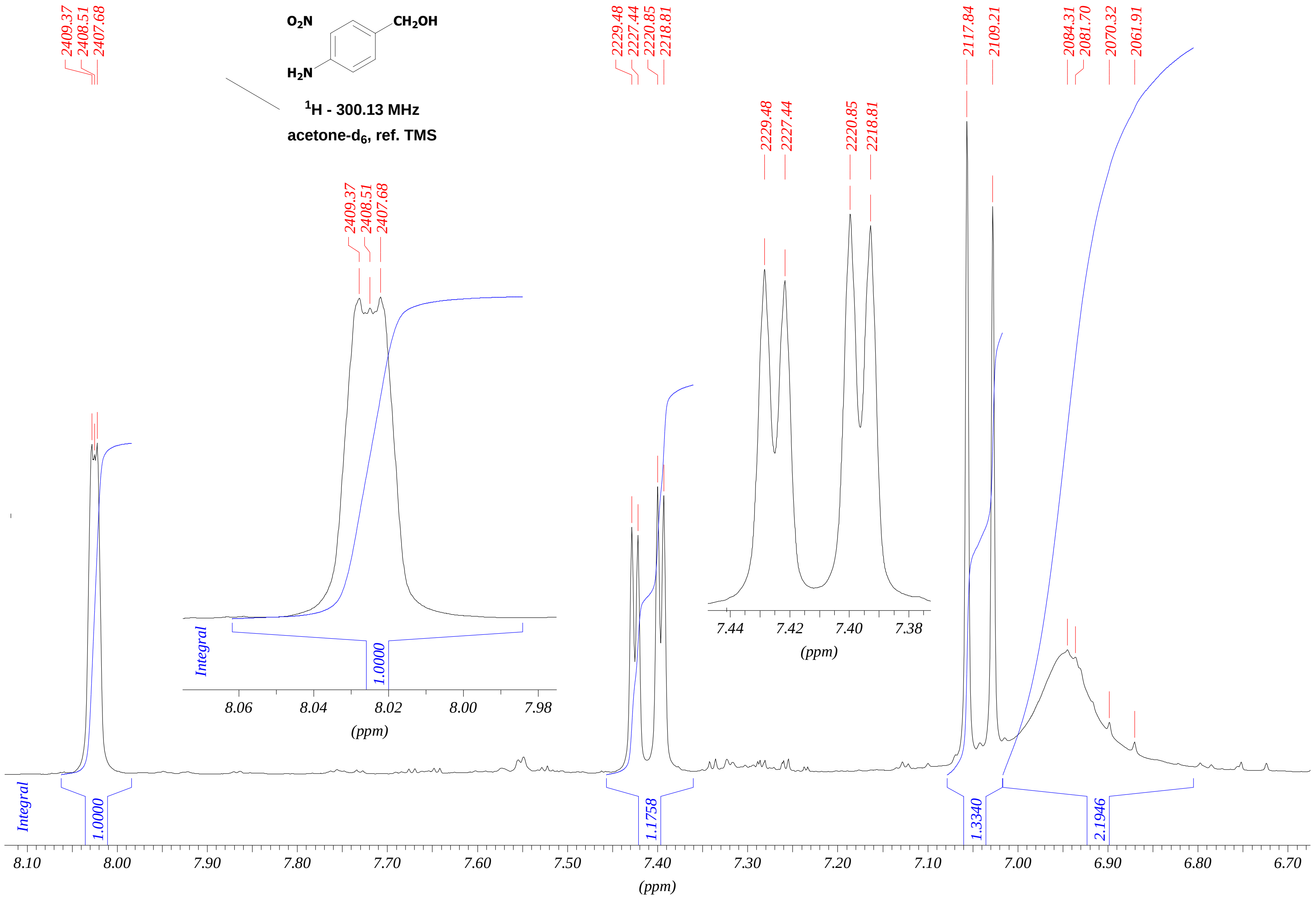

O2N
CH2OH
H2N
1H - 300.13 MHz
acetone-d6, ref. TMS
2409.37
2408.51
2407.68
2229.48
2227.44
2220.85
2218.81
2117.84
2109.21
2084.31
2081.70
2070.32
2061.91
Integral
1.0000
1.1758
1.3340
2.1946
8.10 8.00 7.90 7.80 7.70 7.60 7.50 7.40 7.30 7.20 7.10 7.00 6.90 6.80 6.70
(ppm)
8.06 8.04 8.02 8.00 7.98
7.44 7.42 7.40 7.38

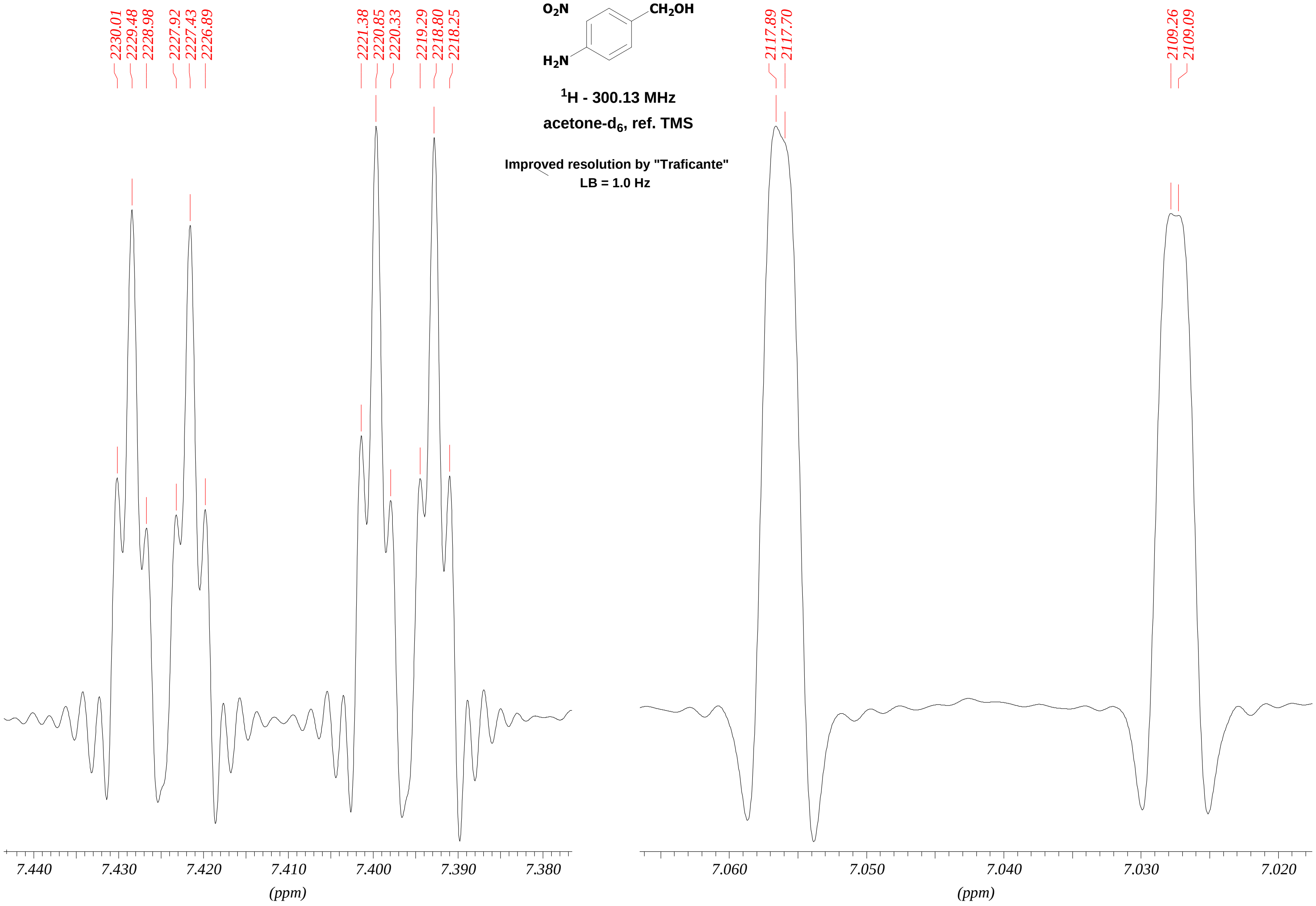
O2N
CH2OH
H2N
1H - 300.13 MHz
acetone-d6, ref. TMS
Improved resolution by "Traficante"
LB = 1.0 Hz
2230.01
2229.48
2228.98
2227.92
2227.43
2226.89
2221.38
2220.85
2220.33
2219.29
2218.80
2218.25
2117.89
2117.70
2109.26
2109.09
7.440
7.430
7.420
7.410
7.400
7.390
7.380
(ppm)
7.060
7.050
7.040
7.030
7.020
(ppm)

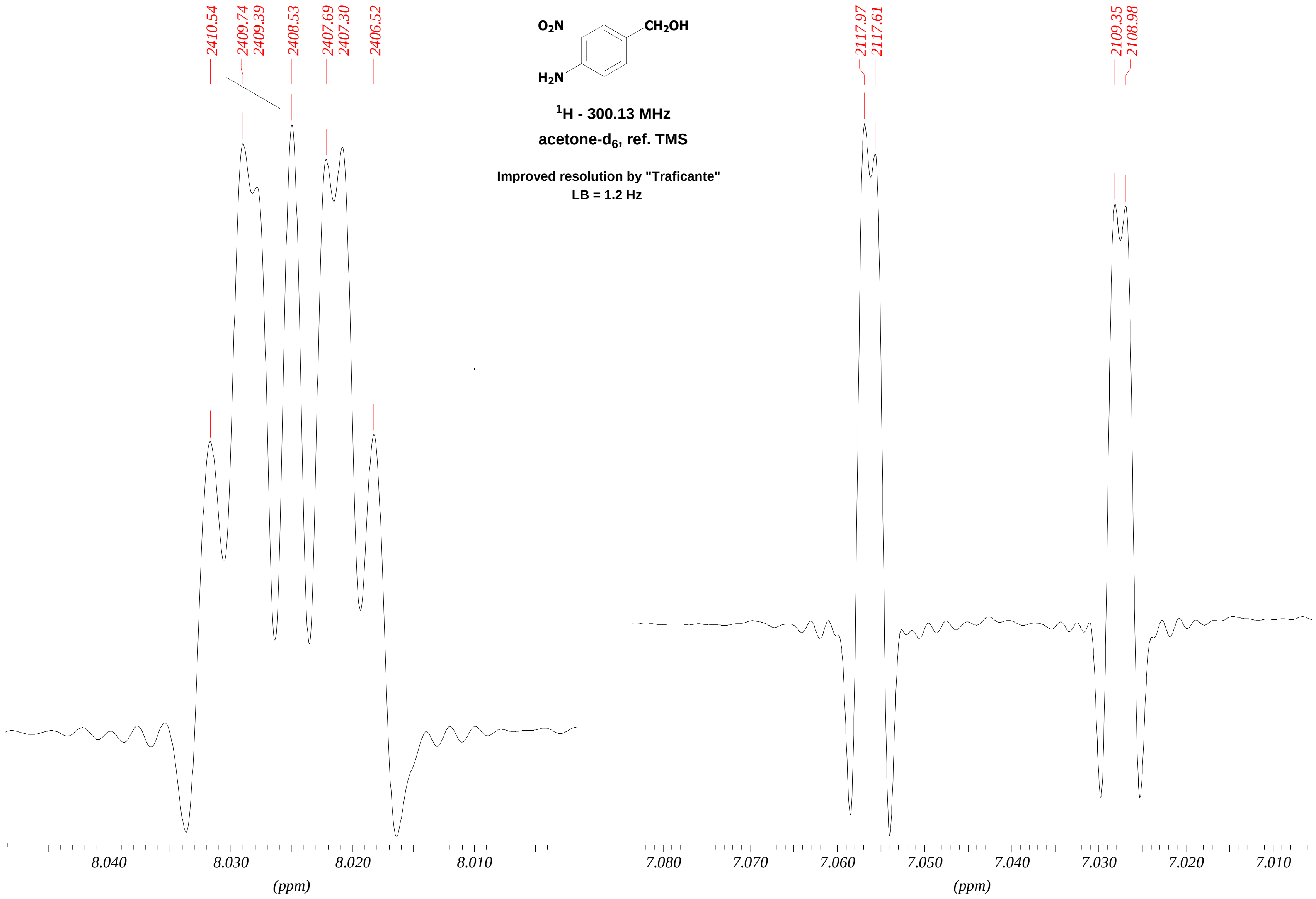

2410.54
2409.74
2409.39
2408.53
2407.69
2407.30
2406.52
O2N
CH2OH
H2N
1H - 300.13 MHz
acetone-d6, ref. TMS
Improved resolution by "Traficante"
LB = 1.2 Hz
2117.97
2117.61
2109.35
2108.98
8.040
8.030
8.020
8.010
(ppm)
7.080
7.070
7.060
7.050
7.040
7.030
7.020
7.010
(ppm)

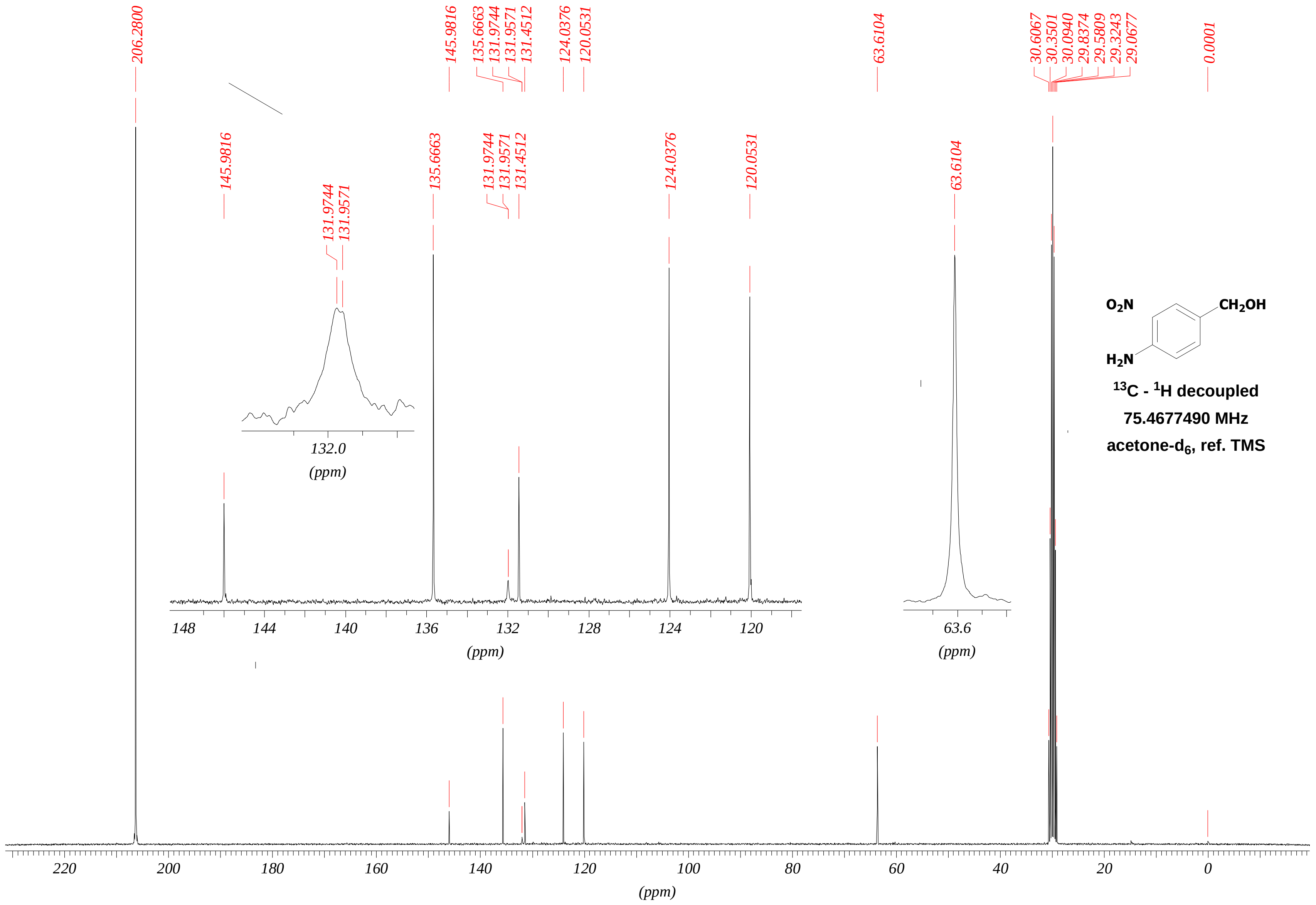
206.2800
145.9816
135.6663
131.9744
131.9571
131.4512
124.0376
120.0531
63.6104
30.6067
30.3501
30.0940
29.8374
29.5809
29.3243
29.0677
0.0001
132.0
(ppm)
148
144
140
136
132
128
124
120
63.6
O2N
CH2OH
H2N
13C - 1H decoupled
75.4677490 MHz
acetone-d6, ref. TMS
220
200
180
160
140
120
100
80
60
40
20
0

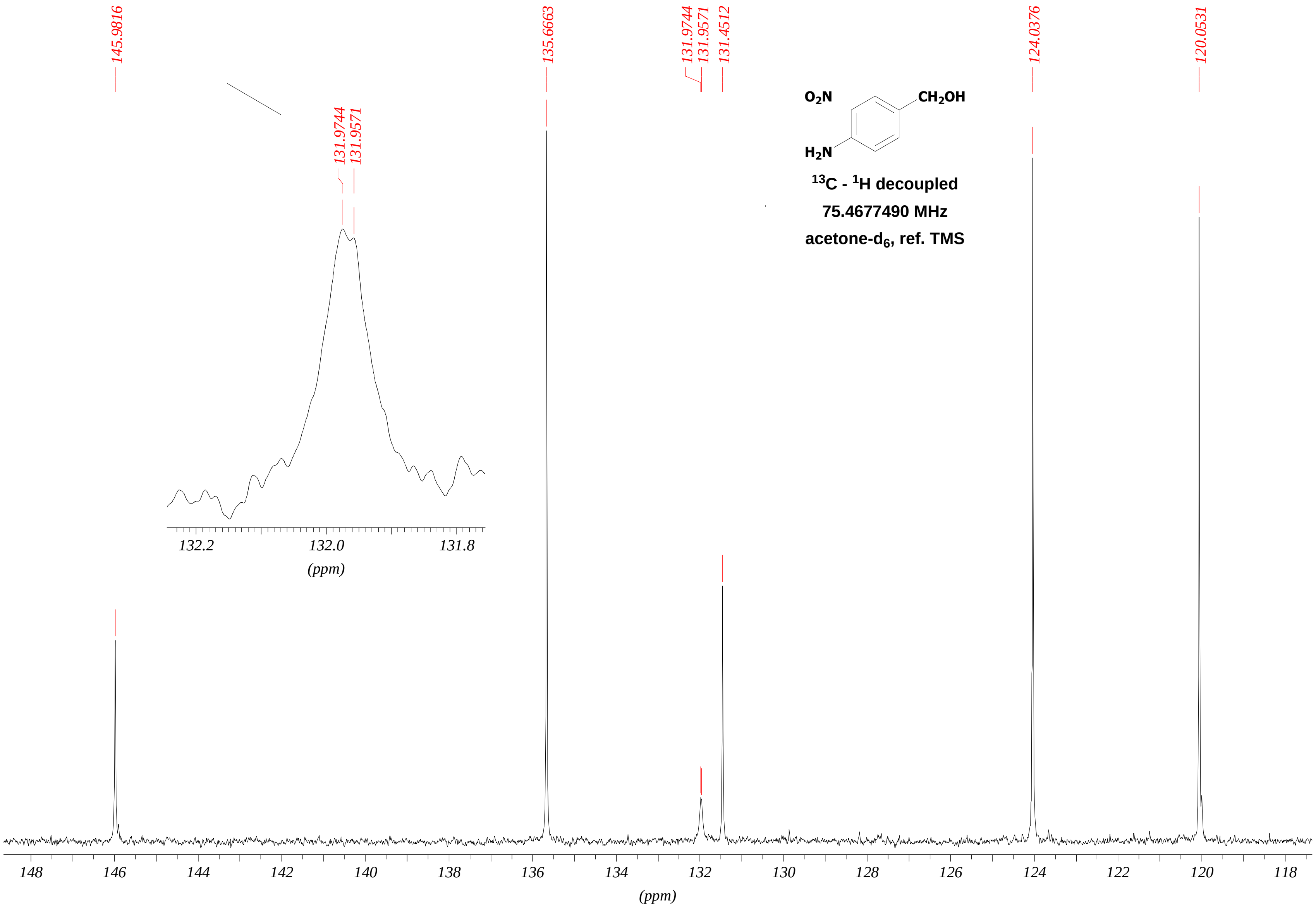
145.9816
135.6663
131.9744
131.9571
131.4512
124.0376
120.0531
131.9744
131.9571
132.2
132.0
131.8
(ppm)
O_2N
CH_2OH
H_2N
^{13}C - ^{1}H decoupled
75.4677490 MHz
acetone-d_6, ref. TMS
148
146
144
142
140
138
136
134
132
130
128
126
124
122
120
118
(ppm)

4-Azido-3-nitrophenol (11c):

Pages S32-S36

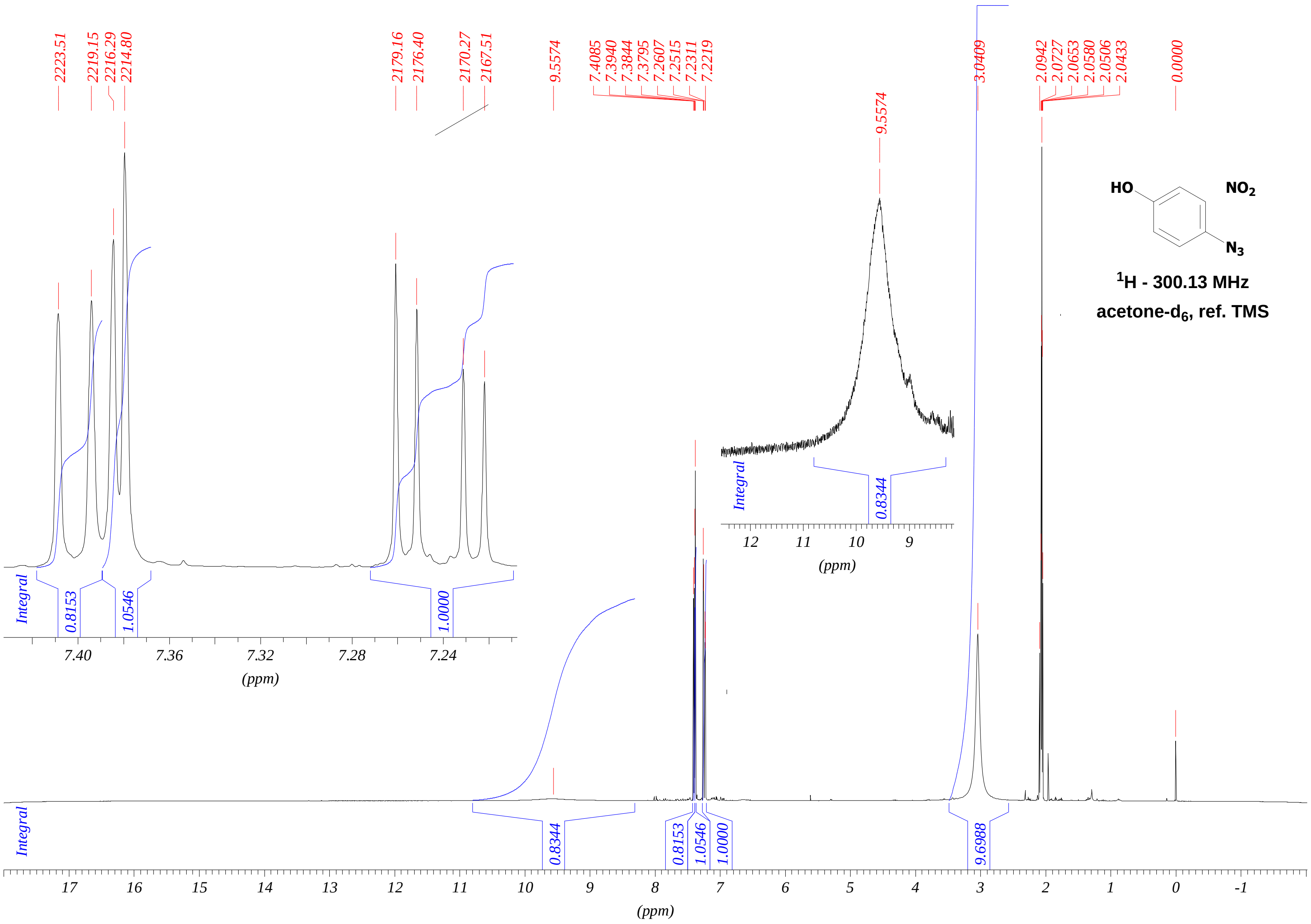

2223.51
2219.15
2216.29
2214.80
2179.16
2176.40
2170.27
2167.51
9.5574
7.4085
7.3940
7.3844
7.3795
7.2607
7.2515
7.2311
7.2219
3.0409
2.0942
2.0727
2.0653
2.0580
2.0506
2.0433
0.0000
HO
NO2
N3
1H - 300.13 MHz
acetone-d6, ref. TMS
Integral
0.8153
1.0546
1.0000
0.8344
9.6988
(ppm)

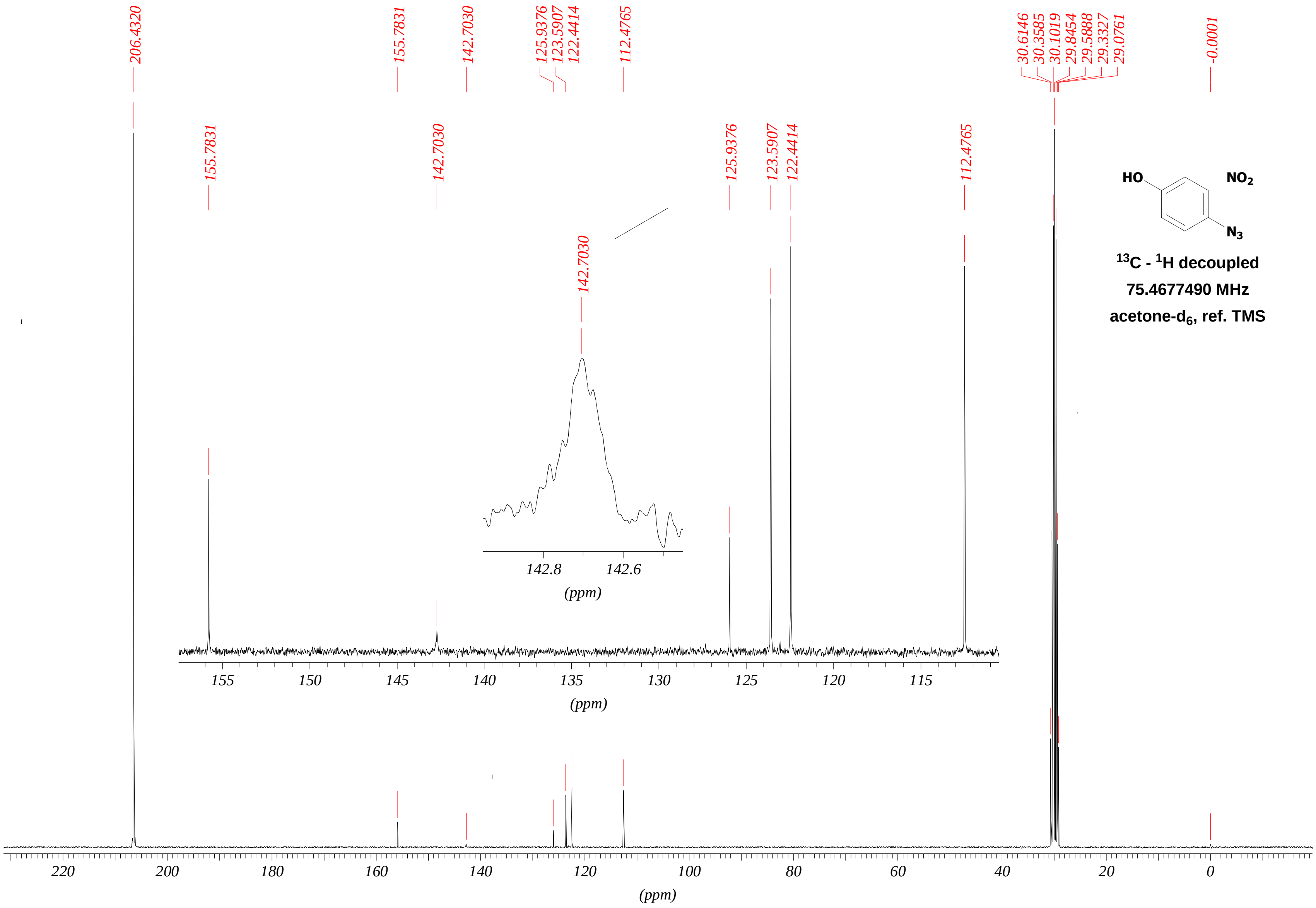
206.4320
155.7831
142.7030
125.9376
123.5907
122.4414
112.4765
30.6146
30.3585
30.1019
29.8454
29.5888
29.3327
29.0761
-0.0001
HO
NO2
N3
13C - 1H decoupled
75.4677490 MHz
acetone-d6, ref. TMS
142.8
142.6
(ppm)
155
150
145
140
135
130
125
120
115
220
200
180
160
140
120
100
80
60
40
20
0

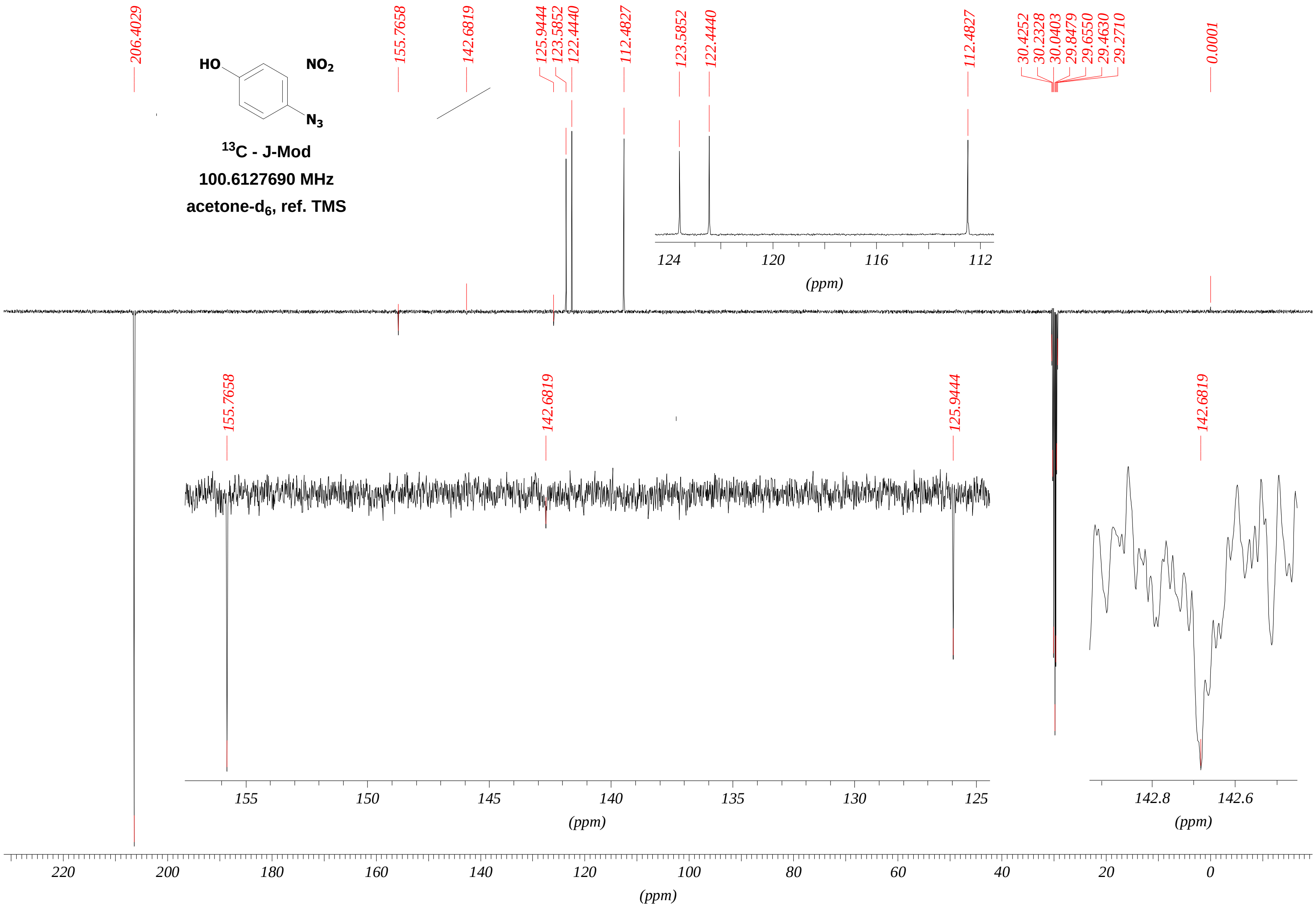
HO
NO2
N3
13C - J-Mod
100.6127690 MHz
acetone-d6, ref. TMS
206.4029
155.7658
142.6819
125.9444
123.5852
122.4440
112.4827
30.4252
30.2328
30.0403
29.8479
29.6550
29.4630
29.2710
0.0001
(ppm)

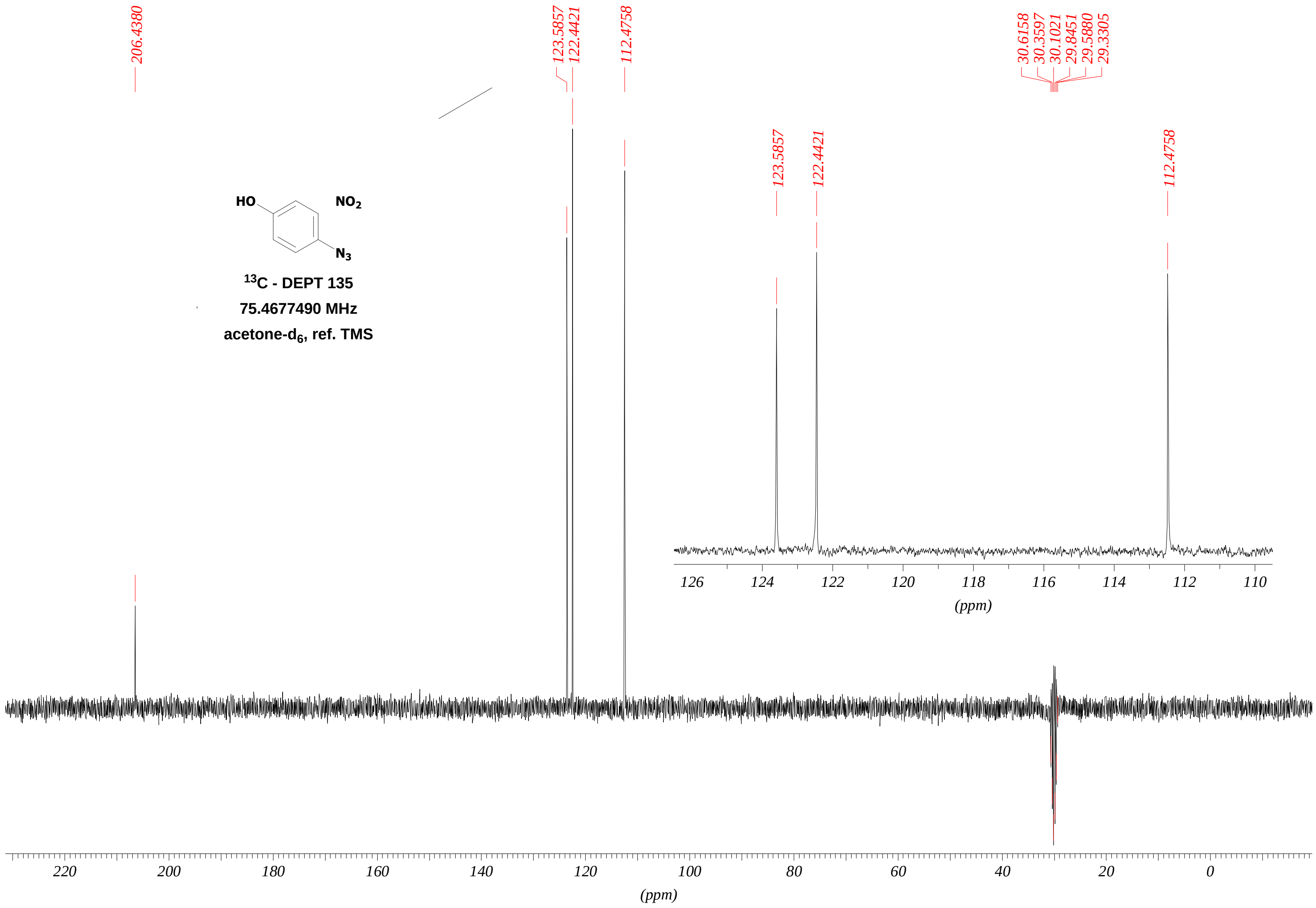
206.4380
123.5857
122.4421
112.4758
30.6158
30.3597
30.1021
29.8451
29.5880
29.3305
HO
NO_2
N_3
^{13}C - DEPT 135
75.4677490 MHz
acetone-d_6, ref. TMS
123.5857
122.4421
112.4758
126
124
122
120
118
116
114
112
110
(ppm)
220
200
180
160
140
120
100
80
60
40
20
0
(ppm)

1-Azido-4-bromo-2-nitrobenzene (11d):

Pages S37-S41

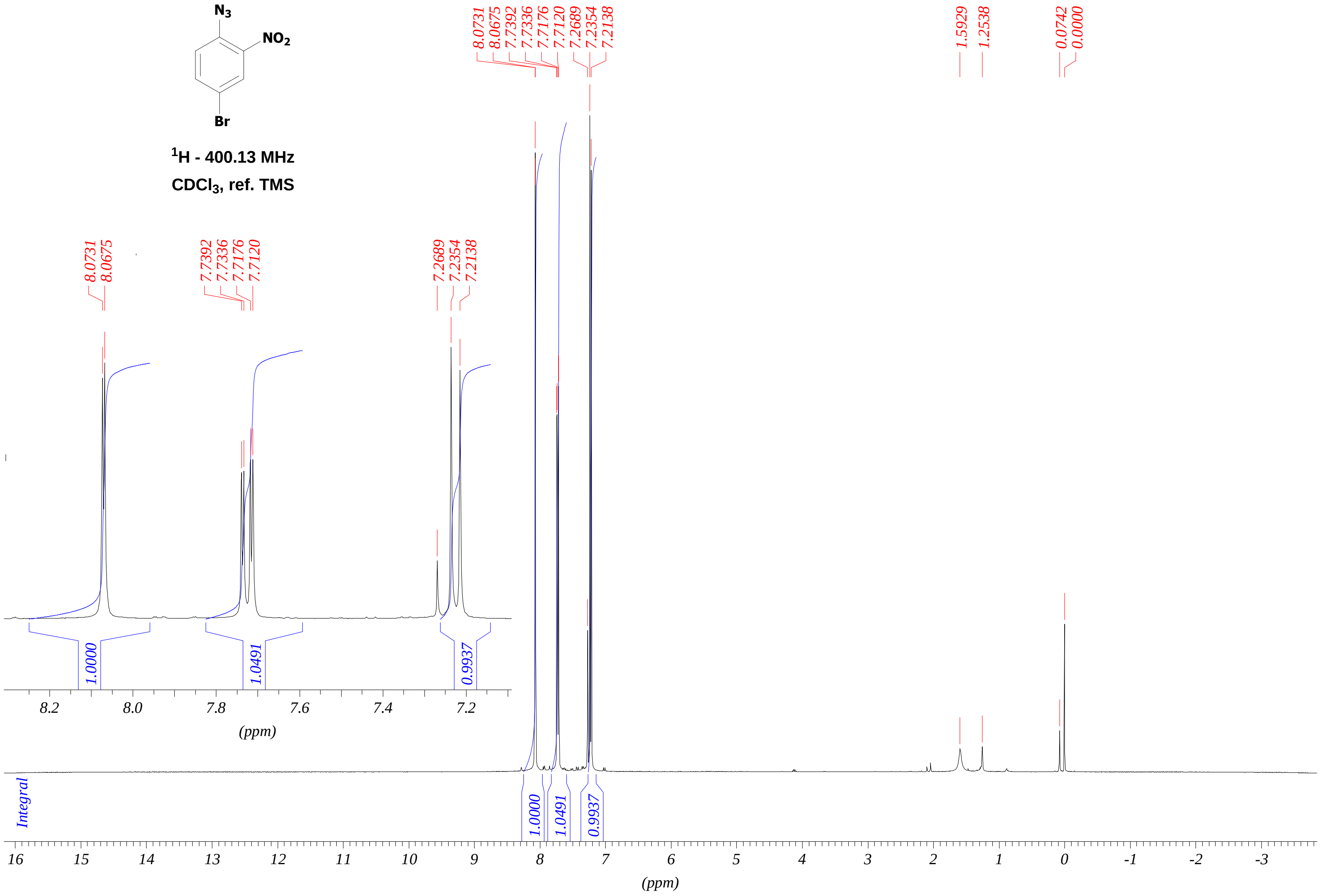

N3
NO2
Br
1H - 400.13 MHz
CDCl3, ref. TMS
8.0731
8.0675
7.7392
7.7336
7.7176
7.7120
7.2689
7.2354
7.2138
1.5929
1.2538
0.0742
0.0000
1.0000
1.0491
0.9937
Integral
(ppm)

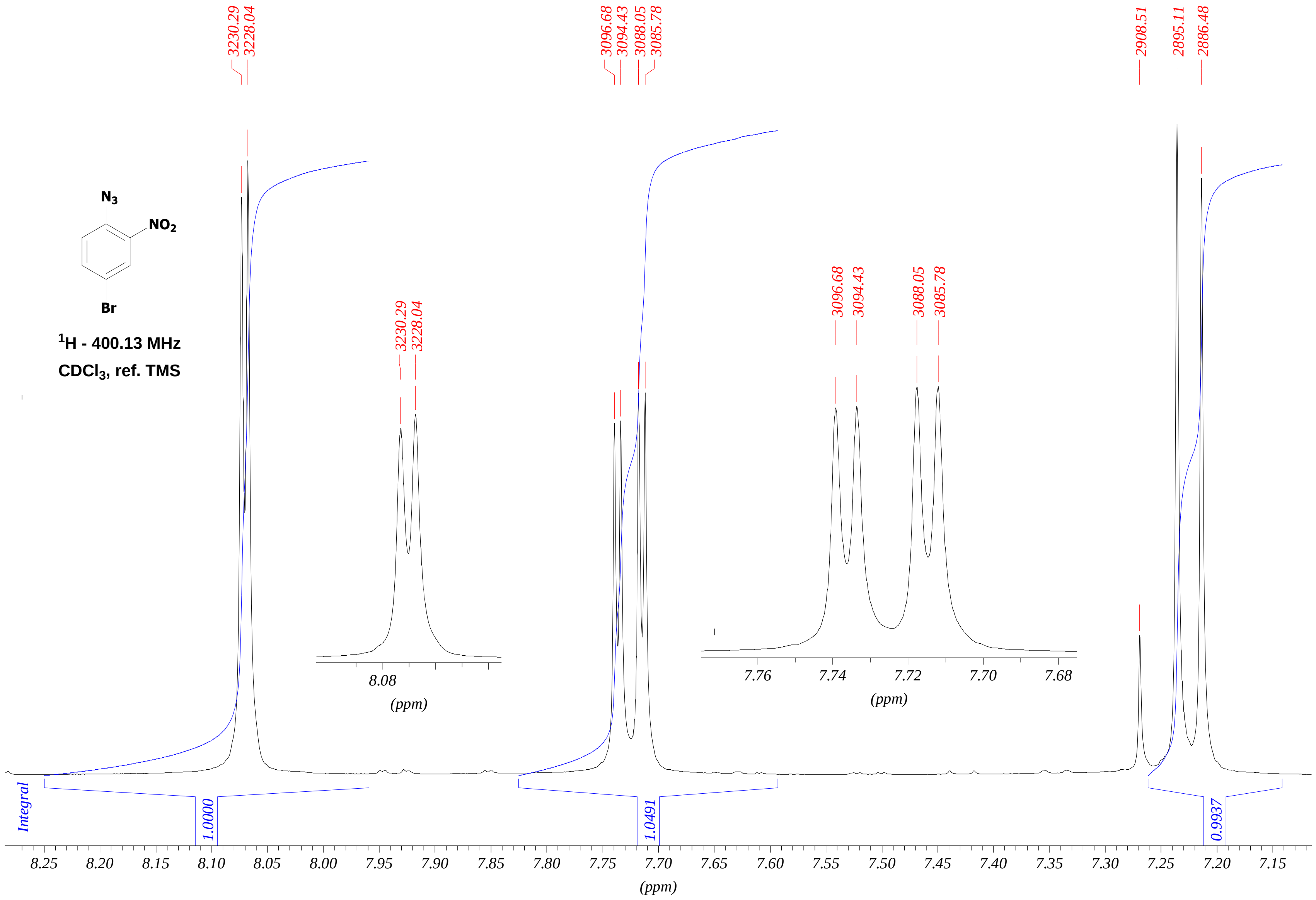
N3
NO2
Br
1H - 400.13 MHz
CDCl3, ref. TMS
3230.29
3228.04
3096.68
3094.43
3088.05
3085.78
2908.51
2895.11
2886.48
Integral
1.0000
1.0491
0.9937
(ppm)

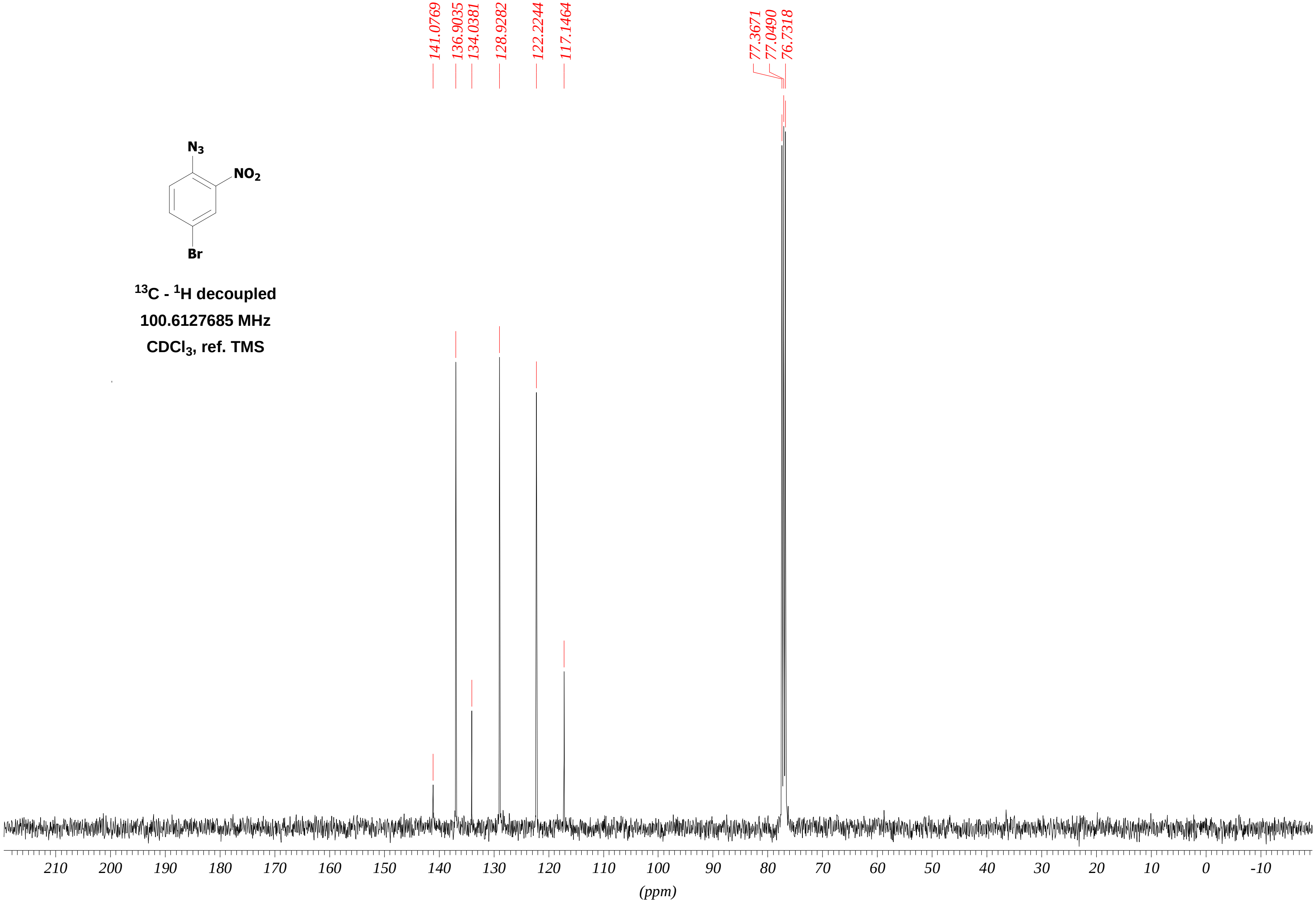

^{13}C - ^{1}H decoupled
100.6127685 MHz
$CDCl_3$, ref. TMS
141.0769
136.9035
134.0381
128.9282
122.2244
117.1464
77.3671
77.0490
76.7318
210 200 190 180 170 160 150 140 130 120 110 100 90 80 70 60 50 40 30 20 10 0 -10
(ppm)

^{13}C - ^{1}H decoupled

100.6127685 MHz

$CDCl_3$, ref. TMS

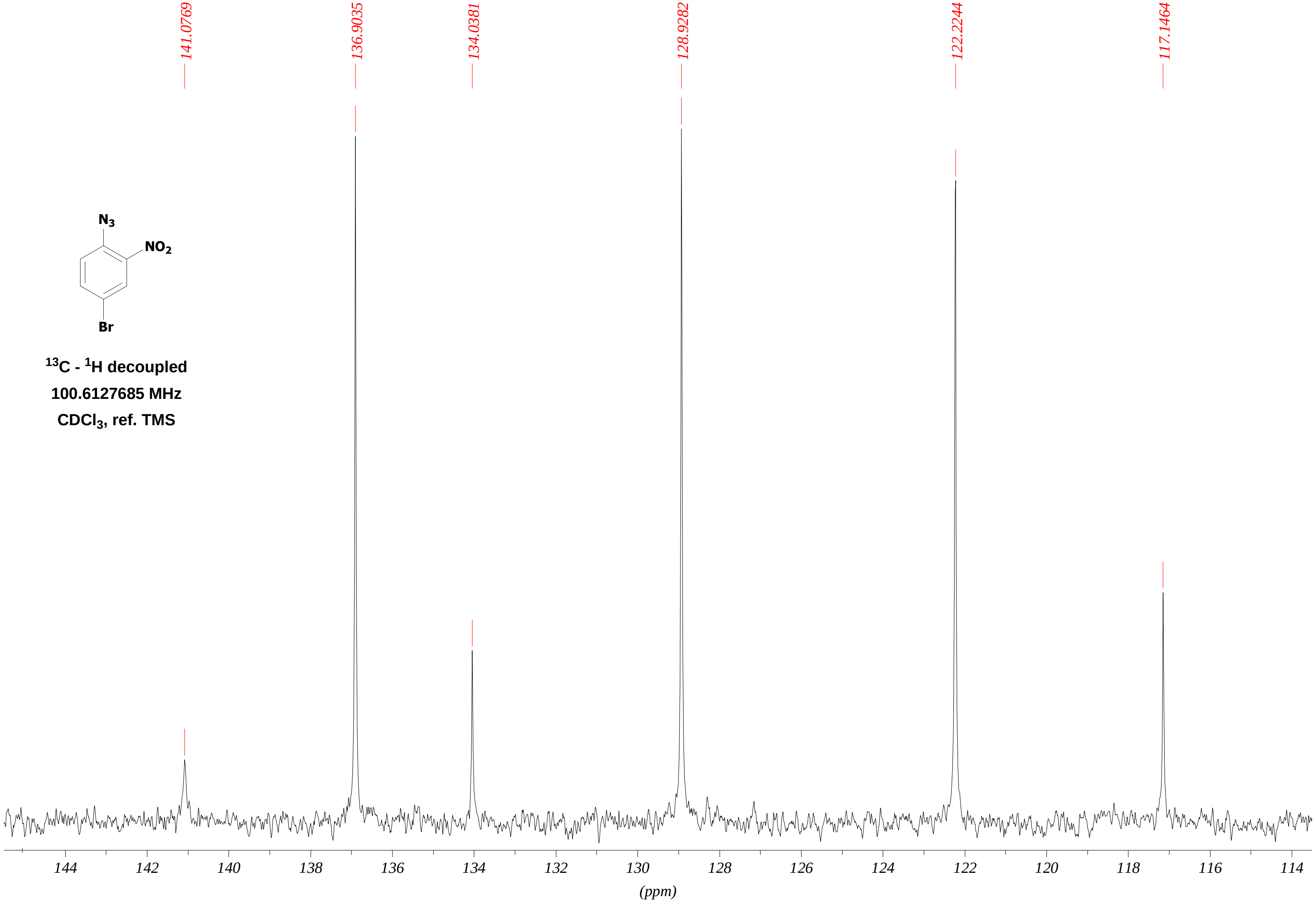

4-Azido-3-nitrobenzyl alcohol (11e):

Pages S42-S48

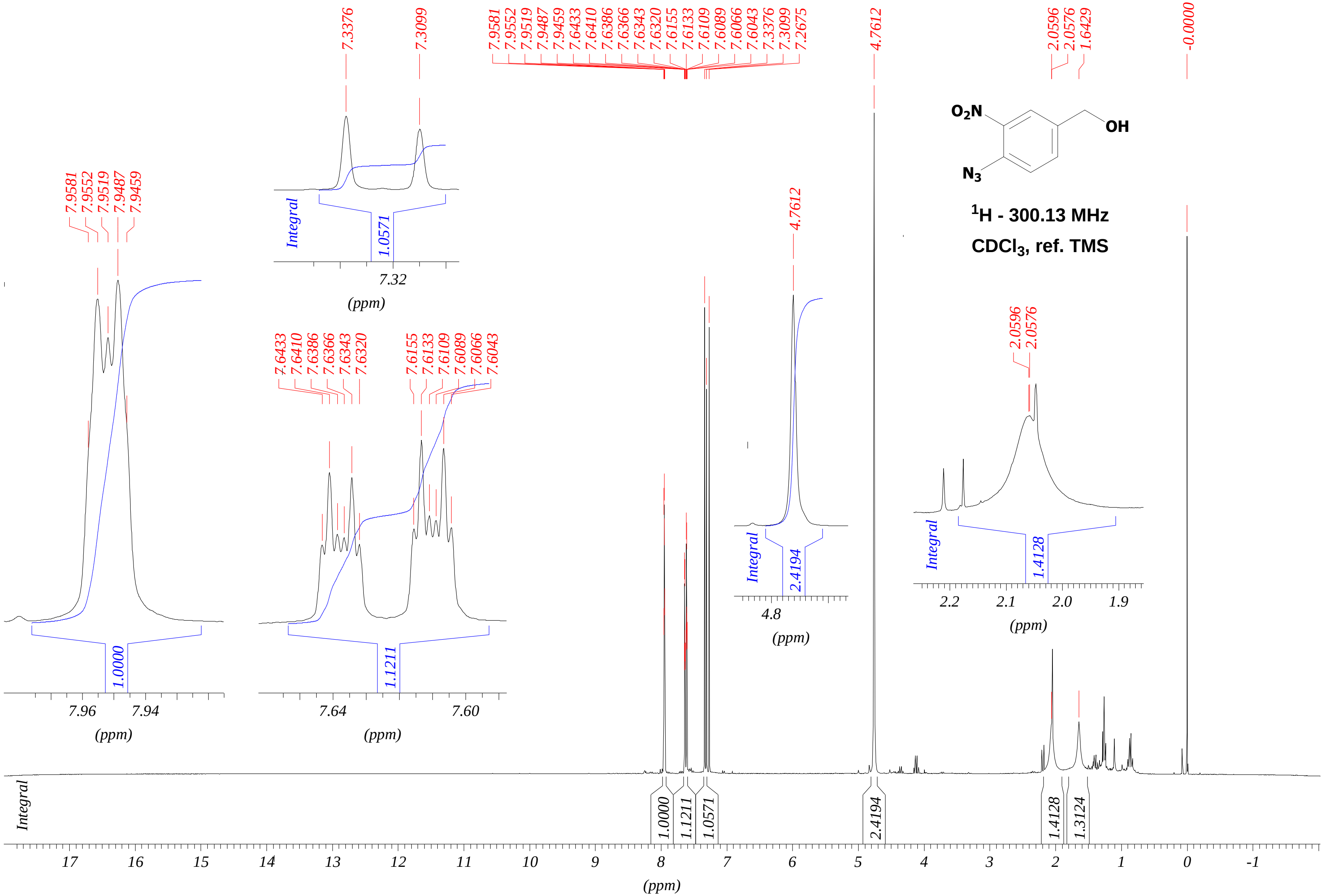

O2N
OH
N3
1H - 300.13 MHz
CDCl3, ref. TMS
(ppm)
Integral

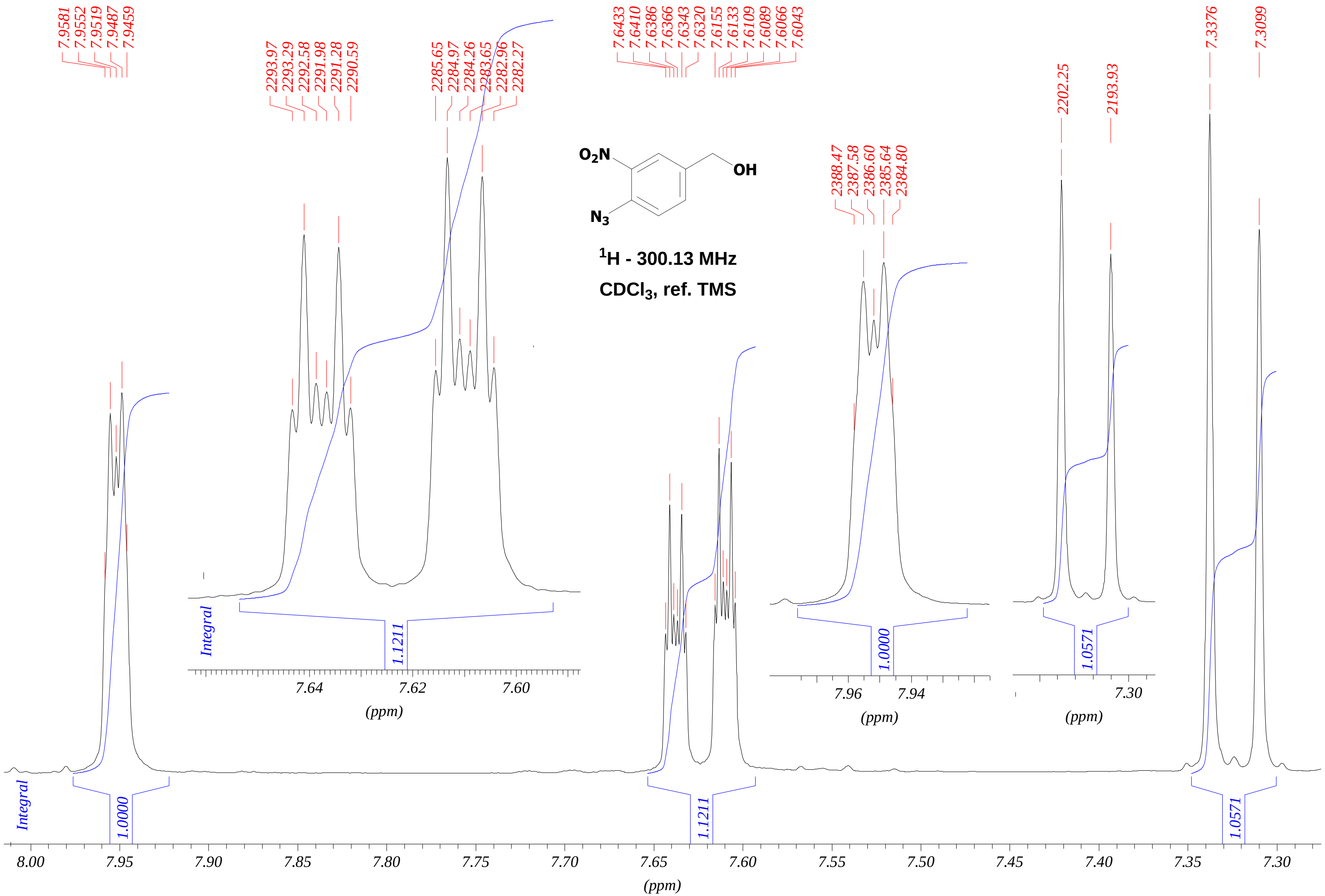

O2N
N3
OH
1H - 300.13 MHz
CDCl3, ref. TMS
7.9581
7.9552
7.9519
7.9487
7.9459
2293.97
2293.29
2292.58
2291.98
2291.28
2290.59
2285.65
2284.97
2284.26
2283.65
2282.96
2282.27
7.6433
7.6410
7.6386
7.6366
7.6343
7.6320
7.6155
7.6133
7.6109
7.6089
7.6066
7.6043
2388.47
2387.58
2386.60
2385.64
2384.80
2202.25
2193.93
7.3376
7.3099
Integral
1.0000
1.1211
1.0571
(ppm)

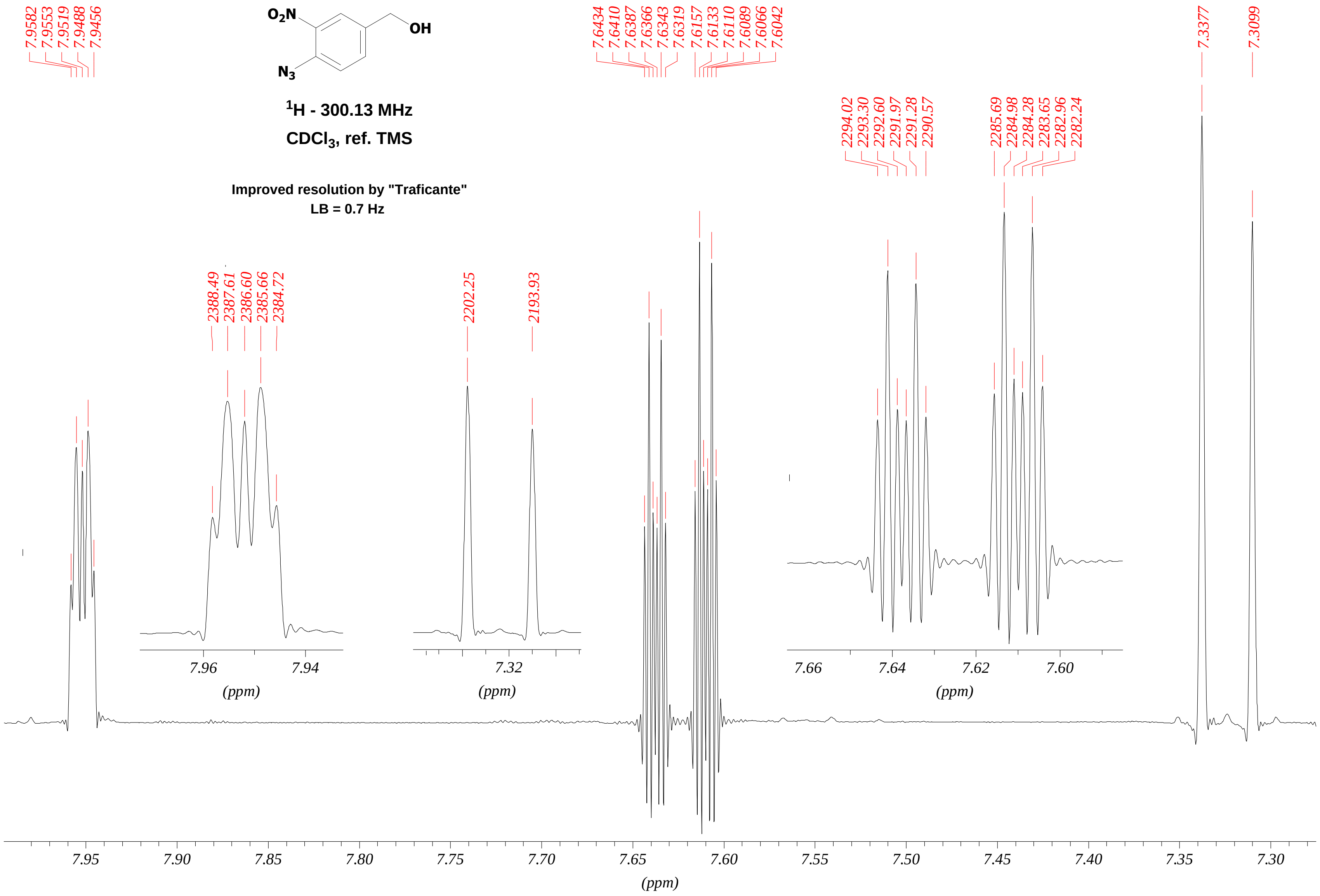

O2N
N3
OH
1H - 300.13 MHz
CDCl3, ref. TMS
Improved resolution by "Traficante"
LB = 0.7 Hz
7.9582
7.9553
7.9519
7.9488
7.9456
7.6434
7.6410
7.6387
7.6366
7.6343
7.6319
7.6157
7.6133
7.6110
7.6089
7.6066
7.6042
7.3377
7.3099
2388.49
2387.61
2386.60
2385.66
2384.72
2202.25
2193.93
2294.02
2293.30
2292.60
2291.97
2291.28
2290.57
2285.69
2284.98
2284.28
2283.65
2282.96
2282.24
7.96
7.94
(ppm)
7.32
(ppm)
7.66
7.64
7.62
7.60
(ppm)
7.95
7.90
7.85
7.80
7.75
7.70
7.65
7.60
7.55
7.50
7.45
7.40
7.35
7.30
(ppm)

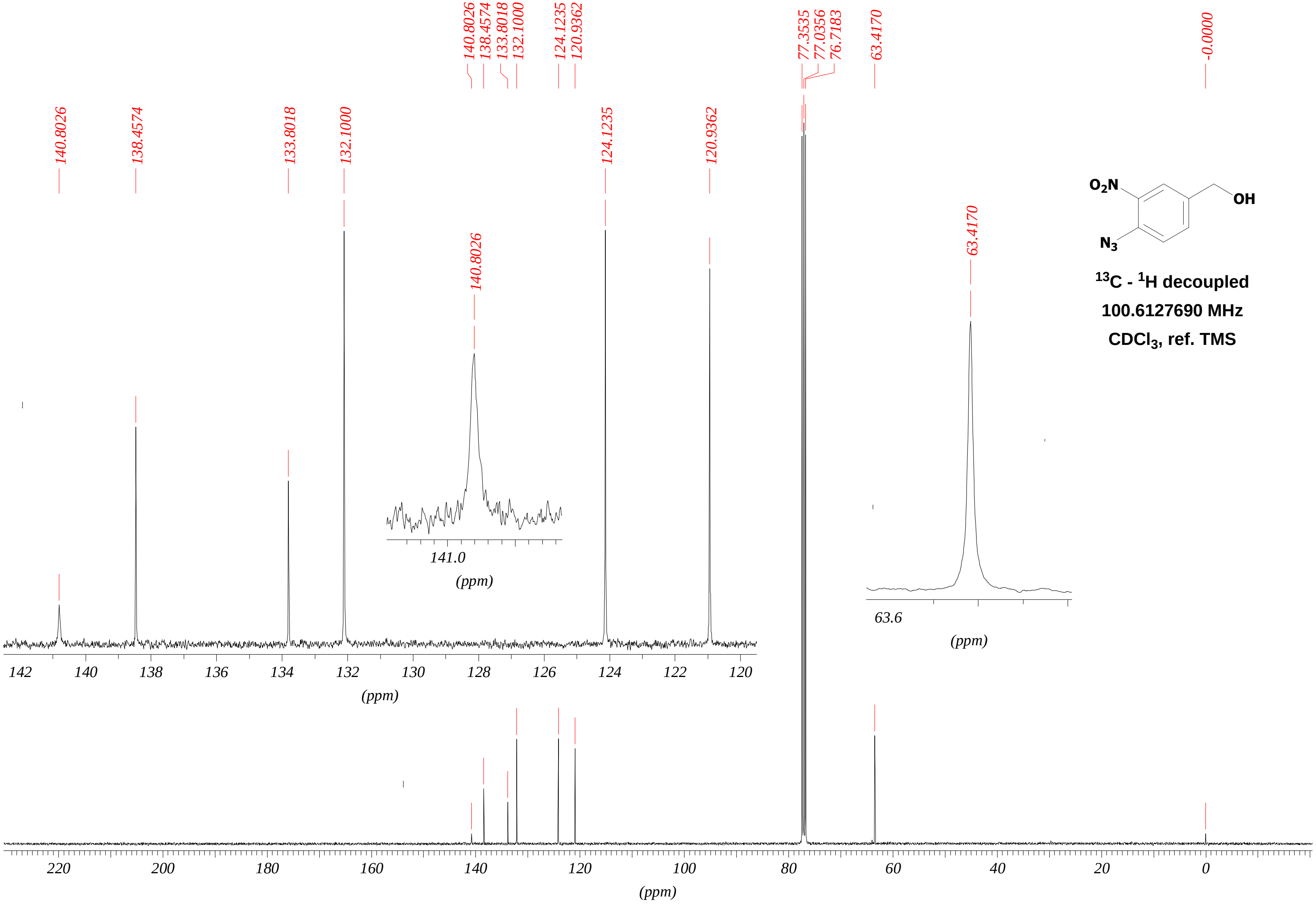
140.8026
138.4574
133.8018
132.1000
124.1235
120.9362
77.3535
77.0356
76.7183
63.4170
-0.0000
^{13}C - ^{1}H decoupled
100.6127690 MHz
$CDCl_3$, ref. TMS
O_2N
N_3
OH
141.0
63.6
(ppm)

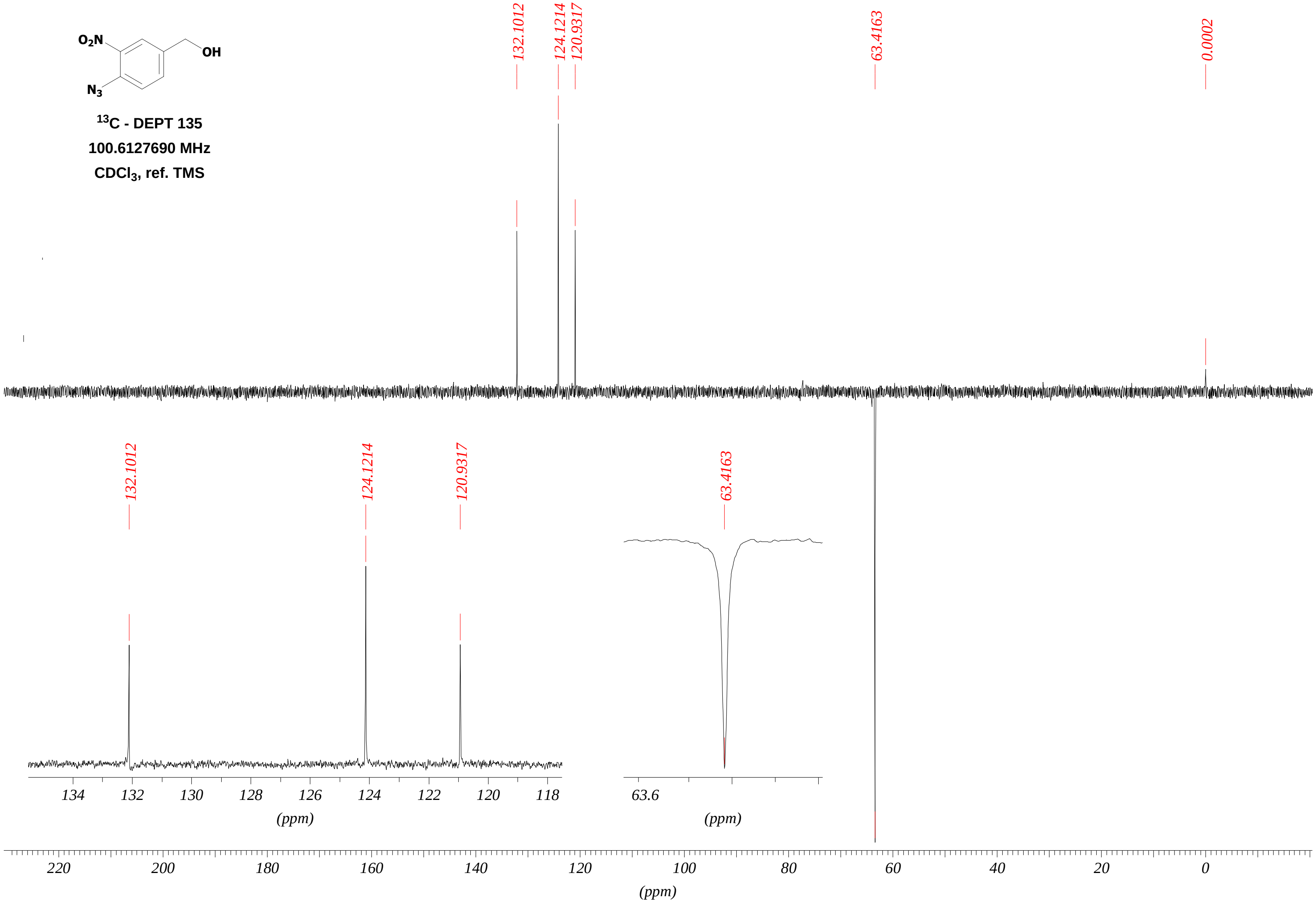

O_2N
OH
N_3
^{13}C - DEPT 135
100.6127690 MHz
$CDCl_3$, ref. TMS
132.1012
124.1214
120.9317
63.4163
0.0002
134
132
130
128
126
124
122
120
118
(ppm)
63.6
(ppm)
220
200
180
160
140
120
100
80
60
40
20
0
(ppm)

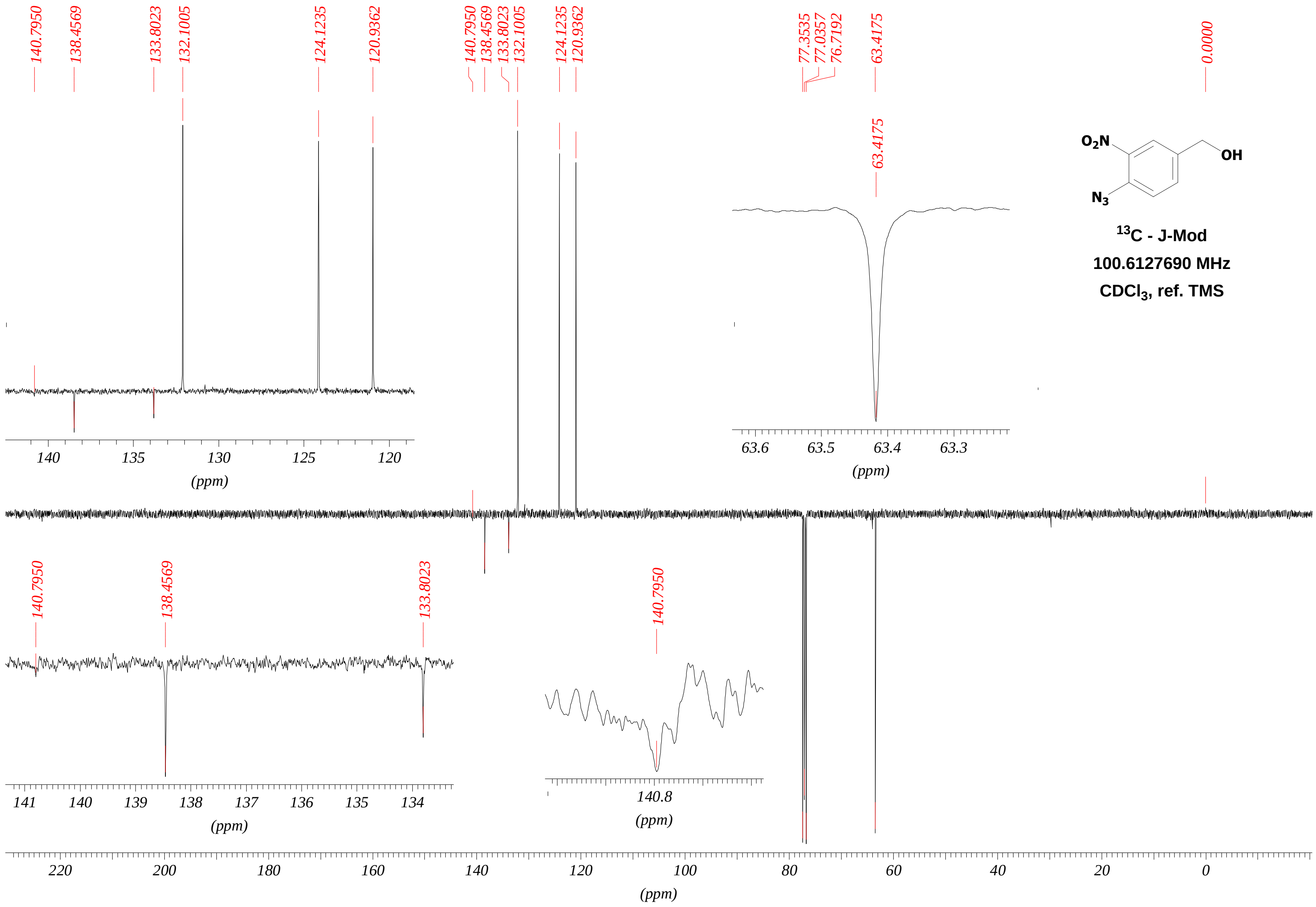

^{13}C - J-Mod
100.6127690 MHz
$CDCl_3$, ref. TMS
O2N
N3
OH
140.7950
138.4569
133.8023
132.1005
124.1235
120.9362
77.3535
77.0357
76.7192
63.4175
0.0000
(ppm)

_N_-(3-Chlorophenyl)-8-((1-(4-hydroxy-2-nitrophenyl)-1_H_-1,2,3-triazol-4-yl)methoxy)quinazolin-2-amine (12c):

Pages S49-S63

In the ^{1}H-NMR spectrum, apart the usual big peak of water at 3.40 ppm which is contained in DMSO-d_6, there is a small additional peak at 2.09 ppm which is due to residual acetone which was used for the purification of this compound.

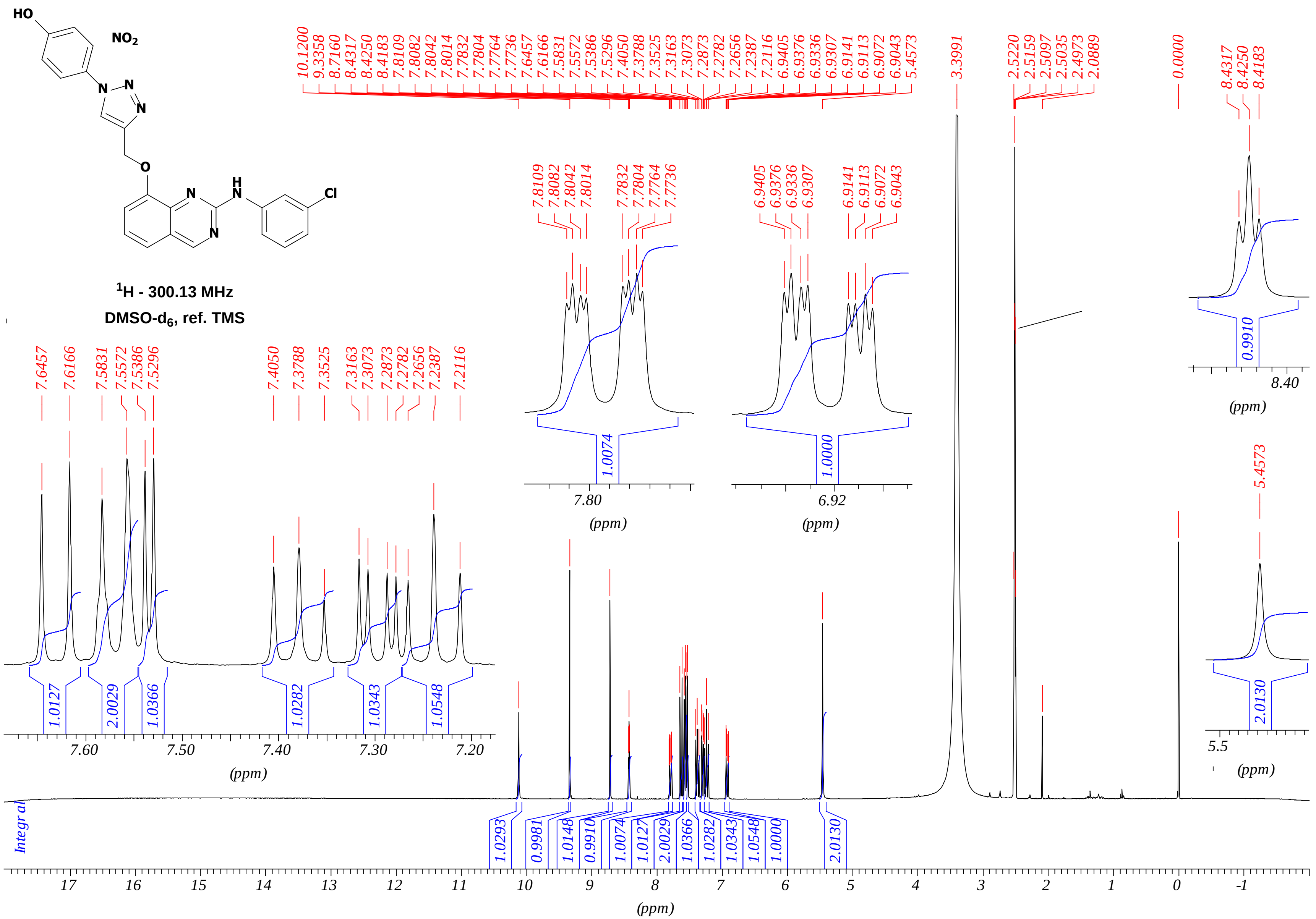

HO
NO2
N
O
H
Cl
1H - 300.13 MHz
DMSO-d6, ref. TMS
10.1200 9.3358 8.7160 8.4317 8.4250 8.4183 7.8109 7.8082 7.8042 7.8014 7.7832 7.7804 7.7764 7.7736 7.6457 7.6166 7.5831 7.5572 7.5386 7.5296 7.4050 7.3788 7.3525 7.3163 7.3073 7.2873 7.2782 7.2656 7.2387 7.2116 6.9405 6.9376 6.9336 6.9307 6.9141 6.9113 6.9072 6.9043 5.4573
3.3991
2.5220 2.5159 2.5097 2.5035 2.4973 2.0889
0.0000
(ppm)

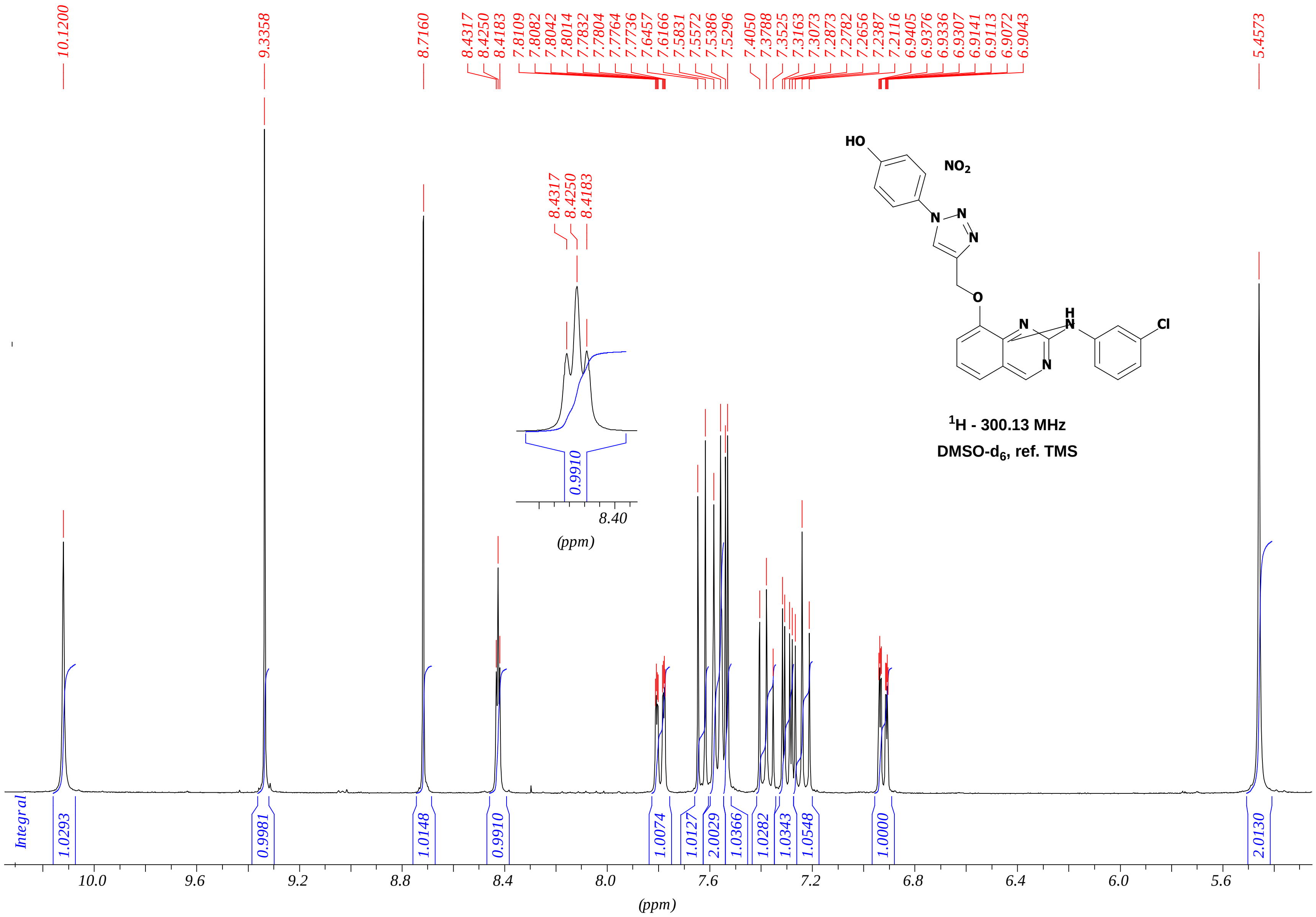

¹H - 300.13 MHz
DMSO-d6, ref. TMS
HO
NO2
N
Cl
O
H
Integral
(ppm)
10.1200
9.3358
8.7160
8.4317
8.4250
8.4183
5.4573

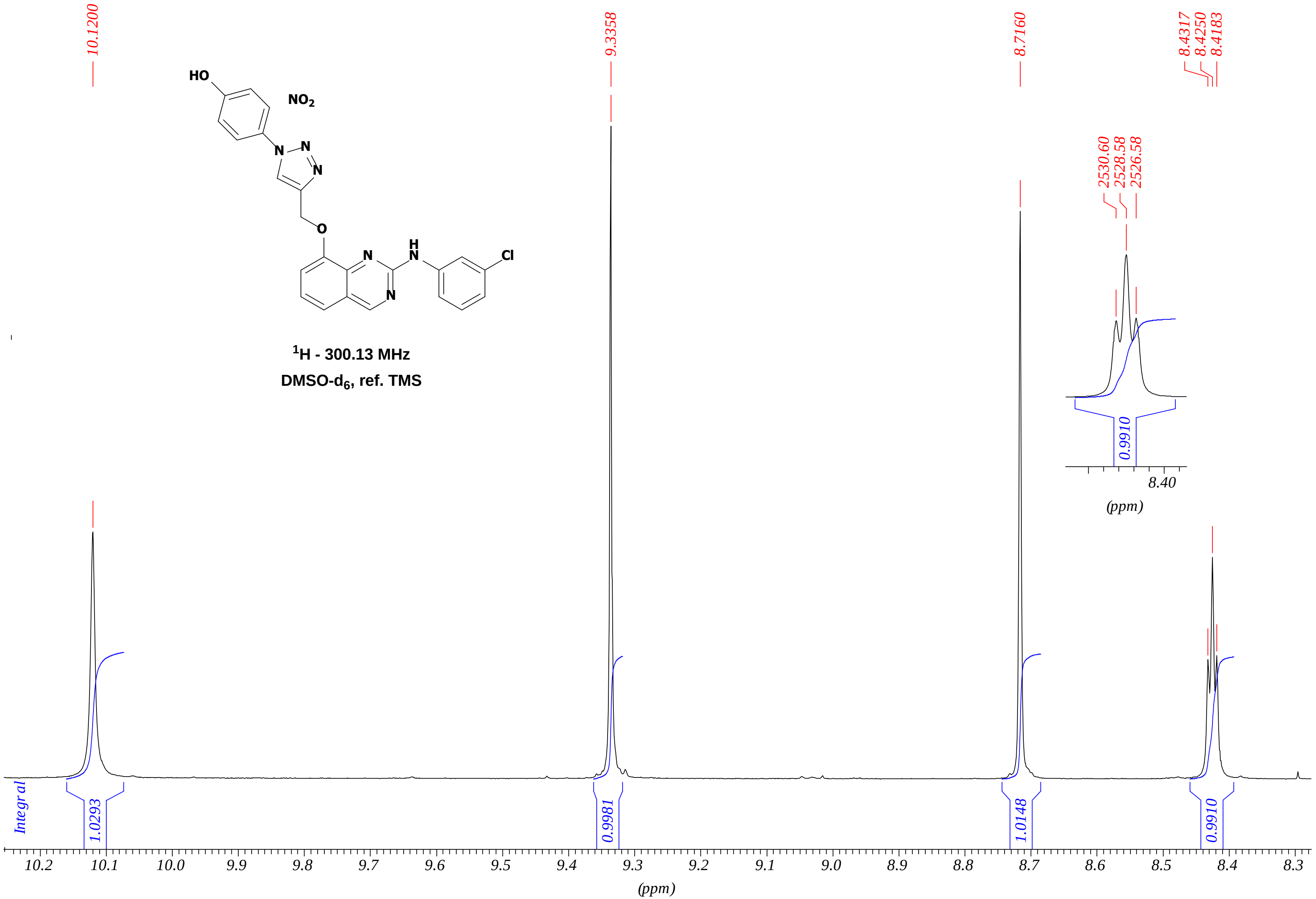
HO
NO_2
N
N
N
O
N
N
H
N
Cl
^{1}H - 300.13 MHz
DMSO-d_6, ref. TMS
10.1200
9.3358
8.7160
8.4317
8.4250
8.4183
2530.60
2528.58
2526.58
0.9910
8.40
(ppm)
Integral
1.0293
0.9981
1.0148
0.9910
10.2
10.1
10.0
9.9
9.8
9.7
9.6
9.5
9.4
9.3
9.2
9.1
9.0
8.9
8.8
8.7
8.6
8.5
8.4
8.3
(ppm)

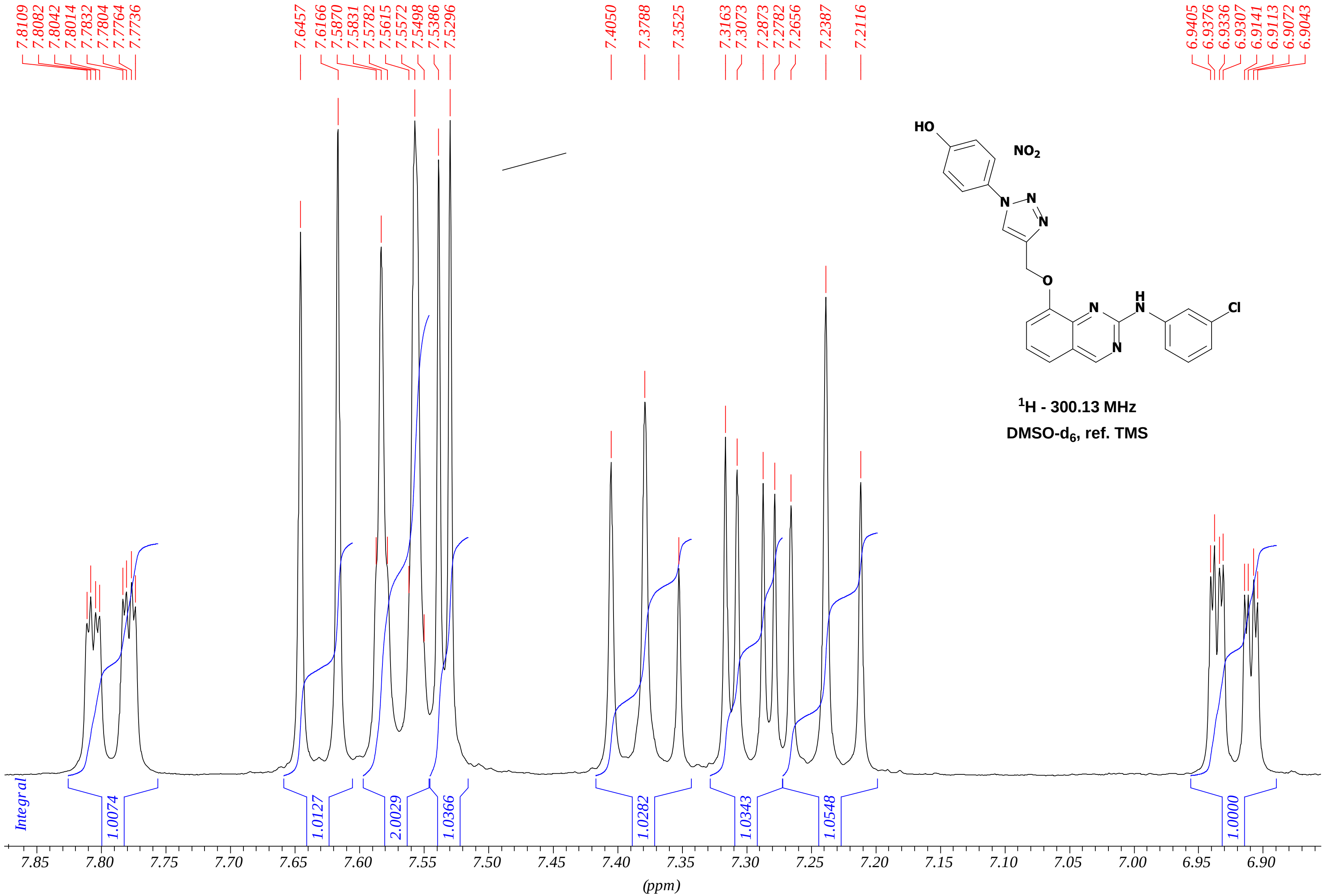

7.8109 7.8082 7.8042 7.8014 7.7832 7.7804 7.7764 7.7736
7.6457 7.6166 7.5870 7.5831 7.5782 7.5615 7.5572 7.5498 7.5386 7.5296
7.4050 7.3788 7.3525
7.3163 7.3073 7.2873 7.2782 7.2656 7.2387 7.2116
6.9405 6.9376 6.9336 6.9307 6.9141 6.9113 6.9072 6.9043
HO
NO2
N
N
N
O
H
N
Cl
N
N
1H - 300.13 MHz
DMSO-d6, ref. TMS
Integral
1.0074
1.0127
2.0029
1.0366
1.0282
1.0343
1.0548
1.0000
7.85 7.80 7.75 7.70 7.65 7.60 7.55 7.50 7.45 7.40 7.35 7.30 7.25 7.20 7.15 7.10 7.05 7.00 6.95 6.90
(ppm)

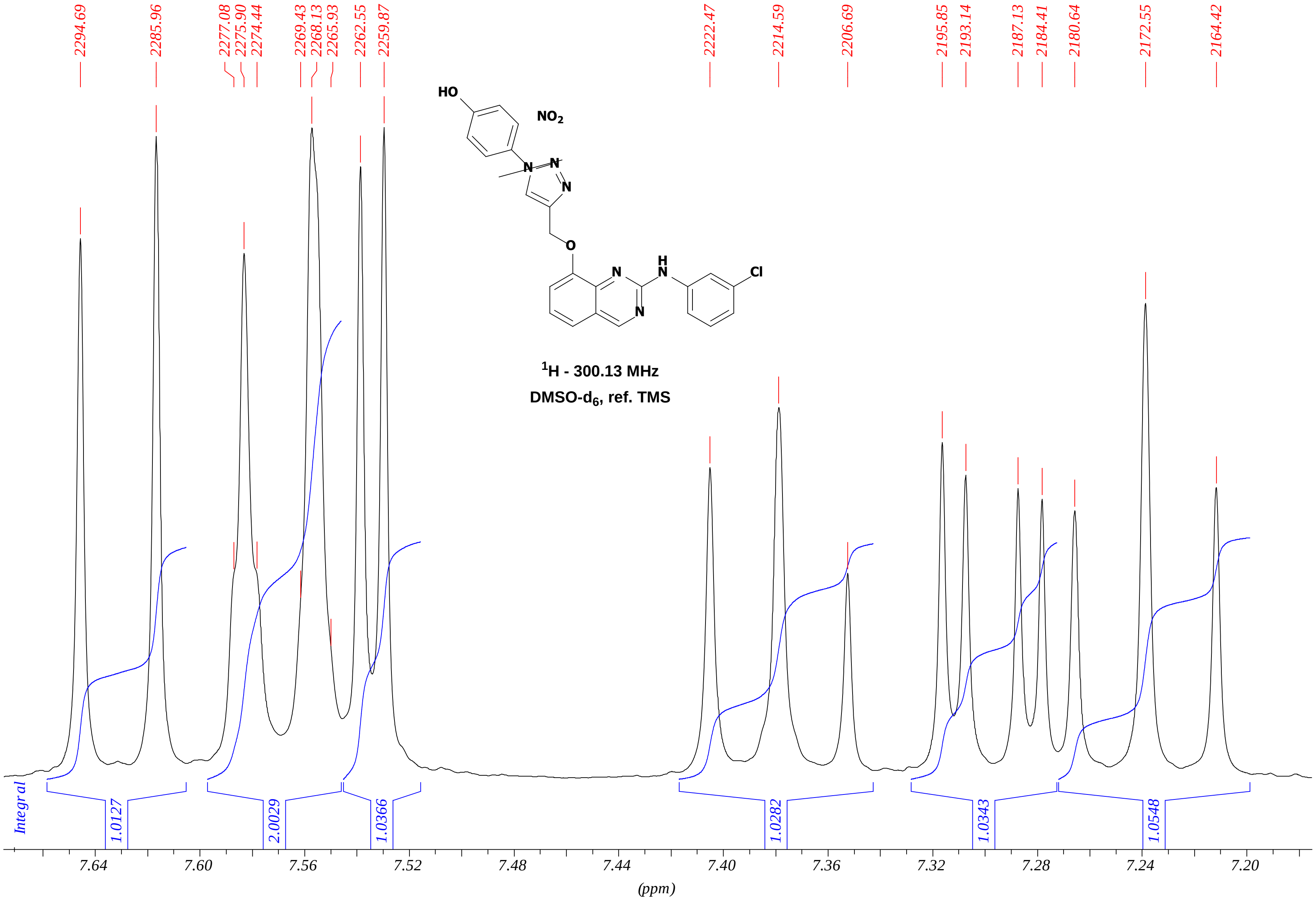
2294.69
2285.96
2277.08
2275.90
2274.44
2269.43
2268.13
2265.93
2262.55
2259.87
2222.47
2214.59
2206.69
2195.85
2193.14
2187.13
2184.41
2180.64
2172.55
2164.42
HO
NO2
N
N
N
O
N
H
N
Cl
N
1H - 300.13 MHz
DMSO-d6, ref. TMS
Integral
1.0127
2.0029
1.0366
1.0282
1.0343
1.0548
7.64
7.60
7.56
7.52
7.48
7.44
7.40
7.36
7.32
7.28
7.24
7.20
(ppm)

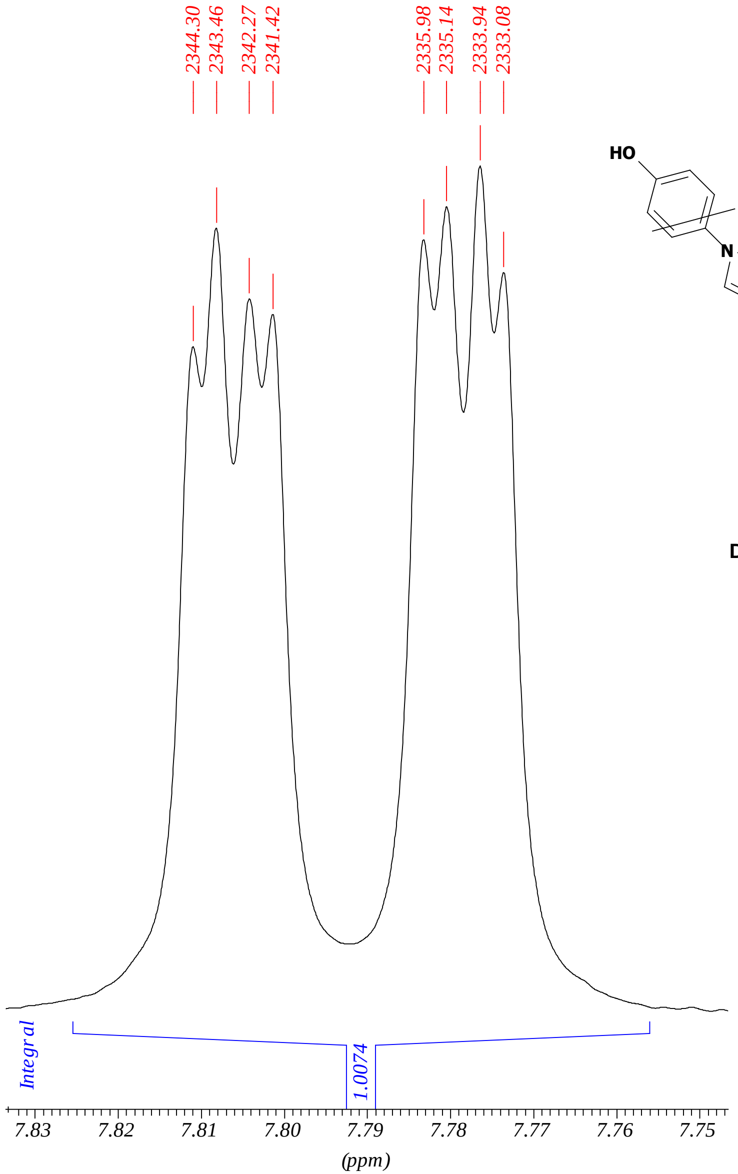

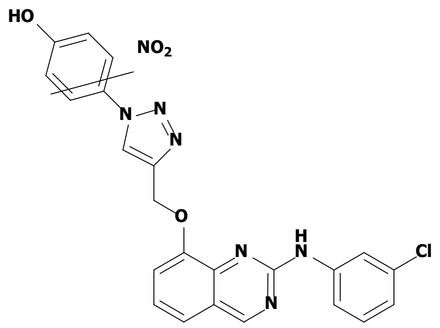

^{1}H - 300.13 MHz

DMSO-d_6, ref. TMS

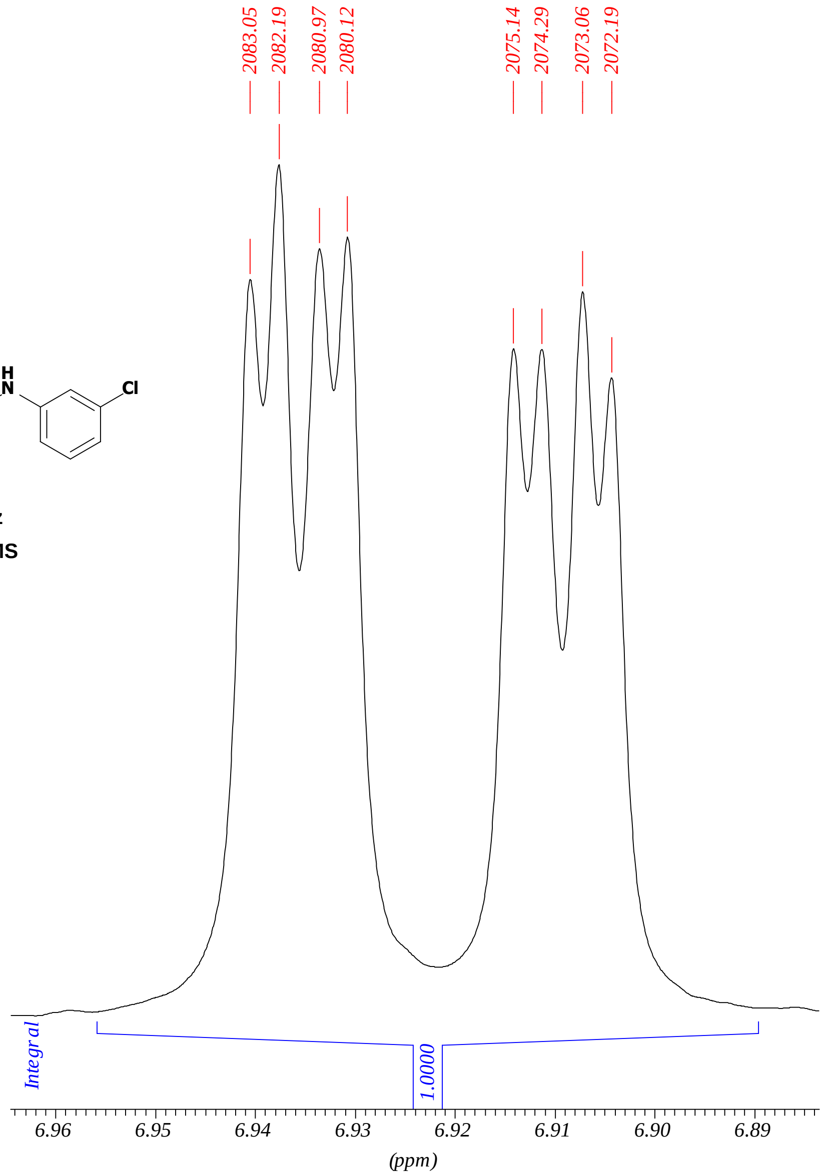

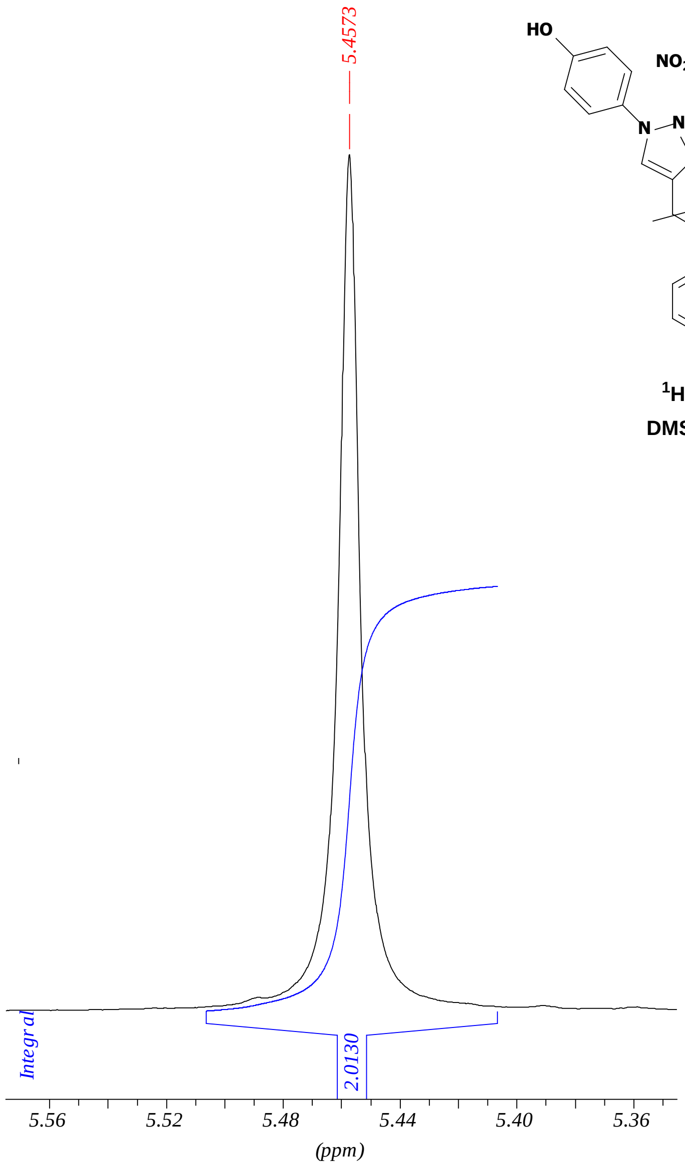

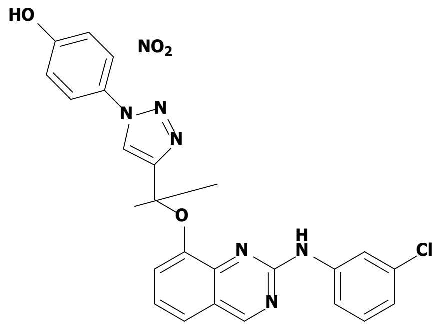

^{1}H - 300.13 MHz
DMSO-d_6, ref. TMS

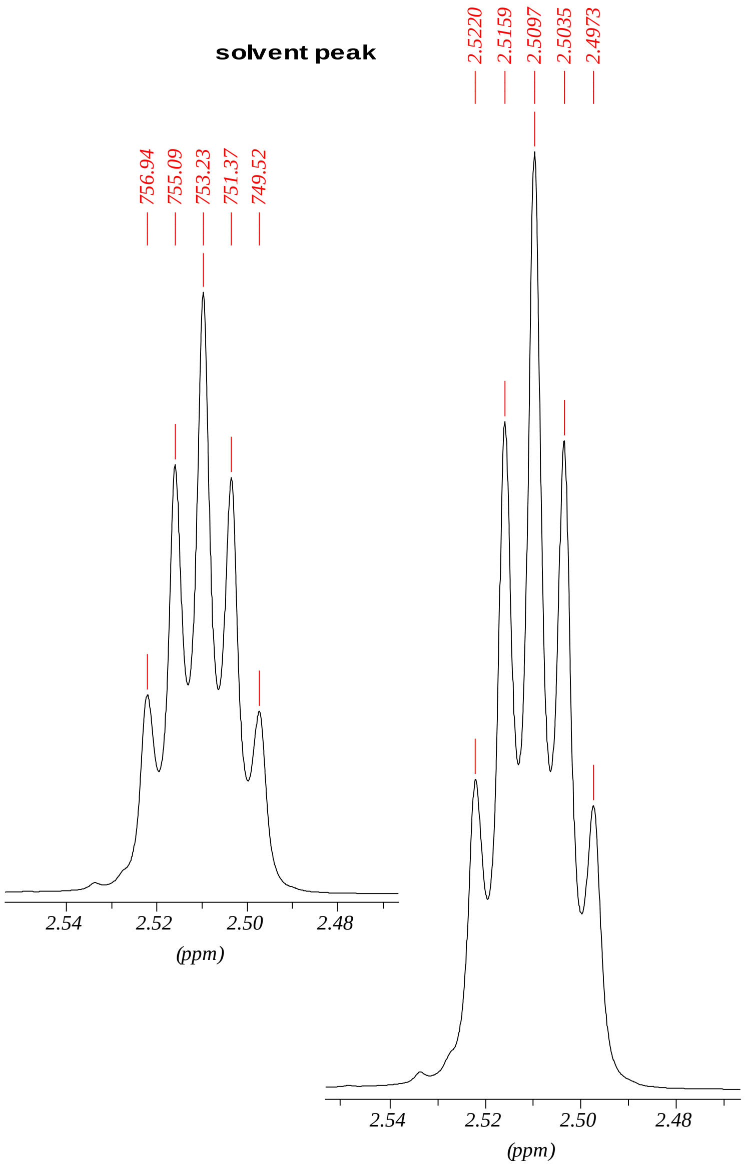

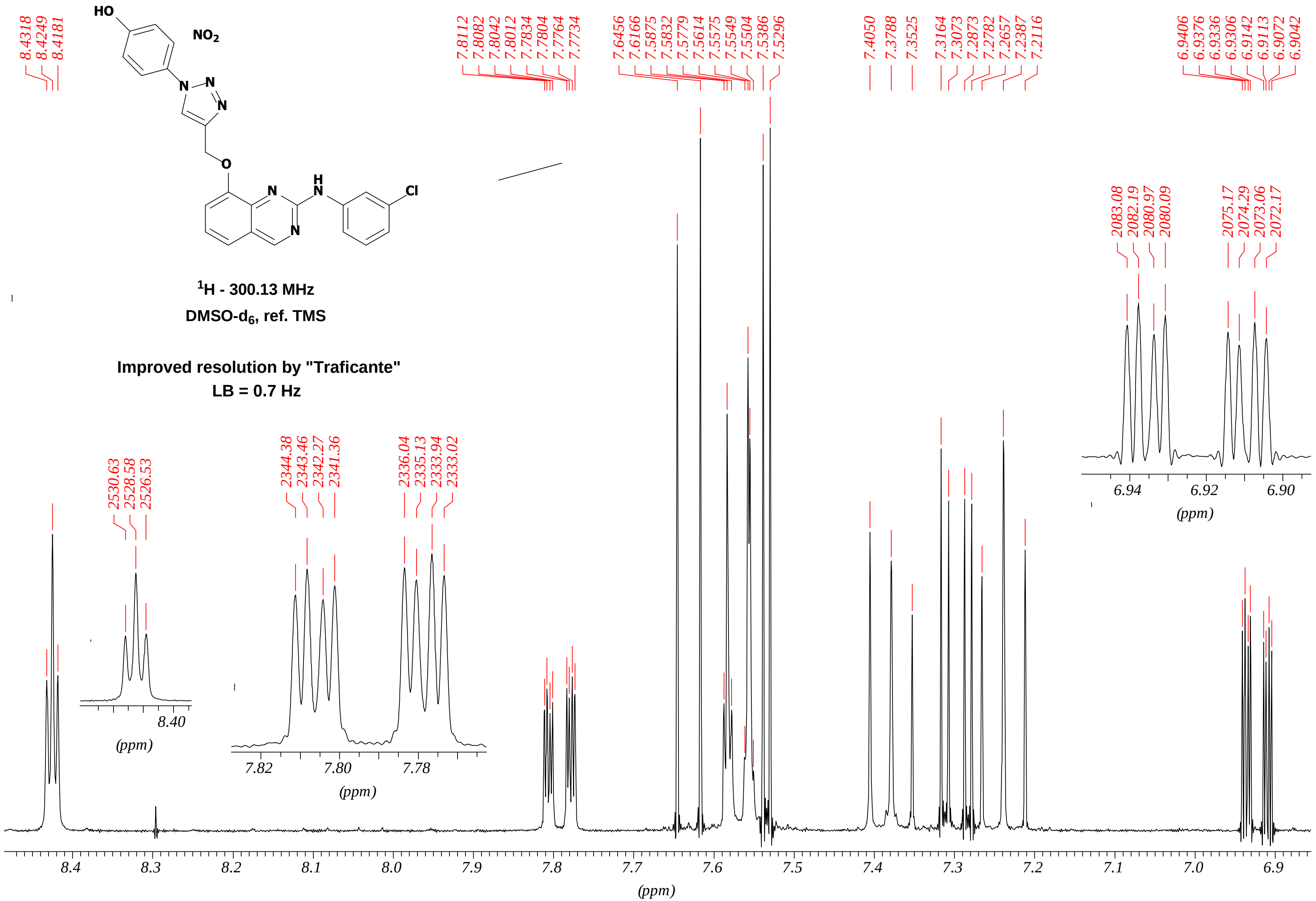
HO
NO2
N
N
N
O
N
N
H
N
Cl
1H - 300.13 MHz
DMSO-d6, ref. TMS
Improved resolution by "Traficante"
LB = 0.7 Hz
8.4318
8.4249
8.4181
7.8112
7.8082
7.8042
7.8012
7.7834
7.7804
7.7764
7.7734
7.6456
7.6166
7.5875
7.5832
7.5779
7.5614
7.5575
7.5549
7.5504
7.5386
7.5296
7.4050
7.3788
7.3525
7.3164
7.3073
7.2873
7.2782
7.2657
7.2387
7.2116
6.9406
6.9376
6.9336
6.9306
6.9142
6.9113
6.9072
6.9042
2530.63
2528.58
2526.53
2344.38
2343.46
2342.27
2341.36
2336.04
2335.13
2333.94
2333.02
2083.08
2082.19
2080.97
2080.09
2075.17
2074.29
2073.06
2072.17
(ppm)

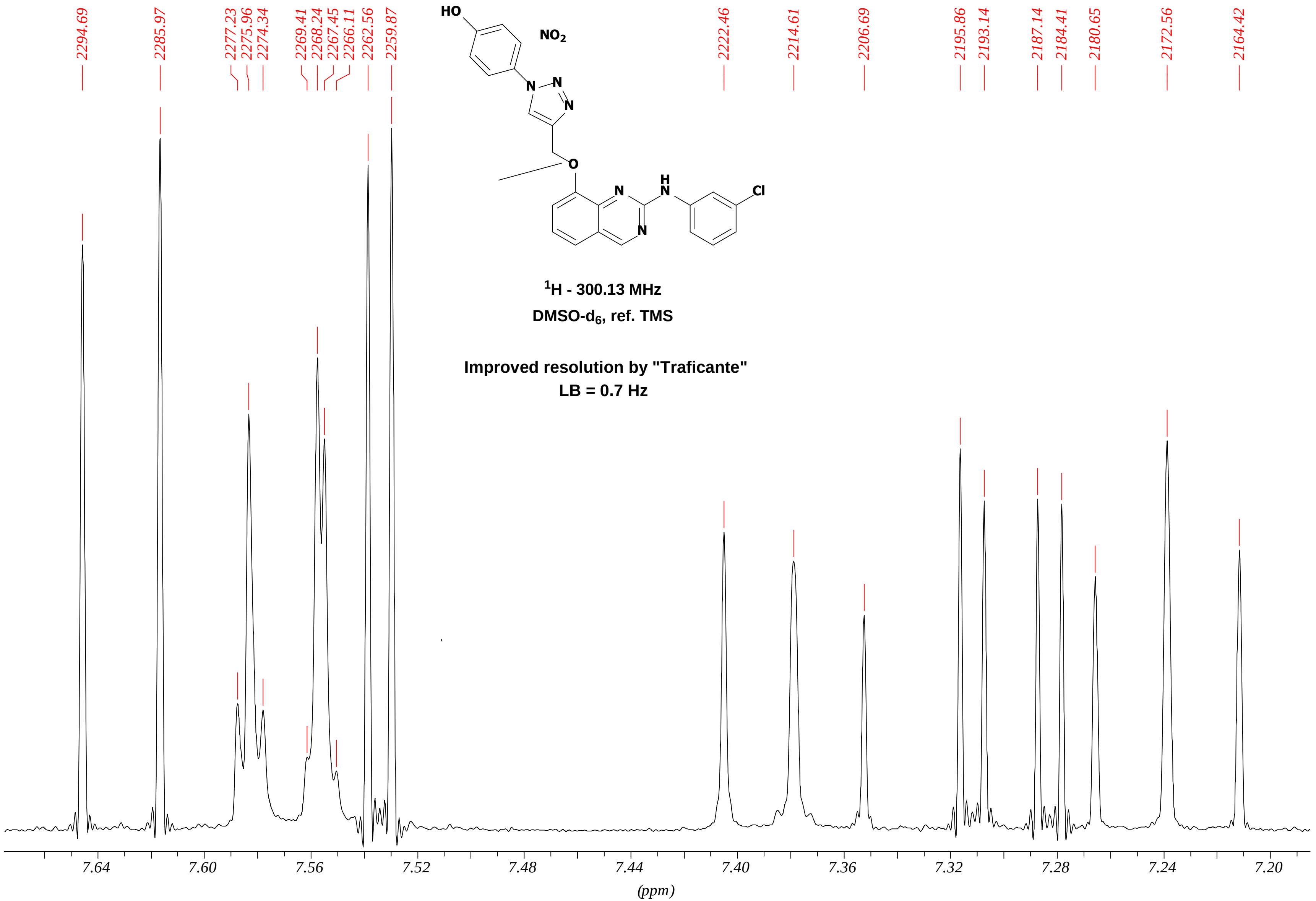

2294.69
2285.97
2277.23
2275.96
2274.34
2269.41
2268.24
2267.45
2266.11
2262.56
2259.87
2222.46
2214.61
2206.69
2195.86
2193.14
2187.14
2184.41
2180.65
2172.56
2164.42
HO
NO_2
N
N
N
O
N
H
N
N
Cl
^1H - 300.13 MHz
DMSO-d_6, ref. TMS
Improved resolution by "Traficante"
LB = 0.7 Hz
7.64
7.60
7.56
7.52
7.48
7.44
7.40
7.36
7.32
7.28
7.24
7.20
(ppm)

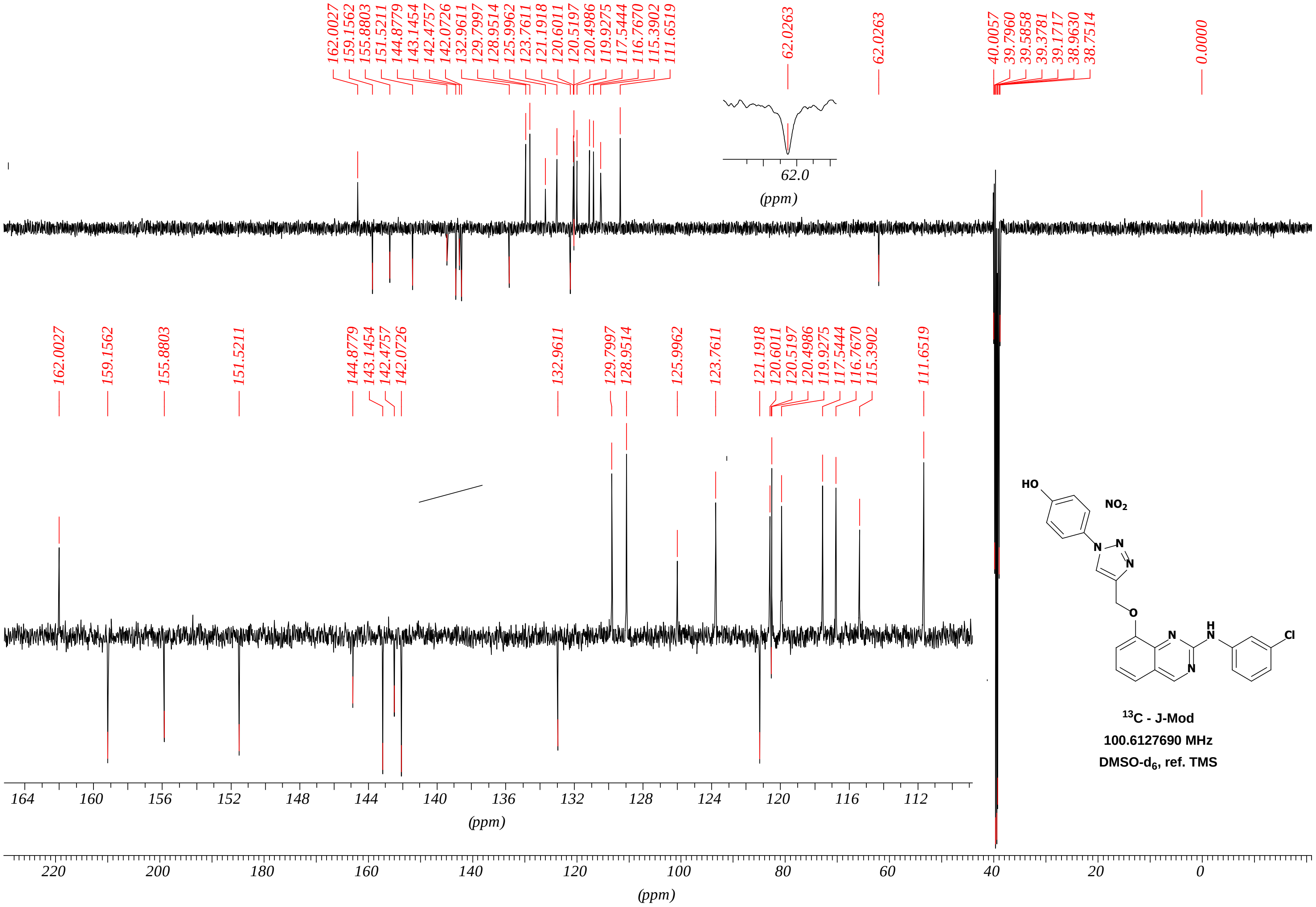
162.0027
159.1562
155.8803
151.5211
144.8779
143.1454
142.4757
142.0726
132.9611
129.7997
128.9514
125.9962
123.7611
121.1918
120.6011
120.5197
120.4986
119.9275
117.5444
116.7670
115.3902
111.6519
62.0263
62.0
(ppm)
40.0057
39.7960
39.5858
39.3781
39.1717
38.9630
38.7514
0.0000
HO
NO2
N
N
N
O
N
N
H
N
Cl
13C - J-Mod
100.6127690 MHz
DMSO-d6, ref. TMS
164 160 156 152 148 144 140 136 132 128 124 120 116 112
(ppm)
220 200 180 160 140 120 100 80 60 40 20 0
(ppm)

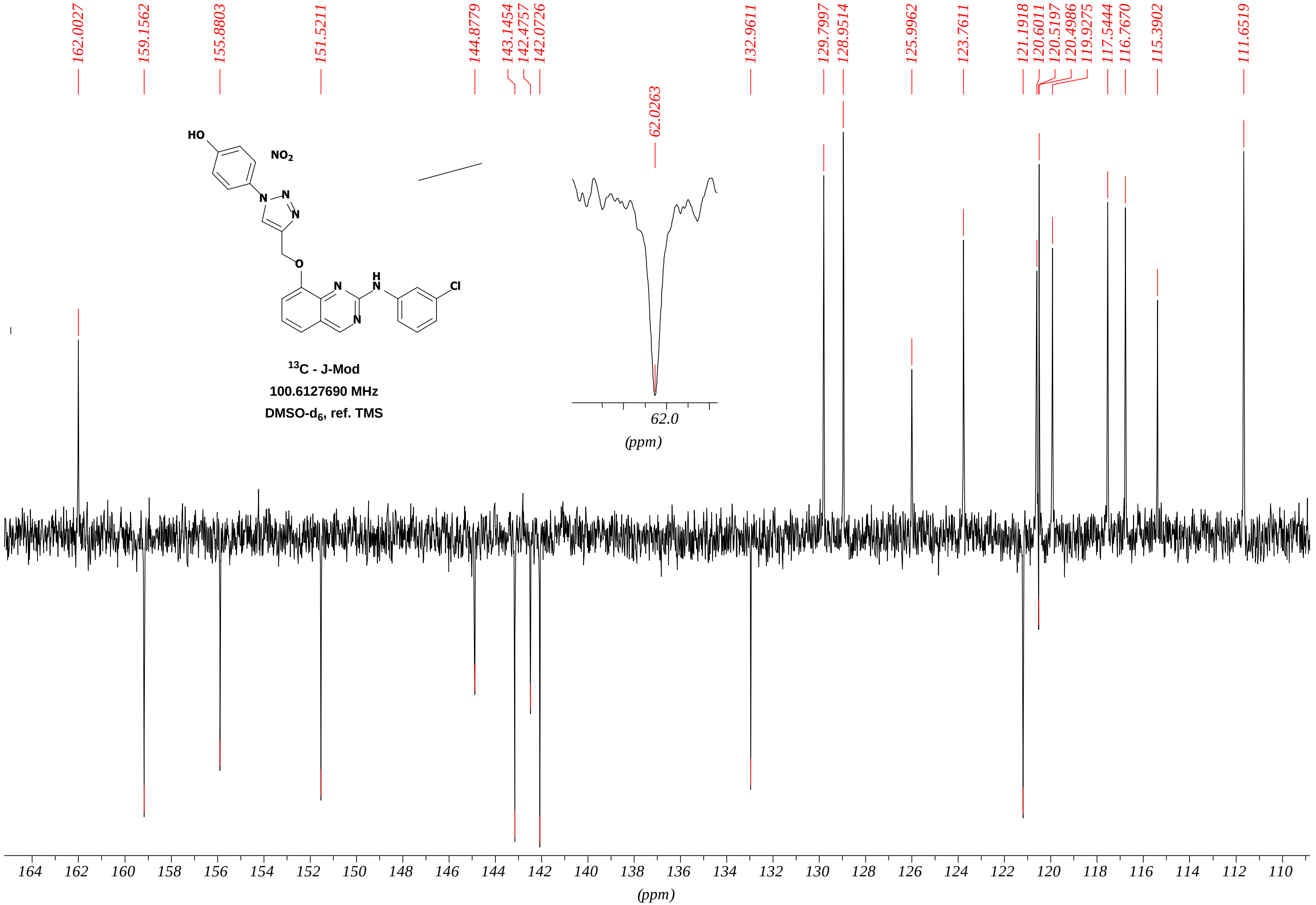
162.0027
159.1562
155.8803
151.5211
144.8779
143.1454
142.4757
142.0726
62.0263
132.9611
129.7997
128.9514
125.9962
123.7611
121.1918
120.6011
120.5197
120.4986
119.9275
117.5444
116.7670
115.3902
111.6519
HO
NO_2
N
N
N
O
N
N
H
N
Cl
^{13}C - J-Mod
100.6127690 MHz
DMSO-d_6, ref. TMS
62.0
(ppm)
164 162 160 158 156 154 152 150 148 146 144 142 140 138 136 134 132 130 128 126 124 122 120 118 116 114 112 110
(ppm)

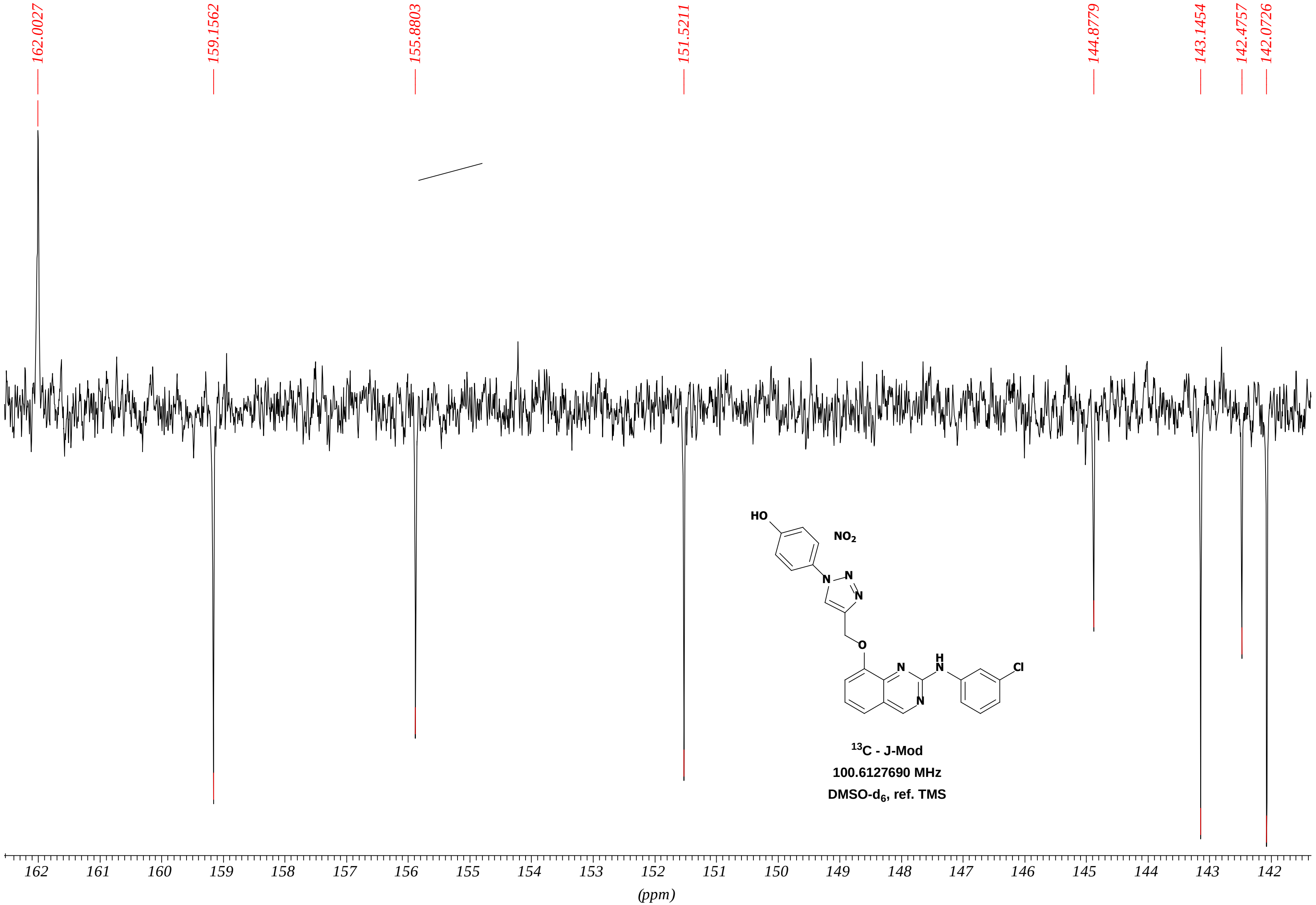
162.0027
159.1562
155.8803
151.5211
144.8779
143.1454
142.4757
142.0726
HO
NO_2
N
N
N
O
N
H
N
N
Cl
^{13}C - J-Mod
100.6127690 MHz
DMSO-d_6, ref. TMS
162
161
160
159
158
157
156
155
154
153
152
151
150
149
148
147
146
145
144
143
142
(ppm)

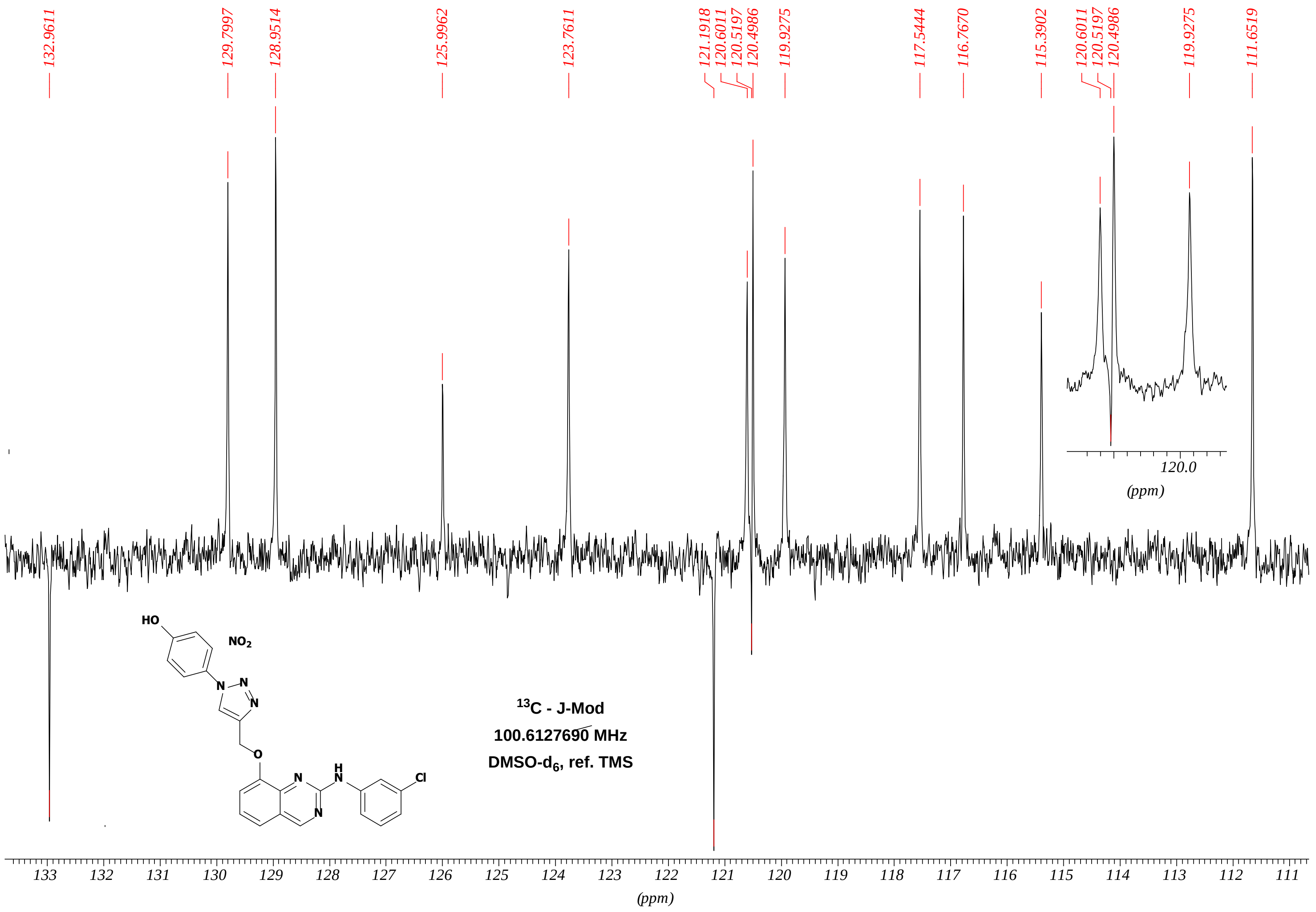
132.9611
129.7997
128.9514
125.9962
123.7611
121.1918
120.6011
120.5197
120.4986
119.9275
117.5444
116.7670
115.3902
111.6519
120.0
(ppm)
^{13}C - J-Mod
100.6127690 MHz
DMSO-d6, ref. TMS
HO
NO2
N
N
N
O
N
N
H
Cl
133
132
131
130
129
128
127
126
125
124
123
122
121
120
119
118
117
116
115
114
113
112
111

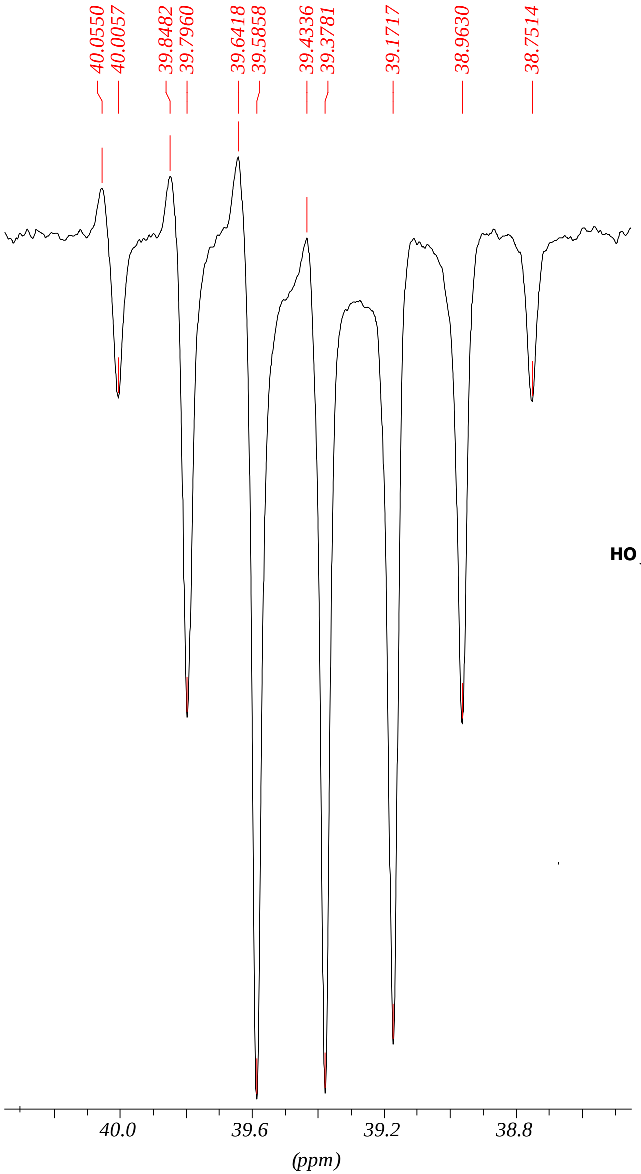

solvent peak

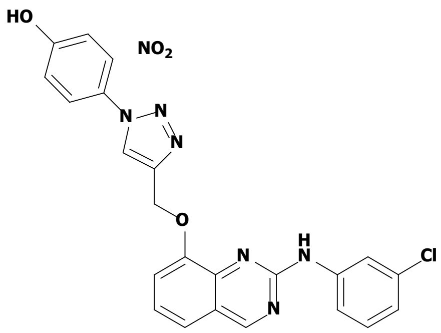

^{13}C - J-Mod
100.6127690 MHz
DMSO-d_6, ref. TMS

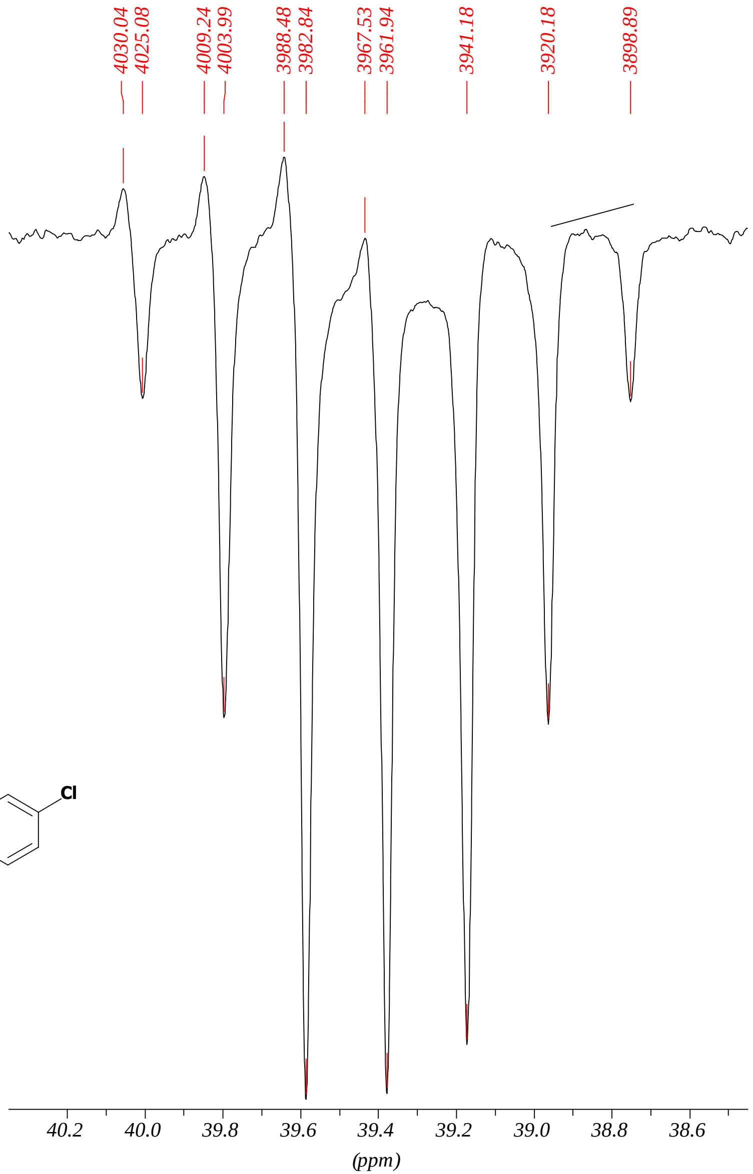

***N*-(3-Chlorophenyl)-8-((1-(4-bromo-2-nitrophenyl)-1*H*-1,2,3-triazol-4-yl)methoxy)quinazolin-2-amine (12d):**

Pages S64-S75

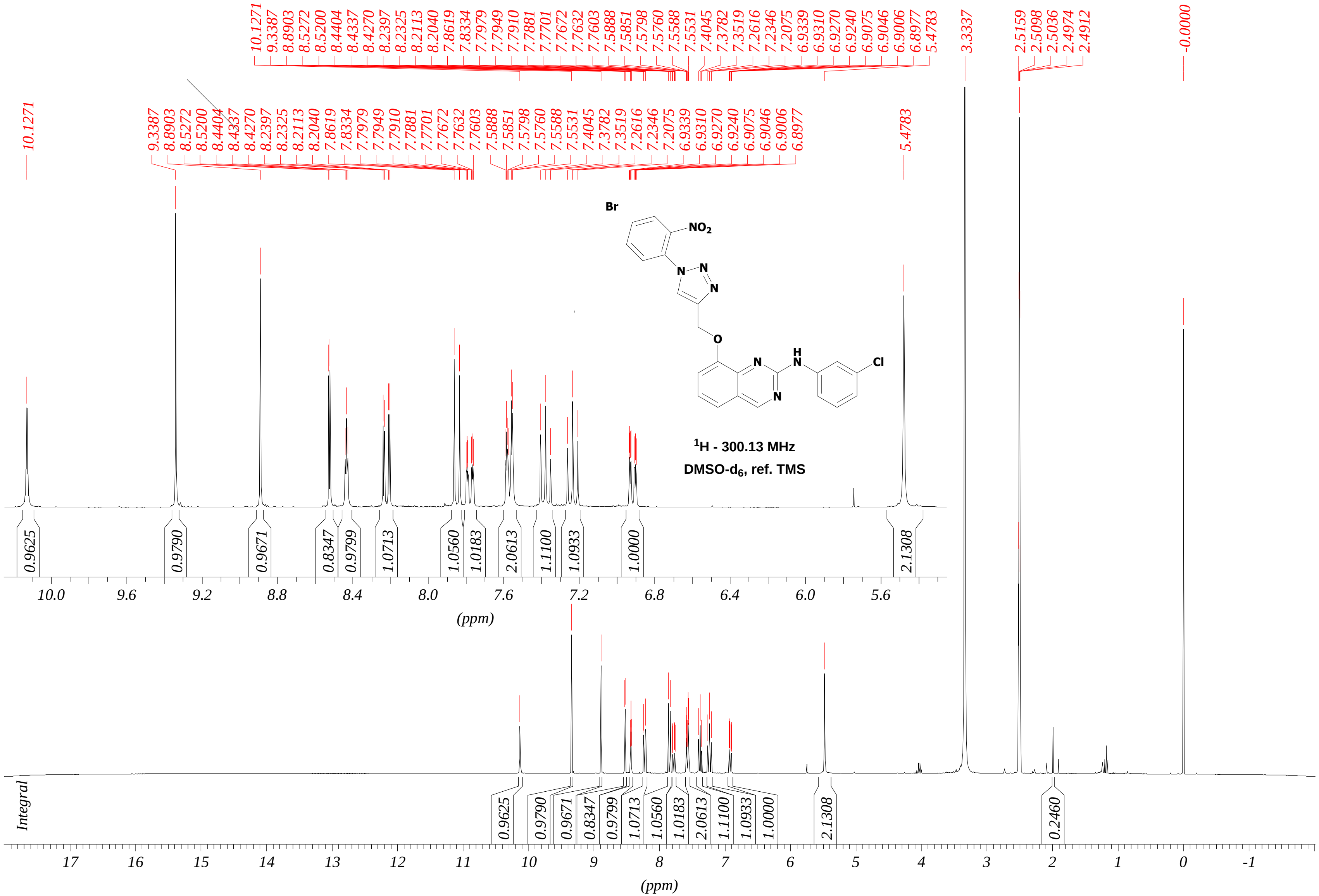

Br
NO2
N
N
N
O
N
H
N
Cl
N
1H - 300.13 MHz
DMSO-d6, ref. TMS
(ppm)
Integral

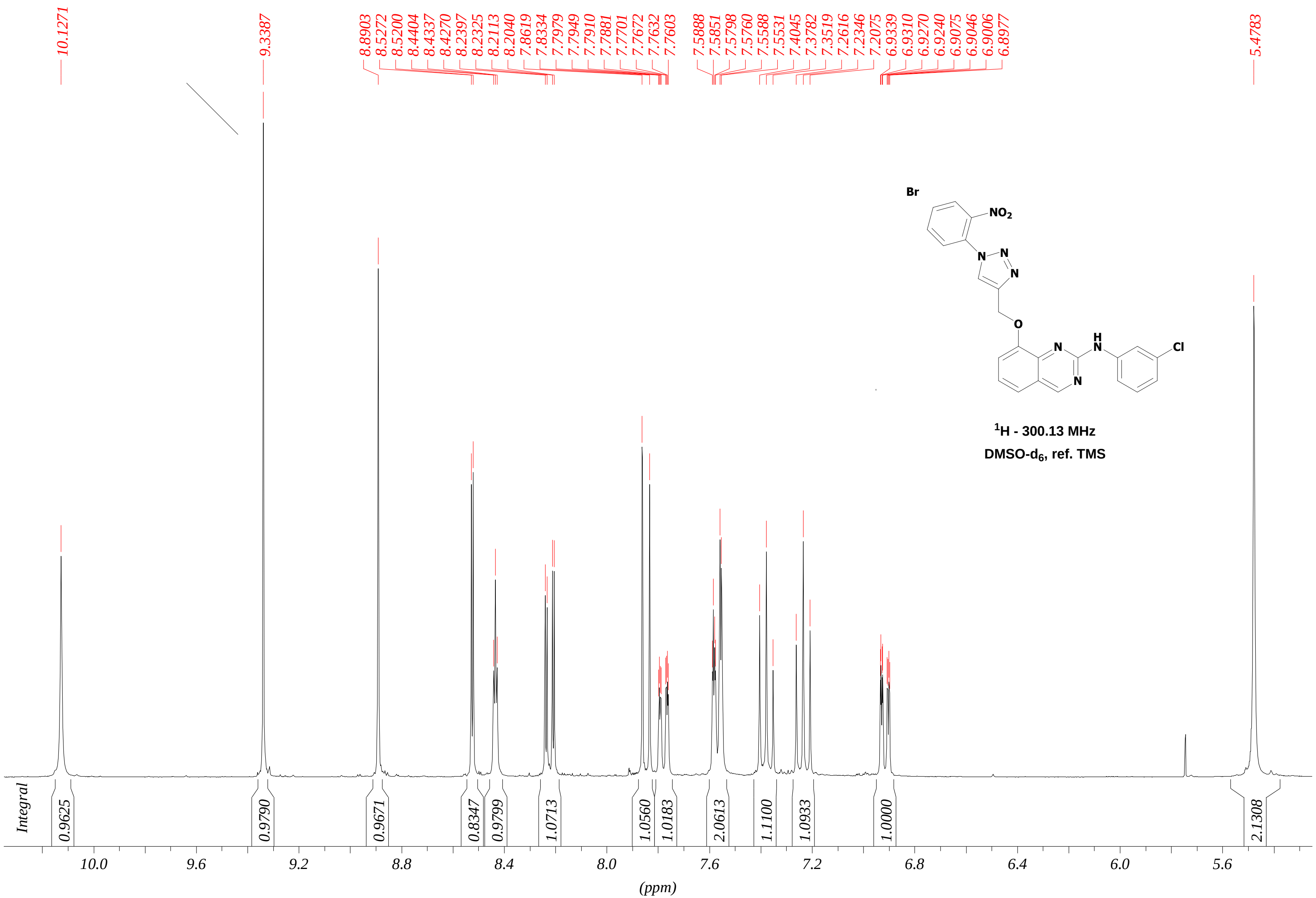
10.1271
9.3387
8.8903
8.5272
8.5200
8.4404
8.4337
8.4270
8.2397
8.2325
8.2113
8.2040
7.8619
7.8334
7.7979
7.7949
7.7910
7.7881
7.7701
7.7672
7.7632
7.7603
7.5888
7.5851
7.5798
7.5760
7.5588
7.5531
7.4045
7.3782
7.3519
7.2616
7.2346
7.2075
6.9339
6.9310
6.9270
6.9240
6.9075
6.9046
6.9006
6.8977
5.4783
Br
NO2
N
N
N
O
N
H
N
Cl
N
1H - 300.13 MHz
DMSO-d6, ref. TMS
Integral
0.9625
0.9790
0.9671
0.8347
0.9799
1.0713
1.0560
1.0183
2.0613
1.1100
1.0933
1.0000
2.1308
10.0
9.6
9.2
8.8
8.4
8.0
7.6
7.2
6.8
6.4
6.0
5.6
(ppm)

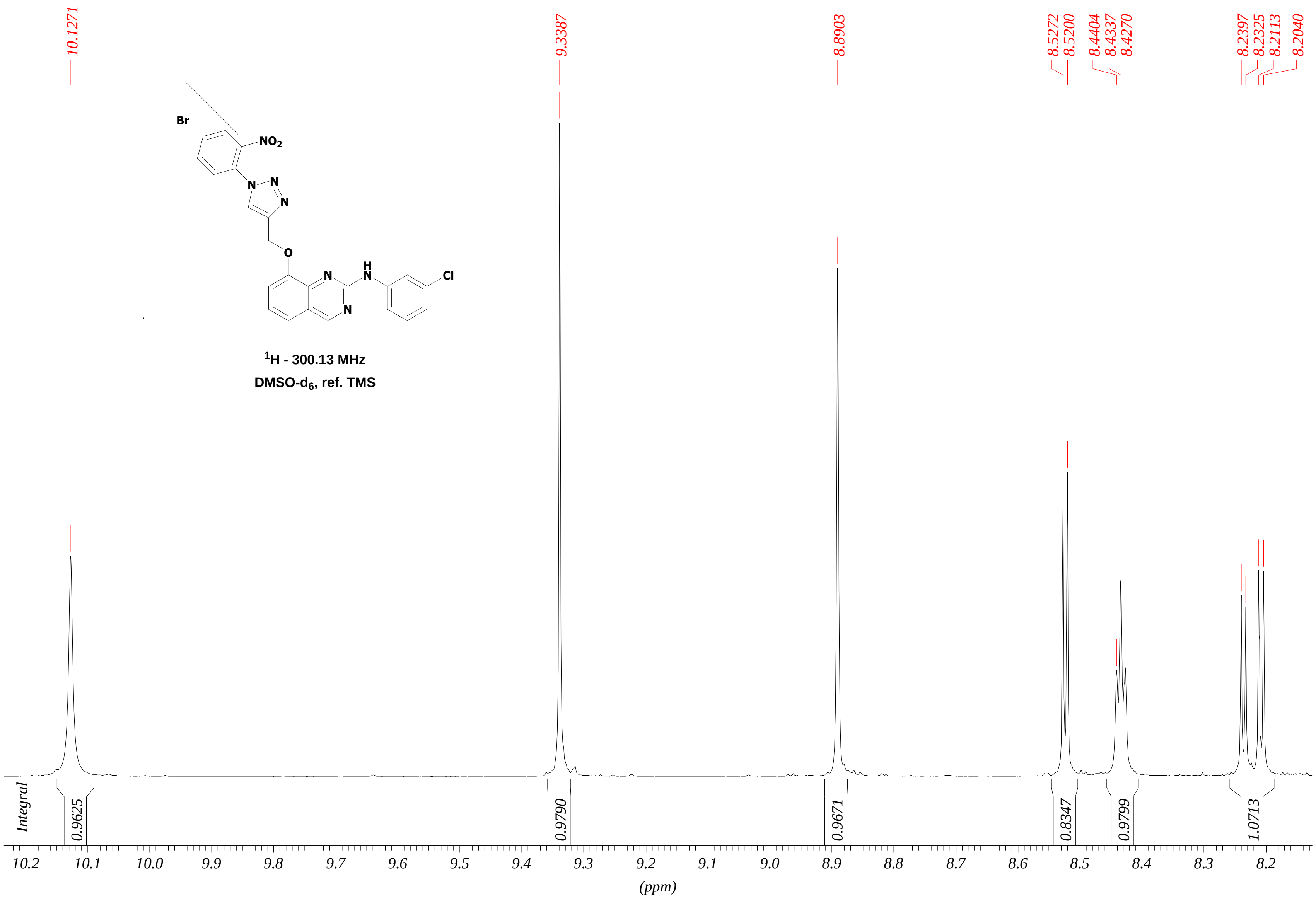
10.1271
9.3387
8.8903
8.5272
8.5200
8.4404
8.4337
8.4270
8.2397
8.2325
8.2113
8.2040
Br
NO2
N
N
N
O
N
H
N
Cl
N
^{1}H - 300.13 MHz
DMSO-d$_6$, ref. TMS
Integral
0.9625
0.9790
0.9671
0.8347
0.9799
1.0713
10.2 10.1 10.0 9.9 9.8 9.7 9.6 9.5 9.4 9.3 9.2 9.1 9.0 8.9 8.8 8.7 8.6 8.5 8.4 8.3 8.2
(ppm)

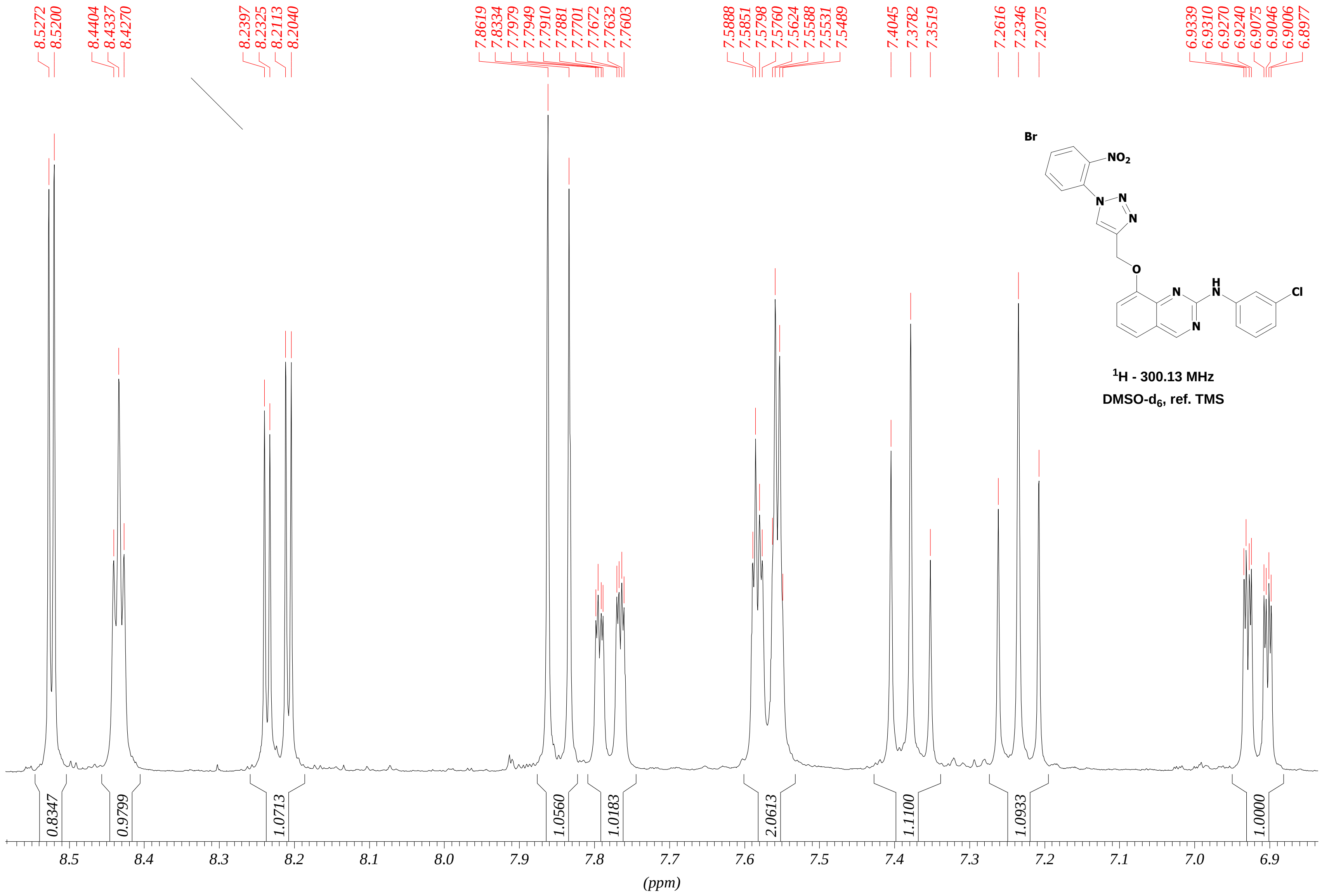

8.5272
8.5200
8.4404
8.4337
8.4270
8.2397
8.2325
8.2113
8.2040
7.8619
7.8334
7.7979
7.7949
7.7910
7.7881
7.7701
7.7672
7.7632
7.7603
7.5888
7.5851
7.5798
7.5760
7.5624
7.5588
7.5531
7.5489
7.4045
7.3782
7.3519
7.2616
7.2346
7.2075
6.9339
6.9310
6.9270
6.9240
6.9075
6.9046
6.9006
6.8977
Br
NO_2
N
N
N
O
N
H
N
Cl
N
^{1}H - 300.13 MHz
DMSO-d_6, ref. TMS
0.8347
0.9799
1.0713
1.0560
1.0183
2.0613
1.1100
1.0933
1.0000
8.5
8.4
8.3
8.2
8.1
8.0
7.9
7.8
7.7
7.6
7.5
7.4
7.3
7.2
7.1
7.0
6.9
(ppm)

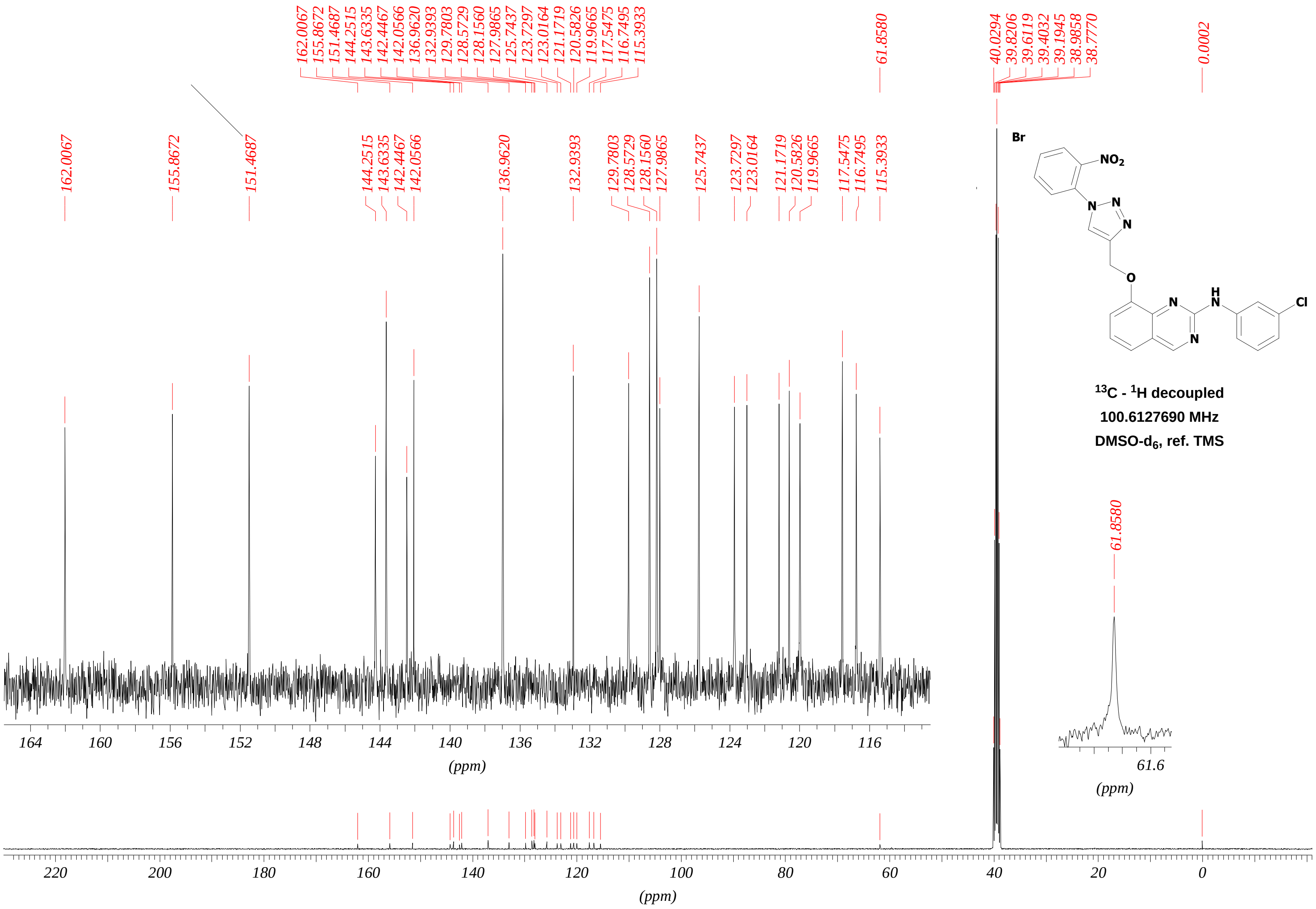

162.0067
155.8672
151.4687
144.2515
143.6335
142.4467
142.0566
136.9620
132.9393
129.7803
128.5729
128.1560
127.9865
125.7437
123.7297
123.0164
121.1719
120.5826
119.9665
117.5475
116.7495
115.3933
61.8580
40.0294
39.8206
39.6119
39.4032
39.1945
38.9858
38.7770
0.0002
Br
NO2
N
N
N
O
N
H
N
N
Cl
13C - 1H decoupled
100.6127690 MHz
DMSO-d6, ref. TMS
(ppm)

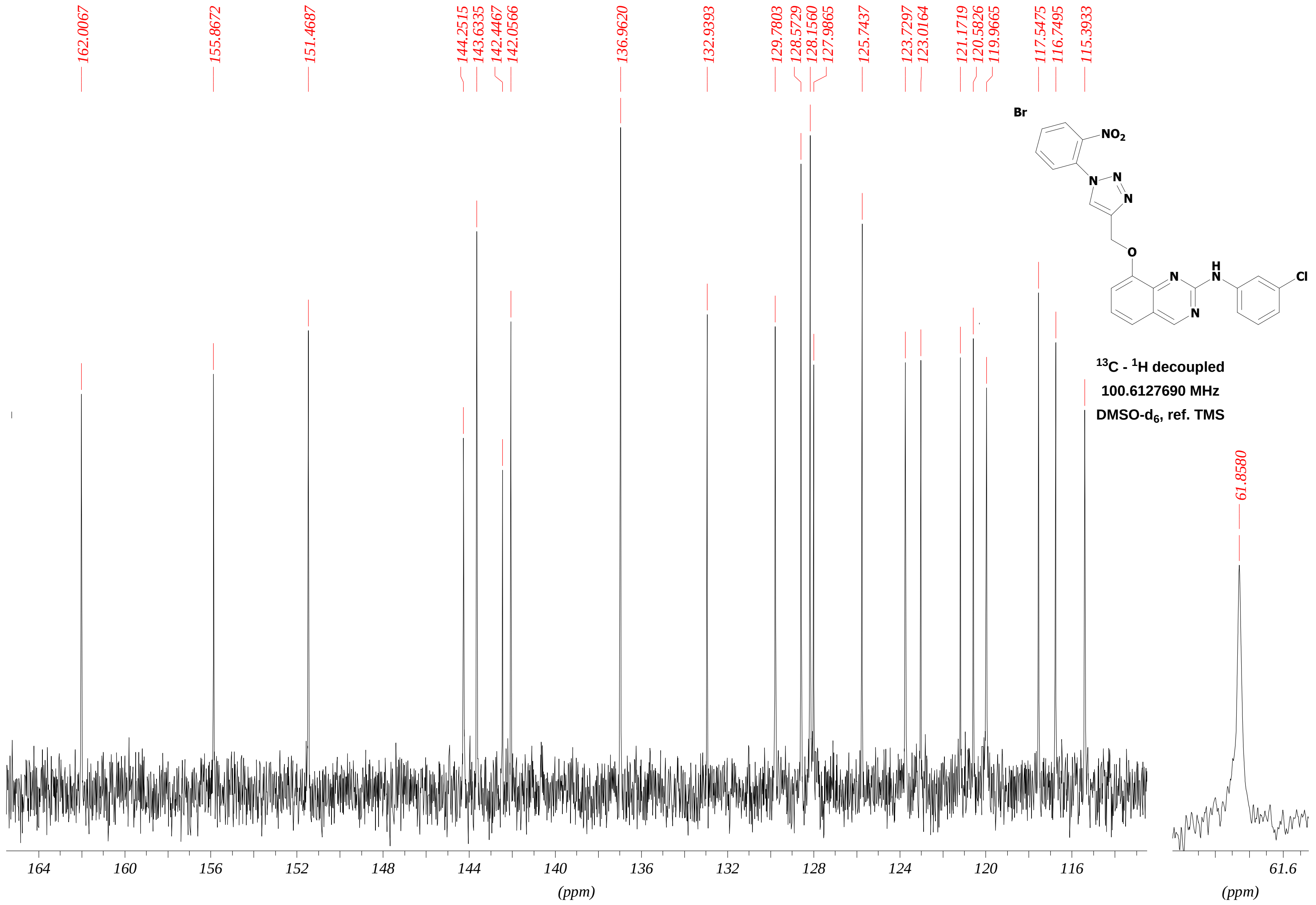
162.0067
155.8672
151.4687
144.2515
143.6335
142.4467
142.0566
136.9620
132.9393
129.7803
128.5729
128.1560
127.9865
125.7437
123.7297
123.0164
121.1719
120.5826
119.9665
117.5475
116.7495
115.3933
61.8580
Br
NO2
N
N
N
O
N
H
N
Cl
N
13C - 1H decoupled
100.6127690 MHz
DMSO-d6, ref. TMS
164
160
156
152
148
144
140
136
132
128
124
120
116
(ppm)
61.6
(ppm)

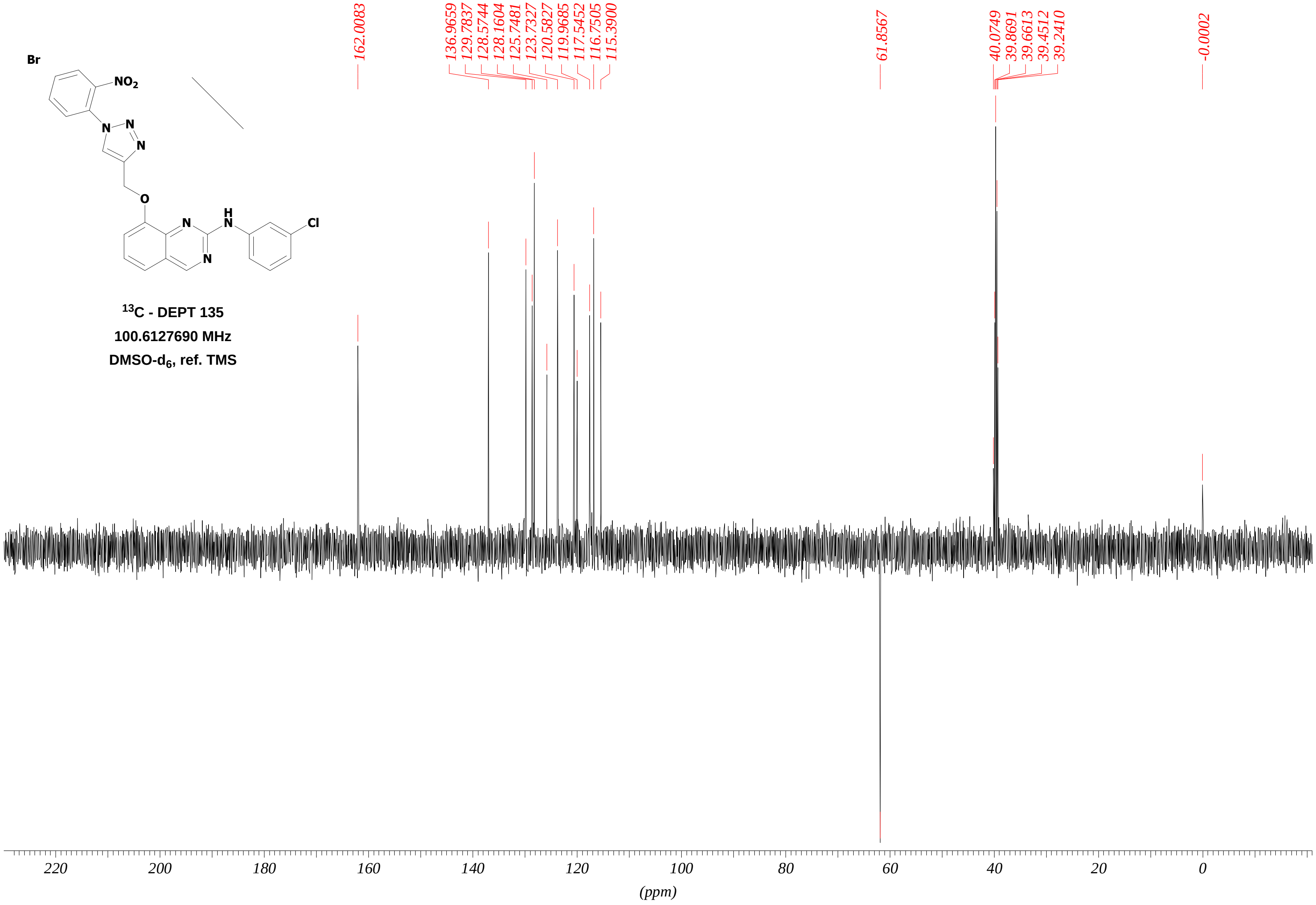

Br
NO2
N
N
N
O
N
H
N
Cl
N
13C - DEPT 135
100.6127690 MHz
DMSO-d6, ref. TMS
162.0083
136.9659
129.7837
128.5744
128.1604
125.7481
123.7327
120.5827
119.9685
117.5452
116.7505
115.3900
61.8567
40.0749
39.8691
39.6613
39.4512
39.2410
-0.0002
220
200
180
160
140
120
100
80
60
40
20
0
(ppm)

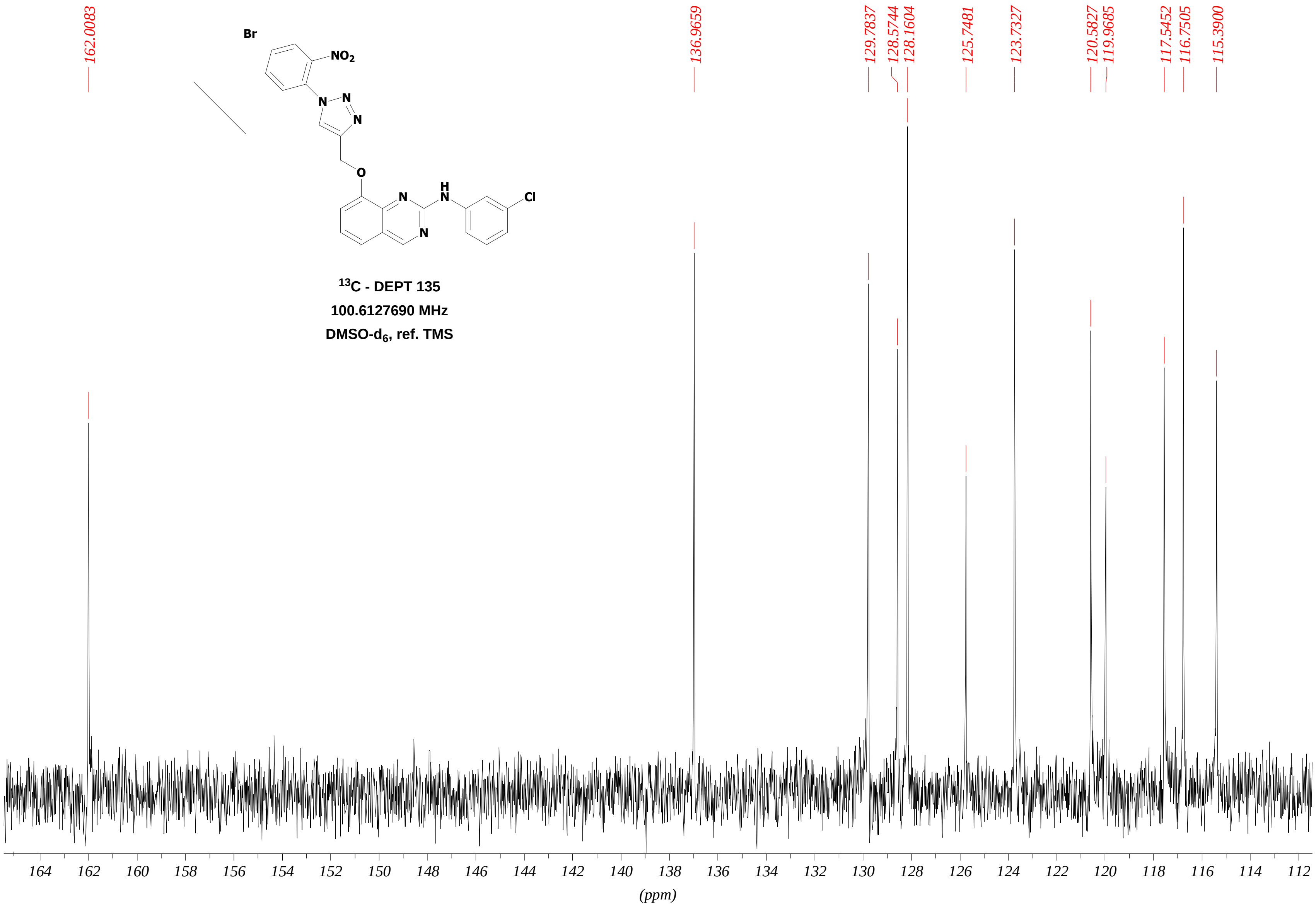

162.0083
136.9659
129.7837
128.5744
128.1604
125.7481
123.7327
120.5827
119.9685
117.5452
116.7505
115.3900
Br
NO2
N
N
N
O
N
N
H
N
Cl
13C - DEPT 135
100.6127690 MHz
DMSO-d6, ref. TMS
164
162
160
158
156
154
152
150
148
146
144
142
140
138
136
134
132
130
128
126
124
122
120
118
116
114
112
(ppm)

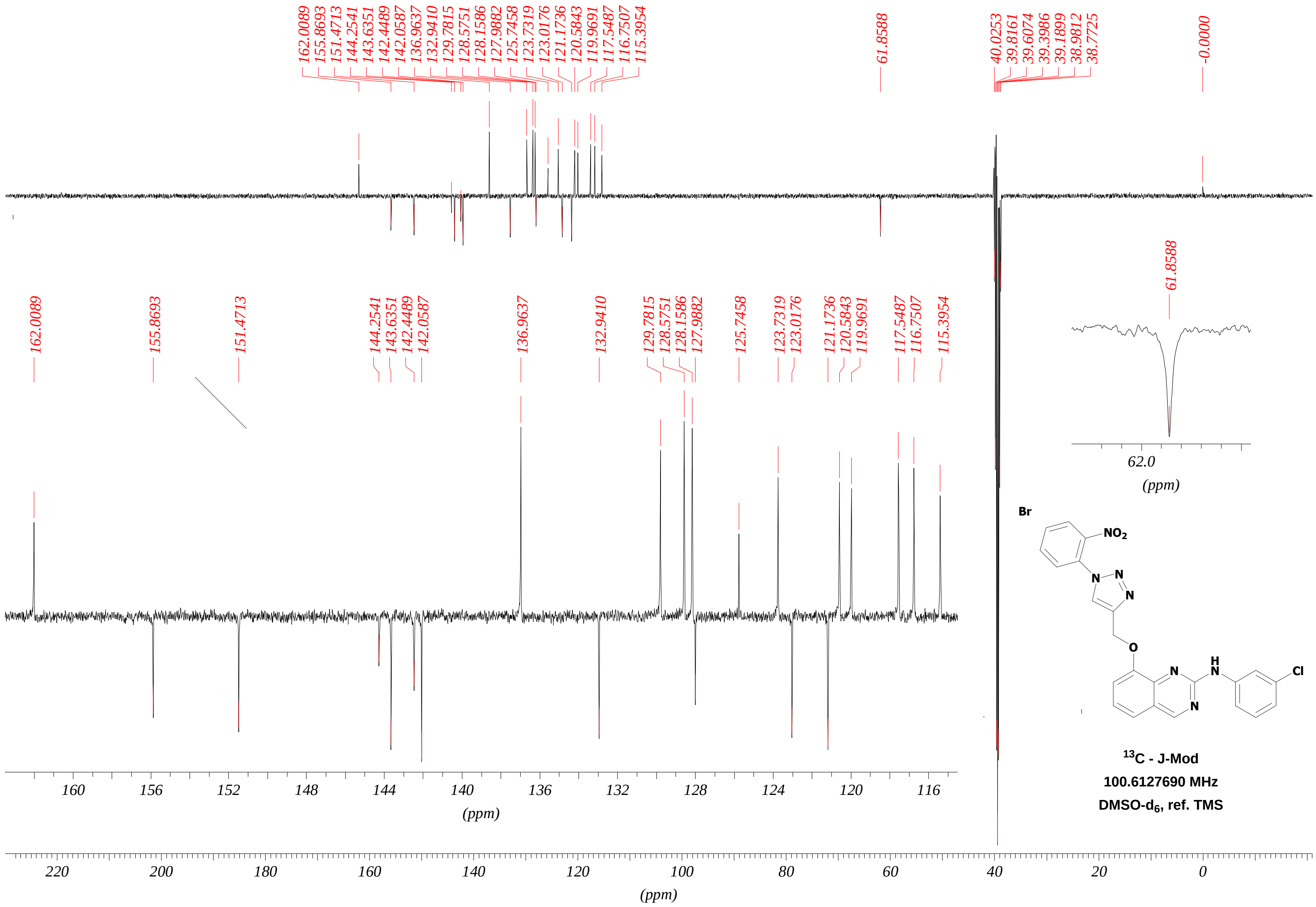

Br
NO_2
N
N
N
O
H
N
N
N
Cl
^{13}C - J-Mod
100.6127690 MHz
DMSO-d_6, ref. TMS

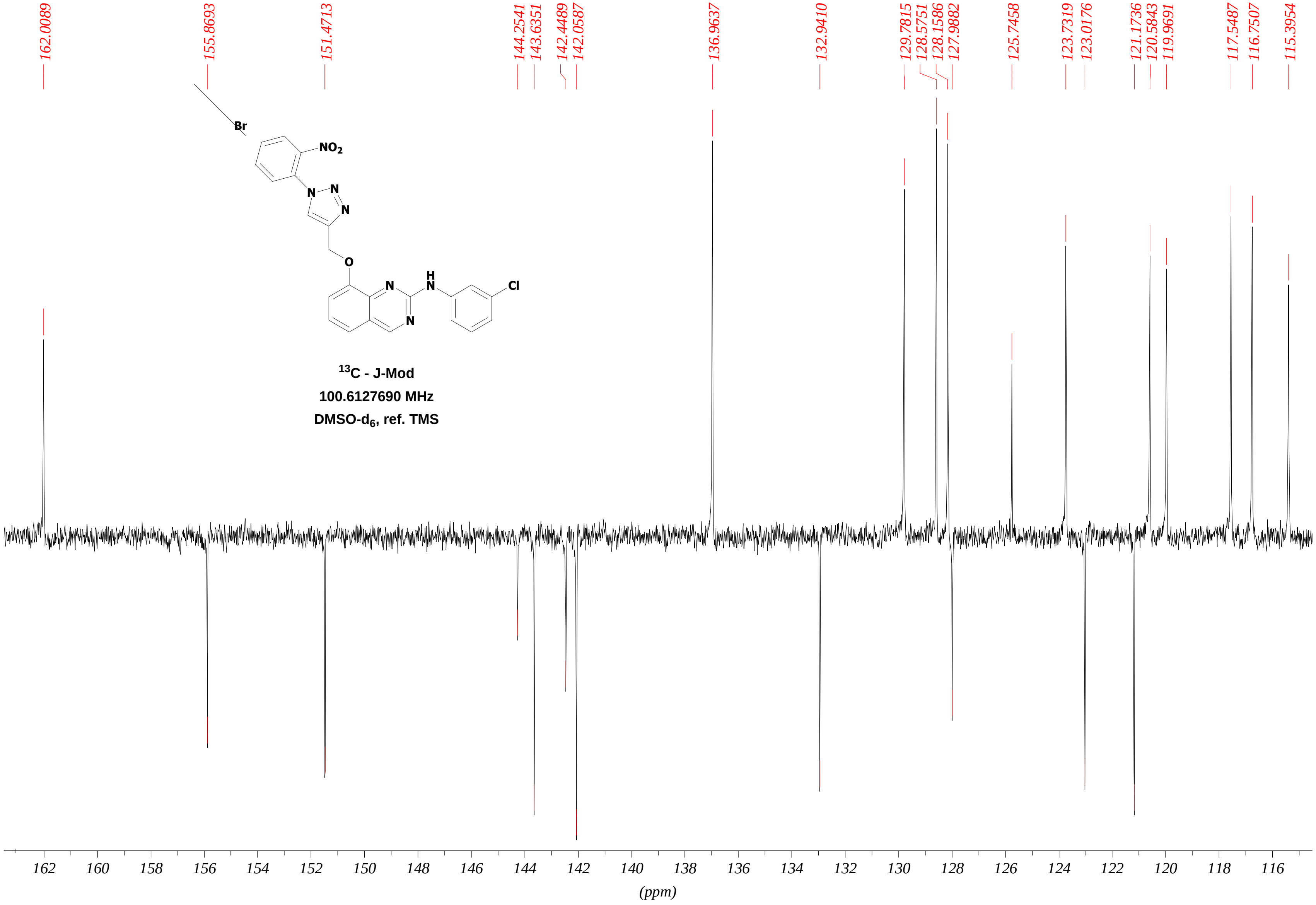
162.0089
155.8693
151.4713
144.2541
143.6351
142.4489
142.0587
136.9637
132.9410
129.7815
128.5751
128.1586
127.9882
125.7458
123.7319
123.0176
121.1736
120.5843
119.9691
117.5487
116.7507
115.3954
Br
NO2
N
N
N
O
N
H
N
Cl
N
13C - J-Mod
100.6127690 MHz
DMSO-d6, ref. TMS
162
160
158
156
154
152
150
148
146
144
142
140
138
136
134
132
130
128
126
124
122
120
118
116
(ppm)

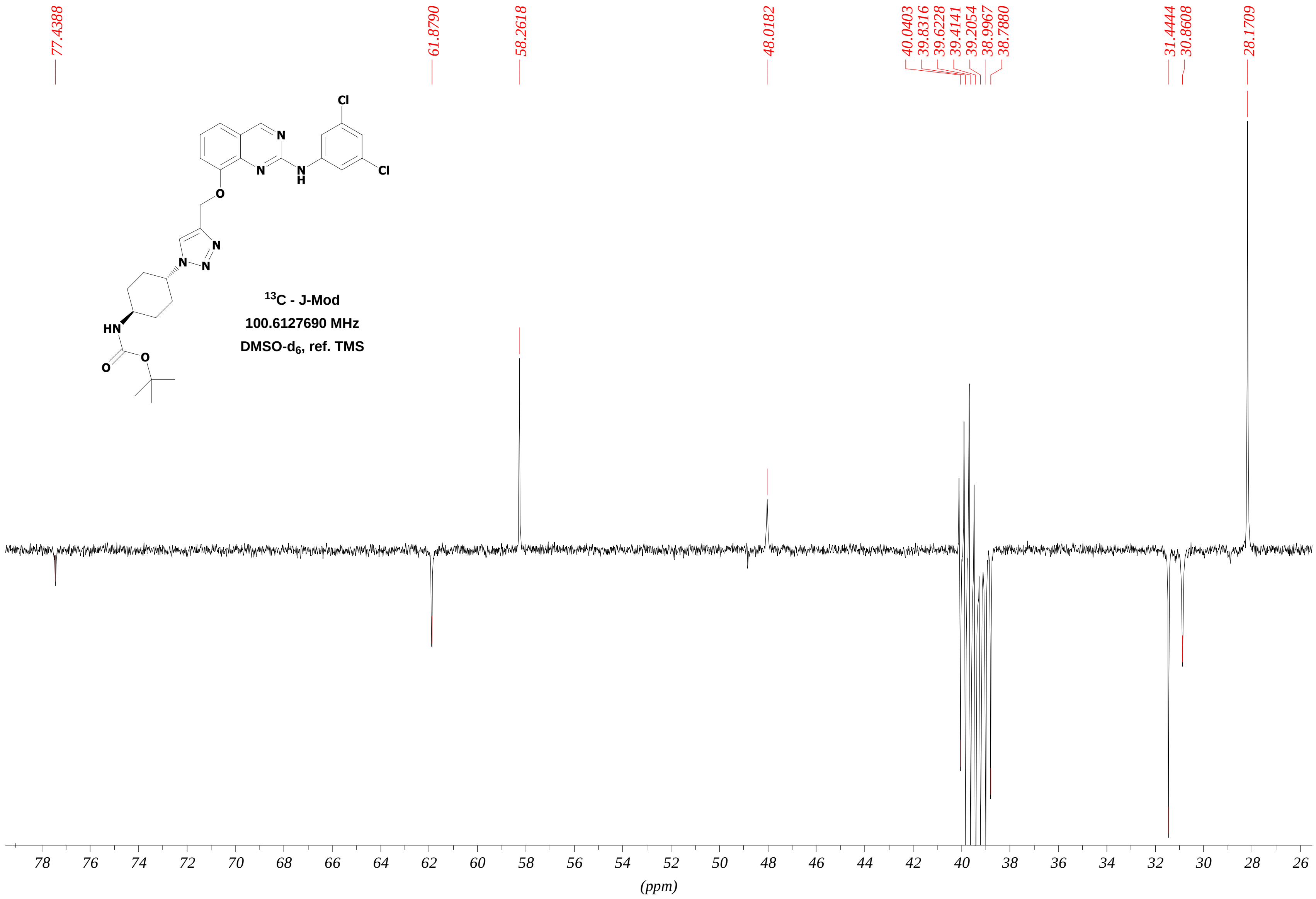

77.4388
61.8790
58.2618
48.0182
40.0403
39.8316
39.6228
39.4141
39.2054
38.9967
38.7880
31.4444
30.8608
28.1709
Cl
N
N
N
H
Cl
O
N
N
N
HN
O
O
13C - J-Mod
100.6127690 MHz
DMSO-d6, ref. TMS
78
76
74
72
70
68
66
64
62
60
58
56
54
52
50
48
46
44
42
40
38
36
34
32
30
28
26
(ppm)

4-(4-(((2-((3-Chlorophenyl)amino)quinazolin-8-yl)oxy)methyl)-1*H*-1,2,3-triazol-1-yl)-3-nitrobenzyl alcohol (12e):

Pages S76-S89

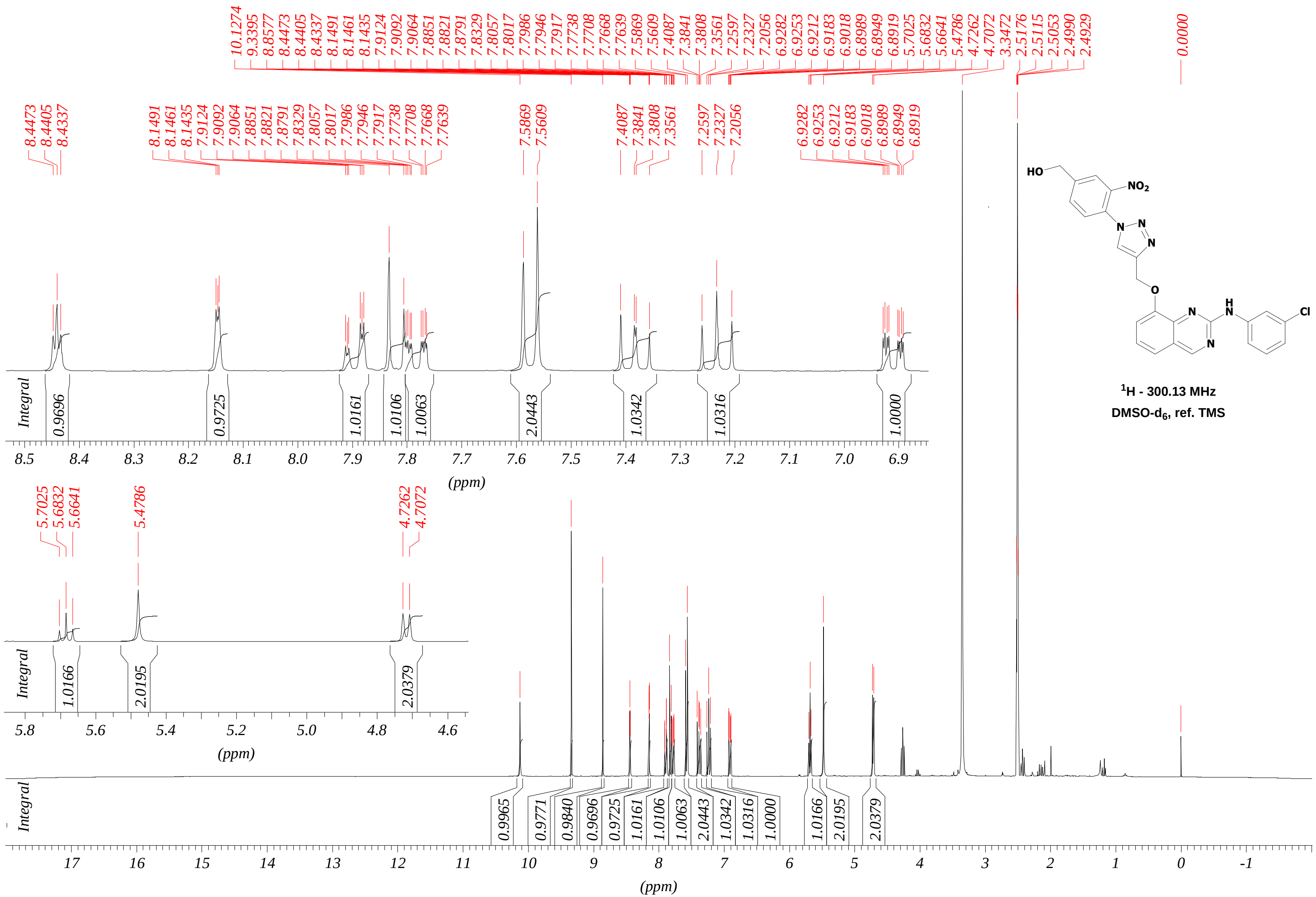
HO
NO2
N
N
N
O
N
N
H
N
Cl
1H - 300.13 MHz
DMSO-d6, ref. TMS
Integral
(ppm)

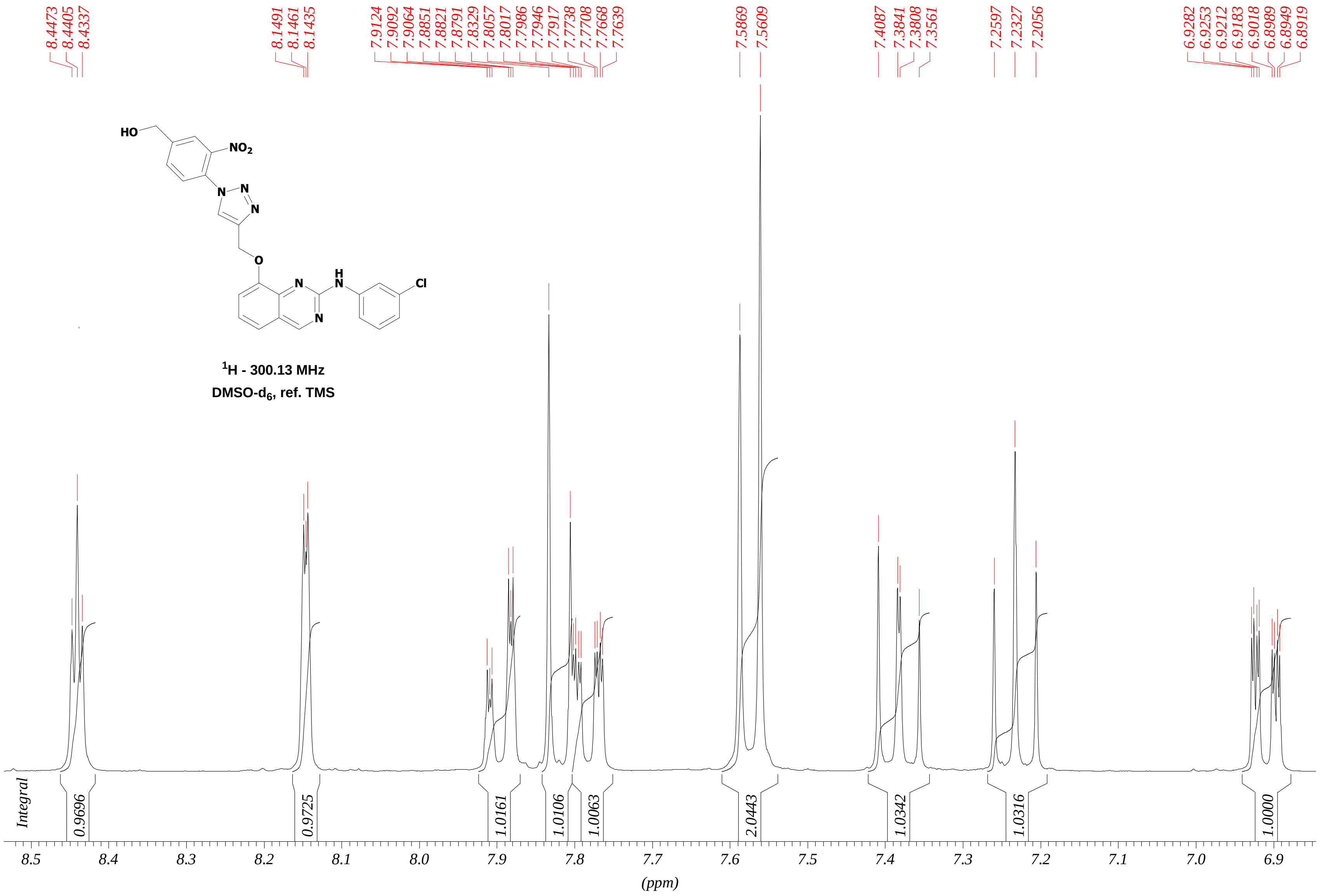
HO
NO2
N
N
N
O
H
N
Cl
N
N
1H - 300.13 MHz
DMSO-d6, ref. TMS
8.4473
8.4405
8.4337
8.1491
8.1461
8.1435
7.9124
7.9092
7.9064
7.8851
7.8821
7.8791
7.8329
7.8057
7.8017
7.7986
7.7946
7.7917
7.7738
7.7708
7.7668
7.7639
7.5869
7.5609
7.4087
7.3841
7.3808
7.3561
7.2597
7.2327
7.2056
6.9282
6.9253
6.9212
6.9183
6.9018
6.8989
6.8949
6.8919
Integral
0.9696
0.9725
1.0161
1.0106
1.0063
2.0443
1.0342
1.0316
1.0000
8.5
8.4
8.3
8.2
8.1
8.0
7.9
7.8
7.7
7.6
7.5
7.4
7.3
7.2
7.1
7.0
6.9
(ppm)

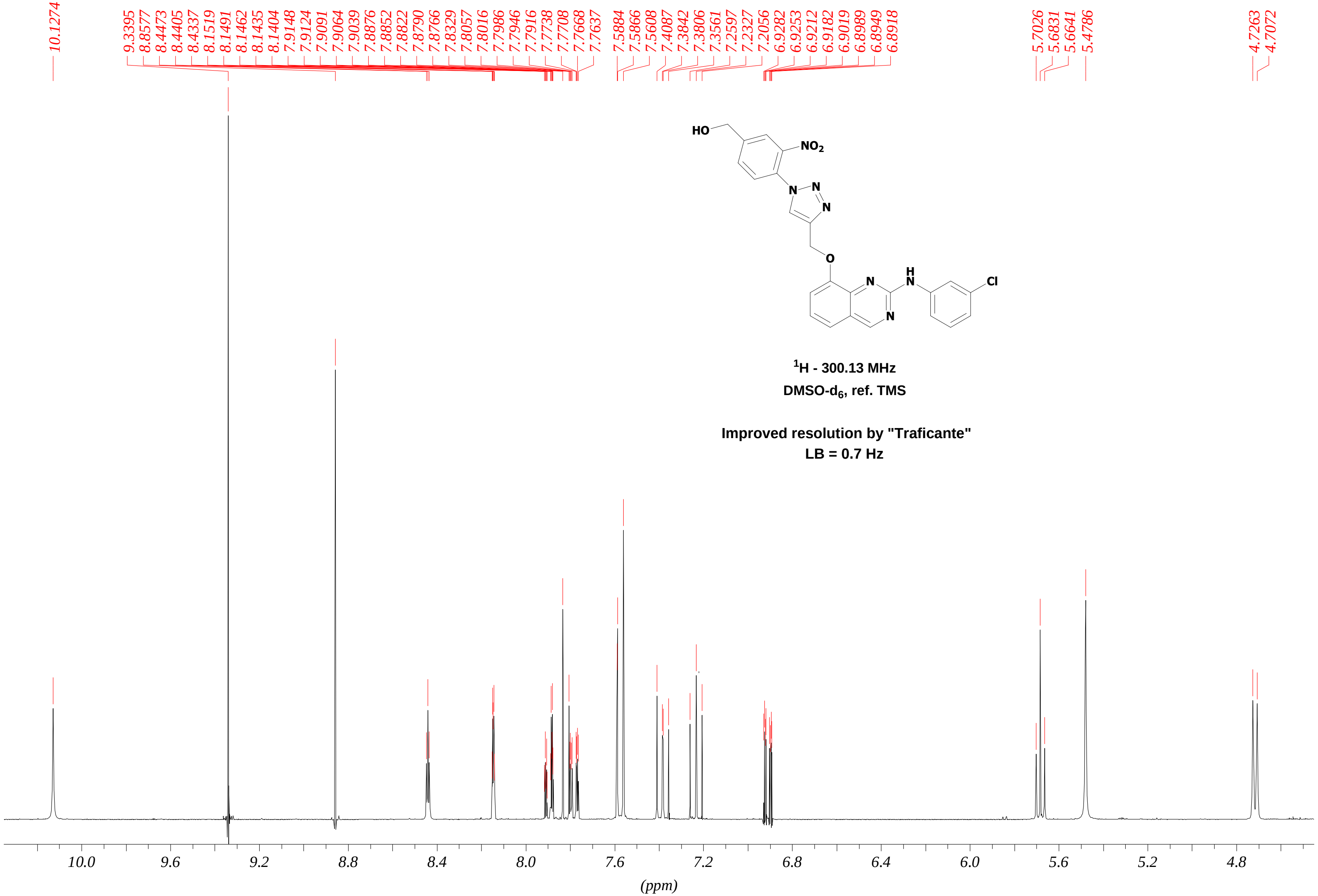
10.1274
9.3395 8.8577 8.4473 8.4405 8.4337 8.1519 8.1491 8.1462 8.1435 8.1404 7.9148 7.9124 7.9091 7.9064 7.9039 7.8876 7.8852 7.8822 7.8790 7.8766 7.8329 7.8057 7.8016 7.7986 7.7946 7.7916 7.7738 7.7708 7.7668 7.7637
7.5884 7.5866 7.5608 7.4087 7.3842 7.3806 7.3561 7.2597 7.2327 7.2056 6.9282 6.9253 6.9212 6.9182 6.9019 6.8989 6.8949 6.8918
5.7026 5.6831 5.6641 5.4786
4.7263 4.7072
HO
NO_2
N
N
N
O
N
H
N
Cl
N
^{1}H - 300.13 MHz
DMSO-d_6, ref. TMS
Improved resolution by "Traficante"
LB = 0.7 Hz
10.0
9.6
9.2
8.8
8.4
8.0
7.6
7.2
6.8
6.4
6.0
5.6
5.2
4.8
(ppm)

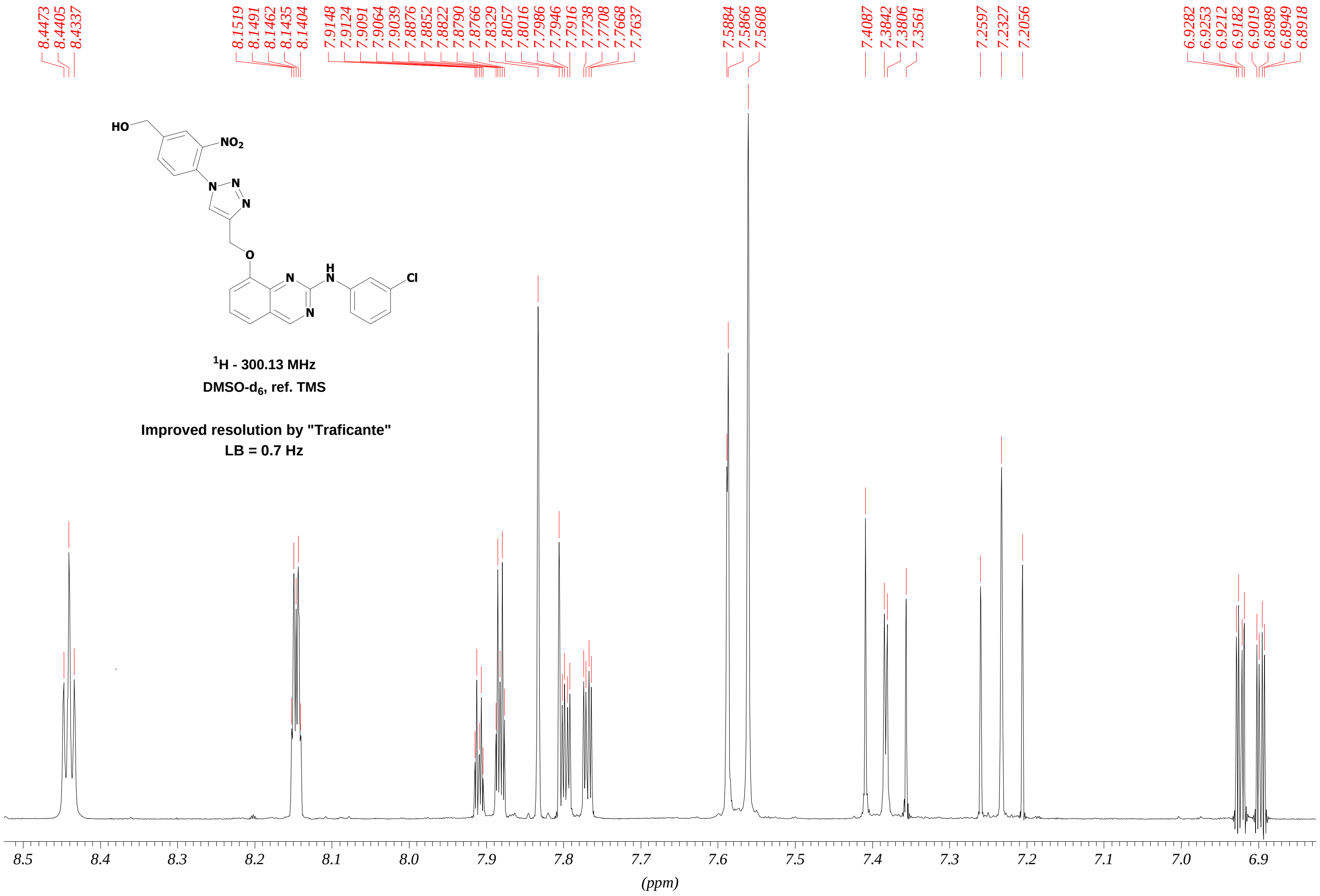

^{1}H - 300.13 MHz
DMSO-d_6, ref. TMS

Improved resolution by "Traficante"
LB = 0.7 Hz

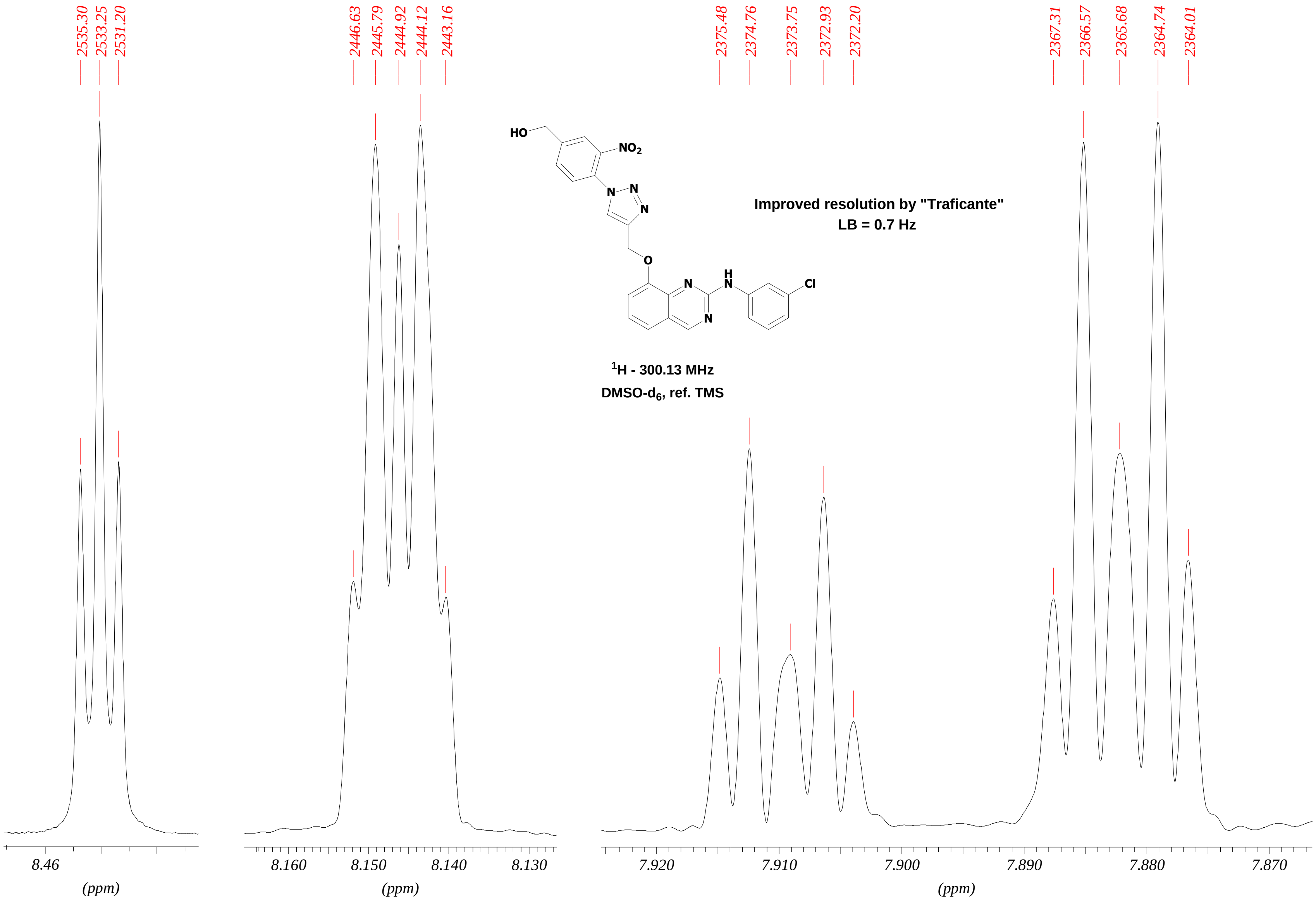

2535.30
2533.25
2531.20
2446.63
2445.79
2444.92
2444.12
2443.16
2375.48
2374.76
2373.75
2372.93
2372.20
2367.31
2366.57
2365.68
2364.74
2364.01
HO
NO2
N
N
N
O
N
H
N
Cl
N
Improved resolution by "Traficante"
LB = 0.7 Hz
1H - 300.13 MHz
DMSO-d6, ref. TMS
8.46
(ppm)
8.160
8.150
8.140
8.130
(ppm)
7.920
7.910
7.900
7.890
7.880
7.870
(ppm)

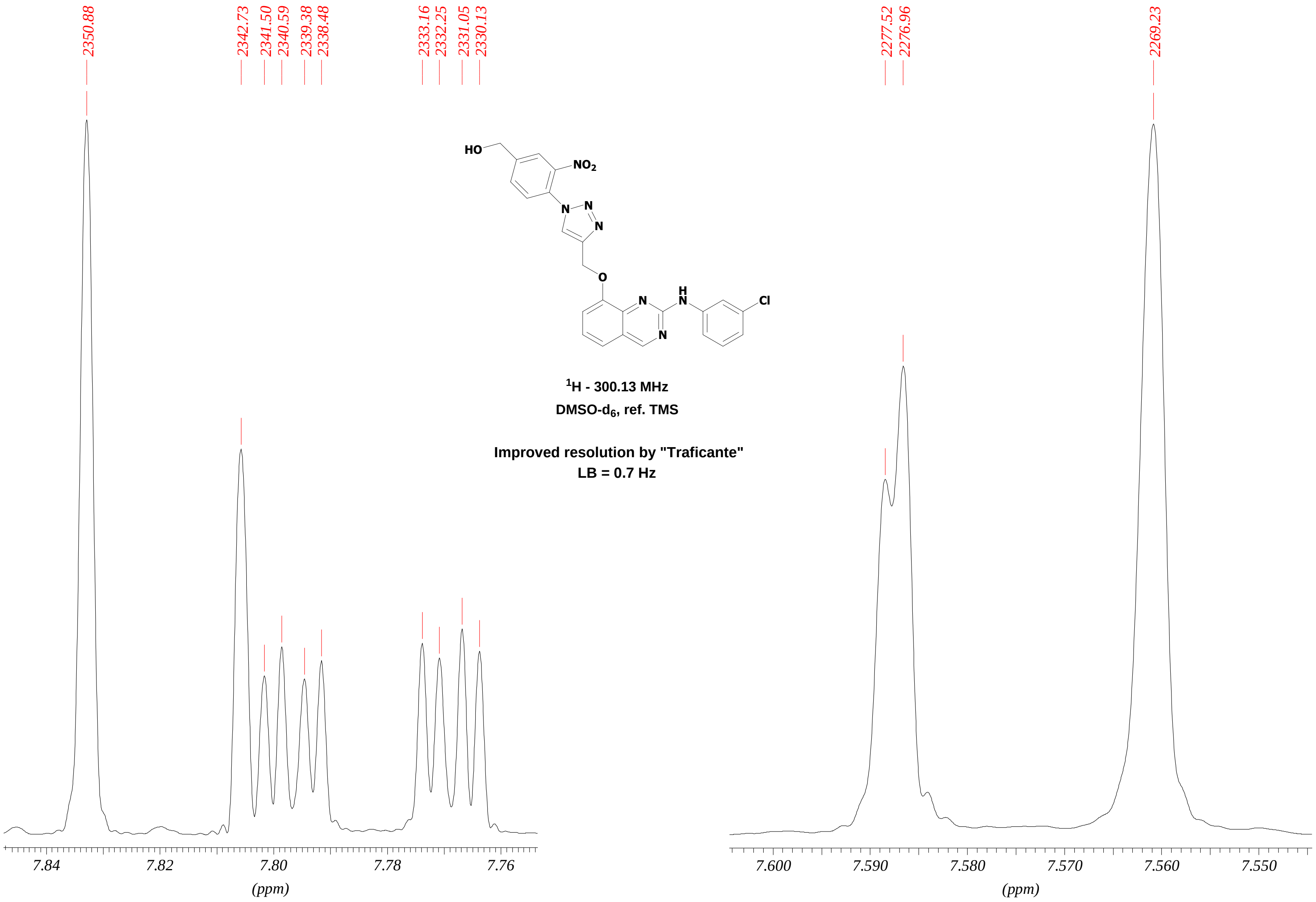

2350.88
2342.73
2341.50
2340.59
2339.38
2338.48
2333.16
2332.25
2331.05
2330.13
2277.52
2276.96
2269.23
HO
NO2
N
N
N
O
N
H
N
Cl
N
1H - 300.13 MHz
DMSO-d6, ref. TMS
Improved resolution by "Traficante"
LB = 0.7 Hz
7.84
7.82
7.80
7.78
7.76
(ppm)
7.600
7.590
7.580
7.570
7.560
7.550
(ppm)

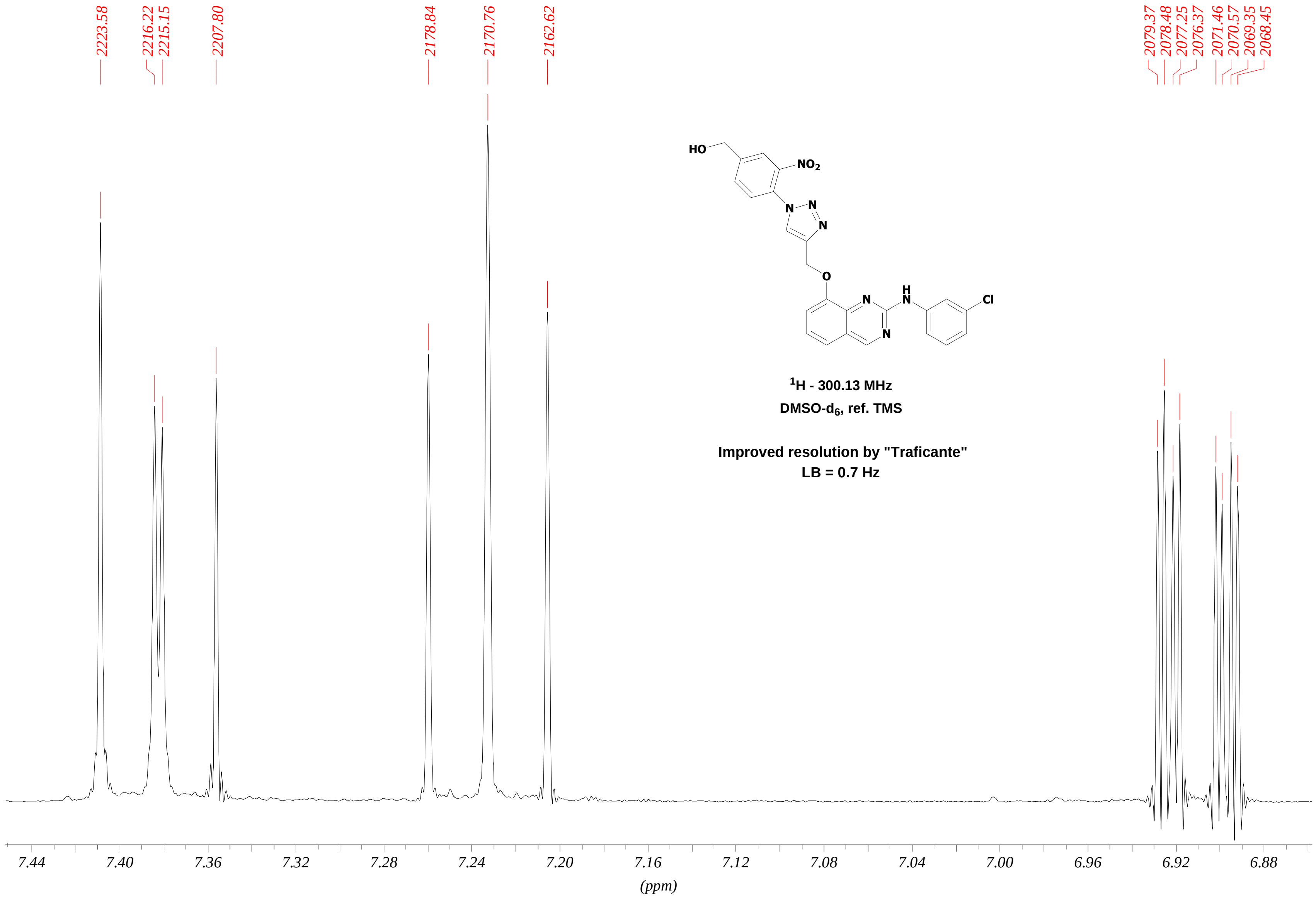
2223.58
2216.22
2215.15
2207.80
2178.84
2170.76
2162.62
2079.37
2078.48
2077.25
2076.37
2071.46
2070.57
2069.35
2068.45
HO
NO2
N
N
N
O
N
H
N
Cl
N
1H - 300.13 MHz
DMSO-d6, ref. TMS
Improved resolution by "Traficante"
LB = 0.7 Hz
7.44
7.40
7.36
7.32
7.28
7.24
7.20
7.16
7.12
7.08
7.04
7.00
6.96
6.92
6.88
(ppm)

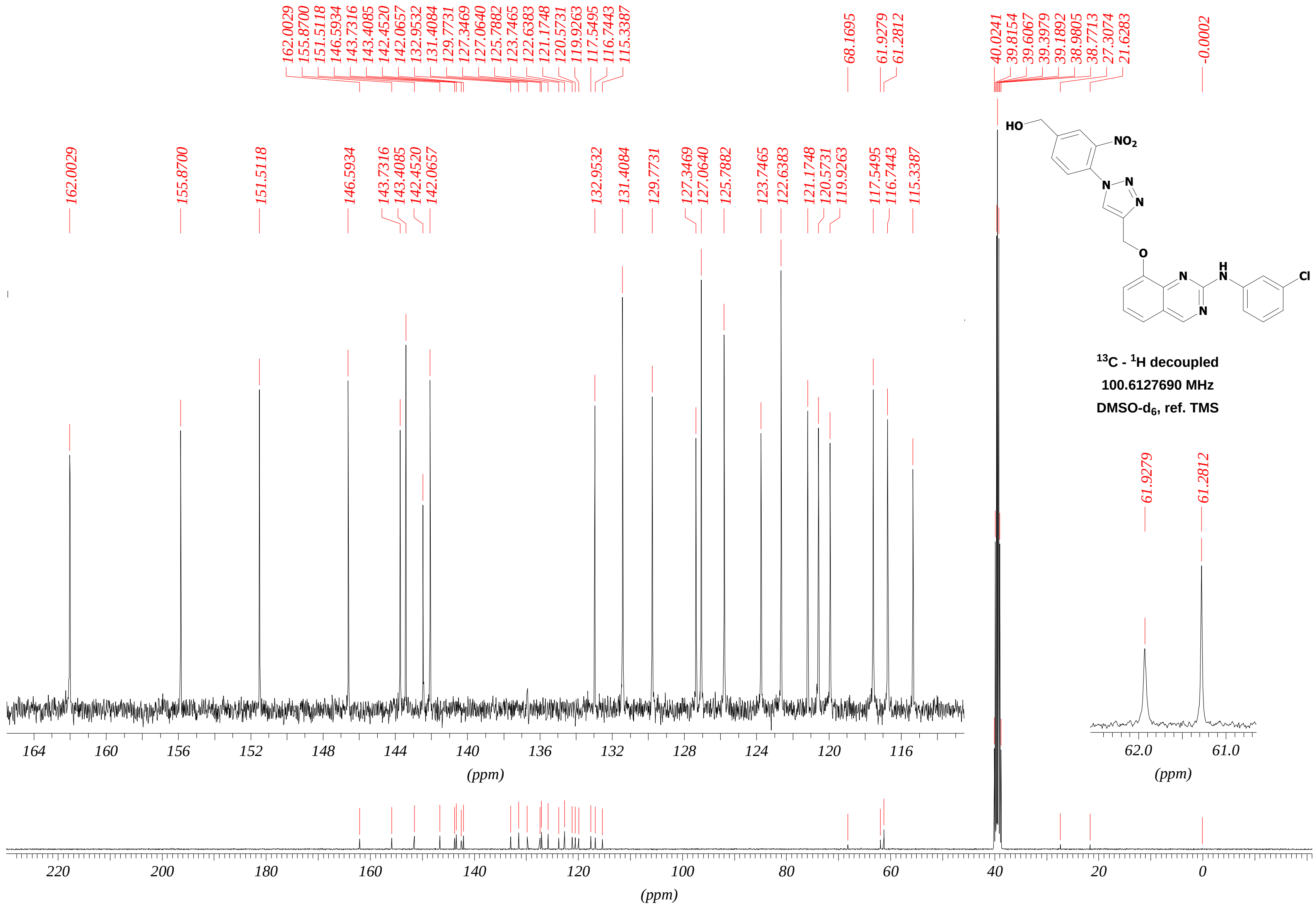

162.0029
155.8700
151.5118
146.5934
143.7316
143.4085
142.4520
142.0657
132.9532
131.4084
129.7731
127.3469
127.0640
125.7882
123.7465
122.6383
121.1748
120.5731
119.9263
117.5495
116.7443
115.3387
68.1695
61.9279
61.2812
40.0241
39.8154
39.6067
39.3979
39.1892
38.9805
38.7713
27.3074
21.6283
-0.0002
HO
NO_2
N
O
H
Cl
^{13}C - ^{1}H decoupled
100.6127690 MHz
DMSO-d_6, ref. TMS
(ppm)

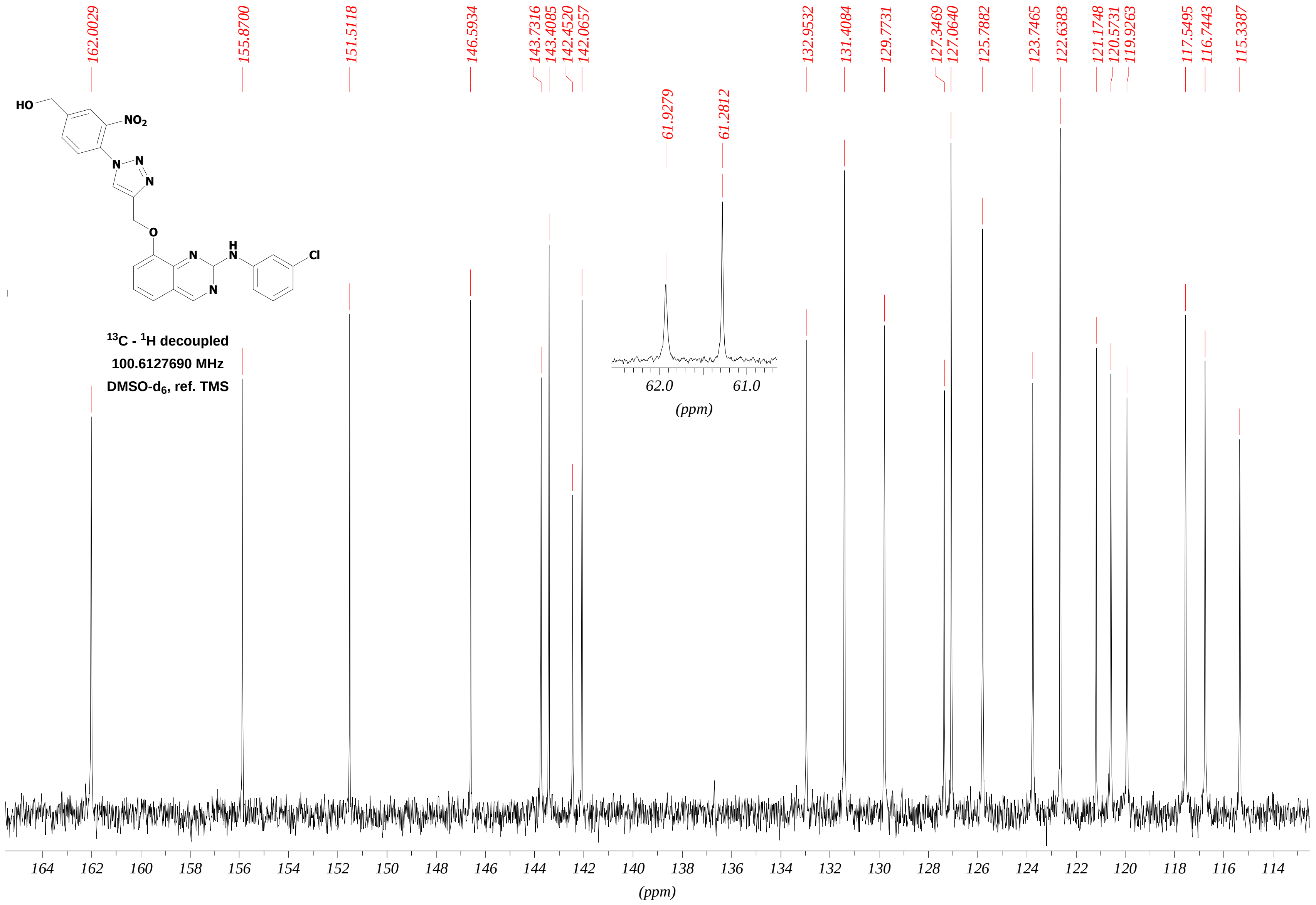

HO
NO2
N
N
N
O
N
H N
Cl
N
13C - 1H decoupled
100.6127690 MHz
DMSO-d6, ref. TMS
162.0029
155.8700
151.5118
146.5934
143.7316
143.4085
142.4520
142.0657
61.9279
61.2812
132.9532
131.4084
129.7731
127.3469
127.0640
125.7882
123.7465
122.6383
121.1748
120.5731
119.9263
117.5495
116.7443
115.3387
62.0
61.0
(ppm)
164
162
160
158
156
154
152
150
148
146
144
142
140
138
136
134
132
130
128
126
124
122
120
118
116
114
(ppm)

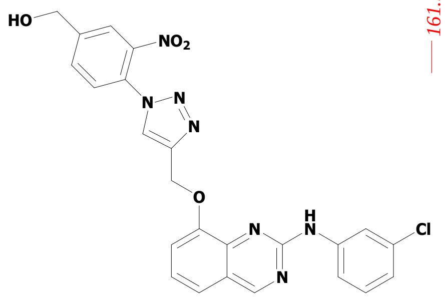

^{13}C - DEPT 135
75.4677490 MHz
DMSO-d$_6$, ref. TMS

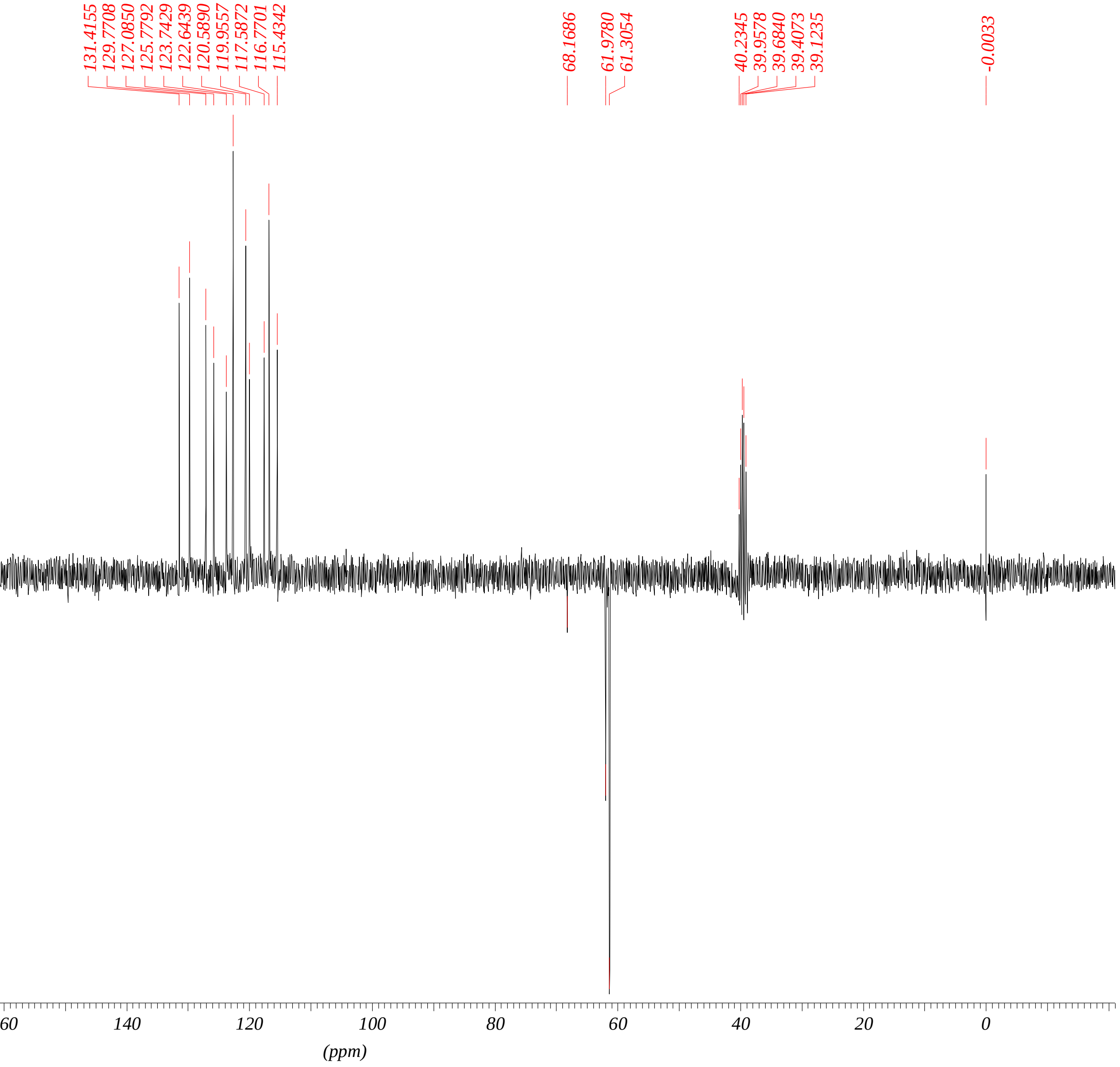

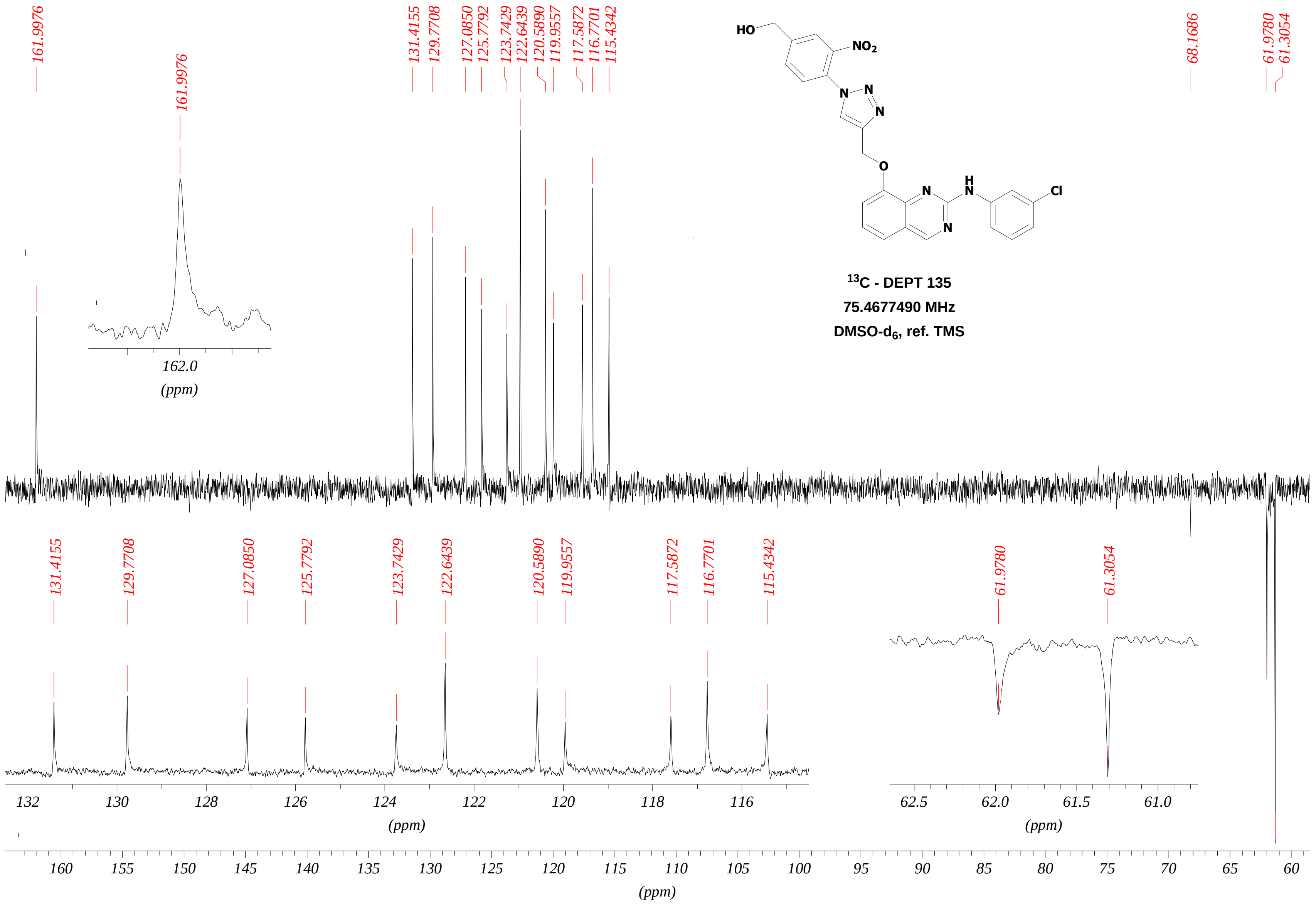

^{13}C - DEPT 135
75.4677490 MHz
DMSO-d_6, ref. TMS
HO
NO_2
N
O
H
Cl
161.9976
131.4155
129.7708
127.0850
125.7792
123.7429
122.6439
120.5890
119.9557
117.5872
116.7701
115.4342
68.1686
61.9780
61.3054
162.0
(ppm)

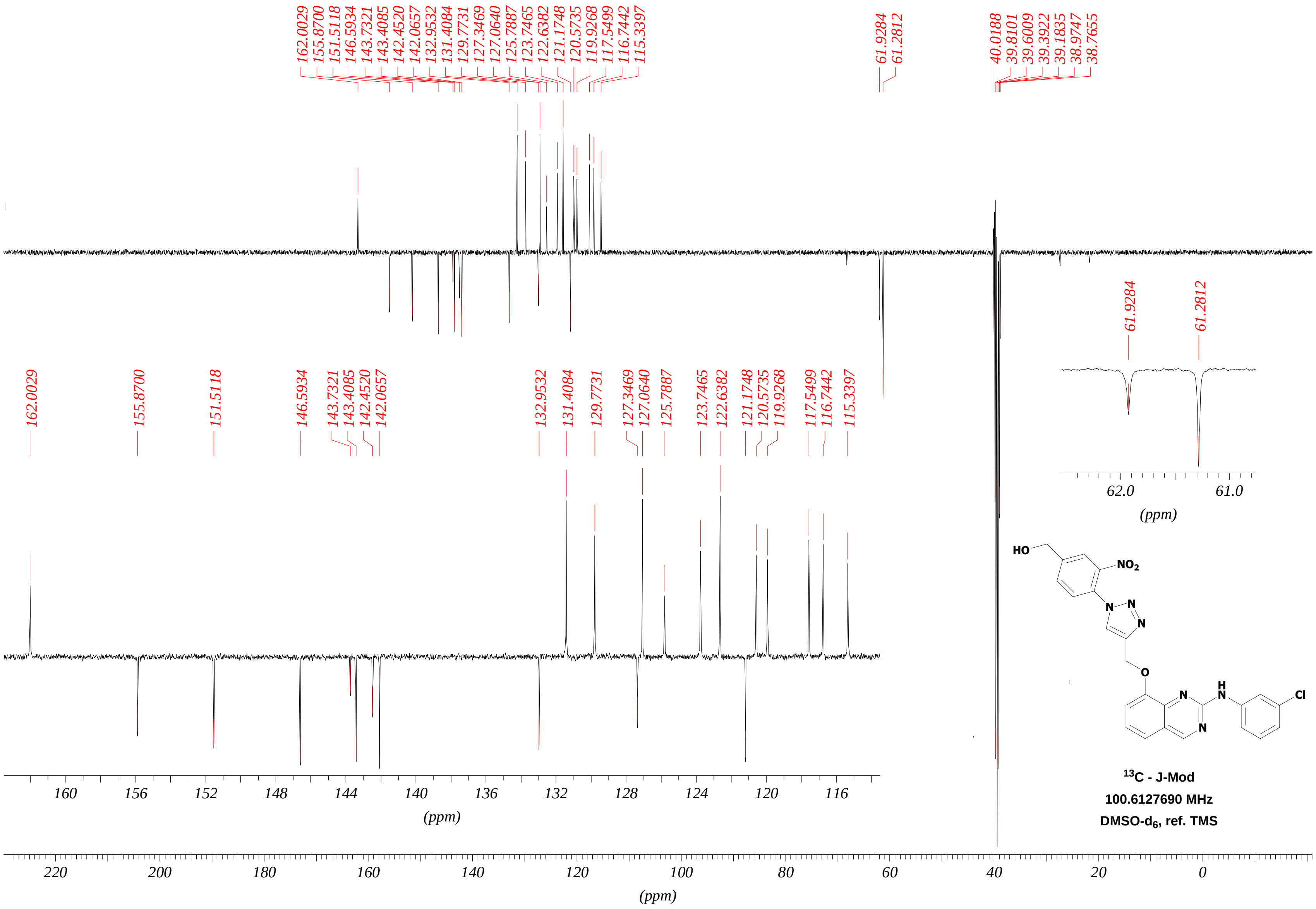
162.0029
155.8700
151.5118
146.5934
143.7321
143.4085
142.4520
142.0657
132.9532
131.4084
129.7731
127.3469
127.0640
125.7887
123.7465
122.6382
121.1748
120.5735
119.9268
117.5499
116.7442
115.3397
61.9284
61.2812
40.0188
39.8101
39.6009
39.3922
39.1835
38.9747
38.7655
HO
NO_2
N
N
N
O
N
H
N
Cl
N
(ppm)
^{13}C - J-Mod
100.6127690 MHz
DMSO-d_6, ref. TMS

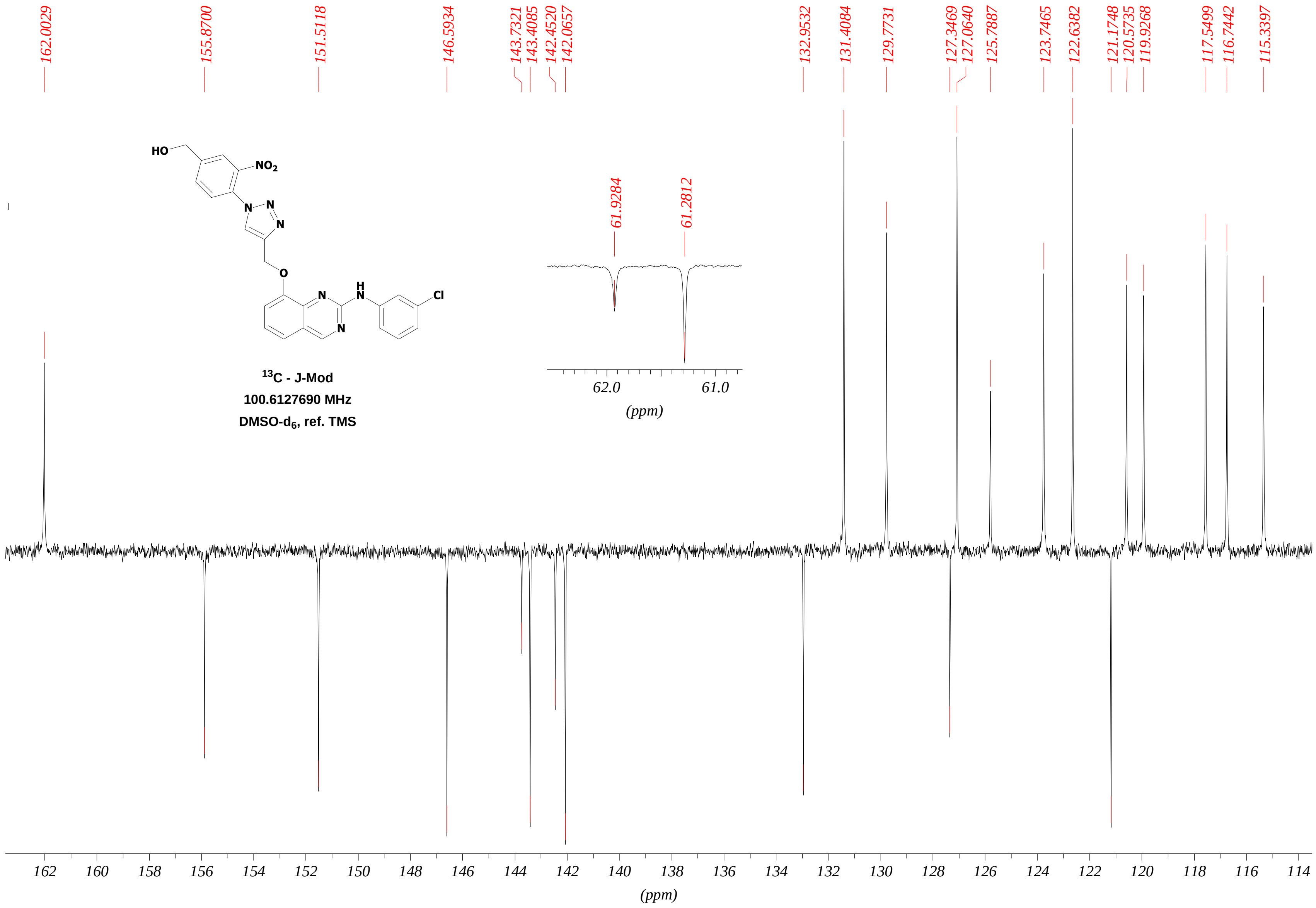

162.0029
155.8700
151.5118
146.5934
143.7321
143.4085
142.4520
142.0657
132.9532
131.4084
129.7731
127.3469
127.0640
125.7887
123.7465
122.6382
121.1748
120.5735
119.9268
117.5499
116.7442
115.3397
HO
NO2
N
N
N
O
N
H
N
Cl
N
13C - J-Mod
100.6127690 MHz
DMSO-d6, ref. TMS
61.9284
61.2812
62.0
61.0
(ppm)
162
160
158
156
154
152
150
148
146
144
142
140
138
136
134
132
130
128
126
124
122
120
118
116
114
(ppm)

Methyl 4-(4-(((2-((3-chlorophenyl)amino)quinazolin-8-yl)oxy)methyl)-1*H*-1,2,3-triazol-1-yl)-3-nitrobenzoate (12f):

Pages S90-S101

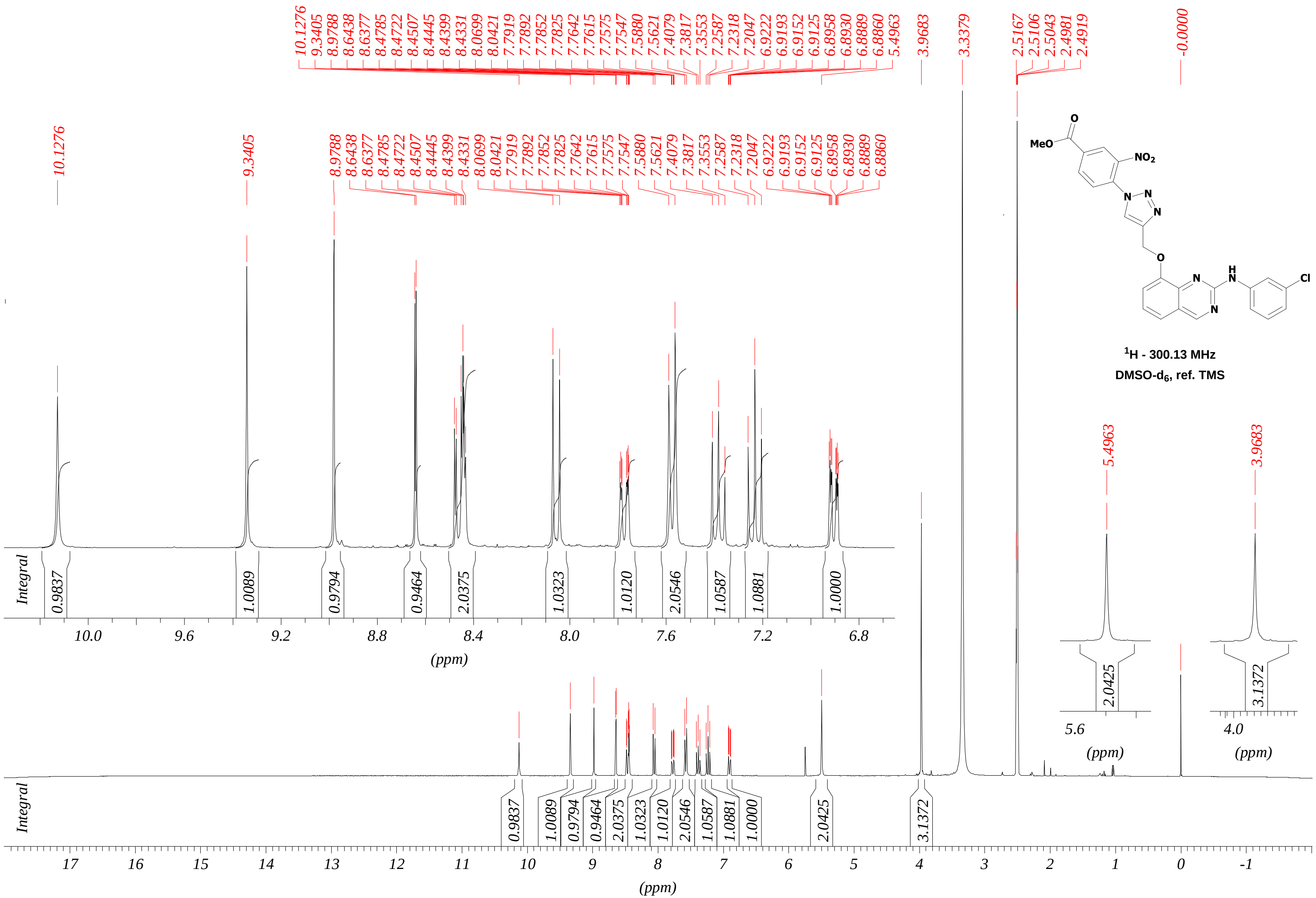
10.1276 9.3405 8.9788 8.6438 8.6377 8.4785 8.4722 8.4507 8.4445 8.4399 8.4331 8.0699 8.0421 7.7919 7.7892 7.7852 7.7825 7.7642 7.7615 7.7575 7.7547 7.5880 7.5621 7.4079 7.3817 7.3553 7.2587 7.2318 7.2047 6.9222 6.9193 6.9152 6.9125 6.8958 6.8930 6.8889 6.8860 5.4963 3.9683 3.3379 2.5167 2.5106 2.5043 2.4981 2.4919 -0.0000
MeO
O
NO2
N
O
H
Cl
1H - 300.13 MHz
DMSO-d6, ref. TMS
Integral
0.9837 1.0089 0.9794 0.9464 2.0375 1.0323 1.0120 2.0546 1.0587 1.0881 1.0000 2.0425 3.1372
(ppm)

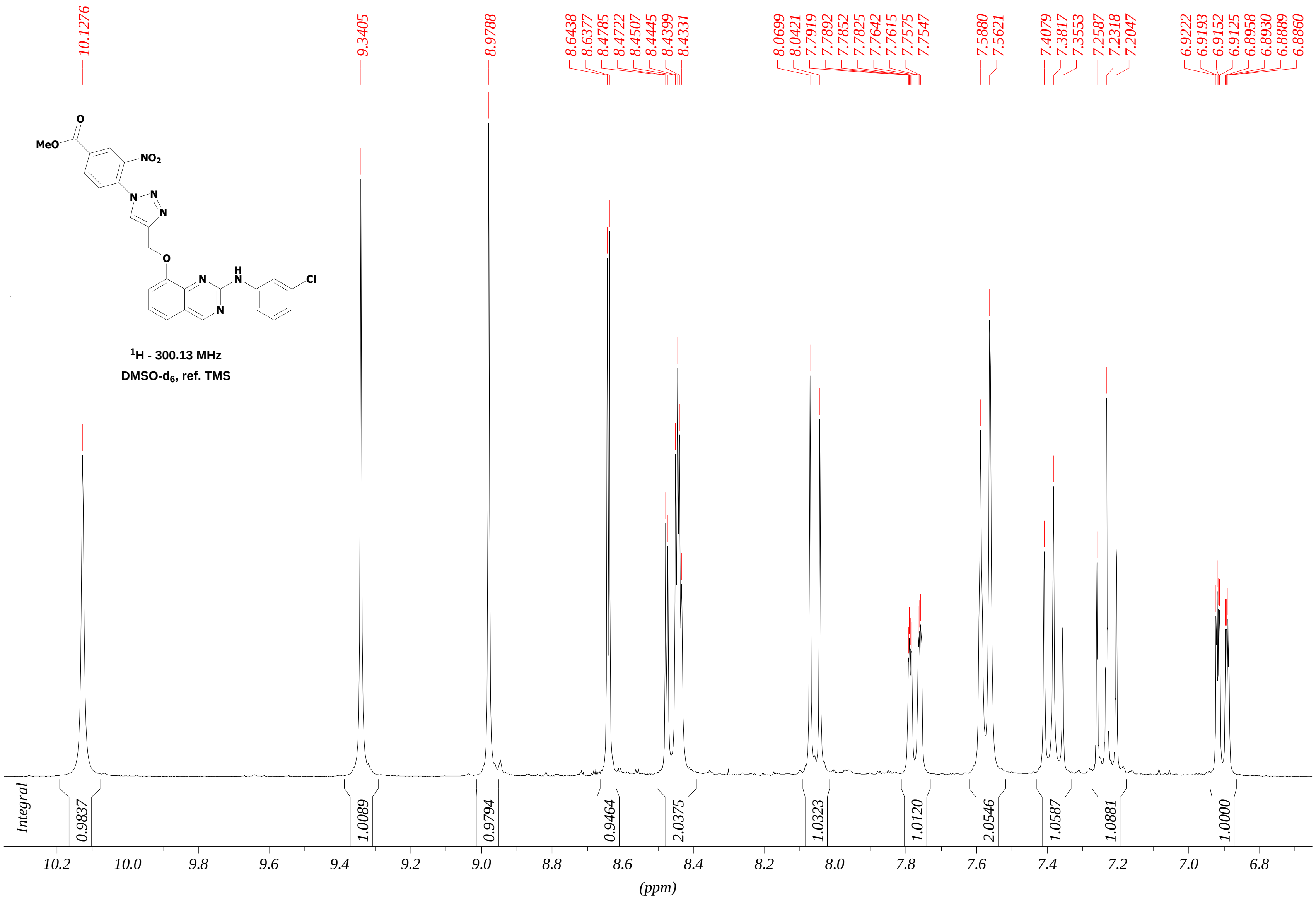
MeO
O
NO2
N
N
N
O
N
N
H
N
Cl
1H - 300.13 MHz
DMSO-d6, ref. TMS
10.1276
9.3405
8.9788
8.6438
8.6377
8.4785
8.4722
8.4507
8.4445
8.4399
8.4331
8.0699
8.0421
7.7919
7.7892
7.7852
7.7825
7.7642
7.7615
7.7575
7.7547
7.5880
7.5621
7.4079
7.3817
7.3553
7.2587
7.2318
7.2047
6.9222
6.9193
6.9152
6.9125
6.8958
6.8930
6.8889
6.8860
Integral
0.9837
1.0089
0.9794
0.9464
2.0375
1.0323
1.0120
2.0546
1.0587
1.0881
1.0000
10.2
10.0
9.8
9.6
9.4
9.2
9.0
8.8
8.6
8.4
8.2
8.0
7.8
7.6
7.4
7.2
7.0
6.8
(ppm)

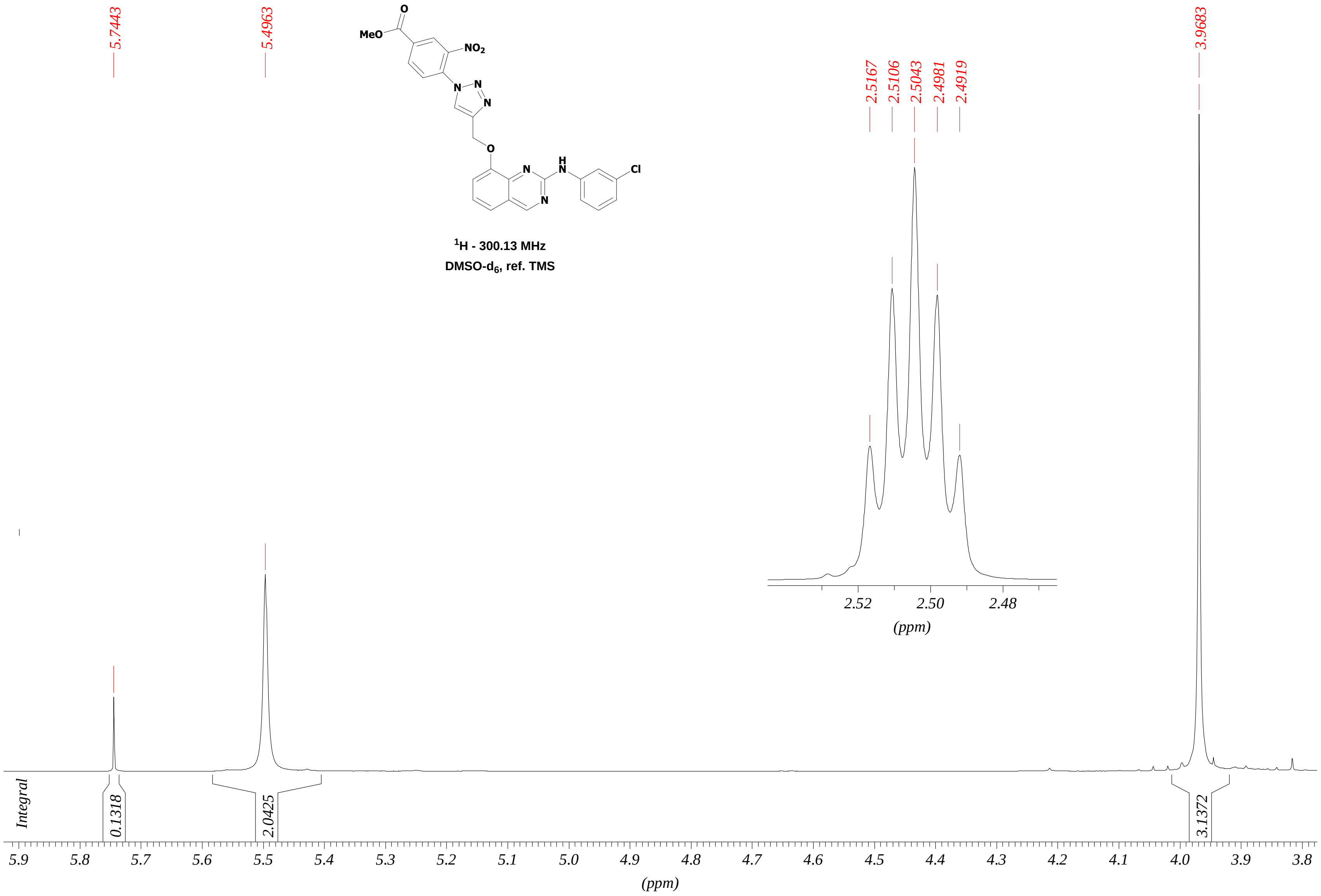

MeO
O
NO2
N
N
N
O
N
N
H
N
Cl
1H - 300.13 MHz
DMSO-d6, ref. TMS
5.7443
5.4963
3.9683
2.5167
2.5106
2.5043
2.4981
2.4919
2.52
2.50
2.48
(ppm)
Integral
0.1318
2.0425
3.1372
5.9
5.8
5.7
5.6
5.5
5.4
5.3
5.2
5.1
5.0
4.9
4.8
4.7
4.6
4.5
4.4
4.3
4.2
4.1
4.0
3.9
3.8
(ppm)

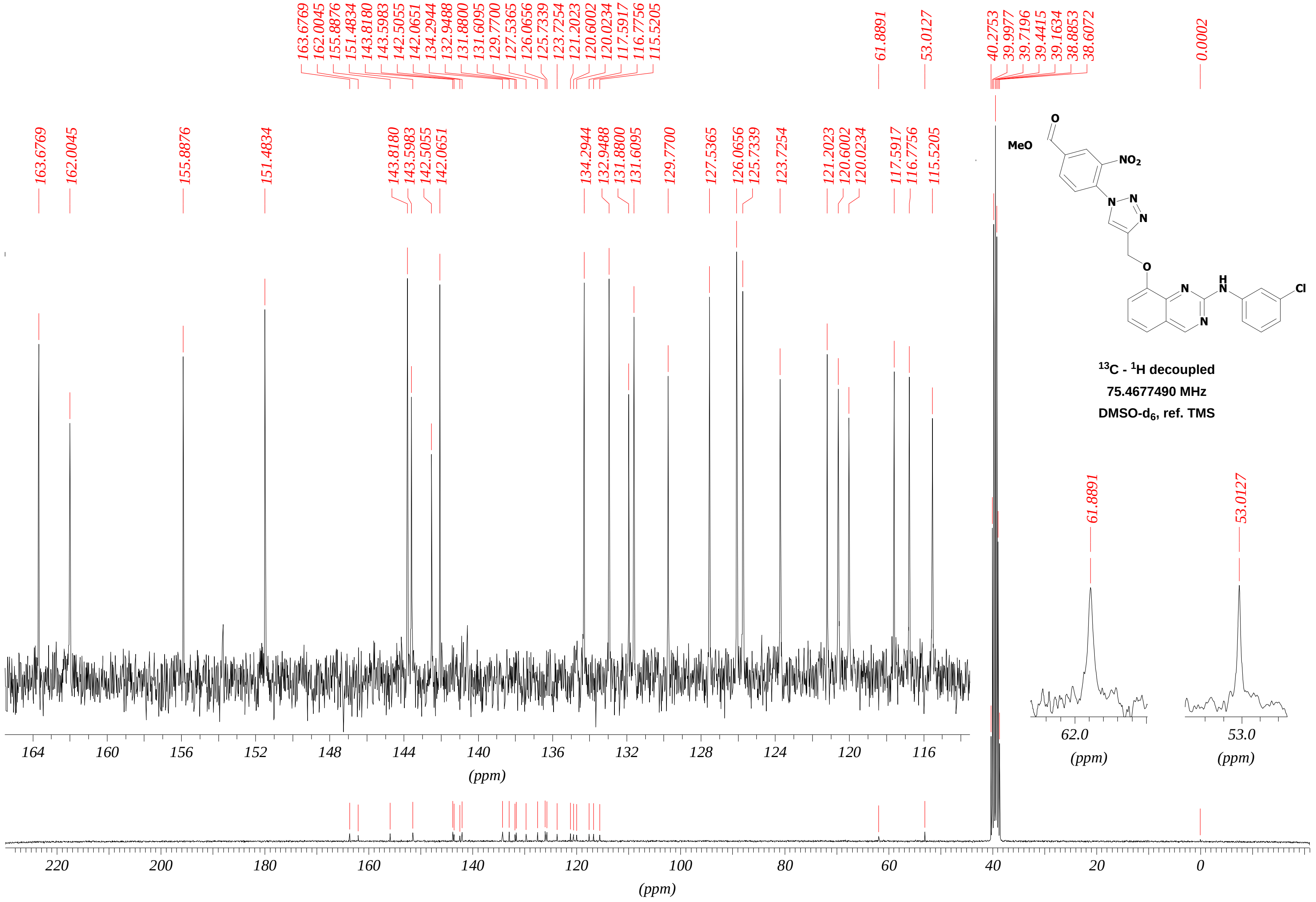
163.6769
162.0045
155.8876
151.4834
143.8180
143.5983
142.5055
142.0651
134.2944
132.9488
131.8800
131.6095
129.7700
127.5365
126.0656
125.7339
123.7254
121.2023
120.6002
120.0234
117.5917
116.7756
115.5205
61.8891
53.0127
40.2753
39.9977
39.7196
39.4415
39.1634
38.8853
38.6072
0.0002
MeO
O
NO_2
N
N
N
O
N
H
N
Cl
N
^{13}C - ^{1}H decoupled
75.4677490 MHz
DMSO-d_6, ref. TMS
(ppm)

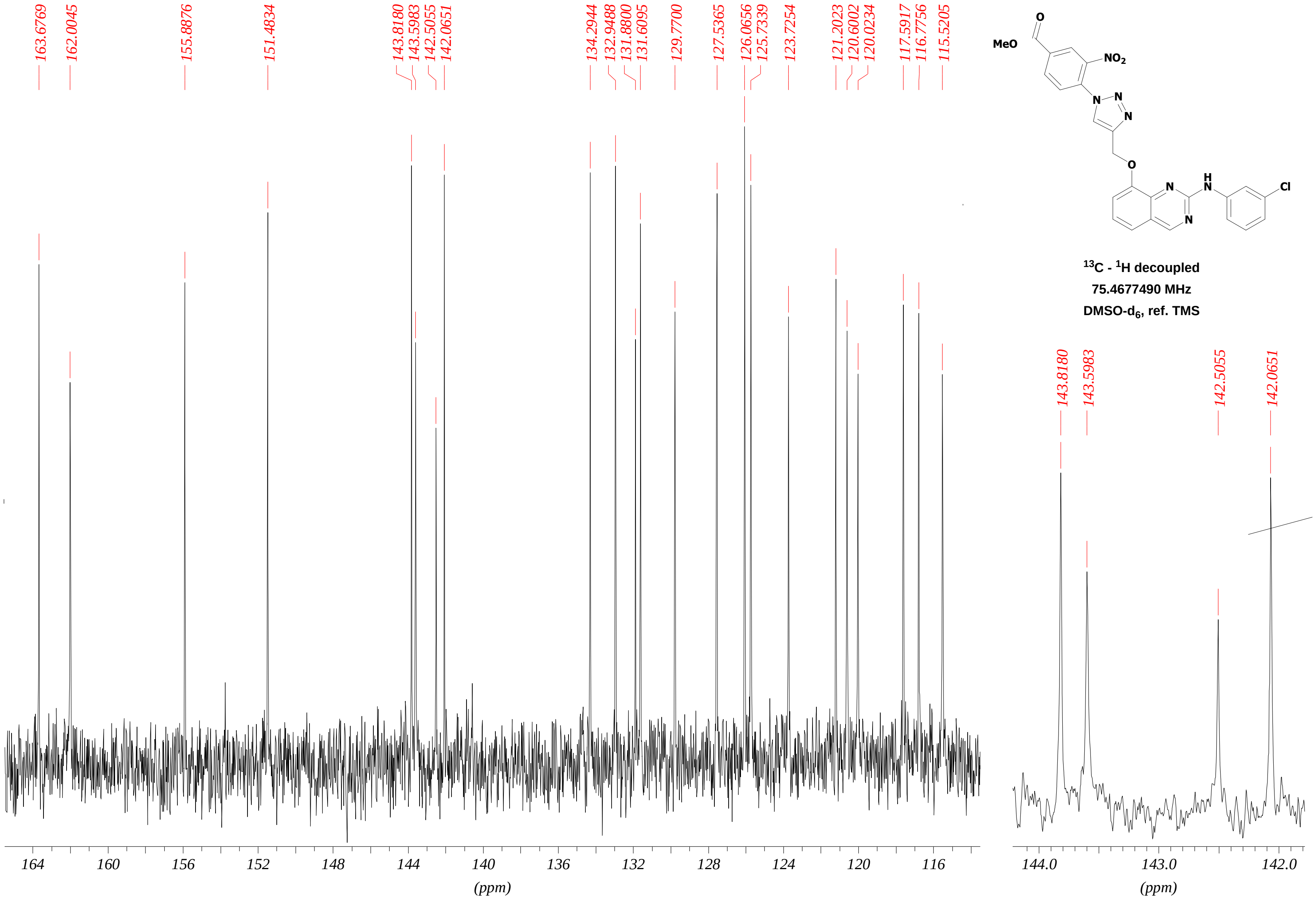
163.6769
162.0045
155.8876
151.4834
143.8180
143.5983
142.5055
142.0651
134.2944
132.9488
131.8800
131.6095
129.7700
127.5365
126.0656
125.7339
123.7254
121.2023
120.6002
120.0234
117.5917
116.7756
115.5205
MeO
O
NO2
N
N
N
O
N
H
N
Cl
N
13C - 1H decoupled
75.4677490 MHz
DMSO-d6, ref. TMS
164
160
156
152
148
144
140
136
132
128
124
120
116
(ppm)
143.8180
143.5983
142.5055
142.0651
144.0
143.0
142.0
(ppm)

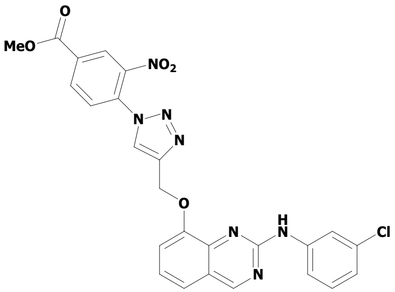

^{13}C - DEPT 135

100.6127690 MHz

DMSO-d_6, ref. TMS

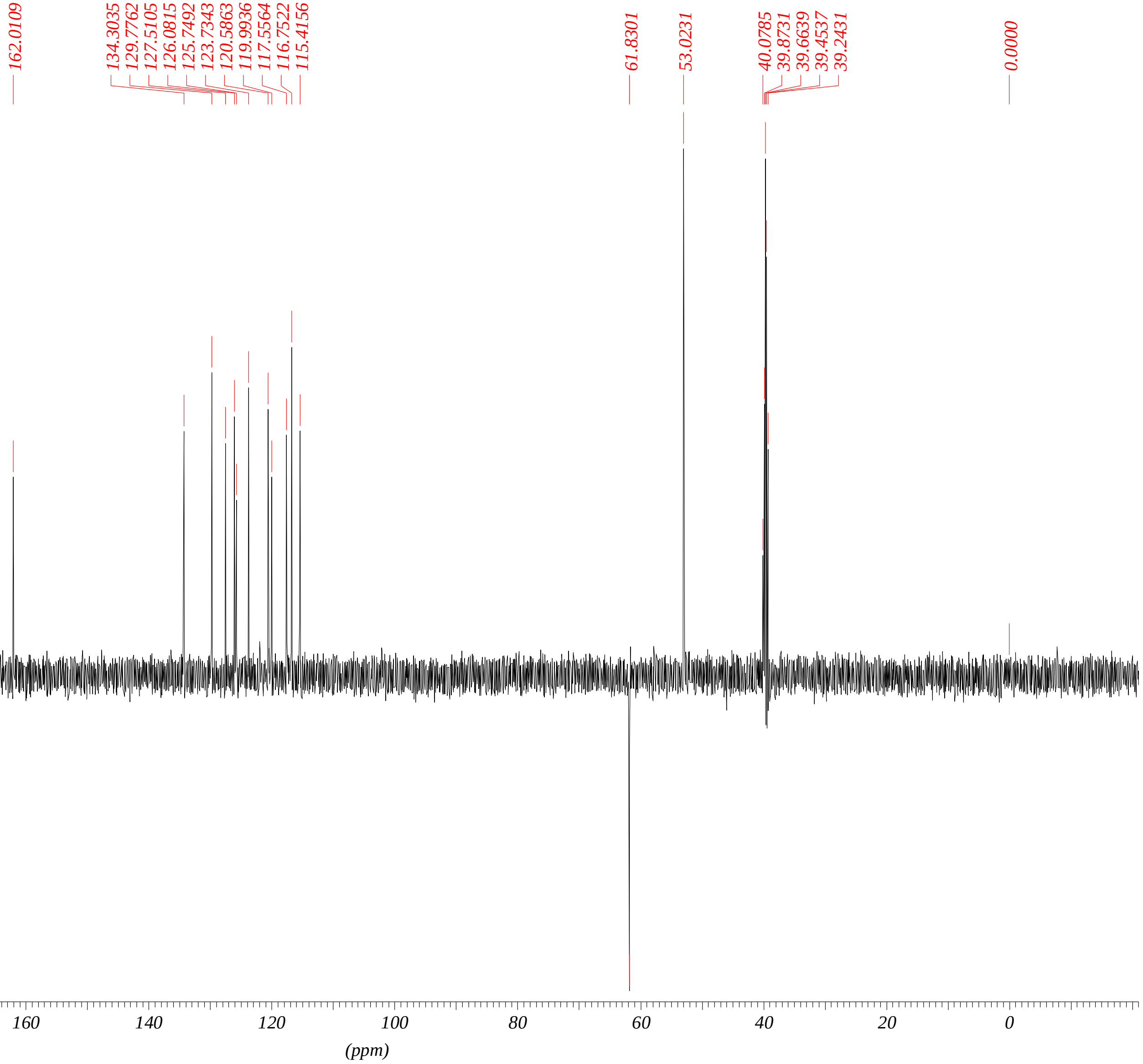

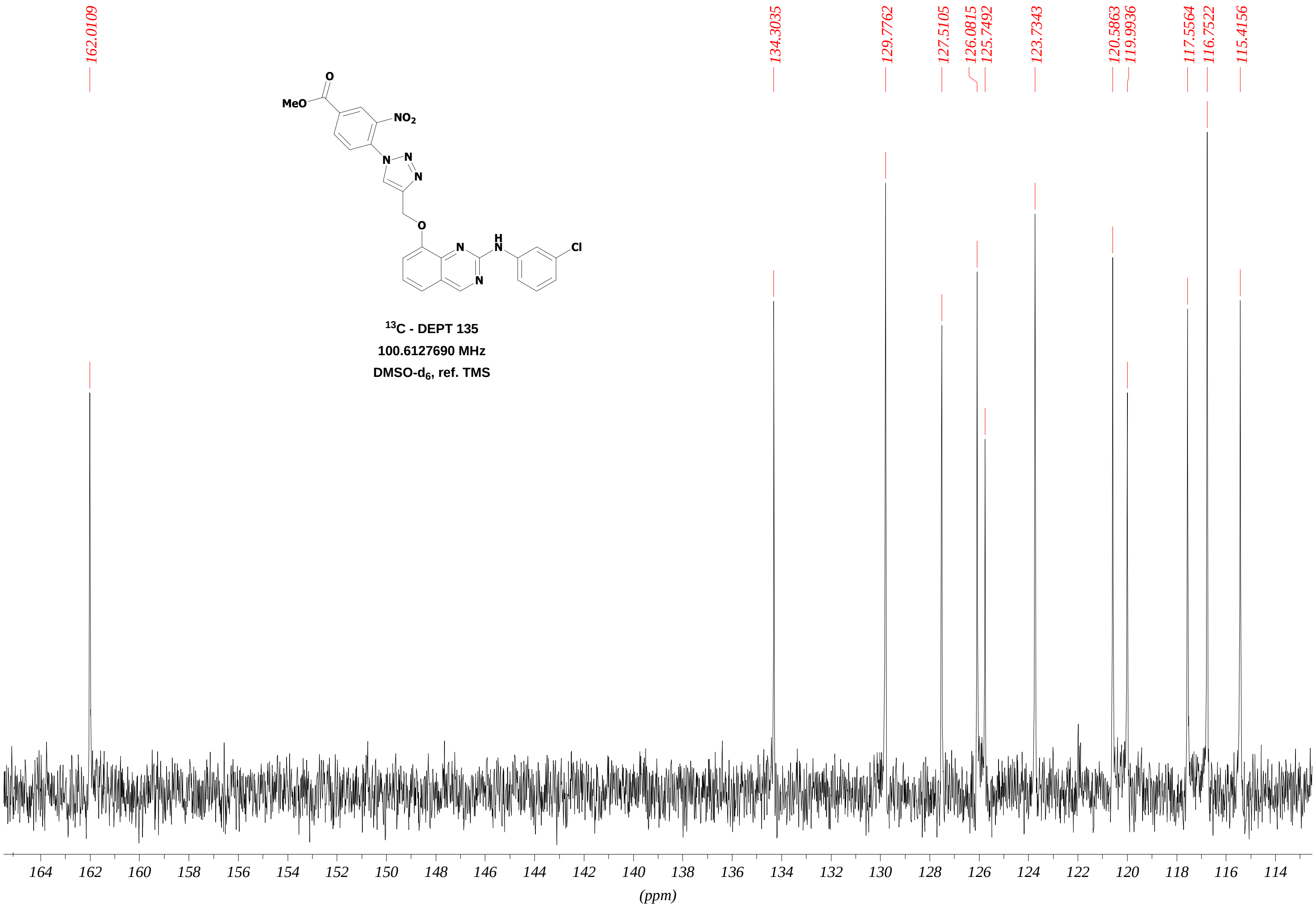
162.0109
134.3035
129.7762
127.5105
126.0815
125.7492
123.7343
120.5863
119.9936
117.5564
116.7522
115.4156
MeO
O
NO_2
N
N
N
O
N
N
H
N
Cl
^{13}C - DEPT 135
100.6127690 MHz
DMSO-d_6, ref. TMS
164
162
160
158
156
154
152
150
148
146
144
142
140
138
136
134
132
130
128
126
124
122
120
118
116
114
(ppm)

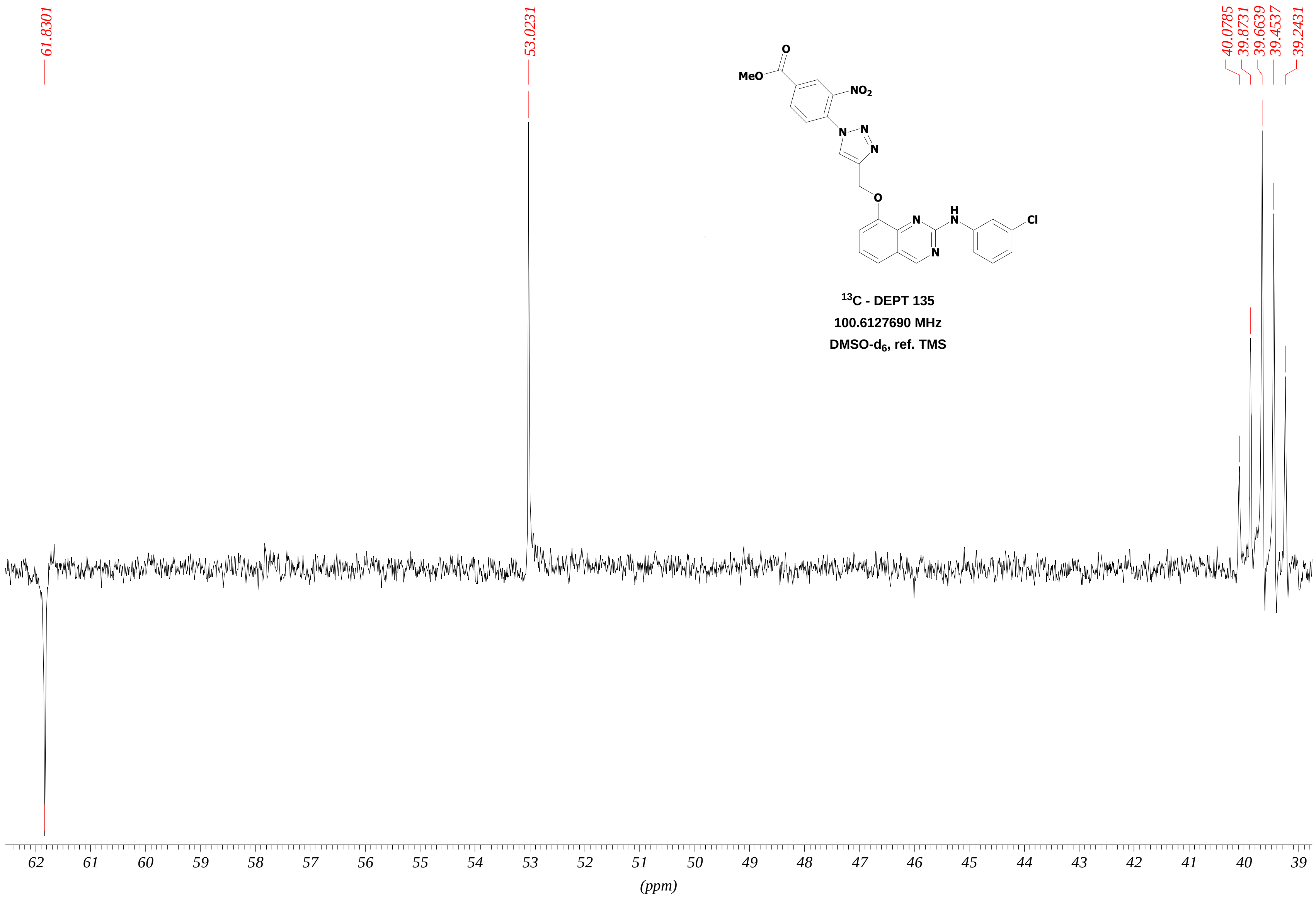

61.8301
53.0231
40.0785
39.8731
39.6639
39.4537
39.2431
MeO
O
NO2
N
N
N
O
N
N
H
N
Cl
13C - DEPT 135
100.6127690 MHz
DMSO-d6, ref. TMS
62
61
60
59
58
57
56
55
54
53
52
51
50
49
48
47
46
45
44
43
42
41
40
39
(ppm)

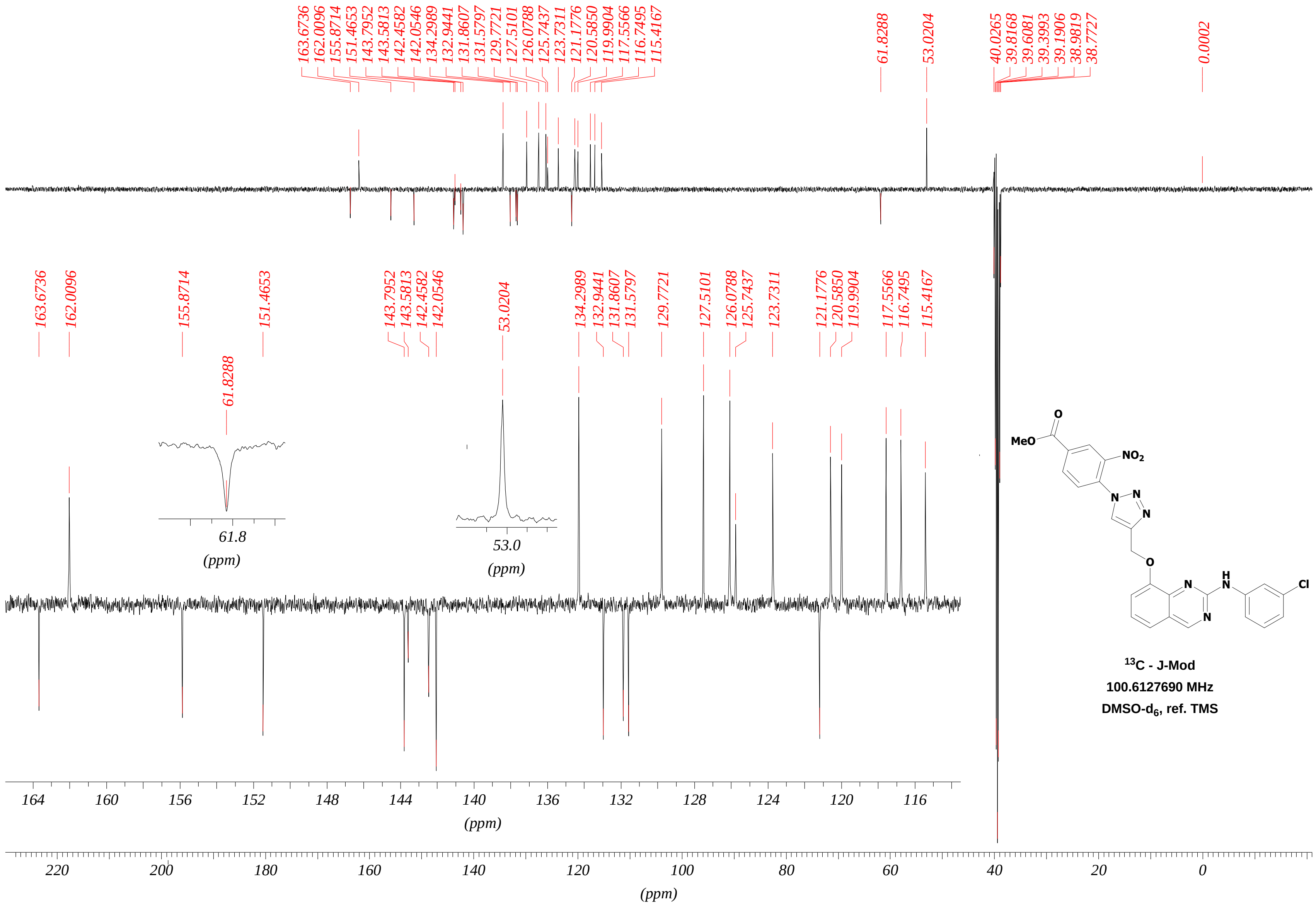

13C - J-Mod
100.6127690 MHz
DMSO-d6, ref. TMS

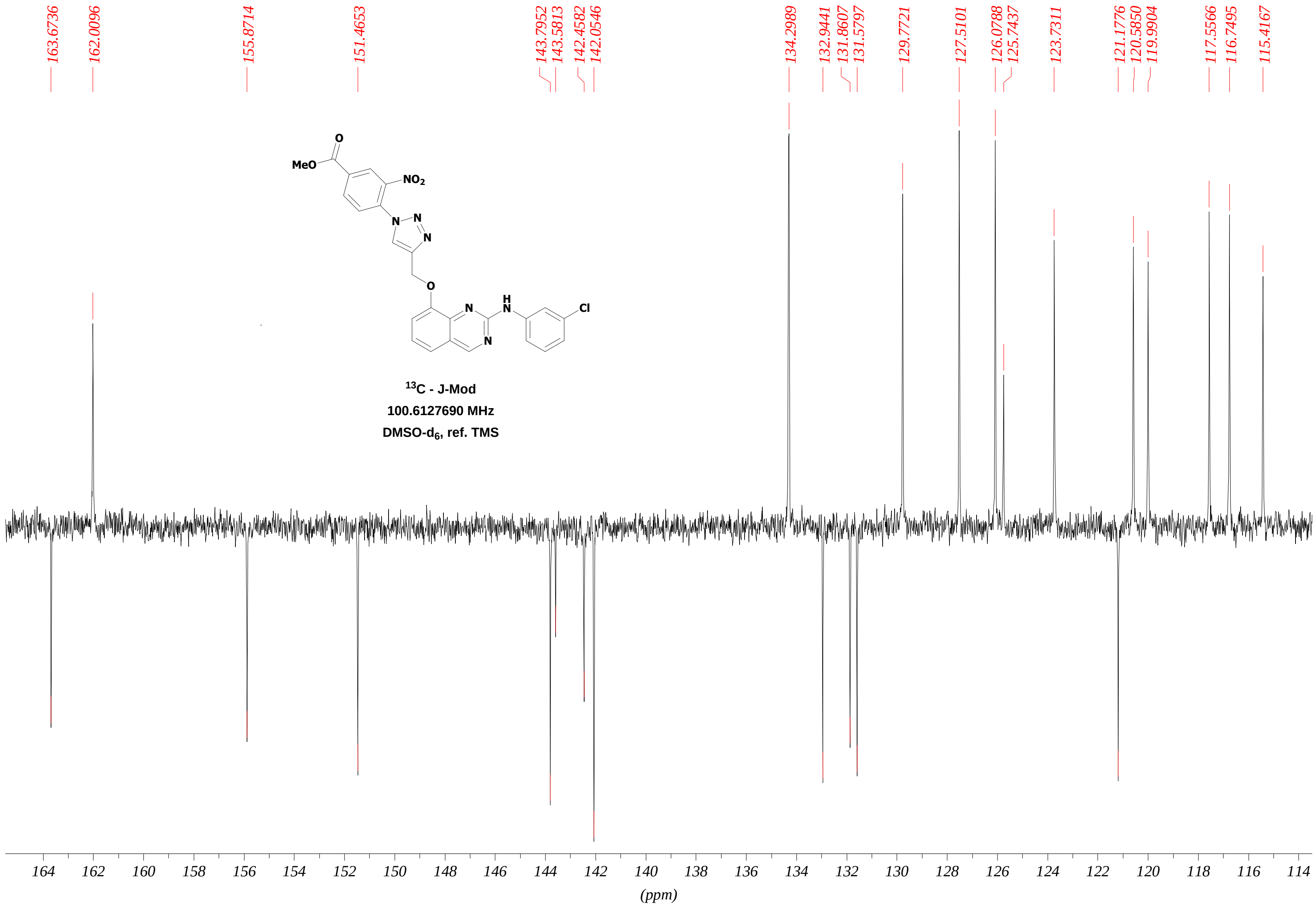
163.6736
162.0096
155.8714
151.4653
143.7952
143.5813
142.4582
142.0546
134.2989
132.9441
131.8607
131.5797
129.7721
127.5101
126.0788
125.7437
123.7311
121.1776
120.5850
119.9904
117.5566
116.7495
115.4167
MeO
O
NO2
N
N
N
O
N
N
H
N
Cl
13C - J-Mod
100.6127690 MHz
DMSO-d6, ref. TMS
164
162
160
158
156
154
152
150
148
146
144
142
140
138
136
134
132
130
128
126
124
122
120
118
116
114
(ppm)

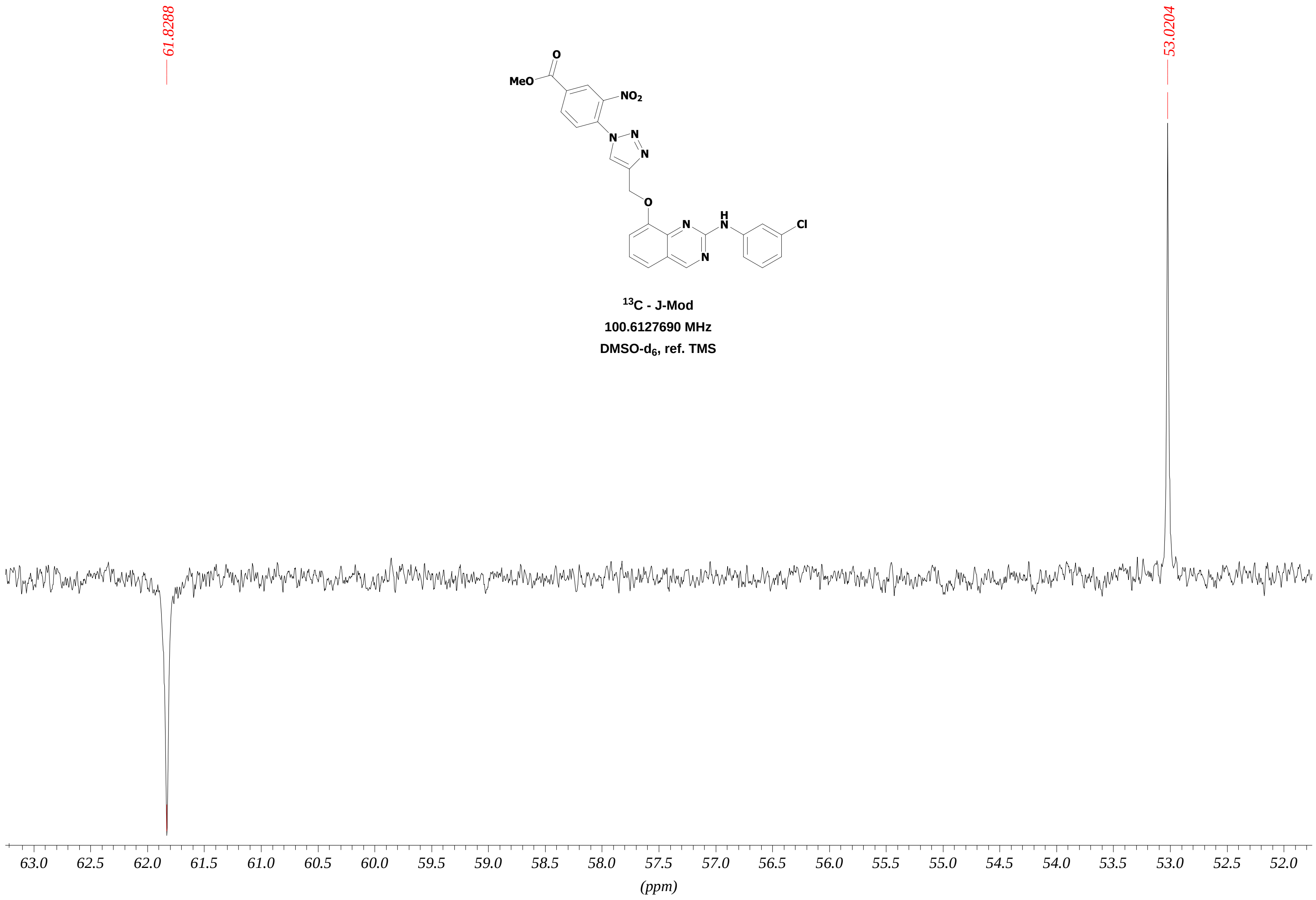

61.8288
53.0204
MeO
O
NO2
N
N
N
O
N
H
N
Cl
N
13C - J-Mod
100.6127690 MHz
DMSO-d6, ref. TMS
63.0
62.5
62.0
61.5
61.0
60.5
60.0
59.5
59.0
58.5
58.0
57.5
57.0
56.5
56.0
55.5
55.0
54.5
54.0
53.5
53.0
52.5
52.0
(ppm)

4-(4-(((2-((3-Chlorophenyl)amino)quinazolin-8-yl)oxy)methyl)-1*H*-1,2,3-triazol-1-yl)-3-nitrobenzoic acid (12g):

Pages S102-S112

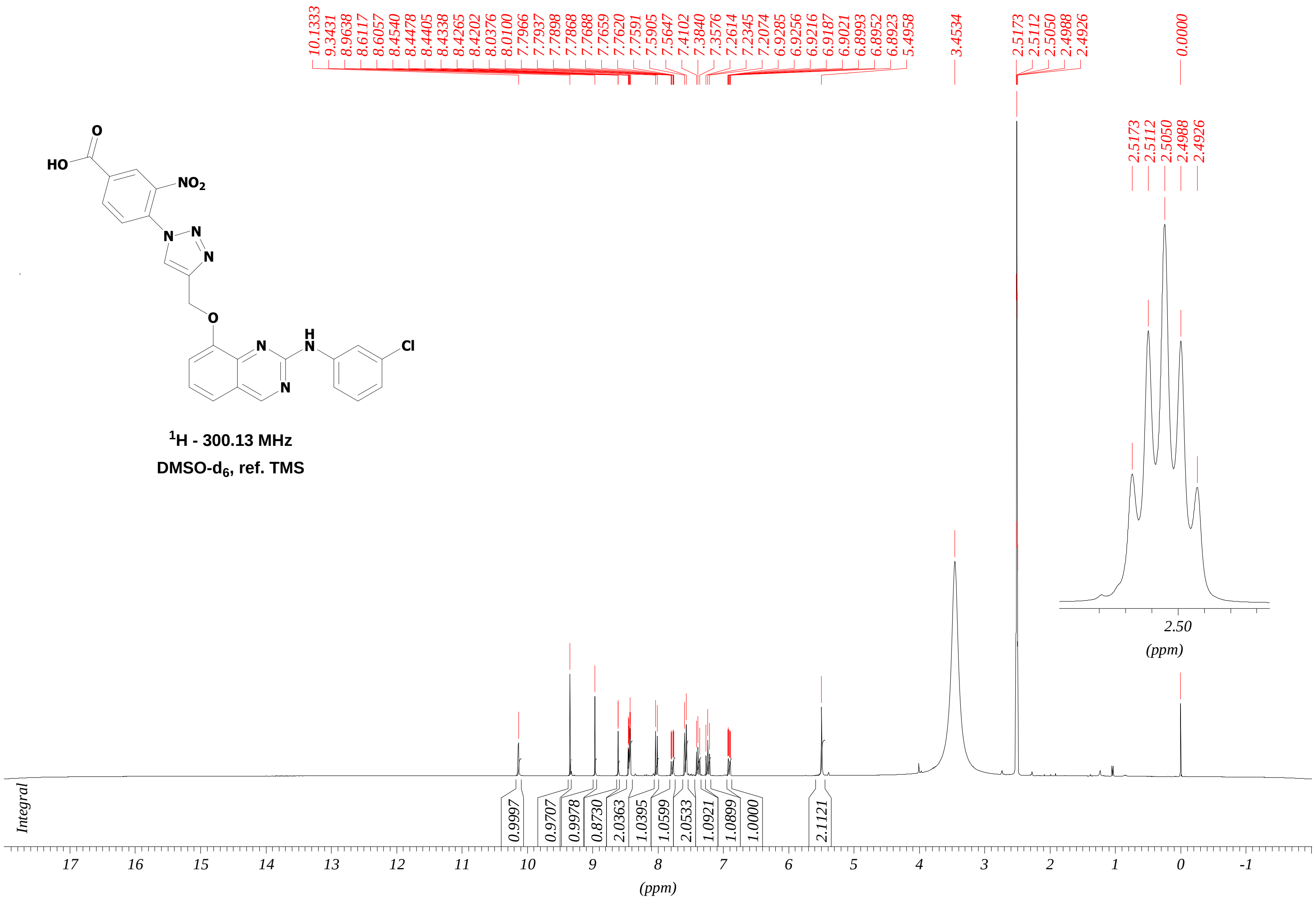

HO
O
NO2
N
N
N
O
N
H
N
Cl
N
1H - 300.13 MHz
DMSO-d6, ref. TMS
10.1333
9.3431
8.9638
8.6117
8.6057
8.4540
8.4478
8.4405
8.4338
8.4265
8.4202
8.0376
8.0100
7.7966
7.7937
7.7898
7.7868
7.7688
7.7659
7.7620
7.7591
7.5905
7.5647
7.4102
7.3840
7.3576
7.2614
7.2345
7.2074
6.9285
6.9256
6.9216
6.9187
6.9021
6.8993
6.8952
6.8923
5.4958
3.4534
2.5173
2.5112
2.5050
2.4988
2.4926
0.0000
2.50
(ppm)
Integral
0.9997
0.9707
0.9978
0.8730
2.0363
1.0395
1.0599
2.0533
1.0921
1.0899
1.0000
2.1121
17 16 15 14 13 12 11 10 9 8 7 6 5 4 3 2 1 0 -1
(ppm)

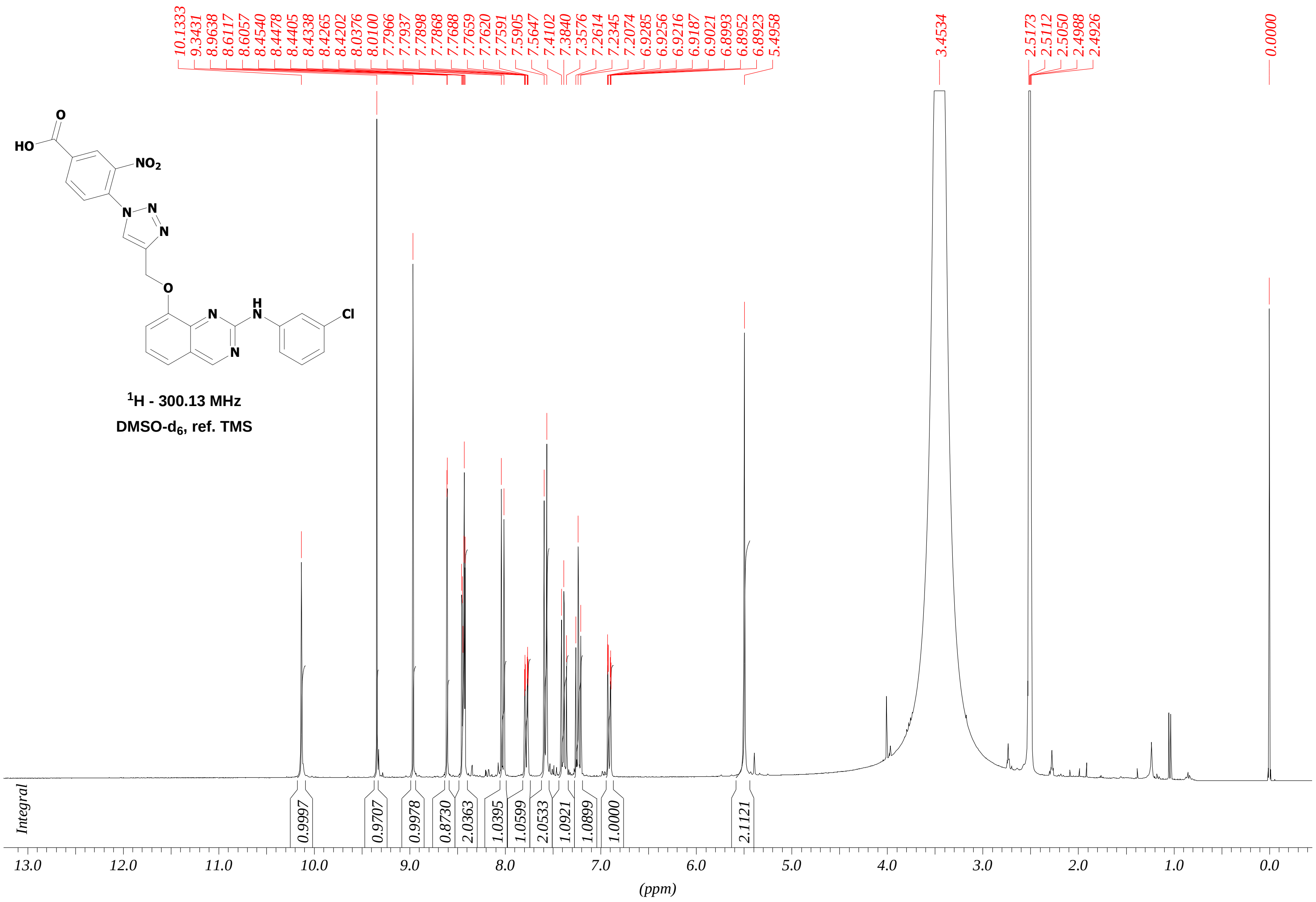
1H - 300.13 MHz
DMSO-d6, ref. TMS
10.1333 9.3431 8.9638 8.6117 8.6057 8.4540 8.4478 8.4405 8.4338 8.4265 8.4202 8.0376 8.0100 7.7966 7.7937 7.7898 7.7868 7.7688 7.7659 7.7620 7.7591 7.5905 7.5647 7.4102 7.3840 7.3576 7.2614 7.2345 7.2074 6.9285 6.9256 6.9216 6.9187 6.9021 6.8993 6.8952 6.8923 5.4958
3.4534
2.5173 2.5112 2.5050 2.4988 2.4926
0.0000
Integral
0.9997 0.9707 0.9978 0.8730 2.0363 1.0395 1.0599 2.0533 1.0921 1.0899 1.0000 2.1121
13.0 12.0 11.0 10.0 9.0 8.0 7.0 6.0 5.0 4.0 3.0 2.0 1.0 0.0
(ppm)

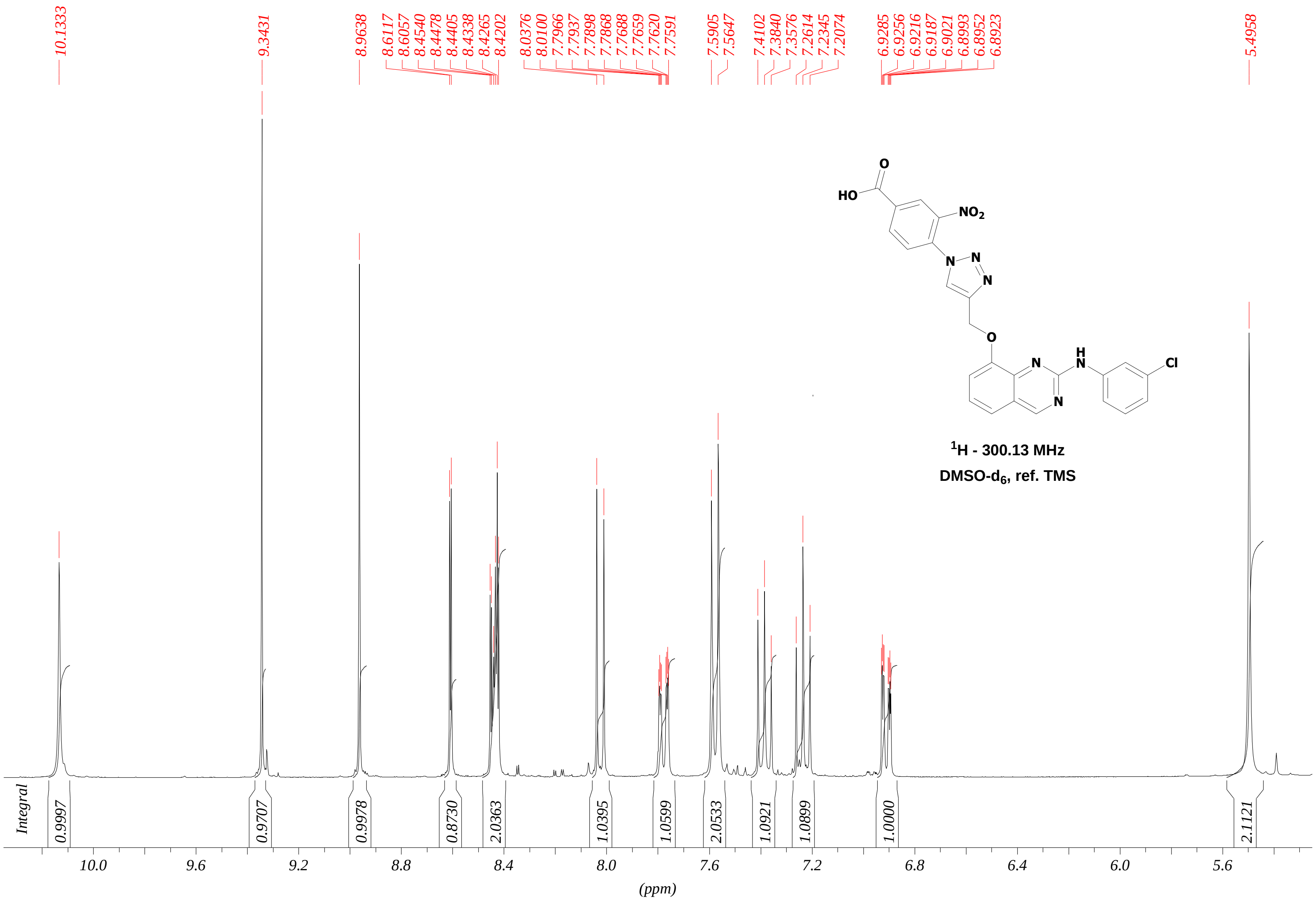

10.1333
9.3431
8.9638
8.6117
8.6057
8.4540
8.4478
8.4405
8.4338
8.4265
8.4202
8.0376
8.0100
7.7966
7.7937
7.7898
7.7868
7.7688
7.7659
7.7620
7.7591
7.5905
7.5647
7.4102
7.3840
7.3576
7.2614
7.2345
7.2074
6.9285
6.9256
6.9216
6.9187
6.9021
6.8993
6.8952
6.8923
5.4958
HO
O
NO2
N
N
N
O
N
H
N
Cl
N
1H - 300.13 MHz
DMSO-d6, ref. TMS
Integral
0.9997
0.9707
0.9978
0.8730
2.0363
1.0395
1.0599
2.0533
1.0921
1.0899
1.0000
2.1121
10.0
9.6
9.2
8.8
8.4
8.0
7.6
7.2
6.8
6.4
6.0
5.6
(ppm)

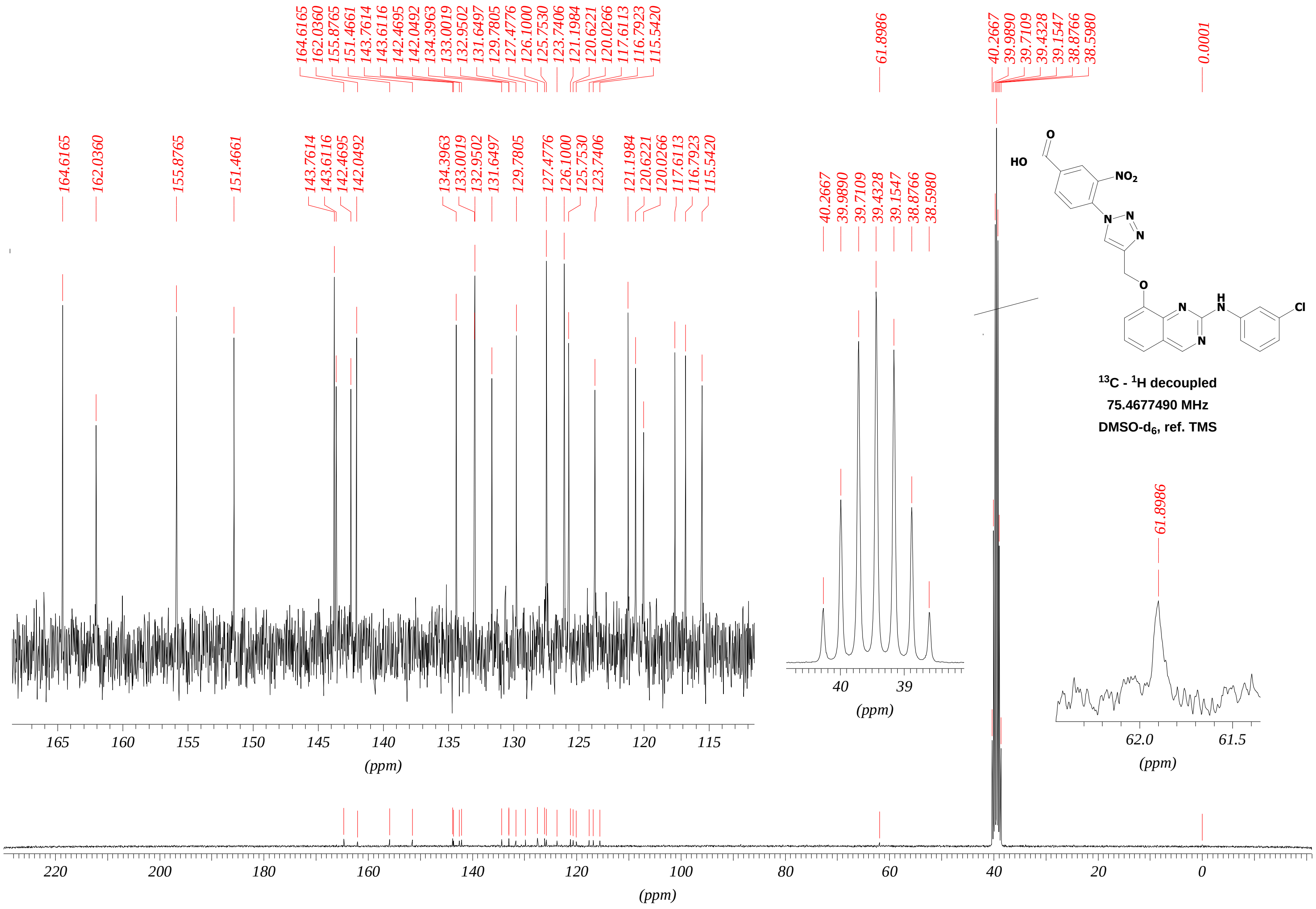

164.6165
162.0360
155.8765
151.4661
143.7614
143.6116
142.4695
142.0492
134.3963
133.0019
132.9502
131.6497
129.7805
127.4776
126.1000
125.7530
123.7406
121.1984
120.6221
120.0266
117.6113
116.7923
115.5420
61.8986
40.2667
39.9890
39.7109
39.4328
39.1547
38.8766
38.5980
0.0001
HO
O
NO2
N
N
N
O
N
H
N
Cl
N
13C - 1H decoupled
75.4677490 MHz
DMSO-d6, ref. TMS
(ppm)

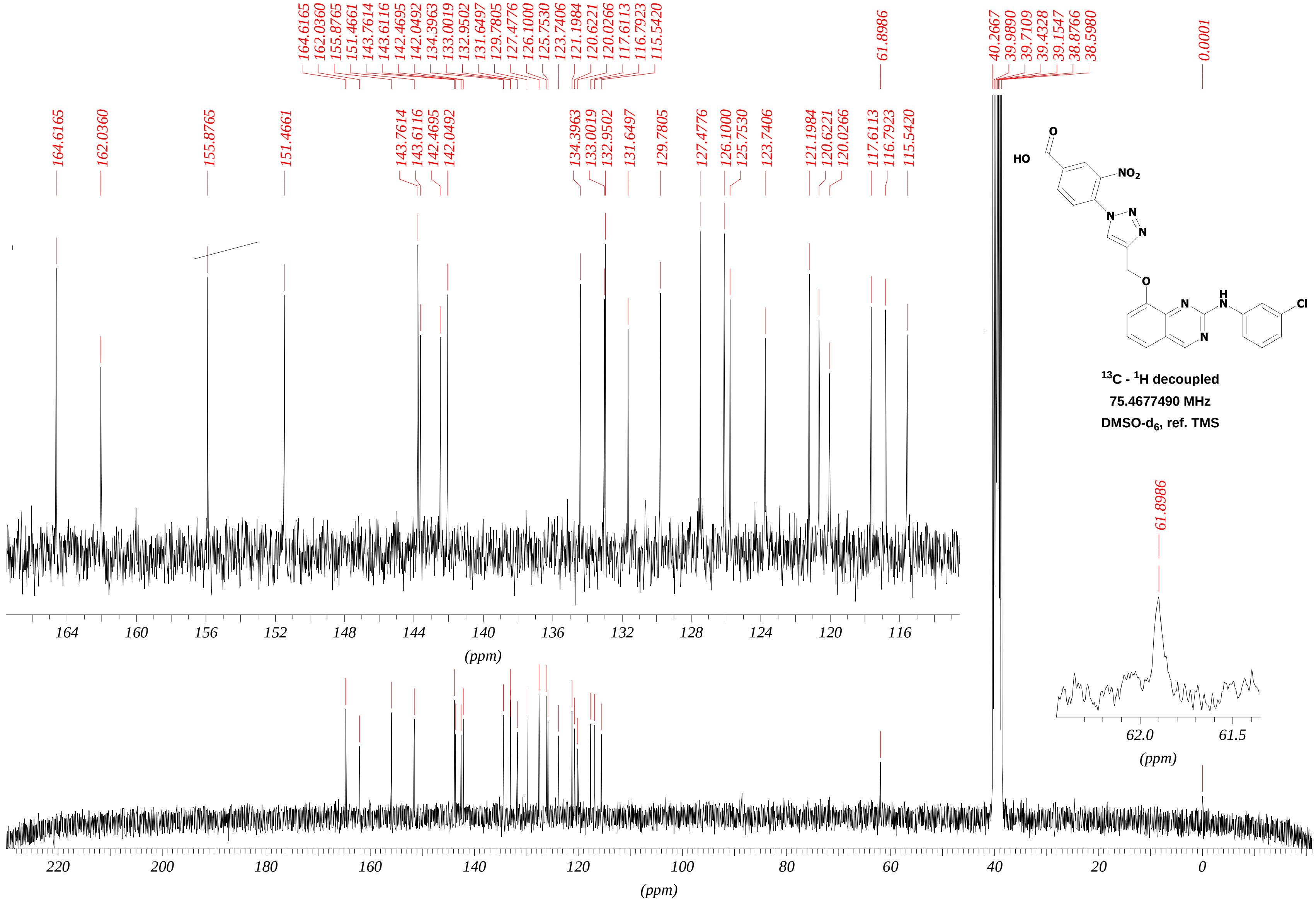
164.6165
162.0360
155.8765
151.4661
143.7614
143.6116
142.4695
142.0492
134.3963
133.0019
132.9502
131.6497
129.7805
127.4776
126.1000
125.7530
123.7406
121.1984
120.6221
120.0266
117.6113
116.7923
115.5420
61.8986
40.2667
39.9890
39.7109
39.4328
39.1547
38.8766
38.5980
0.0001
HO
O
NO2
N
N
N
O
N
H
N
Cl
N
13C - 1H decoupled
75.4677490 MHz
DMSO-d6, ref. TMS
(ppm)
(ppm)
(ppm)

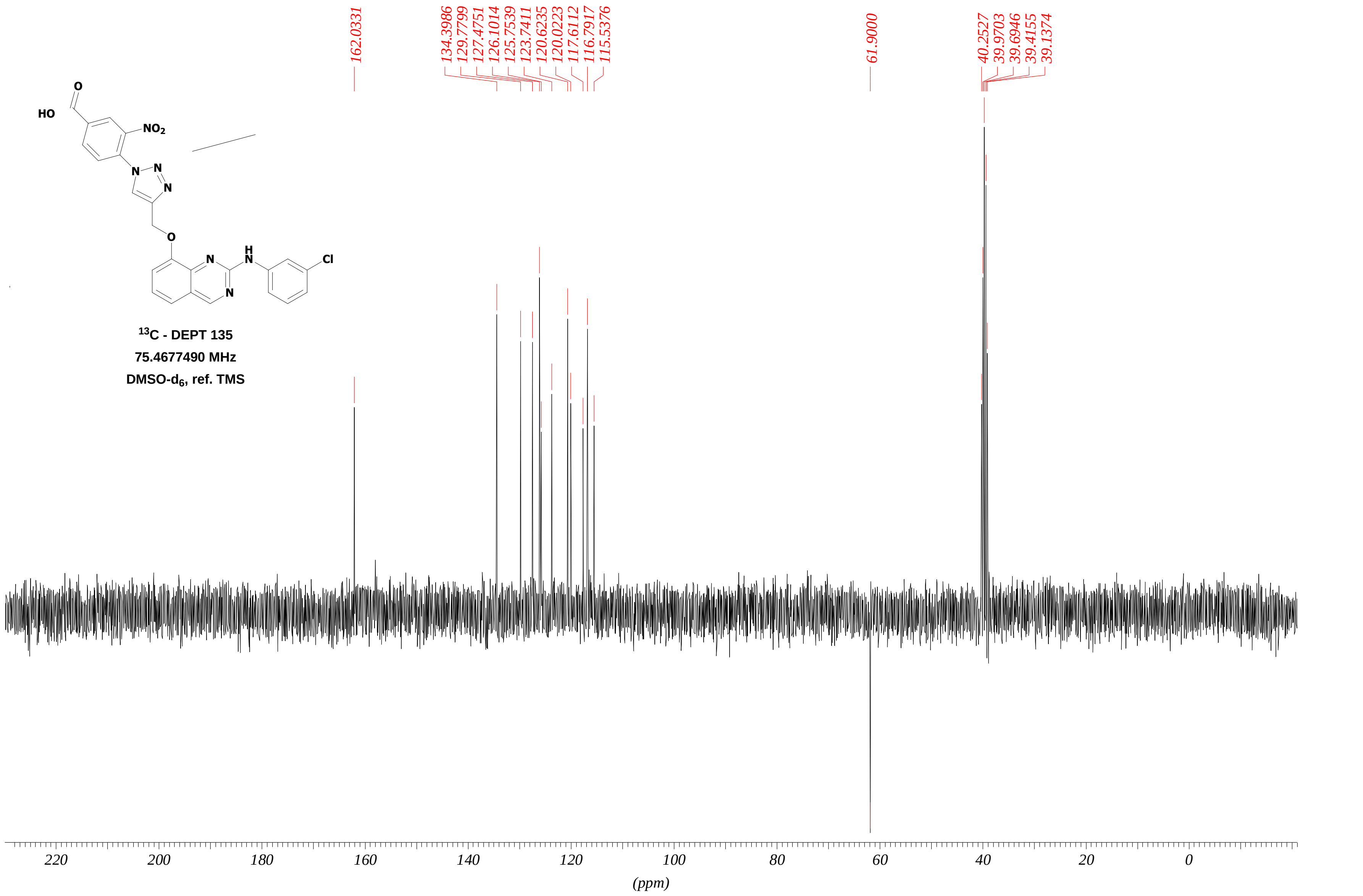

162.0331
134.3986
129.7799
127.4751
126.1014
125.7539
123.7411
120.6235
120.0223
117.6112
116.7917
115.5376
61.9000
40.2527
39.9703
39.6946
39.4155
39.1374
HO
O
NO_2
N
N
N
O
N
N
H
N
Cl
^{13}C - DEPT 135
75.4677490 MHz
DMSO-d_6, ref. TMS
220
200
180
160
140
120
100
80
60
40
20
0
(ppm)

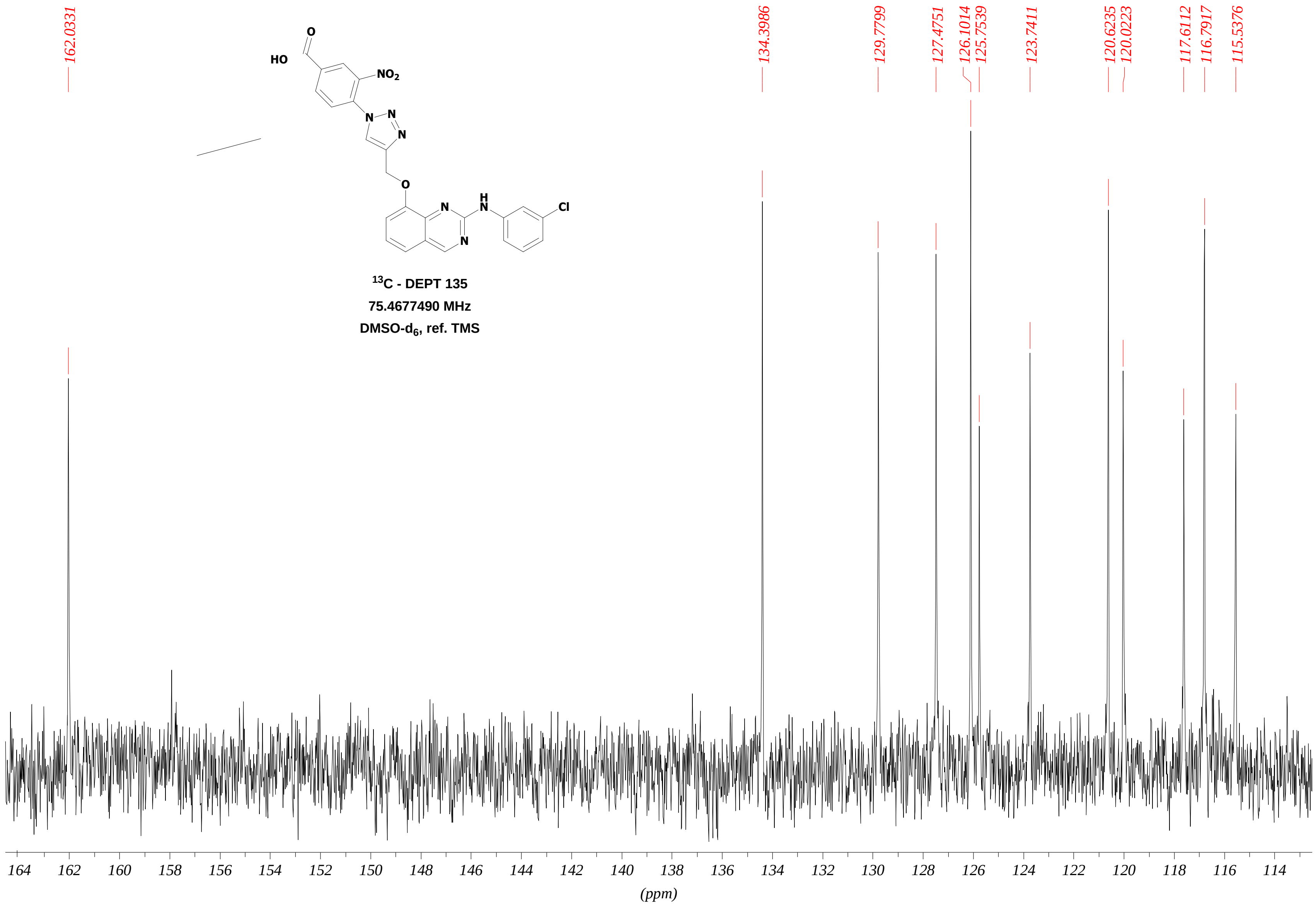

162.0331
134.3986
129.7799
127.4751
126.1014
125.7539
123.7411
120.6235
120.0223
117.6112
116.7917
115.5376
HO
O
NO2
N
N
N
O
N
H
N
Cl
N
13C - DEPT 135
75.4677490 MHz
DMSO-d6, ref. TMS
164
162
160
158
156
154
152
150
148
146
144
142
140
138
136
134
132
130
128
126
124
122
120
118
116
114
(ppm)

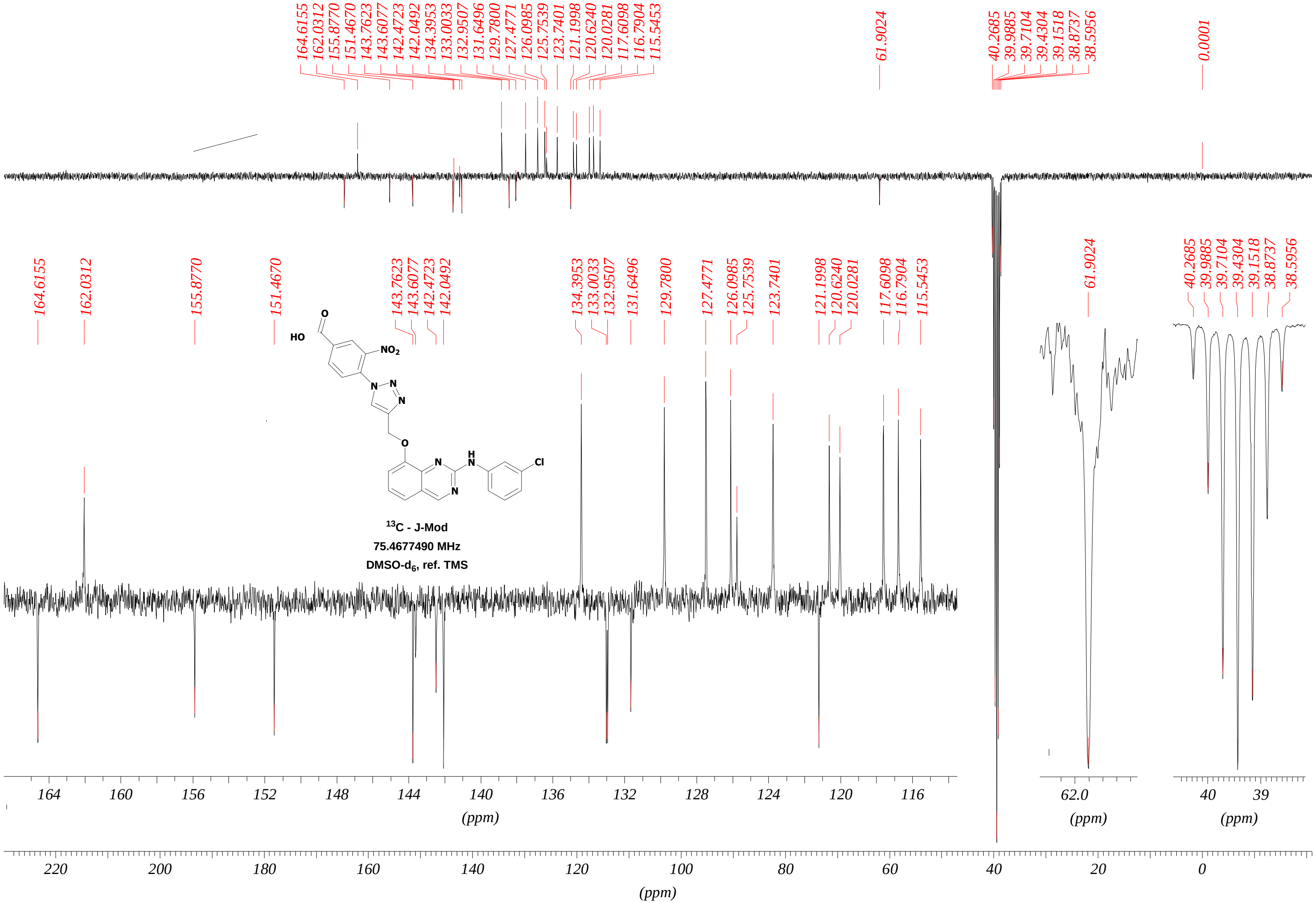
164.6155
162.0312
155.8770
151.4670
143.7623
143.6077
142.4723
142.0492
134.3953
133.0033
132.9507
131.6496
129.7800
127.4771
126.0985
125.7539
123.7401
121.1998
120.6240
120.0281
117.6098
116.7904
115.5453
61.9024
40.2685
39.9885
39.7104
39.4304
39.1518
38.8737
38.5956
0.0001
HO
O
NO_2
N
N
N
O
N
N
H
N
Cl
^{13}C - J-Mod
75.4677490 MHz
DMSO-d_6, ref. TMS
(ppm)

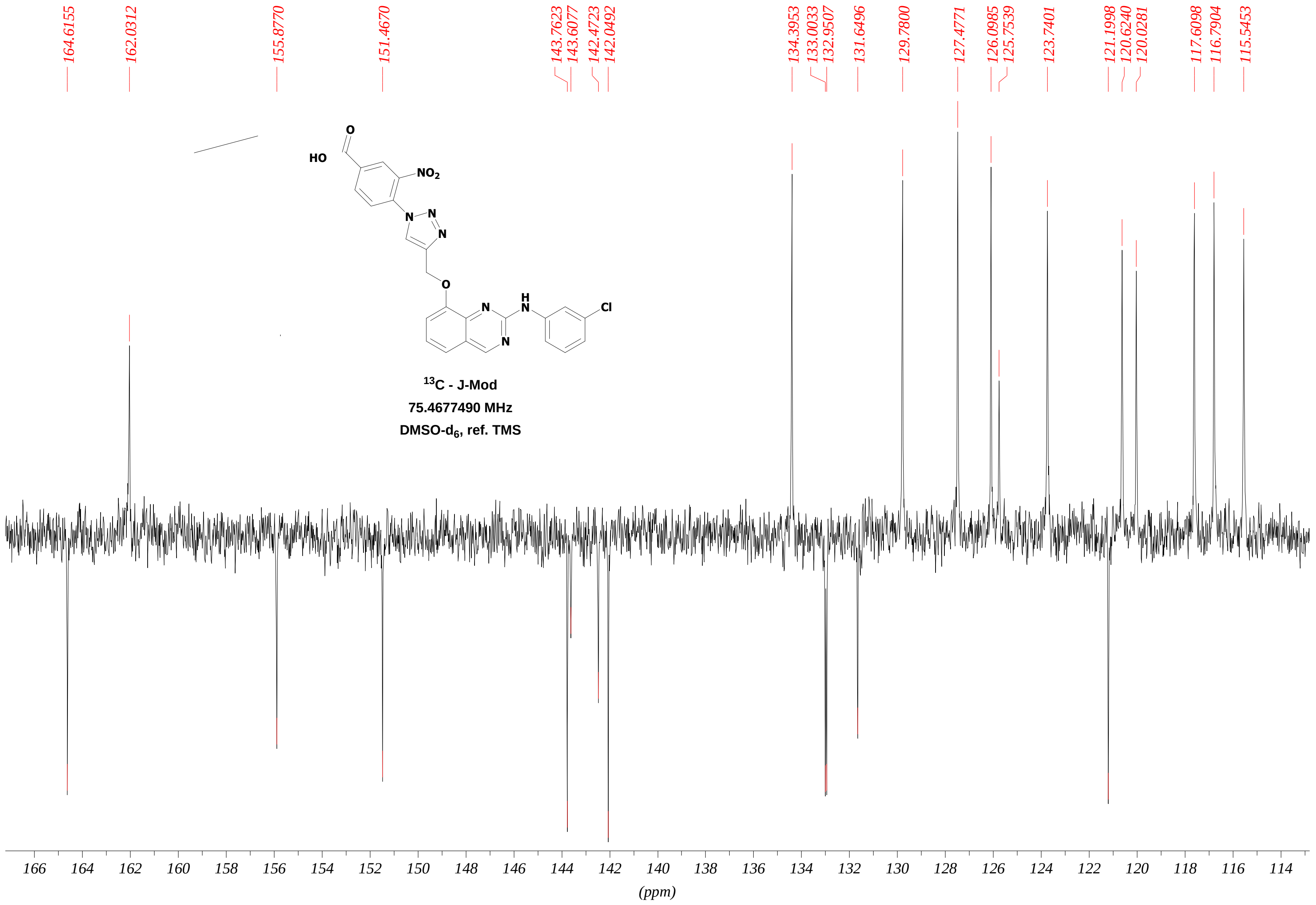
164.6155
162.0312
155.8770
151.4670
143.7623
143.6077
142.4723
142.0492
134.3953
133.0033
132.9507
131.6496
129.7800
127.4771
126.0985
125.7539
123.7401
121.1998
120.6240
120.0281
117.6098
116.7904
115.5453
HO
O
NO_2
N
N
N
O
N
N
H
N
Cl
^{13}C - J-Mod
75.4677490 MHz
DMSO-d_6, ref. TMS
166
164
162
160
158
156
154
152
150
148
146
144
142
140
138
136
134
132
130
128
126
124
122
120
118
116
114
(ppm)

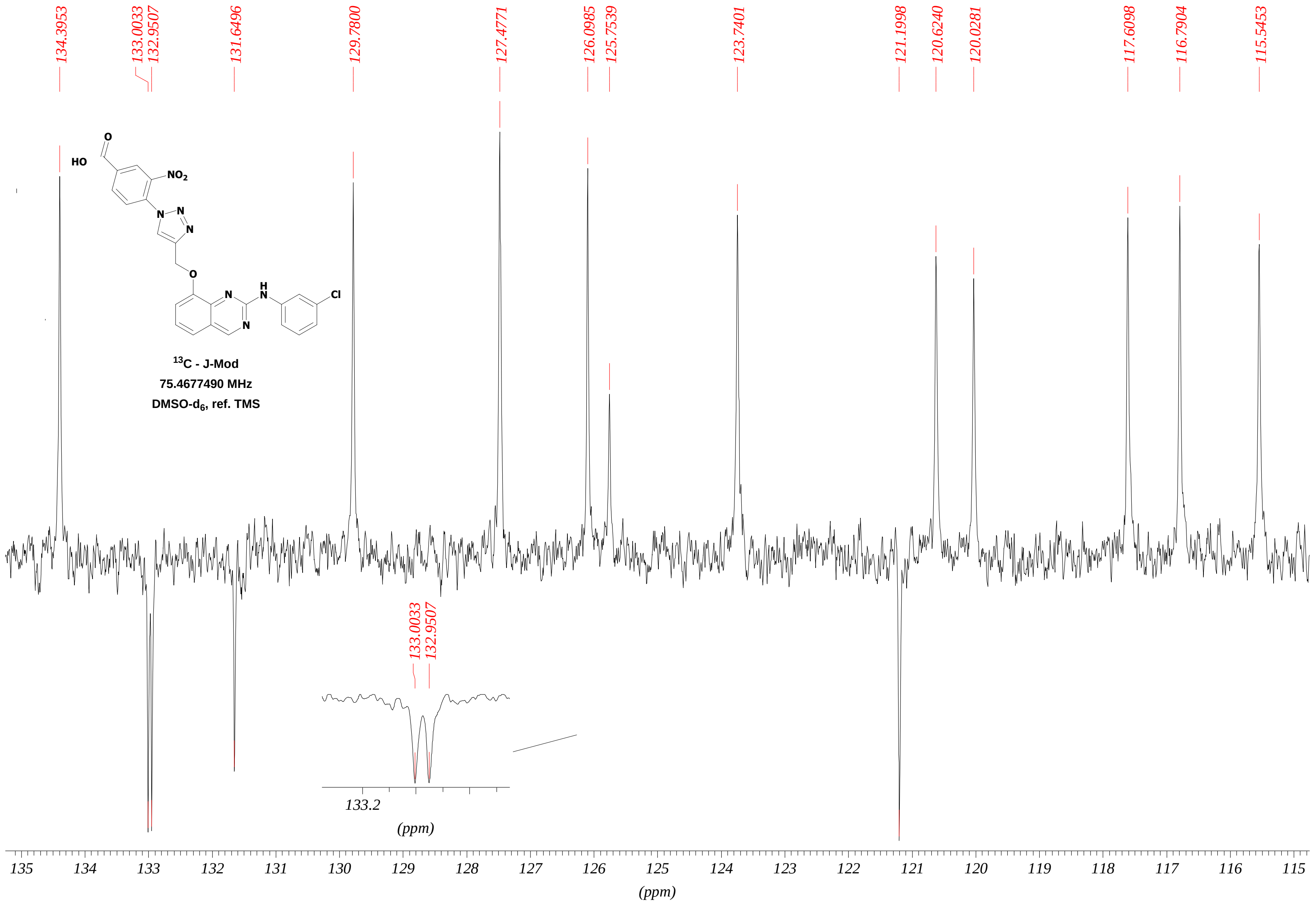
134.3953
133.0033
132.9507
131.6496
129.7800
127.4771
126.0985
125.7539
123.7401
121.1998
120.6240
120.0281
117.6098
116.7904
115.5453
HO
O
NO2
N
N
N
O
N
H
N
Cl
N
13C - J-Mod
75.4677490 MHz
DMSO-d6, ref. TMS
133.2
(ppm)
135
134
133
132
131
130
129
128
127
126
125
124
123
122
121
120
119
118
117
116
115
(ppm)

***tert*-Butyl ((*cis*)-4-(4-(((2-((3-chlorophenyl)amino)quinazolin-8-yl)oxy)methyl)-1*H*-1,2,3-triazol-1-yl)cyclohexyl)carbamate (15a):**

Pages S113-S123

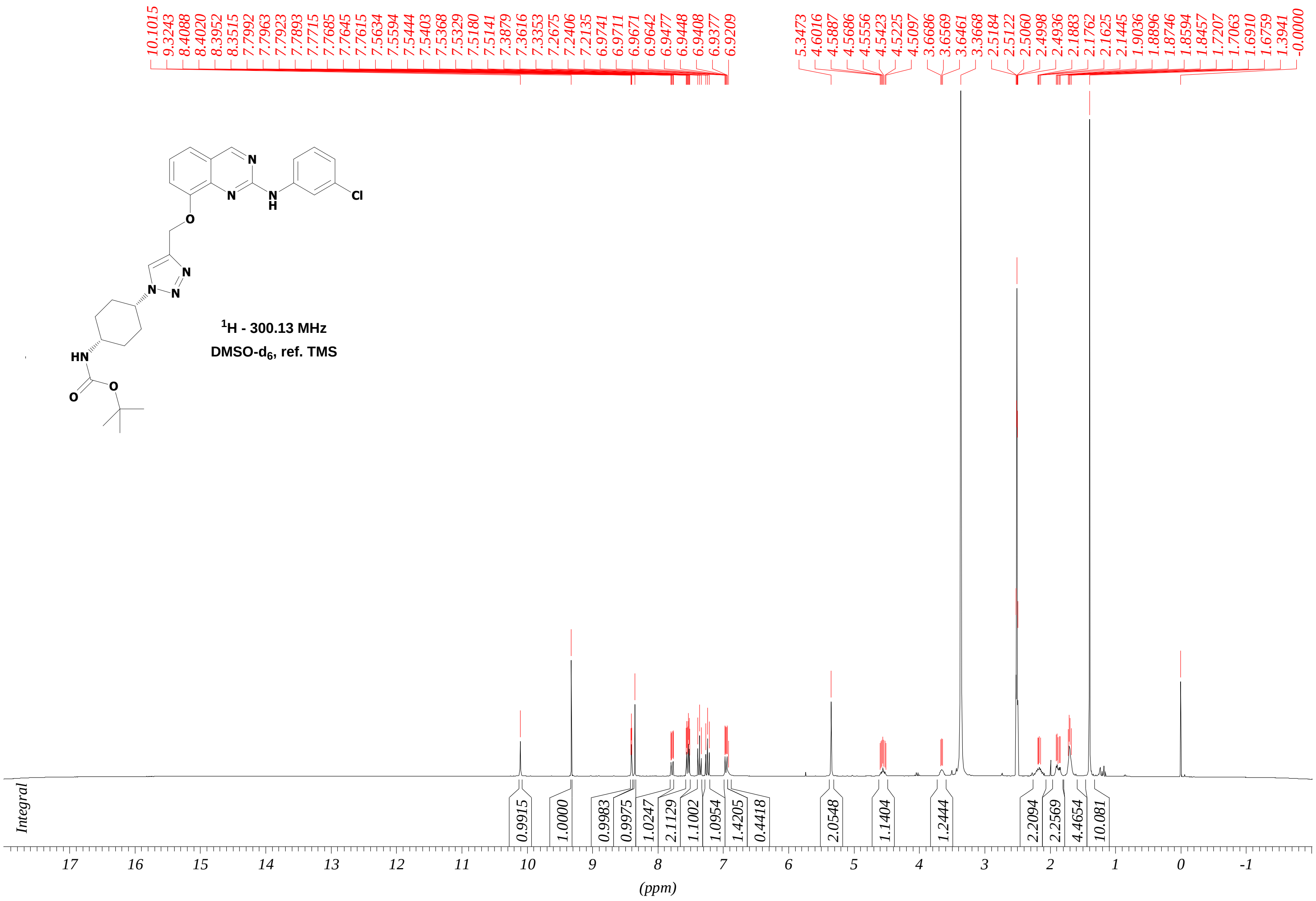

^{1}H - 300.13 MHz
DMSO-d_6, ref. TMS
10.1015 9.3243 8.4088 8.4020 8.3952 8.3515 7.7992 7.7963 7.7923 7.7893 7.7715 7.7685 7.7645 7.7615 7.5634 7.5594 7.5444 7.5403 7.5368 7.5329 7.5180 7.5141 7.3879 7.3616 7.3353 7.2675 7.2406 7.2135 6.9741 6.9711 6.9671 6.9642 6.9477 6.9448 6.9408 6.9377 6.9209
5.3473 4.6016 4.5887 4.5686 4.5556 4.5423 4.5225 4.5097 3.6686 3.6569 3.6461 3.3668 2.5184 2.5122 2.5060 2.4998 2.4936 2.1883 2.1762 2.1625 2.1445 1.9036 1.8896 1.8746 1.8594 1.8457 1.7207 1.7063 1.6910 1.6759 1.3941 -0.0000
Integral
0.9915 1.0000 0.9983 0.9975 1.0247 2.1129 1.1002 1.0954 1.4205 0.4418 2.0548 1.1404 1.2444 2.2094 2.2569 4.4654 10.081
17 16 15 14 13 12 11 10 9 8 7 6 5 4 3 2 1 0 -1
(ppm)

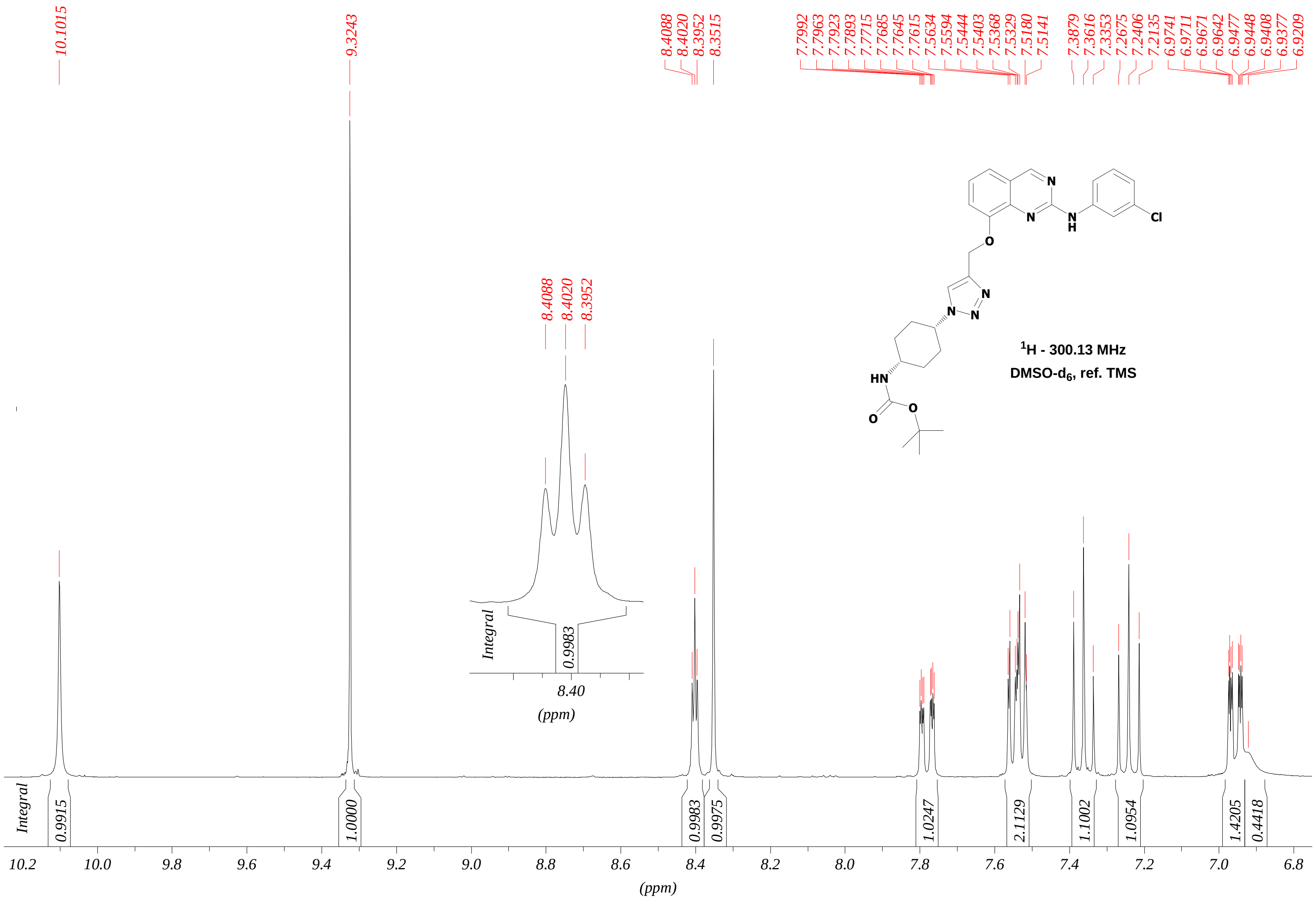

10.1015
9.3243
8.4088
8.4020
8.3952
8.3515
7.7992
7.7963
7.7923
7.7893
7.7715
7.7685
7.7645
7.7615
7.5634
7.5594
7.5444
7.5403
7.5368
7.5329
7.5180
7.5141
7.3879
7.3616
7.3353
7.2675
7.2406
7.2135
6.9741
6.9711
6.9671
6.9642
6.9477
6.9448
6.9408
6.9377
6.9209
N
N
N
H
Cl
O
N
N
N
HN
O
O
^{1}H - 300.13 MHz
DMSO-d$_6$, ref. TMS
Integral
0.9983
8.40
(ppm)
Integral
0.9915
1.0000
0.9983
0.9975
1.0247
2.1129
1.1002
1.0954
1.4205
0.4418
10.2
10.0
9.8
9.6
9.4
9.2
9.0
8.8
8.6
8.4
8.2
8.0
7.8
7.6
7.4
7.2
7.0
6.8
(ppm)

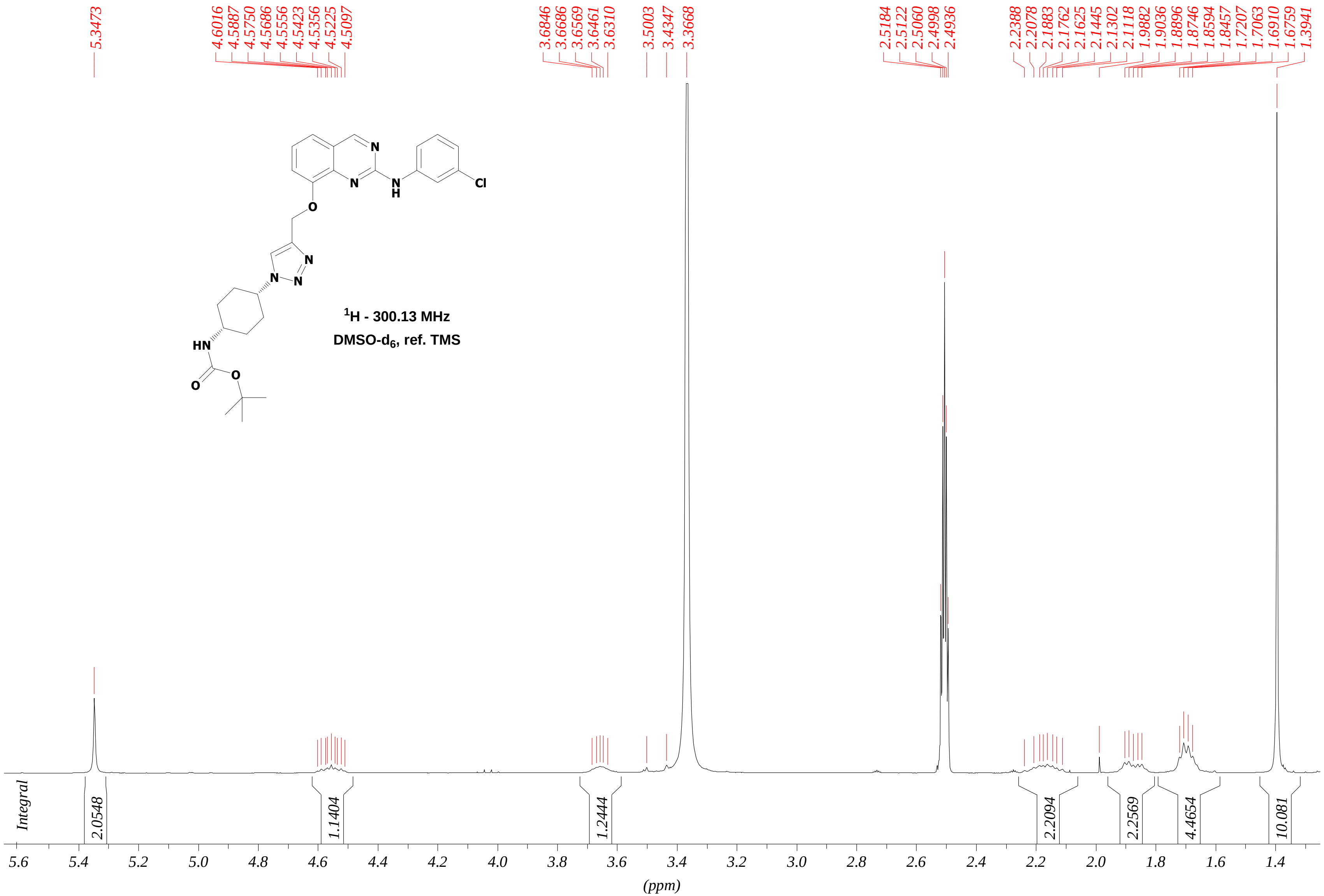
5.3473
4.6016
4.5887
4.5750
4.5686
4.5556
4.5423
4.5356
4.5225
4.5097
3.6846
3.6686
3.6569
3.6461
3.6310
3.5003
3.4347
3.3668
2.5184
2.5122
2.5060
2.4998
2.4936
2.2388
2.2078
2.1883
2.1762
2.1625
2.1445
2.1302
2.1118
1.9882
1.9036
1.8896
1.8746
1.8594
1.8457
1.7207
1.7063
1.6910
1.6759
1.3941
N
N
N
H
Cl
O
N
N
N
HN
O
O
1H - 300.13 MHz
DMSO-d6, ref. TMS
Integral
2.0548
1.1404
1.2444
2.2094
2.2569
4.4654
10.081
5.6
5.4
5.2
5.0
4.8
4.6
4.4
4.2
4.0
3.8
3.6
3.4
3.2
3.0
2.8
2.6
2.4
2.2
2.0
1.8
1.6
1.4
(ppm)

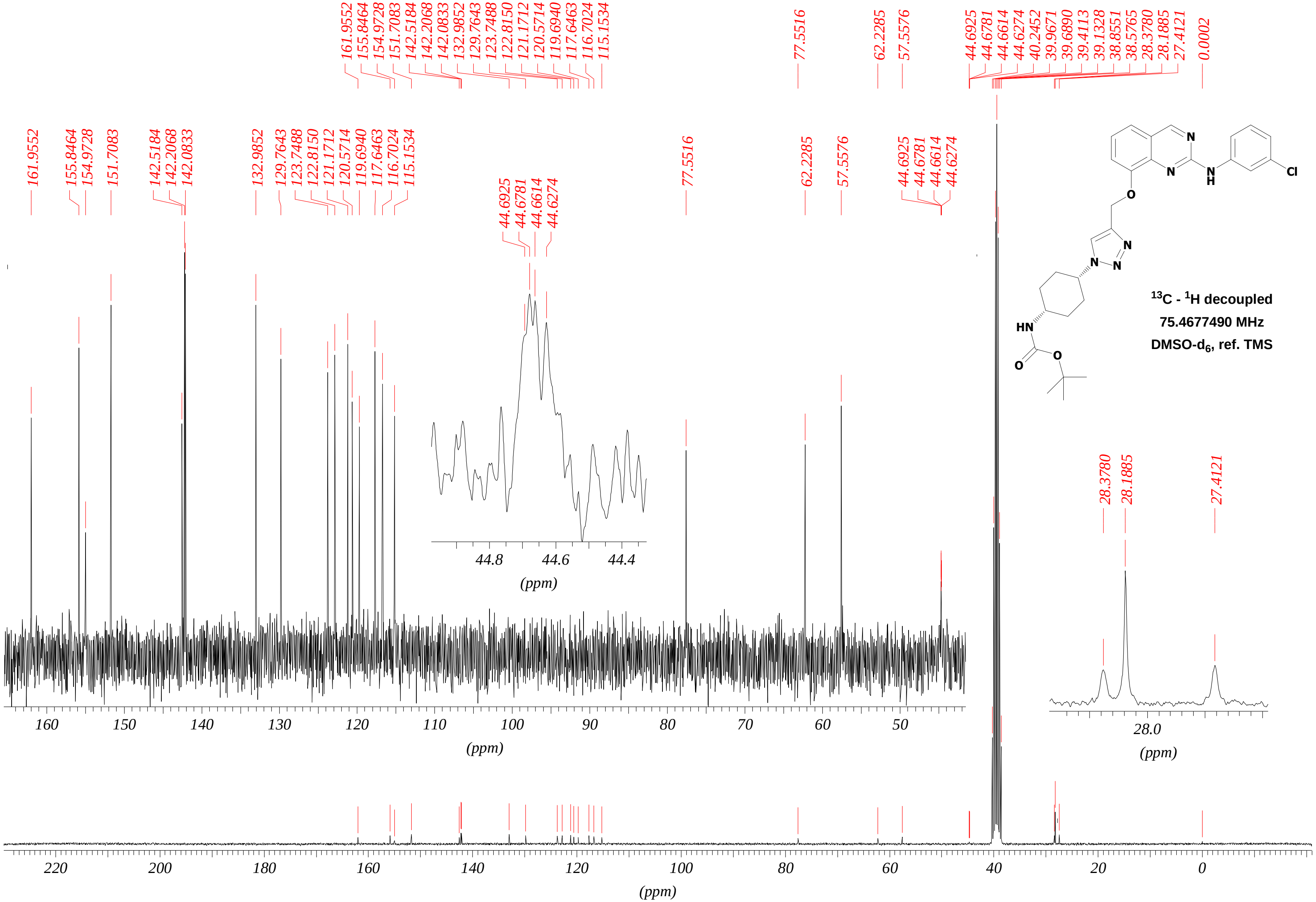
13C - 1H decoupled
75.4677490 MHz
DMSO-d6, ref. TMS
161.9552
155.8464
154.9728
151.7083
142.5184
142.2068
142.0833
132.9852
129.7643
123.7488
122.8150
121.1712
120.5714
119.6940
117.6463
116.7024
115.1534
77.5516
62.2285
57.5576
44.6925
44.6781
44.6614
44.6274
40.2452
39.9671
39.6890
39.4113
39.1328
38.8551
38.5765
28.3780
28.1885
27.4121
0.0002
(ppm)

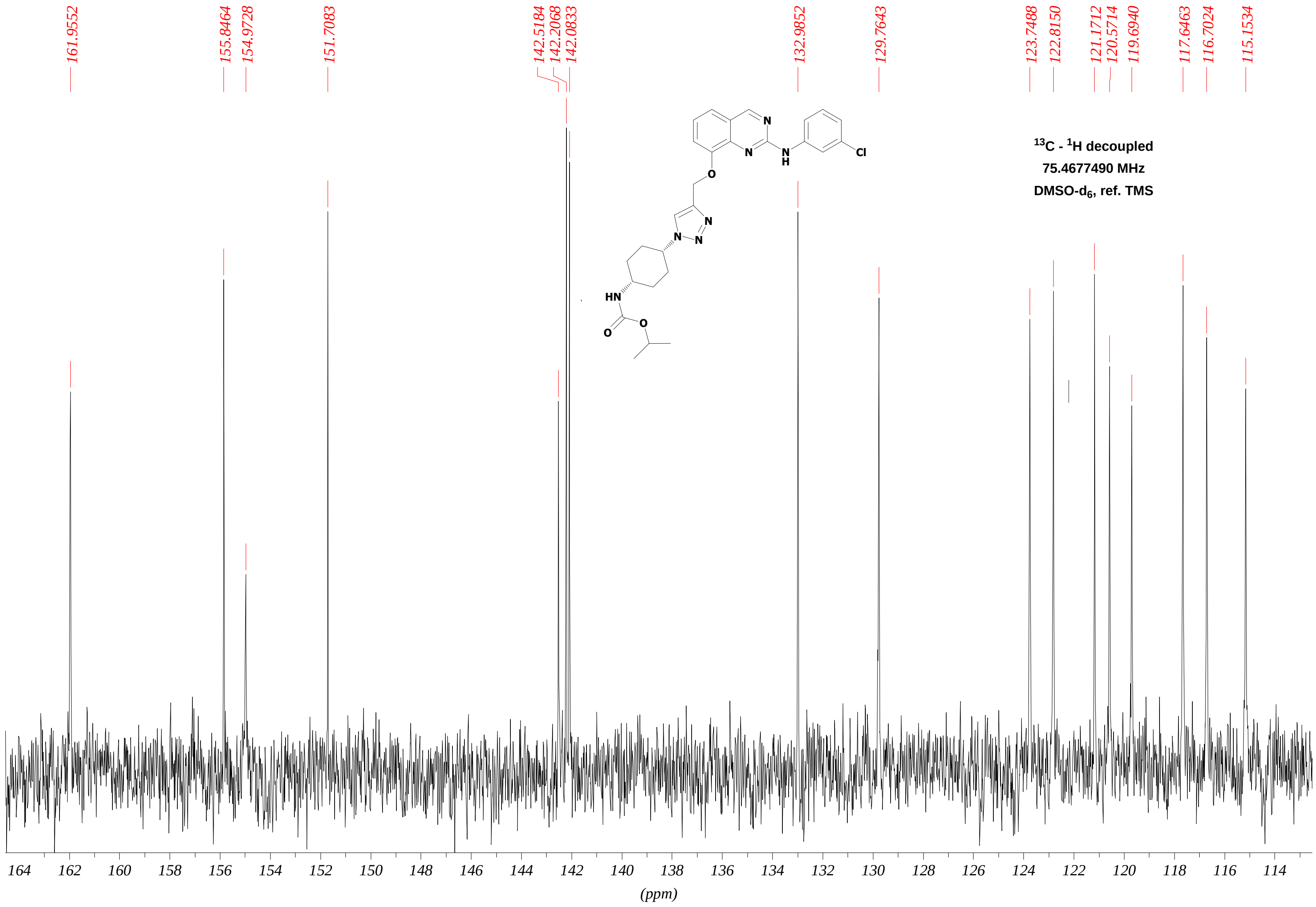

161.9552
155.8464
154.9728
151.7083
142.5184
142.2068
142.0833
132.9852
129.7643
123.7488
122.8150
121.1712
120.5714
119.6940
117.6463
116.7024
115.1534
^{13}C - ^{1}H decoupled
75.4677490 MHz
DMSO-d$_6$, ref. TMS
N
N
N
H
Cl
O
N
N
N
HN
O
O
164 162 160 158 156 154 152 150 148 146 144 142 140 138 136 134 132 130 128 126 124 122 120 118 116 114
(ppm)

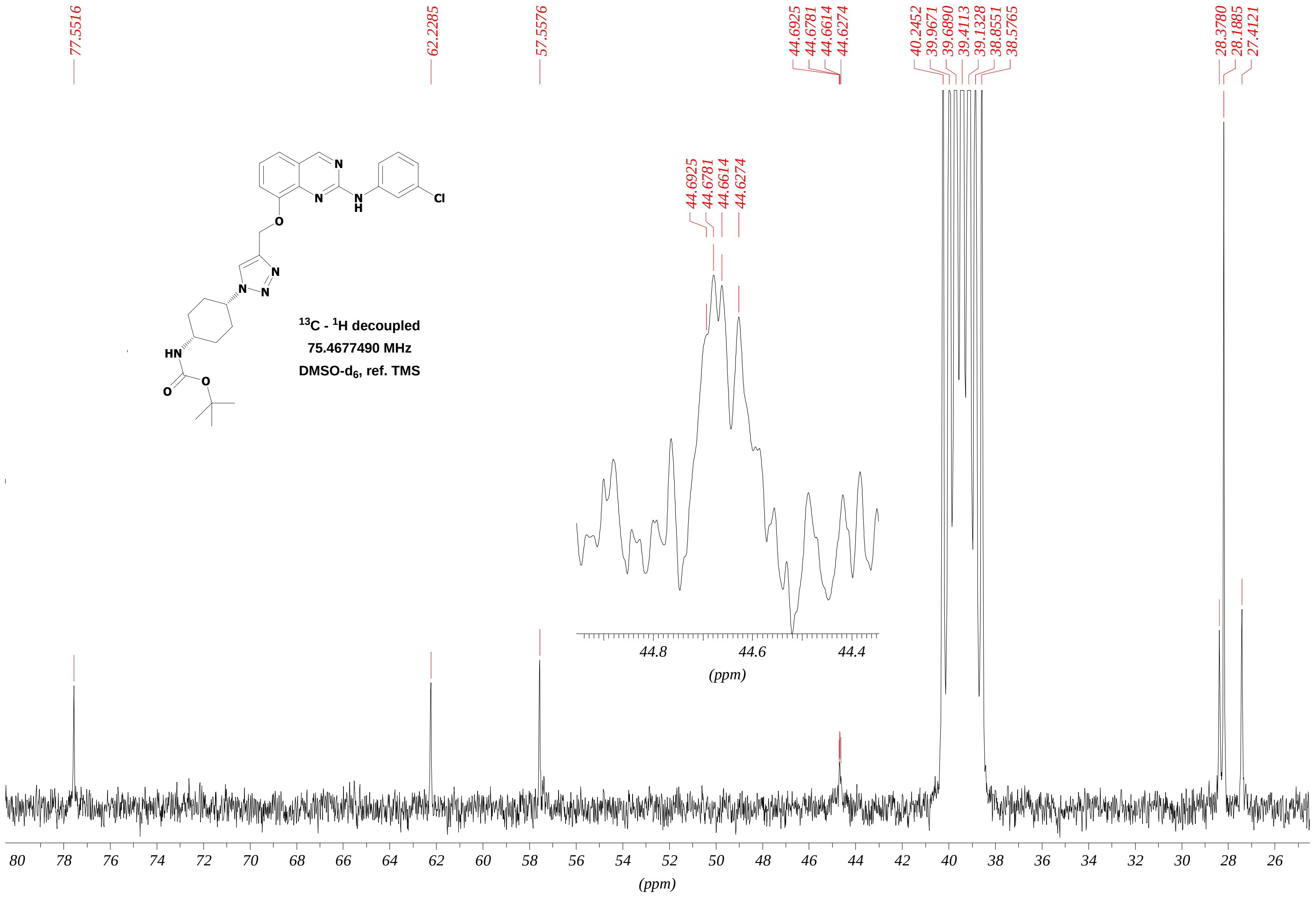
77.5516
62.2285
57.5576
44.6925
44.6781
44.6614
44.6274
40.2452
39.9671
39.6890
39.4113
39.1328
38.8551
38.5765
28.3780
28.1885
27.4121
^{13}C - ^{1}H decoupled
75.4677490 MHz
DMSO-d$_6$, ref. TMS
N
N
N
H
Cl
O
N
N
N
HN
O
O
(ppm)

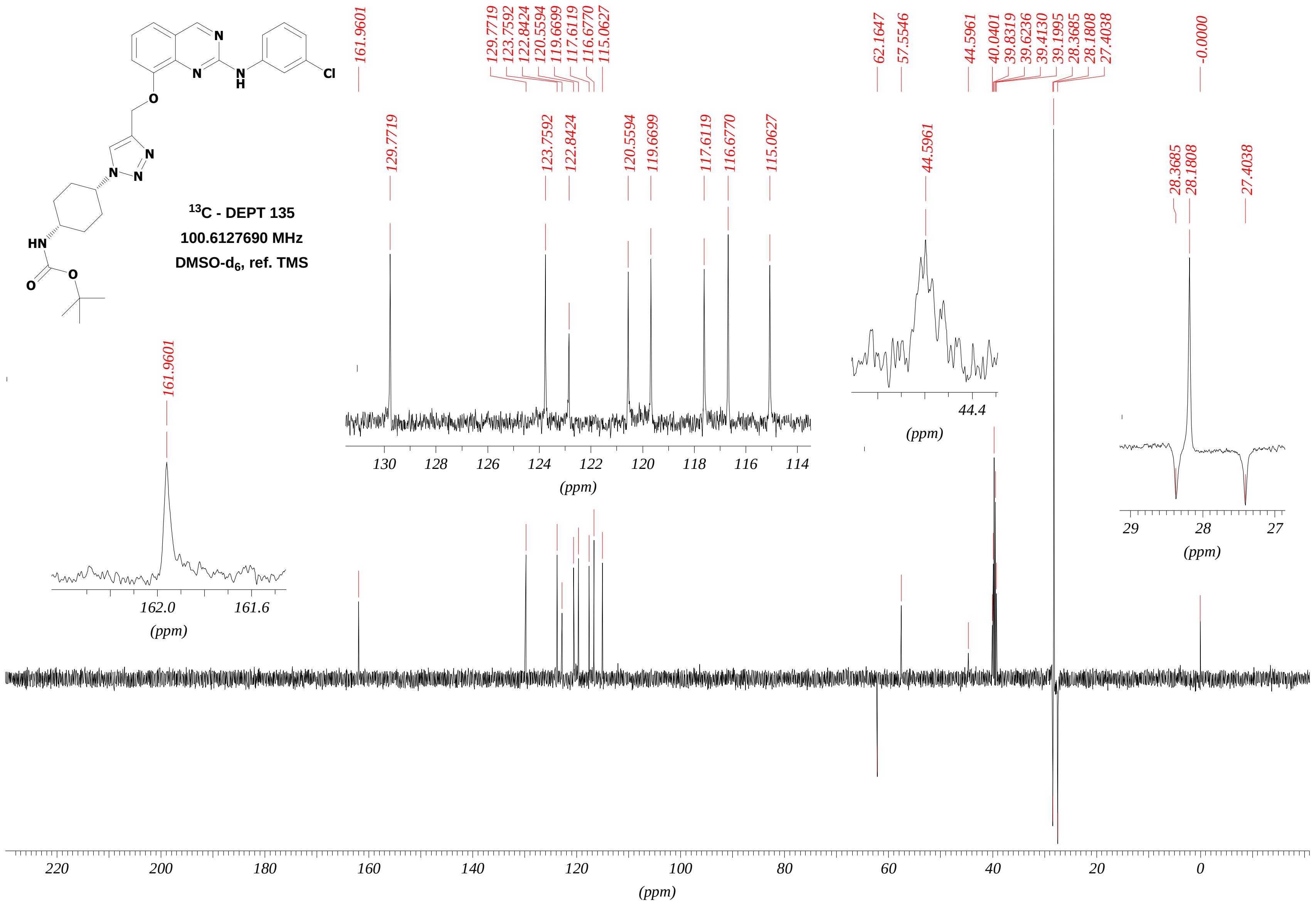

^{13}C - DEPT 135
100.6127690 MHz
DMSO-d$_6$, ref. TMS
161.9601
129.7719
123.7592
122.8424
120.5594
119.6699
117.6119
116.6770
115.0627
62.1647
57.5546
44.5961
40.0401
39.8319
39.6236
39.4130
39.1995
28.3685
28.1808
27.4038
-0.0000
(ppm)

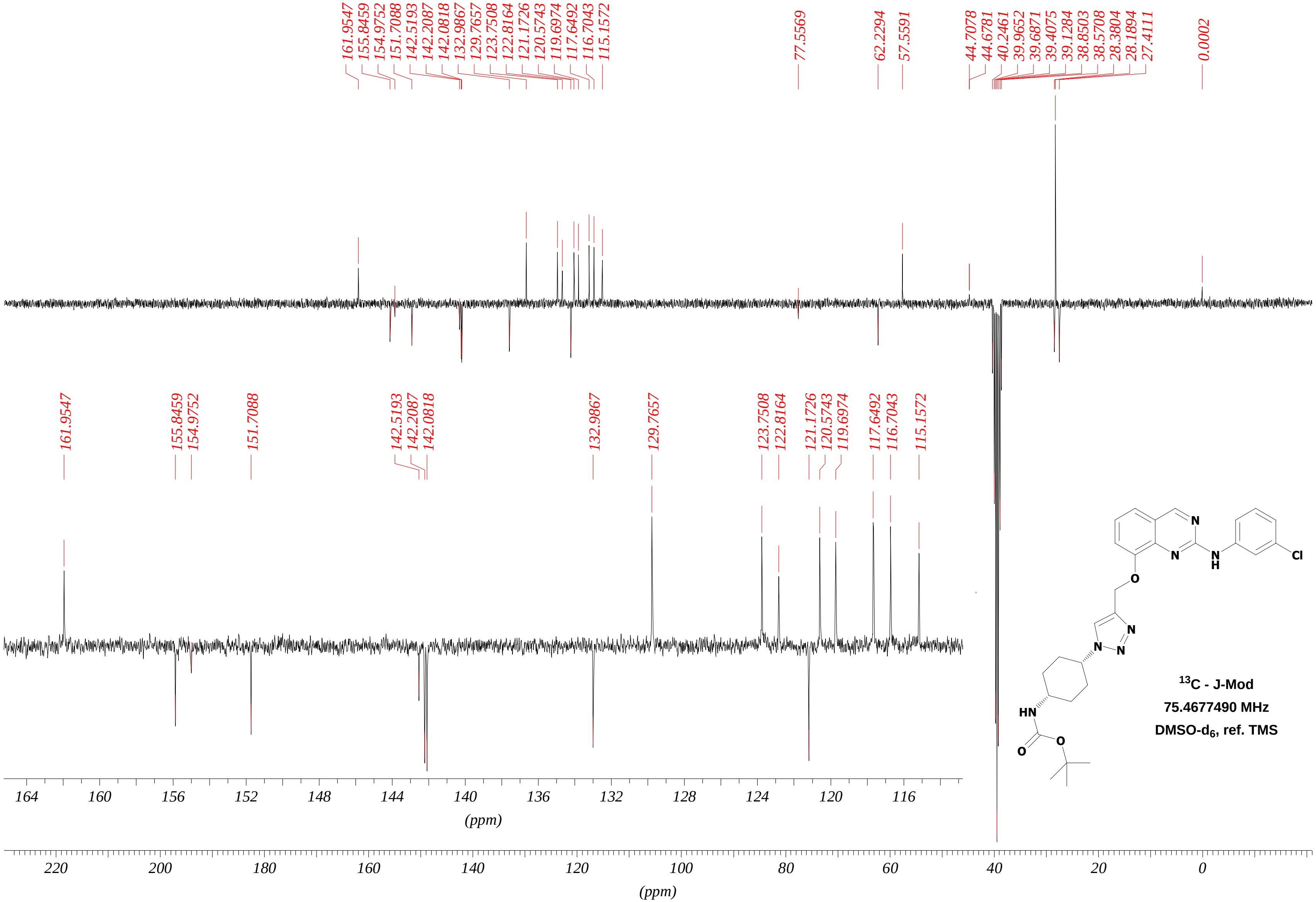
161.9547
155.8459
154.9752
151.7088
142.5193
142.2087
142.0818
132.9867
129.7657
123.7508
122.8164
121.1726
120.5743
119.6974
117.6492
116.7043
115.1572
77.5569
62.2294
57.5591
44.7078
44.6781
40.2461
39.9652
39.6871
39.4075
39.1284
38.8503
38.5708
28.3804
28.1894
27.4111
0.0002
(ppm)
(ppm)
N
N
N
H
Cl
O
N
N
N
HN
O
O
^{13}C - J-Mod
75.4677490 MHz
DMSO-d_6, ref. TMS

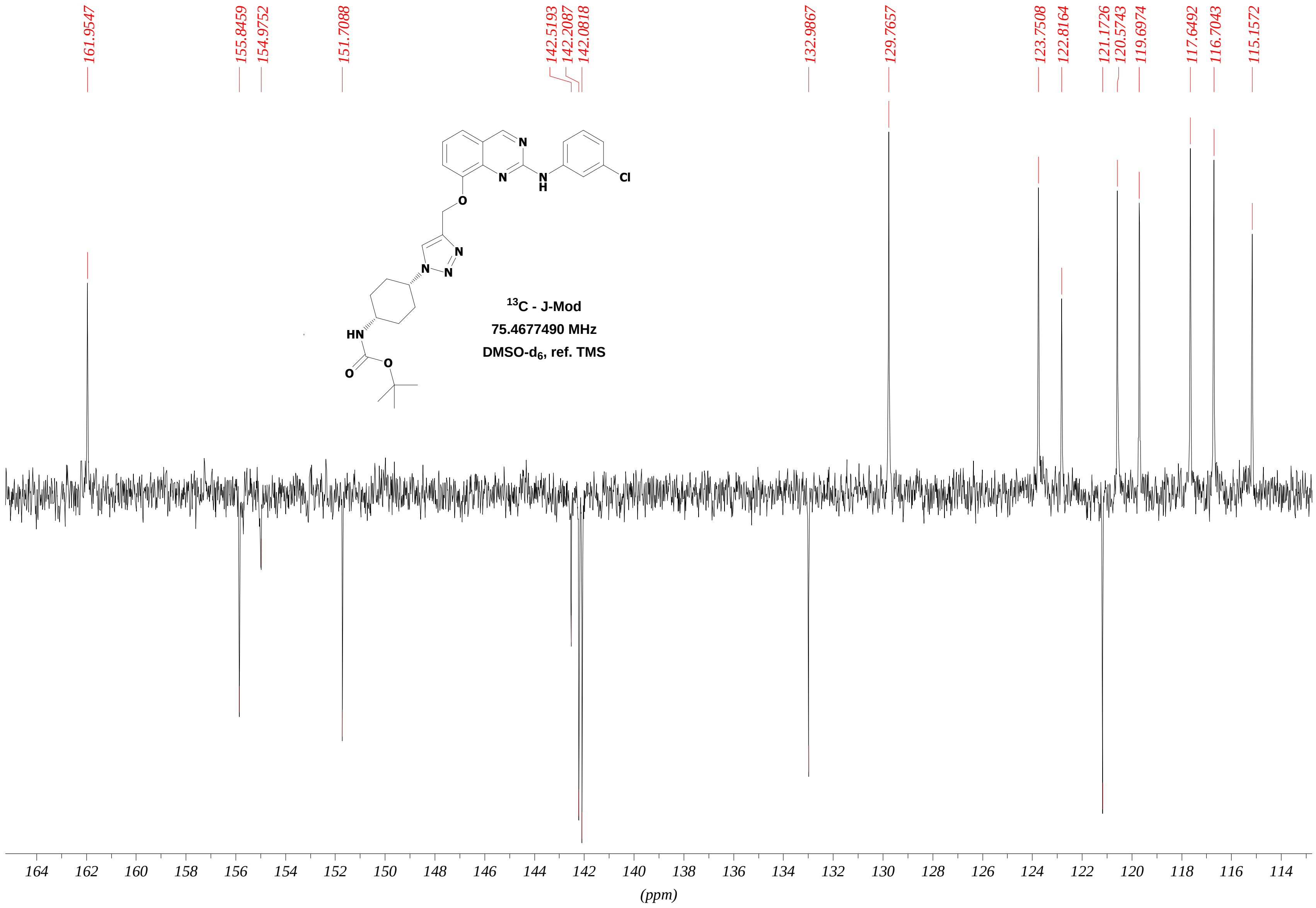
161.9547
155.8459
154.9752
151.7088
142.5193
142.2087
142.0818
132.9867
129.7657
123.7508
122.8164
121.1726
120.5743
119.6974
117.6492
116.7043
115.1572
N
N
N
H
Cl
O
N
N
N
HN
O
O
^{13}C - J-Mod
75.4677490 MHz
DMSO-d_6, ref. TMS
164
162
160
158
156
154
152
150
148
146
144
142
140
138
136
134
132
130
128
126
124
122
120
118
116
114
(ppm)

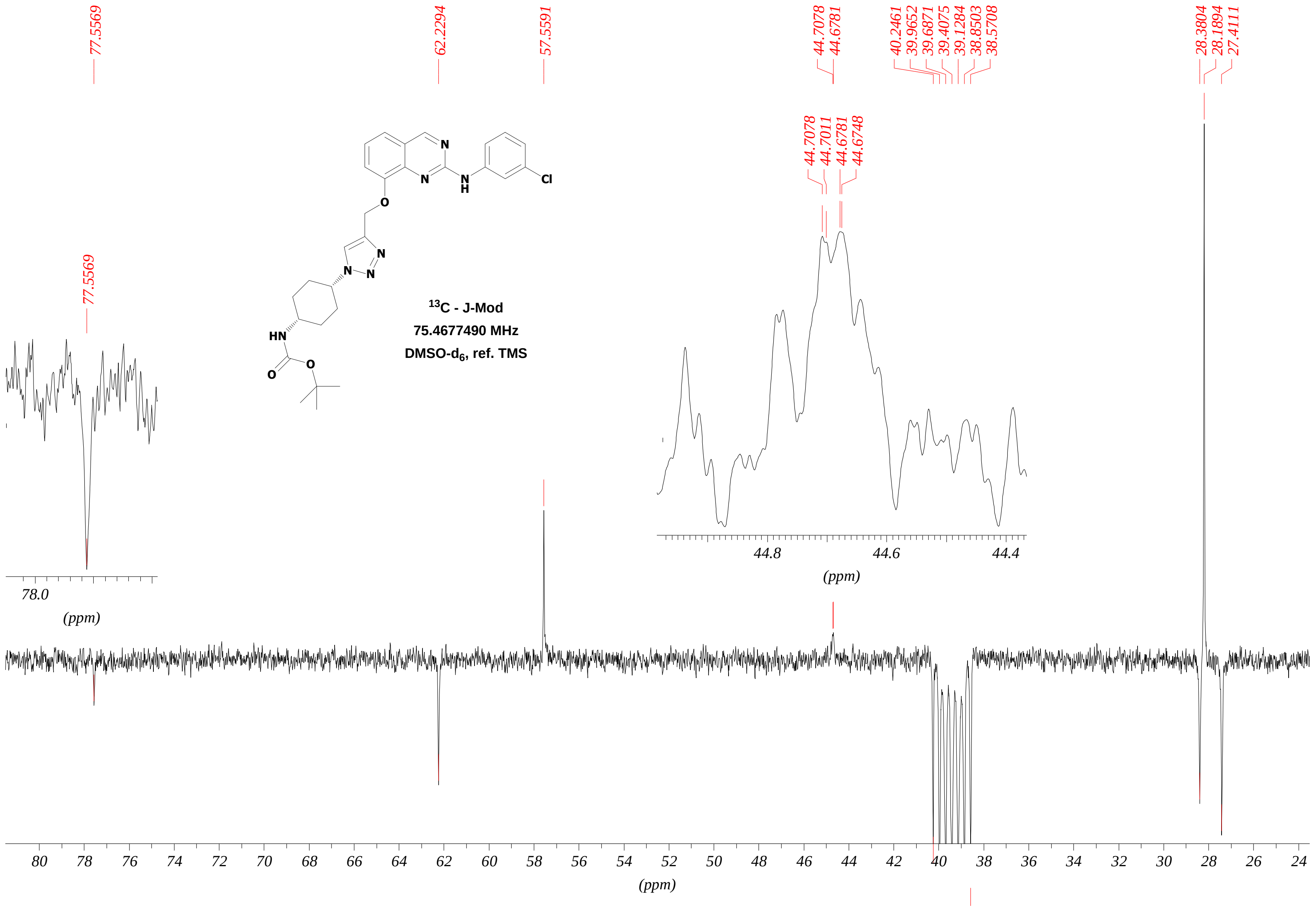

77.5569
62.2294
57.5591
44.7078
44.6781
40.2461
39.9652
39.6871
39.4075
39.1284
38.8503
38.5708
28.3804
28.1894
27.4111
N
N
N
H
Cl
O
N
N
N
HN
O
O
^{13}C - J-Mod
75.4677490 MHz
DMSO-d$_6$, ref. TMS
77.5569
78.0
(ppm)
44.7078
44.7011
44.6781
44.6748
44.8
44.6
44.4
(ppm)
80 78 76 74 72 70 68 66 64 62 60 58 56 54 52 50 48 46 44 42 40 38 36 34 32 30 28 26 24
(ppm)

tert-Butyl ((_cis_)-4-(4-(((2-((3,5-dichlorophenyl)amino)quinazolin-8-yl)oxy)methyl)-1_H_-1,2,3-triazol-1-yl)cyclohexyl)carbamate (15b):

Pages S124-S133

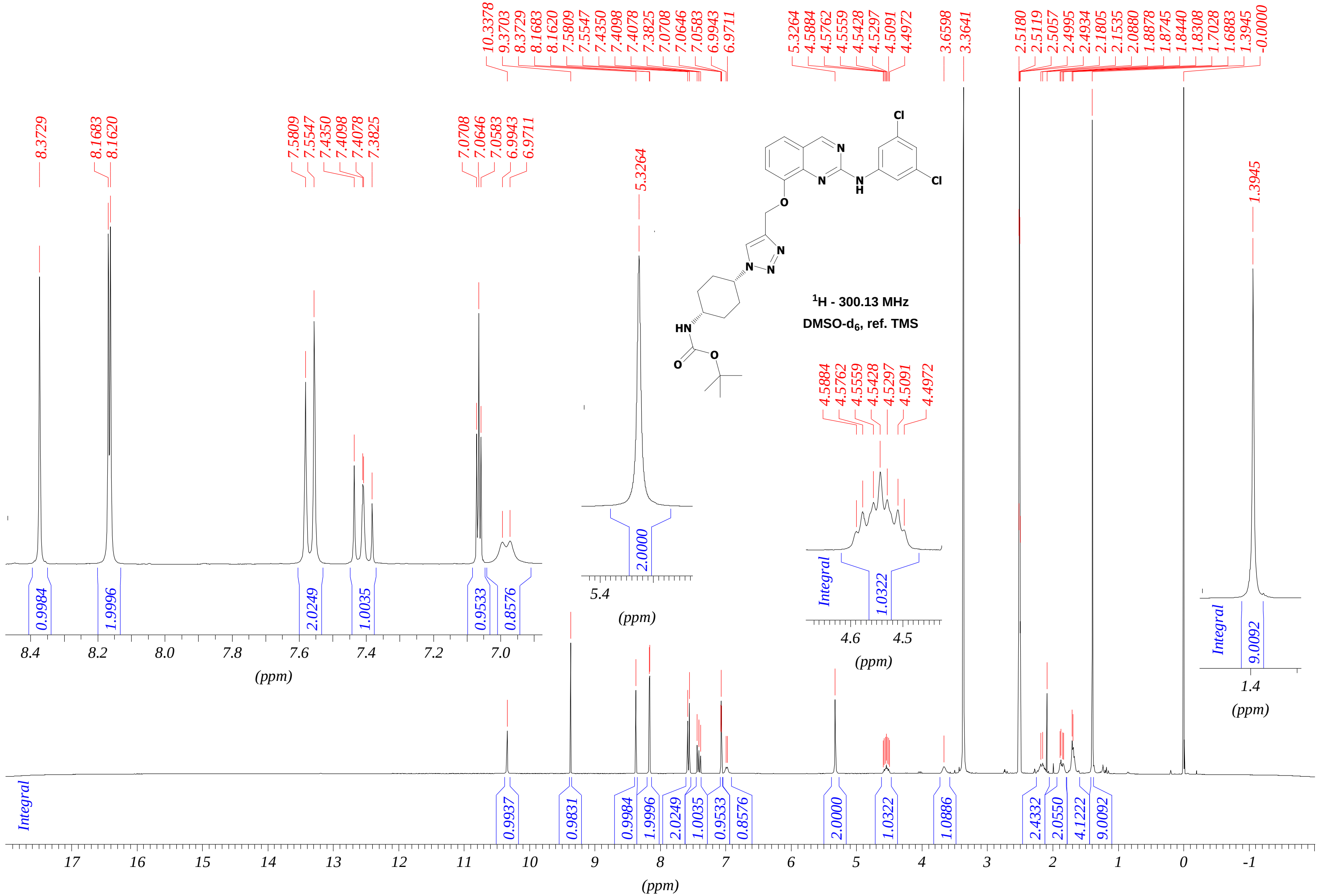

^{1}H - 300.13 MHz
DMSO-d$_6$, ref. TMS
Integral
(ppm)

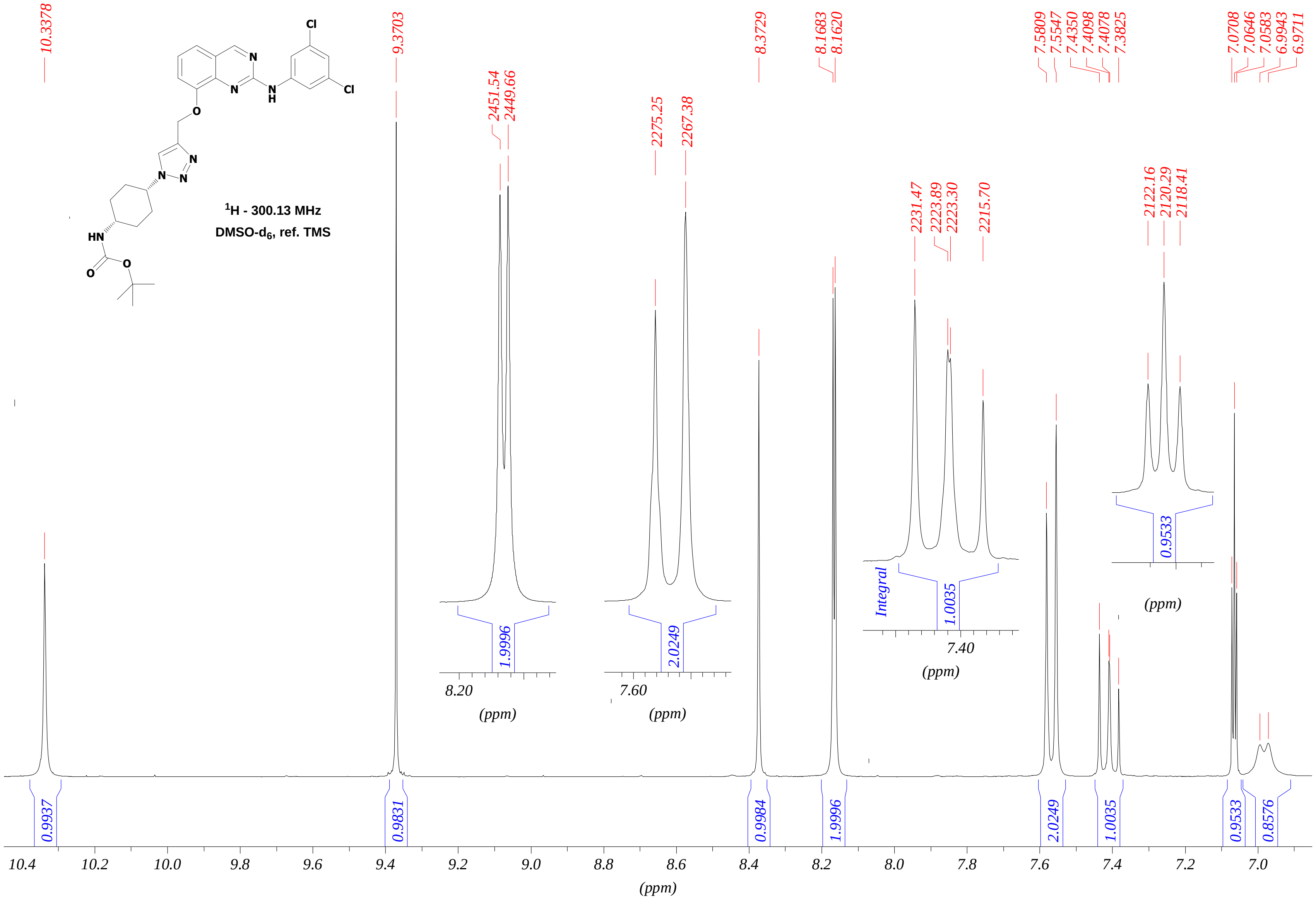

^1H - 300.13 MHz
DMSO-d$_6$, ref. TMS
10.3378
9.3703
8.3729
8.1683
8.1620
7.5809
7.5547
7.4350
7.4098
7.4078
7.3825
7.0708
7.0646
7.0583
6.9943
6.9711
2451.54
2449.66
2275.25
2267.38
2231.47
2223.89
2223.30
2215.70
2122.16
2120.29
2118.41
0.9937
0.9831
0.9984
1.9996
2.0249
1.0035
0.9533
0.8576
Integral
(ppm)

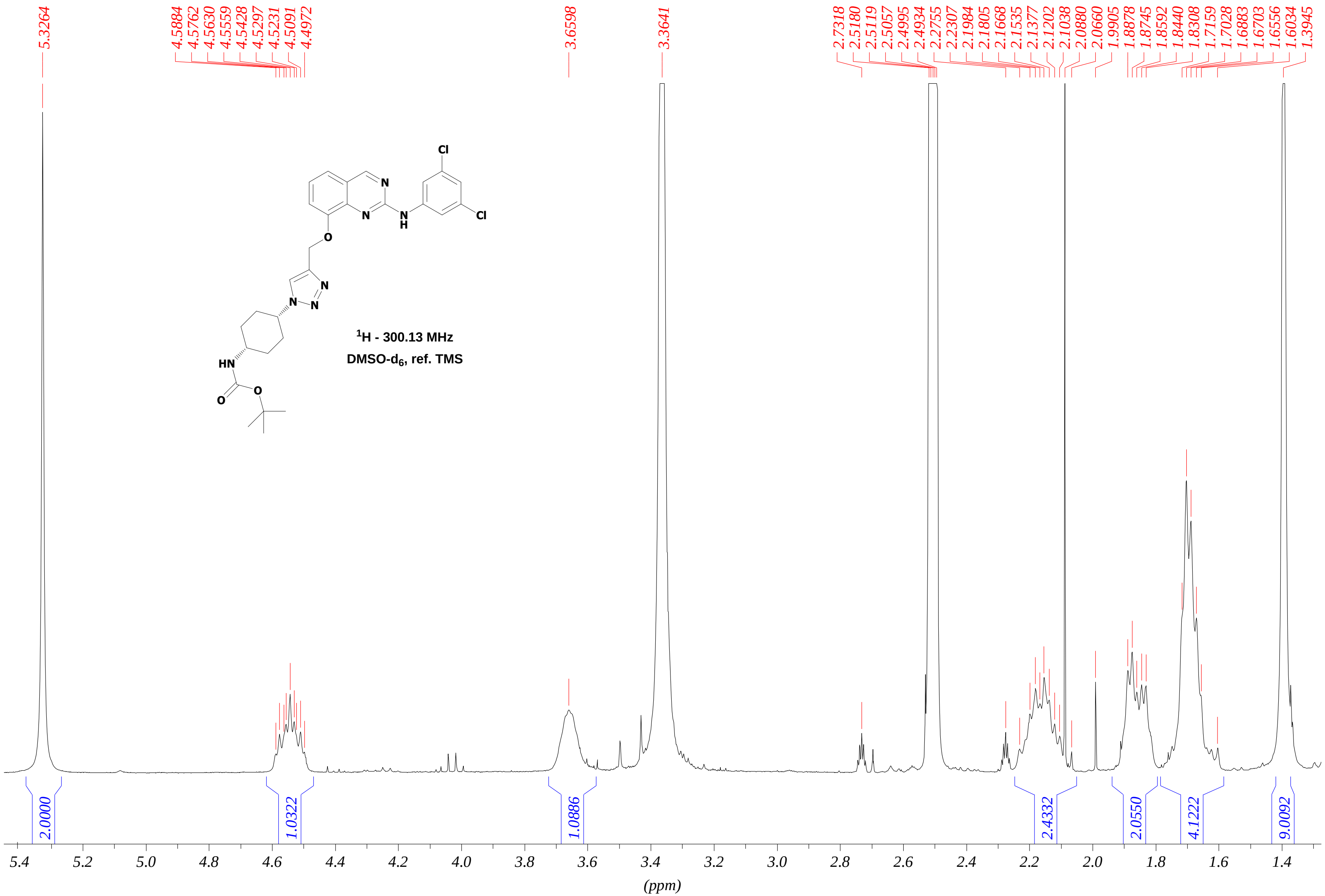
5.3264
4.5884 4.5762 4.5630 4.5559 4.5428 4.5297 4.5231 4.5091 4.4972
3.6598
3.3641
2.7318 2.5180 2.5119 2.5057 2.4995 2.4934 2.2755 2.2307 2.1984 2.1805 2.1668 2.1535 2.1377 2.1202 2.1038 2.0880 2.0660 1.9905 1.8878 1.8745 1.8592 1.8440 1.8308 1.7159 1.7028 1.6883 1.6703 1.6556 1.6034 1.3945
^{1}H - 300.13 MHz
DMSO-d$_6$, ref. TMS
2.0000
1.0322
1.0886
2.4332
2.0550
4.1222
9.0092
5.4 5.2 5.0 4.8 4.6 4.4 4.2 4.0 3.8 3.6 3.4 3.2 3.0 2.8 2.6 2.4 2.2 2.0 1.8 1.6 1.4
(ppm)

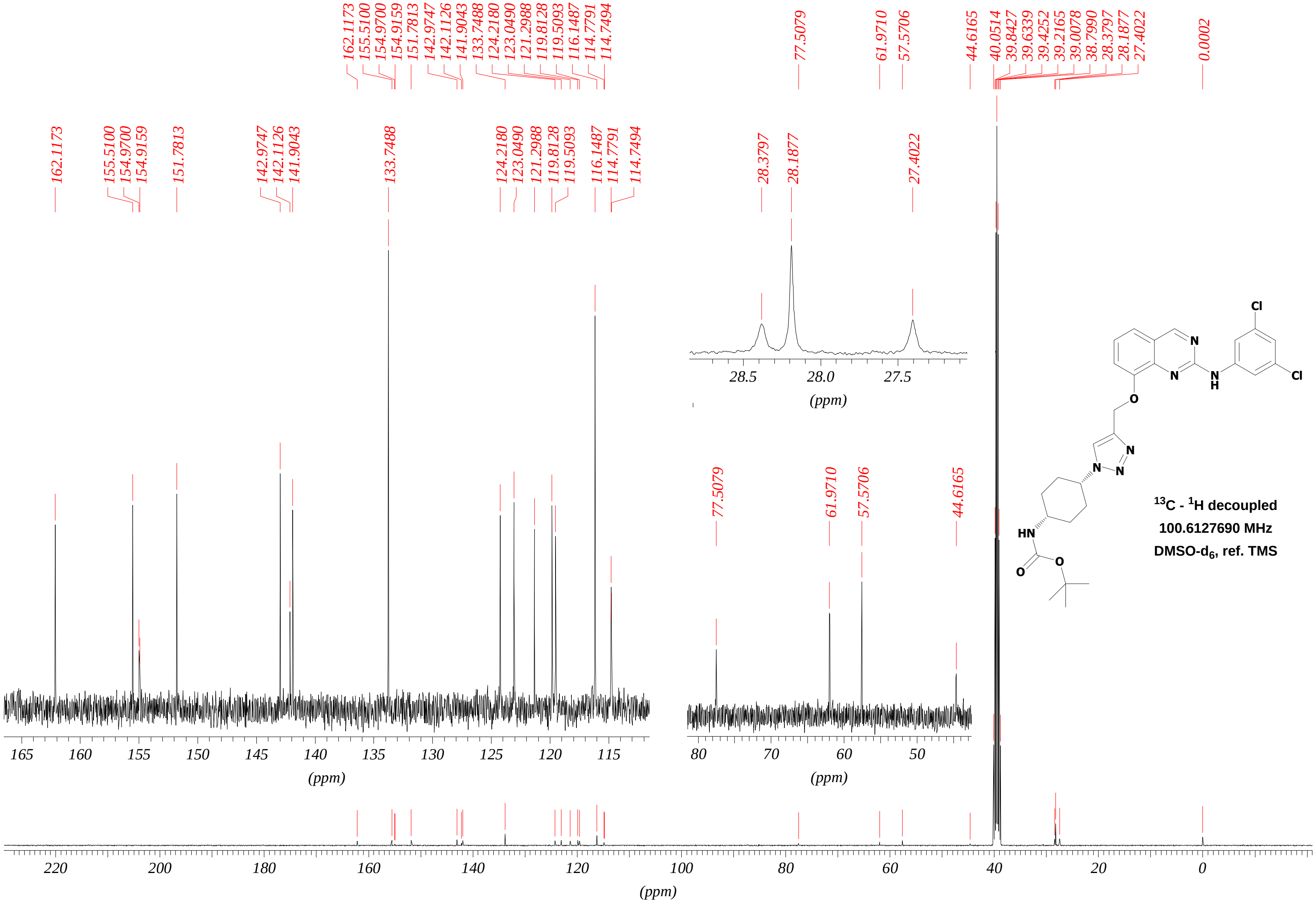
162.1173
155.5100
154.9700
154.9159
151.7813
142.9747
142.1126
141.9043
133.7488
124.2180
123.0490
121.2988
119.8128
119.5093
116.1487
114.7791
114.7494
77.5079
61.9710
57.5706
44.6165
40.0514
39.8427
39.6339
39.4252
39.2165
39.0078
38.7990
28.3797
28.1877
27.4022
0.0002
Cl
N
N
N
H
Cl
O
N
N
N
HN
O
O
^{13}C - ^{1}H decoupled
100.6127690 MHz
DMSO-d$_6$, ref. TMS
(ppm)

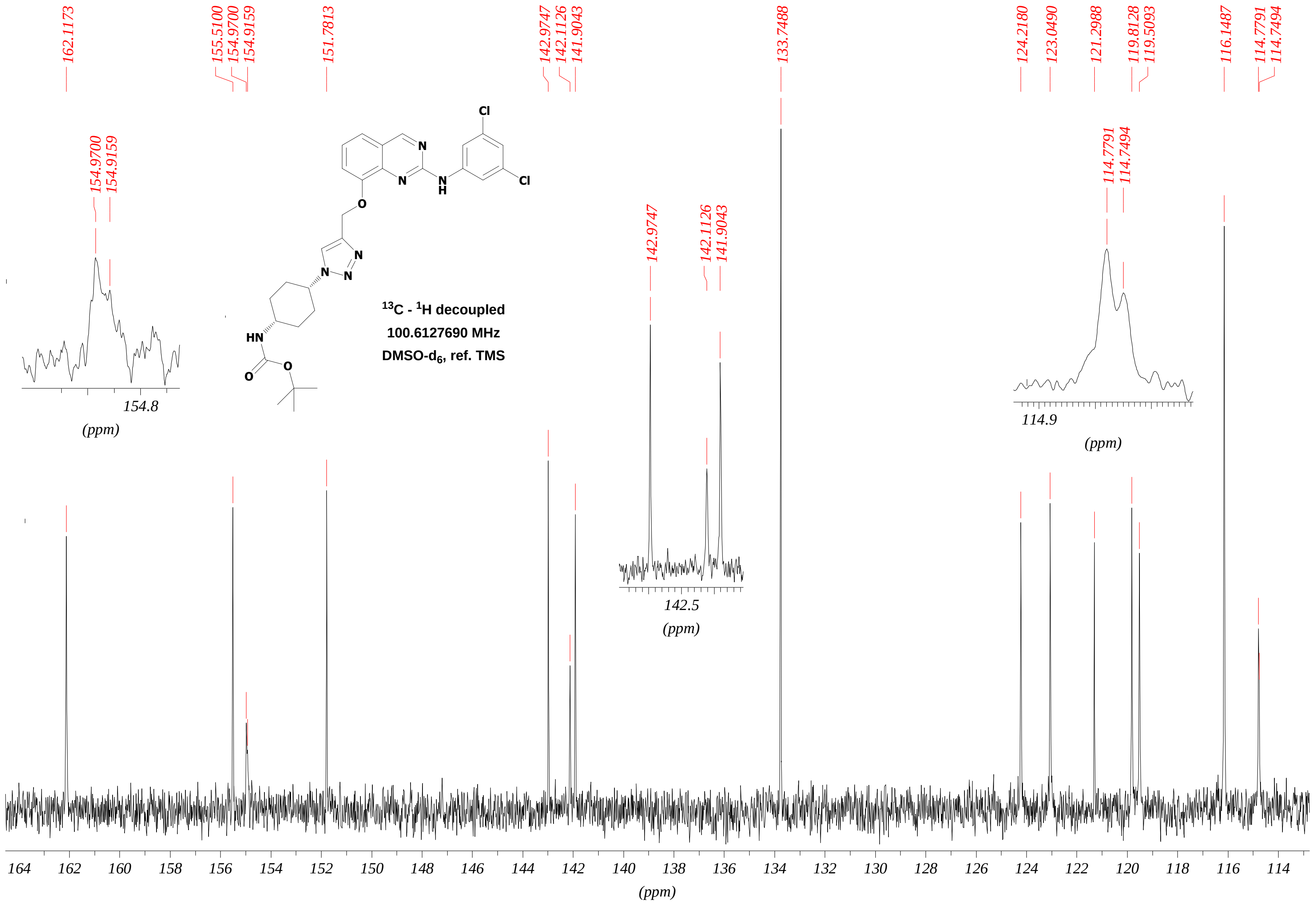

162.1173
155.5100
154.9700
154.9159
151.7813
142.9747
142.1126
141.9043
133.7488
124.2180
123.0490
121.2988
119.8128
119.5093
116.1487
114.7791
114.7494
Cl
N
N
N
H
Cl
O
N
N
N
HN
O
O
^{13}C - ^{1}H decoupled
100.6127690 MHz
DMSO-d$_6$, ref. TMS
154.8
(ppm)
142.5
(ppm)
114.9
(ppm)
164
162
160
158
156
154
152
150
148
146
144
142
140
138
136
134
132
130
128
126
124
122
120
118
116
114
(ppm)

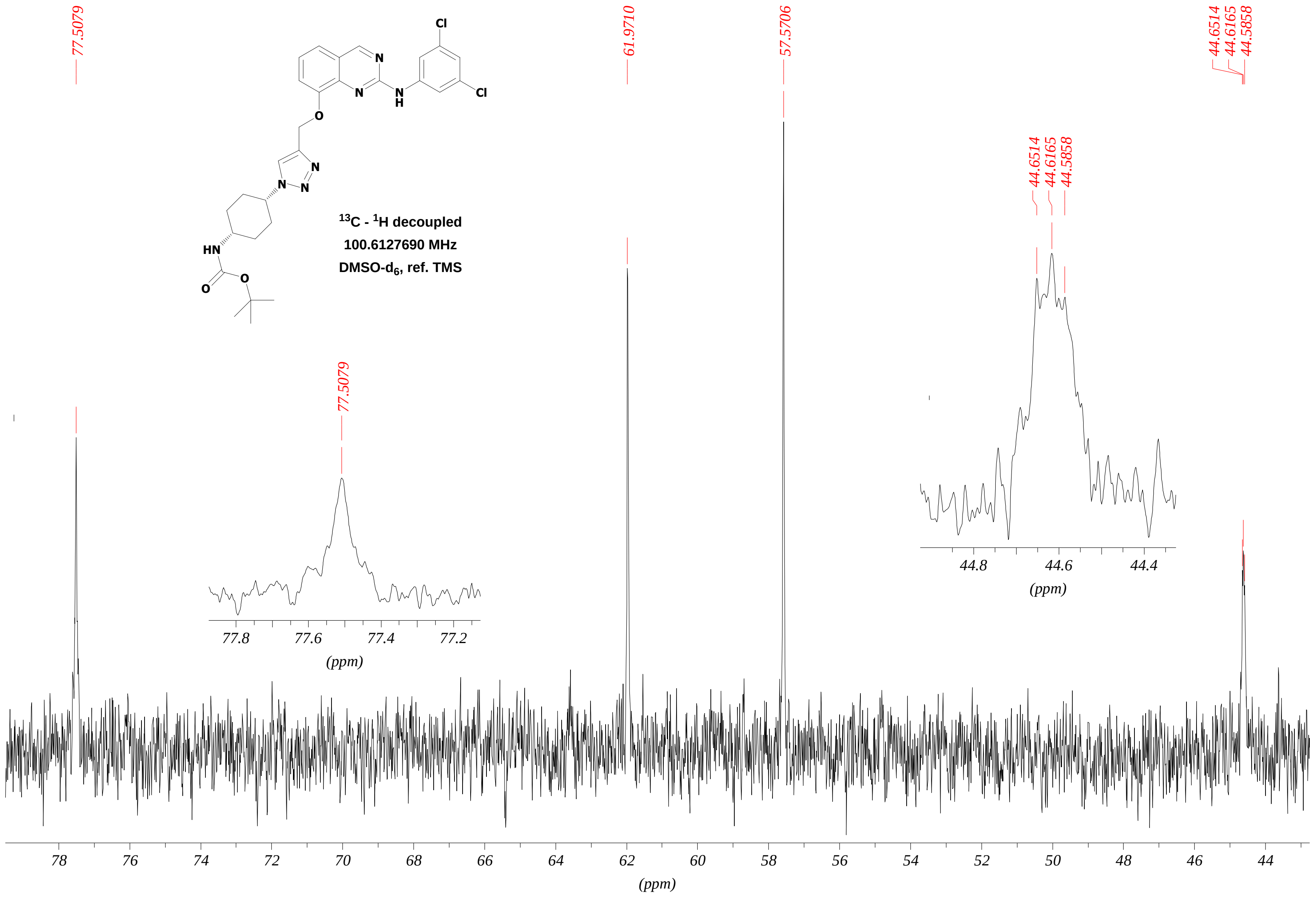
77.5079
61.9710
57.5706
44.6514
44.6165
44.5858
Cl
N
N
N
H
Cl
O
N
N
N
HN
O
O
^{13}C - ^{1}H decoupled
100.6127690 MHz
DMSO-d_6, ref. TMS
77.8
77.6
77.4
77.2
(ppm)
44.8
44.6
44.4
(ppm)
78
76
74
72
70
68
66
64
62
60
58
56
54
52
50
48
46
44
(ppm)

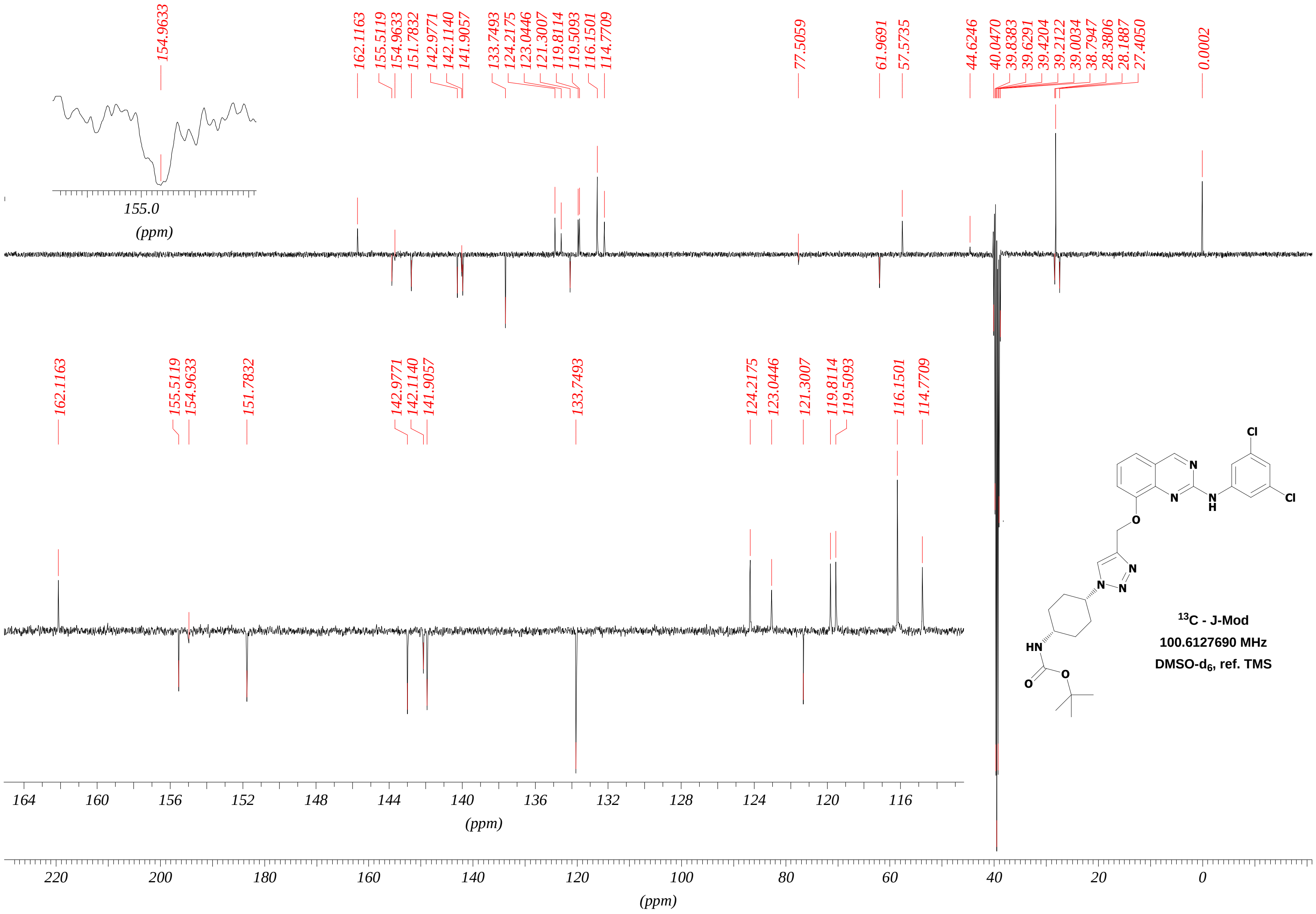
154.9633
155.0
(ppm)
162.1163
155.5119
154.9633
151.7832
142.9771
142.1140
141.9057
133.7493
124.2175
123.0446
121.3007
119.8114
119.5093
116.1501
114.7709
77.5059
61.9691
57.5735
44.6246
40.0470
39.8383
39.6291
39.4204
39.2122
39.0034
38.7947
28.3806
28.1887
27.4050
0.0002
164
160
156
152
148
144
140
136
132
128
124
120
116
(ppm)
220
200
180
160
140
120
100
80
60
40
20
0
(ppm)
Cl
N
N
N
H
Cl
O
N
N
N
HN
O
O
^{13}C - J-Mod
100.6127690 MHz
DMSO-d$_6$, ref. TMS

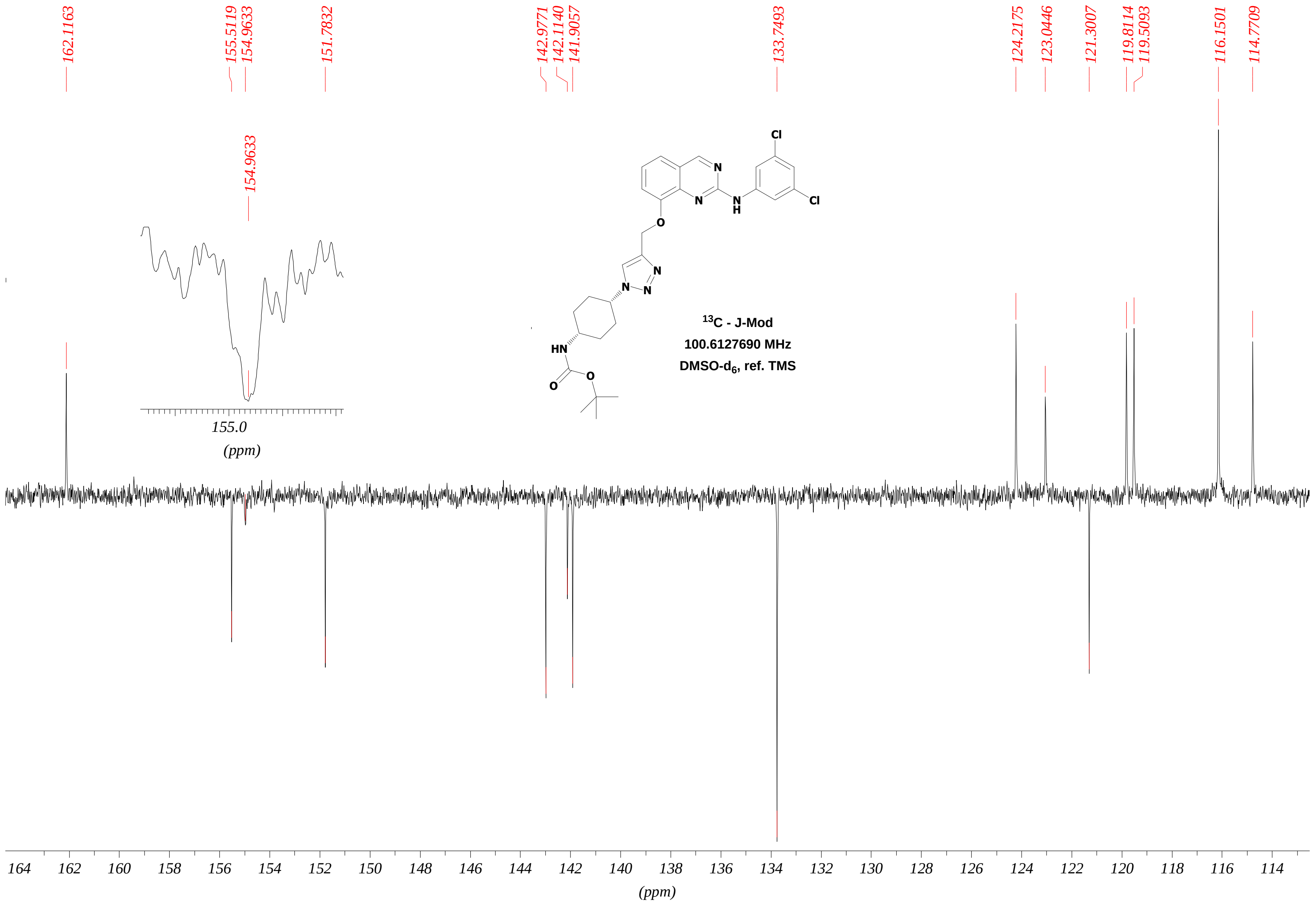
162.1163
155.5119
154.9633
151.7832
142.9771
142.1140
141.9057
133.7493
124.2175
123.0446
121.3007
119.8114
119.5093
116.1501
114.7709
154.9633
155.0
(ppm)
Cl
N
N
N
H
Cl
O
N
N
N
HN
O
O
^{13}C - J-Mod
100.6127690 MHz
DMSO-d_6, ref. TMS
164 162 160 158 156 154 152 150 148 146 144 142 140 138 136 134 132 130 128 126 124 122 120 118 116 114
(ppm)

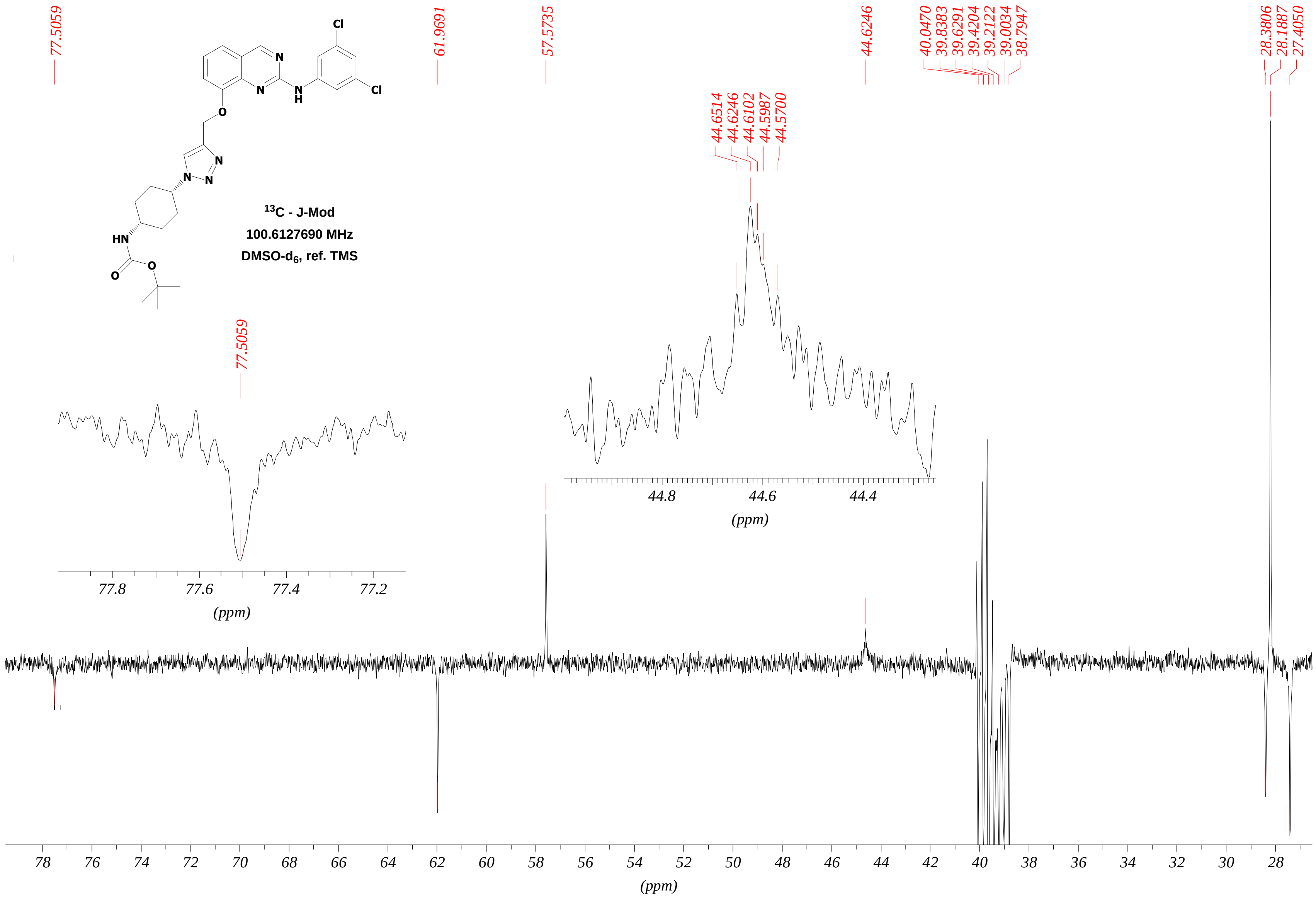
13C - J-Mod
100.6127690 MHz
DMSO-d6, ref. TMS
77.5059
61.9691
57.5735
44.6246
40.0470
39.8383
39.6291
39.4204
39.2122
39.0034
38.7947
28.3806
28.1887
27.4050
44.6514
44.6246
44.6102
44.5987
44.5700
(ppm)

***tert*-Butyl ((*trans*)-4-(4-(((2-((3-chlorophenyl)amino) quinazolin-8-yl) oxy) methyl)-1*H*-1,2,3-triazol-1-yl)cyclohexyl)carbamate (18a):**

Pages S134-S147

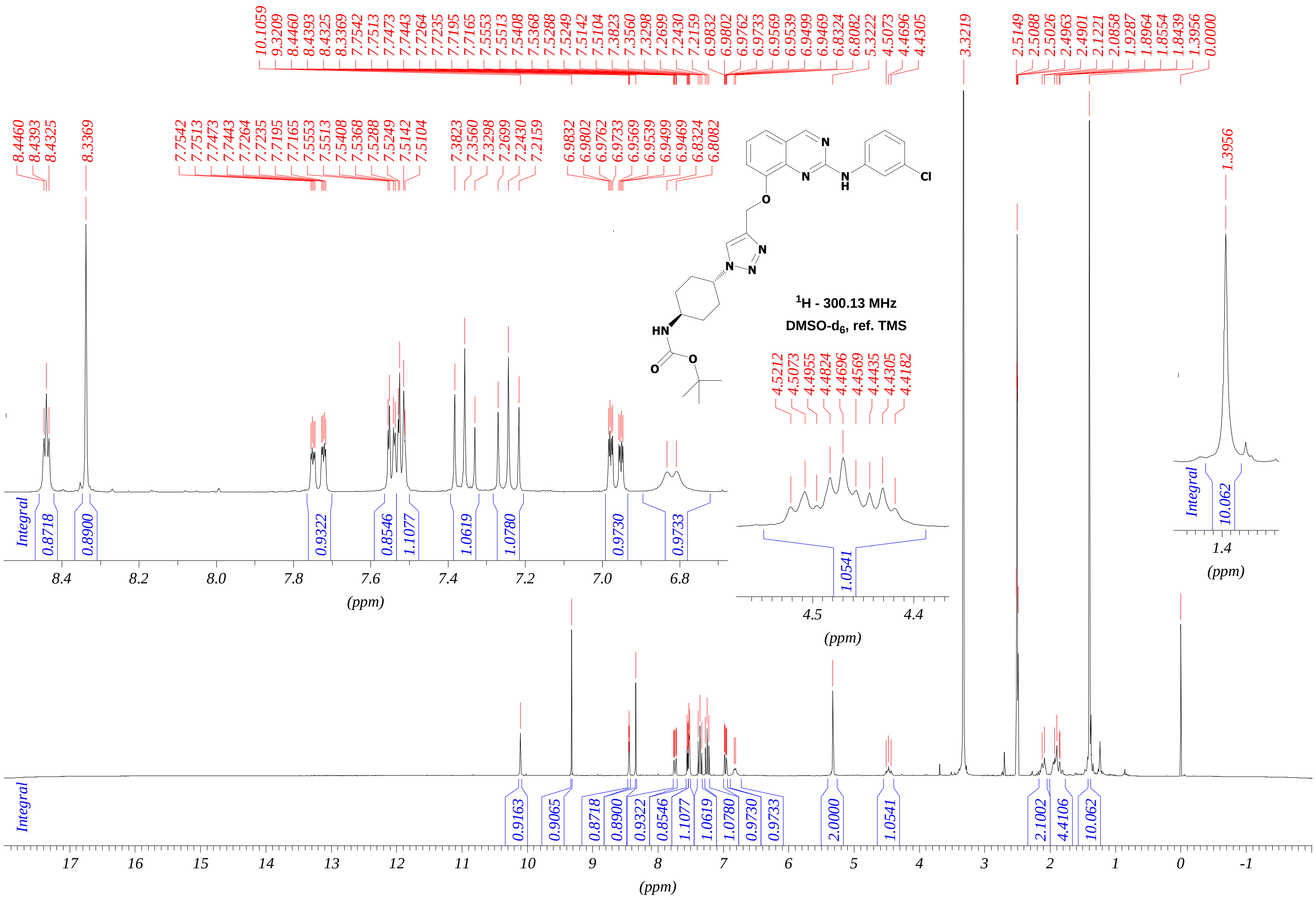

^{1}H - 300.13 MHz
DMSO-d_6, ref. TMS
(ppm)
Integral

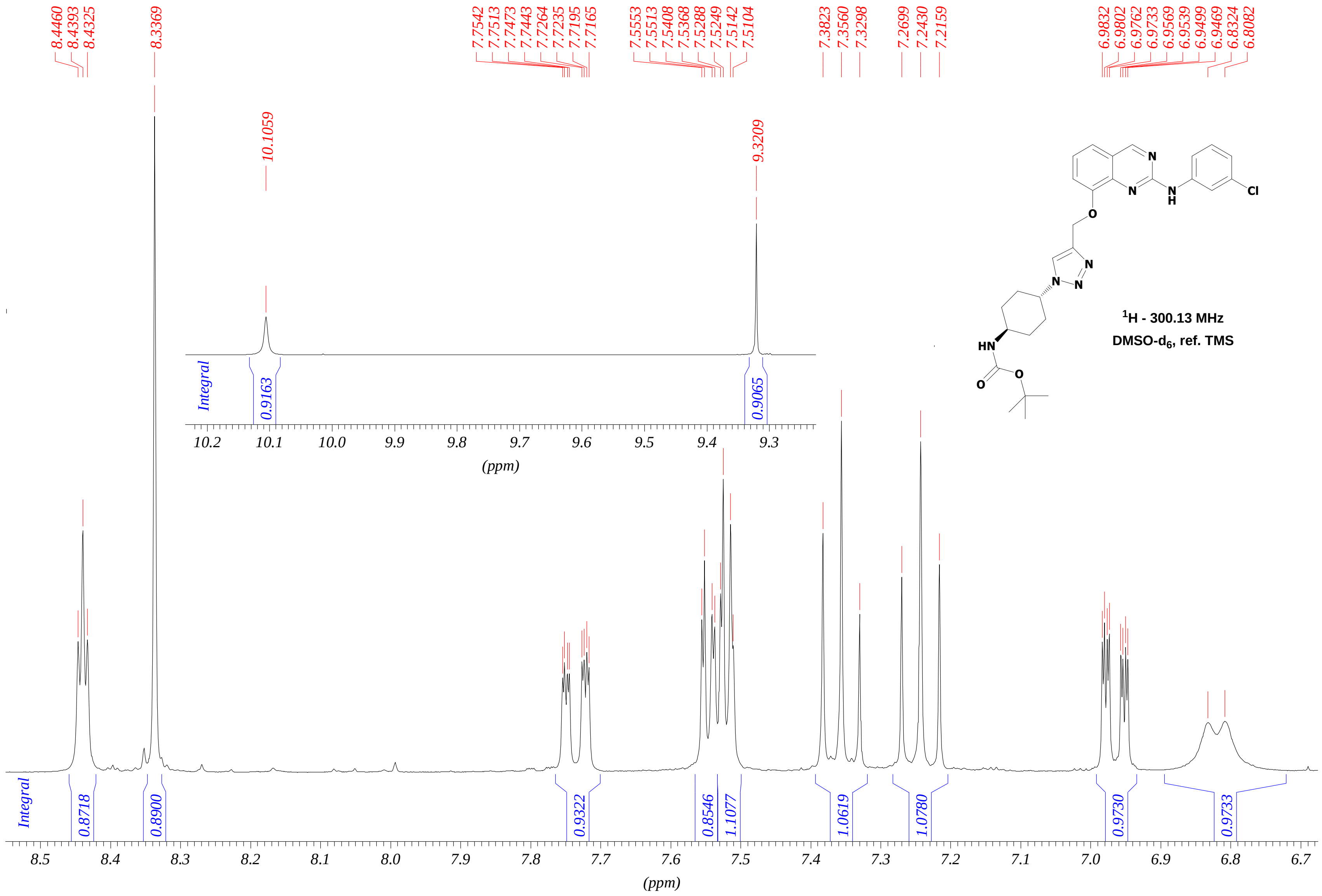

¹H - 300.13 MHz
DMSO-d6, ref. TMS
Integral
(ppm)
8.4460 8.4393 8.4325 8.3369 7.7542 7.7513 7.7473 7.7443 7.7264 7.7235 7.7195 7.7165 7.5553 7.5513 7.5408 7.5368 7.5288 7.5249 7.5142 7.5104 7.3823 7.3560 7.3298 7.2699 7.2430 7.2159 6.9832 6.9802 6.9762 6.9733 6.9569 6.9539 6.9499 6.9469 6.8324 6.8082
10.1059 9.3209
0.9163 0.9065
0.8718 0.8900 0.9322 0.8546 1.1077 1.0619 1.0780 0.9730 0.9733

Addition of D_2O to the NMR sample displaced the peak of water from 3.32 to 3.58 ppm by converting H_2O into DOH. That revealed a proton at 3.33 ppm (m from 3.26 to 3.42 ppm) which was initially superimposed with the peak of water. The NH signal at 6.82 ppm almost disappeared by exchange.

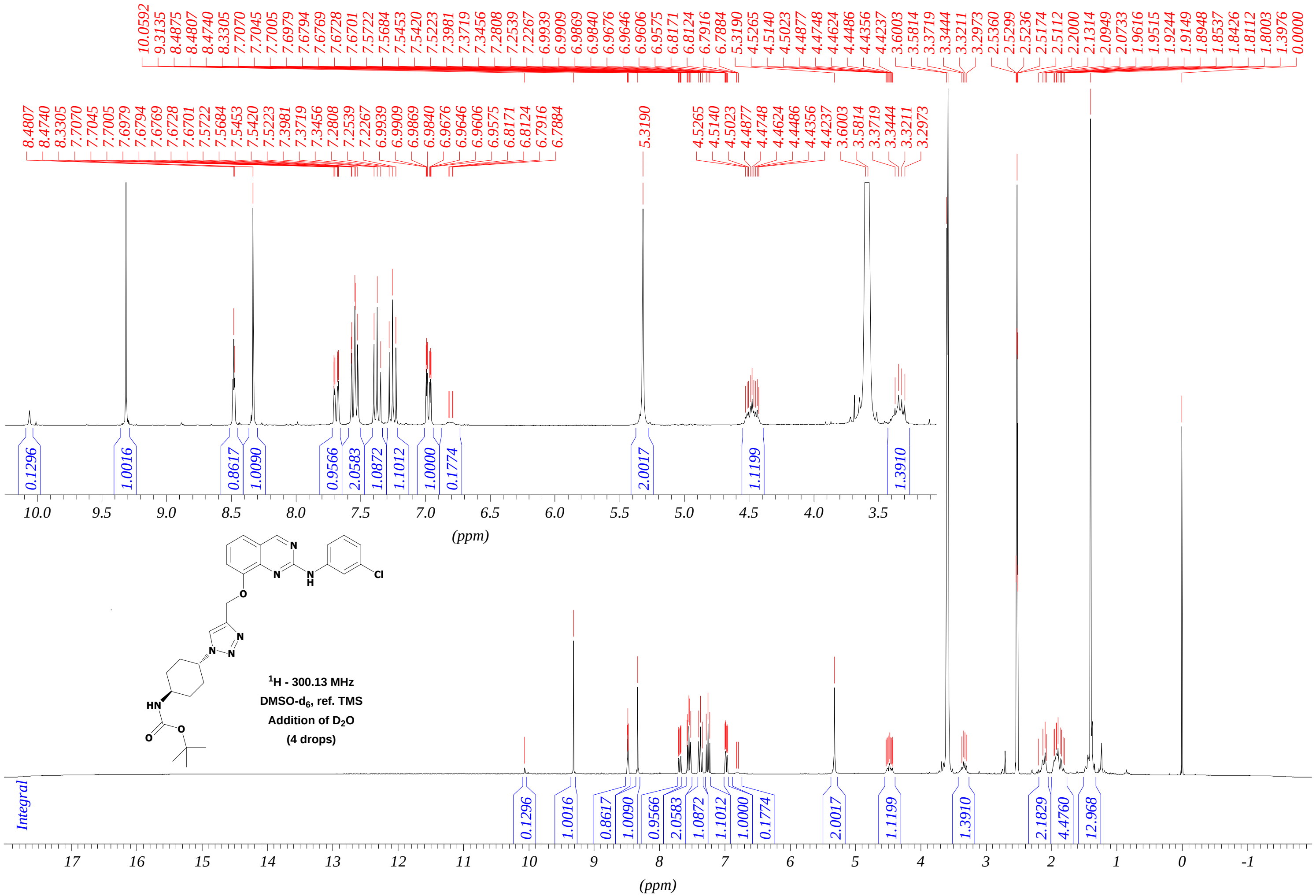

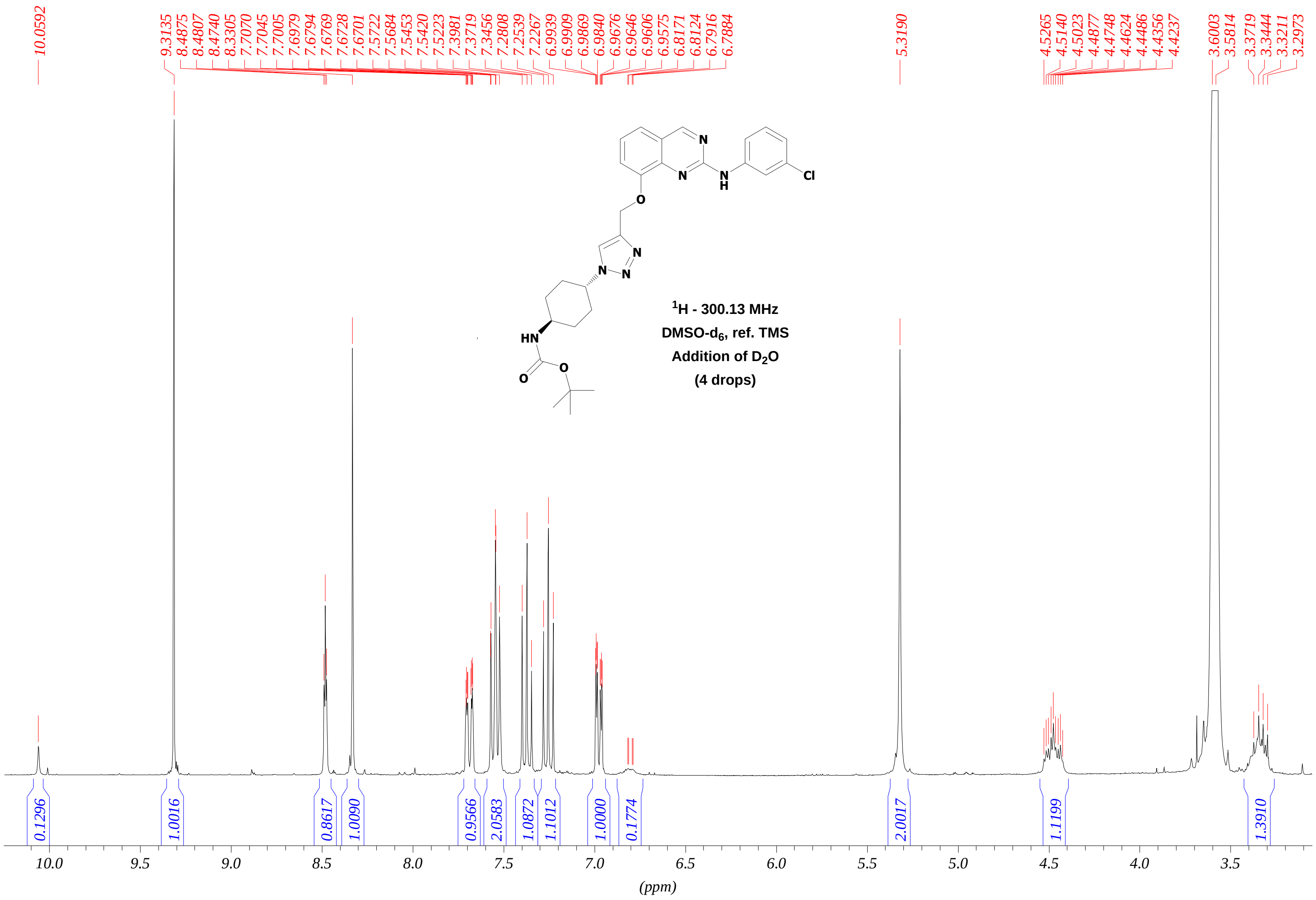

10.0592
9.3135
8.4875
8.4807
8.4740
8.3305
7.7070
7.7045
7.7005
7.6979
7.6794
7.6769
7.6728
7.6701
7.5722
7.5684
7.5453
7.5420
7.5223
7.3981
7.3719
7.3456
7.2808
7.2539
7.2267
6.9939
6.9909
6.9869
6.9840
6.9676
6.9646
6.9606
6.9575
6.8171
6.8124
6.7916
6.7884
5.3190
4.5265
4.5140
4.5023
4.4877
4.4748
4.4624
4.4486
4.4356
4.4237
3.6003
3.5814
3.3719
3.3444
3.3211
3.2973
N
N
N
H
Cl
O
N
N
N
HN
O
O
^{1}H - 300.13 MHz
DMSO-d$_6$, ref. TMS
Addition of D$_2$O
(4 drops)
0.1296
1.0016
0.8617
1.0090
0.9566
2.0583
1.0872
1.1012
1.0000
0.1774
2.0017
1.1199
1.3910
10.0
9.5
9.0
8.5
8.0
7.5
7.0
6.5
6.0
5.5
5.0
4.5
4.0
3.5
(ppm)

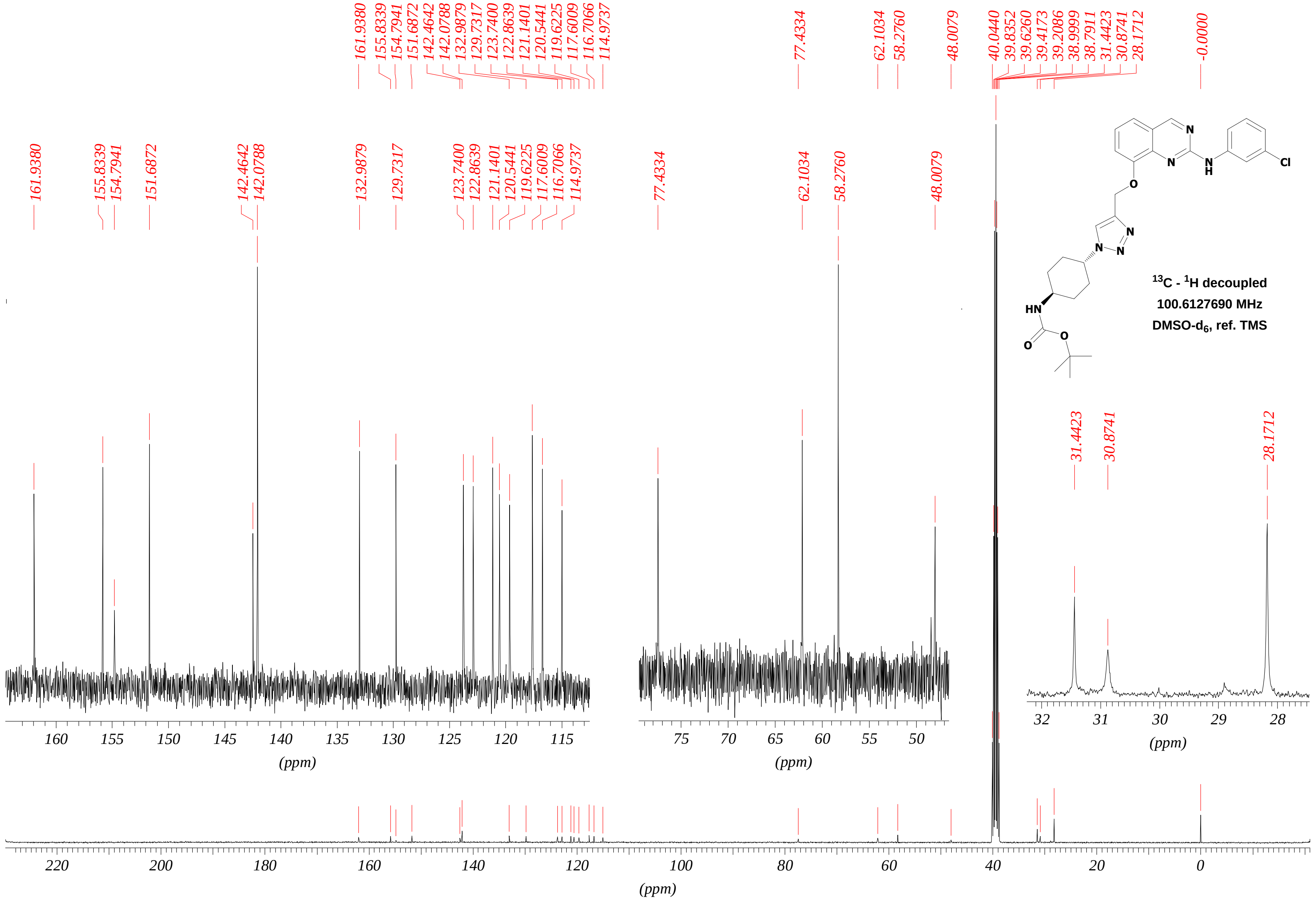

161.9380
155.8339
154.7941
151.6872
142.4642
142.0788
132.9879
129.7317
123.7400
122.8639
121.1401
120.5441
119.6225
117.6009
116.7066
114.9737
77.4334
62.1034
58.2760
48.0079
40.0440
39.8352
39.6260
39.4173
39.2086
38.9999
38.7911
31.4423
30.8741
28.1712
-0.0000
^{13}C - ^{1}H decoupled
100.6127690 MHz
DMSO-d_6, ref. TMS
(ppm)

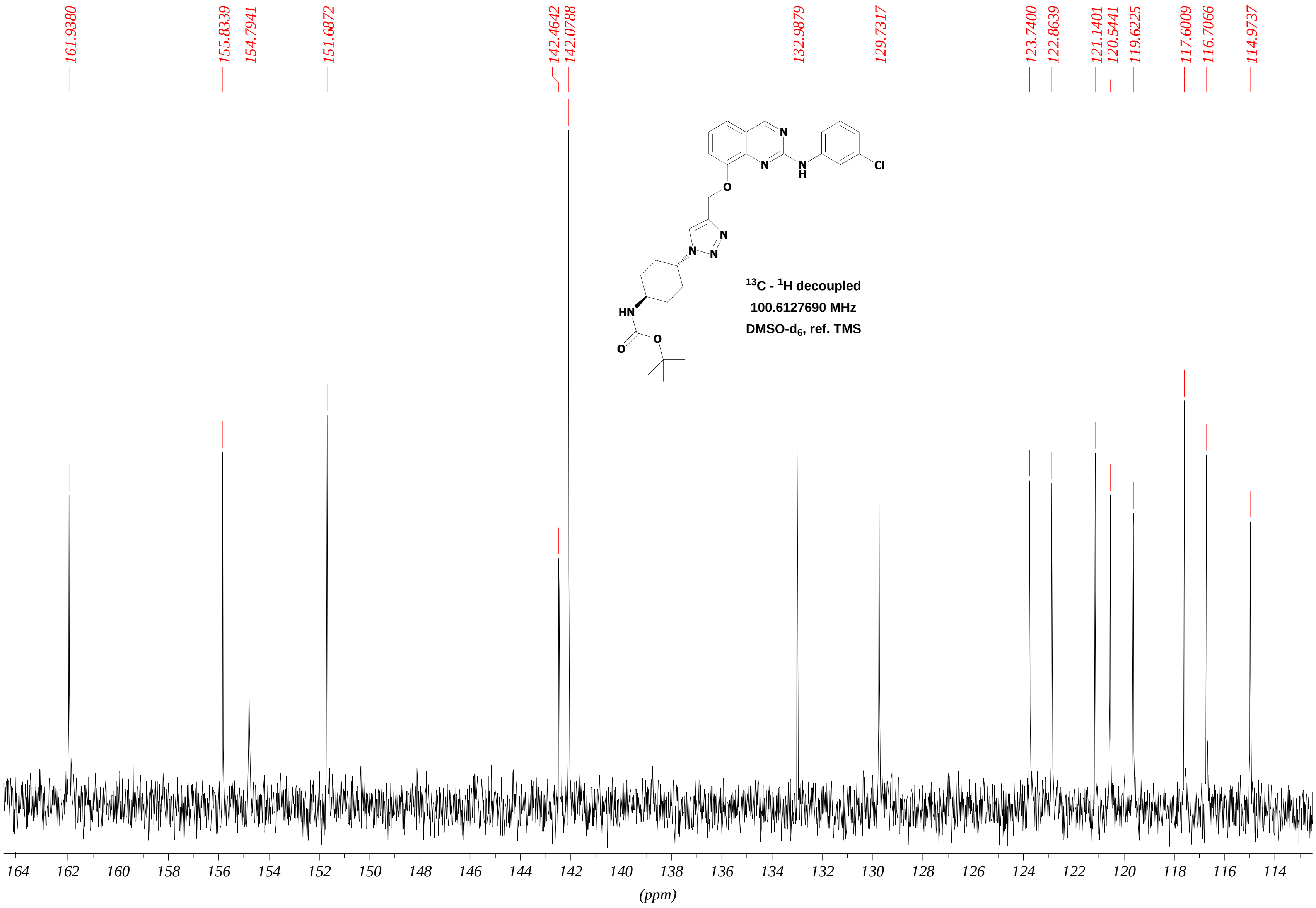
161.9380
155.8339
154.7941
151.6872
142.4642
142.0788
132.9879
129.7317
123.7400
122.8639
121.1401
120.5441
119.6225
117.6009
116.7066
114.9737
N
N
N
H
Cl
O
N
N
N
HN
O
O
^{13}C - ^{1}H decoupled
100.6127690 MHz
DMSO-d$_6$, ref. TMS
164
162
160
158
156
154
152
150
148
146
144
142
140
138
136
134
132
130
128
126
124
122
120
118
116
114
(ppm)

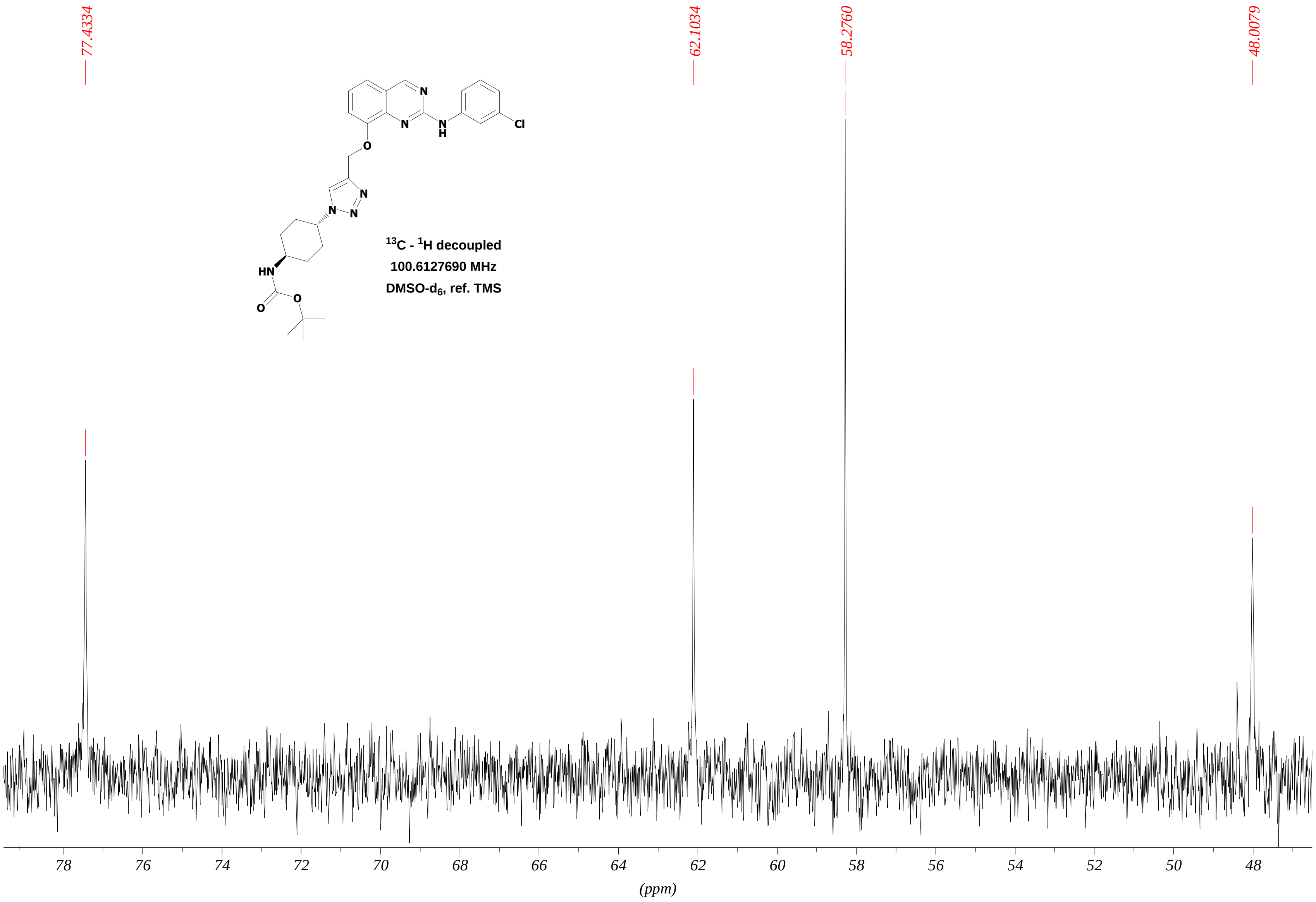
77.4334
62.1034
58.2760
48.0079
N
N
N
H
Cl
O
N
N
N
HN
O
O
^{13}C - ^{1}H decoupled
100.6127690 MHz
DMSO-d$_6$, ref. TMS
78
76
74
72
70
68
66
64
62
60
58
56
54
52
50
48
(ppm)

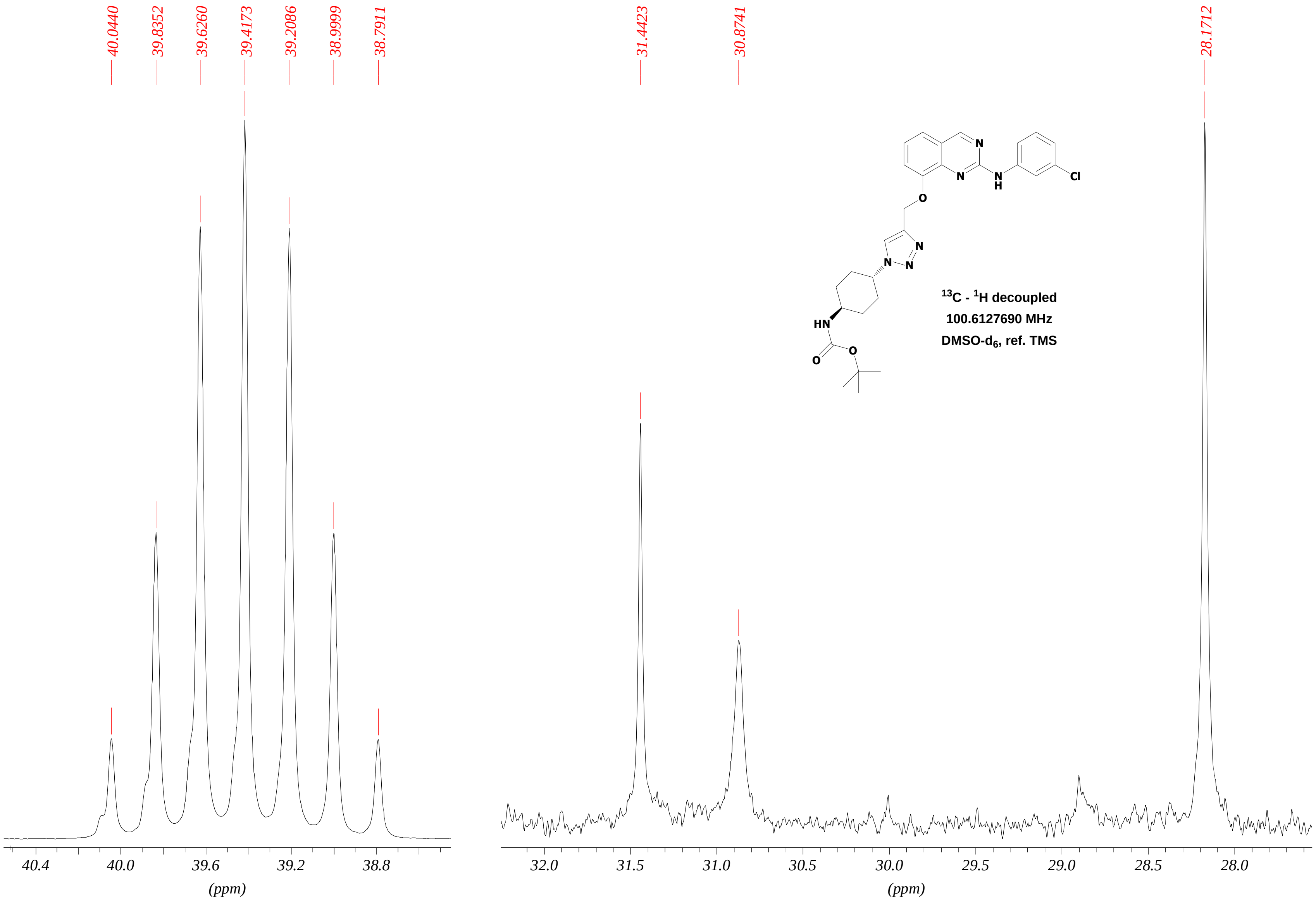
40.0440
39.8352
39.6260
39.4173
39.2086
38.9999
38.7911
31.4423
30.8741
28.1712
^{13}C - ^{1}H decoupled
100.6127690 MHz
DMSO-d$_6$, ref. TMS
40.4
40.0
39.6
39.2
38.8
(ppm)
32.0
31.5
31.0
30.5
30.0
29.5
29.0
28.5
28.0
(ppm)

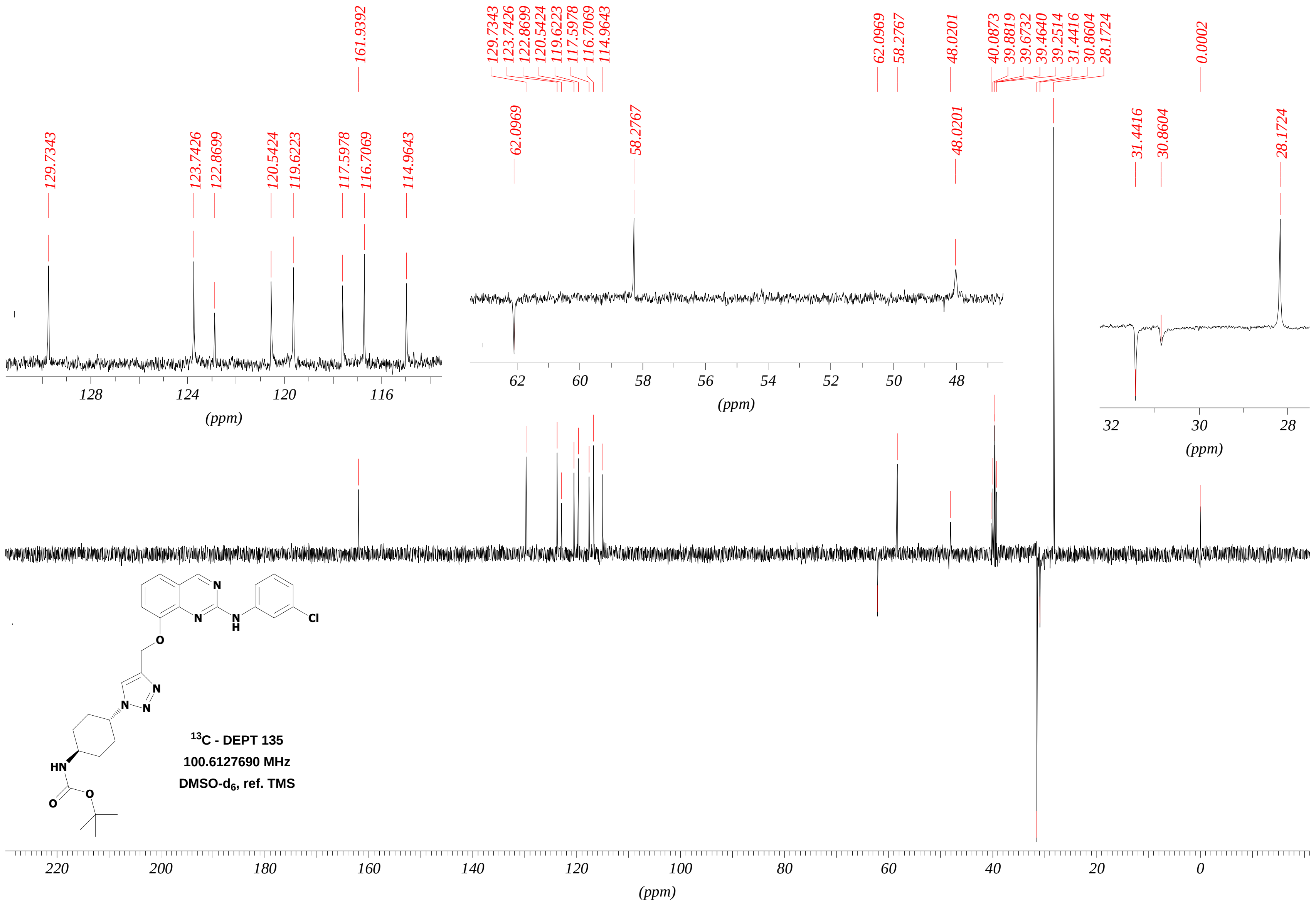

161.9392
129.7343
123.7426
122.8699
120.5424
119.6223
117.5978
116.7069
114.9643
62.0969
58.2767
48.0201
40.0873
39.8819
39.6732
39.4640
39.2514
31.4416
30.8604
28.1724
0.0002
(ppm)
^{13}C - DEPT 135
100.6127690 MHz
DMSO-d_6, ref. TMS

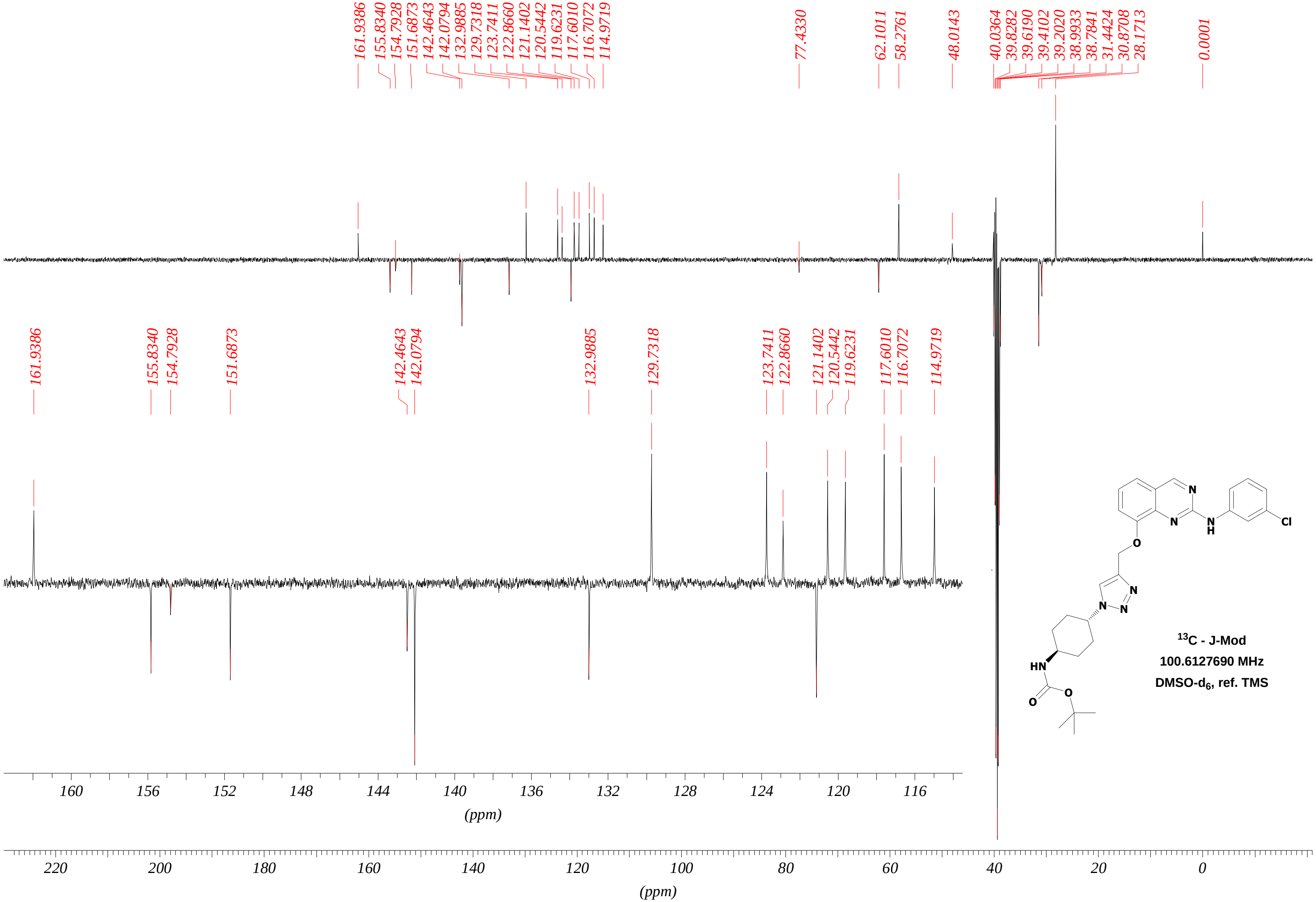

^{13}C - J-Mod
100.6127690 MHz
DMSO-d_6, ref. TMS

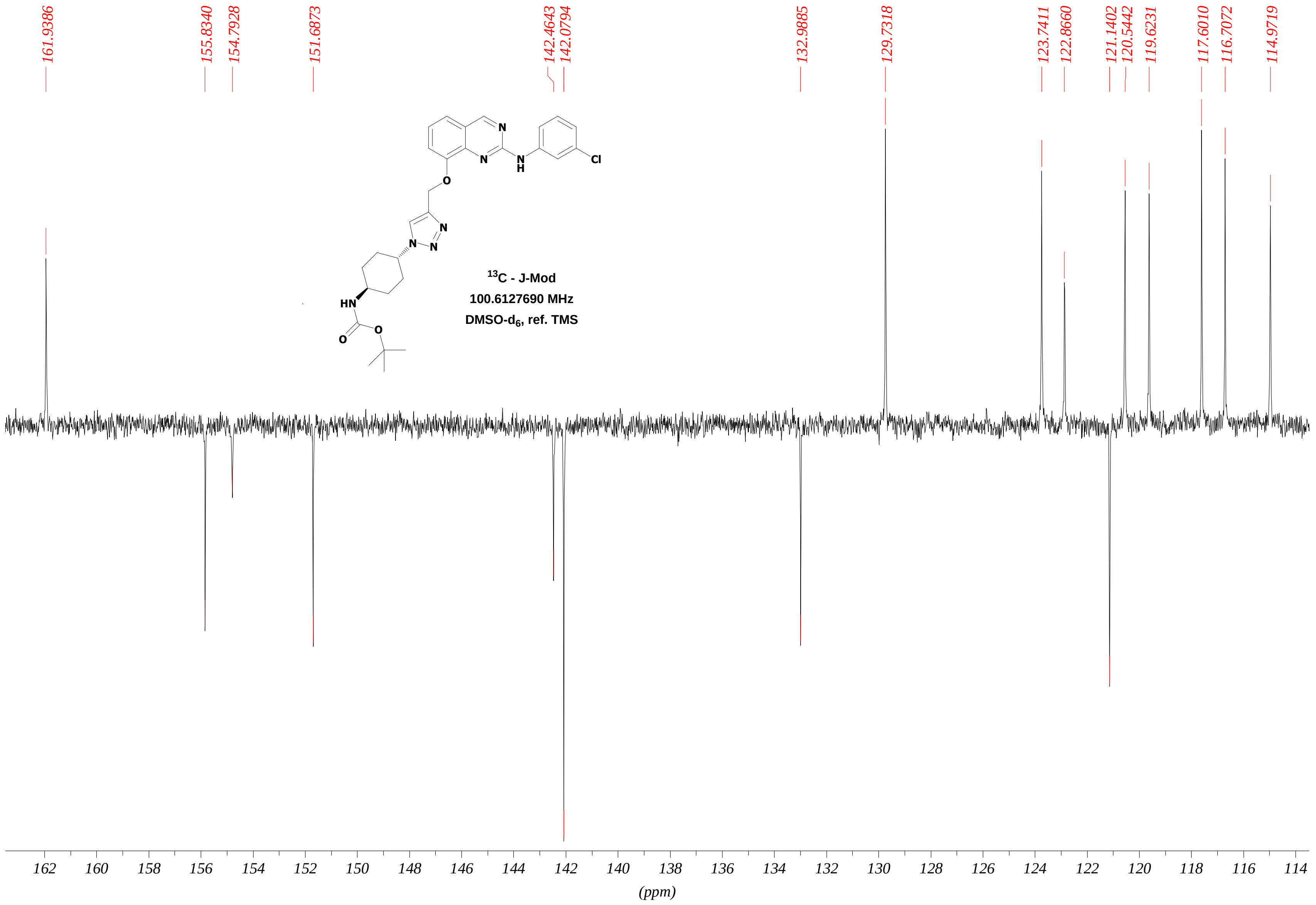

161.9386
155.8340
154.7928
151.6873
142.4643
142.0794
132.9885
129.7318
123.7411
122.8660
121.1402
120.5442
119.6231
117.6010
116.7072
114.9719
N
N
N
H
Cl
O
N
N
N
HN
O
O
13C - J-Mod
100.6127690 MHz
DMSO-d6, ref. TMS
162
160
158
156
154
152
150
148
146
144
142
140
138
136
134
132
130
128
126
124
122
120
118
116
114
(ppm)

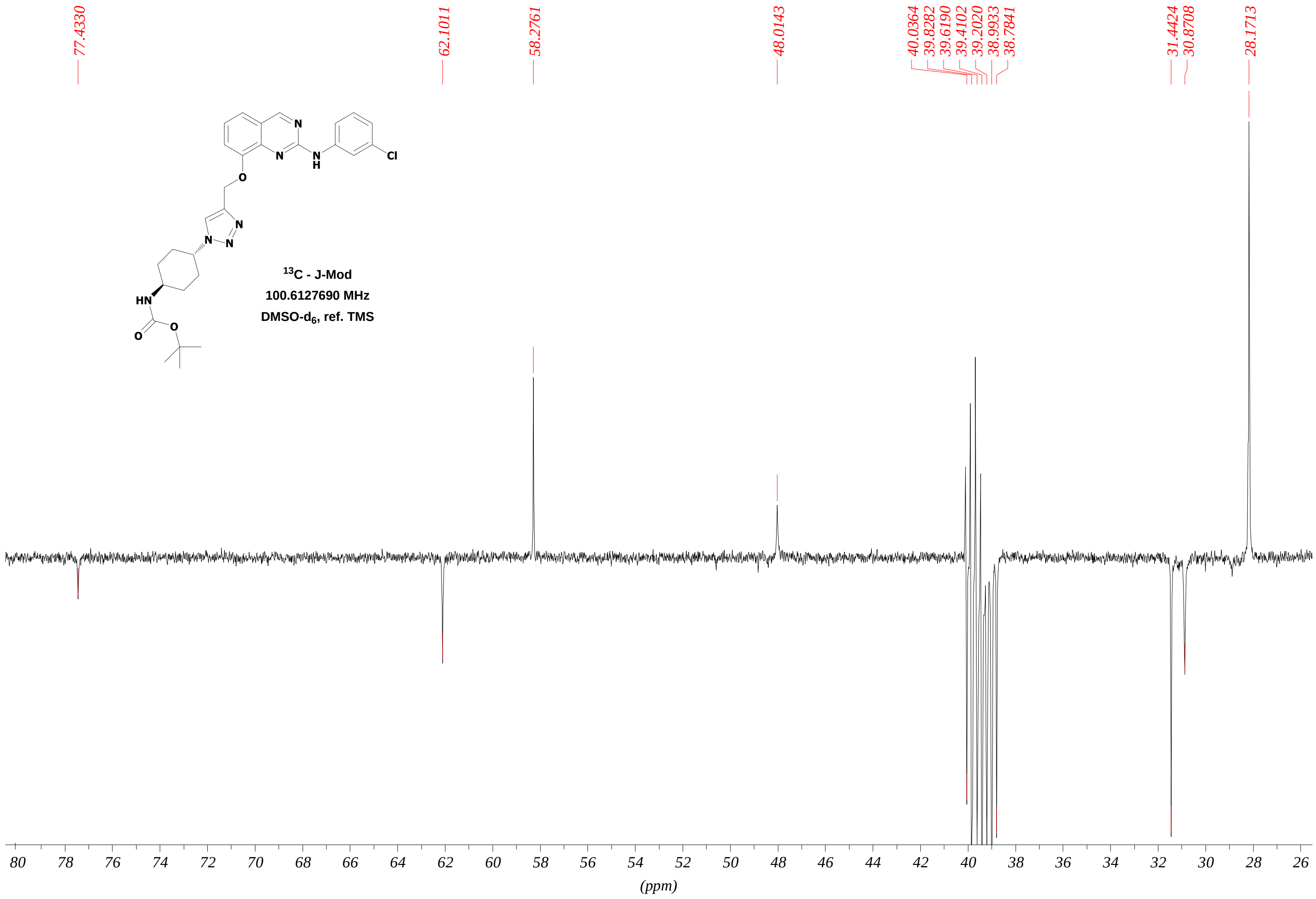

77.4330
62.1011
58.2761
48.0143
40.0364
39.8282
39.6190
39.4102
39.2020
38.9933
38.7841
31.4424
30.8708
28.1713
^{13}C - J-Mod
100.6127690 MHz
DMSO-d_6, ref. TMS
(ppm)

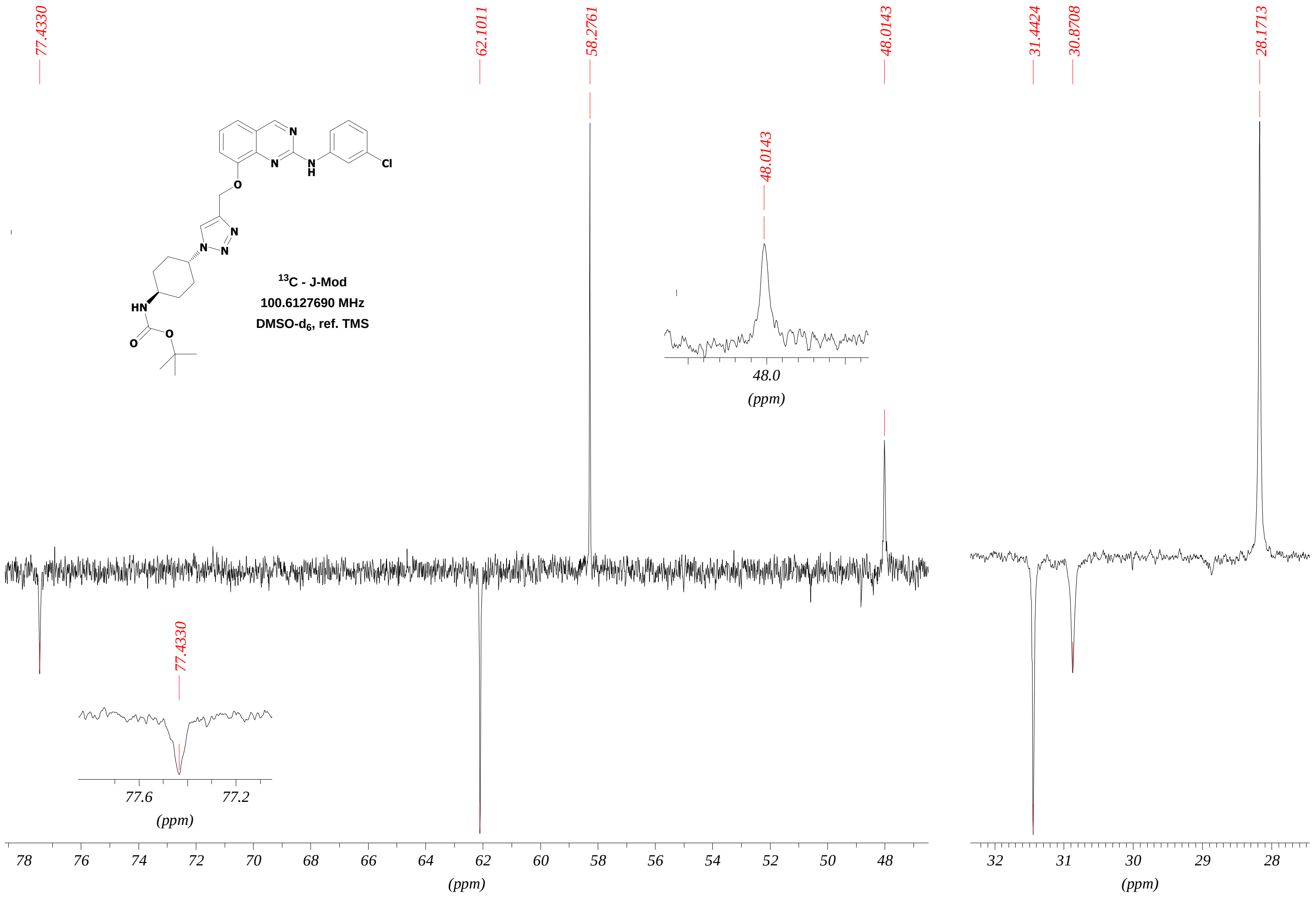

13C - J-Mod
100.6127690 MHz
DMSO-d6, ref. TMS
N
N
N
H
Cl
O
N
N
N
HN
O
O
77.4330
62.1011
58.2761
48.0143
31.4424
30.8708
28.1713
48.0
(ppm)
77.6
77.2
(ppm)
78
76
74
72
70
68
66
64
62
60
58
56
54
52
50
48
(ppm)
32
31
30
29
28
(ppm)

***tert*-Butyl ((*trans*)-4-(4-(((2-((3,5-dichlorophenyl)amino)quinazolin-8-yl)oxy) methyl)-1*H*-1,2,3-triazol-1-yl)cyclohexyl)carbamate (18b):**

Pages S148-S159

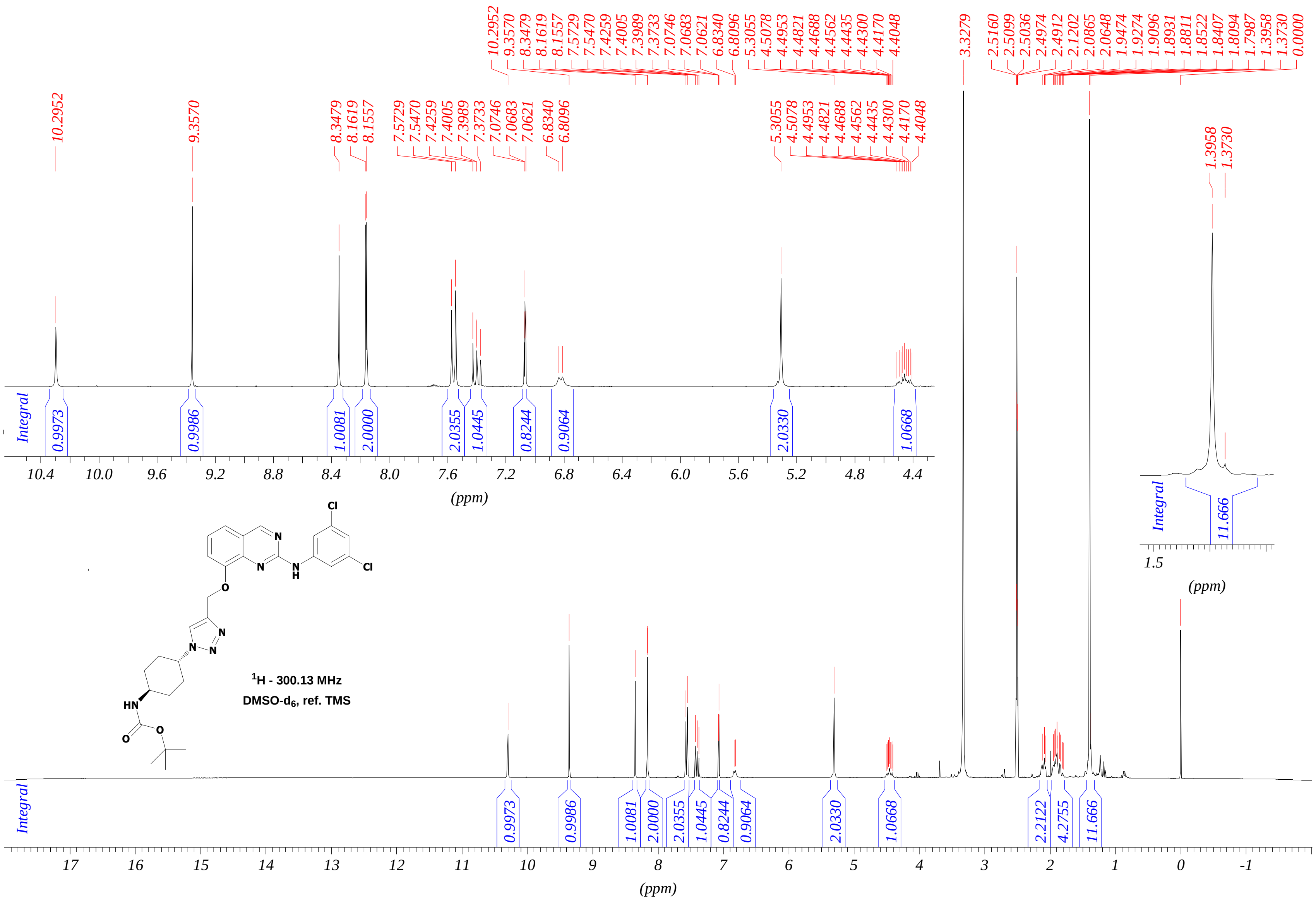

^{1}H - 300.13 MHz
DMSO-d$_6$, ref. TMS
(ppm)
Integral

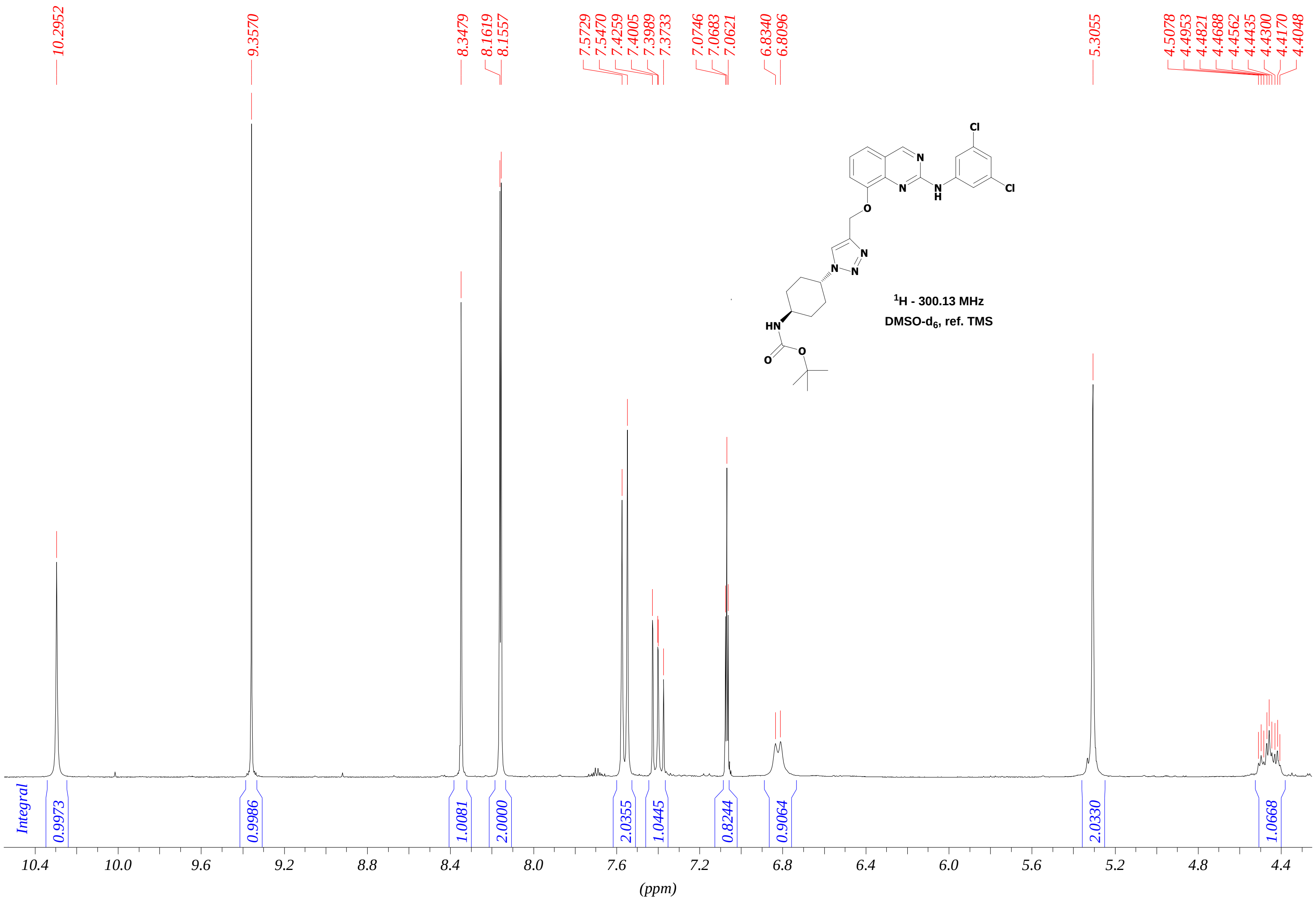
10.2952
9.3570
8.3479
8.1619
8.1557
7.5729
7.5470
7.4259
7.4005
7.3989
7.3733
7.0746
7.0683
7.0621
6.8340
6.8096
5.3055
4.5078
4.4953
4.4821
4.4688
4.4562
4.4435
4.4300
4.4170
4.4048
Cl
N
N
N
H
Cl
O
N
N
N
HN
O
O
^{1}H - 300.13 MHz
DMSO-d$_6$, ref. TMS
Integral
0.9973
0.9986
1.0081
2.0000
2.0355
1.0445
0.8244
0.9064
2.0330
1.0668
10.4
10.0
9.6
9.2
8.8
8.4
8.0
7.6
7.2
6.8
6.4
6.0
5.6
5.2
4.8
4.4
(ppm)

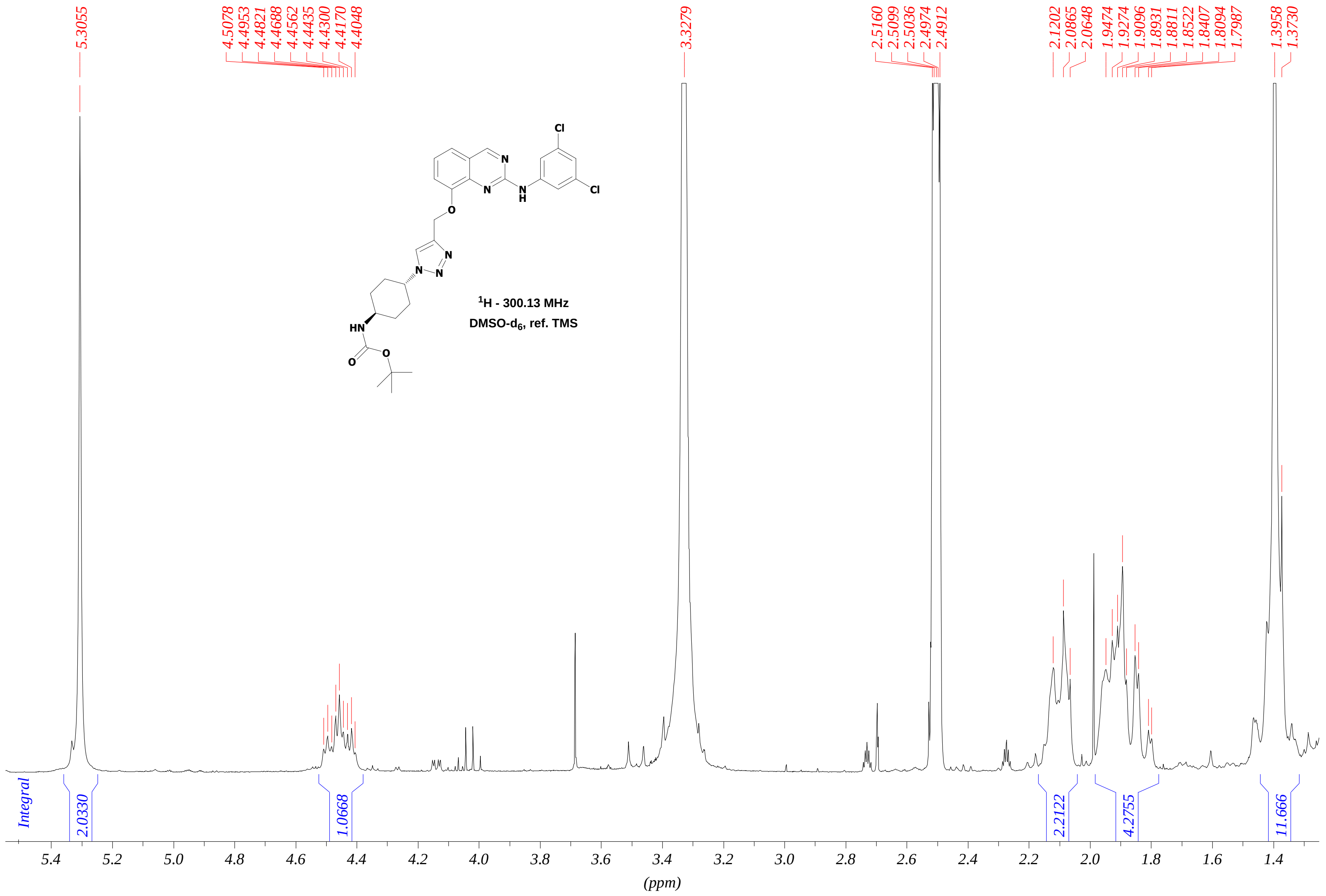

5.3055
4.5078 4.4953 4.4821 4.4688 4.4562 4.4435 4.4300 4.4170 4.4048
3.3279
2.5160 2.5099 2.5036 2.4974 2.4912
2.1202 2.0865 2.0648
1.9474 1.9274 1.9096 1.8931 1.8811 1.8522 1.8407 1.8094 1.7987
1.3958 1.3730
Cl
N
N
N
H
Cl
O
N
N
N
HN
O
O
^{1}H - 300.13 MHz
DMSO-d$_6$, ref. TMS
Integral
2.0330
1.0668
2.2122
4.2755
11.666
5.4 5.2 5.0 4.8 4.6 4.4 4.2 4.0 3.8 3.6 3.4 3.2 3.0 2.8 2.6 2.4 2.2 2.0 1.8 1.6 1.4
(ppm)

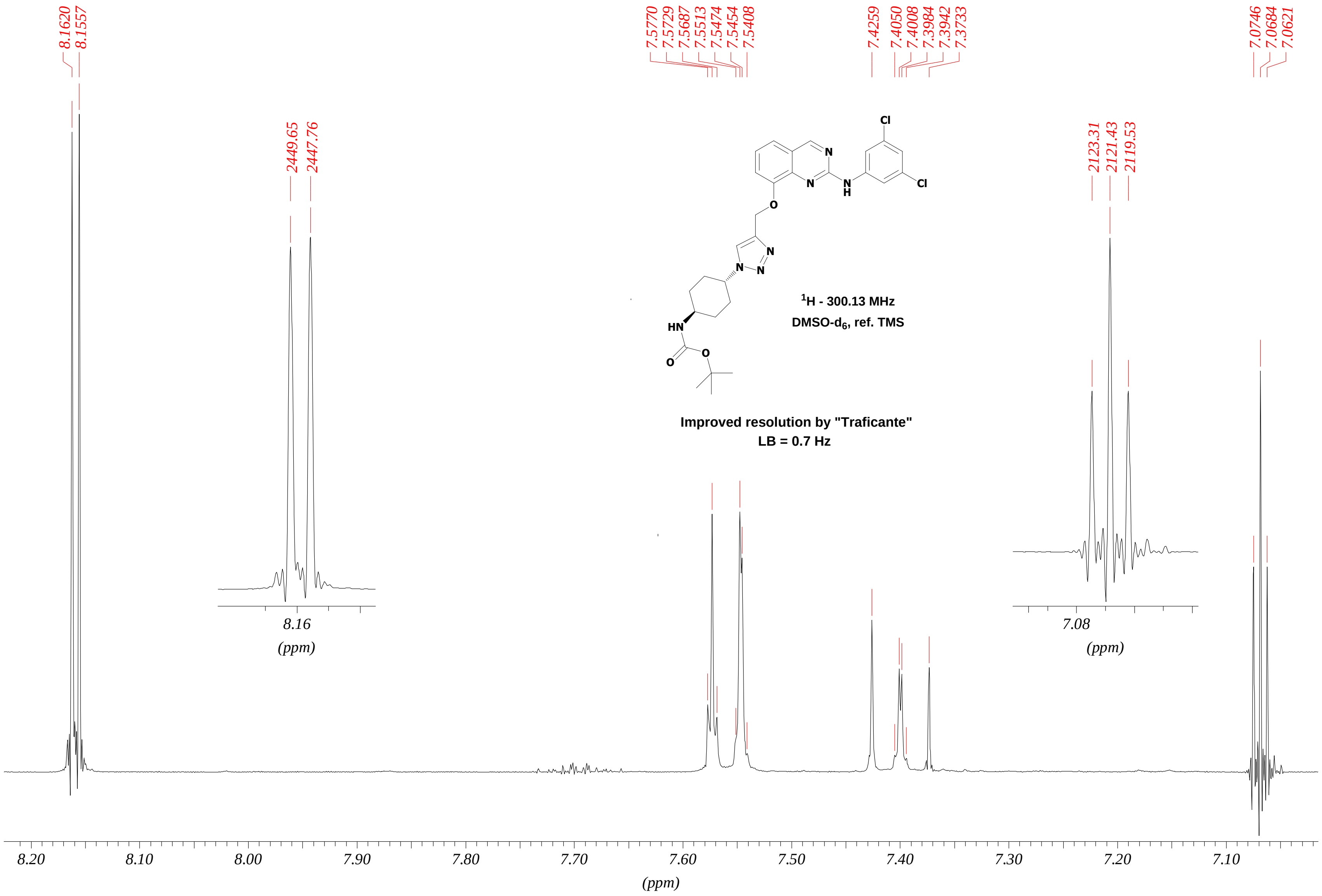
8.1620
8.1557
7.5770
7.5729
7.5687
7.5513
7.5474
7.5454
7.5408
7.4259
7.4050
7.4008
7.3984
7.3942
7.3733
7.0746
7.0684
7.0621
2449.65
2447.76
2123.31
2121.43
2119.53
Cl
N
N
N
H
Cl
O
N
N
N
HN
O
O
1H - 300.13 MHz
DMSO-d6, ref. TMS
Improved resolution by "Traficante"
LB = 0.7 Hz
8.16
(ppm)
7.08
(ppm)
8.20
8.10
8.00
7.90
7.80
7.70
7.60
7.50
7.40
7.30
7.20
7.10
(ppm)

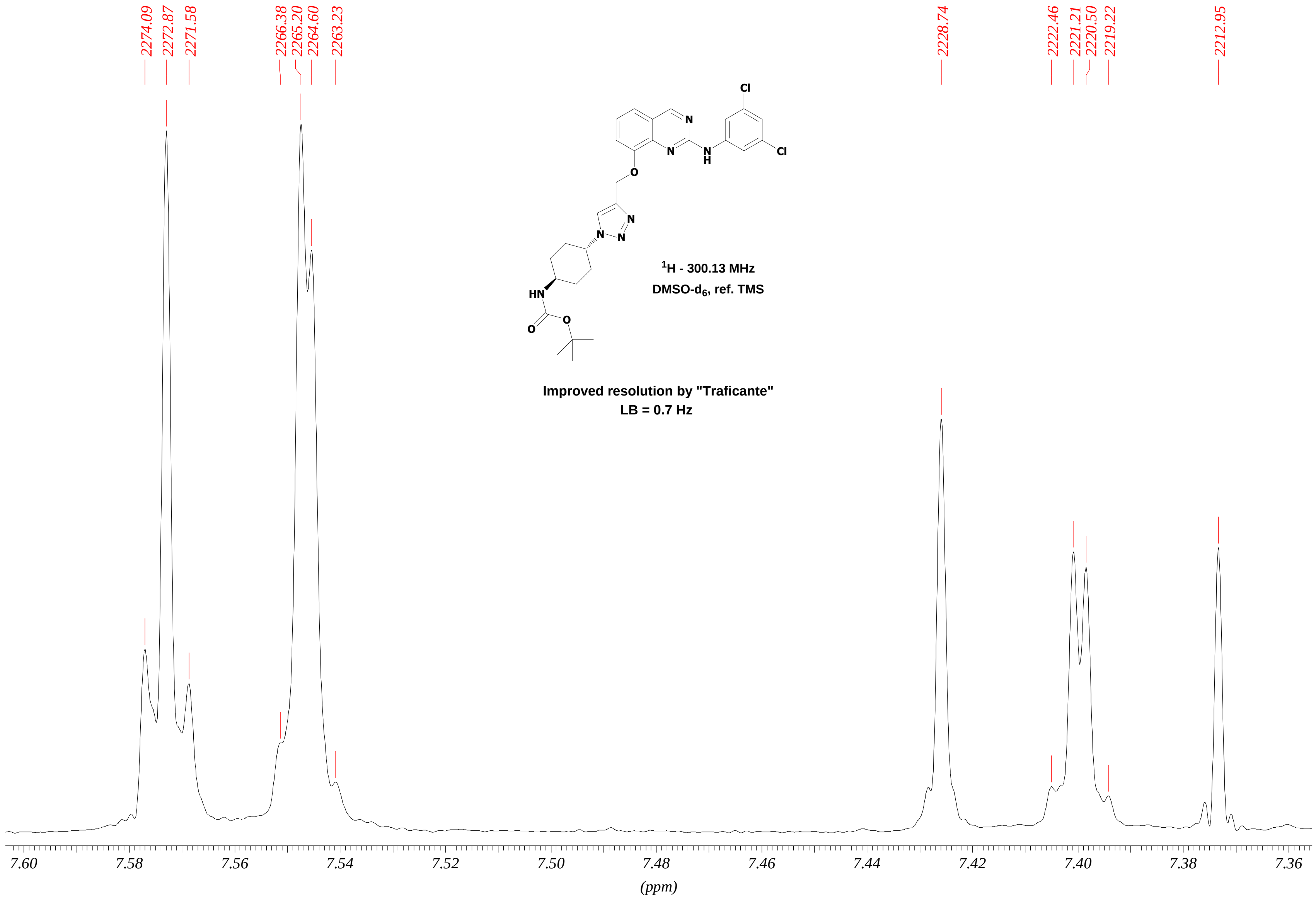
2274.09
2272.87
2271.58
2266.38
2265.20
2264.60
2263.23
2228.74
2222.46
2221.21
2220.50
2219.22
2212.95
Cl
N
N
N
H
Cl
O
N
N
N
HN
O
O
¹H - 300.13 MHz
DMSO-d6, ref. TMS
Improved resolution by "Traficante"
LB = 0.7 Hz
7.60
7.58
7.56
7.54
7.52
7.50
7.48
7.46
7.44
7.42
7.40
7.38
7.36
(ppm)

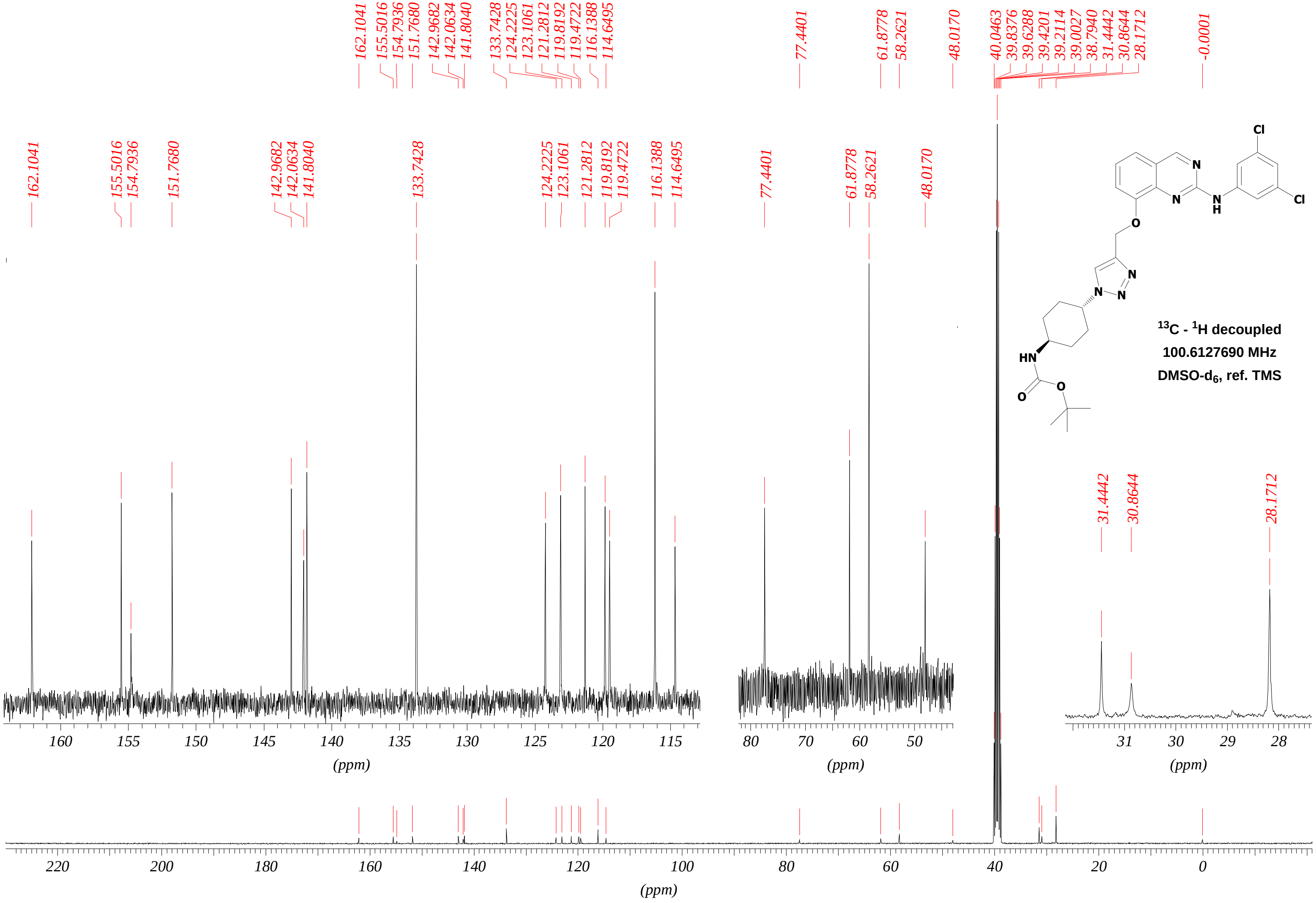

13C - 1H decoupled
100.6127690 MHz
DMSO-d6, ref. TMS
162.1041
155.5016
154.7936
151.7680
142.9682
142.0634
141.8040
133.7428
124.2225
123.1061
121.2812
119.8192
119.4722
116.1388
114.6495
77.4401
61.8778
58.2621
48.0170
40.0463
39.8376
39.6288
39.4201
39.2114
39.0027
38.7940
31.4442
30.8644
28.1712
-0.0001
(ppm)

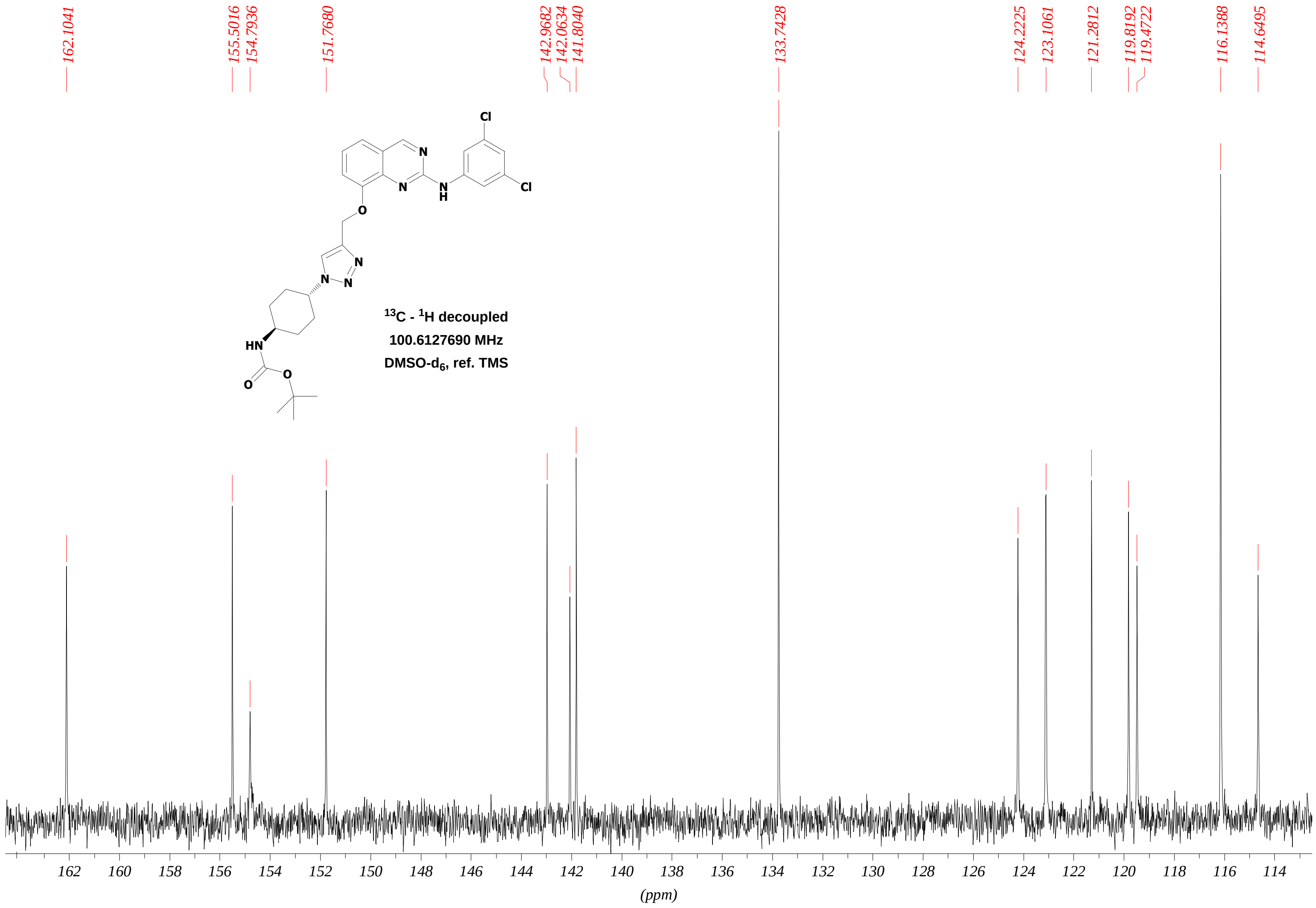

162.1041
155.5016
154.7936
151.7680
142.9682
142.0634
141.8040
133.7428
124.2225
123.1061
121.2812
119.8192
119.4722
116.1388
114.6495
Cl
N
N
N
H
Cl
O
N
N
N
HN
O
O
13C - 1H decoupled
100.6127690 MHz
DMSO-d6, ref. TMS
162
160
158
156
154
152
150
148
146
144
142
140
138
136
134
132
130
128
126
124
122
120
118
116
114
(ppm)

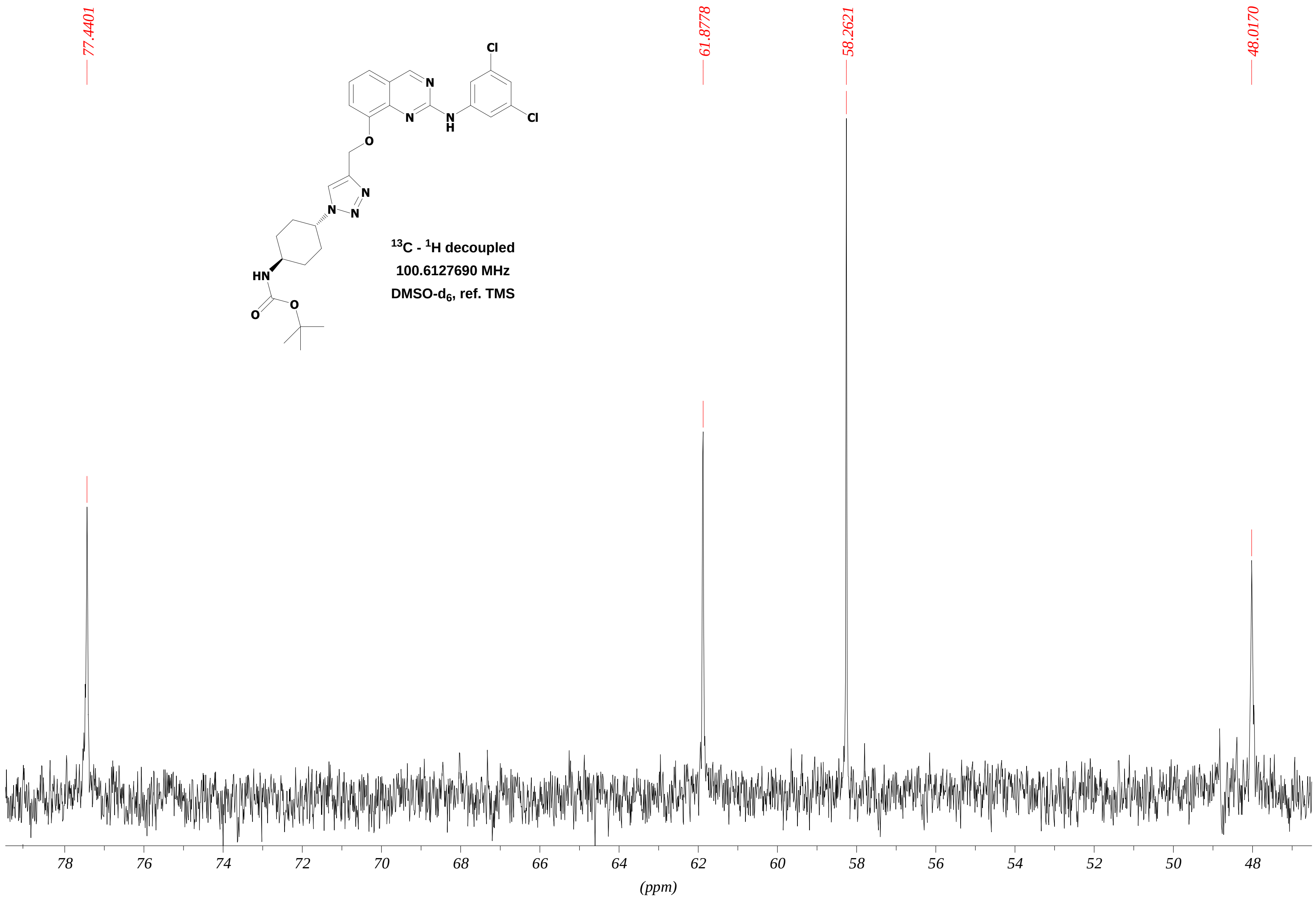

77.4401
61.8778
58.2621
48.0170
Cl
N
N
N
H
Cl
O
N
N
N
HN
O
O
^{13}C - ^{1}H decoupled
100.6127690 MHz
DMSO-d$_6$, ref. TMS
78
76
74
72
70
68
66
64
62
60
58
56
54
52
50
48
(ppm)

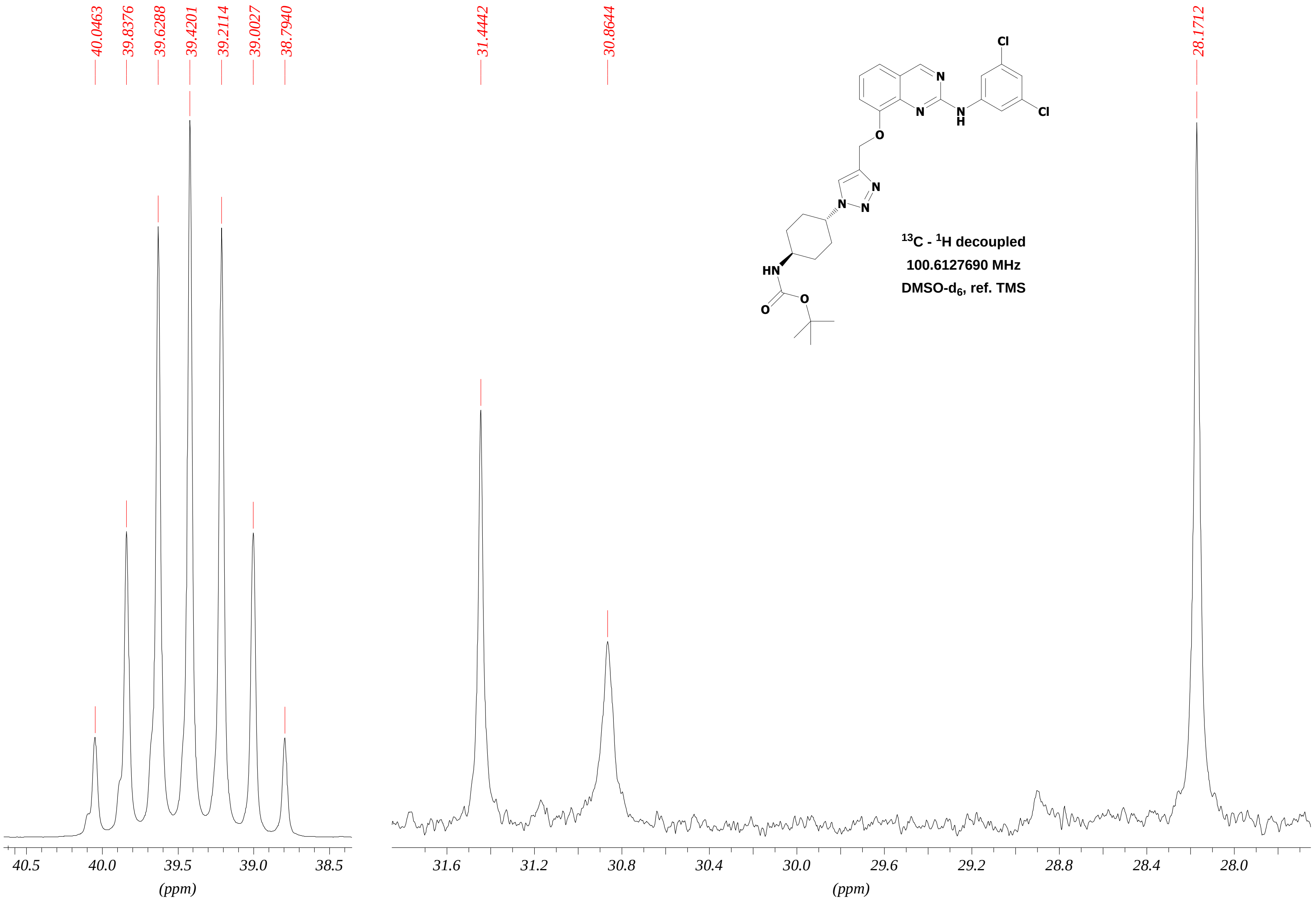
40.0463
39.8376
39.6288
39.4201
39.2114
39.0027
38.7940
31.4442
30.8644
28.1712
Cl
N
N
N
H
Cl
O
N
N
N
HN
O
O
^{13}C - ^{1}H decoupled
100.6127690 MHz
DMSO-d$_6$, ref. TMS
40.5
40.0
39.5
39.0
38.5
(ppm)
31.6
31.2
30.8
30.4
30.0
29.6
29.2
28.8
28.4
28.0
(ppm)

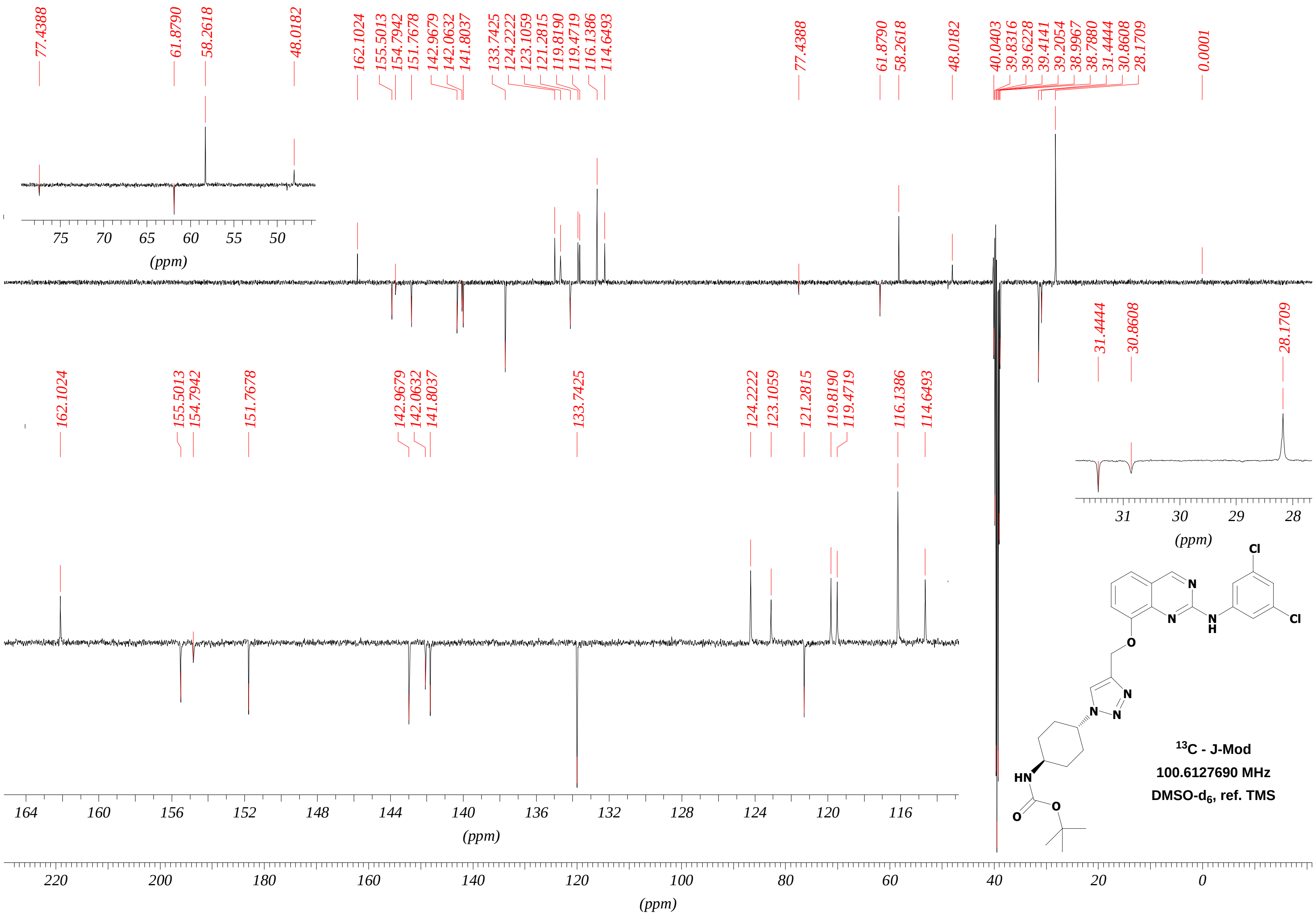

13C - J-Mod
100.6127690 MHz
DMSO-d6, ref. TMS
162.1024
155.5013
154.7942
151.7678
142.9679
142.0632
141.8037
133.7425
124.2222
123.1059
121.2815
119.8190
119.4719
116.1386
114.6493
77.4388
61.8790
58.2618
48.0182
40.0403
39.8316
39.6228
39.4141
39.2054
38.9967
38.7880
31.4444
30.8608
28.1709
0.0001
(ppm)

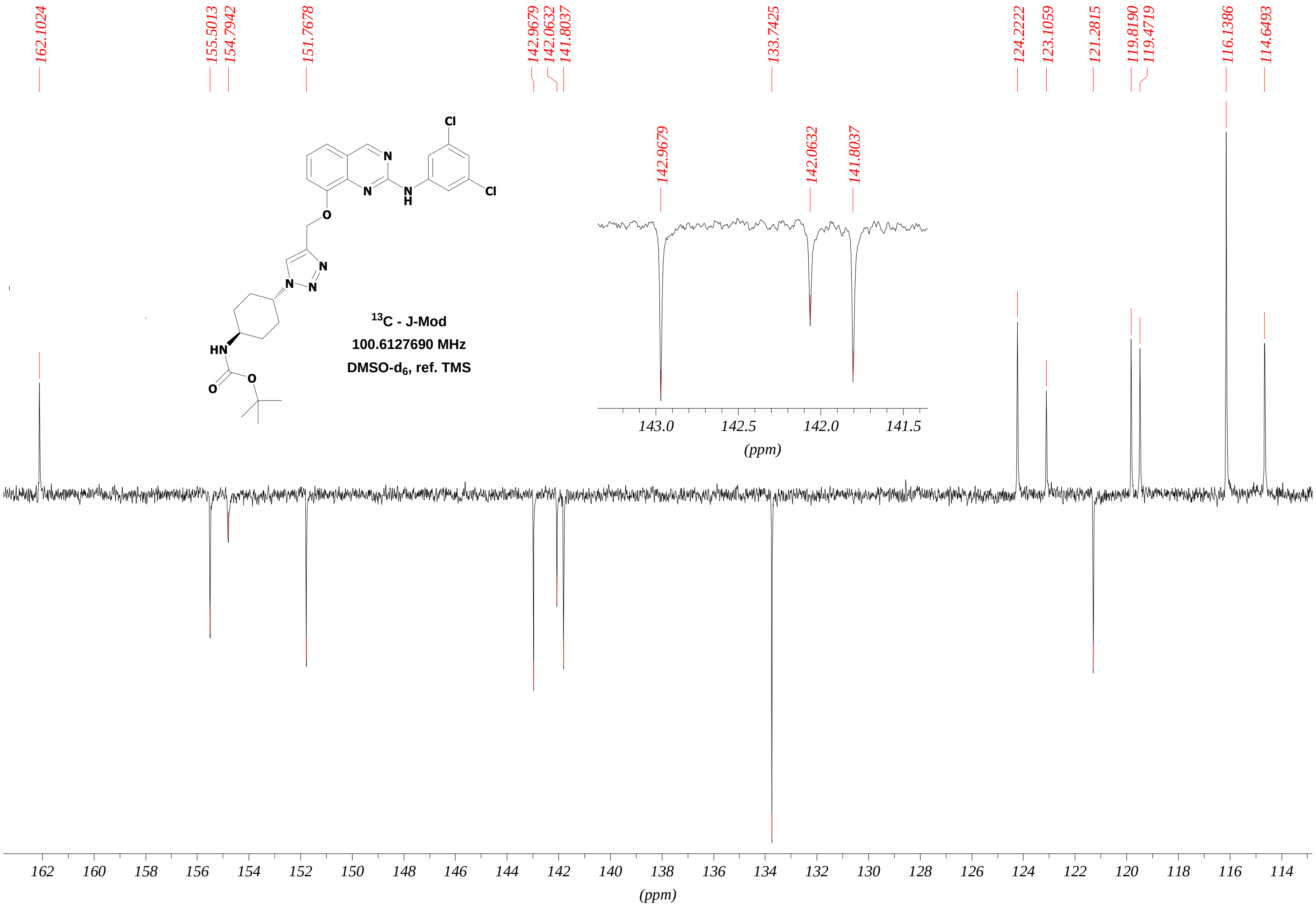

162.1024
155.5013
154.7942
151.7678
142.9679
142.0632
141.8037
133.7425
124.2222
123.1059
121.2815
119.8190
119.4719
116.1386
114.6493
Cl
N
N
N
H
Cl
O
N
N
N
HN
O
O
13C - J-Mod
100.6127690 MHz
DMSO-d6, ref. TMS
143.0
142.5
142.0
141.5
(ppm)
162
160
158
156
154
152
150
148
146
144
142
140
138
136
134
132
130
128
126
124
122
120
118
116
114
(ppm)

8-((1-((*cis*)-4-Aminocyclohexyl)-1*H*-1,2,3-triazol-4-yl)methoxy)-*N*-(3-chlorophenyl)quinazolin-2-amine hydrochloride (16a):

Pages S160-S165

A722-011D

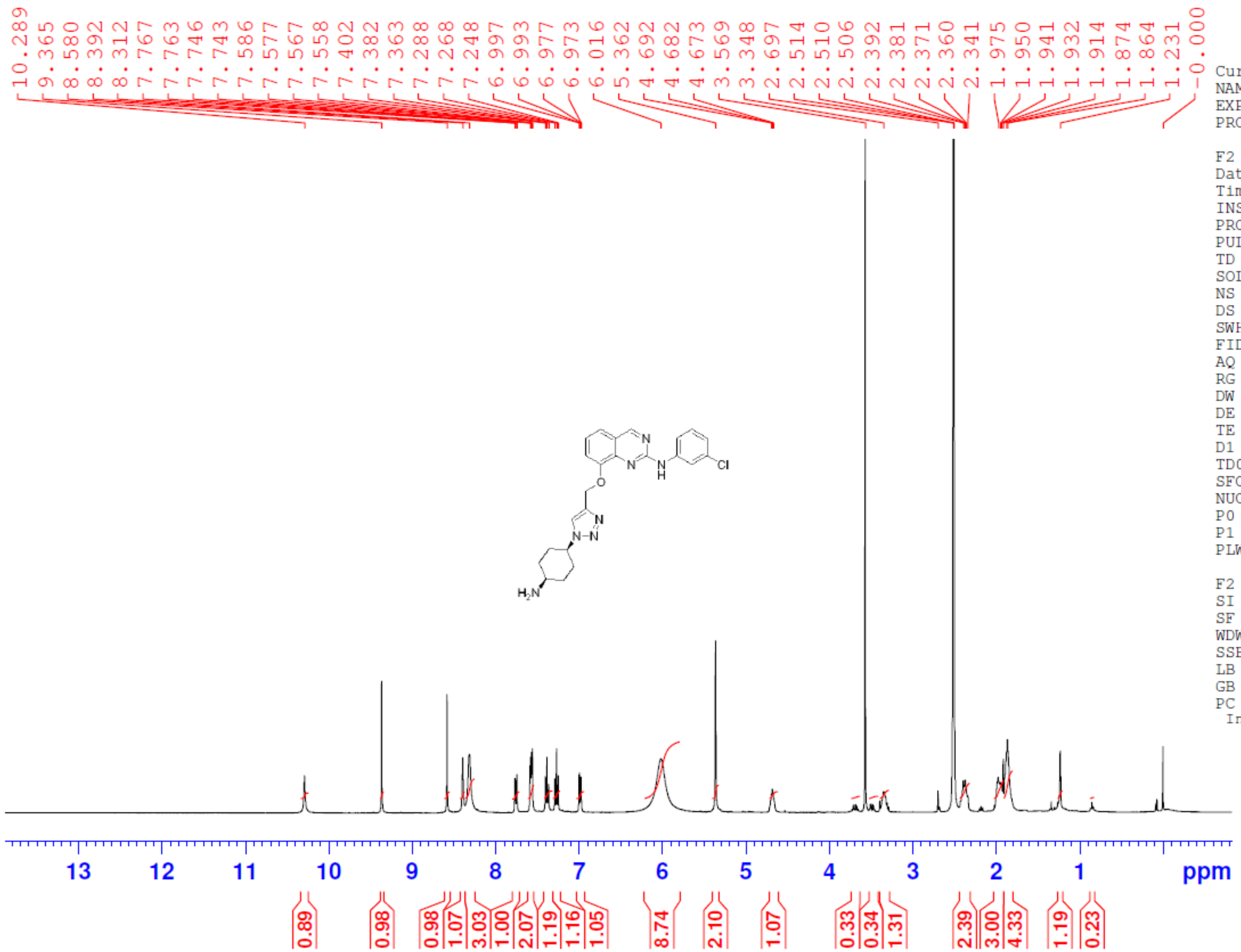

Current Data Parameters
NAME A722-011D
EXPNO 1
PROCNO 1

F2 - Acquisition Parameters
Date_ 20200301
Time 2.46 h
INSTRUM spect
PROBHD Z150453_0004 (
PULPROG zg30
TD 65536
SOLVENT DMSO
NS 16
DS 2
SWH 8012.820 Hz
FIDRES 0.244532 Hz
AQ 4.0894465 sec
RG 147.5
DW 62.400 usec
DE 6.50 usec
TE 298.2 K
D1 1.00000000 sec
TD0 1
SFO1 400.1324708 MHz
NUC1 1H
P0 3.33 usec
P1 10.00 usec
PLW1 16.87899971 W

F2 - Processing parameters
SI 65536
SF 400.1299990 MHz
WDW EM
SSB 0
LB 0.30 Hz
GB 0
PC 1.00
Inst.ID: CVL1-AD-NMR-001

Analyzed By: Harinath

Checked by:

A722-011D

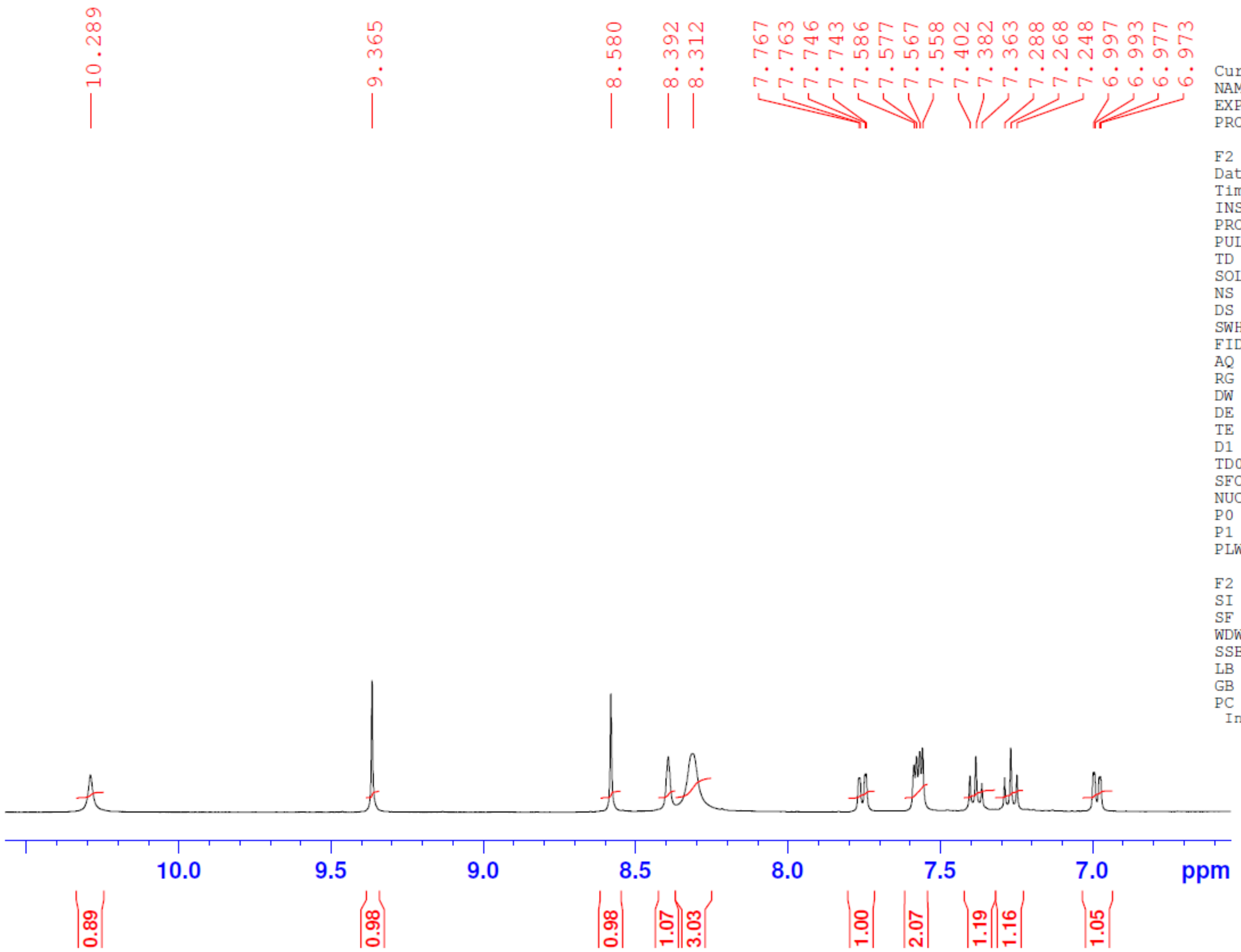


```
Current Data Parameters
NAME             A722-011D
EXPNO                    1
PROCNO                   1

F2 - Acquisition Parameters
Date_             20200301
Time                  2.46 h
INSTRUM              spect
PROBHD    Z150453_0004 (
PULPROG               zg30
TD                   65536
SOLVENT               DMSO
NS                      16
DS                       2
SWH               8012.820 Hz
FIDRES            0.244532 Hz
AQ               4.0894465 sec
RG                   147.5
DW                  62.400 usec
DE                    6.50 usec
TE                   298.2 K
D1              1.00000000 sec
TD0                      1
SFO1           400.1324708 MHz
NUC1                    1H
P0                    3.33 usec
P1                   10.00 usec
PLW1           16.87899971 W

F2 - Processing parameters
SI                   65536
SF             400.1299990 MHz
WDW                     EM
SSB                      0
LB                    0.30 Hz
GB                       0
PC                    1.00
 Inst.ID:  CVL1-AD-NMR-001
```

Analyzed By: Harinath

Checked by:

A722-011D

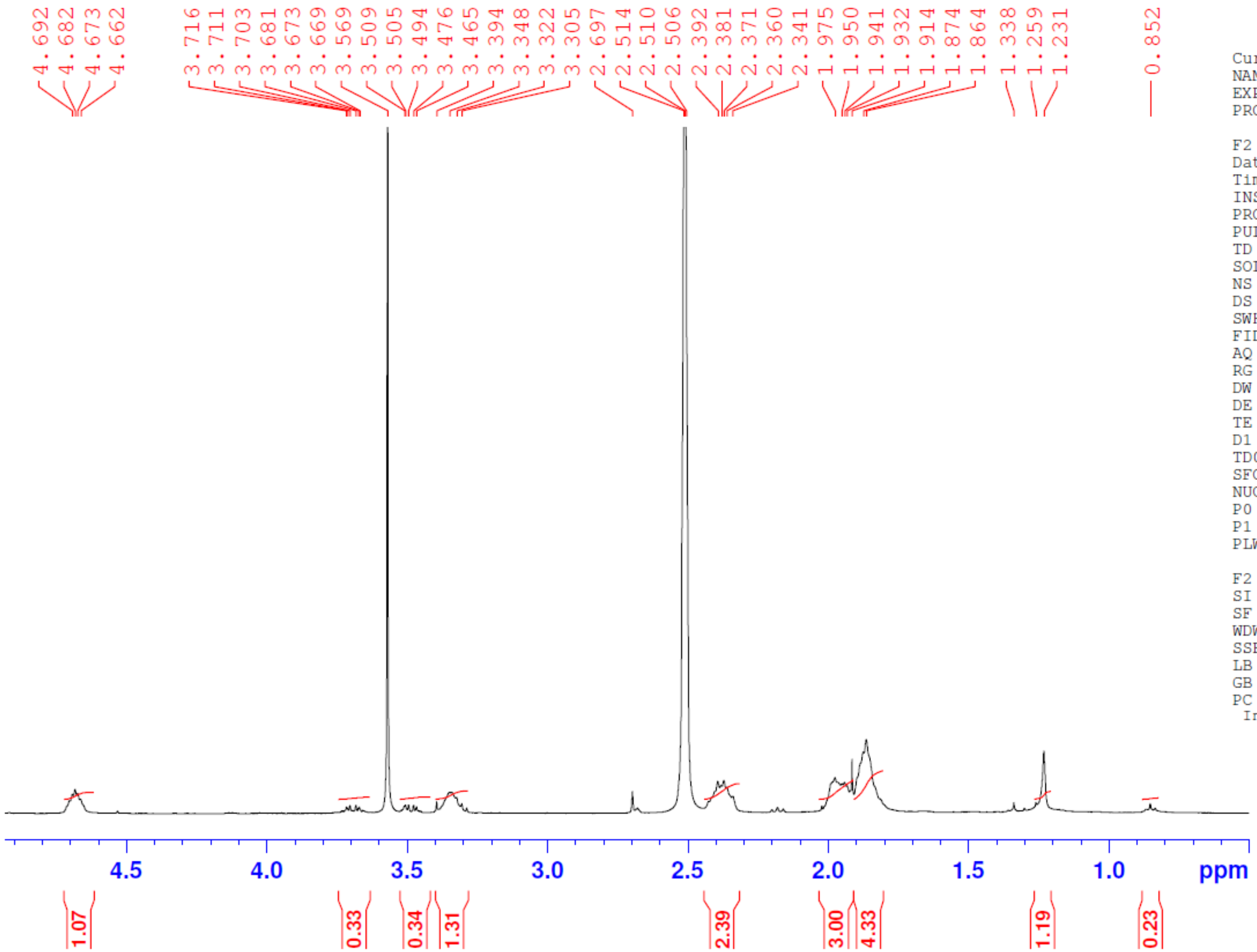


```
Current Data Parameters
NAME              A722-011D
EXPNO                     1
PROCNO                    1

F2 - Acquisition Parameters
Date_              20200301
Time                   2.46 h
INSTRUM               spect
PROBHD    Z150453_0004 (
PULPROG                zg30
TD                    65536
SOLVENT                DMSO
NS                       16
DS                        2
SWH                8012.820 Hz
FIDRES             0.244532 Hz
AQ                4.0894465 sec
RG                    147.5
DW                   62.400 usec
DE                     6.50 usec
TE                    298.2 K
D1               1.00000000 sec
TD0                       1
SFO1            400.1324708 MHz
NUC1                     1H
P0                     3.33 usec
P1                    10.00 usec
PLW1            16.87899971 W

F2 - Processing parameters
SI                    65536
SF              400.1299990 MHz
WDW                      EM
SSB                       0
LB                     0.30 Hz
GB                        0
PC                     1.00
 Inst.ID:   CVL1-AD-NMR-001
```

Analyzed By: Harinath

Checked by:

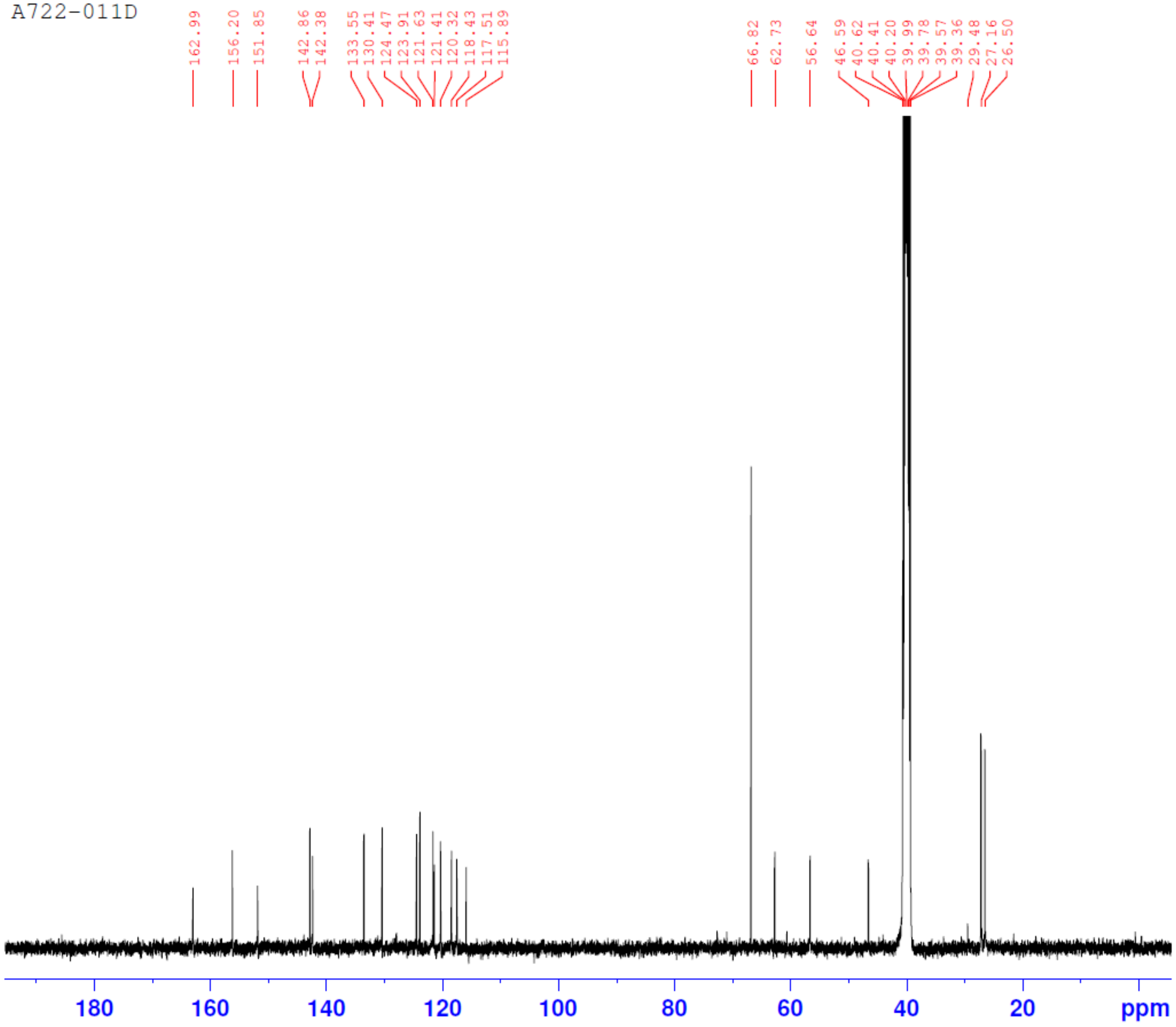

Current Data Parameters
NAME A722-011D
EXPNO 2
PROCNO 1

F2 - Acquisition Parameters
Date_ 20200301
Time 6.36 h
INSTRUM spect
PROBHD Z150453_0004 (
PULPROG zgpg30
TD 65536
SOLVENT DMSO
NS 4000
DS 4
SWH 24038.461 Hz
FIDRES 0.733596 Hz
AQ 1.3631488 sec
RG 204.77
DW 20.800 usec
DE 6.50 usec
TE 299.0 K
D1 2.00000000 sec
D11 0.03000000 sec
TD0 1
SFO1 100.6228298 MHz
NUC1 13C
P0 3.33 usec
P1 10.00 usec
PLW1 66.26200104 W
SFO2 400.1316005 MHz
NUC2 1H
CPDPRG[2 waltz65
PCPD2 90.00 usec
PLW2 16.87899971 W
PLW12 0.20839000 W
PLW13 0.10482000 W

F2 - Processing parameters
SI 32768
SF 100.6127685 MHz
WDW EM
SSB 0
LB 1.00 Hz
GB 0
PC 1.40
Inst.ID: CVL1-AD-NMR-001

Analyzed By:

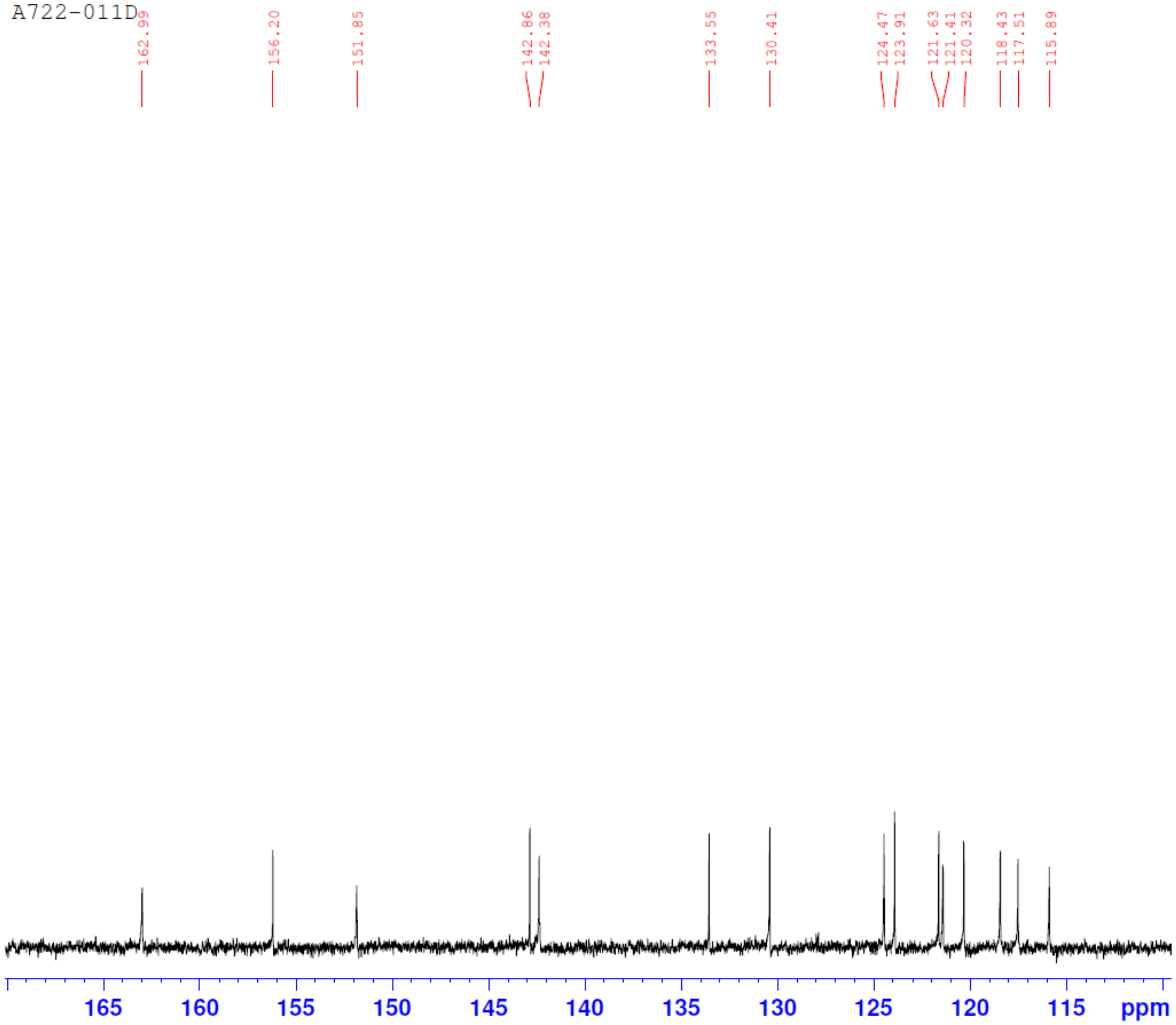

Chemveda Life Sciences

Current Data Parameters
NAME A722-011D
EXPNO 2
PROCNO 1

F2 - Acquisition Parameters
Date_ 20200301
Time 6.36 h
INSTRUM spect
PROBHD Z150453_0004 (
PULPROG zgpg30
TD 65536
SOLVENT DMSO
NS 4000
DS 4
SWH 24038.461 Hz
FIDRES 0.733596 Hz
AQ 1.3631488 sec
RG 204.77
DW 20.800 usec
DE 6.50 usec
TE 299.0 K
D1 2.00000000 sec
D11 0.03000000 sec
TD0 1
SFO1 100.6228298 MHz
NUC1 13C
P0 3.33 usec
P1 10.00 usec
PLW1 66.26200104 W
SFO2 400.1316005 MHz
NUC2 1H
CPDPRG[2 waltz65
PCPD2 90.00 usec
PLW2 16.87899971 W
PLW12 0.20839000 W
PLW13 0.10482000 W

F2 - Processing parameters
SI 32768
SF 100.6127685 MHz
WDW EM
SSB 0
LB 1.00 Hz
GB 0
PC 1.40
Inst.ID: CVL1-AD-NMR-001

Analyzed By:

8-((1-((*cis*)-4-Aminocyclohexyl)-1*H*-1,2,3-triazol-4-yl)methoxy)-*N*-(3,5-dichlorophenyl)quinazolin-2-amine hydrochloride (16b):

Pages S166-S171

A722-013D

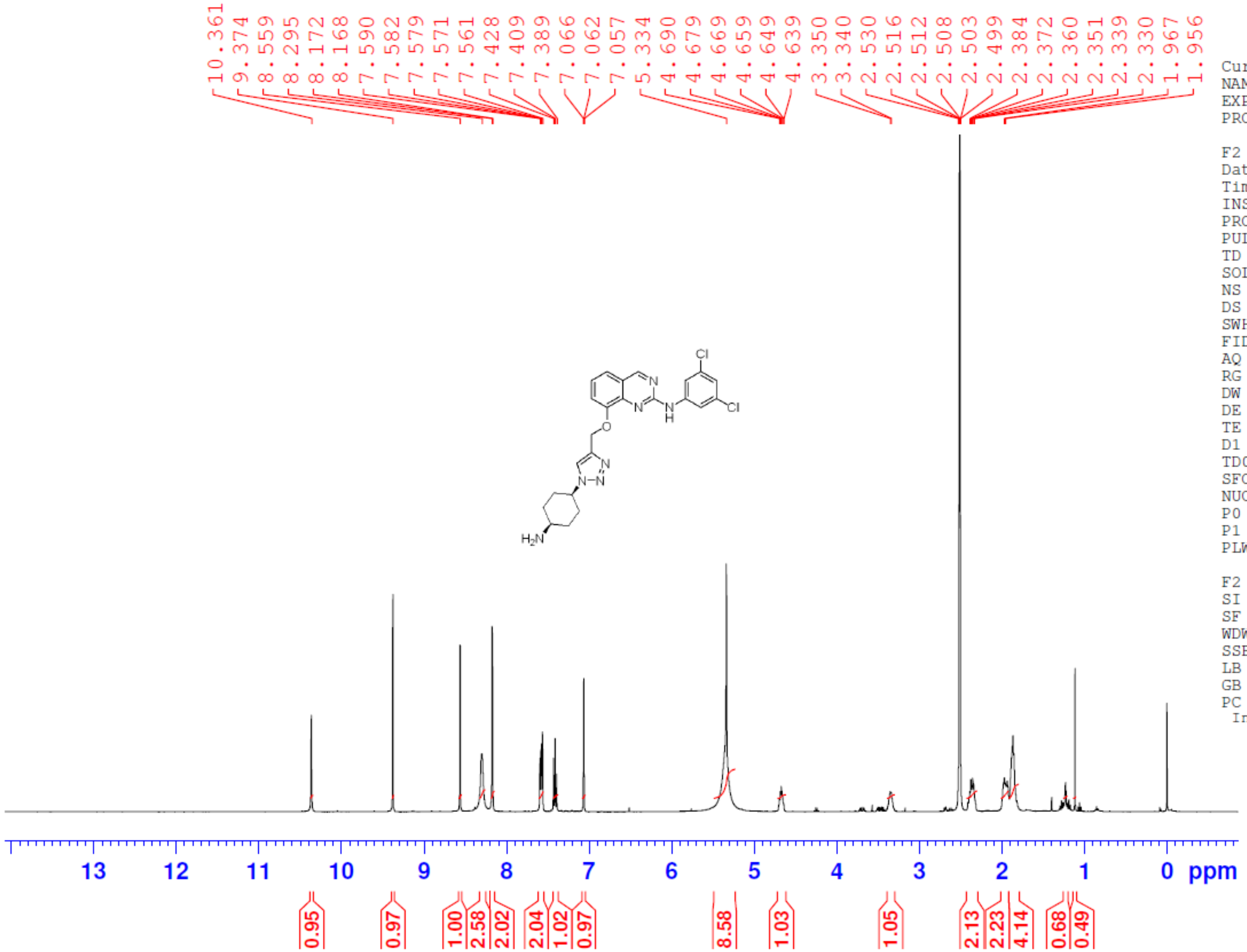


```
Current Data Parameters
NAME            A722-013D
EXPNO                   1
PROCNO                  1

F2 - Acquisition Parameters
Date_            20200229
Time                22.52 h
INSTRUM             spect
PROBHD     Z150453_0004 (
PULPROG              zg30
TD                  65536
SOLVENT              DMSO
NS                     16
DS                      2
SWH              8012.820 Hz
FIDRES           0.244532 Hz
AQ              4.0894465 sec
RG                  147.5
DW                 62.400 usec
DE                   6.50 usec
TE                  298.2 K
D1             1.00000000 sec
TD0                     1
SFO1          400.1324708 MHz
NUC1                   1H
P0                   3.33 usec
P1                  10.00 usec
PLW1          16.87899971 W

F2 - Processing parameters
SI                  65536
SF            400.1300000 MHz
WDW                    EM
SSB                     0
LB                   0.30 Hz
GB                      0
PC                   1.00
 Inst.ID:   CVL1-AD-NMR-001
```

Analyzed By: Harinath

Checked by:

A722-013D

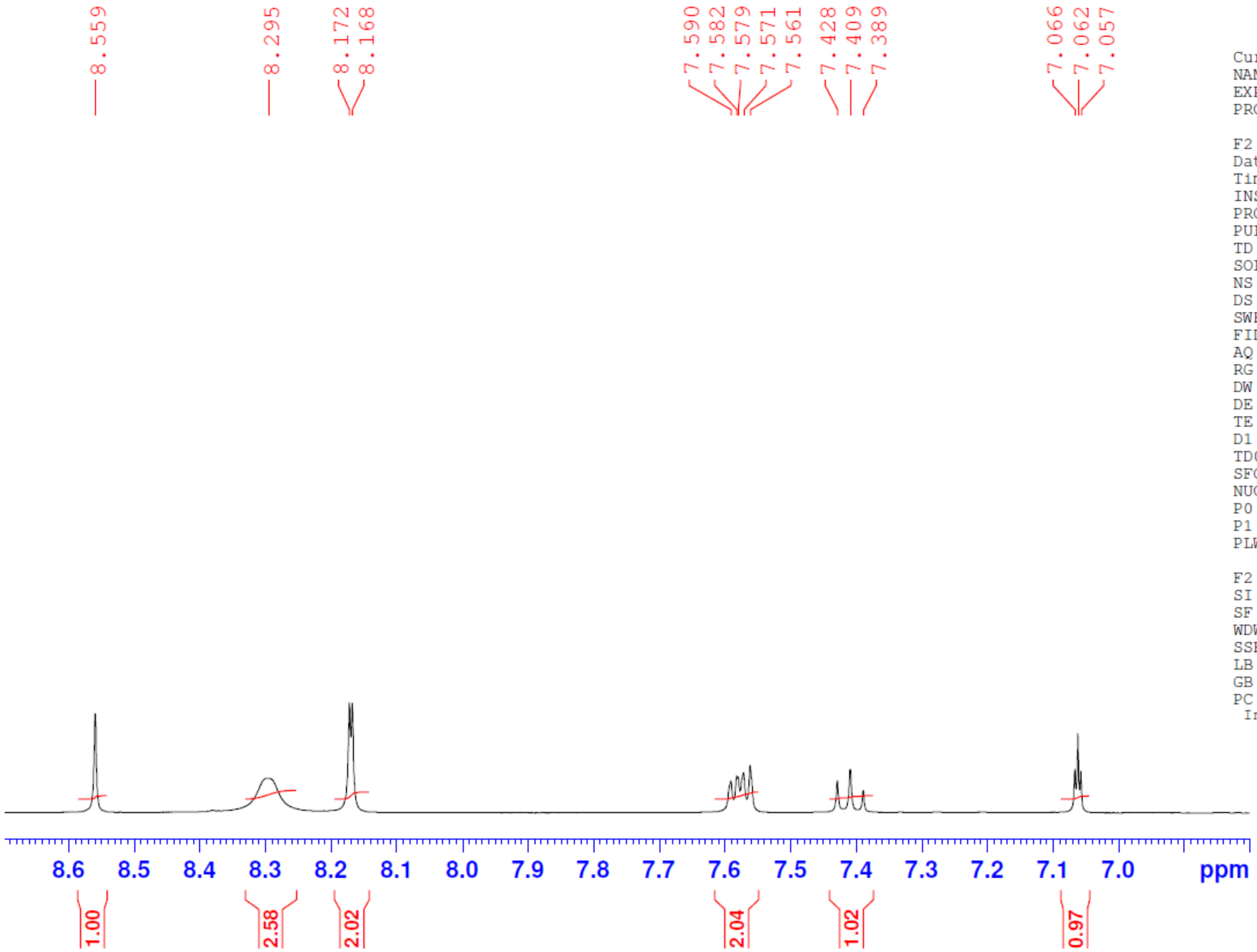

Current Data Parameters
NAME A722-013D
EXPNO 1
PROCNO 1

F2 - Acquisition Parameters
Date_ 20200229
Time 22.52 h
INSTRUM spect
PROBHD Z150453_0004 (
PULPROG zg30
TD 65536
SOLVENT DMSO
NS 16
DS 2
SWH 8012.820 Hz
FIDRES 0.244532 Hz
AQ 4.0894465 sec
RG 147.5
DW 62.400 usec
DE 6.50 usec
TE 298.2 K
D1 1.00000000 sec
TD0 1
SFO1 400.1324708 MHz
NUC1 1H
P0 3.33 usec
P1 10.00 usec
PLW1 16.87899971 W

F2 - Processing parameters
SI 65536
SF 400.1300000 MHz
WDW EM
SSB 0
LB 0.30 Hz
GB 0
PC 1.00
Inst.ID: CVL1-AD-NMR-001

Analyzed By: Harinath

Checked by:

A722-013D

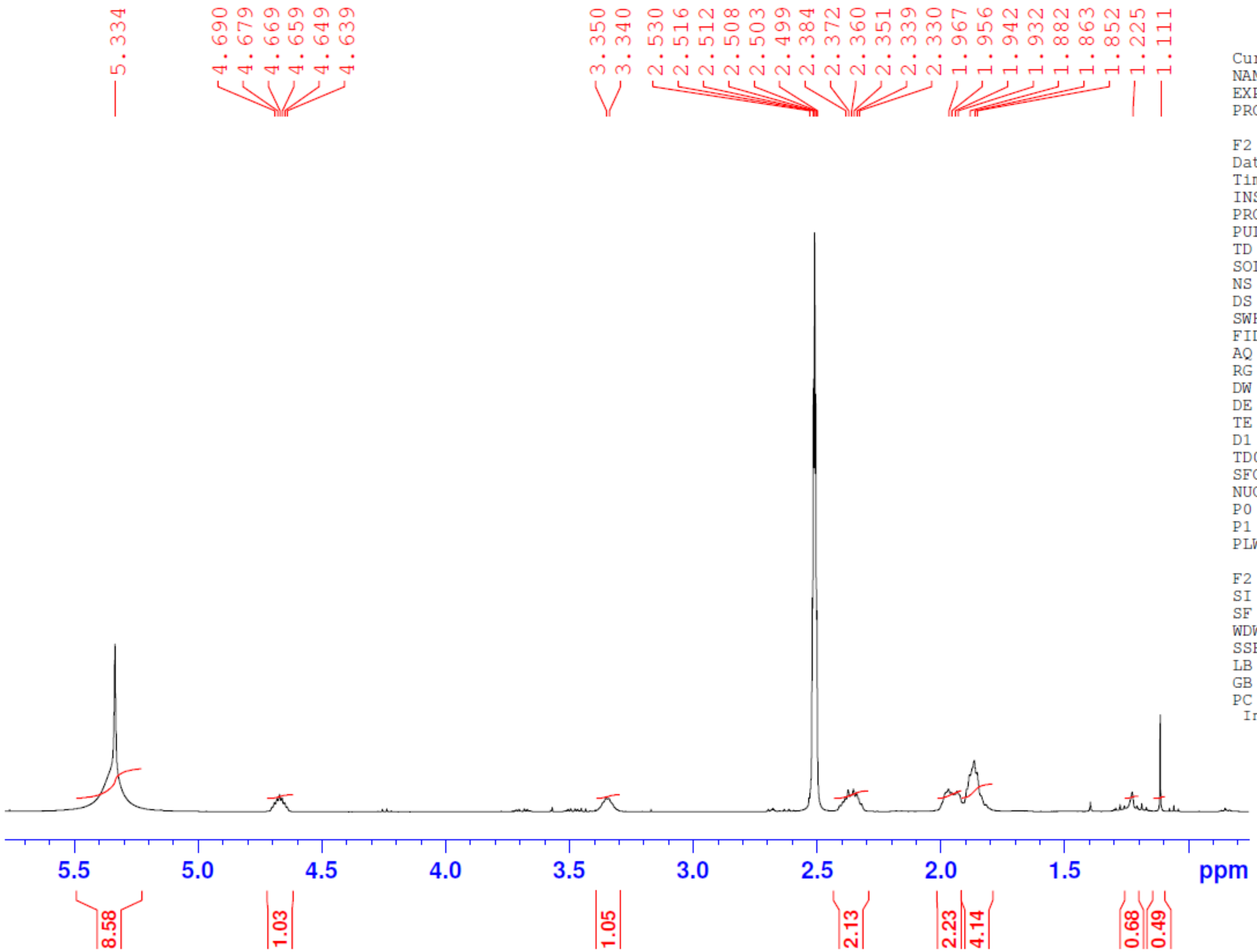

Current Data Parameters
NAME A722-013D
EXPNO 1
PROCNO 1

F2 - Acquisition Parameters
Date_ 20200229
Time 22.52 h
INSTRUM spect
PROBHD Z150453_0004 (
PULPROG zg30
TD 65536
SOLVENT DMSO
NS 16
DS 2
SWH 8012.820 Hz
FIDRES 0.244532 Hz
AQ 4.0894465 sec
RG 147.5
DW 62.400 usec
DE 6.50 usec
TE 298.2 K
D1 1.00000000 sec
TD0 1
SFO1 400.1324708 MHz
NUC1 1H
P0 3.33 usec
P1 10.00 usec
PLW1 16.87899971 W

F2 - Processing parameters
SI 65536
SF 400.1300000 MHz
WDW EM
SSB 0
LB 0.30 Hz
GB 0
PC 1.00
Inst.ID: CVL1-AD-NMR-001

Analyzed By: Harinath

Checked by:

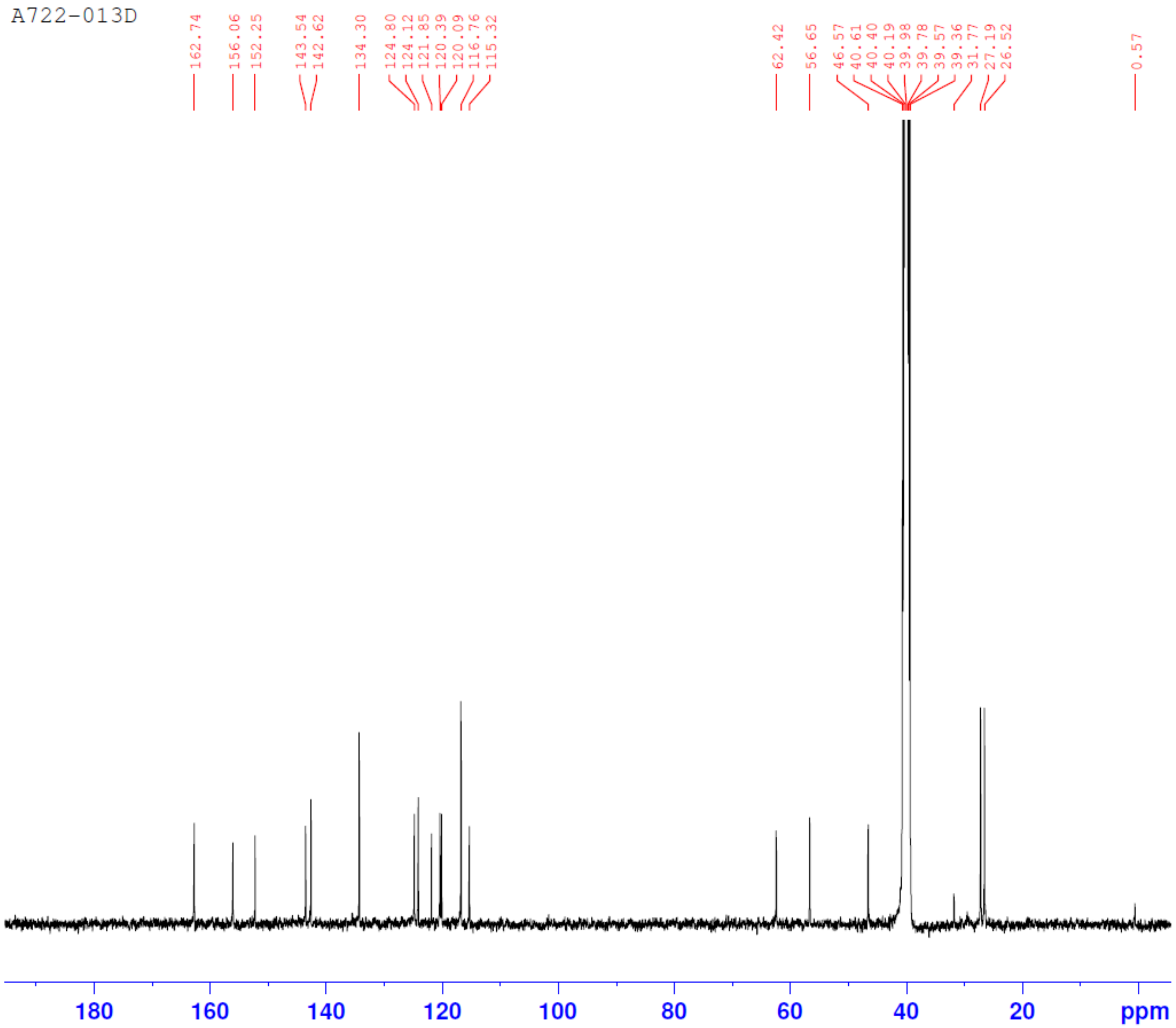

Current Data Parameters

NAME	A722-013D
EXPNO	2
PROCNO	1

F2 - Acquisition Parameters

Date_	20200301
Time	2.42 h
INSTRUM	spect
PROBHD	Z150453_0004 (
PULPROG	zgpg30
TD	65536
SOLVENT	DMSO
NS	4000
DS	4
SWH	24038.461 Hz
FIDRES	0.733596 Hz
AQ	1.3631488 sec
RG	204.77
DW	20.800 usec
DE	6.50 usec
TE	298.2 K
D1	2.00000000 sec
D11	0.03000000 sec
TD0	1
SFO1	100.6228298 MHz
NUC1	13C
P0	3.33 usec
P1	10.00 usec
PLW1	66.26200104 W
SFO2	400.1316005 MHz
NUC2	1H
CPDPRG[2	waltz65
PCPD2	90.00 usec
PLW2	16.87899971 W
PLW12	0.20839000 W
PLW13	0.10482000 W

F2 - Processing parameters

SI	32768
SF	100.6127685 MHz
WDW	EM
SSB	0
LB	3.00 Hz
GB	0
PC	1.40

Inst.ID: CVL1-AD-NMR-001

Analyzed By:

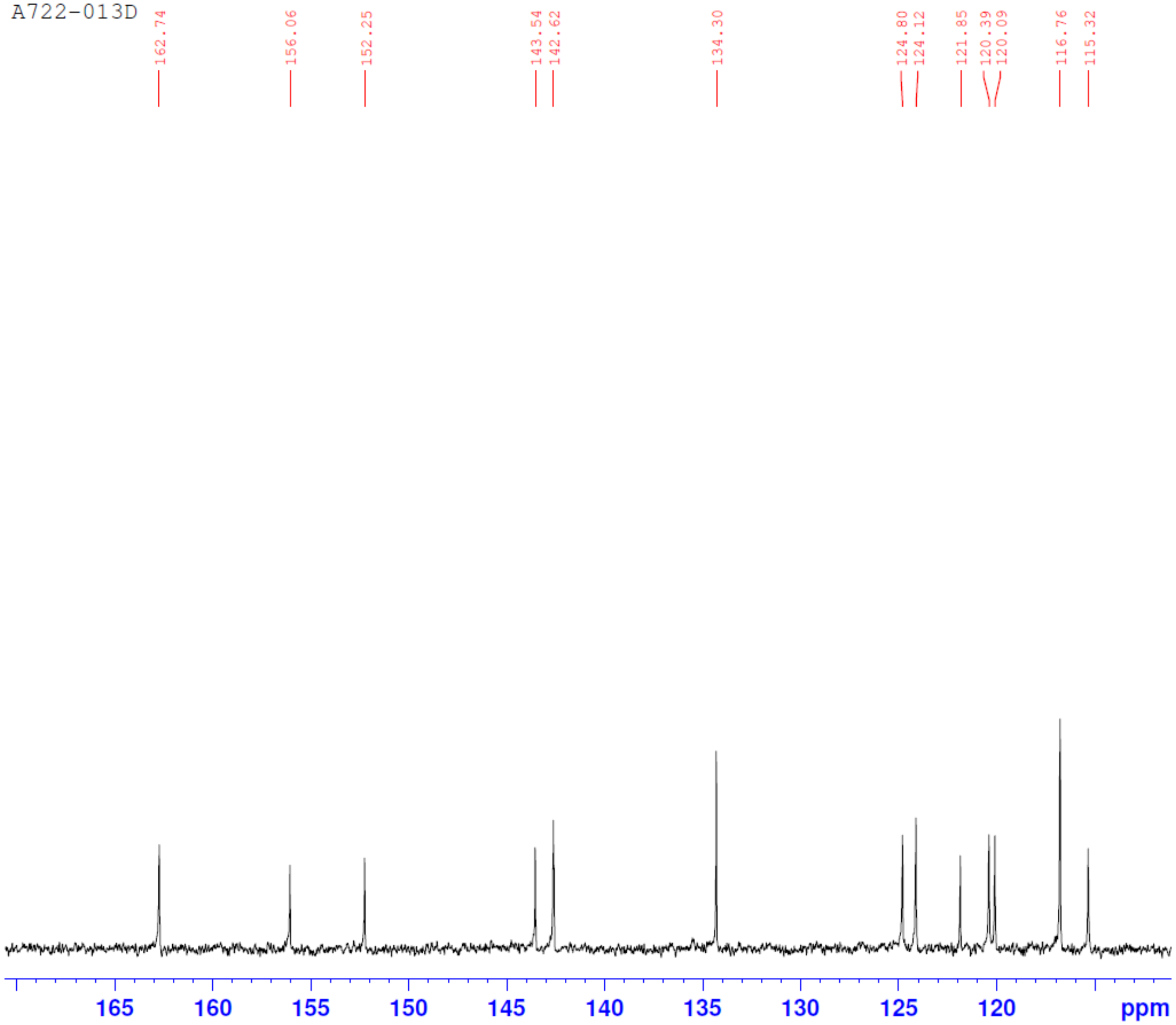

Current Data Parameters
NAME A722-013D
EXPNO 2
PROCNO 1

F2 - Acquisition Parameters
Date_ 20200301
Time 2.42 h
INSTRUM spect
PROBHD Z150453_0004 (
PULPROG zgpg30
TD 65536
SOLVENT DMSO
NS 4000
DS 4
SWH 24038.461 Hz
FIDRES 0.733596 Hz
AQ 1.3631488 sec
RG 204.77
DW 20.800 usec
DE 6.50 usec
TE 298.2 K
D1 2.00000000 sec
D11 0.03000000 sec
TD0 1
SFO1 100.6228298 MHz
NUC1 13C
P0 3.33 usec
P1 10.00 usec
PLW1 66.26200104 W
SFO2 400.1316005 MHz
NUC2 1H
CPDPRG[2 waltz65
PCPD2 90.00 usec
PLW2 16.87899971 W
PLW12 0.20839000 W
PLW13 0.10482000 W

F2 - Processing parameters
SI 32768
SF 100.6127685 MHz
WDW EM
SSB 0
LB 3.00 Hz
GB 0
PC 1.40
Inst.ID: CVL1-AD-NMR-001

Analyzed By:

8-((1-((*trans*)-4-Aminocyclohexyl)-1*H*-1,2,3-triazol-4-yl)methoxy)-*N*-(3-chlorophenyl)quinazolin-2-amine hydrochloride (19a):

Pages S172-S177

A722-15D

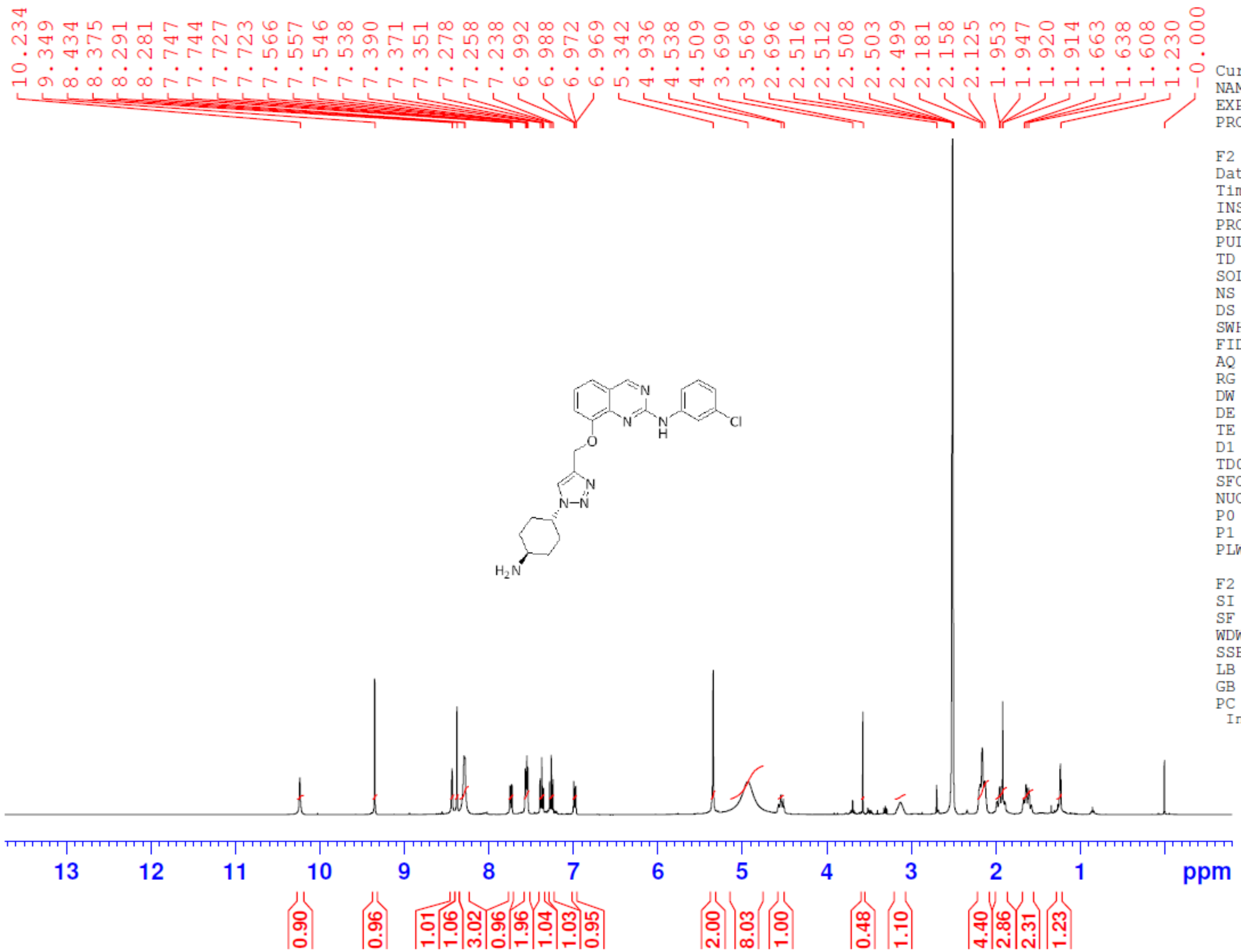

Chemveda Life Sciences

Current Data Parameters
NAME A722-15D
EXPNO 1
PROCNO 1

F2 - Acquisition Parameters
Date_ 20200301
Time 18.21 h
INSTRUM spect
PROBHD Z150453_0004 (
PULPROG zg30
TD 65536
SOLVENT DMSO
NS 16
DS 2
SWH 8012.820 Hz
FIDRES 0.244532 Hz
AQ 4.0894465 sec
RG 147.5
DW 62.400 usec
DE 6.50 usec
TE 300.8 K
D1 1.00000000 sec
TD0 1
SFO1 400.1324708 MHz
NUC1 1H
P0 3.33 usec
P1 10.00 usec
PLW1 16.87899971 W

F2 - Processing parameters
SI 65536
SF 400.1299998 MHz
WDW EM
SSB 0
LB 0.30 Hz
GB 0
PC 1.00
Inst.ID: CVL1-AD-NMR-001

Analyzed By: Harinath

Checked by:

A722-15D

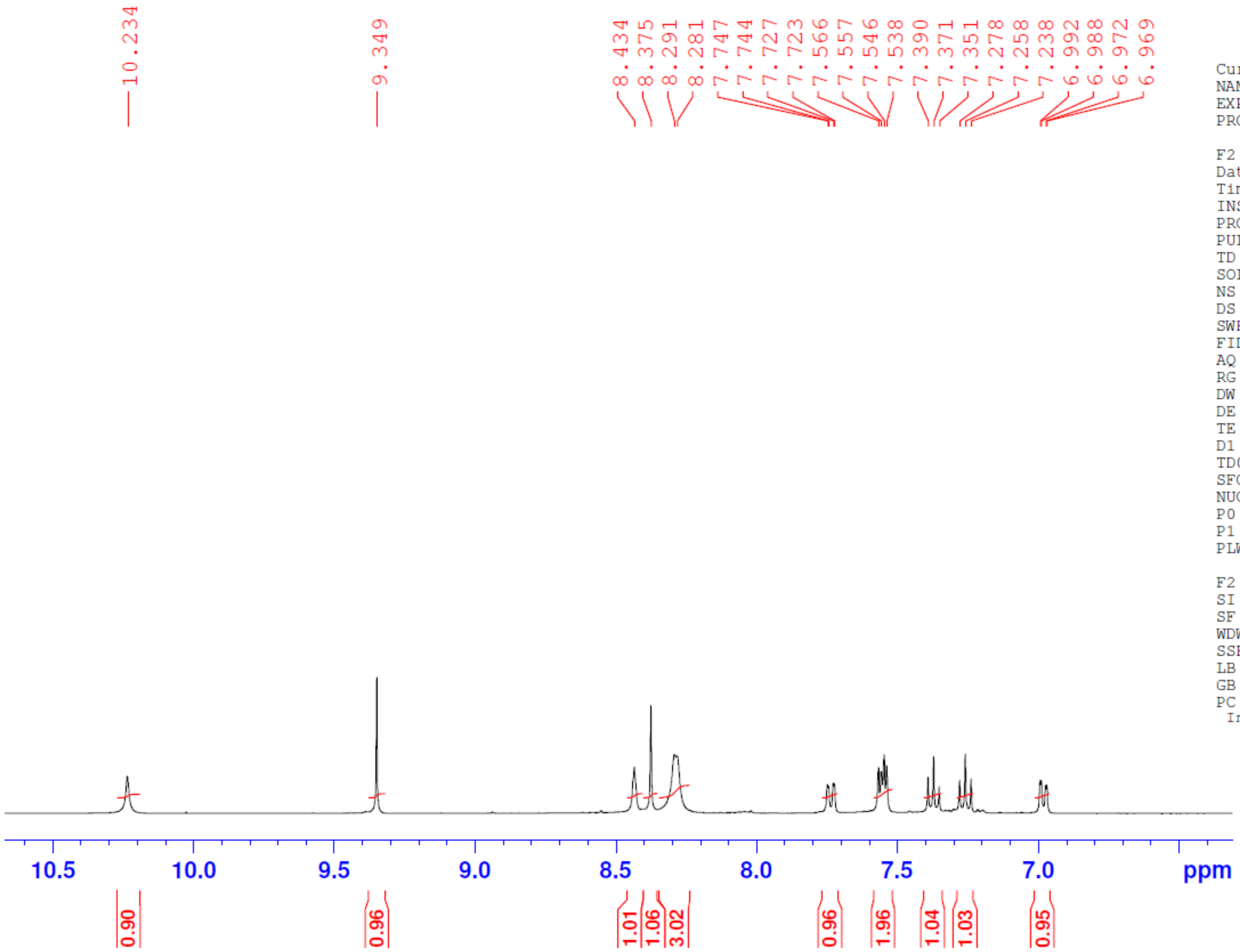

Chemveda Life Sciences

Current Data Parameters
NAME A722-15D
EXPNO 1
PROCNO 1

F2 - Acquisition Parameters
Date_ 20200301
Time 18.21 h
INSTRUM spect
PROBHD Z150453_0004 (
PULPROG zg30
TD 65536
SOLVENT DMSO
NS 16
DS 2
SWH 8012.820 Hz
FIDRES 0.244532 Hz
AQ 4.0894465 sec
RG 147.5
DW 62.400 usec
DE 6.50 usec
TE 300.8 K
D1 1.00000000 sec
TD0 1
SFO1 400.1324708 MHz
NUC1 1H
P0 3.33 usec
P1 10.00 usec
PLW1 16.87899971 W

F2 - Processing parameters
SI 65536
SF 400.1299998 MHz
WDW EM
SSB 0
LB 0.30 Hz
GB 0
PC 1.00
Inst.ID: CVL1-AD-NMR-001

Analyzed By: Harinath

Checked by:

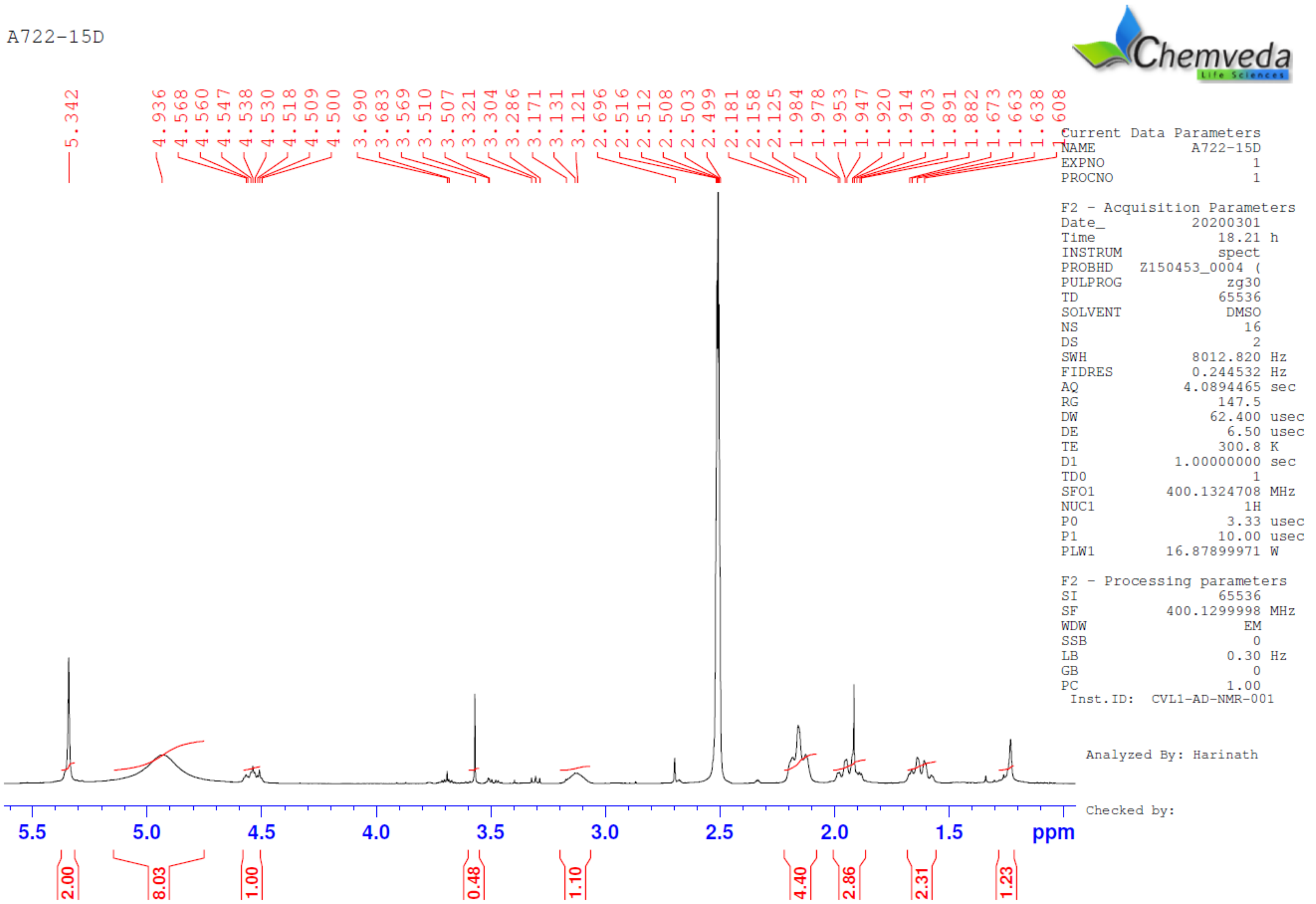
A722-15D
Chemveda
Life Sciences
5.342
4.936
4.568
4.560
4.547
4.538
4.530
4.518
4.509
4.500
3.690
3.683
3.569
3.510
3.507
3.321
3.304
3.286
3.171
3.131
3.121
2.696
2.516
2.512
2.508
2.503
2.499
2.181
2.158
2.125
1.984
1.978
1.953
1.947
1.920
1.914
1.903
1.891
1.882
1.673
1.663
1.638
1.608
Current Data Parameters
NAME A722-15D
EXPNO 1
PROCNO 1
F2 - Acquisition Parameters
Date_ 20200301
Time 18.21 h
INSTRUM spect
PROBHD Z150453_0004 (
PULPROG zg30
TD 65536
SOLVENT DMSO
NS 16
DS 2
SWH 8012.820 Hz
FIDRES 0.244532 Hz
AQ 4.0894465 sec
RG 147.5
DW 62.400 usec
DE 6.50 usec
TE 300.8 K
D1 1.00000000 sec
TD0 1
SFO1 400.1324708 MHz
NUC1 1H
P0 3.33 usec
P1 10.00 usec
PLW1 16.87899971 W
F2 - Processing parameters
SI 65536
SF 400.1299998 MHz
WDW EM
SSB 0
LB 0.30 Hz
GB 0
PC 1.00
Inst.ID: CVL1-AD-NMR-001
Analyzed By: Harinath
Checked by:
5.5
5.0
4.5
4.0
3.5
3.0
2.5
2.0
1.5
ppm
2.00
8.03
1.00
0.48
1.10
4.40
2.86
2.31
1.23

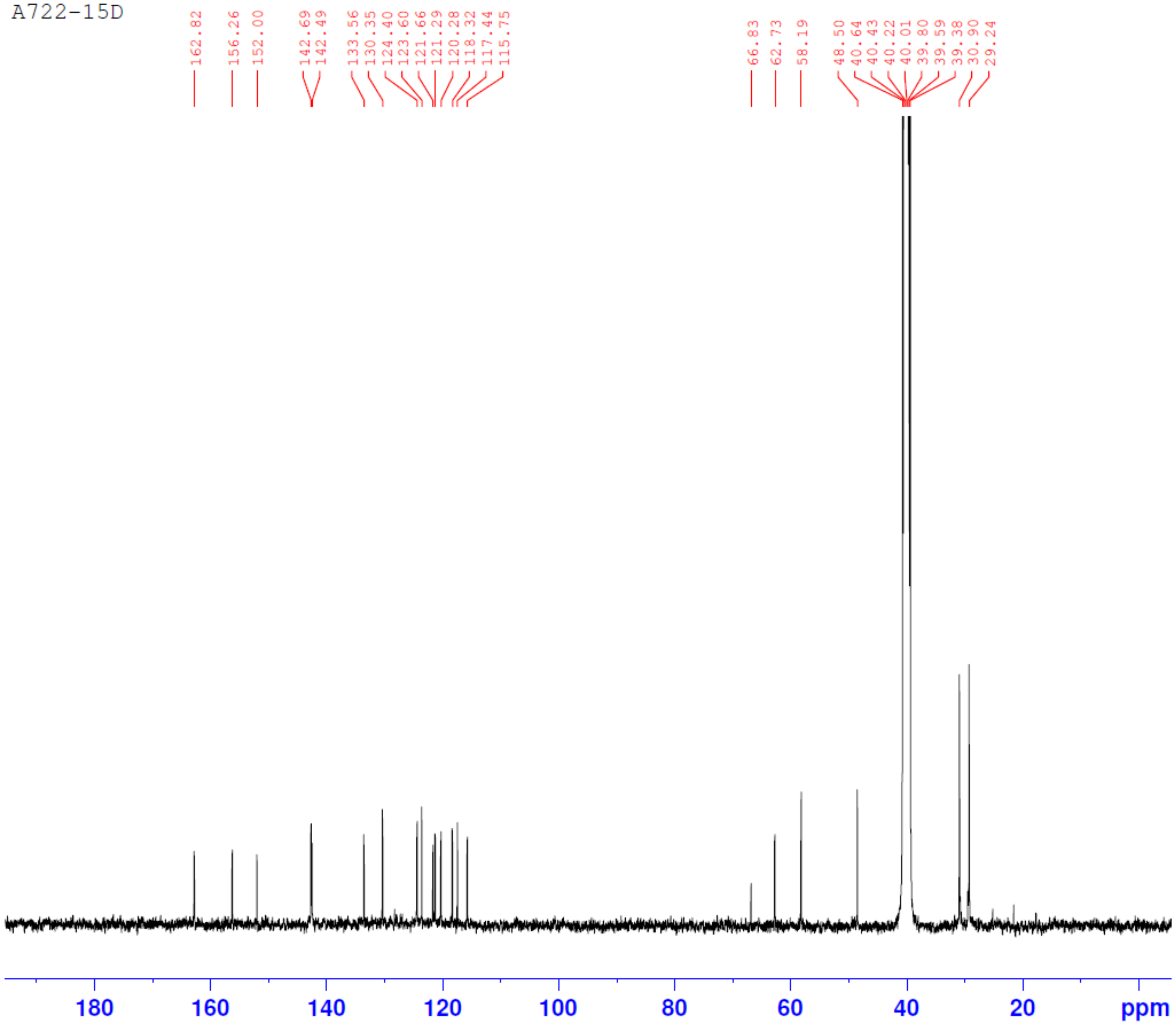


```
Current Data Parameters
NAME              A722-15D
EXPNO                    2
PROCNO                   1

F2 - Acquisition Parameters
Date_             20200301
Time                 22.11 h
INSTRUM              spect
PROBHD    Z150453_0004 (
PULPROG            zgpg30
TD                  65536
SOLVENT              DMSO
NS                   4000
DS                      4
SWH             24038.461 Hz
FIDRES           0.733596 Hz
AQ              1.3631488 sec
RG                 204.77
DW                 20.800 usec
DE                   6.50 usec
TE                  301.7 K
D1             2.00000000 sec
D11            0.03000000 sec
TD0                     1
SFO1          100.6228298 MHz
NUC1                  13C
P0                   3.33 usec
P1                  10.00 usec
PLW1          66.26200104 W
SFO2          400.1316005 MHz
NUC2                   1H
CPDPRG[2          waltz65
PCPD2               90.00 usec
PLW2          16.87899971 W
PLW12          0.20839000 W
PLW13          0.10482000 W

F2 - Processing parameters
SI                  32768
SF            100.6127685 MHz
WDW                    EM
SSB                     0
LB                   3.00 Hz
GB                      0
PC                   1.40
 Inst.ID:  CVL1-AD-NMR-001

 Analyzed By:
```

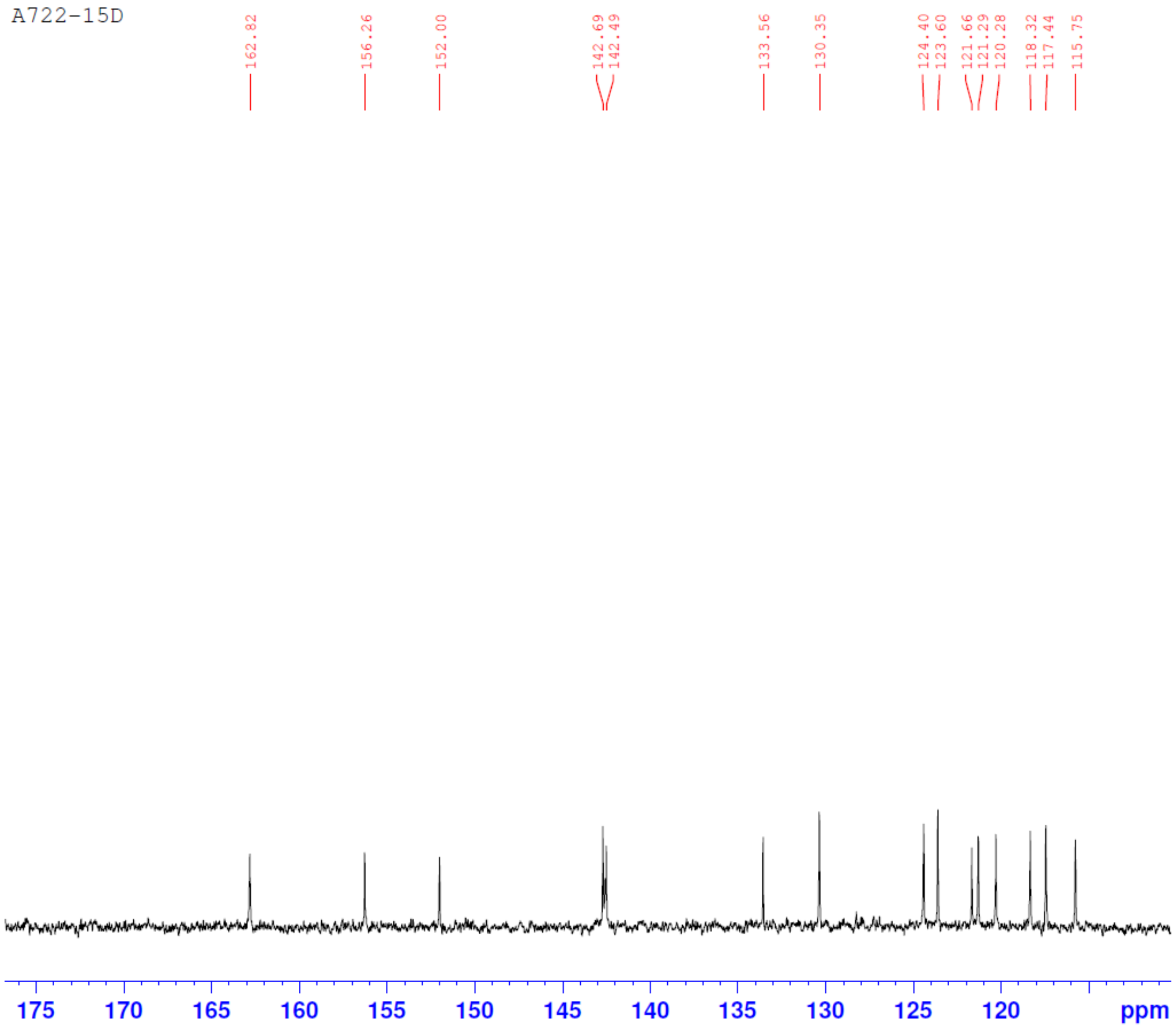

Current Data Parameters
NAME A722-15D
EXPNO 2
PROCNO 1

F2 - Acquisition Parameters

Date_	20200301	
Time	22.11	h
INSTRUM	spect	
PROBHD	Z150453_0004 (	
PULPROG	zgpg30	
TD	65536	
SOLVENT	DMSO	
NS	4000	
DS	4	
SWH	24038.461	Hz
FIDRES	0.733596	Hz
AQ	1.3631488	sec
RG	204.77	
DW	20.800	usec
DE	6.50	usec
TE	301.7	K
D1	2.00000000	sec
D11	0.03000000	sec
TD0	1	
SFO1	100.6228298	MHz
NUC1	13C	
P0	3.33	usec
P1	10.00	usec
PLW1	66.26200104	W
SFO2	400.1316005	MHz
NUC2	1H	
CPDPRG[2	waltz65	
PCPD2	90.00	usec
PLW2	16.87899971	W
PLW12	0.20839000	W
PLW13	0.10482000	W

F2 - Processing parameters

SI	32768	
SF	100.6127685	MHz
WDW	EM	
SSB	0	
LB	3.00	Hz
GB	0	
PC	1.40	

Inst.ID: CVL1-AD-NMR-001

Analyzed By:

8-((1-((*trans*)-4-Aminocyclohexyl)-1*H*-1,2,3-triazol-4-yl)methoxy)-*N*-(3,5-dichlorophenyl)quinazolin-2-amine hydrochloride (19b):

Pages S178-S183

A722-016D

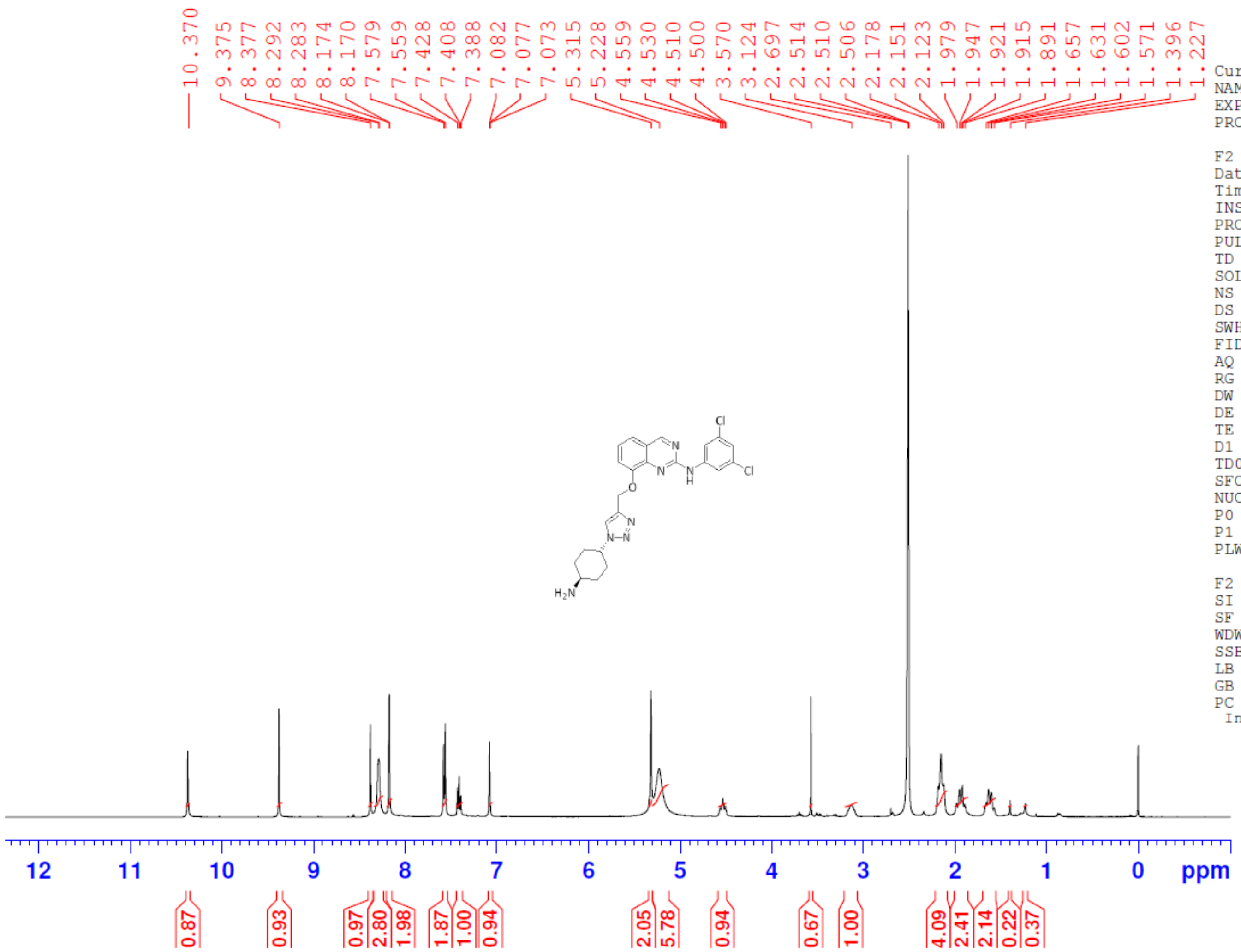

Chemveda Life Sciences

Current Data Parameters
NAME A722-016D
EXPNO 1
PROCNO 1

F2 - Acquisition Parameters
Date_ 20200229
Time 3.14 h
INSTRUM spect
PROBHD Z150453_0004 (
PULPROG zg30
TD 65536
SOLVENT DMSO
NS 16
DS 2
SWH 8012.820 Hz
FIDRES 0.244532 Hz
AQ 4.0894465 sec
RG 101.26
DW 62.400 usec
DE 6.50 usec
TE -10.7 K
D1 1.00000000 sec
TD0 1
SFO1 400.1324708 MHz
NUC1 1H
P0 3.33 usec
P1 10.00 usec
PLW1 16.87899971 W

F2 - Processing parameters
SI 65536
SF 400.1299988 MHz
WDW EM
SSB 0
LB 0.30 Hz
GB 0
PC 1.00
Inst.ID: CVL1-AD-NMR-001

Analyzed By: Harinath

Checked by:

A722-016D

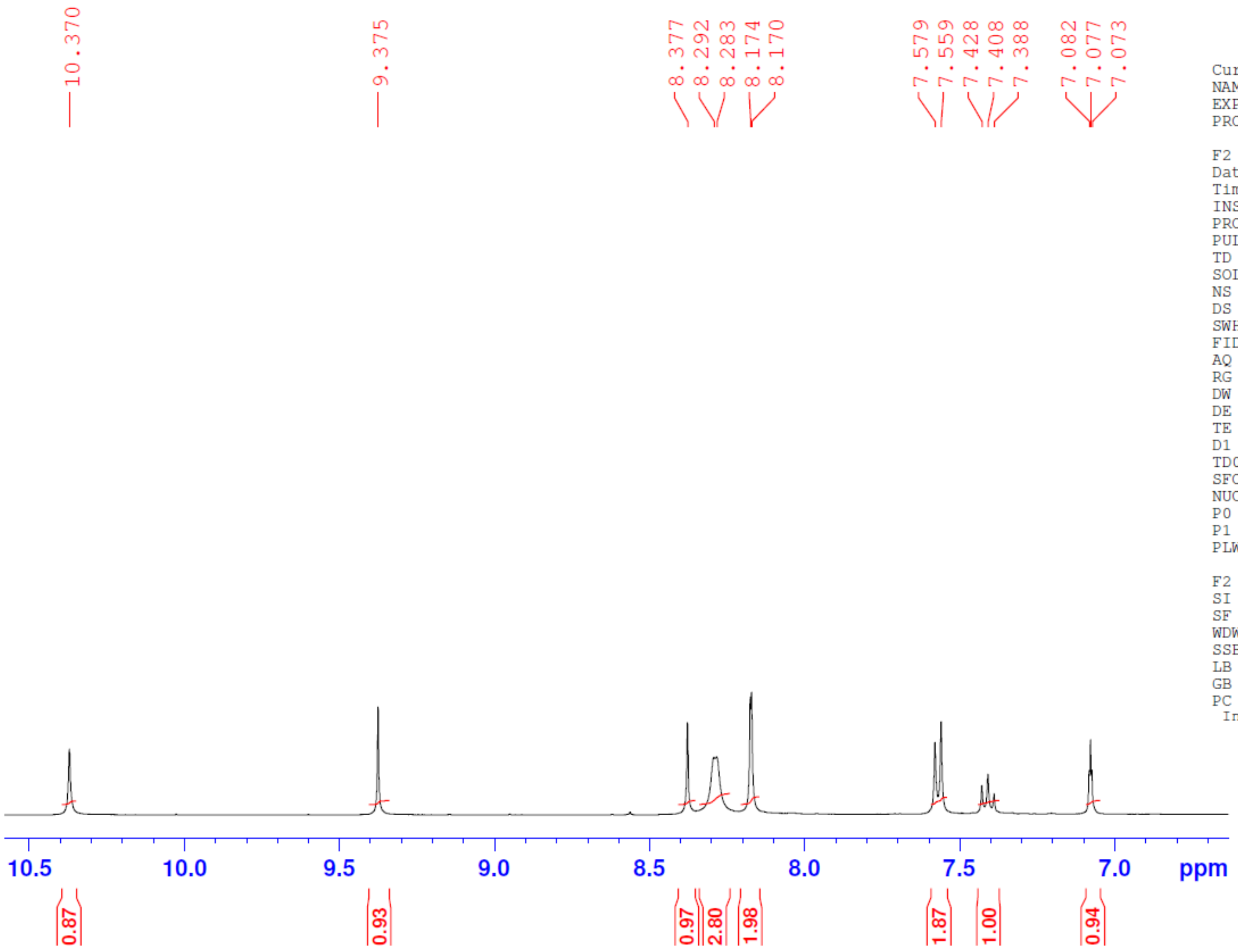


```
Current Data Parameters
NAME              A722-016D
EXPNO                     1
PROCNO                    1

F2 - Acquisition Parameters
Date_             20200229
Time                  3.14 h
INSTRUM              spect
PROBHD   Z150453_0004 (
PULPROG               zg30
TD                   65536
SOLVENT               DMSO
NS                      16
DS                       2
SWH               8012.820 Hz
FIDRES            0.244532 Hz
AQ               4.0894465 sec
RG                  101.26
DW                  62.400 usec
DE                    6.50 usec
TE                   -10.7 K
D1              1.00000000 sec
TD0                      1
SFO1           400.1324708 MHz
NUC1                    1H
P0                    3.33 usec
P1                   10.00 usec
PLW1           16.87899971 W

F2 - Processing parameters
SI                   65536
SF             400.1299988 MHz
WDW                     EM
SSB                      0
LB                    0.30 Hz
GB                       0
PC                    1.00
 Inst.ID:  CVL1-AD-NMR-001
```

Analyzed By: Harinath

Checked by:

A722-016D

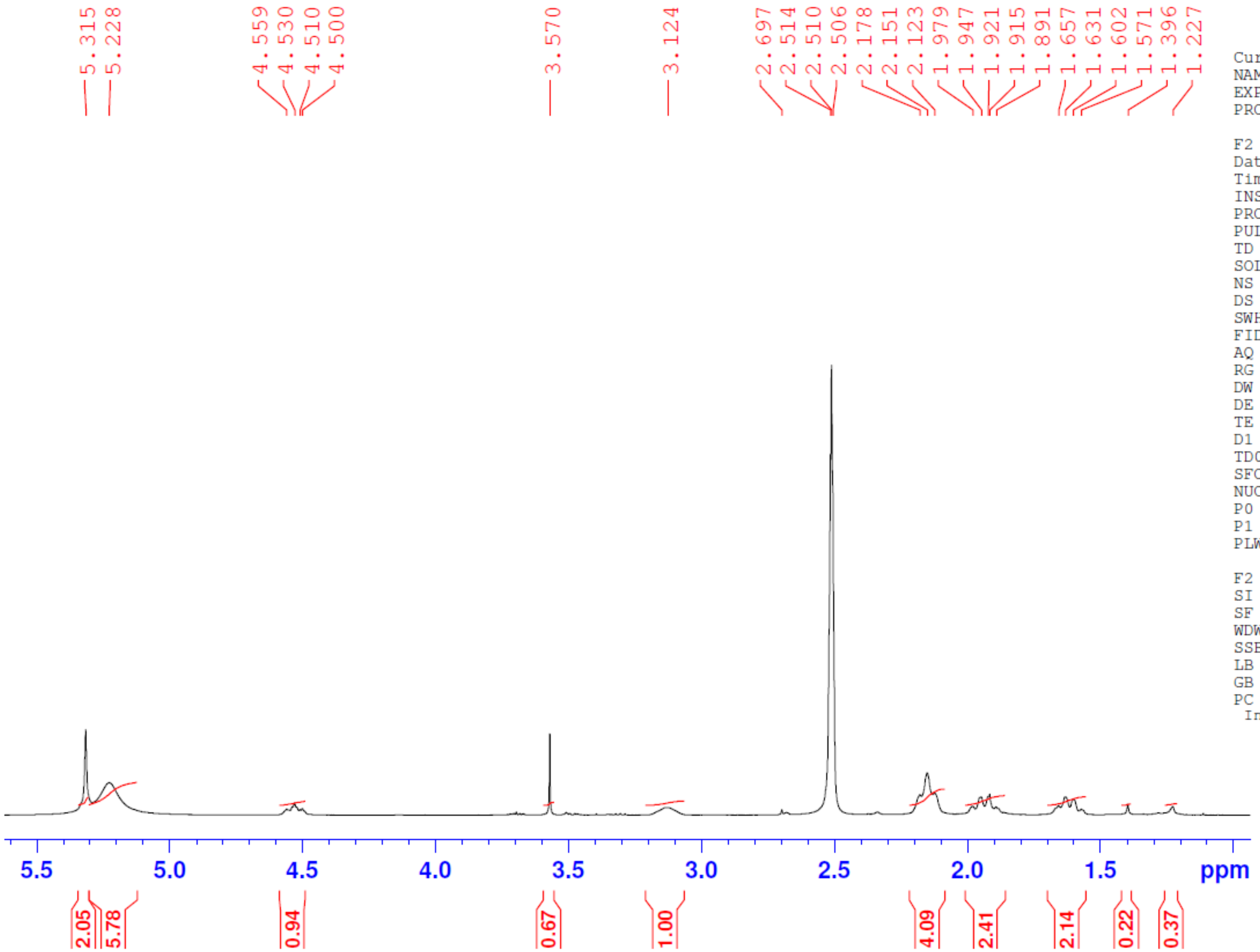

Current Data Parameters
NAME A722-016D
EXPNO 1
PROCNO 1

F2 - Acquisition Parameters
Date_ 20200229
Time 3.14 h
INSTRUM spect
PROBHD Z150453_0004 (
PULPROG zg30
TD 65536
SOLVENT DMSO
NS 16
DS 2
SWH 8012.820 Hz
FIDRES 0.244532 Hz
AQ 4.0894465 sec
RG 101.26
DW 62.400 usec
DE 6.50 usec
TE -10.7 K
D1 1.00000000 sec
TD0 1
SFO1 400.1324708 MHz
NUC1 1H
P0 3.33 usec
P1 10.00 usec
PLW1 16.87899971 W

F2 - Processing parameters
SI 65536
SF 400.1299988 MHz
WDW EM
SSB 0
LB 0.30 Hz
GB 0
PC 1.00
Inst.ID: CVL1-AD-NMR-001

Analyzed By: Harinath

Checked by:

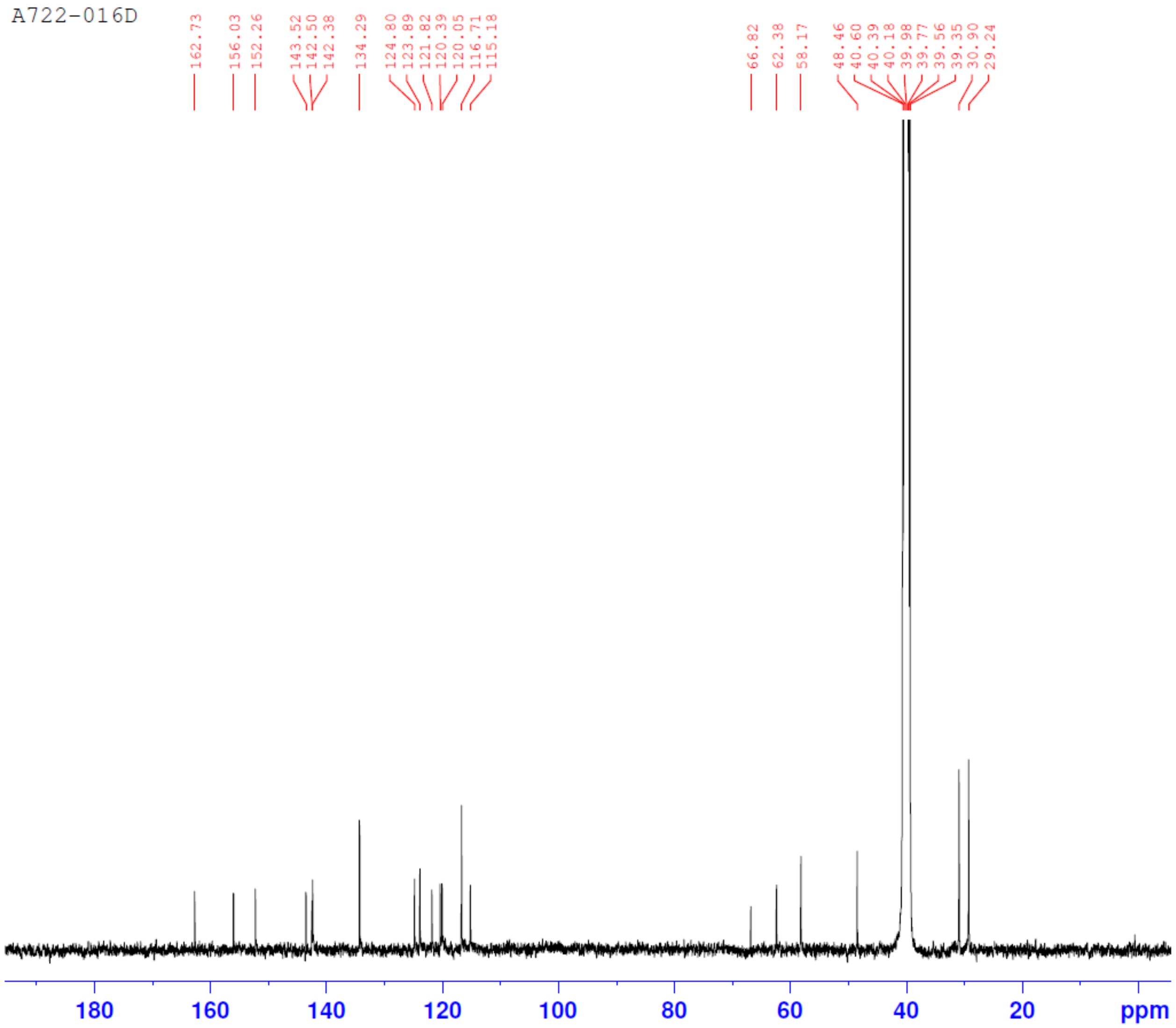

Current Data Parameters
NAME A722-016D
EXPNO 2
PROCNO 1

F2 - Acquisition Parameters
Date_ 20200229
Time 5.10 h
INSTRUM spect
PROBHD Z150453_0004 (
PULPROG zgpg30
TD 65536
SOLVENT DMSO
NS 2000
DS 4
SWH 24038.461 Hz
FIDRES 0.733596 Hz
AQ 1.3631488 sec
RG 204.77
DW 20.800 usec
DE 6.50 usec
TE -10.7 K
D1 2.00000000 sec
D11 0.03000000 sec
TD0 1
SFO1 100.6228298 MHz
NUC1 13C
P0 3.33 usec
P1 10.00 usec
PLW1 66.26200104 W
SFO2 400.1316005 MHz
NUC2 1H
CPDPRG[2 waltz65
PCPD2 90.00 usec
PLW2 16.87899971 W
PLW12 0.20839000 W
PLW13 0.10482000 W

F2 - Processing parameters
SI 32768
SF 100.6127685 MHz
WDW EM
SSB 0
LB 3.00 Hz
GB 0
PC 1.40
Inst.ID: CVL1-AD-NMR-001

Analyzed By:

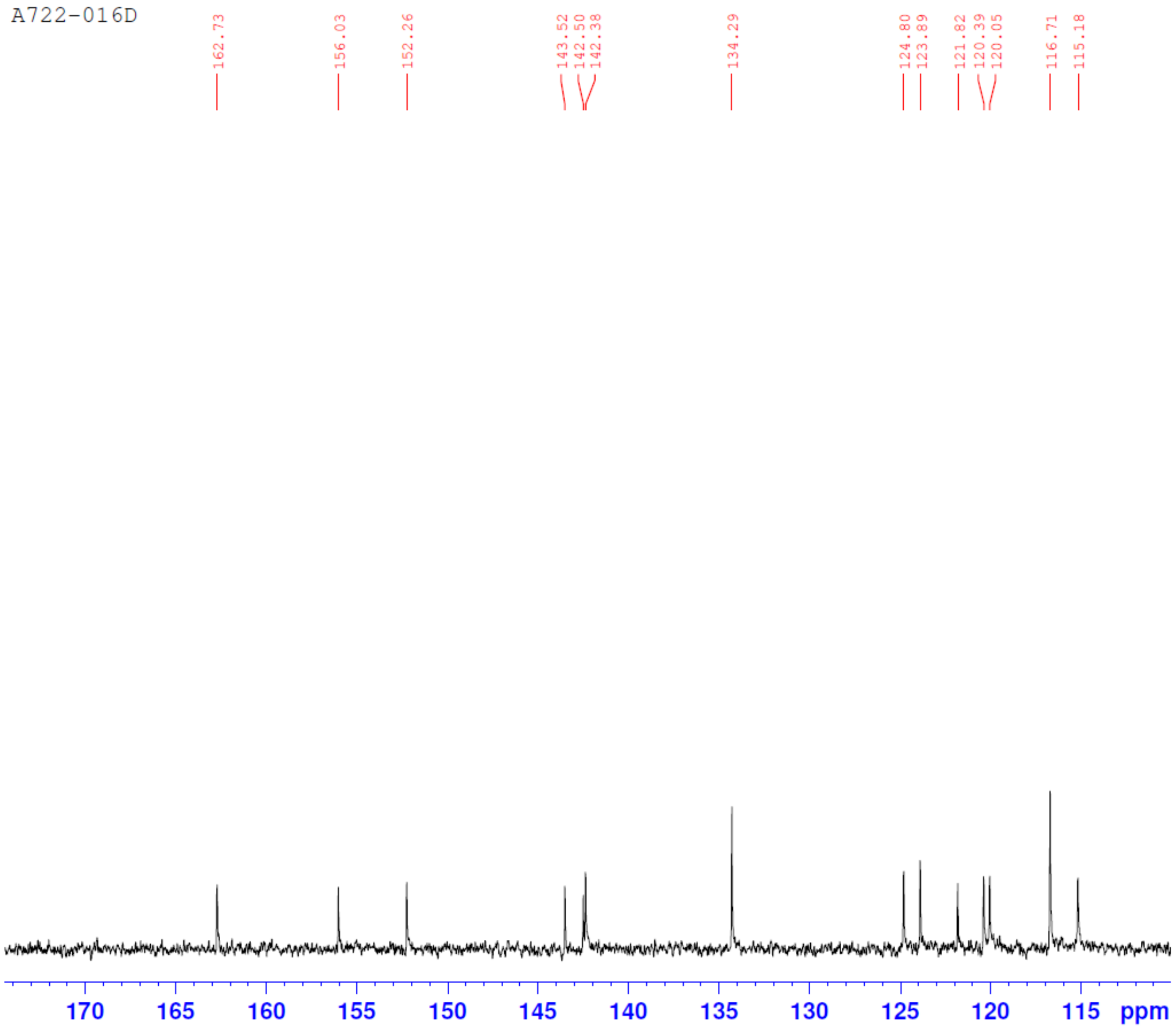

Current Data Parameters
NAME A722-016D
EXPNO 2
PROCNO 1

F2 - Acquisition Parameters
Date_ 20200229
Time 5.10 h
INSTRUM spect
PROBHD Z150453_0004 (
PULPROG zgpg30
TD 65536
SOLVENT DMSO
NS 2000
DS 4
SWH 24038.461 Hz
FIDRES 0.733596 Hz
AQ 1.3631488 sec
RG 204.77
DW 20.800 usec
DE 6.50 usec
TE -10.7 K
D1 2.00000000 sec
D11 0.03000000 sec
TD0 1
SFO1 100.6228298 MHz
NUC1 13C
P0 3.33 usec
P1 10.00 usec
PLW1 66.26200104 W
SFO2 400.1316005 MHz
NUC2 1H
CPDPRG[2 waltz65
PCPD2 90.00 usec
PLW2 16.87899971 W
PLW12 0.20839000 W
PLW13 0.10482000 W

F2 - Processing parameters
SI 32768
SF 100.6127685 MHz
WDW EM
SSB 0
LB 3.00 Hz
GB 0
PC 1.40
Inst.ID: CVL1-AD-NMR-001

Analyzed By:

Centre régional de mesures physiques de l'Ouest (CRMPO) - RAPPORT D'ANALYSE

Analysis Info

Analysis Name	D:\Data\CRMPO\ESI_12888_MS_01.d	Acquisition Date	1/10/2022 12:27:30 PM
Method	CRMPO_tune_low.m	Operator	Fabian LAMBERT
Sample Name	DB Ph	Instrument	maXis
Comment	P. MOSSET DB Ph Solvant : CH3OH/CH2Cl2 (90/10)		

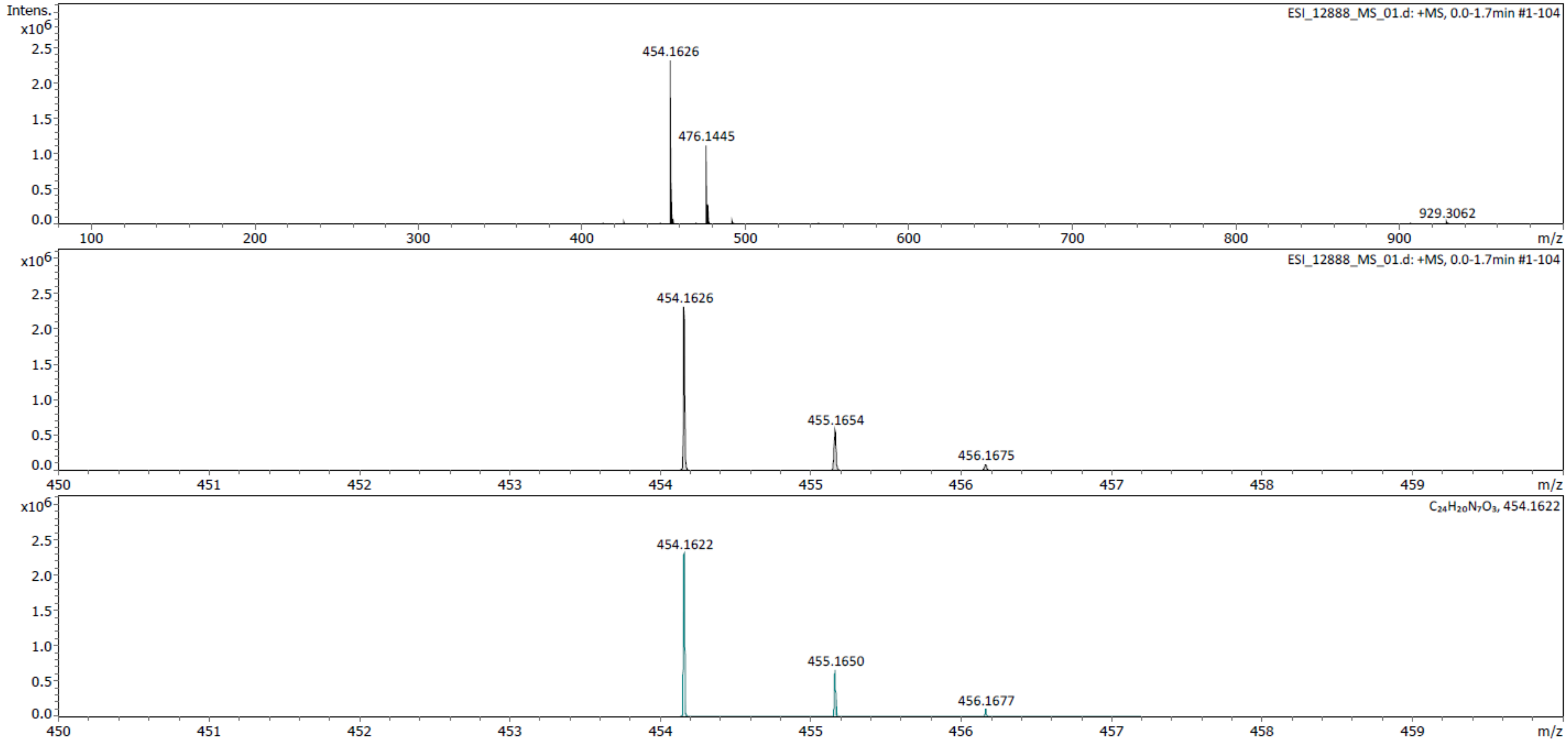

Centre régional de mesures physiques de l'Ouest (CRMPO) - RAPPORT D'ANALYSE

Analysis Info

Analysis Name	D:\Data\CRMPO\ESI_12889_MS_01.d	Acquisition Date	1/10/2022 2:36:53 PM
Method	CRMPO_tune_low.m	Operator	Fabian LAMBERT
Sample Name	PM 8173	Instrument	maXis
Comment	P. MOSSET PM 8173 Solvant : CH3OH/CH2Cl2 (90/10)		

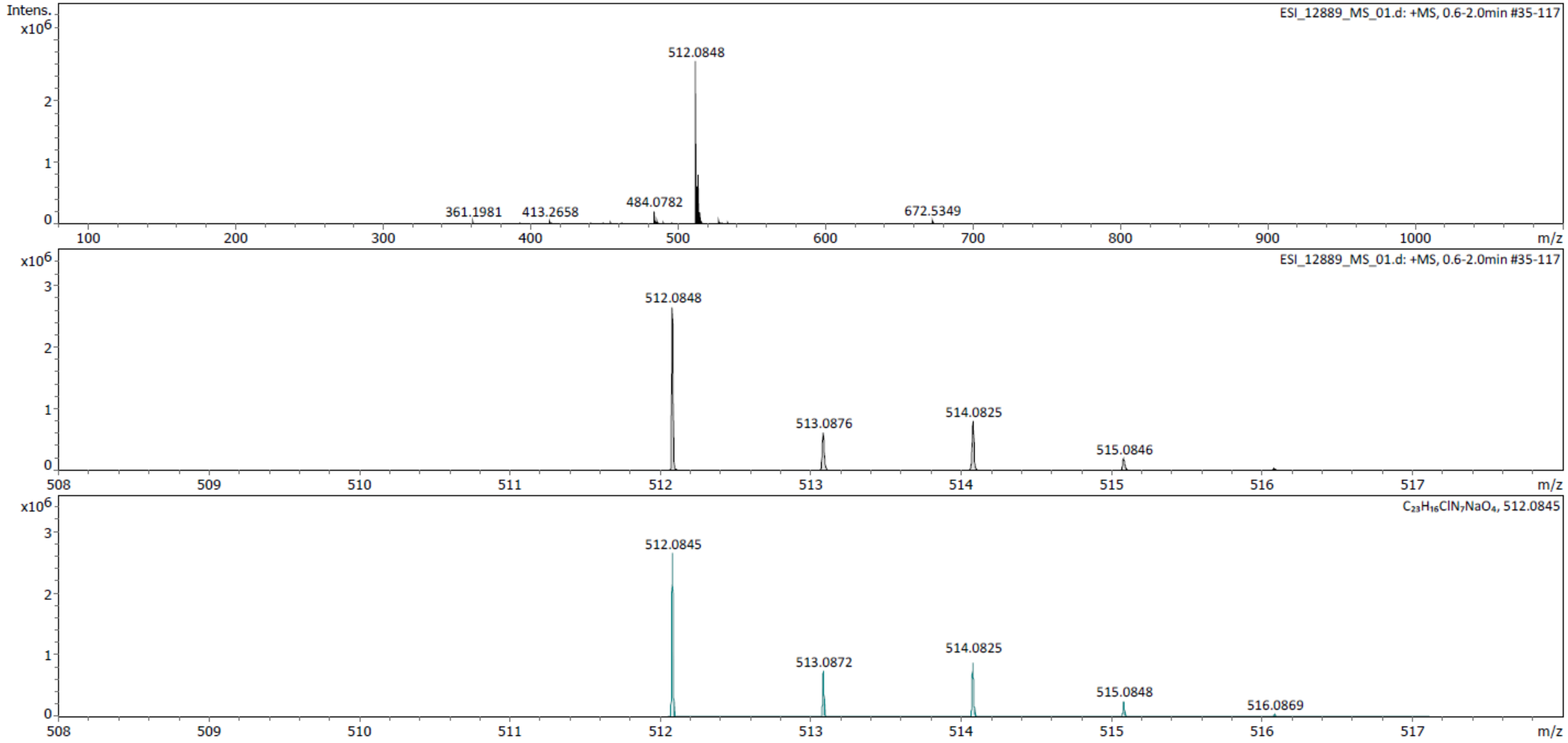

Centre régional de mesures physiques de l'Ouest (CRMPO) - RAPPORT D'ANALYSE

**Analysis Info**		Acquisition Date	1/10/2022 9:50:36 AM
Analysis Name	D:\Data\CRMPO\ESI_12885_MS_01.d		
Method	CRMPO_tune_low.m	Operator	Fabian LAMBERT
Sample Name	DB 33	Instrument	maXis
Comment	P. MOSSET DB 33 Solvant : CH3OH/CH2Cl2 (90/10)		

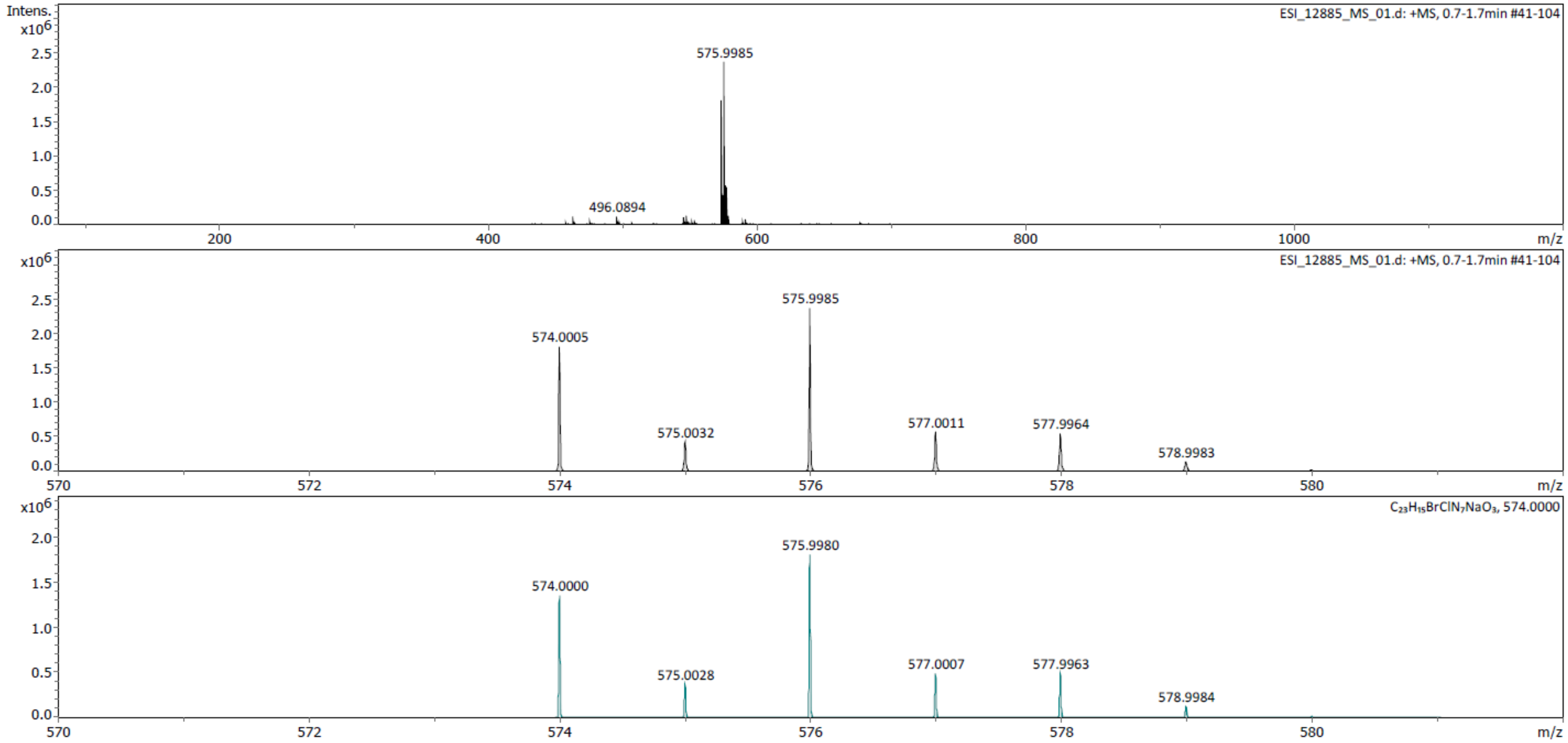

Centre régional de mesures physiques de l'Ouest (CRMPO) - RAPPORT D'ANALYSE

Analysis Info

Analysis Name	D:\Data\CRMPO\ESI_12880_MS_01.d	Acquisition Date	1/6/2022 11:57:58 AM
Method	CRMPO_tune_low.m	Operator	Fabian LAMBERT
Sample Name	PM 9019	Instrument	maXis
Comment	P. MOSSET PM 9019 Solvant : CH3OH/CH2Cl2 (90/10)		

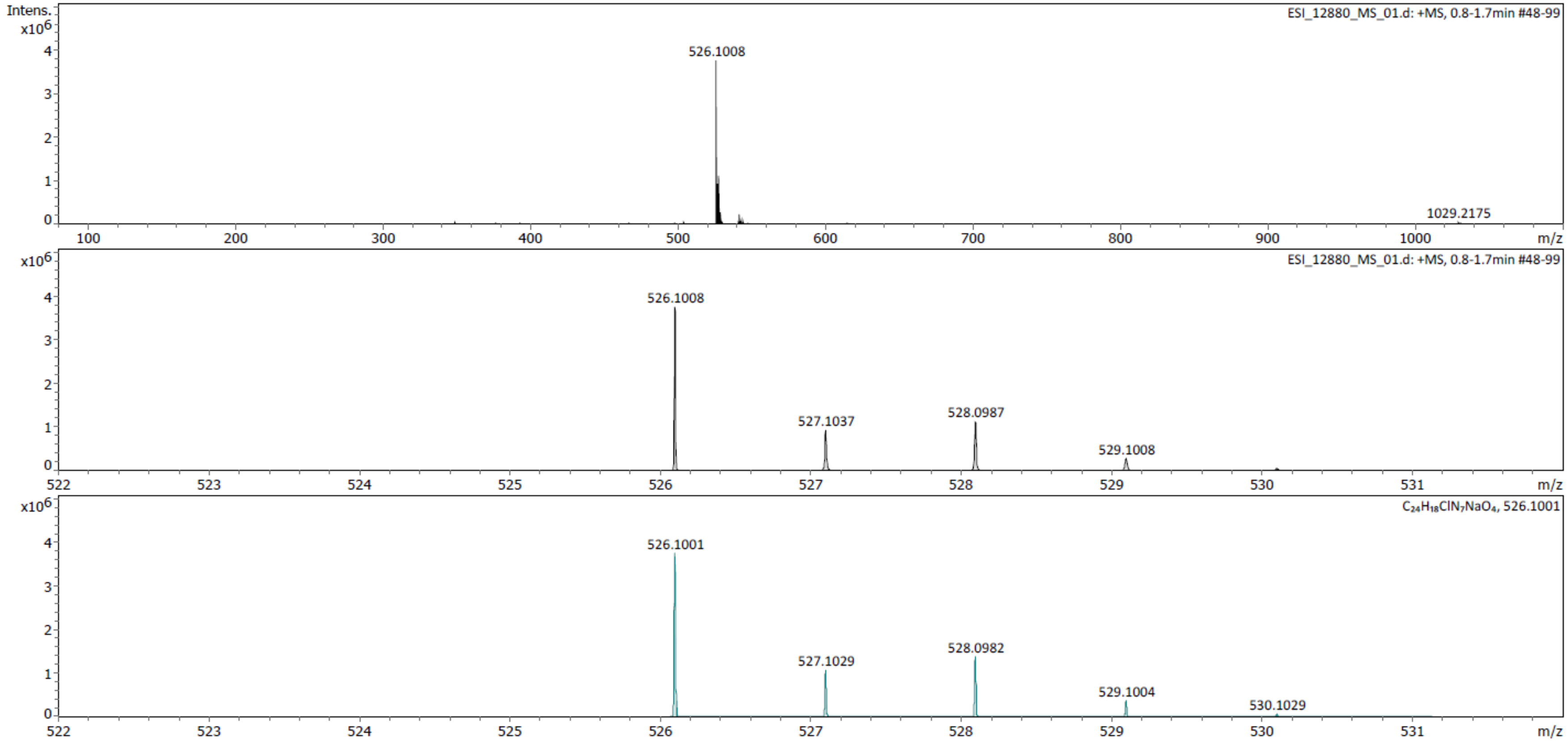

Centre régional de mesures physiques de l'Ouest (CRMPO) - RAPPORT D'ANALYSE

Analysis Info

Analysis Name	D:\Data\CRMPO\ESI_12886_MS_01.d	Acquisition Date	1/10/2022 10:25:20 AM
Method	CRMPO_tune_low.m	Operator	Fabian LAMBERT
Sample Name	DB 34	Instrument	maXis
Comment	P. MOSSET DB 34 Solvant : CH3OH/CH2Cl2 (90/10)		

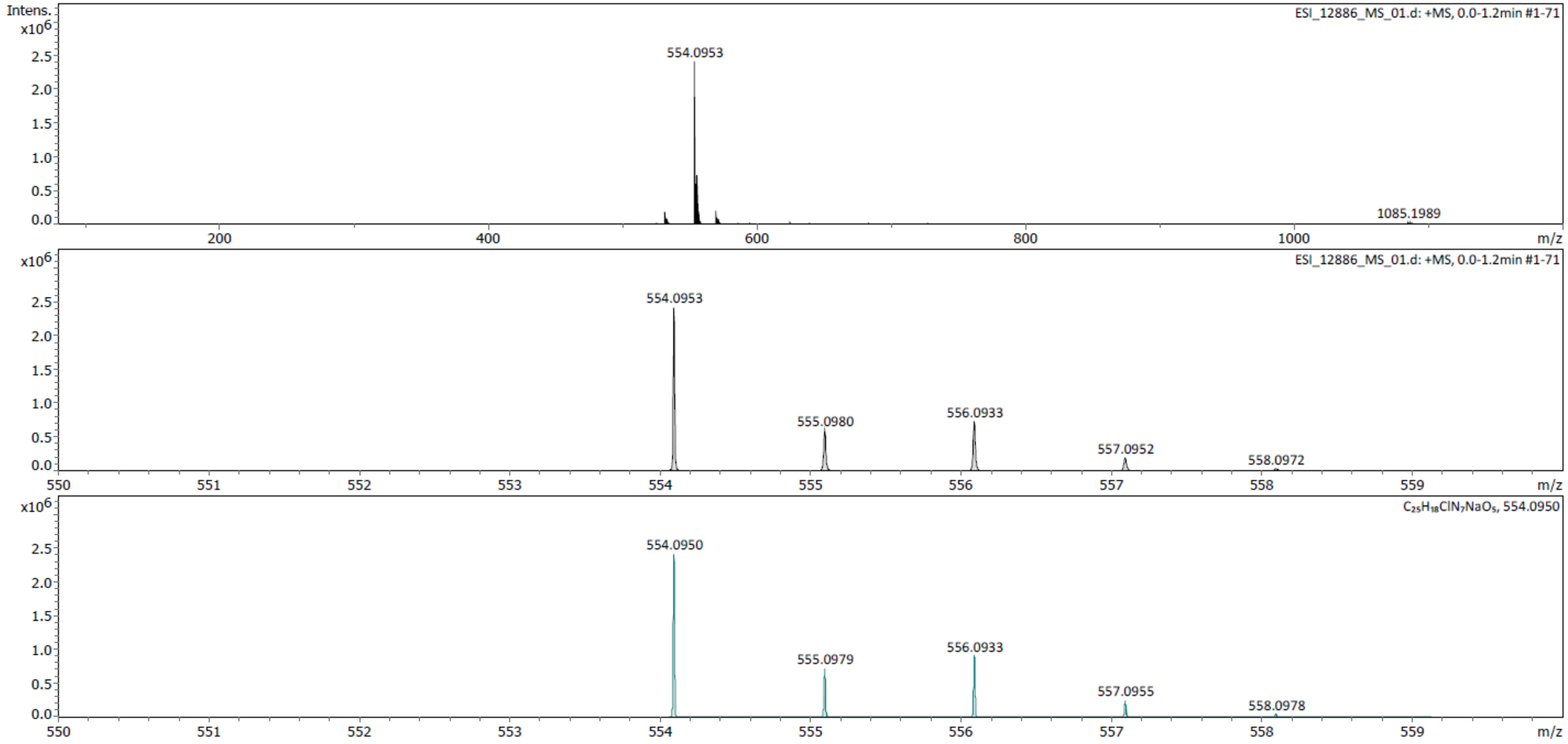

Centre régional de mesures physiques de l'Ouest (CRMPO) - RAPPORT D'ANALYSE

**Analysis Info**		Acquisition Date	1/10/2022 11:11:20 AM
Analysis Name	D:\Data\CRMPO\ESI_12887_MS_02.d		
Method	CRMPO_tune_low_neg.m	Operator	Fabian LAMBERT
Sample Name	DB 35	Instrument	maXis
Comment	P. MOSSET DB 35 Solvant : CH3OH/CH2Cl2 (90/10)		

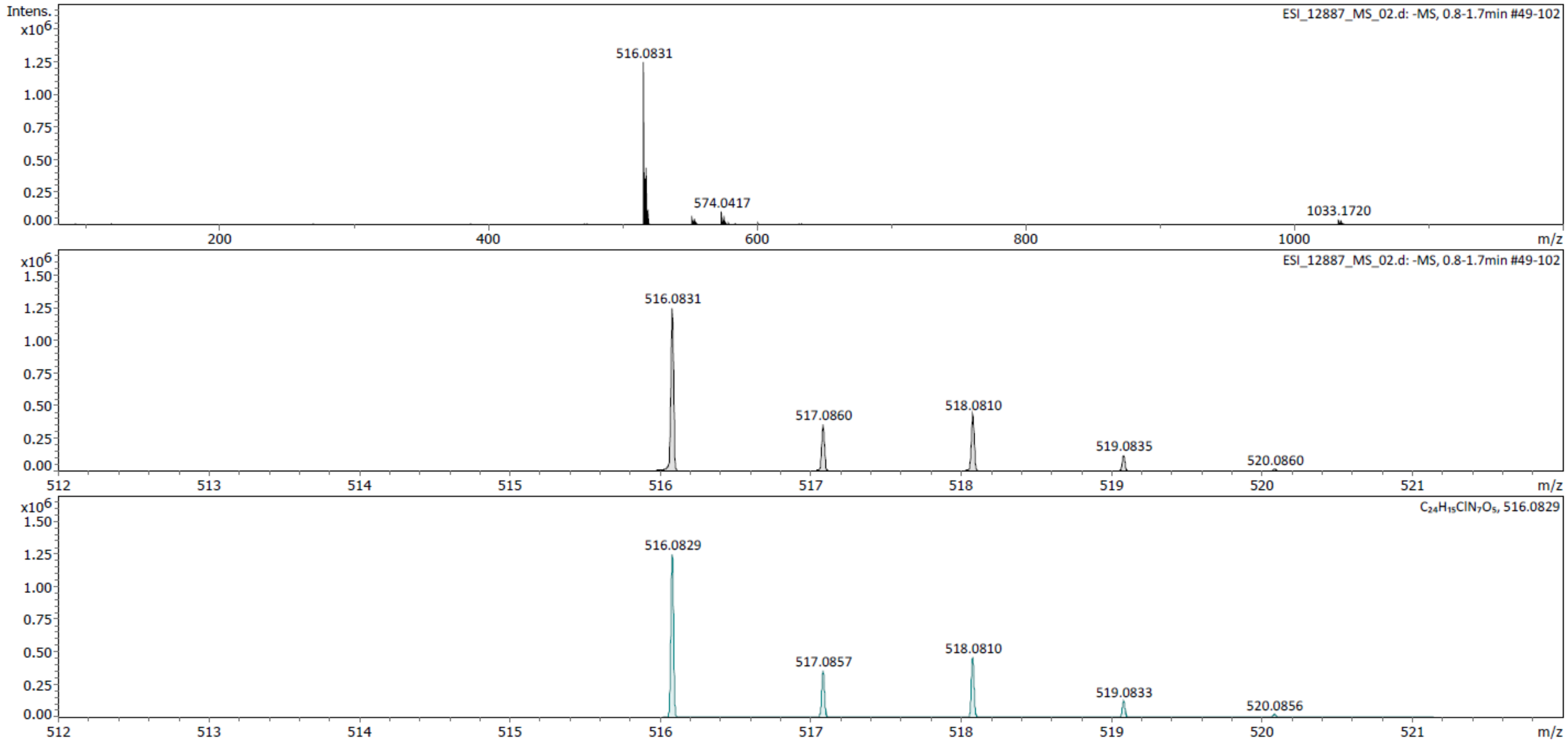

Centre régional de mesures physiques de l'Ouest (CRMPO) - RAPPORT D'ANALYSE

Analysis Info

Analysis Name	D:\Data\CRMPO\ESI_12881_MS_01.d	Acquisition Date	1/6/2022 12:26:55 PM
Method	CRMPO_tune_low.m	Operator	Fabian LAMBERT
Sample Name	DB 25	Instrument	maXis
Comment	P. MOSSET DB 25 Solvant : CH3OH/CH2Cl2 (90/10)		

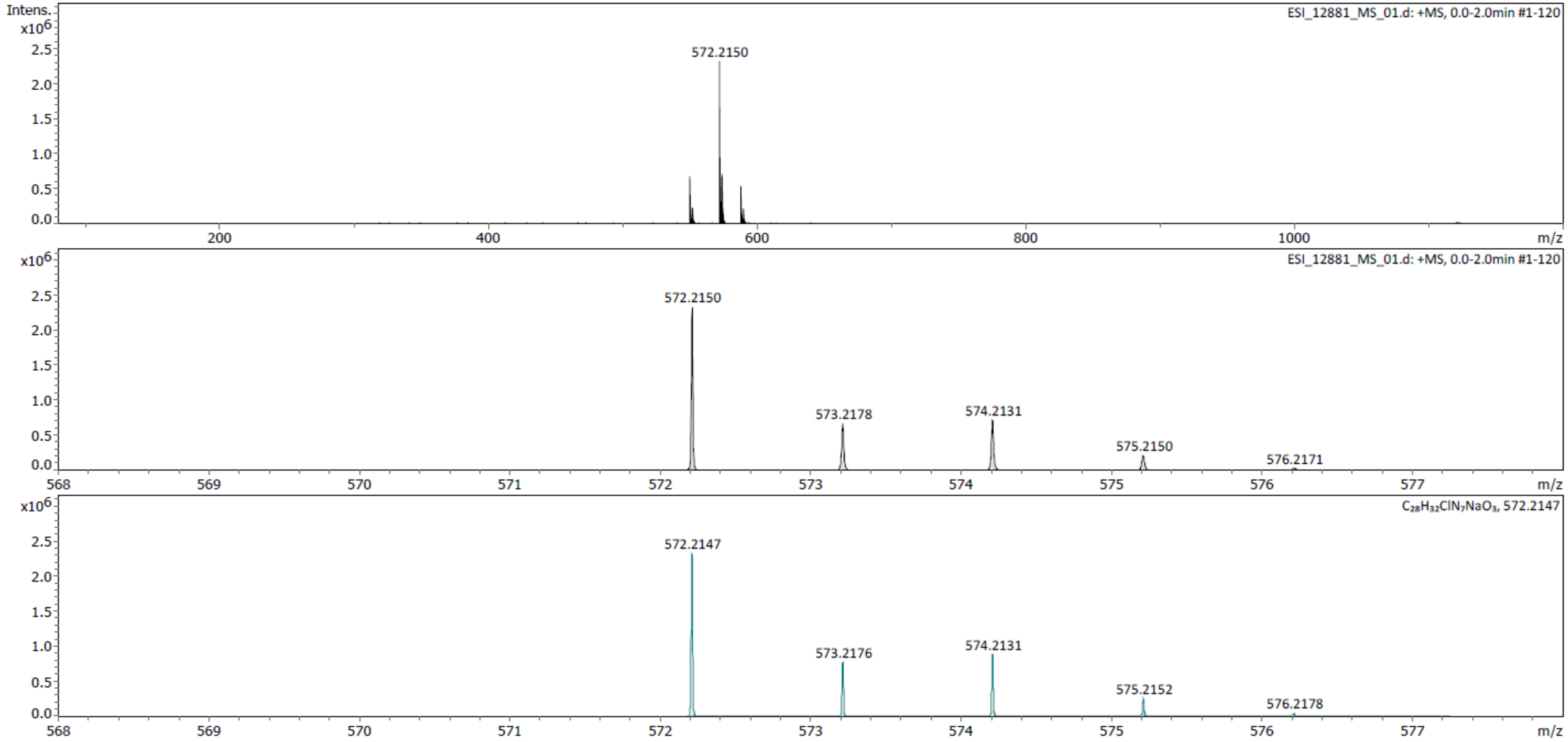

Centre régional de mesures physiques de l'Ouest (CRMPO) - RAPPORT D'ANALYSE

Analysis Info

Analysis Name	D:\Data\CRMPO\ESI_12882_MS_01.d	Acquisition Date	1/7/2022 3:03:32 PM
Method	CRMPO_tune_low.m	Operator	Fabian LAMBERT
Sample Name	DB 26	Instrument	maXis
Comment	P. MOSSET DB 26 Solvant : CH3OH/CH2Cl2 (90/10)		

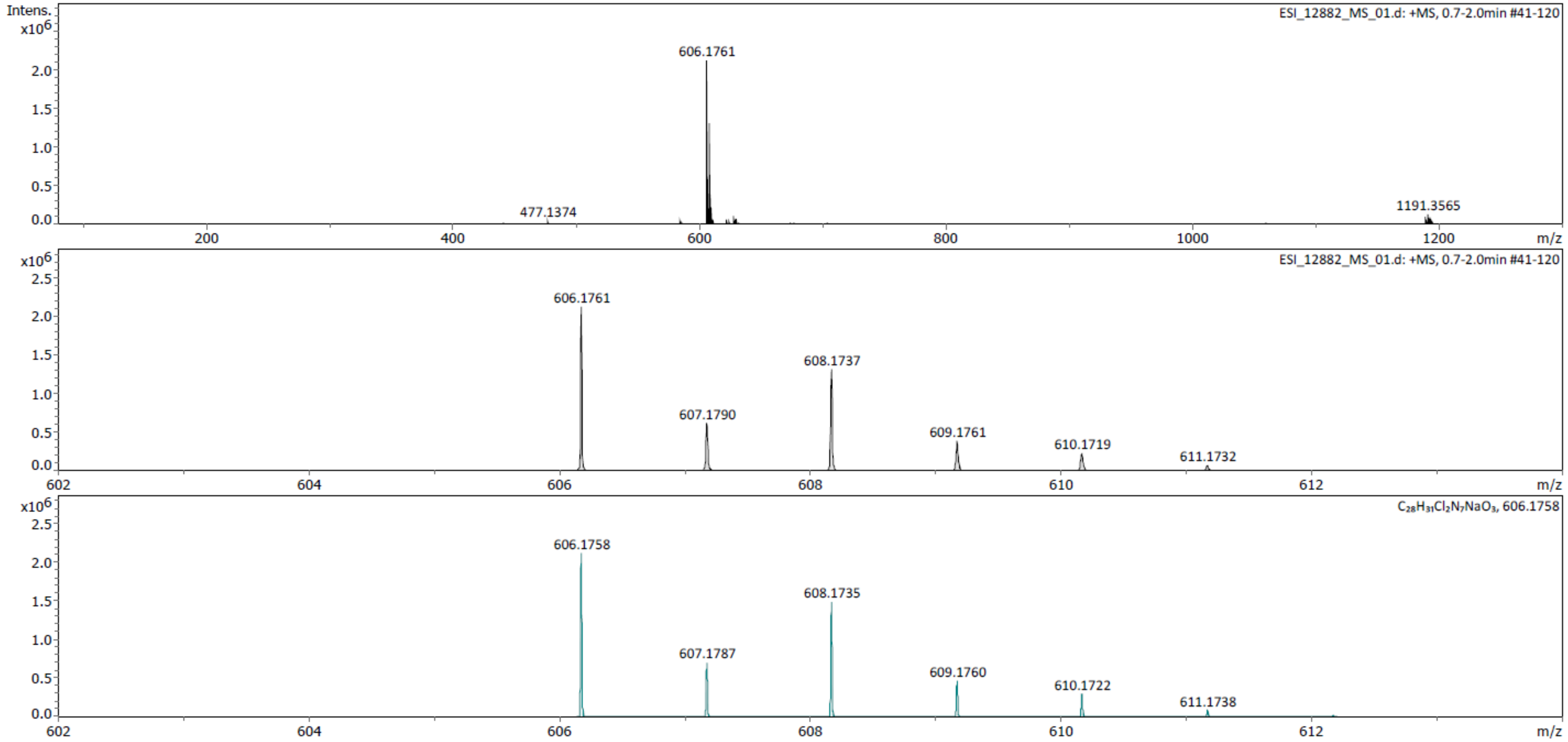

Centre régional de mesures physiques de l'Ouest (CRMPO) - RAPPORT D'ANALYSE

Analysis Info

Analysis Name	D:\Data\CRMPO\ESI_12883_MS_01.d	Acquisition Date	1/7/2022 3:43:08 PM
Method	CRMPO_tune_low.m	Operator	Fabian LAMBERT
Sample Name	DB 27	Instrument	maXis
Comment	P. MOSSET DB 27 Solvant : CH3OH/CH2Cl2 (90/10)		

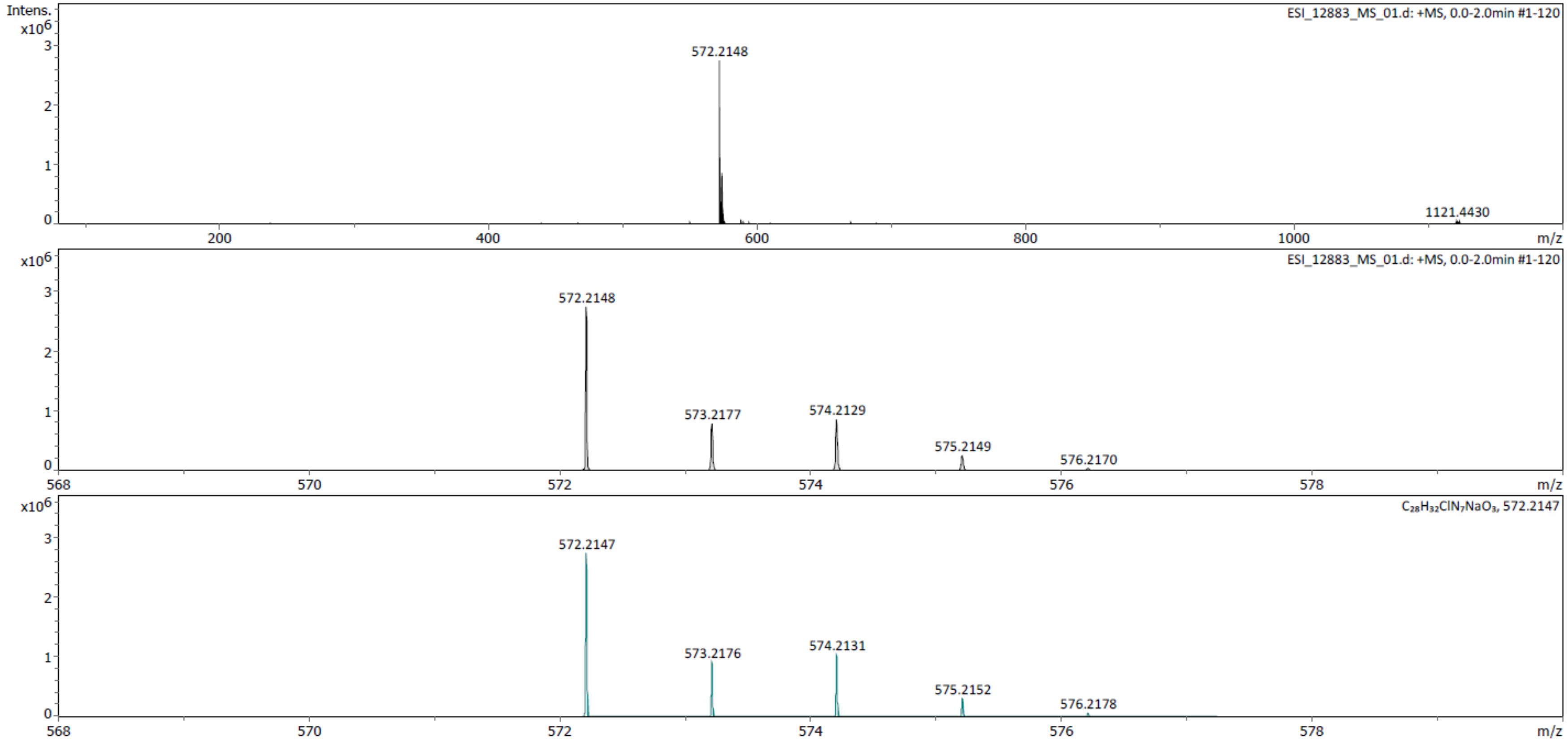

Centre régional de mesures physiques de l'Ouest (CRMPO) - RAPPORT D'ANALYSE

**Analysis Info**		Acquisition Date	1/7/2022 4:05:07 PM
Analysis Name	D:\Data\CRMPO\ESI_12884_MS_01.d		
Method	CRMPO_tune_low.m	Operator	Fabian LAMBERT
Sample Name	DB 28	Instrument	maXis
Comment	P. MOSSET DB 28 Solvant : CH3OH/CH2Cl2 (90/10)		

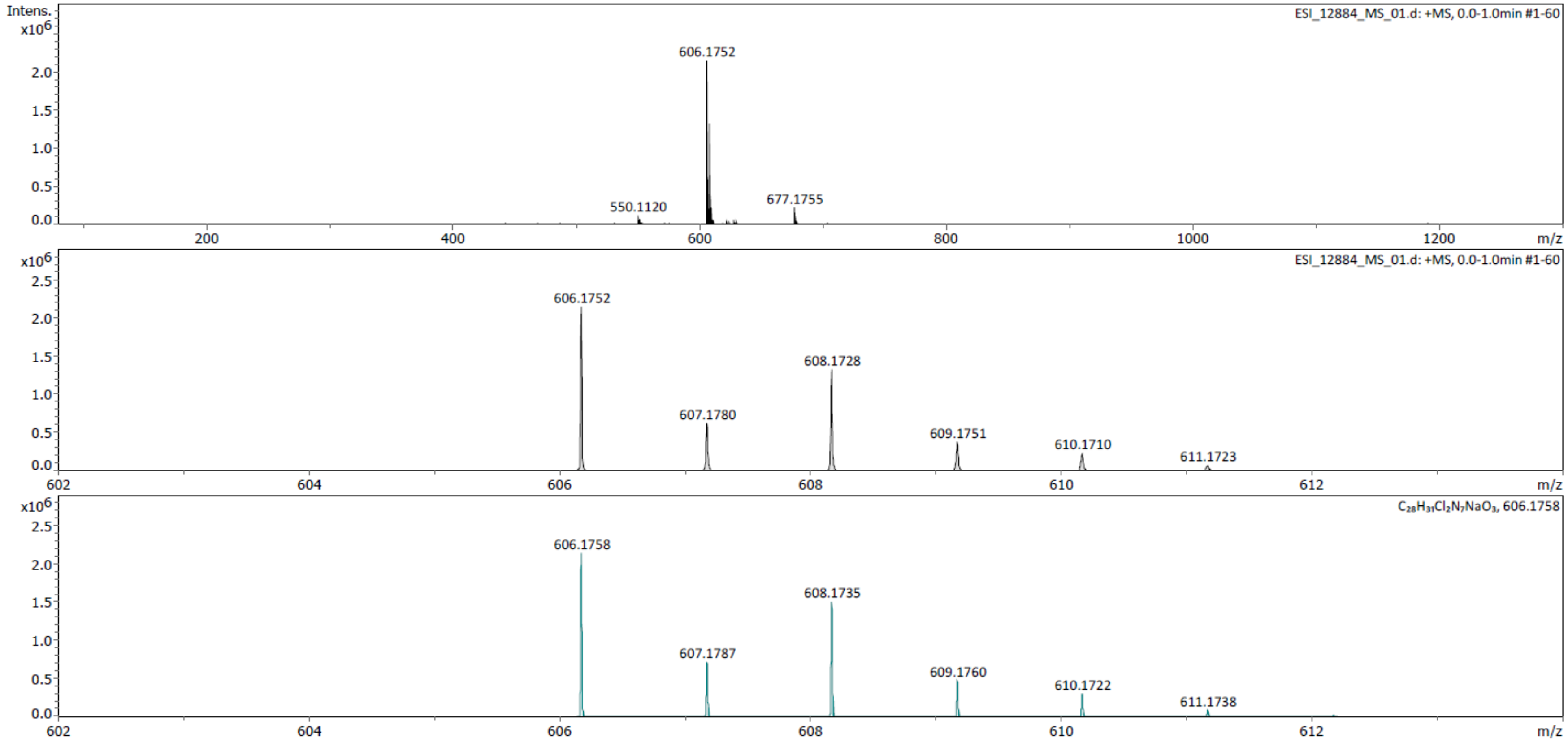

Supplement: Supplementary file 1 [file molecules-27-06149-s001.zip › 2022 DB3 Molecules SI part 2.pdf]
